# Analysis of *Arabidopsis thaliana* Redox Gene Network Indicates Evolutionary Expansion of Class III Peroxidase in Plants

**DOI:** 10.1038/s41598-019-52299-y

**Published:** 2019-10-31

**Authors:** Raffael Azevedo de Carvalho Oliveira, Abraão Silveira de Andrade, Danilo Oliveira Imparato, Juliana Gabriela Silva de Lima, Ricardo Victor Machado de Almeida, João Paulo Matos Santos Lima, Matheus Augusto de Bittencourt Pasquali, Rodrigo Juliani Siqueira Dalmolin

**Affiliations:** 1 Bioinformatics Multidisciplinary Environment - IMD, Federal University of Rio Grande do Norte, Natal, Brazil; 2 Department of Biochemistry, Federal University of Rio Grande do Norte, Natal, Brazil; 3 Institute of Tropical Medicine, Federal University of Rio Grande do Norte, Natal, Brazil; 4 Food Engineering Unit, UAEALI, UFCG, Campina Grande, Brazil; 5 Graduate Program in Natural Resources, PPGRN, UFCG, Campina Grande, Brazil

**Keywords:** Evolution, Plant evolution, Evolvability

## Abstract

Reactive oxygen species (ROS) are byproducts of aerobic metabolism and may cause oxidative damage to biomolecules. Plants have a complex redox system, involving enzymatic and non-enzymatic compounds. The evolutionary origin of enzymatic antioxidant defense in plants is yet unclear. Here, we describe the redox gene network for *A. thaliana* and investigate the evolutionary origin of this network. We gathered from public repositories 246 *A. thaliana* genes directly involved with ROS metabolism and proposed an *A. thaliana* redox gene network. Using orthology information of 238 Eukaryotes from STRINGdb, we inferred the evolutionary root of each gene to reconstruct the evolutionary history of *A. thaliana* antioxidant gene network. We found two interconnected clusters: one formed by SOD-related, Thiol-redox, peroxidases, and other oxido-reductase; and the other formed entirely by class III peroxidases. Each cluster emerged in different periods of evolution: the cluster formed by SOD-related, Thiol-redox, peroxidases, and other oxido-reductase emerged before opisthokonta-plant divergence; the cluster composed by class III peroxidases emerged after opisthokonta-plant divergence and therefore contained the most recent network components. According to our results, class III peroxidases are in expansion throughout plant evolution, with new orthologs emerging in each evaluated plant clade divergence.

## Introduction

Reactive oxygen species (ROS) are byproducts of normal metabolism of aerobic organisms, being produced in a constitutive and not controlled manner during photosynthesis in plants and during respiration in most living beings. External agents such as UV radiation, pathogens, extreme temperatures, pollution, and xenobiotics can contribute to ROS production. Organisms have developed antioxidant systems during evolution to avoid ROS toxicity. Plants are constantly exposed to stressing agents, mainly due to their sessile nature. Many of those agents can imbalance ROS production, leading to oxidative stress^1^. In spite of their toxic trait, ROS can be useful in plants acting as signaling molecules^2–4^ and in defense against pathogens^5,6^. Enzymes such as RBOH (Respiratory Burst Oxidase Homologue), a homologous enzyme of mammalian NADPH-oxidase, as well as some peroxidases, are responsible for producing H_2_O_2_ in plants^7,8^. H_2_O_2_ has several roles in plant physiology as signaling for cell expansion^9^, seed germination^10^, stomatal closure^11^, and hormone production^12^. Additionally, H_2_O_2_ has an important role in cell wall reinforcement since it is involved with lignin synthesis^7^. The balance between ROS production and scavenging should be spatially and temporally regulated in plants to avoid oxidative damage and to allow ROS-mediated cell signaling^13,14^.

Plants have developed complex antioxidant machinery comprising enzymatic and non-enzymatic mechanisms to protect against ROS overproduction^15^. The non-enzymatic antioxidant machinery involves phenolic compounds, ascorbate, tocopherols, carotenoids, flavonoids, abscisic and jasmonic acids, forming a defense line against ROS^16^. The non-enzymatic defenses act coordinately with several antioxidant enzymes, such as catalase (CAT), peroxidases (PER), superoxide dismutases (SOD), and other proteins with no catalytic activity, to maintain redox homeostasis^17^. Both enzymatic and non-enzymatic defenses work together in many known reactions, like the glutathione-ascorbate cycle, characterizing a redox-buffer system^18^. Peroxidases (EC 1.11.1.X) have a central role in plant metabolism. Besides hydrogen peroxide scavenging, peroxidases can act in H_2_O_2_ generation and are essential players in auxin catabolism, suberization, programmed cell death, defenses against pathogens, and lignification of cell wall^19–21^, which might have been a critical evolutionary step on land colonization. Antioxidant and ROS-generating enzymes work together in a redox network to coordinately maintain redox homeostasis in plants^22,23^.

The evolutionary origin of plant redox systems is a matter of debate. Antioxidant defenses are thought to date back Paleoproterozoic Great Oxidation Event or earlier^24,25^. Many antioxidant enzymes found in plants have orthologs in fungi and metazoan^25–27^. Plants, however, have particularities regarding redox metabolism since ROS perform many specific functions in plants as mentioned before. It is reasonable to think that plant redox gene network differs from other eukaryotic groups and those differences might be crucial, for instance, to plant acclimation to stressing conditions^27^. There are three peroxidase groups in eukaryotes: (*i*) class I peroxidases, widely distributed within eukaryotes; (*ii*) class II peroxidases, exclusive to fungi; and (*iii*) class III peroxidases, exclusive to plants^21,26^. Several class III peroxidase isozymes have been described in different angiosperms such as *A. thaliana, Oryza sativa*, and *Zea mays*^21^. The vast number of plant-exclusive peroxidases found in angiosperms brings clues about its importance in the expansion of redox system in plants.

Here, we propose the plant redox gene network using the model organism *Arabidopsis thaliana*. Using PeroxiBase, Gene Ontology, and TAIR database, a reference database for *A. thaliana*, we identified 323 genes directly involved with ROS metabolism, being 246 of them present in the network main connected component. The network, based on protein-protein and protein-chemical interactions, presented two clusters: one composed by Thiol-redox proteins, SOD-related proteins, peroxidases, and other oxido-reductase, and the other formed exclusively by class III peroxidases. We used orthology information regarding 238 eukaryotes to infer the evolutionary root of each gene to reconstruct the evolutionary history of the *A. thaliana* redox gene network. We found class III peroxidases as the most recent components of the network, showing that class III peroxidases cluster is in expansion in plants.

## Results

### *A*. thaliana redox gene network

**Figure 1:**
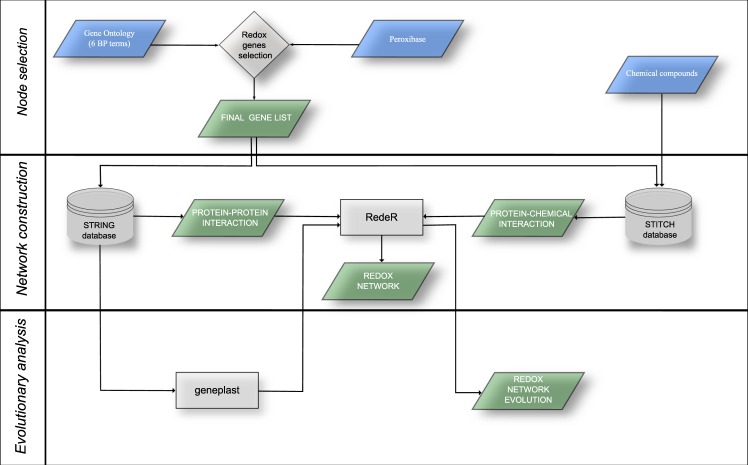
Analysis workflow. Redox genes were selected from Gene Ontology and PeroxiBase and then manually curated (Node selection). Protein-protein interactions were obtained from STRING database, protein-chemical interactions were obtained from STITCH database, and the final network was handled using RedeR Bioconductor package (Network construction). The evolutionary root of each *A. thaliana* antioxidant gene was inferred using geneplast Bioconductor package (Evolutionary analysis).

**Figure 2:**
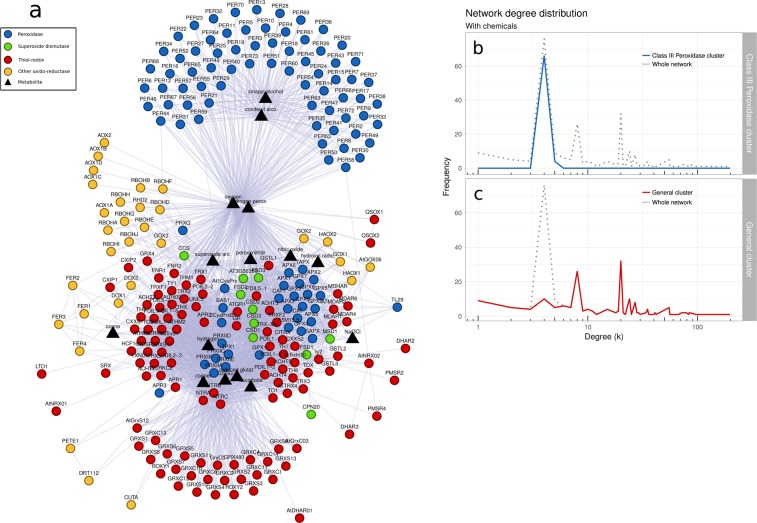
*Arabidopsis thaliana* redox gene network. (**a**) Protein-protein interaction and protein-chemical interaction among 246 *A. thaliana* redox genes (circle nodes) and 15 antioxidant enzyme subtracts and/or products (triangle nodes). Node color indicates protein class: peroxidase (blue), superoxide dismutase (green), thiol-redox (red), and other oxido-reductase (yellow). Chemical nodes are shown in black. (**b**) Degree distribution (red line) of proteins from *class III peroxidase cluster*. The dotted line shows degree distribution of the whole network, including chemical compounds. (**c**) Degree distribution (blue line) of proteins from the *general cluster*. The dotted line shows degree distribution of the whole network, including chemical compounds.

*A. thaliana* redox genes were selected according to two criteria: using GO consortium and PeroxiBase (Fig. 1). For the first criteria, we selected genes from six GO terms associated with redox metabolism, and for the second criteria, we selected *A. thaliana* genes annotated in PeroxiBase. The merged gene list included 323 genes. Among them, 246 were connected to the network main connected component, and 77 genes presented no connection when following the parameters described above, including *PER48*, *PER1*, and *PER62* (Supplementary Table S3). The final network includes 246 genes and 15 chemical compounds directly involved with the redox enzymes, being their subtracts or products (Fig. 2a).

The result is a metabolic network involving protein-protein interaction and protein-chemical interaction. Antioxidant systems are described to work coordinately to maintain physiological redox status, acting as an antioxidant-defense network^22,28,29^. Those properties can be clearly observed in Fig. 2a by the entanglement of the network components.

Genes were classified according to their function: 106 thiol-redox, 100 peroxidases, 10 SOD-related genes, and 30 other oxido-reductase (including RBOH isoforms, ferritin isoforms, and alternative oxidases). According to Fig. 2a, *A. thaliana* redox gene network consists of two clusters with distinct functions. The cluster on the bottom is formed by SOD-related, thiol-redox, peroxidases, and other oxido-reductase, totalizing 176 genes. From now on we will refer to the bottom cluster as *general cluster*. The cluster on the top is composed entirely by class III peroxidase genes, totalizing 70 genes. We will refer to the top cluster as c*lass III peroxidase cluster*. The nodes in the *general cluster* are very interconnected while genes in the *class III peroxidase cluster* are linked mainly by the substrates/products O_2_, H_2_O_2_, coniferyl, and sinapyl (Fig. 2b,c and Supplementary Fig. S1). O_2_ and H_2_O_2_ work as hubs linking the two clusters. Hydrogen peroxide is known to perform several physiological functions in plants and its central position in the network denotes its importance in redox plant metabolism.

### Evolutionary Origin Of *A. thaliana* Redox Gene Network

**Figure 3:**
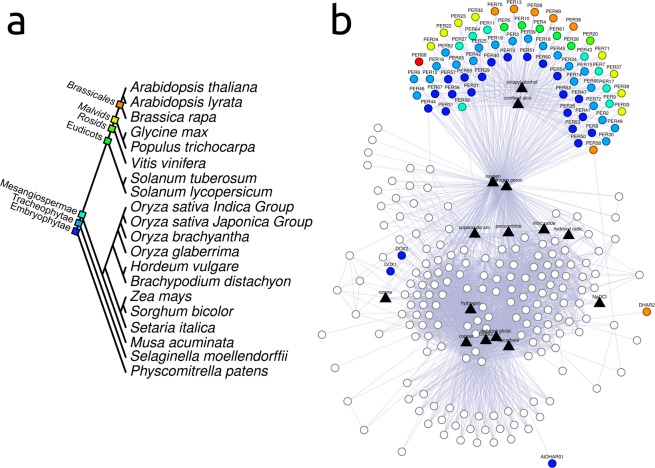
Evolutionary root of *A. thaliana* class III peroxidases orthologs. (**a**) Evolutionary tree of 20 land plants used in the rooting analysis. Colored diamonds indicate the last common ancestral of each taxonomic group. (**b**) *A. thaliana* redox gene network showing the inferred evolutionary root of each gene on the network. Node color represents the evolutionary root of each gene, according to the evolutionary tree shown in A. Genes which the inferred evolutionary root was placed before plant divergence are shown in white. It was not possible to determine the evolutionary root of *PER68* (shown in red).

While proteins within the *general cluster* are found in other eukaryotes (*e.g*., SOD, GPX, and other peroxidases), class III peroxidases are exclusive to plants. We then assessed the evolutionary root of each redox gene to investigate the evolutionary origin of *A. thaliana* redox gene network. The idea is to identify the most ancient genetic archetype of each *A. thaliana* redox gene that has been vertically inherited. To do that, we looked for *A. thaliana* redox gene orthologs in 238 eukaryotes (see Supplementary Fig. S2). According to our results, the majority of the genes within the *general cluster* emerged before Viridiplantae divergence (Fig. 3). Except for two oxido-reductase (*DOX1* and *DOX2*) and two thiol-redox (*DHAR2* and *DHAR01*), all the other thiol-redox protein-coding genes, all gluthatione peroxidases, ascorbate peroxidases, catalases, thioredoxin-dependent peroxidases, superoxide dismutases isoforms (Mn, Cu-Zn, and Fe), and their cofactor-supplier proteins (ATCCS and CPN20) compose the ancient portion of the network. *Naegleria gruberi* and *Giardia lamblia* are the most phylogenetically distant species from *A. thaliana* in our species set (Supplementary Fig. S2). At total, 146 proteins of the *general cluster* have orthologs rooted at the divergence among *Naegleria gruberi/Giardia lamblia and A. thaliana*. It represents that more than 80% of the *general cluster* is rooted at the origin of eukaryotes. Additionally, only 5% of the *general cluster* arrived after the divergence among opisthokonta and plants. These results suggest that *general cluster* is the ancient part of *A. thaliana* redox network (for a detailed analysis of the *general cluster* evolution, please see Supplementary Figs S2 and S3).

On the other hand, all *A. thaliana* class III peroxidases orthologs emerged at Viridiplantae divergence or afterwards. It is important to mention that *PER68* is not assigned in any orthologous group (OG) on STRINGdb, so it was not possible to determine its evolutionary root following the strategy used here. This might be a result of the COG algorithm, which needs a triangulation among at least three species to build a cluster of orthologs. Accordingly, proteins found in less than three species do not take part in an COG. We found 20 class III peroxidase genes rooted at Viridiplantae divergence, 21 rooted at tracheophytes divergence, 8 at angiosperms divergence, 5 at eudicots divergence, 1 at rosids divergence, 8 at malvids divergence, and 6 at brassicales divergence (Fig. 3 and Supplementary Fig. S4).

### Class III Peroxidase Abundance

**Figure 4:**
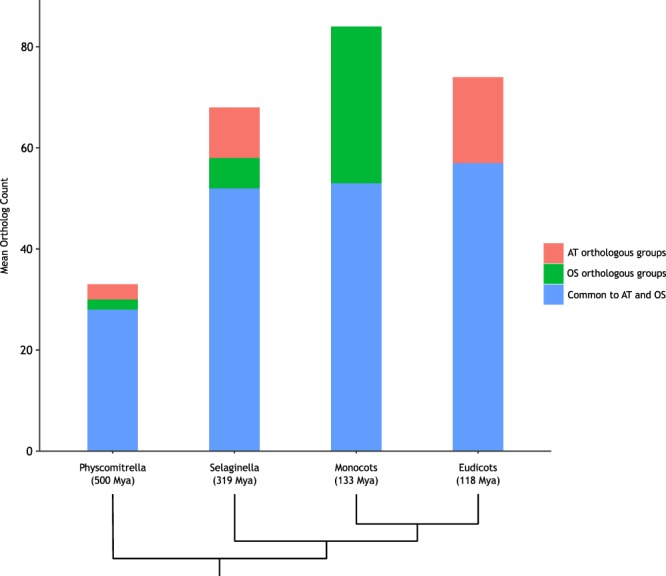
Class III peroxidase orthologs abundance. Bars indicate the number of class III peroxidase isozymes by species of each taxonomic group (Bryophyta - *Physcomitrella patens*, Lycopodiophyta - *Selaginella moellendorffii*, Eudicots - 8 species, and Monocots - 10 species). Colors indicate whether the OG have *A. thaliana* orthologs (AT orthologous groups - pink), *O. sativa* orthologs (OS orthologous groups - green) or orthologs from both species (Common to AT and OS - blue). The dendrogram at the bottom shows the evolutionary relationship among the groups. Estimated divergence times are shown.

As mentioned before, class III peroxidases are very abundant in *A. thaliana*. We evaluate the abundance of class III peroxidases orthologs in the different plant taxonomic groups available in our dataset. We assessed two sets of orthologous groups, *A.thaliana* class III peroxidases OGs and *Oryza sativa* class III peroxidases OGs (see methods), to cover the OGs distribution in the two main monophyletic groups of angiosperms: monocots and eudicots. Figure 4 shows the average number of class III peroxidases orthologs per species in eudicots (8 species), monocots (10 species), *Selaginella moellendorffii*, and *Physcomitrella patens*. *Physcomitrella patens*, a non-vascular plant, have roughly half of class III peroxidases when compared with vascular plants, including the non-seed vascular plant *Selaginella moellendorffii* (Fig. 4). To avoid a possible bias due to the differences in the number of genes in each evaluated species, we normalized class III peroxidases OGs abundance by the number of genes of each species, finding basically the same results: *P. patens* have roughly half of class III peroxidases when compared with vascular plants even taking into account the number of genes in each species (Supplementary Fig. S4). The distribution pattern is similar in OGs shared by *A. thaliana* and *O. sativa*. Angiosperms have more class III peroxidases per species when compared to *S. moellendorffii*. However, it is difficult to draw conclusions about class III peroxidases distribution since we have only one Moss and one Lycopodiopsida (*i.e., P. patens and S. moellendorffii*, respectively) in our dataset. Focusing on angiosperms, we can observe a high number of class III peroxidases orthologs found in eudicots which are not found in monocots and vice versa. Additionally, we can observe a higher number of class III peroxidases in monocots when compared to eudicots (Fig. 4).

Using PeroxiBase information, we redid the analysis including *Marchantia polymorpha*, a species not represented in the dataset used here. According to PeroxiBase, *M. polymorpha* has 167 class III peroxidase distributed in 23 OGs, 8 of which containing orthologs from both *A. thaliana* and *O. sativa*, 1 OG containing orthologs from *A. thaliana* (with no *O. sativa* ortholog) and 1 OG containing orthologs from *O. sativa* (with no ortholog of *A. thaliana*). We used *M. polymorpha* OGs inferred by PeroxiBase since *M. polymorpha* is not among the 238 eukaryotes present in STRING. At total, *M. polymorpha* has 23 class III peroxidases that were identified as ortholog of *A. thaliana* class III peroxidases in PeroxiBase. When compared to *Physcomitrella patens*, *M. polymorpha* has a similar number of class III peroxidases which have orthologs in *A. thaliana* and/or *O. sativa*. However, there are too few species of basal plants in our sample to allow more conclusive results (Supplementary Fig. S5).

## Discussion

Plants are constantly exposed to stressing conditions and have developed mechanisms to deal with stress compounds. Oxidant compounds are recognized as being one of the most important stressing agents in plants^30,31^. On the other hand, ROS are also extensively known to take part in several physiological processes, being indispensable for plants^13,23,32^. Plant redox system should be able to finely tune cellular redox status to deal with the double-edged sword behavior of oxidant compounds correctly^23^. Here, we reconstructed the redox network of *A. thaliana* and tracked down the evolutionary roots of its genes to identify the origin of genetic redox system in plants.

In order to depict the *A. thaliana* redox network, we gathered canonical protein-protein interaction (PPI) and protein-chemical interaction involving the main redox enzyme substrates and/or products. A similar strategy has been used by Gelain and collaborators to describe the human antioxidant gene network^28^. According to the authors, antioxidant proteins act coordinately, forming an antioxidant buffer. Therefore, it is necessary to evaluate their connections among each other, as well as the connections with their respective subtracts and/or products, to get a complete perspective of the redox gene network^28^. In 2004, Mittler and collaborators proposed a reactive oxygen gene network of plants^22^. They gathered both ROS-producer and ROS-scavenger protein-coding genes that are part of an extensive redox system. They proposed that the plant ROS network would have about 150 genes, including all ROS-producing RBOH isozymes and other oxidases. Here, we found 60% more genes when compared with Mittler’s results. It is important to note that the work conducted by Mittler and collaborators did not include class III peroxidases, in spite of the authors have assumed that the so-called *classical plant peroxidases* act in hydrogen peroxide scavenging^22^. Although some authors have described the genes involved with redox homeostasis in plants, the network-based analysis proposed here allowed the identification of *class III peroxidase cluster*, which is specific to plants and consequently evolved after opisthokonta-plant divergence. According to our results, modern plants share the redox enzymatic core (*e.g*., SOD, glutathione peroxidases, and other peroxidases and oxido-reductases) with other Eukaryotes. This agrees with literature where antioxidant enzymes, such as SOD, CAT, and several Thiol-redox, have been described as ancient^21,33,34^. Some of those genes, like the Fe-SOD, could be found in Archea^35^ and other proteins known as antioxidant in modern plants emerged before the great oxidation event^25,36^. Even ascorbate peroxidase (APX) enzymes, which are central to plant redox homeostasis, are known to have evolved before plant divergence and are well described in ancestral taxonomic groups such as Trypanosomatidae^27^.

On the other hand, we showed that *A. thaliana* class III peroxidase orthologs have emerged at opisthokonta-plant divergence and the group has increased during plant evolutionary expansion since it was possible to identify the emergence of new class III peroxidases COGs in each evaluated plant clade divergence. Inupakutika and collaborators investigated the evolution of plant ROS gene network, focusing SOD, APX, and RBOH genes. They evaluated 11 genes, involving ROS generator and ROS scavenger genes, in 33 species, including 30 eukaryotes and 6 plants. The authors found SOD, peroxiredoxins (PRXR), CAT, and APX as ancient genes, shared by many taxonomic groups^25^. We have very similar results regarding the antioxidant core (*i.e*., the *general cluster*). However, we used a different methodology designed to evaluate entire genetic systems, allowing to investigate the evolutionary origin of all 246 *A. thaliana* redox genes present in the network main connected component and using 238 eukaryotes as sample space.

Besides the importance of recognizing the core antioxidant enzymes (*i.e*., SOD, CAT, PRXRs, APX, other class I peroxidases, and other oxido-reductases) as ancient, our results suggest class III peroxidase as particularly important to plants. Not just because they are plant-exclusive, but also due to their evolutionary expansion throughout plant phylogeny (Figs 3 and 4). Studies made in Chinese pear and maize shows that class III peroxidase genes underwent positive selection^37,38^. Additionally, the fact that 72 isozymes fit in 44 different orthologous groups also corroborates with the complexity related to this class of protein^39^. Our result corroborates with many evolutionary analyses made for class III peroxidases^40–42^, as well as with the theory that some class III peroxidases isozymes emerged more likely at the beginning of land colonization by plants^43^, in spite of some works having described class III peroxidases members in algae^26,44^.

Class III peroxidases are intimately related with H_2_O_2_ dynamics in plants since this class of peroxidases works in H_2_O_2_ detoxification and formation, including those which have lignification as primary function^45^. H_2_O_2_ is crucial to plant redox signaling, defense against pathogens, and is potentially toxic as ROS^46^. Class III peroxidases are intimately involved with lignin polymerization, but we found class III peroxidases orthologs that emerged with Embryophyta divergence. Accordingly, it is difficult to say if class III peroxidases emergence was related to lignification or to deal with increased ROS in an atmosphere with high oxygen concentration during land colonization by plants^8,44^. Only at Tracheophyta divergence, other class III peroxidase emergence seems to be related to lignification and suberization processes^8^.

The results shown here improve the comprehension of the evolutionary dynamics of the redox gene network in plants and suggest an essential role of class III peroxidases on plant ROS metabolism since *class III peroxidase cluster* seems to evolve continuously throughout plant evolution. It is important to mention that enzymatic antioxidant defenses are just part of the whole redox machinery. Plants have complex biochemical pathways to produce non-enzymatic antioxidant compounds, such as carotenoids and flavonoids, and those molecules are crucial to redox metabolism. The genetic network proposed here allowed to identify classical antioxidant enzymes, such as SOD, CAT, glutathione peroxidases (GPX), etc., as ancient and class III peroxidases as the novelty regarding redox plant metabolism. Taking together, the results herein suggest that class III peroxidases have a pivotal role in plant redox homeostasis and the expansion of redox network throughout plant evolution are coincident with the continuous emergence of class III peroxidases new orthologs.

## Methods

### Arabidopsis redox gene selection

*A. thaliana* redox genes were selected using GO consortium^47^ and PeroxiBase^48^, only including genes also annotated on TAIR database (https://www.arabidopsis.org/)^49^. We selected *A. thaliana* redox genes from six GO terms associated with redox metabolism: (i) *oxygen species metabolic process* (GO:0072593), (ii) *cell redox homeostasis* (GO:0045454), (iii) *cellular response to reactive oxygen species* (GO:0034614), (iv) *response to inorganic substance* (GO:0010035), (v) *oxidation-reduction process* (GO:0055114), and (vi) *response to oxidative stress* (GO:0006979). Gene function was checked in TAIR database (https://www.arabidopsis.org/)^49^. Genes were then manually curated according to the criteria previously established by Gelain and collaborators^28^. Briefly, the selection includes genes whose products are described as primarily antioxidant scavengers as well as genes indispensable to antioxidant enzymes activity. The objective was to include only gene products that directly deal with oxidants. The resulting gene set was manually curated to identify 208 genes directly involved with redox homeostasis (Supplementary Table S1 and S3).

We also collected *A. thaliana* genes annotated in PeroxiBase (now named as RedoxiBase, since the name has changed during the editorial processes). PeroxiBase is a manually curated database dedicated mainly to peroxidase superfamilies, including other proteins known to deal with ROS^48^. Gene function was checked in TAIR database and the final PeroxiBase gene list content reached 293 genes (Supplementary Table S1 and S3).

The final gene set includes 323 genes (see Supplementary Table S1 and S3). The genes were classified into four categories according to the TAIR database description: thiol-redox, peroxidase, SOD, and other oxido-reductase.

### Redox gene network

*A. thaliana* redox gene network was built using STRING database version 10.5 (https://version-10-5.string-db.org/)^50^ with input options ‘databases’, ‘experiments’, and 0.400 confidence level. STRING integrates different curated databases containing information on direct and indirect functional protein–protein associations. The search was carried out using TAIR id obtained from TAIR database and the connections among the 323 redox genes were collected. In addition to redox genes, the network also included chemical compounds that are substrates or products of the antioxidant enzymes. In total, were included 15 chemical compounds (see Supplementary Table S2). The connections involving the 15 chemical compounds and the 323 redox genes were retrieved from STITCH database version 5.0 (http://stitch.embl.de/)^51^ with input options ‘databases’, ‘experiments’, and 0.400 confidence level. STITCH integrates information regarding interactions between proteins and small molecules. 77 genes presented no connection when following the parameters described above (Supplementary Table S3). The resultant network includes protein-protein interactions as well as protein-chemical interactions among 246 *A. thaliana* redox genes and the 15 redox-related chemical compounds (Supplementary Fig. S2). The final network was handled using RedeR, an R package for interactive visualization and manipulation of networks^52^.

### Evolutionary rooting analysis

The evolutionary rooting analysis was carried out using geneplast, an R package designed to perform evolutionary rooting based on the distribution of orthologous groups, set with default parameters^53^. The method uses orthology information to infer the probability that a feature had been present in each Last Common Ancestor of a given species tree. The problem was previously addressed by Mirkin^54^ and Castro^55^. Concerning our investigation, the task is: to find its earliest ortholog in the eukaryote phylogeny for each orthologous group associated with the *A. thaliana* redox network. Orthology information of the 246 *A. thaliana* redox genes was retrieved from clusters of orthologous groups (COGs) from STRINGdb v10.5^50^. The analysis was performed using 238 eukaryotes available in STRINGdb (Supplementary Fig. S1). In total, 119 orthologous groups were identified for 245 of the 246 genes present in the network main connected component (Supplementary Table S1). The evolutionary trees describing the phyletic pattern of each redox gene are presented in Supplementary Material (see Supplementary Figs S6 to S125).

### Class III peroxidase abundance

The class III peroxidase genes abundance was calculated taking into account the orthologous genes from *A. thaliana* and/or *O. sativa* class III peroxidase genes in the 20 land plant species present on STRING database. *A. thaliana* class III peroxidase orthologous genes were collected as described above. From the original 119 redox *A. thaliana* OGs, 44 were annotated as having class III peroxidase proteins (see Supplementary Table S4). To identify *O. sativa* class III peroxidase OGs not shared with *A. thaliana*, we searched for *O. sativa* class III peroxidase genes in PeroxiBase^48^ and then translated to STRING format using UniProt^56^ and Ensembl Biomart^57^. At total, 53 *O. sativa* class III peroxidase OGs were used to retrieve the orthologous groups from STRING database (see Supplementary Table S5), which 29 were shared with *A. thaliana*.

## Supplementary information

Supplementary Information

Table S1

Table S2

Table S3

Table S4

Table S5

## Data Availability

The datasets analyzed during the current study are publicly available as Supplementary Material. Any other data/protocol is available from the corresponding author on request.
