# Supplementary material for: Analysis of Arabidopsis thaliana Redox Gene Network Indicates Evolutionary Expansion of Class III Peroxidase in Plants
Source: Sci Rep. 2019 Oct 31;9:15741. doi: 10.1038/s41598-019-52299-y (PMC6823369; doi:10.1038/s41598-019-52299-y)
Supplement: Supplementary file 1 — Supplementary Information [file 41598_2019_52299_MOESM1_ESM.pdf]

SUPPLEMENTARY INFORMATION

for

Analysis of *Arabidopsis thaliana* Redox Gene Network Indicates Evolutionary Expansion of Class III Peroxidase in Plants

Authors: Raffael Azevedo de Carvalho Oliveira[1,2]; Abraão Silveira de Andrade[1]; Danilo Oliveira Imparato[1]; Juliana Gabriela Silva de Lima[2]; Ricardo Victor Machado de Almeida[2]; João Paulo Matos Santos Lima[1,2]; Matheus Augusto de Bittencourt Pasquali[3,4,5]; Rodrigo Juliani Siqueira Dalmolin[1,2*]

[1]Bioinformatics Multidisciplinary Environment - IMD, Federal University of Rio Grande do Norte, Natal, Brazil;

[2]Department of Biochemistry, Federal University of Rio Grande do Norte, Natal, Brazil.

[3]Institute of Tropical Medicine, Federal University of Rio Grande do Norte, Natal, Brazil.

[4]Unidade Acadêmica de Engenharia de Alimentos, UAEALI, UFCG

[5]Programa de Pós-graduação em Recursos Naturais, PPGRN, UFCG

* Corresponding author

Rodrigo Juliani Siqueira Dalmolin

BioME – Bioinformatics Multidisciplinary Environment.

Address: Rua Odilon Gomes de Lima, 1722, Natal – RN. Brazil. Zip code 59078-400.

e-mail: rodrigo.dalmolin@imd.ufrn.br

Contents

SUPPLEMENTARY RESULTS AND DISCUSSION....3
Network clusters connectivity....3
Evolutionary roots of all network nodes....3
Class III peroxidase orthologs abundance....3
Marc*hantia polymorpha orth*ologs....4
Evolutionary trees of orthologous groups....4
SUPPLEMENTARY FIGURES LEGENDS....5
SUPPLEMENTARY TABLES LEGENDS....7

1. SUPPLEMENTARY RESULTS AND DISCUSSION

1.1. Network clusters connectivity

Supplementary Figure S1 shows the node degree distribution of the network clusters. In (a) is depicted node degree distribution of all proteins from *class III peroxidase* cluster (red continuous line) *versus* the node degree distribution of all proteins in the network (black dotted line), disregarding chemical compounds; In (b) is depicted node degree distribution of all proteins from *general* cluster (blue continuous line) *versus* the node degree distribution of all proteins in the network (black dotted line), disregarding chemical compounds. This result shows that connectivity is higher in the *general* cluster than *class III peroxidase* cluster; the latter cluster is composed solely by class III peroxidases, which have few connections between each other, whilst proteins of *general* cluster are well connected.

1.2. Evolutionary roots of all network nodes

In order to assess evolutionary roots of every gene present in the network, the *geneplast* R/Bioconductor was used. As described in Methods, it calculates the evolutionary root based on orthology information. Supplementary Figure S2 shows the eukaryote species tree from STRINGdb (a), and the redox network of *A. thaliana* (b). This figure is like the one presented in Figure 3, but with evolutionary root information about every gene in the analysis. Colored diamonds on root labels are linked to node color in the network: color code range from blue (more ancient root) to orange (more recent root). It is noteworthy that almost all proteins that emerged in root 9 (which represents the last common ancestor of land plants) belong to the *class III peroxidase* cluster. Supplementary Figure S3 depicts a projection of the network, showing the emergence of new nodes at each root of the species tree. Most of the *general* cluster emerged at the roots of eukaryotes.

1.3. Class III peroxidase orthologs abundance

The following analysis were made to verify the abundance of class III peroxidases in the 20 species of land plants (present on STRINGdb v10). Supplementary Figure S4 shows a boxplot of class III peroxidase orthologs abundance, which was calculated by the mean of:

$$\frac{Number\ of\ CIII\ Prx\ orthologs}{Total\ gene\ count}$$

for each species. In (a) is depicted the abundance of class III peroxidase orthologs using *A. thaliana* ortholgs, and (b) the abundance using *O. sativa* orthologs. The results obtained indicates that the same pattern is present, using different sources of orthologous groups, which corroborates with the established hypothesis.

1.4. *Marchantia polymorpha* orthologs

Data regarding annotation of *M. polymorpha* class III peroxidases were obtained from PeroxiBase. Orthogroup annotation from PeroxiBase was used to obtain orthology information of *M. polymorpha.* We identified 8 orthogroups shared by *M. polymorpha, A. thaliana,* and *O. sativa*, containing 22 *M. polymorpha* orthologs; 1 orthogroup shared by *M. polymorpha* and *A. thaliana* (without any *O. sativa* ortholog), containing 1 *M. polymorpha* ortholog; and 1 orthogroup shared by *M. polymorpha* and *O. sativa* (without any *A. thaliana* ortholog), containing 2 *M. polymorpha* orthologs. The results are shown in Supplementary Figure S5.

1.5. Evolutionary trees of orthologous groups

Finally, Supplementary Figures S6-S125 shows the most parsimonious scenario for the 119 orthologous groups obtained in the analysis. Each tree represents an orthologous group, which contains one or more genes present in the network. All but PER68 (AT5G58400) were in the analysis.

2. SUPPLEMENTARY FIGURES LEGENDS

Supplementary Figure S1: Degree distribution of the network clusters, taking into account only protein-protein interaction. (a) Degree distribution of proteins from *class III peroxidase* cluster (red). Dotted lines shows degree distribution of whole network, disregarding chemical compounds. (B) Degree distribution (blue line) of proteins from *general* cluster. Dotted lines shows degree distribution of whole network, disregarding chemical compounds.

Supplementary Figure S2: Evolutionary scenario of *Arabidopsis thaliana* redox network. In (a) is depicted the eukaryote species tree from STRING v10.5. Each leaf node denotes an species and their phylogeny is drawn in the tree. Root nodes represents the last common ancestor (LCA) for each taxon. Colored diamonds aside each root node assign color for the network in (b). Supplementary Figure S2 (b) shows the same redox network, but with evolution scenario for all network nodes. Colors range from dark blue (more ancient) to dark orange (more recent). Red nodes have no annotation for evolutionary root.

Supplementary Figure S3: Projection of evolutionary roots in the network. This figure shows how the redox network of *A. thaliana* have arised and organized its connections. Network organization ranging from more ancient proteins (dark blue, root 19) to more recent proteins (root 3, dark orange). Red nodes have no annotation for evolutionary root, and therefore accounted for root 0.

Supplementary Figure S4: Class III peroxidase abundace. (a) Shows a boxplot of the class III peroxidase abundance in each taxon analyzed. Eudicots were analyzed using orthologous groups obtained from STRING v10, using *A. thaliana* class III peroxidases as query. (b) Shows a boxplot of the class III peroxidase abundance in each taxon analyzed. Monocots were analyzed using orthologous groups obtained from STRING v10, using *O. sativa* class III peroxidases as query.

Supplementary Figure S5: Class III peroxidase orthologs abundance. Bars indicate the abundance of class III peroxidase isozymes by species of each taxonomic group (*Marchantia polymorpha*, *Physcomitrella patens*, *Selaginella moellendorffii*, Eudicots - 8 species, and Monocots - 10 species). Colors indicate whether the OG have A. thaliana orthologs (AT orthologous groups - pink), O. sativa orthologs (OS orthologous groups - green) or orthologs from both species (Common to AT and OS - blue). Dendrogram at the

bottom shows the evolutionary relationship among the groups, and its estimated divergence times (in millions of years – Mya). Abundance is calculated as the mean of number of class III peroxidase orthologs divided by the gene count of each species.

Supplementary Figures S6-125: The figures S6 to S125 shows the evolutionary roots for the 119 COGs obtained in our analysis, built using package *geneplast*. In all figures, green circles represents COG present in that species, and gray circles, absence. Red-hollow diamond represents inferred root for a given COG and the D-score, which estimates stability of the inferred root. P-values are generated by permutation analysis.

3. SUPPLEMENTARY TABLES LEGENDS

Supplementary Table S1: This table brings description of all genes used in our analysis. First column indicates STRING ID for each protein; the second column indicates the locus ID for each protein; third column shows the alias obtained from STRING v10; fourth column shows alias obtained from PeroxiBase; fifth column shows the defined name shown in the networks; sixth column indicates protein class (whether is a peroxidase (PRX), thiol-redox (TR), SOD or other oxido-reductase); the seventh column indicates protein annotation obtained from STRING v10; eighth column indicates the orthologous group that each gene is part of; ninth column indicates the infered evolutionary root calculated by *geneplast* for each orthologous group; tenth column shows the D-score for each root; eleventh column shows the adjusted P-value of the *geneplast* analysis, and the twelfth column shows wether the gene was found only in the GO search (our), PeroxiBase search (peroxibase), or in both (intersect).

Supplementary Table S2: This table describes the 15 chemical compounds used in our network analysis. The first column indicates the chemical element name, followed in the second column by its formula. The third and fourth columns shows identifiers for different databases: KEGG and PubChem. Hydride and Ozone did not have entries in KEGG database, therefore have empty values.

Supplementary Table S3: This table shows information regarding the 77 genes that not connect with the network main component. The first column indicates STRING ID for each protein; the second column indicates the locus ID for each protein; third column shows the alias obtained from STRING v10; fourth column shows alias obtained from PeroxiBase; fifth column shows the defined name shown in the networks; sixth column indicates protein class (whether is a peroxidase (PRX), thiol-redox (TR), SOD or other oxido-reductase); the seventh column indicates protein annotation obtained from STRING v10; eighth column indicates the orthologous group that each gene is part of; ninth column indicates the infered evolutionary root calculated by *geneplast* for each orthologous group; tenth column shows the D-score for each root; eleventh column shows the adjusted P-value of the *geneplast* analysis, and the twelfth column shows wether the gene was found only in the GO search (our), PeroxiBase search (peroxibase), or in both (intersect).

Supplementary Table S4: This table provides information about gene abundance of the whole network, using orthology information obtained from *A. thaliana* OGs. The OGs were

divided according to the function related to. In total, 44 COGs class III peroxidase-related; 8 peroxidase-related; 4 SOD-related, 28 thiol-redox related, and 35 COGs related to other oxido-reductases. Each column represents one of the 20 land plant species. The STRING identifier for each species is provided above its name.

Supplementary Table S5: This table provides information of class III protein abundance, using orthology information obtained from *O. sativa* COGs. 44 COGs were used in this table. Each column represents one of the 20 land plant species, which are divided based on its taxonomic group. The STRING identifier for each species is provided above its name. The ortholog count, total of genes and the class III genes ratio is provided for each species in the last lines. This ratio is the total amount of class III orthologs divided by the total number of genes for each species.

Supplementary Figure S1

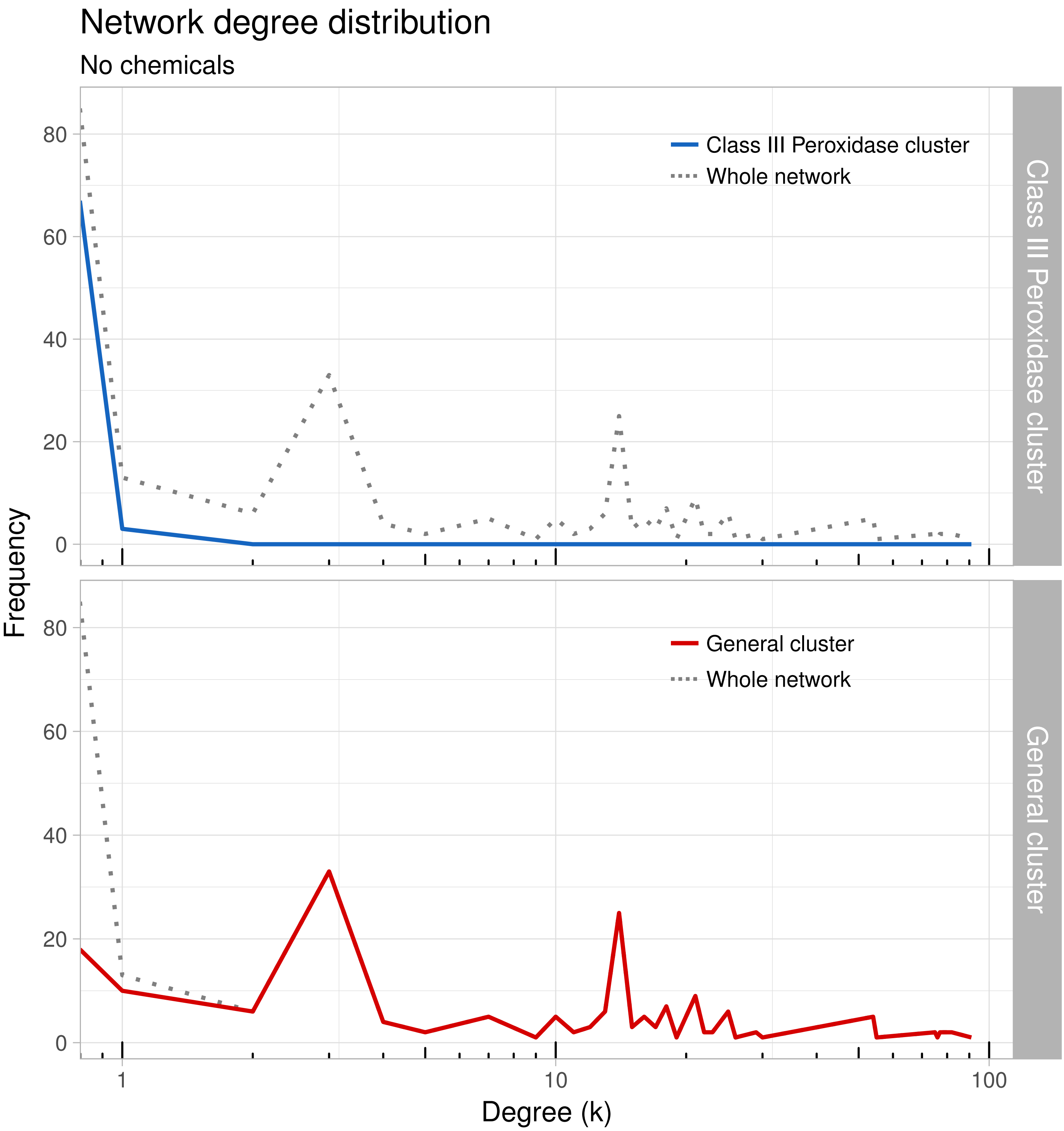

Supplementary Figure S1: Degree distribution of the network clusters, taking into account only protein-protein interaction. (a) Degree distribution of proteins from class III peroxidase cluster (red). Dotted lines shows degree distribution of whole network, disregarding chemical compounds. (b) Degree distribution (blue line) of proteins from general cluster. Dotted lines shows degree distribution of whole network, disregarding chemical compounds.

Supplementary Figure S2

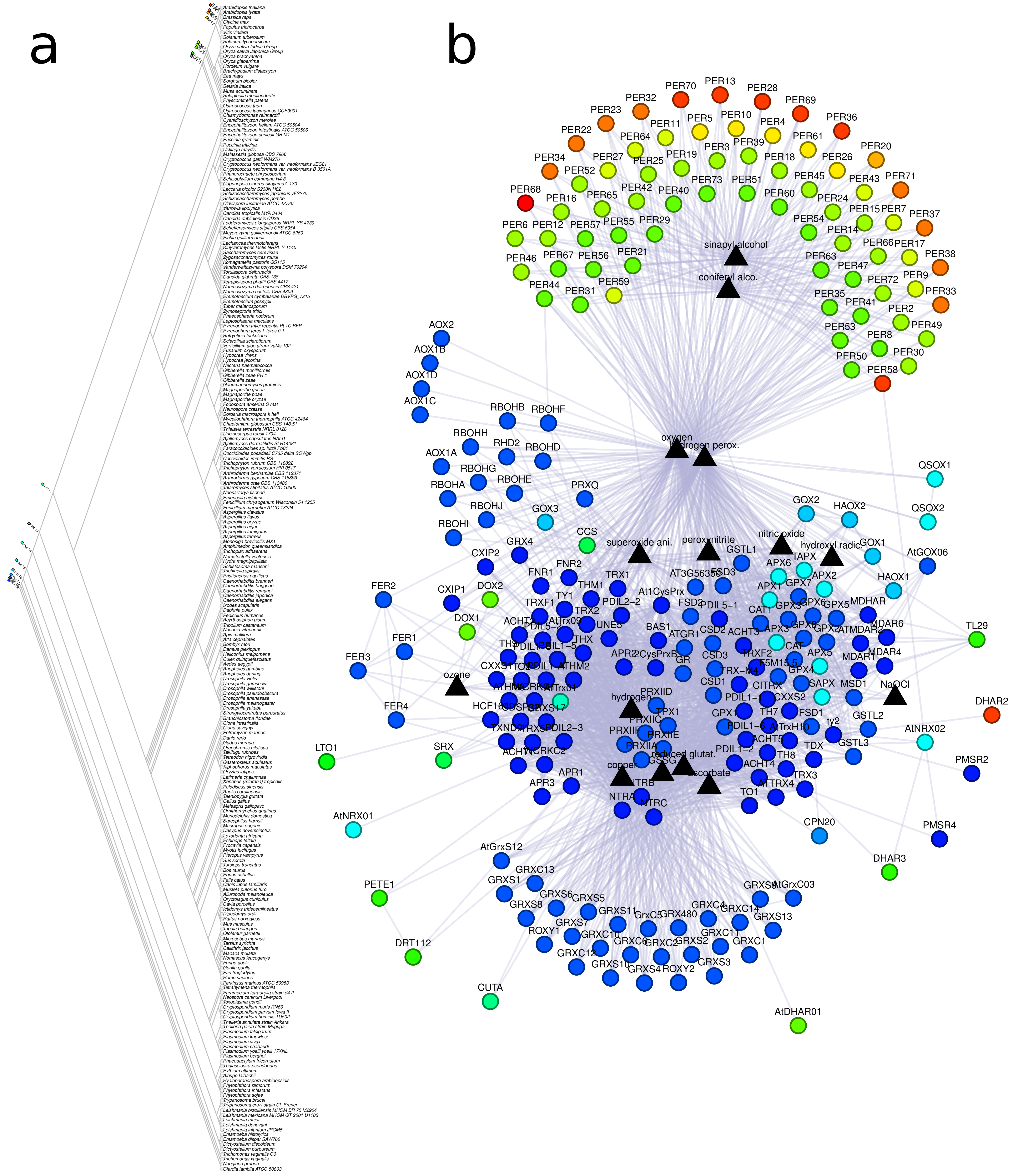

Supplementary Figure S2: Evolutionary scenario of Arabidopsis thaliana redox network. In (a) is depicted the eukaryote species tree from STRING v10.5. Each leaf node denotes an species and their phylogeny is drawn in the tree. Root nodes represents the last common ancestor (LCA) for each taxon. Colored diamonds aside each root node assign color for the network in (b). Supplementary Figure S2 (b) shows the same redox network, but with evolution scenario for all network nodes. Colors range from dark blue (more ancient) to dark orange (more recent). Red nodes have no annotation for evolutionary root.

Supplementary Figure S3

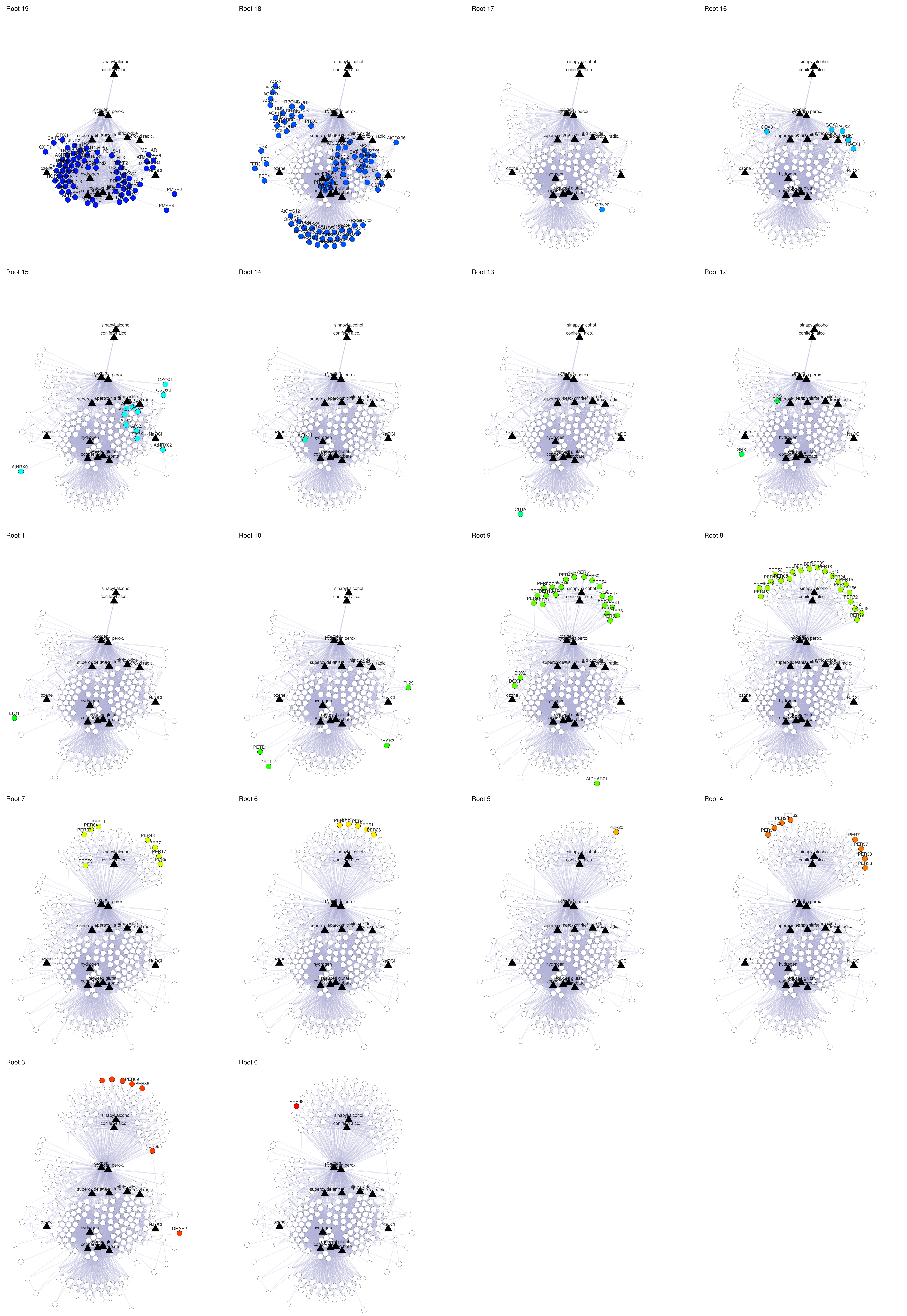

Supplementary Figure S3: Projection of evolutionary roots in the network. This figure shows how the redox network of A. thaliana have arised and organized its connections. Network organization ranging from more ancient proteins (dark blue, root 19) to more recent proteins (root 3, dark orange). Red nodes have no annotation for evolutionary root, and therefore accounted for root 0.

Supplementary Figure S4

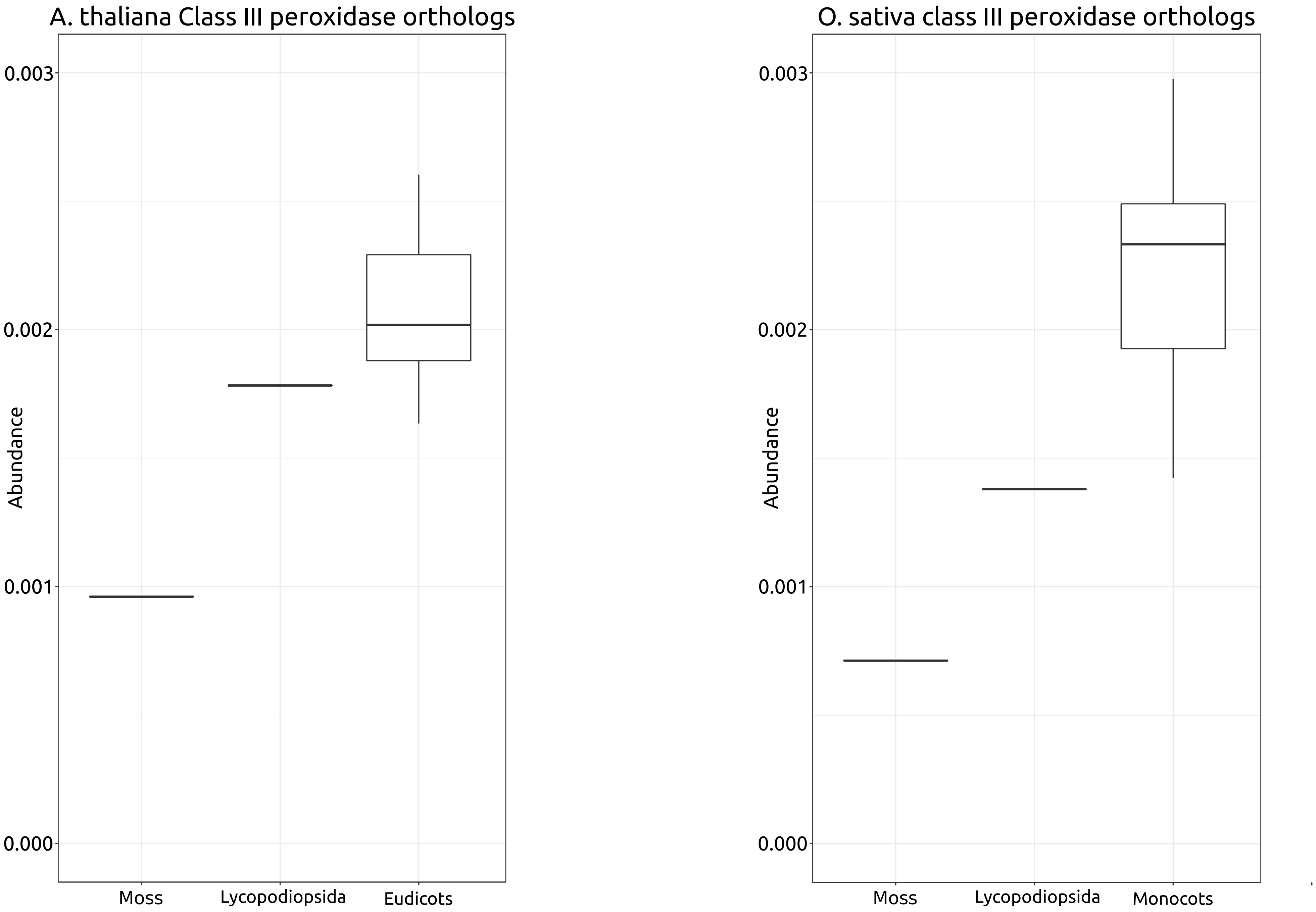

Supplementary Figure S4: Class III peroxidase abundace. (a) Shows a boxplot of the class III peroxidase abundance in each taxon analyzed. Eudicots were analyzed using orthologous groups obtained from STRING v10, using A. thaliana class III peroxidases as query. (b) Shows a boxplot of the class III peroxidase abundance in each taxon analyzed. Monocots were analyzed using orthologous groups obtained from STRING v10, using O. sativa class III peroxidases as query.

Supplementary Figure S5

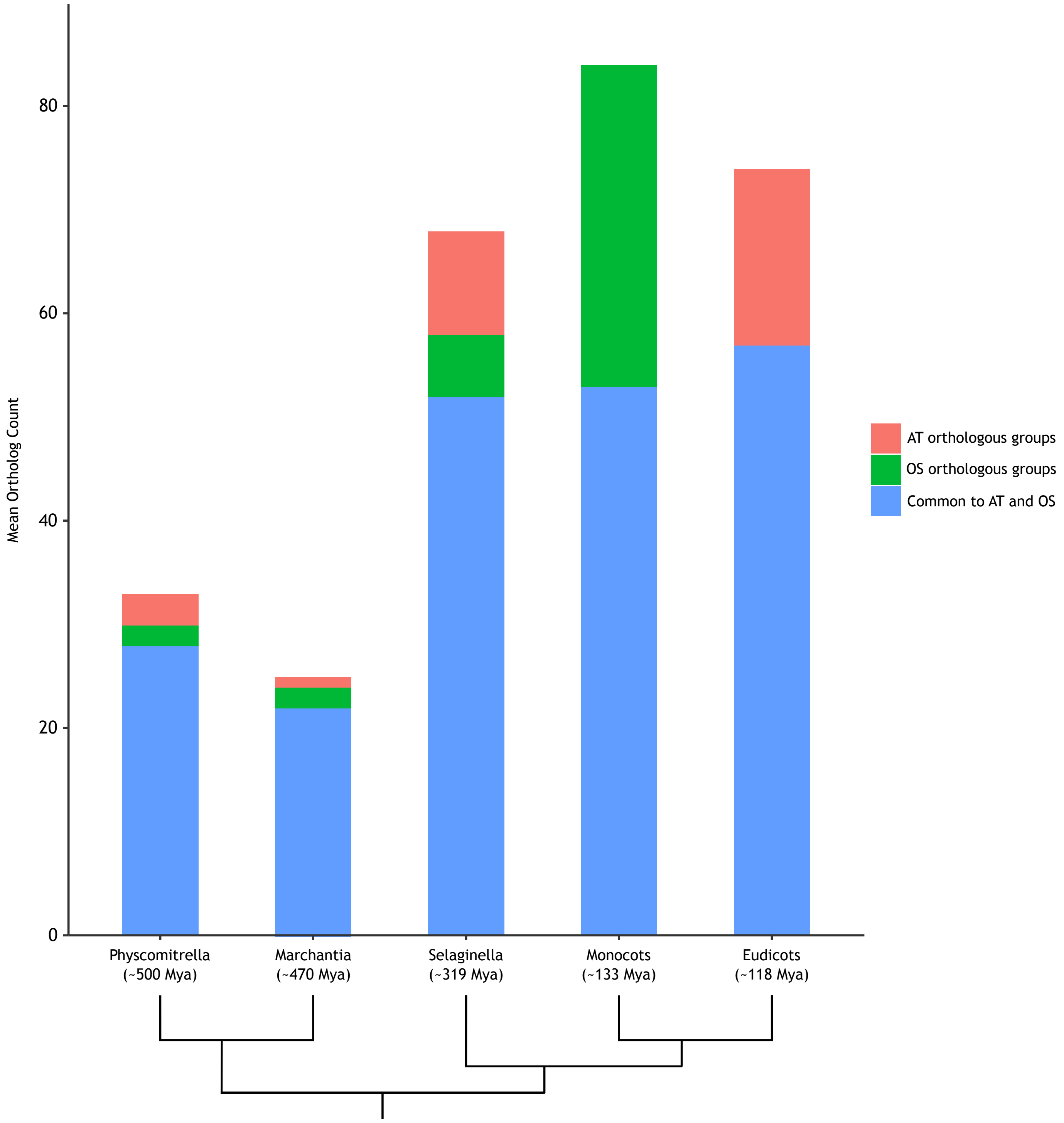

Supplementary Figure S5: Class III peroxidase orthologs abundance. Bars indicate the abundance of class III peroxidase isozymes by species of each taxonomic group (Marchantia polymorpha, Physcomitrella patens, Selaginella moellendorffii, Eudicots - 8 species, and Monocots - 10 species). Colors indicate whether the OG have A. thaliana orthologs (AT orthologous groups - pink), O. sativa orthologs (OS orthologous groups - green) or orthologs from both species (Common to AT and OS - blue). Dendrogram at the bottom shows the evolutionary relationship among the groups, and its estimated divergence times (in millions of years – Mya). Abundance is calculated as the mean of number of class III peroxidase orthologs divided by the gene count of each species.

COG0175

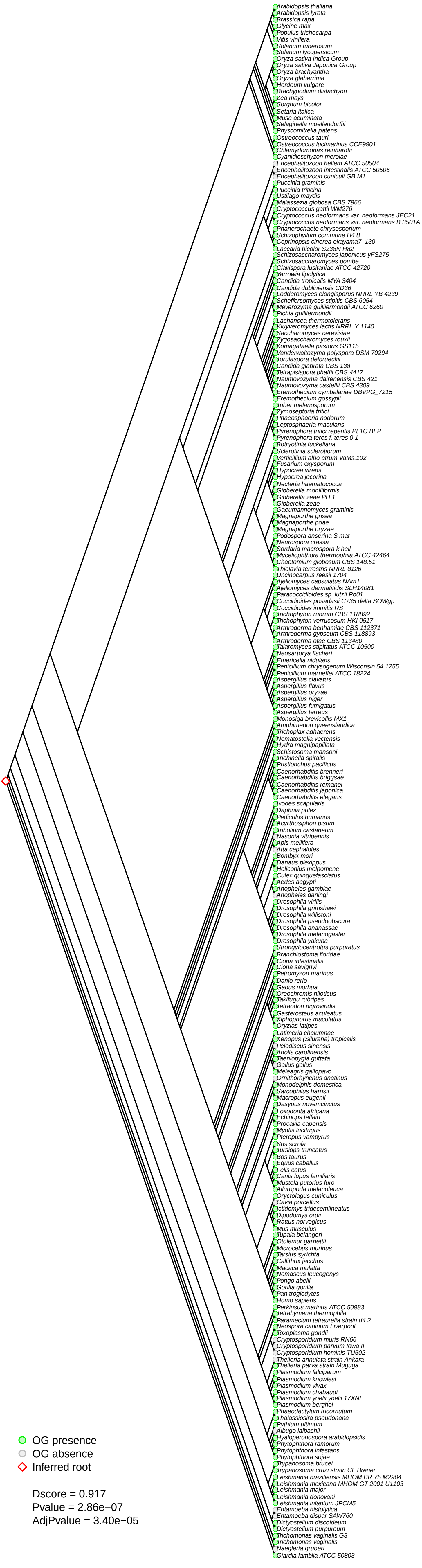

Arabidopsis thaliana
Arabidopsis lyrata
Brassica rapa
Glycine max
Populus trichocarpa
Vitis vinifera
Solanum tuberosum
Solanum lycopersicum
Oryza sativa Indica Group
Oryza sativa Japonica Group
Oryza brachyantha
Oryza glaberrima
Hordeum vulgare
Brachypodium distachyon
Zea mays
Sorghum bicolor
Setaria italica
Musa acuminata
Selaginella moellendorffii
Physcomitrella patens
Ostreococcus tauri
Ostreococcus lucimarinus CCE9901
Chlamydomonas reinhardtii
Cyanidioschyzon merolae
Encephalitozoon hellem ATCC 50504
Encephalitozoon intestinalis ATCC 50506
Encephalitozoon cuniculi GB M1
Puccinia graminis
Puccinia triticina
Ustilago maydis
Malassezia globosa CBS 7966
Cryptococcus gattii WM276
Cryptococcus neoformans var. neoformans JEC21
Cryptococcus neoformans var. neoformans B 3501A
Phanerochaete chrysosporium
Schizophyllum commune H4 8
Coprinopsis cinerea okayama7_130
Laccaria bicolor S238N H82
Schizosaccharomyces japonicus yFS275
Schizosaccharomyces pombe
Clavispora lusitaniae ATCC 42720
Yarrowia lipolytica
Candida tropicalis MYA 3404
Candida dubliniensis CD36
Lodderomyces elongisporus NRRL YB 4239
Scheffersomyces stipitis CBS 6054
Meyerozyma guilliermondii ATCC 6260
Pichia guilliermondii
Lachancea thermotolerans
Kluyveromyces lactis NRRL Y 1140
Saccharomyces cerevisiae
Zygosaccharomyces rouxii
Komagataella pastoris GS115
Vanderwaltozyma polyspora DSM 70294
Torulaspora delbrueckii
Candida glabrata CBS 138
Tetrapisispora phaffii CBS 4417
Naumovozyma dairenensis CBS 421
Naumovozyma castellii CBS 4309
Eremothecium cymbalariae DBVPG_7215
Eremothecium gossypii
Tuber melanosporum
Zymoseptoria tritici
Phaeosphaeria nodorum
Leptosphaeria maculans
Pyrenophora tritici repentis Pt 1C BFP
Pyrenophora teres f. teres 0 1
Botryotinia fuckeliana
Sclerotinia sclerotiorum
Verticillium albo atrum VaMs.102
Fusarium oxysporum
Hypocrea virens
Hypocrea jecorina
Necteria haematococca
Gibberella moniliformis
Gibberella zeae PH 1
Gibberella zeae
Gaeumannomyces graminis
Magnaporthe grisea
Magnaporthe poae
Magnaporthe oryzae
Podospora anserina S mat
Neurospora crassa
Sordaria macrospora k hell
Myceliophthora thermophila ATCC 42464
Chaetomium globosum CBS 148.51
Thielavia terrestris NRRL 8126
Uncinocarpus reesii 1704
Ajellomyces capsulatus NAm1
Ajellomyces dermatitidis SLH14081
Paracoccidioides sp. lutzii Pb01
Coccidioides posadasii C735 delta SOWgp
Coccidioides immitis RS
Trichophyton rubrum CBS 118892
Trichophyton verrucosum HKI 0517
Arthroderma benhamiae CBS 112371
Arthroderma gypseum CBS 118893
Arthroderma otae CBS 113480
Talaromyces stipitatus ATCC 10500
Neosartorya fischeri
Emericella nidulans
Penicillium chrysogenum Wisconsin 54 1255
Penicillium marneffei ATCC 18224
Aspergillus clavatus
Aspergillus flavus
Aspergillus oryzae
Aspergillus niger
Aspergillus fumigatus
Aspergillus terreus
Monosiga brevicollis MX1
Amphimedon queenslandica
Trichoplax adhaerens
Nematostella vectensis
Hydra magnipapillata
Schistosoma mansoni
Trichinella spiralis
Pristionchus pacificus
Caenorhabditis brenneri
Caenorhabditis briggsae
Caenorhabditis remanei
Caenorhabditis japonica
Caenorhabditis elegans
Ixodes scapularis
Daphnia pulex
Pediculus humanus
Acyrthosiphon pisum
Tribolium castaneum
Nasonia vitripennis
Apis mellifera
Atta cephalotes
Bombyx mori
Danaus plexippus
Heliconius melpomene
Culex quinquefasciatus
Aedes aegypti
Anopheles gambiae
Anopheles darlingi
Drosophila virilis
Drosophila grimshawi
Drosophila willistoni
Drosophila pseudoobscura
Drosophila ananassae
Drosophila melanogaster
Drosophila yakuba
Strongylocentrotus purpuratus
Branchiostoma floridae
Ciona intestinalis
Ciona savignyi
Petromyzon marinus
Danio rerio
Gadus morhua
Oreochromis niloticus
Takifugu rubripes
Tetraodon nigroviridis
Gasterosteus aculeatus
Xiphophorus maculatus
Oryzias latipes
Latimeria chalumnae
Xenopus (Silurana) tropicalis
Pelodiscus sinensis
Anolis carolinensis
Taeniopygia guttata
Gallus gallus
Meleagris gallopavo
Ornithorhynchus anatinus
Monodelphis domestica
Sarcophilus harrisii
Macropus eugenii
Dasypus novemcinctus
Loxodonta africana
Echinops telfairi
Procavia capensis
Myotis lucifugus
Pteropus vampyrus
Sus scrofa
Tursiops truncatus
Bos taurus
Equus caballus
Felis catus
Canis lupus familiaris
Mustela putorius furo
Ailuropoda melanoleuca
Oryctolagus cuniculus
Cavia porcellus
Ictidomys tridecemlineatus
Dipodomys ordii
Rattus norvegicus
Mus musculus
Tupaia belangeri
Otolemur garnettii
Microcebus murinus
Tarsius syrichta
Callithrix jacchus
Macaca mulatta
Nomascus leucogenys
Pongo abelii
Gorilla gorilla
Pan troglodytes
Homo sapiens
Perkinsus marinus ATCC 50983
Tetrahymena thermophila
Paramecium tetraurelia strain d4 2
Neospora caninum Liverpool
Toxoplasma gondii
Cryptosporidium muris RN66
Cryptosporidium parvum Iowa II
Cryptosporidium hominis TU502
Theileria annulata strain Ankara
Theileria parva strain Muguga
Plasmodium falciparum
Plasmodium knowlesi
Plasmodium vivax
Plasmodium chabaudi
Plasmodium yoelii yoelii 17XNL
Plasmodium berghei
Phaeodactylum tricornutum
Thalassiosira pseudonana
Pythium ultimum
Albugo laibachii
Hyaloperonospora arabidopsidis
Phytophthora ramorum
Phytophthora infestans
Phytophthora sojae
Trypanosoma brucei
Trypanosoma cruzi strain CL Brener
Leishmania braziliensis MHOM BR 75 M2904
Leishmania mexicana MHOM GT 2001 U1103
Leishmania major
Leishmania donovani
Leishmania infantum JPCM5
Entamoeba histolytica
Entamoeba dispar SAW760
Dictyostelium discoideum
Dictyostelium purpureum
Trichomonas vaginalis G3
Trichomonas vaginalis
Naegleria gruberi
Giardia lamblia ATCC 50803
OG presence
OG absence
Inferred root
Dscore = 0.917
Pvalue = 2.86e−07
AdjPvalue = 3.40e−05

COG0225

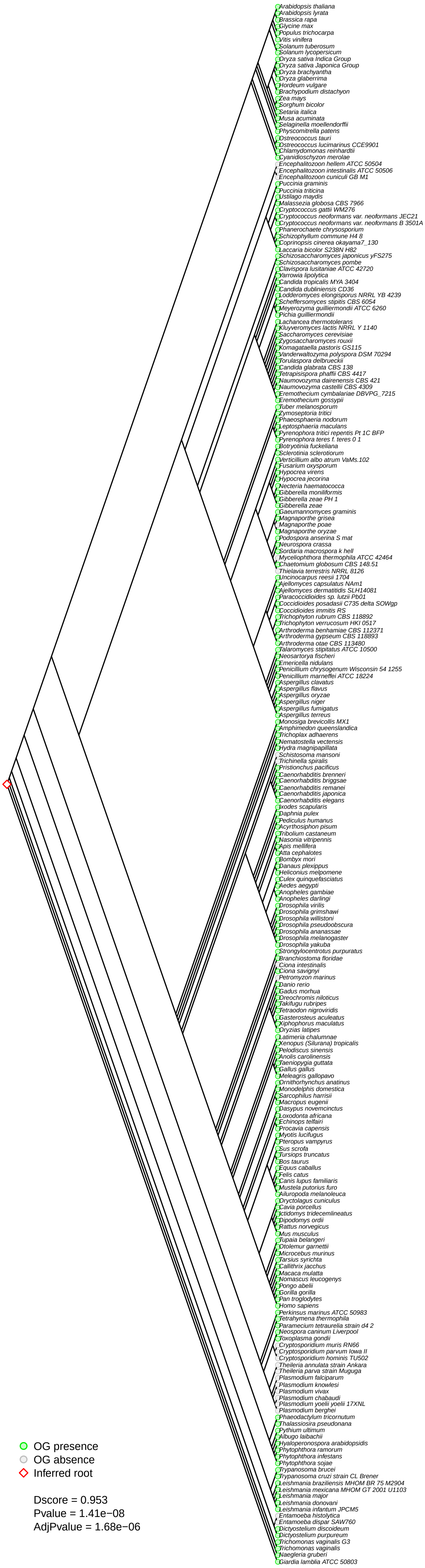
Arabidopsis thaliana
Arabidopsis lyrata
Brassica rapa
Glycine max
Populus trichocarpa
Vitis vinifera
Solanum tuberosum
Solanum lycopersicum
Oryza sativa Indica Group
Oryza sativa Japonica Group
Oryza brachyantha
Oryza glaberrima
Hordeum vulgare
Brachypodium distachyon
Zea mays
Sorghum bicolor
Setaria italica
Musa acuminata
Selaginella moellendorffii
Physcomitrella patens
Ostreococcus tauri
Ostreococcus lucimarinus CCE9901
Chlamydomonas reinhardtii
Cyanidioschyzon merolae
Encephalitozoon hellem ATCC 50504
Encephalitozoon intestinalis ATCC 50506
Encephalitozoon cuniculi GB M1
Puccinia graminis
Puccinia triticina
Ustilago maydis
Malassezia globosa CBS 7966
Cryptococcus gattii WM276
Cryptococcus neoformans var. neoformans JEC21
Cryptococcus neoformans var. neoformans B 3501A
Phanerochaete chrysosporium
Schizophyllum commune H4 8
Coprinopsis cinerea okayama7_130
Laccaria bicolor S238N H82
Schizosaccharomyces japonicus yFS275
Schizosaccharomyces pombe
Clavispora lusitaniae ATCC 42720
Yarrowia lipolytica
Candida tropicalis MYA 3404
Candida dubliniensis CD36
Lodderomyces elongisporus NRRL YB 4239
Scheffersomyces stipitis CBS 6054
Meyerozyma guilliermondii ATCC 6260
Pichia guilliermondii
Lachancea thermotolerans
Kluyveromyces lactis NRRL Y 1140
Saccharomyces cerevisiae
Zygosaccharomyces rouxii
Komagataella pastoris GS115
Vanderwaltozyma polyspora DSM 70294
Torulaspora delbrueckii
Candida glabrata CBS 138
Tetrapisispora phaffii CBS 4417
Naumovozyma dairenensis CBS 421
Naumovozyma castellii CBS 4309
Eremothecium cymbalariae DBVPG_7215
Eremothecium gossypii
Tuber melanosporum
Zymoseptoria tritici
Phaeosphaeria nodorum
Leptosphaeria maculans
Pyrenophora tritici repentis Pt 1C BFP
Pyrenophora teres f. teres 0 1
Botryotinia fuckeliana
Sclerotinia sclerotiorum
Verticillium albo atrum VaMs.102
Fusarium oxysporum
Hypocrea virens
Hypocrea jecorina
Nectria haematococca
Gibberella moniliformis
Gibberella zeae PH 1
Gibberella zeae
Gaeumannomyces graminis
Magnaporthe grisea
Magnaporthe poae
Magnaporthe oryzae
Podospora anserina S mat
Neurospora crassa
Sordaria macrospora k hell
Myceliophthora thermophila ATCC 42464
Chaetomium globosum CBS 148.51
Thielavia terrestris NRRL 8126
Uncinocarpus reesii 1704
Ajellomyces capsulatus NAm1
Ajellomyces dermatitidis SLH14081
Paracoccidioides sp. lutzii Pb01
Coccidioides posadasii C735 delta SOWgp
Coccidioides immitis RS
Trichophyton rubrum CBS 118892
Trichophyton verrucosum HKI 0517
Arthroderma benhamiae CBS 112371
Arthroderma gypseum CBS 118893
Arthroderma otae CBS 113480
Talaromyces stipitatus ATCC 10500
Neosartorya fischeri
Emericella nidulans
Penicillium chrysogenum Wisconsin 54 1255
Penicillium marneffei ATCC 18224
Aspergillus clavatus
Aspergillus flavus
Aspergillus oryzae
Aspergillus niger
Aspergillus fumigatus
Aspergillus terreus
Monosiga brevicollis MX1
Amphimedon queenslandica
Trichoplax adhaerens
Nematostella vectensis
Hydra magnipapillata
Schistosoma mansoni
Trichinella spiralis
Pristionchus pacificus
Caenorhabditis brenneri
Caenorhabditis briggsae
Caenorhabditis remanei
Caenorhabditis japonica
Caenorhabditis elegans
Ixodes scapularis
Daphnia pulex
Pediculus humanus
Acyrthosiphon pisum
Tribolium castaneum
Nasonia vitripennis
Apis mellifera
Atta cephalotes
Bombyx mori
Danaus plexippus
Heliconius melpomene
Culex quinquefasciatus
Aedes aegypti
Anopheles gambiae
Anopheles darlingi
Drosophila virilis
Drosophila grimshawi
Drosophila willistoni
Drosophila pseudoobscura
Drosophila ananassae
Drosophila melanogaster
Drosophila yakuba
Strongylocentrotus purpuratus
Branchiostoma floridae
Ciona intestinalis
Ciona savignyi
Petromyzon marinus
Danio rerio
Gadus morhua
Oreochromis niloticus
Takifugu rubripes
Tetraodon nigroviridis
Gasterosteus aculeatus
Xiphophorus maculatus
Oryzias latipes
Latimeria chalumnae
Xenopus (Silurana) tropicalis
Pelodiscus sinensis
Anolis carolinensis
Taeniopygia guttata
Gallus gallus
Meleagris gallopavo
Ornithorhynchus anatinus
Monodelphis domestica
Sarcophilus harrisii
Macropus eugenii
Dasypus novemcinctus
Loxodonta africana
Echinops telfairi
Procavia capensis
Myotis lucifugus
Pteropus vampyrus
Sus scrofa
Tursiops truncatus
Bos taurus
Equus caballus
Felis catus
Canis lupus familiaris
Mustela putorius furo
Ailuropoda melanoleuca
Oryctolagus cuniculus
Cavia porcellus
Ictidomys tridecemlineatus
Dipodomys ordii
Rattus norvegicus
Mus musculus
Tupaia belangeri
Otolemur garnettii
Microcebus murinus
Tarsius syrichta
Callithrix jacchus
Macaca mulatta
Nomascus leucogenys
Pongo abelii
Gorilla gorilla
Pan troglodytes
Homo sapiens
Perkinsus marinus ATCC 50983
Tetrahymena thermophila
Paramecium tetraurelia strain d4 2
Neospora caninum Liverpool
Toxoplasma gondii
Cryptosporidium muris RN66
Cryptosporidium parvum Iowa II
Cryptosporidium hominis TU502
Theileria annulata strain Ankara
Theileria parva strain Muguga
Plasmodium falciparum
Plasmodium knowlesi
Plasmodium vivax
Plasmodium chabaudi
Plasmodium yoelii yoelii 17XNL
Plasmodium berghei
Phaeodactylum tricornutum
Thalassiosira pseudonana
Pythium ultimum
Albugo laibachii
Hyaloperonospora arabidopsidis
Phytophthora ramorum
Phytophthora infestans
Phytophthora sojae
Trypanosoma brucei
Trypanosoma cruzi strain CL Brener
Leishmania braziliensis MHOM BR 75 M2904
Leishmania mexicana MHOM GT 2001 U1103
Leishmania major
Leishmania donovani
Leishmania infantum JPCM5
Entamoeba histolytica
Entamoeba dispar SAW760
Dictyostelium discoideum
Dictyostelium purpureum
Trichomonas vaginalis G3
Trichomonas vaginalis
Naegleria gruberi
Giardia lamblia ATCC 50803
OG presence
OG absence
Inferred root
Dscore = 0.953
Pvalue = 1.41e−08
AdjPvalue = 1.68e−06

COG0234

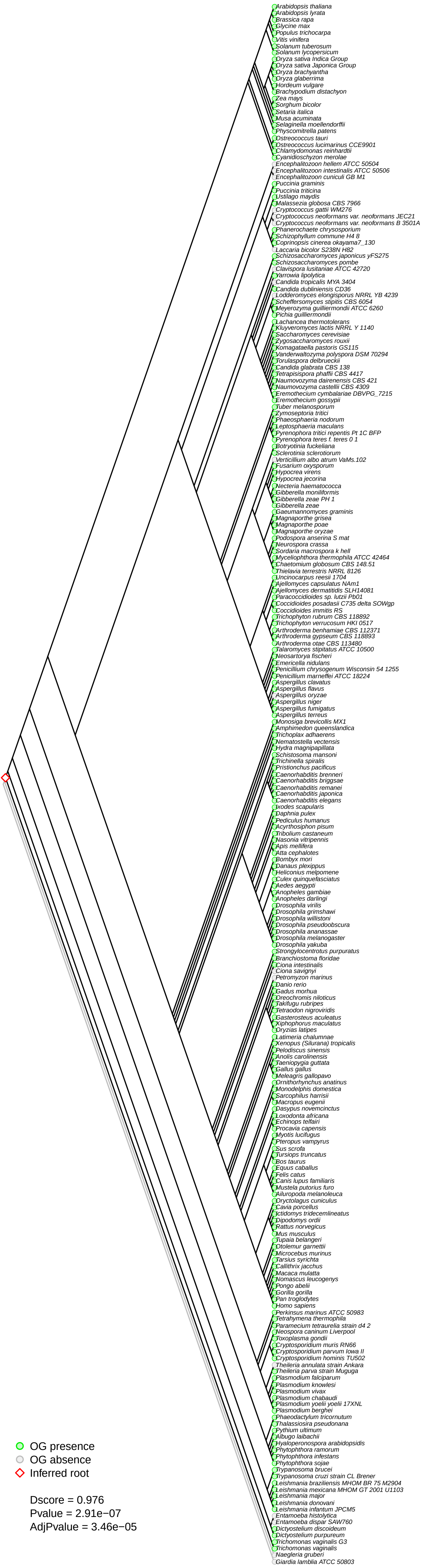

Arabidopsis thaliana
Arabidopsis lyrata
Brassica rapa
Glycine max
Populus trichocarpa
Vitis vinifera
Solanum tuberosum
Solanum lycopersicum
Oryza sativa Indica Group
Oryza sativa Japonica Group
Oryza brachyantha
Oryza glaberrima
Hordeum vulgare
Brachypodium distachyon
Zea mays
Sorghum bicolor
Setaria italica
Musa acuminata
Selaginella moellendorffii
Physcomitrella patens
Ostreococcus tauri
Ostreococcus lucimarinus CCE9901
Chlamydomonas reinhardtii
Cyanidioschyzon merolae
Encephalitozoon hellem ATCC 50504
Encephalitozoon intestinalis ATCC 50506
Encephalitozoon cuniculi GB M1
Puccinia graminis
Puccinia triticina
Ustilago maydis
Malassezia globosa CBS 7966
Cryptococcus gattii WM276
Cryptococcus neoformans var. neoformans JEC21
Cryptococcus neoformans var. neoformans B 3501A
Phanerochaete chrysosporium
Schizophyllum commune H4 8
Coprinopsis cinerea okayama7_130
Laccaria bicolor S238N H82
Schizosaccharomyces japonicus yFS275
Schizosaccharomyces pombe
Clavispora lusitaniae ATCC 42720
Yarrowia lipolytica
Candida tropicalis MYA 3404
Candida dubliniensis CD36
Lodderomyces elongisporus NRRL YB 4239
Scheffersomyces stipitis CBS 6054
Meyerozyma guilliermondii ATCC 6260
Pichia guilliermondii
Lachancea thermotolerans
Kluyveromyces lactis NRRL Y 1140
Saccharomyces cerevisiae
Zygosaccharomyces rouxii
Komagataella pastoris GS115
Vanderwaltozyma polyspora DSM 70294
Torulaspora delbrueckii
Candida glabrata CBS 138
Tetrapisispora phaffii CBS 4417
Naumovozyma dairenensis CBS 421
Naumovozyma castellii CBS 4309
Eremothecium cymbalariae DBVPG_7215
Eremothecium gossypii
Tuber melanosporum
Zymoseptoria tritici
Phaeosphaeria nodorum
Leptosphaeria maculans
Pyrenophora tritici repentis Pt 1C BFP
Pyrenophora teres f. teres 0 1
Botryotinia fuckeliana
Sclerotinia sclerotiorum
Verticillium albo atrum VaMs.102
Fusarium oxysporum
Hypocrea virens
Hypocrea jecorina
Nectria haematococca
Gibberella moniliformis
Gibberella zeae PH 1
Gibberella zeae
Gaeumannomyces graminis
Magnaporthe grisea
Magnaporthe poae
Magnaporthe oryzae
Podospora anserina S mat
Neurospora crassa
Sordaria macrospora k hell
Myceliophthora thermophila ATCC 42464
Chaetomium globosum CBS 148.51
Thielavia terrestris NRRL 8126
Uncinocarpus reesii 1704
Ajellomyces capsulatus NAm1
Ajellomyces dermatitidis SLH14081
Paracoccidioides sp. lutzii Pb01
Coccidioides posadasii C735 delta SOWgp
Coccidioides immitis RS
Trichophyton rubrum CBS 118892
Trichophyton verrucosum HKI 0517
Arthroderma benhamiae CBS 112371
Arthroderma gypseum CBS 118893
Arthroderma otae CBS 113480
Talaromyces stipitatus ATCC 10500
Neosartorya fischeri
Emericella nidulans
Penicillium chrysogenum Wisconsin 54 1255
Penicillium marneffei ATCC 18224
Aspergillus clavatus
Aspergillus flavus
Aspergillus oryzae
Aspergillus niger
Aspergillus fumigatus
Aspergillus terreus
Monosiga brevicollis MX1
Amphimedon queenslandica
Trichoplax adhaerens
Nematostella vectensis
Hydra magnipapillata
Schistosoma mansoni
Trichinella spiralis
Pristionchus pacificus
Caenorhabditis brenneri
Caenorhabditis briggsae
Caenorhabditis remanei
Caenorhabditis japonica
Caenorhabditis elegans
Ixodes scapularis
Daphnia pulex
Pediculus humanus
Acyrthosiphon pisum
Tribolium castaneum
Nasonia vitripennis
Apis mellifera
Atta cephalotes
Bombyx mori
Danaus plexippus
Heliconius melpomene
Culex quinquefasciatus
Aedes aegypti
Anopheles gambiae
Anopheles darlingi
Drosophila virilis
Drosophila grimshawi
Drosophila willistoni
Drosophila pseudoobscura
Drosophila ananassae
Drosophila melanogaster
Drosophila yakuba
Strongylocentrotus purpuratus
Branchiostoma floridae
Ciona intestinalis
Ciona savignyi
Petromyzon marinus
Danio rerio
Gadus morhua
Oreochromis niloticus
Takifugu rubripes
Tetraodon nigroviridis
Gasterosteus aculeatus
Xiphophorus maculatus
Oryzias latipes
Latimeria chalumnae
Xenopus (Silurana) tropicalis
Pelodiscus sinensis
Anolis carolinensis
Taeniopygia guttata
Gallus gallus
Meleagris gallopavo
Ornithorhynchus anatinus
Monodelphis domestica
Sarcophilus harrisii
Macropus eugenii
Dasypus novemcinctus
Loxodonta africana
Echinops telfairi
Procavia capensis
Myotis lucifugus
Pteropus vampyrus
Sus scrofa
Tursiops truncatus
Bos taurus
Equus caballus
Felis catus
Canis lupus familiaris
Mustela putorius furo
Ailuropoda melanoleuca
Oryctolagus cuniculus
Cavia porcellus
Ictidomys tridecemlineatus
Dipodomys ordii
Rattus norvegicus
Mus musculus
Tupaia belangeri
Otolemur garnettii
Microcebus murinus
Tarsius syrichta
Callithrix jacchus
Macaca mulatta
Nomascus leucogenys
Pongo abelii
Gorilla gorilla
Pan troglodytes
Homo sapiens
Perkinsus marinus ATCC 50983
Tetrahymena thermophila
Paramecium tetraurelia strain d4 2
Neospora caninum Liverpool
Toxoplasma gondii
Cryptosporidium muris RN66
Cryptosporidium parvum Iowa II
Cryptosporidium hominis TU502
Theileria annulata strain Ankara
Theileria parva strain Muguga
Plasmodium falciparum
Plasmodium knowlesi
Plasmodium vivax
Plasmodium chabaudi
Plasmodium yoelii yoelii 17XNL
Plasmodium berghei
Phaeodactylum tricornutum
Thalassiosira pseudonana
Pythium ultimum
Albugo laibachii
Hyaloperonospora arabidopsidis
Phytophthora ramorum
Phytophthora infestans
Phytophthora sojae
Trypanosoma brucei
Trypanosoma cruzi strain CL Brener
Leishmania braziliensis MHOM BR 75 M2904
Leishmania mexicana MHOM GT 2001 U1103
Leishmania major
Leishmania donovani
Leishmania infantum JPCM5
Entamoeba histolytica
Entamoeba dispar SAW760
Dictyostelium discoideum
Dictyostelium purpureum
Trichomonas vaginalis G3
Trichomonas vaginalis
Naegleria gruberi
Giardia lamblia ATCC 50803
OG presence
OG absence
Inferred root
Dscore = 0.976
Pvalue = 2.91e−07
AdjPvalue = 3.46e−05

COG0278

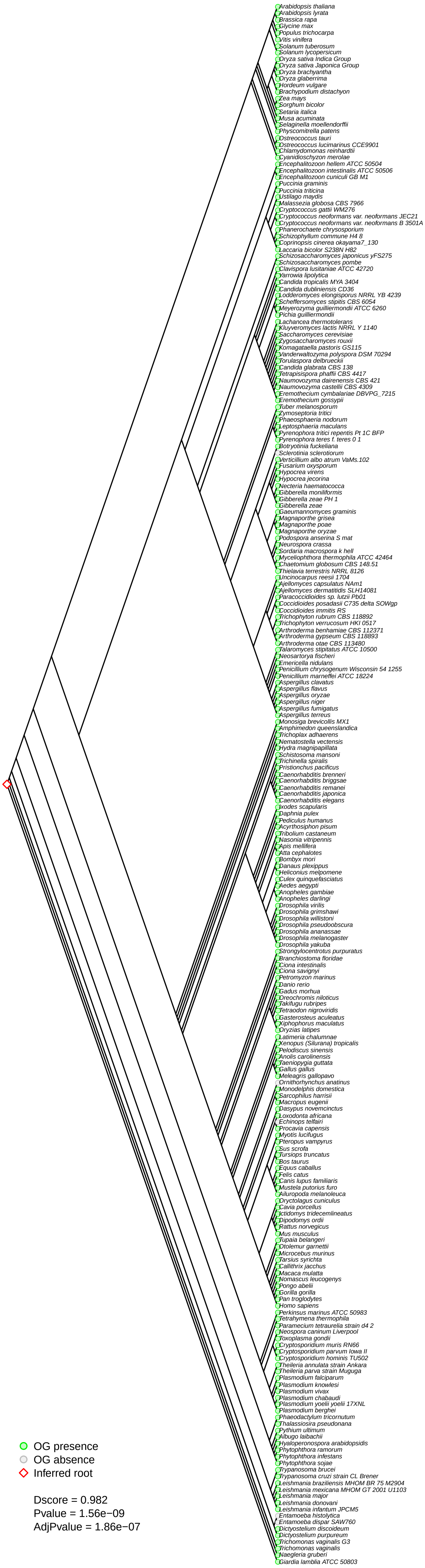
Arabidopsis thaliana
Arabidopsis lyrata
Brassica rapa
Glycine max
Populus trichocarpa
Vitis vinifera
Solanum tuberosum
Solanum lycopersicum
Oryza sativa Indica Group
Oryza sativa Japonica Group
Oryza brachyantha
Oryza glaberrima
Hordeum vulgare
Brachypodium distachyon
Zea mays
Sorghum bicolor
Setaria italica
Musa acuminata
Selaginella moellendorffii
Physcomitrella patens
Ostreococcus tauri
Ostreococcus lucimarinus CCE9901
Chlamydomonas reinhardtii
Cyanidioschyzon merolae
Encephalitozoon hellem ATCC 50504
Encephalitozoon intestinalis ATCC 50506
Encephalitozoon cuniculi GB M1
Puccinia graminis
Puccinia triticina
Ustilago maydis
Malassezia globosa CBS 7966
Cryptococcus gattii WM276
Cryptococcus neoformans var. neoformans JEC21
Cryptococcus neoformans var. neoformans B 3501A
Phanerochaete chrysosporium
Schizophyllum commune H4 8
Coprinopsis cinerea okayama7_130
Laccaria bicolor S238N H82
Schizosaccharomyces japonicus yFS275
Schizosaccharomyces pombe
Clavispora lusitaniae ATCC 42720
Yarrowia lipolytica
Candida tropicalis MYA 3404
Candida dubliniensis CD36
Lodderomyces elongisporus NRRL YB 4239
Scheffersomyces stipitis CBS 6054
Meyerozyma guilliermondii ATCC 6260
Pichia guilliermondii
Lachancea thermotolerans
Kluyveromyces lactis NRRL Y 1140
Saccharomyces cerevisiae
Zygosaccharomyces rouxii
Komagataella pastoris GS115
Vanderwaltozyma polyspora DSM 70294
Torulaspora delbrueckii
Candida glabrata CBS 138
Tetrapisispora phaffii CBS 4417
Naumovozyma dairenensis CBS 421
Naumovozyma castellii CBS 4309
Eremothecium cymbalariae DBVPG_7215
Eremothecium gossypii
Tuber melanosporum
Zymoseptoria tritici
Phaeosphaeria nodorum
Leptosphaeria maculans
Pyrenophora tritici repentis Pt 1C BFP
Pyrenophora teres f. teres 0 1
Botryotinia fuckeliana
Sclerotinia sclerotiorum
Verticillium albo atrum VaMs.102
Fusarium oxysporum
Hypocrea virens
Hypocrea jecorina
Nectria haematococca
Gibberella moniliformis
Gibberella zeae PH 1
Gibberella zeae
Gaeumannomyces graminis
Magnaporthe grisea
Magnaporthe poae
Magnaporthe oryzae
Podospora anserina S mat
Neurospora crassa
Sordaria macrospora k hell
Myceliophthora thermophila ATCC 42464
Chaetomium globosum CBS 148.51
Thielavia terrestris NRRL 8126
Uncinocarpus reesii 1704
Ajellomyces capsulatus NAm1
Ajellomyces dermatitidis SLH14081
Paracoccidioides sp. lutzii Pb01
Coccidioides posadasii C735 delta SOWgp
Coccidioides immitis RS
Trichophyton rubrum CBS 118892
Trichophyton verrucosum HKI 0517
Arthroderma benhamiae CBS 112371
Arthroderma gypseum CBS 118893
Arthroderma otae CBS 113480
Talaromyces stipitatus ATCC 10500
Neosartorya fischeri
Emericella nidulans
Penicillium chrysogenum Wisconsin 54 1255
Penicillium marneffei ATCC 18224
Aspergillus clavatus
Aspergillus flavus
Aspergillus oryzae
Aspergillus niger
Aspergillus fumigatus
Aspergillus terreus
Monosiga brevicollis MX1
Amphimedon queenslandica
Trichoplax adhaerens
Nematostella vectensis
Hydra magnipapillata
Schistosoma mansoni
Trichinella spiralis
Pristionchus pacificus
Caenorhabditis brenneri
Caenorhabditis briggsae
Caenorhabditis remanei
Caenorhabditis japonica
Caenorhabditis elegans
Ixodes scapularis
Daphnia pulex
Pediculus humanus
Acyrthosiphon pisum
Tribolium castaneum
Nasonia vitripennis
Apis mellifera
Atta cephalotes
Bombyx mori
Danaus plexippus
Heliconius melpomene
Culex quinquefasciatus
Aedes aegypti
Anopheles gambiae
Anopheles darlingi
Drosophila virilis
Drosophila grimshawi
Drosophila willistoni
Drosophila pseudoobscura
Drosophila ananassae
Drosophila melanogaster
Drosophila yakuba
Strongylocentrotus purpuratus
Branchiostoma floridae
Ciona intestinalis
Ciona savignyi
Petromyzon marinus
Danio rerio
Gadus morhua
Oreochromis niloticus
Takifugu rubripes
Tetraodon nigroviridis
Gasterosteus aculeatus
Xiphophorus maculatus
Oryzias latipes
Latimeria chalumnae
Xenopus (Silurana) tropicalis
Pelodiscus sinensis
Anolis carolinensis
Taeniopygia guttata
Gallus gallus
Meleagris gallopavo
Ornithorhynchus anatinus
Monodelphis domestica
Sarcophilus harrisii
Macropus eugenii
Dasypus novemcinctus
Loxodonta africana
Echinops telfairi
Procavia capensis
Myotis lucifugus
Pteropus vampyrus
Sus scrofa
Tursiops truncatus
Bos taurus
Equus caballus
Felis catus
Canis lupus familiaris
Mustela putorius furo
Ailuropoda melanoleuca
Oryctolagus cuniculus
Cavia porcellus
Ictidomys tridecemlineatus
Dipodomys ordii
Rattus norvegicus
Mus musculus
Tupaia belangeri
Otolemur garnettii
Microcebus murinus
Tarsius syrichta
Callithrix jacchus
Macaca mulatta
Nomascus leucogenys
Pongo abelii
Gorilla gorilla
Pan troglodytes
Homo sapiens
Perkinsus marinus ATCC 50983
Tetrahymena thermophila
Paramecium tetraurelia strain d4 2
Neospora caninum Liverpool
Toxoplasma gondii
Cryptosporidium muris RN66
Cryptosporidium parvum Iowa II
Cryptosporidium hominis TU502
Theileria annulata strain Ankara
Theileria parva strain Muguga
Plasmodium falciparum
Plasmodium knowlesi
Plasmodium vivax
Plasmodium chabaudi
Plasmodium yoelii yoelii 17XNL
Plasmodium berghei
Phaeodactylum tricornutum
Thalassiosira pseudonana
Pythium ultimum
Albugo laibachii
Hyaloperonospora arabidopsidis
Phytophthora ramorum
Phytophthora infestans
Phytophthora sojae
Trypanosoma brucei
Trypanosoma cruzi strain CL Brener
Leishmania braziliensis MHOM BR 75 M2904
Leishmania mexicana MHOM GT 2001 U1103
Leishmania major
Leishmania donovani
Leishmania infantum JPCM5
Entamoeba histolytica
Entamoeba dispar SAW760
Dictyostelium discoideum
Dictyostelium purpureum
Trichomonas vaginalis G3
Trichomonas vaginalis
Naegleria gruberi
Giardia lamblia ATCC 50803
OG presence
OG absence
Inferred root
Dscore = 0.982
Pvalue = 1.56e−09
AdjPvalue = 1.86e−07

COG0369

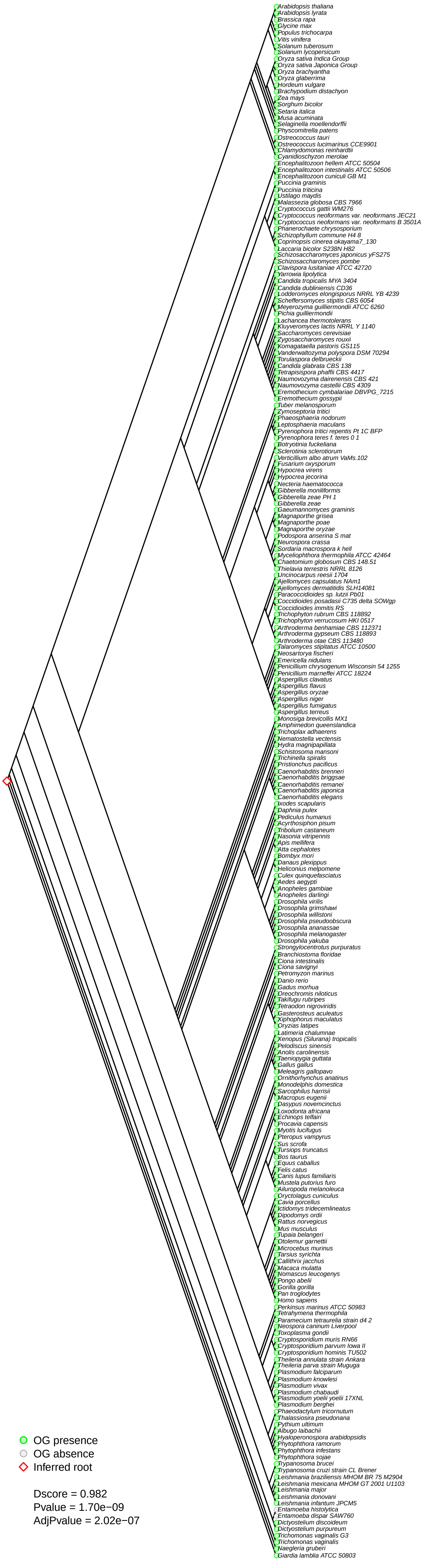

Arabidopsis thaliana
Arabidopsis lyrata
Brassica rapa
Glycine max
Populus trichocarpa
Vitis vinifera
Solanum tuberosum
Solanum lycopersicum
Oryza sativa Indica Group
Oryza sativa Japonica Group
Oryza brachyantha
Oryza glaberrima
Hordeum vulgare
Brachypodium distachyon
Zea mays
Sorghum bicolor
Setaria italica
Musa acuminata
Selaginella moellendorffii
Physcomitrella patens
Ostreococcus tauri
Ostreococcus lucimarinus CCE9901
Chlamydomonas reinhardtii
Cyanidioschyzon merolae
Encephalitozoon hellem ATCC 50504
Encephalitozoon intestinalis ATCC 50506
Encephalitozoon cuniculi GB M1
Puccinia graminis
Puccinia triticina
Ustilago maydis
Malassezia globosa CBS 7966
Cryptococcus gattii WM276
Cryptococcus neoformans var. neoformans JEC21
Cryptococcus neoformans var. neoformans B 3501A
Phanerochaete chrysosporium
Schizophyllum commune H4 8
Coprinopsis cinerea okayama7_130
Laccaria bicolor S238N H82
Schizosaccharomyces japonicus yFS275
Schizosaccharomyces pombe
Clavispora lusitaniae ATCC 42720
Yarrowia lipolytica
Candida tropicalis MYA 3404
Candida dubliniensis CD36
Lodderomyces elongisporus NRRL YB 4239
Scheffersomyces stipitis CBS 6054
Meyerozyma guilliermondii ATCC 6260
Pichia guilliermondii
Lachancea thermotolerans
Kluyveromyces lactis NRRL Y 1140
Saccharomyces cerevisiae
Zygosaccharomyces rouxii
Komagataella pastoris GS115
Vanderwaltozyma polyspora DSM 70294
Torulaspora delbrueckii
Candida glabrata CBS 138
Tetrapisispora phaffii CBS 4417
Naumovozyma dairenensis CBS 421
Naumovozyma castellii CBS 4309
Eremothecium cymbalariae DBVPG_7215
Eremothecium gossypii
Tuber melanosporum
Zymoseptoria tritici
Phaeosphaeria nodorum
Leptosphaeria maculans
Pyrenophora tritici repentis Pt 1C BFP
Pyrenophora teres f. teres 0 1
Botryotinia fuckeliana
Sclerotinia sclerotiorum
Verticillium albo atrum VaMs.102
Fusarium oxysporum
Hypocrea virens
Hypocrea jecorina
Nectria haematococca
Gibberella moniliformis
Gibberella zeae PH 1
Gibberella zeae
Gaeumannomyces graminis
Magnaporthe grisea
Magnaporthe poae
Magnaporthe oryzae
Podospora anserina S mat
Neurospora crassa
Sordaria macrospora k hell
Myceliophthora thermophila ATCC 42464
Chaetomium globosum CBS 148.51
Thielavia terrestris NRRL 8126
Uncinocarpus reesii 1704
Ajellomyces capsulatus NAm1
Ajellomyces dermatitidis SLH14081
Paracoccidioides sp. lutzii Pb01
Coccidioides posadasii C735 delta SOWgp
Coccidioides immitis RS
Trichophyton rubrum CBS 118892
Trichophyton verrucosum HKI 0517
Arthroderma benhamiae CBS 112371
Arthroderma gypseum CBS 118893
Arthroderma otae CBS 113480
Talaromyces stipitatus ATCC 10500
Neosartorya fischeri
Emericella nidulans
Penicillium chrysogenum Wisconsin 54 1255
Penicillium marneffei ATCC 18224
Aspergillus clavatus
Aspergillus flavus
Aspergillus oryzae
Aspergillus niger
Aspergillus fumigatus
Aspergillus terreus
Monosiga brevicollis MX1
Amphimedon queenslandica
Trichoplax adhaerens
Nematostella vectensis
Hydra magnipapillata
Schistosoma mansoni
Trichinella spiralis
Pristionchus pacificus
Caenorhabditis brenneri
Caenorhabditis briggsae
Caenorhabditis remanei
Caenorhabditis japonica
Caenorhabditis elegans
Ixodes scapularis
Daphnia pulex
Pediculus humanus
Acyrthosiphon pisum
Tribolium castaneum
Nasonia vitripennis
Apis mellifera
Atta cephalotes
Bombyx mori
Danaus plexippus
Heliconius melpomene
Culex quinquefasciatus
Aedes aegypti
Anopheles gambiae
Anopheles darlingi
Drosophila virilis
Drosophila grimshawi
Drosophila willistoni
Drosophila pseudoobscura
Drosophila ananassae
Drosophila melanogaster
Drosophila yakuba
Strongylocentrotus purpuratus
Branchiostoma floridae
Ciona intestinalis
Ciona savignyi
Petromyzon marinus
Danio rerio
Gadus morhua
Oreochromis niloticus
Takifugu rubripes
Tetraodon nigroviridis
Gasterosteus aculeatus
Xiphophorus maculatus
Oryzias latipes
Latimeria chalumnae
Xenopus (Silurana) tropicalis
Pelodiscus sinensis
Anolis carolinensis
Taeniopygia guttata
Gallus gallus
Meleagris gallopavo
Ornithorhynchus anatinus
Monodelphis domestica
Sarcophilus harrisii
Macropus eugenii
Dasypus novemcinctus
Loxodonta africana
Echinops telfairi
Procavia capensis
Myotis lucifugus
Pteropus vampyrus
Sus scrofa
Tursiops truncatus
Bos taurus
Equus caballus
Felis catus
Canis lupus familiaris
Mustela putorius furo
Ailuropoda melanoleuca
Oryctolagus cuniculus
Cavia porcellus
Ictidomys tridecemlineatus
Dipodomys ordii
Rattus norvegicus
Mus musculus
Tupaia belangeri
Otolemur garnettii
Microcebus murinus
Tarsius syrichta
Callithrix jacchus
Macaca mulatta
Nomascus leucogenys
Pongo abelii
Gorilla gorilla
Pan troglodytes
Homo sapiens
Perkinsus marinus ATCC 50983
Tetrahymena thermophila
Paramecium tetraurelia strain d4 2
Neospora caninum Liverpool
Toxoplasma gondii
Cryptosporidium muris RN66
Cryptosporidium parvum Iowa II
Cryptosporidium hominis TU502
Theileria annulata strain Ankara
Theileria parva strain Muguga
Plasmodium falciparum
Plasmodium knowlesi
Plasmodium vivax
Plasmodium chabaudi
Plasmodium yoelii yoelii 17XNL
Plasmodium berghei
Phaeodactylum tricornutum
Thalassiosira pseudonana
Pythium ultimum
Albugo laibachii
Hyaloperonospora arabidopsidis
Phytophthora ramorum
Phytophthora infestans
Phytophthora sojae
Trypanosoma brucei
Trypanosoma cruzi strain CL Brener
Leishmania braziliensis MHOM BR 75 M2904
Leishmania mexicana MHOM GT 2001 U1103
Leishmania major
Leishmania donovani
Leishmania infantum JPCM5
Entamoeba histolytica
Entamoeba dispar SAW760
Dictyostelium discoideum
Dictyostelium purpureum
Trichomonas vaginalis G3
Trichomonas vaginalis
Naegleria gruberi
Giardia lamblia ATCC 50803
OG presence
OG absence
Inferred root
Dscore = 0.982
Pvalue = 1.70e−09
AdjPvalue = 2.02e−07

COG0376

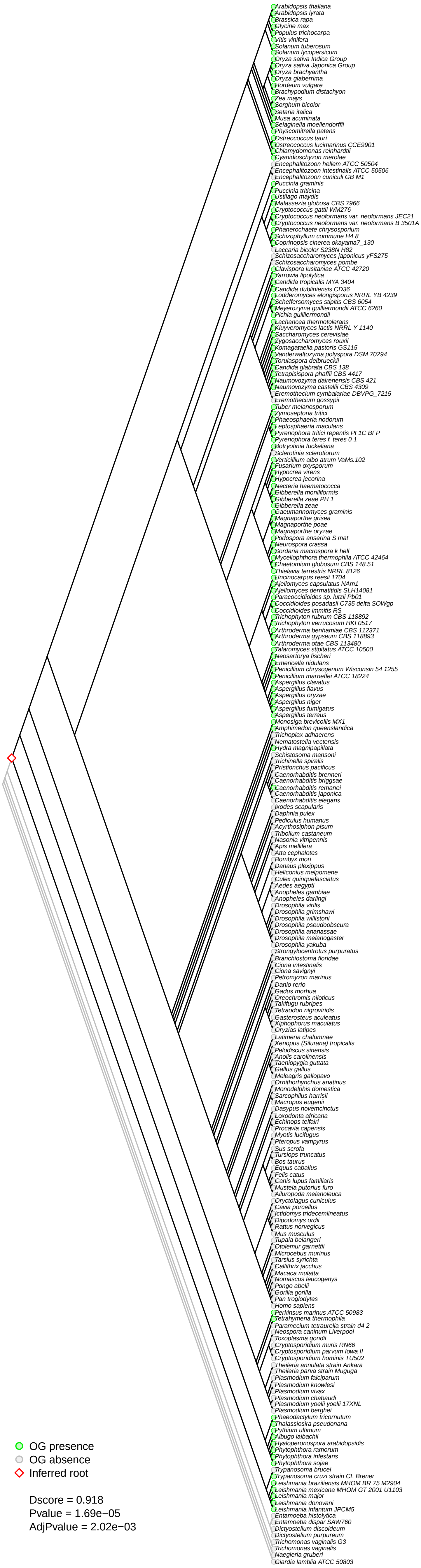
Arabidopsis thaliana
Arabidopsis lyrata
Brassica rapa
Glycine max
Populus trichocarpa
Vitis vinifera
Solanum tuberosum
Solanum lycopersicum
Oryza sativa Indica Group
Oryza sativa Japonica Group
Oryza brachyantha
Oryza glaberrima
Hordeum vulgare
Brachypodium distachyon
Zea mays
Sorghum bicolor
Setaria italica
Musa acuminata
Selaginella moellendorffii
Physcomitrella patens
Ostreococcus tauri
Ostreococcus lucimarinus CCE9901
Chlamydomonas reinhardtii
Cyanidioschyzon merolae
Encephalitozoon hellem ATCC 50504
Encephalitozoon intestinalis ATCC 50506
Encephalitozoon cuniculi GB M1
Puccinia graminis
Puccinia triticina
Ustilago maydis
Malassezia globosa CBS 7966
Cryptococcus gattii WM276
Cryptococcus neoformans var. neoformans JEC21
Cryptococcus neoformans var. neoformans B 3501A
Phanerochaete chrysosporium
Schizophyllum commune H4 8
Coprinopsis cinerea okayama7_130
Laccaria bicolor S238N H82
Schizosaccharomyces japonicus yFS275
Schizosaccharomyces pombe
Clavispora lusitaniae ATCC 42720
Yarrowia lipolytica
Candida tropicalis MYA 3404
Candida dubliniensis CD36
Lodderomyces elongisporus NRRL YB 4239
Scheffersomyces stipitis CBS 6054
Meyerozyma guilliermondii ATCC 6260
Pichia guilliermondii
Lachancea thermotolerans
Kluyveromyces lactis NRRL Y 1140
Saccharomyces cerevisiae
Zygosaccharomyces rouxii
Komagataella pastoris GS115
Vanderwaltozyma polyspora DSM 70294
Torulaspora delbrueckii
Candida glabrata CBS 138
Tetrapisispora phaffii CBS 4417
Naumovozyma dairenensis CBS 421
Naumovozyma castellii CBS 4309
Eremothecium cymbalariae DBVPG_7215
Eremothecium gossypii
Tuber melanosporum
Zymoseptoria tritici
Phaeosphaeria nodorum
Leptosphaeria maculans
Pyrenophora tritici repentis Pt 1C BFP
Pyrenophora teres f. teres 0 1
Botryotinia fuckeliana
Sclerotinia sclerotiorum
Verticillium albo atrum VaMs.102
Fusarium oxysporum
Hypocrea virens
Hypocrea jecorina
Nectria haematococca
Gibberella moniliformis
Gibberella zeae PH 1
Gibberella zeae
Gaeumannomyces graminis
Magnaporthe grisea
Magnaporthe poae
Magnaporthe oryzae
Podospora anserina S mat
Neurospora crassa
Sordaria macrospora k hell
Myceliophthora thermophila ATCC 42464
Chaetomium globosum CBS 148.51
Thielavia terrestris NRRL 8126
Uncinocarpus reesii 1704
Ajellomyces capsulatus NAm1
Ajellomyces dermatitidis SLH14081
Paracoccidioides sp. lutzii Pb01
Coccidioides posadasii C735 delta SOWgp
Coccidioides immitis RS
Trichophyton rubrum CBS 118892
Trichophyton verrucosum HKI 0517
Arthroderma benhamiae CBS 112371
Arthroderma gypseum CBS 118893
Arthroderma otae CBS 113480
Talaromyces stipitatus ATCC 10500
Neosartorya fischeri
Emericella nidulans
Penicillium chrysogenum Wisconsin 54 1255
Penicillium marneffei ATCC 18224
Aspergillus clavatus
Aspergillus flavus
Aspergillus oryzae
Aspergillus niger
Aspergillus fumigatus
Aspergillus terreus
Monosiga brevicollis MX1
Amphimedon queenslandica
Trichoplax adhaerens
Nematostella vectensis
Hydra magnipapillata
Schistosoma mansoni
Trichinella spiralis
Pristionchus pacificus
Caenorhabditis brenneri
Caenorhabditis briggsae
Caenorhabditis remanei
Caenorhabditis japonica
Caenorhabditis elegans
Ixodes scapularis
Daphnia pulex
Pediculus humanus
Acyrthosiphon pisum
Tribolium castaneum
Nasonia vitripennis
Apis mellifera
Atta cephalotes
Bombyx mori
Danaus plexippus
Heliconius melpomene
Culex quinquefasciatus
Aedes aegypti
Anopheles gambiae
Anopheles darlingi
Drosophila virilis
Drosophila grimshawi
Drosophila willistoni
Drosophila pseudoobscura
Drosophila ananassae
Drosophila melanogaster
Drosophila yakuba
Strongylocentrotus purpuratus
Branchiostoma floridae
Ciona intestinalis
Ciona savignyi
Petromyzon marinus
Danio rerio
Gadus morhua
Oreochromis niloticus
Takifugu rubripes
Tetraodon nigroviridis
Gasterosteus aculeatus
Xiphophorus maculatus
Oryzias latipes
Latimeria chalumnae
Xenopus (Silurana) tropicalis
Pelodiscus sinensis
Anolis carolinensis
Taeniopygia guttata
Gallus gallus
Meleagris gallopavo
Ornithorhynchus anatinus
Monodelphis domestica
Sarcophilus harrisii
Macropus eugenii
Dasypus novemcinctus
Loxodonta africana
Echinops telfairi
Procavia capensis
Myotis lucifugus
Pteropus vampyrus
Sus scrofa
Tursiops truncatus
Bos taurus
Equus caballus
Felis catus
Canis lupus familiaris
Mustela putorius furo
Ailuropoda melanoleuca
Oryctolagus cuniculus
Cavia porcellus
Ictidomys tridecemlineatus
Dipodomys ordii
Rattus norvegicus
Mus musculus
Tupaia belangeri
Otolemur garnettii
Microcebus murinus
Tarsius syrichta
Callithrix jacchus
Macaca mulatta
Nomascus leucogenys
Pongo abelii
Gorilla gorilla
Pan troglodytes
Homo sapiens
Perkinsus marinus ATCC 50983
Tetrahymena thermophila
Paramecium tetraurelia strain d4 2
Neospora caninum Liverpool
Toxoplasma gondii
Cryptosporidium muris RN66
Cryptosporidium parvum Iowa II
Cryptosporidium hominis TU502
Theileria annulata strain Ankara
Theileria parva strain Muguga
Plasmodium falciparum
Plasmodium knowlesi
Plasmodium vivax
Plasmodium chabaudi
Plasmodium yoelii yoelii 17XNL
Plasmodium berghei
Phaeodactylum tricornutum
Thalassiosira pseudonana
Pythium ultimum
Albugo laibachii
Hyaloperonospora arabidopsidis
Phytophthora ramorum
Phytophthora infestans
Phytophthora sojae
Trypanosoma brucei
Trypanosoma cruzi strain CL Brener
Leishmania braziliensis MHOM BR 75 M2904
Leishmania mexicana MHOM GT 2001 U1103
Leishmania major
Leishmania donovani
Leishmania infantum JPCM5
Entamoeba histolytica
Entamoeba dispar SAW760
Dictyostelium discoideum
Dictyostelium purpureum
Trichomonas vaginalis G3
Trichomonas vaginalis
Naegleria gruberi
Giardia lamblia ATCC 50803
OG presence
OG absence
Inferred root
Dscore = 0.918
Pvalue = 1.69e−05
AdjPvalue = 2.02e−03

COG0386

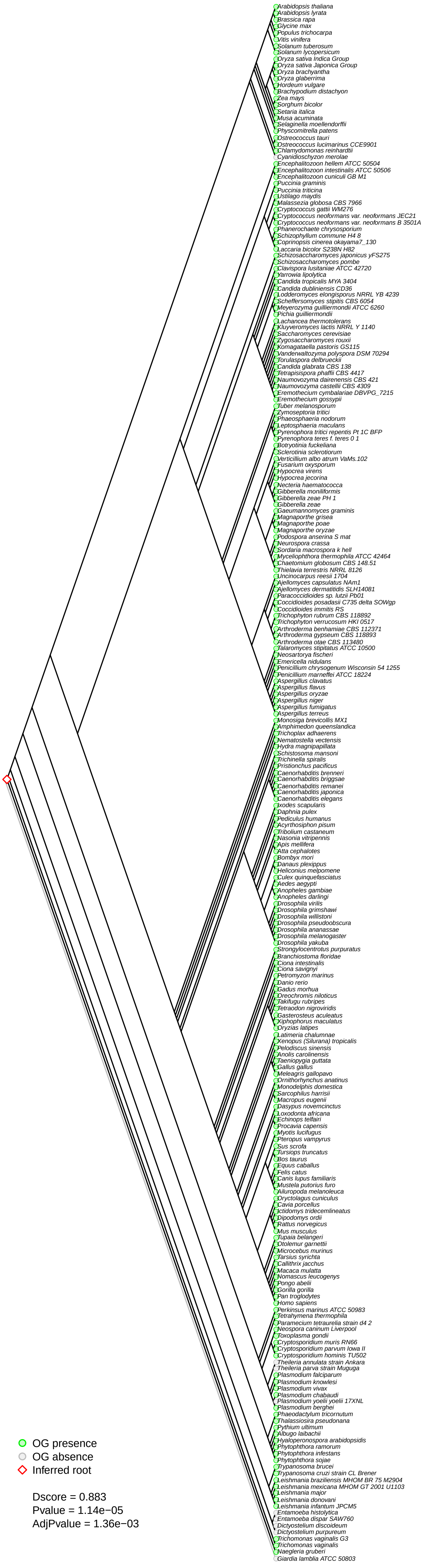

Arabidopsis thaliana
Arabidopsis lyrata
Brassica rapa
Glycine max
Populus trichocarpa
Vitis vinifera
Solanum tuberosum
Solanum lycopersicum
Oryza sativa Indica Group
Oryza sativa Japonica Group
Oryza brachyantha
Oryza glaberrima
Hordeum vulgare
Brachypodium distachyon
Zea mays
Sorghum bicolor
Setaria italica
Musa acuminata
Selaginella moellendorffii
Physcomitrella patens
Ostreococcus tauri
Ostreococcus lucimarinus CCE9901
Chlamydomonas reinhardtii
Cyanidioschyzon merolae
Encephalitozoon hellem ATCC 50504
Encephalitozoon intestinalis ATCC 50506
Encephalitozoon cuniculi GB M1
Puccinia graminis
Puccinia triticina
Ustilago maydis
Malassezia globosa CBS 7966
Cryptococcus gattii WM276
Cryptococcus neoformans var. neoformans JEC21
Cryptococcus neoformans var. neoformans B 3501A
Phanerochaete chrysosporium
Schizophyllum commune H4 8
Coprinopsis cinerea okayama7_130
Laccaria bicolor S238N H82
Schizosaccharomyces japonicus yFS275
Schizosaccharomyces pombe
Clavispora lusitaniae ATCC 42720
Yarrowia lipolytica
Candida tropicalis MYA 3404
Candida dubliniensis CD36
Lodderomyces elongisporus NRRL YB 4239
Scheffersomyces stipitis CBS 6054
Meyerozyma guilliermondii ATCC 6260
Pichia guilliermondii
Lachancea thermotolerans
Kluyveromyces lactis NRRL Y 1140
Saccharomyces cerevisiae
Zygosaccharomyces rouxii
Komagataella pastoris GS115
Vanderwaltozyma polyspora DSM 70294
Torulaspora delbrueckii
Candida glabrata CBS 138
Tetrapisispora phaffii CBS 4417
Naumovozyma dairenensis CBS 421
Naumovozyma castellii CBS 4309
Eremothecium cymbalariae DBVPG_7215
Eremothecium gossypii
Tuber melanosporum
Zymoseptoria tritici
Phaeosphaeria nodorum
Leptosphaeria maculans
Pyrenophora tritici repentis Pt 1C BFP
Pyrenophora teres f. teres 0 1
Botryotinia fuckeliana
Sclerotinia sclerotiorum
Verticillium albo atrum VaMs.102
Fusarium oxysporum
Hypocrea virens
Hypocrea jecorina
Nectria haematococca
Gibberella moniliformis
Gibberella zeae PH 1
Gibberella zeae
Gaeumannomyces graminis
Magnaporthe grisea
Magnaporthe poae
Magnaporthe oryzae
Podospora anserina S mat
Neurospora crassa
Sordaria macrospora k hell
Myceliophthora thermophila ATCC 42464
Chaetomium globosum CBS 148.51
Thielavia terrestris NRRL 8126
Uncinocarpus reesii 1704
Ajellomyces capsulatus NAm1
Ajellomyces dermatitidis SLH14081
Paracoccidioides sp. lutzii Pb01
Coccidioides posadasii C735 delta SOWgp
Coccidioides immitis RS
Trichophyton rubrum CBS 118892
Trichophyton verrucosum HKI 0517
Arthroderma benhamiae CBS 112371
Arthroderma gypseum CBS 118893
Arthroderma otae CBS 113480
Talaromyces stipitatus ATCC 10500
Neosartorya fischeri
Emericella nidulans
Penicillium chrysogenum Wisconsin 54 1255
Penicillium marneffei ATCC 18224
Aspergillus clavatus
Aspergillus flavus
Aspergillus oryzae
Aspergillus niger
Aspergillus fumigatus
Aspergillus terreus
Monosiga brevicollis MX1
Amphimedon queenslandica
Trichoplax adhaerens
Nematostella vectensis
Hydra magnipapillata
Schistosoma mansoni
Trichinella spiralis
Pristionchus pacificus
Caenorhabditis brenneri
Caenorhabditis briggsae
Caenorhabditis remanei
Caenorhabditis japonica
Caenorhabditis elegans
Ixodes scapularis
Daphnia pulex
Pediculus humanus
Acyrthosiphon pisum
Tribolium castaneum
Nasonia vitripennis
Apis mellifera
Atta cephalotes
Bombyx mori
Danaus plexippus
Heliconius melpomene
Culex quinquefasciatus
Aedes aegypti
Anopheles gambiae
Anopheles darlingi
Drosophila virilis
Drosophila grimshawi
Drosophila willistoni
Drosophila pseudoobscura
Drosophila ananassae
Drosophila melanogaster
Drosophila yakuba
Strongylocentrotus purpuratus
Branchiostoma floridae
Ciona intestinalis
Ciona savignyi
Petromyzon marinus
Danio rerio
Gadus morhua
Oreochromis niloticus
Takifugu rubripes
Tetraodon nigroviridis
Gasterosteus aculeatus
Xiphophorus maculatus
Oryzias latipes
Latimeria chalumnae
Xenopus (Silurana) tropicalis
Pelodiscus sinensis
Anolis carolinensis
Taeniopygia guttata
Gallus gallus
Meleagris gallopavo
Ornithorhynchus anatinus
Monodelphis domestica
Sarcophilus harrisii
Macropus eugenii
Dasypus novemcinctus
Loxodonta africana
Echinops telfairi
Procavia capensis
Myotis lucifugus
Pteropus vampyrus
Sus scrofa
Tursiops truncatus
Bos taurus
Equus caballus
Felis catus
Canis lupus familiaris
Mustela putorius furo
Ailuropoda melanoleuca
Oryctolagus cuniculus
Cavia porcellus
Ictidomys tridecemlineatus
Dipodomys ordii
Rattus norvegicus
Mus musculus
Tupaia belangeri
Otolemur garnettii
Microcebus murinus
Tarsius syrichta
Callithrix jacchus
Macaca mulatta
Nomascus leucogenys
Pongo abelii
Gorilla gorilla
Pan troglodytes
Homo sapiens
Perkinsus marinus ATCC 50983
Tetrahymena thermophila
Paramecium tetraurelia strain d4 2
Neospora caninum Liverpool
Toxoplasma gondii
Cryptosporidium muris RN66
Cryptosporidium parvum Iowa II
Cryptosporidium hominis TU502
Theileria annulata strain Ankara
Theileria parva strain Muguga
Plasmodium falciparum
Plasmodium knowlesi
Plasmodium vivax
Plasmodium chabaudi
Plasmodium yoelii yoelii 17XNL
Plasmodium berghei
Phaeodactylum tricornutum
Thalassiosira pseudonana
Pythium ultimum
Albugo laibachii
Hyaloperonospora arabidopsidis
Phytophthora ramorum
Phytophthora infestans
Phytophthora sojae
Trypanosoma brucei
Trypanosoma cruzi strain CL Brener
Leishmania braziliensis MHOM BR 75 M2904
Leishmania mexicana MHOM GT 2001 U1103
Leishmania major
Leishmania donovani
Leishmania infantum JPCM5
Entamoeba histolytica
Entamoeba dispar SAW760
Dictyostelium discoideum
Dictyostelium purpureum
Trichomonas vaginalis G3
Trichomonas vaginalis
Naegleria gruberi
Giardia lamblia ATCC 50803
OG presence
OG absence
Inferred root
Dscore = 0.883
Pvalue = 1.14e−05
AdjPvalue = 1.36e−03

COG0446

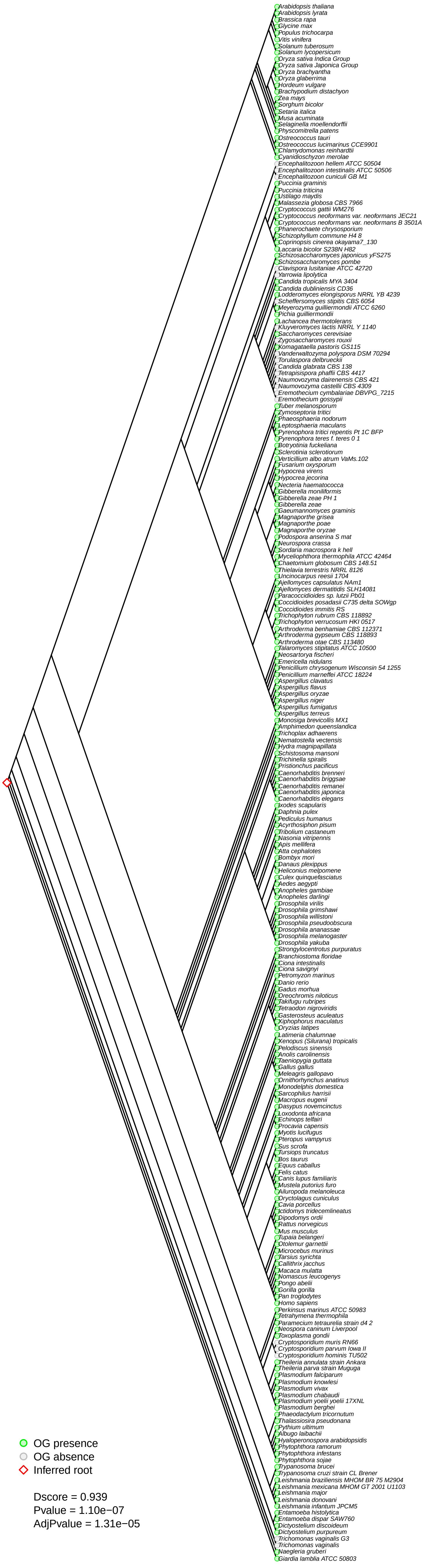

Arabidopsis thaliana
Arabidopsis lyrata
Brassica rapa
Glycine max
Populus trichocarpa
Vitis vinifera
Solanum tuberosum
Solanum lycopersicum
Oryza sativa Indica Group
Oryza sativa Japonica Group
Oryza brachyantha
Oryza glaberrima
Hordeum vulgare
Brachypodium distachyon
Zea mays
Sorghum bicolor
Setaria italica
Musa acuminata
Selaginella moellendorffii
Physcomitrella patens
Ostreococcus tauri
Ostreococcus lucimarinus CCE9901
Chlamydomonas reinhardtii
Cyanidioschyzon merolae
Encephalitozoon hellem ATCC 50504
Encephalitozoon intestinalis ATCC 50506
Encephalitozoon cuniculi GB M1
Puccinia graminis
Puccinia triticina
Ustilago maydis
Malassezia globosa CBS 7966
Cryptococcus gattii WM276
Cryptococcus neoformans var. neoformans JEC21
Cryptococcus neoformans var. neoformans B 3501A
Phanerochaete chrysosporium
Schizophyllum commune H4 8
Coprinopsis cinerea okayama7_130
Laccaria bicolor S238N H82
Schizosaccharomyces japonicus yFS275
Schizosaccharomyces pombe
Clavispora lusitaniae ATCC 42720
Yarrowia lipolytica
Candida tropicalis MYA 3404
Candida dubliniensis CD36
Lodderomyces elongisporus NRRL YB 4239
Scheffersomyces stipitis CBS 6054
Meyerozyma guilliermondii ATCC 6260
Pichia guilliermondii
Lachancea thermotolerans
Kluyveromyces lactis NRRL Y 1140
Saccharomyces cerevisiae
Zygosaccharomyces rouxii
Komagataella pastoris GS115
Vanderwaltozyma polyspora DSM 70294
Torulaspora delbrueckii
Candida glabrata CBS 138
Tetrapisispora phaffii CBS 4417
Naumovozyma dairenensis CBS 421
Naumovozyma castellii CBS 4309
Eremothecium cymbalariae DBVPG_7215
Eremothecium gossypii
Tuber melanosporum
Zymoseptoria tritici
Phaeosphaeria nodorum
Leptosphaeria maculans
Pyrenophora tritici repentis Pt 1C BFP
Pyrenophora teres f. teres 0 1
Botryotinia fuckeliana
Sclerotinia sclerotiorum
Verticillium albo atrum VaMs.102
Fusarium oxysporum
Hypocrea virens
Hypocrea jecorina
Nectria haematococca
Gibberella moniliformis
Gibberella zeae PH 1
Gibberella zeae
Gaeumannomyces graminis
Magnaporthe grisea
Magnaporthe poae
Magnaporthe oryzae
Podospora anserina S mat
Neurospora crassa
Sordaria macrospora k hell
Myceliophthora thermophila ATCC 42464
Chaetomium globosum CBS 148.51
Thielavia terrestris NRRL 8126
Uncinocarpus reesii 1704
Ajellomyces capsulatus NAm1
Ajellomyces dermatitidis SLH14081
Paracoccidioides sp. lutzii Pb01
Coccidioides posadasii C735 delta SOWgp
Coccidioides immitis RS
Trichophyton rubrum CBS 118892
Trichophyton verrucosum HKI 0517
Arthroderma benhamiae CBS 112371
Arthroderma gypseum CBS 118893
Arthroderma otae CBS 113480
Talaromyces stipitatus ATCC 10500
Neosartorya fischeri
Emericella nidulans
Penicillium chrysogenum Wisconsin 54 1255
Penicillium marneffei ATCC 18224
Aspergillus clavatus
Aspergillus flavus
Aspergillus oryzae
Aspergillus niger
Aspergillus fumigatus
Aspergillus terreus
Monosiga brevicollis MX1
Amphimedon queenslandica
Trichoplax adhaerens
Nematostella vectensis
Hydra magnipapillata
Schistosoma mansoni
Trichinella spiralis
Pristionchus pacificus
Caenorhabditis brenneri
Caenorhabditis briggsae
Caenorhabditis remanei
Caenorhabditis japonica
Caenorhabditis elegans
Ixodes scapularis
Daphnia pulex
Pediculus humanus
Acyrthosiphon pisum
Tribolium castaneum
Nasonia vitripennis
Apis mellifera
Atta cephalotes
Bombyx mori
Danaus plexippus
Heliconius melpomene
Culex quinquefasciatus
Aedes aegypti
Anopheles gambiae
Anopheles darlingi
Drosophila virilis
Drosophila grimshawi
Drosophila willistoni
Drosophila pseudoobscura
Drosophila ananassae
Drosophila melanogaster
Drosophila yakuba
Strongylocentrotus purpuratus
Branchiostoma floridae
Ciona intestinalis
Ciona savignyi
Petromyzon marinus
Danio rerio
Gadus morhua
Oreochromis niloticus
Takifugu rubripes
Tetraodon nigroviridis
Gasterosteus aculeatus
Xiphophorus maculatus
Oryzias latipes
Latimeria chalumnae
Xenopus (Silurana) tropicalis
Pelodiscus sinensis
Anolis carolinensis
Taeniopygia guttata
Gallus gallus
Meleagris gallopavo
Ornithorhynchus anatinus
Monodelphis domestica
Sarcophilus harrisii
Macropus eugenii
Dasypus novemcinctus
Loxodonta africana
Echinops telfairi
Procavia capensis
Myotis lucifugus
Pteropus vampyrus
Sus scrofa
Tursiops truncatus
Bos taurus
Equus caballus
Felis catus
Canis lupus familiaris
Mustela putorius furo
Ailuropoda melanoleuca
Oryctolagus cuniculus
Cavia porcellus
Ictidomys tridecemlineatus
Dipodomys ordii
Rattus norvegicus
Mus musculus
Tupaia belangeri
Otolemur garnettii
Microcebus murinus
Tarsius syrichta
Callithrix jacchus
Macaca mulatta
Nomascus leucogenys
Pongo abelii
Gorilla gorilla
Pan troglodytes
Homo sapiens
Perkinsus marinus ATCC 50983
Tetrahymena thermophila
Paramecium tetraurelia strain d4 2
Neospora caninum Liverpool
Toxoplasma gondii
Cryptosporidium muris RN66
Cryptosporidium parvum Iowa II
Cryptosporidium hominis TU502
Theileria annulata strain Ankara
Theileria parva strain Muguga
Plasmodium falciparum
Plasmodium knowlesi
Plasmodium vivax
Plasmodium chabaudi
Plasmodium yoelii yoelii 17XNL
Plasmodium berghei
Phaeodactylum tricornutum
Thalassiosira pseudonana
Pythium ultimum
Albugo laibachii
Hyaloperonospora arabidopsidis
Phytophthora ramorum
Phytophthora infestans
Phytophthora sojae
Trypanosoma brucei
Trypanosoma cruzi strain CL Brener
Leishmania braziliensis MHOM BR 75 M2904
Leishmania mexicana MHOM GT 2001 U1103
Leishmania major
Leishmania donovani
Leishmania infantum JPCM5
Entamoeba histolytica
Entamoeba dispar SAW760
Dictyostelium discoideum
Dictyostelium purpureum
Trichomonas vaginalis G3
Trichomonas vaginalis
Naegleria gruberi
Giardia lamblia ATCC 50803
OG presence
OG absence
Inferred root
Dscore = 0.939
Pvalue = 1.10e−07
AdjPvalue = 1.31e−05

COG0450

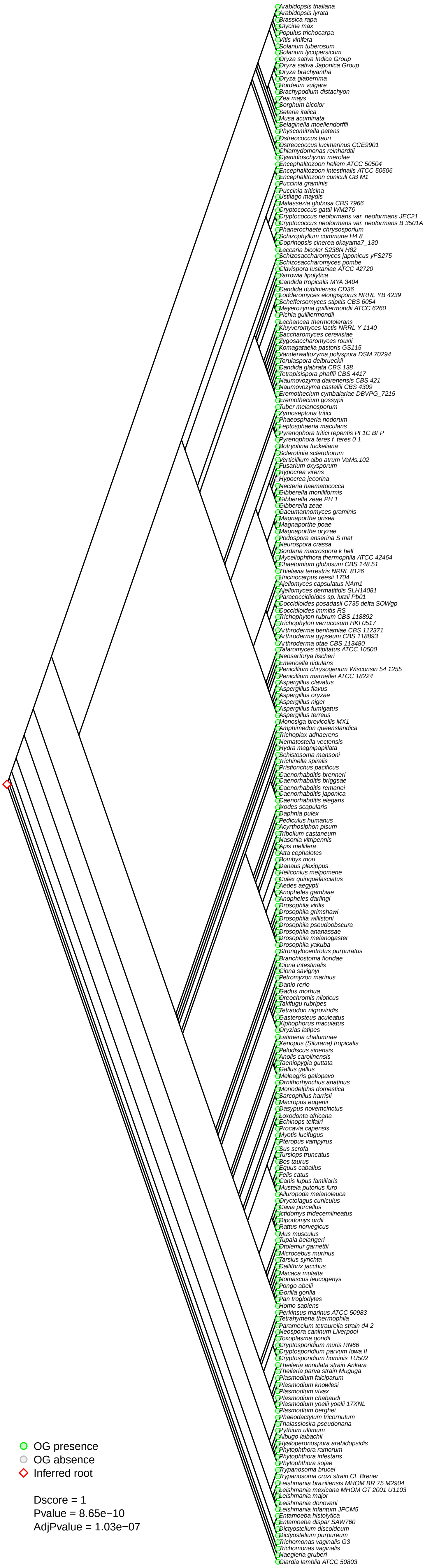
Arabidopsis thaliana
Arabidopsis lyrata
Brassica rapa
Glycine max
Populus trichocarpa
Vitis vinifera
Solanum tuberosum
Solanum lycopersicum
Oryza sativa Indica Group
Oryza sativa Japonica Group
Oryza brachyantha
Oryza glaberrima
Hordeum vulgare
Brachypodium distachyon
Zea mays
Sorghum bicolor
Setaria italica
Musa acuminata
Selaginella moellendorffii
Physcomitrella patens
Ostreococcus tauri
Ostreococcus lucimarinus CCE9901
Chlamydomonas reinhardtii
Cyanidioschyzon merolae
Encephalitozoon hellem ATCC 50504
Encephalitozoon intestinalis ATCC 50506
Encephalitozoon cuniculi GB M1
Puccinia graminis
Puccinia triticina
Ustilago maydis
Malassezia globosa CBS 7966
Cryptococcus gattii WM276
Cryptococcus neoformans var. neoformans JEC21
Cryptococcus neoformans var. neoformans B 3501A
Phanerochaete chrysosporium
Schizophyllum commune H4 8
Coprinopsis cinerea okayama7_130
Laccaria bicolor S238N H82
Schizosaccharomyces japonicus yFS275
Schizosaccharomyces pombe
Clavispora lusitaniae ATCC 42720
Yarrowia lipolytica
Candida tropicalis MYA 3404
Candida dubliniensis CD36
Lodderomyces elongisporus NRRL YB 4239
Scheffersomyces stipitis CBS 6054
Meyerozyma guilliermondii ATCC 6260
Pichia guilliermondii
Lachancea thermotolerans
Kluyveromyces lactis NRRL Y 1140
Saccharomyces cerevisiae
Zygosaccharomyces rouxii
Komagataella pastoris GS115
Vanderwaltozyma polyspora DSM 70294
Torulaspora delbrueckii
Candida glabrata CBS 138
Tetrapisispora phaffii CBS 4417
Naumovozyma dairenensis CBS 421
Naumovozyma castellii CBS 4309
Eremothecium cymbalariae DBVPG_7215
Eremothecium gossypii
Tuber melanosporum
Zymoseptoria tritici
Phaeosphaeria nodorum
Leptosphaeria maculans
Pyrenophora tritici repentis Pt 1C BFP
Pyrenophora teres f. teres 0 1
Botryotinia fuckeliana
Sclerotinia sclerotiorum
Verticillium albo atrum VaMs.102
Fusarium oxysporum
Hypocrea virens
Hypocrea jecorina
Nectria haematococca
Gibberella moniliformis
Gibberella zeae PH 1
Gibberella zeae
Gaeumannomyces graminis
Magnaporthe grisea
Magnaporthe poae
Magnaporthe oryzae
Podospora anserina S mat
Neurospora crassa
Sordaria macrospora k hell
Myceliophthora thermophila ATCC 42464
Chaetomium globosum CBS 148.51
Thielavia terrestris NRRL 8126
Uncinocarpus reesii 1704
Ajellomyces capsulatus NAm1
Ajellomyces dermatitidis SLH14081
Paracoccidioides sp. lutzii Pb01
Coccidioides posadasii C735 delta SOWgp
Coccidioides immitis RS
Trichophyton rubrum CBS 118892
Trichophyton verrucosum HKI 0517
Arthroderma benhamiae CBS 112371
Arthroderma gypseum CBS 118893
Arthroderma otae CBS 113480
Talaromyces stipitatus ATCC 10500
Neosartorya fischeri
Emericella nidulans
Penicillium chrysogenum Wisconsin 54 1255
Penicillium marneffei ATCC 18224
Aspergillus clavatus
Aspergillus flavus
Aspergillus oryzae
Aspergillus niger
Aspergillus fumigatus
Aspergillus terreus
Monosiga brevicollis MX1
Amphimedon queenslandica
Trichoplax adhaerens
Nematostella vectensis
Hydra magnipapillata
Schistosoma mansoni
Trichinella spiralis
Pristionchus pacificus
Caenorhabditis brenneri
Caenorhabditis briggsae
Caenorhabditis remanei
Caenorhabditis japonica
Caenorhabditis elegans
Ixodes scapularis
Daphnia pulex
Pediculus humanus
Acyrthosiphon pisum
Tribolium castaneum
Nasonia vitripennis
Apis mellifera
Atta cephalotes
Bombyx mori
Danaus plexippus
Heliconius melpomene
Culex quinquefasciatus
Aedes aegypti
Anopheles gambiae
Anopheles darlingi
Drosophila virilis
Drosophila grimshawi
Drosophila willistoni
Drosophila pseudoobscura
Drosophila ananassae
Drosophila melanogaster
Drosophila yakuba
Strongylocentrotus purpuratus
Branchiostoma floridae
Ciona intestinalis
Ciona savignyi
Petromyzon marinus
Danio rerio
Gadus morhua
Oreochromis niloticus
Takifugu rubripes
Tetraodon nigroviridis
Gasterosteus aculeatus
Xiphophorus maculatus
Oryzias latipes
Latimeria chalumnae
Xenopus (Silurana) tropicalis
Pelodiscus sinensis
Anolis carolinensis
Taeniopygia guttata
Gallus gallus
Meleagris gallopavo
Ornithorhynchus anatinus
Monodelphis domestica
Sarcophilus harrisii
Macropus eugenii
Dasypus novemcinctus
Loxodonta africana
Echinops telfairi
Procavia capensis
Myotis lucifugus
Pteropus vampyrus
Sus scrofa
Tursiops truncatus
Bos taurus
Equus caballus
Felis catus
Canis lupus familiaris
Mustela putorius furo
Ailuropoda melanoleuca
Oryctolagus cuniculus
Cavia porcellus
Ictidomys tridecemlineatus
Dipodomys ordii
Rattus norvegicus
Mus musculus
Tupaia belangeri
Otolemur garnettii
Microcebus murinus
Tarsius syrichta
Callithrix jacchus
Macaca mulatta
Nomascus leucogenys
Pongo abelii
Gorilla gorilla
Pan troglodytes
Homo sapiens
Perkinsus marinus ATCC 50983
Tetrahymena thermophila
Paramecium tetraurelia strain d4 2
Neospora caninum Liverpool
Toxoplasma gondii
Cryptosporidium muris RN66
Cryptosporidium parvum Iowa II
Cryptosporidium hominis TU502
Theileria annulata strain Ankara
Theileria parva strain Muguga
Plasmodium falciparum
Plasmodium knowlesi
Plasmodium vivax
Plasmodium chabaudi
Plasmodium yoelii yoelii 17XNL
Plasmodium berghei
Phaeodactylum tricornutum
Thalassiosira pseudonana
Pythium ultimum
Albugo laibachii
Hyaloperonospora arabidopsidis
Phytophthora ramorum
Phytophthora infestans
Phytophthora sojae
Trypanosoma brucei
Trypanosoma cruzi strain CL Brener
Leishmania braziliensis MHOM BR 75 M2904
Leishmania mexicana MHOM GT 2001 U1103
Leishmania major
Leishmania donovani
Leishmania infantum JPCM5
Entamoeba histolytica
Entamoeba dispar SAW760
Dictyostelium discoideum
Dictyostelium purpureum
Trichomonas vaginalis G3
Trichomonas vaginalis
Naegleria gruberi
Giardia lamblia ATCC 50803
OG presence
OG absence
Inferred root
Dscore = 1
Pvalue = 8.65e−10
AdjPvalue = 1.03e−07

COG0492

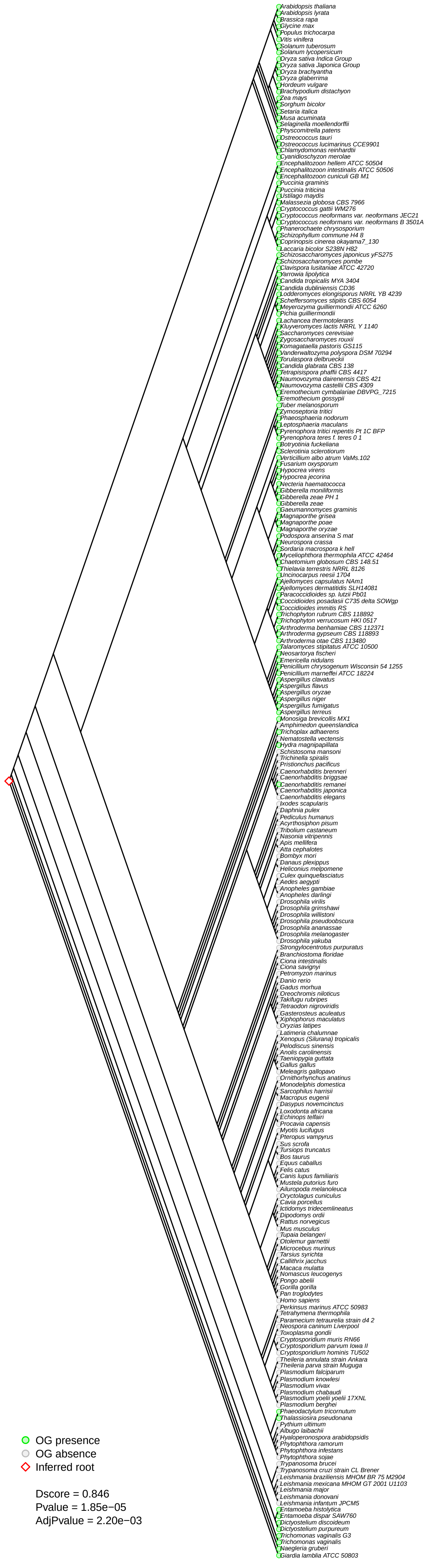
Arabidopsis thaliana
Arabidopsis lyrata
Brassica rapa
Glycine max
Populus trichocarpa
Vitis vinifera
Solanum tuberosum
Solanum lycopersicum
Oryza sativa Indica Group
Oryza sativa Japonica Group
Oryza brachyantha
Oryza glaberrima
Hordeum vulgare
Brachypodium distachyon
Zea mays
Sorghum bicolor
Setaria italica
Musa acuminata
Selaginella moellendorffii
Physcomitrella patens
Ostreococcus tauri
Ostreococcus lucimarinus CCE9901
Chlamydomonas reinhardtii
Cyanidioschyzon merolae
Encephalitozoon hellem ATCC 50504
Encephalitozoon intestinalis ATCC 50506
Encephalitozoon cuniculi GB M1
Puccinia graminis
Puccinia triticina
Ustilago maydis
Malassezia globosa CBS 7966
Cryptococcus gattii WM276
Cryptococcus neoformans var. neoformans JEC21
Cryptococcus neoformans var. neoformans B 3501A
Phanerochaete chrysosporium
Schizophyllum commune H4 8
Coprinopsis cinerea okayama7_130
Laccaria bicolor S238N H82
Schizosaccharomyces japonicus yFS275
Schizosaccharomyces pombe
Clavispora lusitaniae ATCC 42720
Yarrowia lipolytica
Candida tropicalis MYA 3404
Candida dubliniensis CD36
Lodderomyces elongisporus NRRL YB 4239
Scheffersomyces stipitis CBS 6054
Meyerozyma guilliermondii ATCC 6260
Pichia guilliermondii
Lachancea thermotolerans
Kluyveromyces lactis NRRL Y 1140
Saccharomyces cerevisiae
Zygosaccharomyces rouxii
Komagataella pastoris GS115
Vanderwaltozyma polyspora DSM 70294
Torulaspora delbrueckii
Candida glabrata CBS 138
Tetrapisispora phaffii CBS 4417
Naumovozyma dairenensis CBS 421
Naumovozyma castellii CBS 4309
Eremothecium cymbalariae DBVPG_7215
Eremothecium gossypii
Tuber melanosporum
Zymoseptoria tritici
Phaeosphaeria nodorum
Leptosphaeria maculans
Pyrenophora tritici repentis Pt 1C BFP
Pyrenophora teres f. teres 0 1
Botryotinia fuckeliana
Sclerotinia sclerotiorum
Verticillium albo atrum VaMs.102
Fusarium oxysporum
Hypocrea virens
Hypocrea jecorina
Nectria haematococca
Gibberella moniliformis
Gibberella zeae PH 1
Gibberella zeae
Gaeumannomyces graminis
Magnaporthe grisea
Magnaporthe poae
Magnaporthe oryzae
Podospora anserina S mat
Neurospora crassa
Sordaria macrospora k hell
Myceliophthora thermophila ATCC 42464
Chaetomium globosum CBS 148.51
Thielavia terrestris NRRL 8126
Uncinocarpus reesii 1704
Ajellomyces capsulatus NAm1
Ajellomyces dermatitidis SLH14081
Paracoccidioides sp. lutzii Pb01
Coccidioides posadasii C735 delta SOWgp
Coccidioides immitis RS
Trichophyton rubrum CBS 118892
Trichophyton verrucosum HKI 0517
Arthroderma benhamiae CBS 112371
Arthroderma gypseum CBS 118893
Arthroderma otae CBS 113480
Talaromyces stipitatus ATCC 10500
Neosartorya fischeri
Emericella nidulans
Penicillium chrysogenum Wisconsin 54 1255
Penicillium marneffei ATCC 18224
Aspergillus clavatus
Aspergillus flavus
Aspergillus oryzae
Aspergillus niger
Aspergillus fumigatus
Aspergillus terreus
Monosiga brevicollis MX1
Amphimedon queenslandica
Trichoplax adhaerens
Nematostella vectensis
Hydra magnipapillata
Schistosoma mansoni
Trichinella spiralis
Pristionchus pacificus
Caenorhabditis brenneri
Caenorhabditis briggsae
Caenorhabditis remanei
Caenorhabditis japonica
Caenorhabditis elegans
Ixodes scapularis
Daphnia pulex
Pediculus humanus
Acyrthosiphon pisum
Tribolium castaneum
Nasonia vitripennis
Apis mellifera
Atta cephalotes
Bombyx mori
Danaus plexippus
Heliconius melpomene
Culex quinquefasciatus
Aedes aegypti
Anopheles gambiae
Anopheles darlingi
Drosophila virilis
Drosophila grimshawi
Drosophila willistoni
Drosophila pseudoobscura
Drosophila ananassae
Drosophila melanogaster
Drosophila yakuba
Strongylocentrotus purpuratus
Branchiostoma floridae
Ciona intestinalis
Ciona savignyi
Petromyzon marinus
Danio rerio
Gadus morhua
Oreochromis niloticus
Takifugu rubripes
Tetraodon nigroviridis
Gasterosteus aculeatus
Xiphophorus maculatus
Oryzias latipes
Latimeria chalumnae
Xenopus (Silurana) tropicalis
Pelodiscus sinensis
Anolis carolinensis
Taeniopygia guttata
Gallus gallus
Meleagris gallopavo
Ornithorhynchus anatinus
Monodelphis domestica
Sarcophilus harrisii
Macropus eugenii
Dasypus novemcinctus
Loxodonta africana
Echinops telfairi
Procavia capensis
Myotis lucifugus
Pteropus vampyrus
Sus scrofa
Tursiops truncatus
Bos taurus
Equus caballus
Felis catus
Canis lupus familiaris
Mustela putorius furo
Ailuropoda melanoleuca
Oryctolagus cuniculus
Cavia porcellus
Ictidomys tridecemlineatus
Dipodomys ordii
Rattus norvegicus
Mus musculus
Tupaia belangeri
Otolemur garnettii
Microcebus murinus
Tarsius syrichta
Callithrix jacchus
Macaca mulatta
Nomascus leucogenys
Pongo abelii
Gorilla gorilla
Pan troglodytes
Homo sapiens
Perkinsus marinus ATCC 50983
Tetrahymena thermophila
Paramecium tetraurelia strain d4 2
Neospora caninum Liverpool
Toxoplasma gondii
Cryptosporidium muris RN66
Cryptosporidium parvum Iowa II
Cryptosporidium hominis TU502
Theileria annulata strain Ankara
Theileria parva strain Muguga
Plasmodium falciparum
Plasmodium knowlesi
Plasmodium vivax
Plasmodium chabaudi
Plasmodium yoelii yoelii 17XNL
Plasmodium berghei
Phaeodactylum tricornutum
Thalassiosira pseudonana
Pythium ultimum
Albugo laibachii
Hyaloperonospora arabidopsidis
Phytophthora ramorum
Phytophthora infestans
Phytophthora sojae
Trypanosoma brucei
Trypanosoma cruzi strain CL Brener
Leishmania braziliensis MHOM BR 75 M2904
Leishmania mexicana MHOM GT 2001 U1103
Leishmania major
Leishmania donovani
Leishmania infantum JPCM5
Entamoeba histolytica
Entamoeba dispar SAW760
Dictyostelium discoideum
Dictyostelium purpureum
Trichomonas vaginalis G3
Trichomonas vaginalis
Naegleria gruberi
Giardia lamblia ATCC 50803
OG presence
OG absence
Inferred root
Dscore = 0.846
Pvalue = 1.85e−05
AdjPvalue = 2.20e−03

COG0526

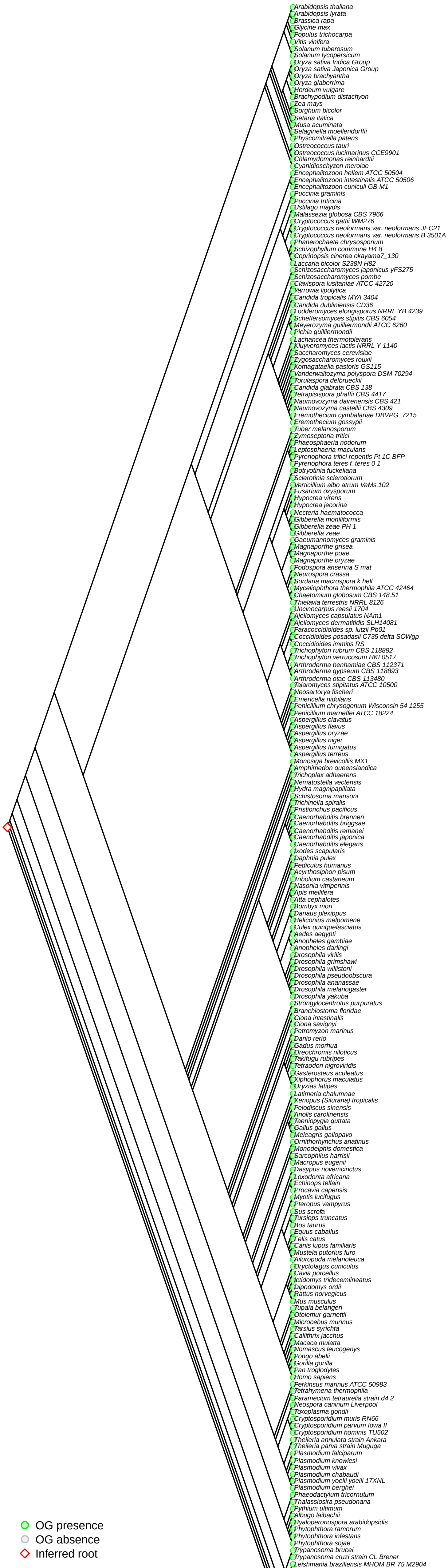

Dscore = 1
Pvalue = 1.92e−09
AdjPvalue = 2.29e−07

COG0605

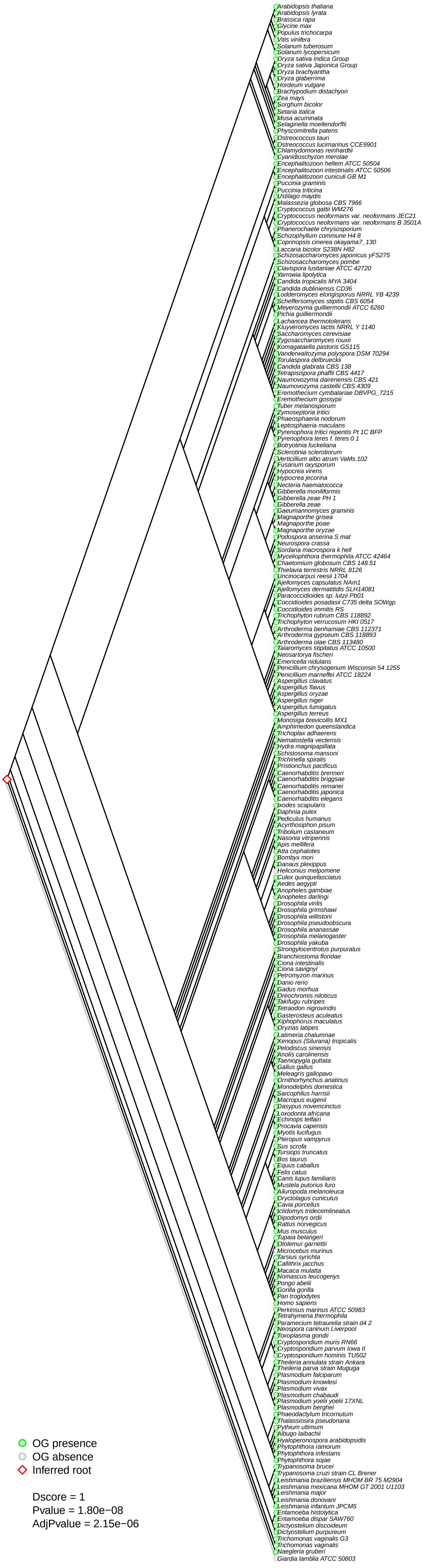
Arabidopsis thaliana
Arabidopsis lyrata
Brassica rapa
Glycine max
Populus trichocarpa
Vitis vinifera
Solanum tuberosum
Solanum lycopersicum
Oryza sativa Indica Group
Oryza sativa Japonica Group
Oryza brachyantha
Oryza glaberrima
Hordeum vulgare
Brachypodium distachyon
Zea mays
Sorghum bicolor
Setaria italica
Musa acuminata
Selaginella moellendorffii
Physcomitrella patens
Ostreococcus tauri
Ostreococcus lucimarinus CCE9901
Chlamydomonas reinhardtii
Cyanidioschyzon merolae
Encephalitozoon hellem ATCC 50504
Encephalitozoon intestinalis ATCC 50506
Encephalitozoon cuniculi GB M1
Puccinia graminis
Puccinia triticina
Ustilago maydis
Malassezia globosa CBS 7966
Cryptococcus gattii WM276
Cryptococcus neoformans var. neoformans JEC21
Cryptococcus neoformans var. neoformans B 3501A
Phanerochaete chrysosporium
Schizophyllum commune H4 8
Coprinopsis cinerea okayama7_130
Laccaria bicolor S238N H82
Schizosaccharomyces japonicus yFS275
Schizosaccharomyces pombe
Clavispora lusitaniae ATCC 42720
Yarrowia lipolytica
Candida tropicalis MYA 3404
Candida dubliniensis CD36
Lodderomyces elongisporus NRRL YB 4239
Scheffersomyces stipitis CBS 6054
Meyerozyma guilliermondii ATCC 6260
Pichia guilliermondii
Lachancea thermotolerans
Kluyveromyces lactis NRRL Y 1140
Saccharomyces cerevisiae
Zygosaccharomyces rouxii
Komagataella pastoris GS115
Vanderwaltozyma polyspora DSM 70294
Torulaspora delbrueckii
Candida glabrata CBS 138
Tetrapisispora phaffii CBS 4417
Naumovozyma dairenensis CBS 421
Naumovozyma castellii CBS 4309
Eremothecium cymbalariae DBVPG_7215
Eremothecium gossypii
Tuber melanosporum
Zymoseptoria tritici
Phaeosphaeria nodorum
Leptosphaeria maculans
Pyrenophora tritici repentis Pt 1C BFP
Pyrenophora teres f. teres 0 1
Botryotinia fuckeliana
Sclerotinia sclerotiorum
Verticillium albo atrum VaMs.102
Fusarium oxysporum
Hypocrea virens
Hypocrea jecorina
Nectria haematococca
Gibberella moniliformis
Gibberella zeae PH 1
Gibberella zeae
Gaeumannomyces graminis
Magnaporthe grisea
Magnaporthe poae
Magnaporthe oryzae
Podospora anserina S mat
Neurospora crassa
Sordaria macrospora k hell
Myceliophthora thermophila ATCC 42464
Chaetomium globosum CBS 148.51
Thielavia terrestris NRRL 8126
Uncinocarpus reesii 1704
Ajellomyces capsulatus NAm1
Ajellomyces dermatitidis SLH14081
Paracoccidioides sp. lutzii Pb01
Coccidioides posadasii C735 delta SOWgp
Coccidioides immitis RS
Trichophyton rubrum CBS 118892
Trichophyton verrucosum HKI 0517
Arthroderma benhamiae CBS 112371
Arthroderma gypseum CBS 118893
Arthroderma otae CBS 113480
Talaromyces stipitatus ATCC 10500
Neosartorya fischeri
Emericella nidulans
Penicillium chrysogenum Wisconsin 54 1255
Penicillium marneffei ATCC 18224
Aspergillus clavatus
Aspergillus flavus
Aspergillus oryzae
Aspergillus niger
Aspergillus fumigatus
Aspergillus terreus
Monosiga brevicollis MX1
Amphimedon queenslandica
Trichoplax adhaerens
Nematostella vectensis
Hydra magnipapillata
Schistosoma mansoni
Trichinella spiralis
Pristionchus pacificus
Caenorhabditis brenneri
Caenorhabditis briggsae
Caenorhabditis remanei
Caenorhabditis japonica
Caenorhabditis elegans
Ixodes scapularis
Daphnia pulex
Pediculus humanus
Acyrthosiphon pisum
Tribolium castaneum
Nasonia vitripennis
Apis mellifera
Atta cephalotes
Bombyx mori
Danaus plexippus
Heliconius melpomene
Culex quinquefasciatus
Aedes aegypti
Anopheles gambiae
Anopheles darlingi
Drosophila virilis
Drosophila grimshawi
Drosophila willistoni
Drosophila pseudoobscura
Drosophila ananassae
Drosophila melanogaster
Drosophila yakuba
Strongylocentrotus purpuratus
Branchiostoma floridae
Ciona intestinalis
Ciona savignyi
Petromyzon marinus
Danio rerio
Gadus morhua
Oreochromis niloticus
Takifugu rubripes
Tetraodon nigroviridis
Gasterosteus aculeatus
Xiphophorus maculatus
Oryzias latipes
Latimeria chalumnae
Xenopus (Silurana) tropicalis
Pelodiscus sinensis
Anolis carolinensis
Taeniopygia guttata
Gallus gallus
Meleagris gallopavo
Ornithorhynchus anatinus
Monodelphis domestica
Sarcophilus harrisii
Macropus eugenii
Dasypus novemcinctus
Loxodonta africana
Echinops telfairi
Procavia capensis
Myotis lucifugus
Pteropus vampyrus
Sus scrofa
Tursiops truncatus
Bos taurus
Equus caballus
Felis catus
Canis lupus familiaris
Mustela putorius furo
Ailuropoda melanoleuca
Oryctolagus cuniculus
Cavia porcellus
Ictidomys tridecemlineatus
Dipodomys ordii
Rattus norvegicus
Mus musculus
Tupaia belangeri
Otolemur garnettii
Microcebus murinus
Tarsius syrichta
Callithrix jacchus
Macaca mulatta
Nomascus leucogenys
Pongo abelii
Gorilla gorilla
Pan troglodytes
Homo sapiens
Perkinsus marinus ATCC 50983
Tetrahymena thermophila
Paramecium tetraurelia strain d4 2
Neospora caninum Liverpool
Toxoplasma gondii
Cryptosporidium muris RN66
Cryptosporidium parvum Iowa II
Cryptosporidium hominis TU502
Theileria annulata strain Ankara
Theileria parva strain Muguga
Plasmodium falciparum
Plasmodium knowlesi
Plasmodium vivax
Plasmodium chabaudi
Plasmodium yoelii yoelii 17XNL
Plasmodium berghei
Phaeodactylum tricornutum
Thalassiosira pseudonana
Pythium ultimum
Albugo laibachii
Hyaloperonospora arabidopsidis
Phytophthora ramorum
Phytophthora infestans
Phytophthora sojae
Trypanosoma brucei
Trypanosoma cruzi strain CL Brener
Leishmania braziliensis MHOM BR 75 M2904
Leishmania mexicana MHOM GT 2001 U1103
Leishmania major
Leishmania donovani
Leishmania infantum JPCM5
Entamoeba histolytica
Entamoeba dispar SAW760
Dictyostelium discoideum
Dictyostelium purpureum
Trichomonas vaginalis G3
Trichomonas vaginalis
Naegleria gruberi
Giardia lamblia ATCC 50803
OG presence
OG absence
Inferred root
Dscore = 1
Pvalue = 1.80e−08
AdjPvalue = 2.15e−06

COG0678

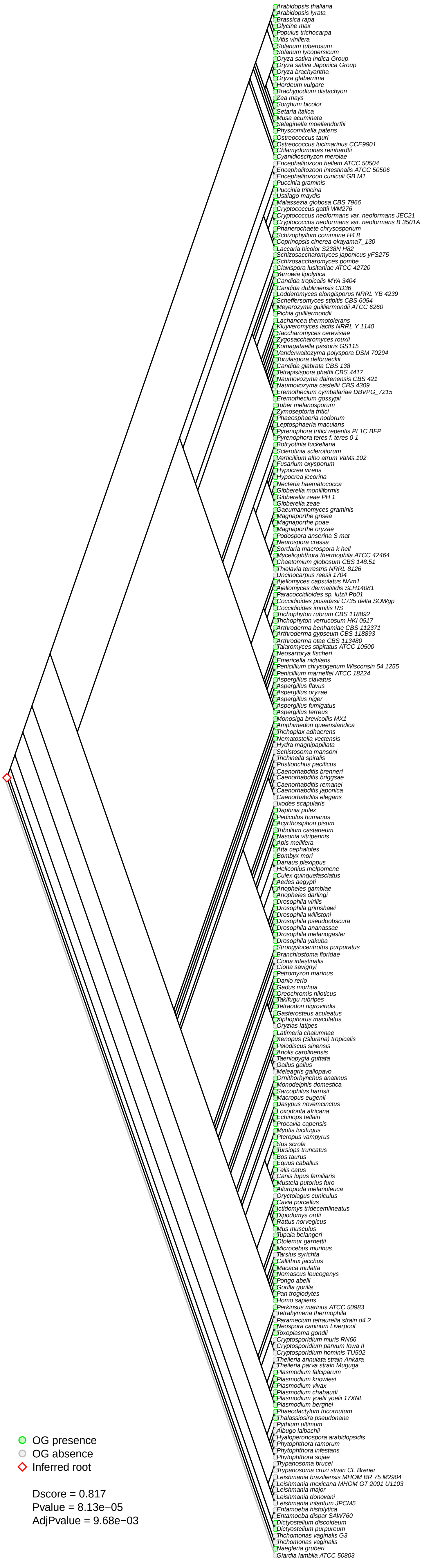

Arabidopsis thaliana
Arabidopsis lyrata
Brassica rapa
Glycine max
Populus trichocarpa
Vitis vinifera
Solanum tuberosum
Solanum lycopersicum
Oryza sativa Indica Group
Oryza sativa Japonica Group
Oryza brachyantha
Oryza glaberrima
Hordeum vulgare
Brachypodium distachyon
Zea mays
Sorghum bicolor
Setaria italica
Musa acuminata
Selaginella moellendorffii
Physcomitrella patens
Ostreococcus tauri
Ostreococcus lucimarinus CCE9901
Chlamydomonas reinhardtii
Cyanidioschyzon merolae
Encephalitozoon hellem ATCC 50504
Encephalitozoon intestinalis ATCC 50506
Encephalitozoon cuniculi GB M1
Puccinia graminis
Puccinia triticina
Ustilago maydis
Malassezia globosa CBS 7966
Cryptococcus gattii WM276
Cryptococcus neoformans var. neoformans JEC21
Cryptococcus neoformans var. neoformans B 3501A
Phanerochaete chrysosporium
Schizophyllum commune H4 8
Coprinopsis cinerea okayama7_130
Laccaria bicolor S238N H82
Schizosaccharomyces japonicus yFS275
Schizosaccharomyces pombe
Clavispora lusitaniae ATCC 42720
Yarrowia lipolytica
Candida tropicalis MYA 3404
Candida dubliniensis CD36
Lodderomyces elongisporus NRRL YB 4239
Scheffersomyces stipitis CBS 6054
Meyerozyma guilliermondii ATCC 6260
Pichia guilliermondii
Lachancea thermotolerans
Kluyveromyces lactis NRRL Y 1140
Saccharomyces cerevisiae
Zygosaccharomyces rouxii
Komagataella pastoris GS115
Vanderwaltozyma polyspora DSM 70294
Torulaspora delbrueckii
Candida glabrata CBS 138
Tetrapisispora phaffii CBS 4417
Naumovozyma dairenensis CBS 421
Naumovozyma castellii CBS 4309
Eremothecium cymbalariae DBVPG_7215
Eremothecium gossypii
Tuber melanosporum
Zymoseptoria tritici
Phaeosphaeria nodorum
Leptosphaeria maculans
Pyrenophora tritici repentis Pt 1C BFP
Pyrenophora teres f. teres 0 1
Botryotinia fuckeliana
Sclerotinia sclerotiorum
Verticillium albo atrum VaMs.102
Fusarium oxysporum
Hypocrea virens
Hypocrea jecorina
Nectria haematococca
Gibberella moniliformis
Gibberella zeae PH 1
Gibberella zeae
Gaeumannomyces graminis
Magnaporthe grisea
Magnaporthe poae
Magnaporthe oryzae
Podospora anserina S mat
Neurospora crassa
Sordaria macrospora k hell
Myceliophthora thermophila ATCC 42464
Chaetomium globosum CBS 148.51
Thielavia terrestris NRRL 8126
Uncinocarpus reesii 1704
Ajellomyces capsulatus NAm1
Ajellomyces dermatitidis SLH14081
Paracoccidioides sp. lutzii Pb01
Coccidioides posadasii C735 delta SOWgp
Coccidioides immitis RS
Trichophyton rubrum CBS 118892
Trichophyton verrucosum HKI 0517
Arthroderma benhamiae CBS 112371
Arthroderma gypseum CBS 118893
Arthroderma otae CBS 113480
Talaromyces stipitatus ATCC 10500
Neosartorya fischeri
Emericella nidulans
Penicillium chrysogenum Wisconsin 54 1255
Penicillium marneffei ATCC 18224
Aspergillus clavatus
Aspergillus flavus
Aspergillus oryzae
Aspergillus niger
Aspergillus fumigatus
Aspergillus terreus
Monosiga brevicollis MX1
Amphimedon queenslandica
Trichoplax adhaerens
Nematostella vectensis
Hydra magnipapillata
Schistosoma mansoni
Trichinella spiralis
Pristionchus pacificus
Caenorhabditis brenneri
Caenorhabditis briggsae
Caenorhabditis remanei
Caenorhabditis japonica
Caenorhabditis elegans
Ixodes scapularis
Daphnia pulex
Pediculus humanus
Acyrthosiphon pisum
Tribolium castaneum
Nasonia vitripennis
Apis mellifera
Atta cephalotes
Bombyx mori
Danaus plexippus
Heliconius melpomene
Culex quinquefasciatus
Aedes aegypti
Anopheles gambiae
Anopheles darlingi
Drosophila virilis
Drosophila grimshawi
Drosophila willistoni
Drosophila pseudoobscura
Drosophila ananassae
Drosophila melanogaster
Drosophila yakuba
Strongylocentrotus purpuratus
Branchiostoma floridae
Ciona intestinalis
Ciona savignyi
Petromyzon marinus
Danio rerio
Gadus morhua
Oreochromis niloticus
Takifugu rubripes
Tetraodon nigroviridis
Gasterosteus aculeatus
Xiphophorus maculatus
Oryzias latipes
Latimeria chalumnae
Xenopus (Silurana) tropicalis
Pelodiscus sinensis
Anolis carolinensis
Taeniopygia guttata
Gallus gallus
Meleagris gallopavo
Ornithorhynchus anatinus
Monodelphis domestica
Sarcophilus harrisii
Macropus eugenii
Dasypus novemcinctus
Loxodonta africana
Echinops telfairi
Procavia capensis
Myotis lucifugus
Pteropus vampyrus
Sus scrofa
Tursiops truncatus
Bos taurus
Equus caballus
Felis catus
Canis lupus familiaris
Mustela putorius furo
Ailuropoda melanoleuca
Oryctolagus cuniculus
Cavia porcellus
Ictidomys tridecemlineatus
Dipodomys ordii
Rattus norvegicus
Mus musculus
Tupaia belangeri
Otolemur garnettii
Microcebus murinus
Tarsius syrichta
Callithrix jacchus
Macaca mulatta
Nomascus leucogenys
Pongo abelii
Gorilla gorilla
Pan troglodytes
Homo sapiens
Perkinsus marinus ATCC 50983
Tetrahymena thermophila
Paramecium tetraurelia strain d4 2
Neospora caninum Liverpool
Toxoplasma gondii
Cryptosporidium muris RN66
Cryptosporidium parvum Iowa II
Cryptosporidium hominis TU502
Theileria annulata strain Ankara
Theileria parva strain Muguga
Plasmodium falciparum
Plasmodium knowlesi
Plasmodium vivax
Plasmodium chabaudi
Plasmodium yoelii yoelii 17XNL
Plasmodium berghei
Phaeodactylum tricornutum
Thalassiosira pseudonana
Pythium ultimum
Albugo laibachii
Hyaloperonospora arabidopsidis
Phytophthora ramorum
Phytophthora infestans
Phytophthora sojae
Trypanosoma brucei
Trypanosoma cruzi strain CL Brener
Leishmania braziliensis MHOM BR 75 M2904
Leishmania mexicana MHOM GT 2001 U1103
Leishmania major
Leishmania donovani
Leishmania infantum JPCM5
Entamoeba histolytica
Entamoeba dispar SAW760
Dictyostelium discoideum
Dictyostelium purpureum
Trichomonas vaginalis G3
Trichomonas vaginalis
Naegleria gruberi
Giardia lamblia ATCC 50803
OG presence
OG absence
Inferred root
Dscore = 0.817
Pvalue = 8.13e−05
AdjPvalue = 9.68e−03

COG0695

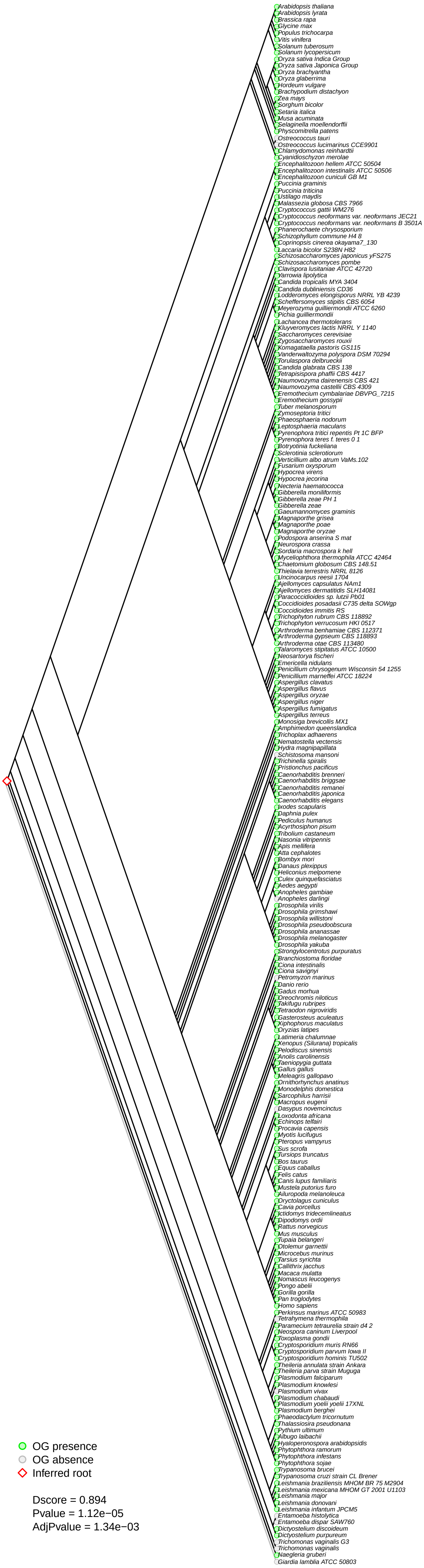
Arabidopsis thaliana
Arabidopsis lyrata
Brassica rapa
Glycine max
Populus trichocarpa
Vitis vinifera
Solanum tuberosum
Solanum lycopersicum
Oryza sativa Indica Group
Oryza sativa Japonica Group
Oryza brachyantha
Oryza glaberrima
Hordeum vulgare
Brachypodium distachyon
Zea mays
Sorghum bicolor
Setaria italica
Musa acuminata
Selaginella moellendorffii
Physcomitrella patens
Ostreococcus tauri
Ostreococcus lucimarinus CCE9901
Chlamydomonas reinhardtii
Cyanidioschyzon merolae
Encephalitozoon hellem ATCC 50504
Encephalitozoon intestinalis ATCC 50506
Encephalitozoon cuniculi GB M1
Puccinia graminis
Puccinia triticina
Ustilago maydis
Malassezia globosa CBS 7966
Cryptococcus gattii WM276
Cryptococcus neoformans var. neoformans JEC21
Cryptococcus neoformans var. neoformans B 3501A
Phanerochaete chrysosporium
Schizophyllum commune H4 8
Coprinopsis cinerea okayama7_130
Laccaria bicolor S238N H82
Schizosaccharomyces japonicus yFS275
Schizosaccharomyces pombe
Clavispora lusitaniae ATCC 42720
Yarrowia lipolytica
Candida tropicalis MYA 3404
Candida dubliniensis CD36
Lodderomyces elongisporus NRRL YB 4239
Scheffersomyces stipitis CBS 6054
Meyerozyma guilliermondii ATCC 6260
Pichia guilliermondii
Lachancea thermotolerans
Kluyveromyces lactis NRRL Y 1140
Saccharomyces cerevisiae
Zygosaccharomyces rouxii
Komagataella pastoris GS115
Vanderwaltozyma polyspora DSM 70294
Torulaspora delbrueckii
Candida glabrata CBS 138
Tetrapisispora phaffii CBS 4417
Naumovozyma dairenensis CBS 421
Naumovozyma castellii CBS 4309
Eremothecium cymbalariae DBVPG_7215
Eremothecium gossypii
Tuber melanosporum
Zymoseptoria tritici
Phaeosphaeria nodorum
Leptosphaeria maculans
Pyrenophora tritici repentis Pt 1C BFP
Pyrenophora teres f. teres 0 1
Botryotinia fuckeliana
Sclerotinia sclerotiorum
Verticillium albo atrum VaMs.102
Fusarium oxysporum
Hypocrea virens
Hypocrea jecorina
Nectria haematococca
Gibberella moniliformis
Gibberella zeae PH 1
Gibberella zeae
Gaeumannomyces graminis
Magnaporthe grisea
Magnaporthe poae
Magnaporthe oryzae
Podospora anserina S mat
Neurospora crassa
Sordaria macrospora k hell
Myceliophthora thermophila ATCC 42464
Chaetomium globosum CBS 148.51
Thielavia terrestris NRRL 8126
Uncinocarpus reesii 1704
Ajellomyces capsulatus NAm1
Ajellomyces dermatitidis SLH14081
Paracoccidioides sp. lutzii Pb01
Coccidioides posadasii C735 delta SOWgp
Coccidioides immitis RS
Trichophyton rubrum CBS 118892
Trichophyton verrucosum HKI 0517
Arthroderma benhamiae CBS 112371
Arthroderma gypseum CBS 118893
Arthroderma otae CBS 113480
Talaromyces stipitatus ATCC 10500
Neosartorya fischeri
Emericella nidulans
Penicillium chrysogenum Wisconsin 54 1255
Penicillium marneffei ATCC 18224
Aspergillus clavatus
Aspergillus flavus
Aspergillus oryzae
Aspergillus niger
Aspergillus fumigatus
Aspergillus terreus
Monosiga brevicollis MX1
Amphimedon queenslandica
Trichoplax adhaerens
Nematostella vectensis
Hydra magnipapillata
Schistosoma mansoni
Trichinella spiralis
Pristionchus pacificus
Caenorhabditis brenneri
Caenorhabditis briggsae
Caenorhabditis remanei
Caenorhabditis japonica
Caenorhabditis elegans
Ixodes scapularis
Daphnia pulex
Pediculus humanus
Acyrthosiphon pisum
Tribolium castaneum
Nasonia vitripennis
Apis mellifera
Atta cephalotes
Bombyx mori
Danaus plexippus
Heliconius melpomene
Culex quinquefasciatus
Aedes aegypti
Anopheles gambiae
Anopheles darlingi
Drosophila virilis
Drosophila grimshawi
Drosophila willistoni
Drosophila pseudoobscura
Drosophila ananassae
Drosophila melanogaster
Drosophila yakuba
Strongylocentrotus purpuratus
Branchiostoma floridae
Ciona intestinalis
Ciona savignyi
Petromyzon marinus
Danio rerio
Gadus morhua
Oreochromis niloticus
Takifugu rubripes
Tetraodon nigroviridis
Gasterosteus aculeatus
Xiphophorus maculatus
Oryzias latipes
Latimeria chalumnae
Xenopus (Silurana) tropicalis
Pelodiscus sinensis
Anolis carolinensis
Taeniopygia guttata
Gallus gallus
Meleagris gallopavo
Ornithorhynchus anatinus
Monodelphis domestica
Sarcophilus harrisii
Macropus eugenii
Dasypus novemcinctus
Loxodonta africana
Echinops telfairi
Procavia capensis
Myotis lucifugus
Pteropus vampyrus
Sus scrofa
Tursiops truncatus
Bos taurus
Equus caballus
Felis catus
Canis lupus familiaris
Mustela putorius furo
Ailuropoda melanoleuca
Oryctolagus cuniculus
Cavia porcellus
Ictidomys tridecemlineatus
Dipodomys ordii
Rattus norvegicus
Mus musculus
Tupaia belangeri
Otolemur garnettii
Microcebus murinus
Tarsius syrichta
Callithrix jacchus
Macaca mulatta
Nomascus leucogenys
Pongo abelii
Gorilla gorilla
Pan troglodytes
Homo sapiens
Perkinsus marinus ATCC 50983
Tetrahymena thermophila
Paramecium tetraurelia strain d4 2
Neospora caninum Liverpool
Toxoplasma gondii
Cryptosporidium muris RN66
Cryptosporidium parvum Iowa II
Cryptosporidium hominis TU502
Theileria annulata strain Ankara
Theileria parva strain Muguga
Plasmodium falciparum
Plasmodium knowlesi
Plasmodium vivax
Plasmodium chabaudi
Plasmodium yoelii yoelii 17XNL
Plasmodium berghei
Phaeodactylum tricornutum
Thalassiosira pseudonana
Pythium ultimum
Albugo laibachii
Hyaloperonospora arabidopsidis
Phytophthora ramorum
Phytophthora infestans
Phytophthora sojae
Trypanosoma brucei
Trypanosoma cruzi strain CL Brener
Leishmania braziliensis MHOM BR 75 M2904
Leishmania mexicana MHOM GT 2001 U1103
Leishmania major
Leishmania donovani
Leishmania infantum JPCM5
Entamoeba histolytica
Entamoeba dispar SAW760
Dictyostelium discoideum
Dictyostelium purpureum
Trichomonas vaginalis G3
Trichomonas vaginalis
Naegleria gruberi
Giardia lamblia ATCC 50803
OG presence
OG absence
Inferred root
Dscore = 0.894
Pvalue = 1.12e−05
AdjPvalue = 1.34e−03

COG0753

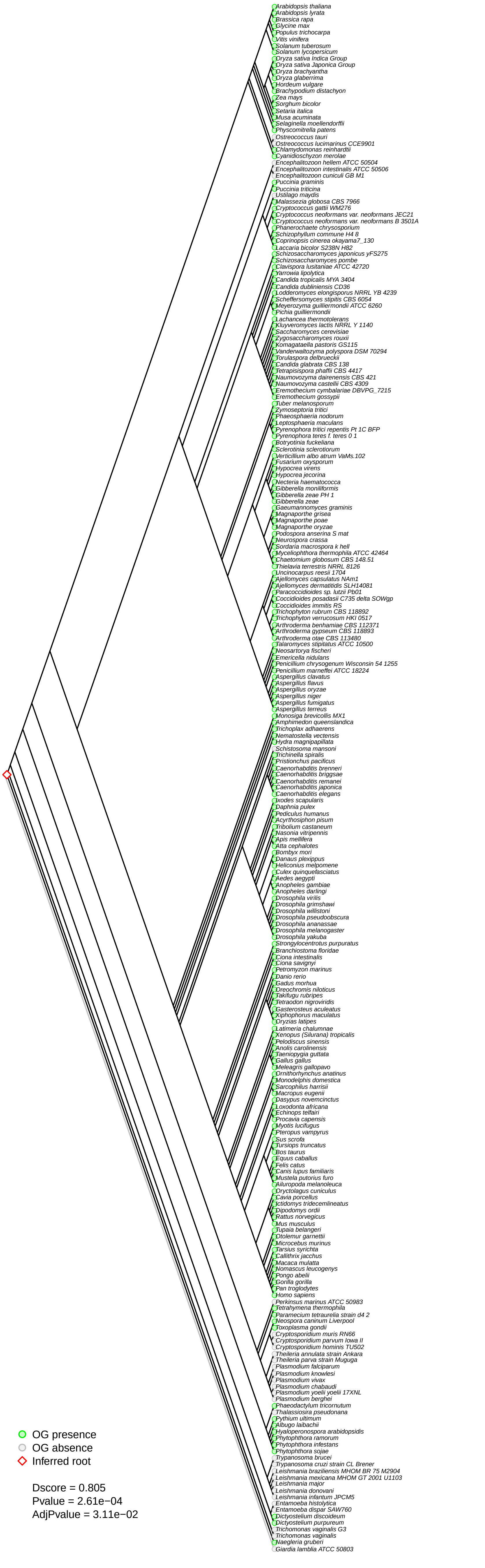

Arabidopsis thaliana
Arabidopsis lyrata
Brassica rapa
Glycine max
Populus trichocarpa
Vitis vinifera
Solanum tuberosum
Solanum lycopersicum
Oryza sativa Indica Group
Oryza sativa Japonica Group
Oryza brachyantha
Oryza glaberrima
Hordeum vulgare
Brachypodium distachyon
Zea mays
Sorghum bicolor
Setaria italica
Musa acuminata
Selaginella moellendorffii
Physcomitrella patens
Ostreococcus tauri
Ostreococcus lucimarinus CCE9901
Chlamydomonas reinhardtii
Cyanidioschyzon merolae
Encephalitozoon hellem ATCC 50504
Encephalitozoon intestinalis ATCC 50506
Encephalitozoon cuniculi GB M1
Puccinia graminis
Puccinia triticina
Ustilago maydis
Malassezia globosa CBS 7966
Cryptococcus gattii WM276
Cryptococcus neoformans var. neoformans JEC21
Cryptococcus neoformans var. neoformans B 3501A
Phanerochaete chrysosporium
Schizophyllum commune H4 8
Coprinopsis cinerea okayama7_130
Laccaria bicolor S238N H82
Schizosaccharomyces japonicus yFS275
Schizosaccharomyces pombe
Clavispora lusitaniae ATCC 42720
Yarrowia lipolytica
Candida tropicalis MYA 3404
Candida dubliniensis CD36
Lodderomyces elongisporus NRRL YB 4239
Scheffersomyces stipitis CBS 6054
Meyerozyma guilliermondii ATCC 6260
Pichia guilliermondii
Lachancea thermotolerans
Kluyveromyces lactis NRRL Y 1140
Saccharomyces cerevisiae
Zygosaccharomyces rouxii
Komagataella pastoris GS115
Vanderwaltozyma polyspora DSM 70294
Torulaspora delbrueckii
Candida glabrata CBS 138
Tetrapisispora phaffii CBS 4417
Naumovozyma dairenensis CBS 421
Naumovozyma castellii CBS 4309
Eremothecium cymbalariae DBVPG_7215
Eremothecium gossypii
Tuber melanosporum
Zymoseptoria tritici
Phaeosphaeria nodorum
Leptosphaeria maculans
Pyrenophora tritici repentis Pt 1C BFP
Pyrenophora teres f. teres 0 1
Botryotinia fuckeliana
Sclerotinia sclerotiorum
Verticillium albo atrum VaMs.102
Fusarium oxysporum
Hypocrea virens
Hypocrea jecorina
Nectria haematococca
Gibberella moniliformis
Gibberella zeae PH 1
Gibberella zeae
Gaeumannomyces graminis
Magnaporthe grisea
Magnaporthe poae
Magnaporthe oryzae
Podospora anserina S mat
Neurospora crassa
Sordaria macrospora k hell
Myceliophthora thermophila ATCC 42464
Chaetomium globosum CBS 148.51
Thielavia terrestris NRRL 8126
Uncinocarpus reesii 1704
Ajellomyces capsulatus NAm1
Ajellomyces dermatitidis SLH14081
Paracoccidioides sp. lutzii Pb01
Coccidioides posadasii C735 delta SOWgp
Coccidioides immitis RS
Trichophyton rubrum CBS 118892
Trichophyton verrucosum HKI 0517
Arthroderma benhamiae CBS 112371
Arthroderma gypseum CBS 118893
Arthroderma otae CBS 113480
Talaromyces stipitatus ATCC 10500
Neosartorya fischeri
Emericella nidulans
Penicillium chrysogenum Wisconsin 54 1255
Penicillium marneffei ATCC 18224
Aspergillus clavatus
Aspergillus flavus
Aspergillus oryzae
Aspergillus niger
Aspergillus fumigatus
Aspergillus terreus
Monosiga brevicollis MX1
Amphimedon queenslandica
Trichoplax adhaerens
Nematostella vectensis
Hydra magnipapillata
Schistosoma mansoni
Trichinella spiralis
Pristionchus pacificus
Caenorhabditis brenneri
Caenorhabditis briggsae
Caenorhabditis remanei
Caenorhabditis japonica
Caenorhabditis elegans
Ixodes scapularis
Daphnia pulex
Pediculus humanus
Acyrthosiphon pisum
Tribolium castaneum
Nasonia vitripennis
Apis mellifera
Atta cephalotes
Bombyx mori
Danaus plexippus
Heliconius melpomene
Culex quinquefasciatus
Aedes aegypti
Anopheles gambiae
Anopheles darlingi
Drosophila virilis
Drosophila grimshawi
Drosophila willistoni
Drosophila pseudoobscura
Drosophila ananassae
Drosophila melanogaster
Drosophila yakuba
Strongylocentrotus purpuratus
Branchiostoma floridae
Ciona intestinalis
Ciona savignyi
Petromyzon marinus
Danio rerio
Gadus morhua
Oreochromis niloticus
Takifugu rubripes
Tetraodon nigroviridis
Gasterosteus aculeatus
Xiphophorus maculatus
Oryzias latipes
Latimeria chalumnae
Xenopus (Silurana) tropicalis
Pelodiscus sinensis
Anolis carolinensis
Taeniopygia guttata
Gallus gallus
Meleagris gallopavo
Ornithorhynchus anatinus
Monodelphis domestica
Sarcophilus harrisii
Macropus eugenii
Dasypus novemcinctus
Loxodonta africana
Echinops telfairi
Procavia capensis
Myotis lucifugus
Pteropus vampyrus
Sus scrofa
Tursiops truncatus
Bos taurus
Equus caballus
Felis catus
Canis lupus familiaris
Mustela putorius furo
Ailuropoda melanoleuca
Oryctolagus cuniculus
Cavia porcellus
Ictidomys tridecemlineatus
Dipodomys ordii
Rattus norvegicus
Mus musculus
Tupaia belangeri
Otolemur garnettii
Microcebus murinus
Tarsius syrichta
Callithrix jacchus
Macaca mulatta
Nomascus leucogenys
Pongo abelii
Gorilla gorilla
Pan troglodytes
Homo sapiens
Perkinsus marinus ATCC 50983
Tetrahymena thermophila
Paramecium tetraurelia strain d4 2
Neospora caninum Liverpool
Toxoplasma gondii
Cryptosporidium muris RN66
Cryptosporidium parvum Iowa II
Cryptosporidium hominis TU502
Theileria annulata strain Ankara
Theileria parva strain Muguga
Plasmodium falciparum
Plasmodium knowlesi
Plasmodium vivax
Plasmodium chabaudi
Plasmodium yoelii yoelii 17XNL
Plasmodium berghei
Phaeodactylum tricornutum
Thalassiosira pseudonana
Pythium ultimum
Albugo laibachii
Hyaloperonospora arabidopsidis
Phytophthora ramorum
Phytophthora infestans
Phytophthora sojae
Trypanosoma brucei
Trypanosoma cruzi strain CL Brener
Leishmania braziliensis MHOM BR 75 M2904
Leishmania mexicana MHOM GT 2001 U1103
Leishmania major
Leishmania donovani
Leishmania infantum JPCM5
Entamoeba histolytica
Entamoeba dispar SAW760
Dictyostelium discoideum
Dictyostelium purpureum
Trichomonas vaginalis G3
Trichomonas vaginalis
Naegleria gruberi
Giardia lamblia ATCC 50803
OG presence
OG absence
Inferred root
Dscore = 0.805
Pvalue = 2.61e−04
AdjPvalue = 3.11e−02

COG1225

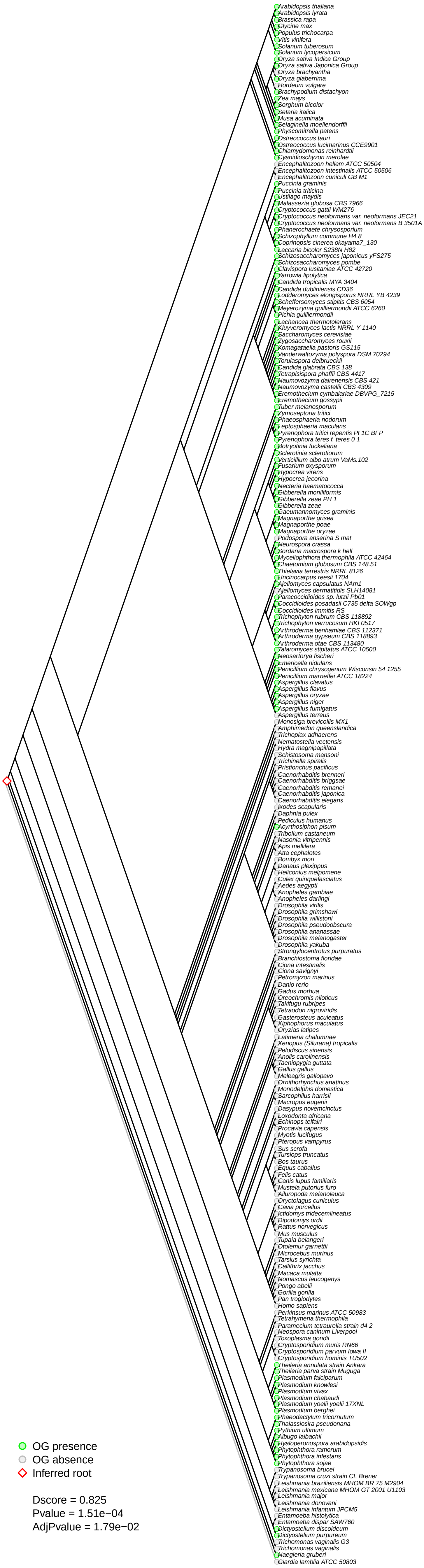

Arabidopsis thaliana
Arabidopsis lyrata
Brassica rapa
Glycine max
Populus trichocarpa
Vitis vinifera
Solanum tuberosum
Solanum lycopersicum
Oryza sativa Indica Group
Oryza sativa Japonica Group
Oryza brachyantha
Oryza glaberrima
Hordeum vulgare
Brachypodium distachyon
Zea mays
Sorghum bicolor
Setaria italica
Musa acuminata
Selaginella moellendorffii
Physcomitrella patens
Ostreococcus tauri
Ostreococcus lucimarinus CCE9901
Chlamydomonas reinhardtii
Cyanidioschyzon merolae
Encephalitozoon hellem ATCC 50504
Encephalitozoon intestinalis ATCC 50506
Encephalitozoon cuniculi GB M1
Puccinia graminis
Puccinia triticina
Ustilago maydis
Malassezia globosa CBS 7966
Cryptococcus gattii WM276
Cryptococcus neoformans var. neoformans JEC21
Cryptococcus neoformans var. neoformans B 3501A
Phanerochaete chrysosporium
Schizophyllum commune H4 8
Coprinopsis cinerea okayama7_130
Laccaria bicolor S238N H82
Schizosaccharomyces japonicus yFS275
Schizosaccharomyces pombe
Clavispora lusitaniae ATCC 42720
Yarrowia lipolytica
Candida tropicalis MYA 3404
Candida dubliniensis CD36
Lodderomyces elongisporus NRRL YB 4239
Scheffersomyces stipitis CBS 6054
Meyerozyma guilliermondii ATCC 6260
Pichia guilliermondii
Lachancea thermotolerans
Kluyveromyces lactis NRRL Y 1140
Saccharomyces cerevisiae
Zygosaccharomyces rouxii
Komagataella pastoris GS115
Vanderwaltozyma polyspora DSM 70294
Torulaspora delbrueckii
Candida glabrata CBS 138
Tetrapisispora phaffii CBS 4417
Naumovozyma dairenensis CBS 421
Naumovozyma castellii CBS 4309
Eremothecium cymbalariae DBVPG_7215
Eremothecium gossypii
Tuber melanosporum
Zymoseptoria tritici
Phaeosphaeria nodorum
Leptosphaeria maculans
Pyrenophora tritici repentis Pt 1C BFP
Pyrenophora teres f. teres 0 1
Botryotinia fuckeliana
Sclerotinia sclerotiorum
Verticillium albo atrum VaMs.102
Fusarium oxysporum
Hypocrea virens
Hypocrea jecorina
Nectria haematococca
Gibberella moniliformis
Gibberella zeae PH 1
Gibberella zeae
Gaeumannomyces graminis
Magnaporthe grisea
Magnaporthe poae
Magnaporthe oryzae
Podospora anserina S mat
Neurospora crassa
Sordaria macrospora k hell
Myceliophthora thermophila ATCC 42464
Chaetomium globosum CBS 148.51
Thielavia terrestris NRRL 8126
Uncinocarpus reesii 1704
Ajellomyces capsulatus NAm1
Ajellomyces dermatitidis SLH14081
Paracoccidioides sp. lutzii Pb01
Coccidioides posadasii C735 delta SOWgp
Coccidioides immitis RS
Trichophyton rubrum CBS 118892
Trichophyton verrucosum HKI 0517
Arthroderma benhamiae CBS 112371
Arthroderma gypseum CBS 118893
Arthroderma otae CBS 113480
Talaromyces stipitatus ATCC 10500
Neosartorya fischeri
Emericella nidulans
Penicillium chrysogenum Wisconsin 54 1255
Penicillium marneffei ATCC 18224
Aspergillus clavatus
Aspergillus flavus
Aspergillus oryzae
Aspergillus niger
Aspergillus fumigatus
Aspergillus terreus
Monosiga brevicollis MX1
Amphimedon queenslandica
Trichoplax adhaerens
Nematostella vectensis
Hydra magnipapillata
Schistosoma mansoni
Trichinella spiralis
Pristionchus pacificus
Caenorhabditis brenneri
Caenorhabditis briggsae
Caenorhabditis remanei
Caenorhabditis japonica
Caenorhabditis elegans
Ixodes scapularis
Daphnia pulex
Pediculus humanus
Acyrthosiphon pisum
Tribolium castaneum
Nasonia vitripennis
Apis mellifera
Atta cephalotes
Bombyx mori
Danaus plexippus
Heliconius melpomene
Culex quinquefasciatus
Aedes aegypti
Anopheles gambiae
Anopheles darlingi
Drosophila virilis
Drosophila grimshawi
Drosophila willistoni
Drosophila pseudoobscura
Drosophila ananassae
Drosophila melanogaster
Drosophila yakuba
Strongylocentrotus purpuratus
Branchiostoma floridae
Ciona intestinalis
Ciona savignyi
Petromyzon marinus
Danio rerio
Gadus morhua
Oreochromis niloticus
Takifugu rubripes
Tetraodon nigroviridis
Gasterosteus aculeatus
Xiphophorus maculatus
Oryzias latipes
Latimeria chalumnae
Xenopus (Silurana) tropicalis
Pelodiscus sinensis
Anolis carolinensis
Taeniopygia guttata
Gallus gallus
Meleagris gallopavo
Ornithorhynchus anatinus
Monodelphis domestica
Sarcophilus harrisii
Macropus eugenii
Dasypus novemcinctus
Loxodonta africana
Echinops telfairi
Procavia capensis
Myotis lucifugus
Pteropus vampyrus
Sus scrofa
Tursiops truncatus
Bos taurus
Equus caballus
Felis catus
Canis lupus familiaris
Mustela putorius furo
Ailuropoda melanoleuca
Oryctolagus cuniculus
Cavia porcellus
Ictidomys tridecemlineatus
Dipodomys ordii
Rattus norvegicus
Mus musculus
Tupaia belangeri
Otolemur garnettii
Microcebus murinus
Tarsius syrichta
Callithrix jacchus
Macaca mulatta
Nomascus leucogenys
Pongo abelii
Gorilla gorilla
Pan troglodytes
Homo sapiens
Perkinsus marinus ATCC 50983
Tetrahymena thermophila
Paramecium tetraurelia strain d4 2
Neospora caninum Liverpool
Toxoplasma gondii
Cryptosporidium muris RN66
Cryptosporidium parvum Iowa II
Cryptosporidium hominis TU502
Theileria annulata strain Ankara
Theileria parva strain Muguga
Plasmodium falciparum
Plasmodium knowlesi
Plasmodium vivax
Plasmodium chabaudi
Plasmodium yoelii yoelii 17XNL
Plasmodium berghei
Phaeodactylum tricornutum
Thalassiosira pseudonana
Pythium ultimum
Albugo laibachii
Hyaloperonospora arabidopsidis
Phytophthora ramorum
Phytophthora infestans
Phytophthora sojae
Trypanosoma brucei
Trypanosoma cruzi strain CL Brener
Leishmania braziliensis MHOM BR 75 M2904
Leishmania mexicana MHOM GT 2001 U1103
Leishmania major
Leishmania donovani
Leishmania infantum JPCM5
Entamoeba histolytica
Entamoeba dispar SAW760
Dictyostelium discoideum
Dictyostelium purpureum
Trichomonas vaginalis G3
Trichomonas vaginalis
Naegleria gruberi
Giardia lamblia ATCC 50803
OG presence
OG absence
Inferred root
Dscore = 0.825
Pvalue = 1.51e−04
AdjPvalue = 1.79e−02

COG1249

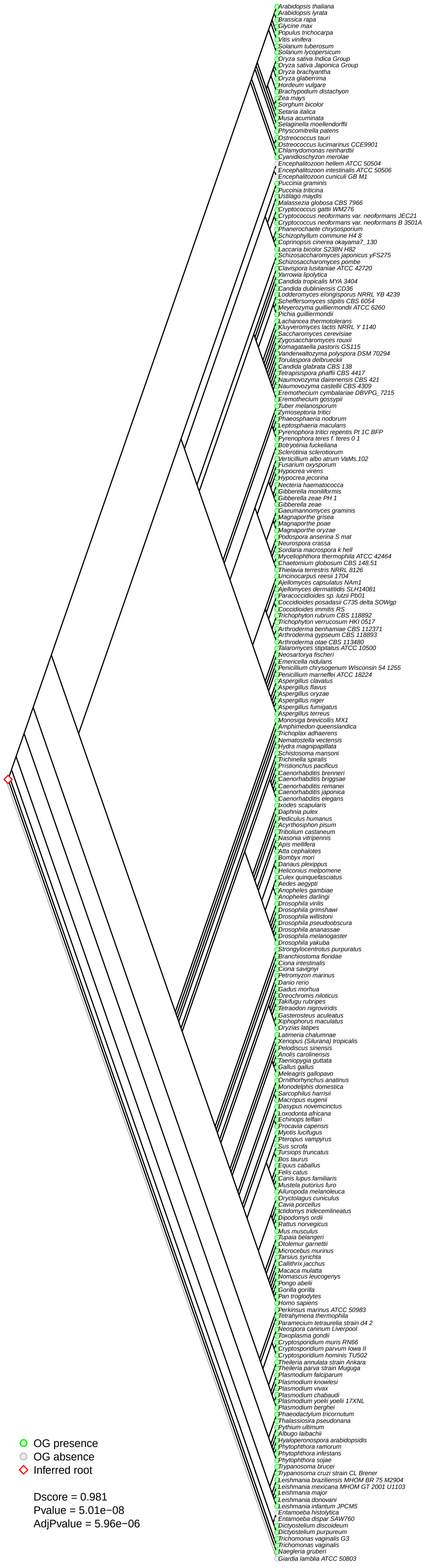

Arabidopsis thaliana
Arabidopsis lyrata
Brassica rapa
Glycine max
Populus trichocarpa
Vitis vinifera
Solanum tuberosum
Solanum lycopersicum
Oryza sativa Indica Group
Oryza sativa Japonica Group
Oryza brachyantha
Oryza glaberrima
Hordeum vulgare
Brachypodium distachyon
Zea mays
Sorghum bicolor
Setaria italica
Musa acuminata
Selaginella moellendorffii
Physcomitrella patens
Ostreococcus tauri
Ostreococcus lucimarinus CCE9901
Chlamydomonas reinhardtii
Cyanidioschyzon merolae
Encephalitozoon hellem ATCC 50504
Encephalitozoon intestinalis ATCC 50506
Encephalitozoon cuniculi GB M1
Puccinia graminis
Puccinia triticina
Ustilago maydis
Malassezia globosa CBS 7966
Cryptococcus gattii WM276
Cryptococcus neoformans var. neoformans JEC21
Cryptococcus neoformans var. neoformans B 3501A
Phanerochaete chrysosporium
Schizophyllum commune H4 8
Coprinopsis cinerea okayama7_130
Laccaria bicolor S238N H82
Schizosaccharomyces japonicus yFS275
Schizosaccharomyces pombe
Clavispora lusitaniae ATCC 42720
Yarrowia lipolytica
Candida tropicalis MYA 3404
Candida dubliniensis CD36
Lodderomyces elongisporus NRRL YB 4239
Scheffersomyces stipitis CBS 6054
Meyerozyma guilliermondii ATCC 6260
Pichia guilliermondii
Lachancea thermotolerans
Kluyveromyces lactis NRRL Y 1140
Saccharomyces cerevisiae
Zygosaccharomyces rouxii
Komagataella pastoris GS115
Vanderwaltozyma polyspora DSM 70294
Torulaspora delbrueckii
Candida glabrata CBS 138
Tetrapisispora phaffii CBS 4417
Naumovozyma dairenensis CBS 421
Naumovozyma castellii CBS 4309
Eremothecium cymbalariae DBVPG_7215
Eremothecium gossypii
Tuber melanosporum
Zymoseptoria tritici
Phaeosphaeria nodorum
Leptosphaeria maculans
Pyrenophora tritici repentis Pt 1C BFP
Pyrenophora teres f. teres 0 1
Botryotinia fuckeliana
Sclerotinia sclerotiorum
Verticillium albo atrum VaMs.102
Fusarium oxysporum
Hypocrea virens
Hypocrea jecorina
Necteria haematococca
Gibberella moniliformis
Gibberella zeae PH 1
Gibberella zeae
Gaeumannomyces graminis
Magnaporthe grisea
Magnaporthe poae
Magnaporthe oryzae
Podospora anserina S mat
Neurospora crassa
Sordaria macrospora k hell
Myceliophthora thermophila ATCC 42464
Chaetomium globosum CBS 148.51
Thielavia terrestris NRRL 8126
Uncinocarpus reesii 1704
Ajellomyces capsulatus NAm1
Ajellomyces dermatitidis SLH14081
Paracoccidioides sp. lutzii Pb01
Coccidioides posadasii C735 delta SOWgp
Coccidioides immitis RS
Trichophyton rubrum CBS 118892
Trichophyton verrucosum HKI 0517
Arthroderma benhamiae CBS 112371
Arthroderma gypseum CBS 118893
Arthroderma otae CBS 113480
Talaromyces stipitatus ATCC 10500
Neosartorya fischeri
Emericella nidulans
Penicillium chrysogenum Wisconsin 54 1255
Penicillium marneffei ATCC 18224
Aspergillus clavatus
Aspergillus flavus
Aspergillus oryzae
Aspergillus niger
Aspergillus fumigatus
Aspergillus terreus
Monosiga brevicollis MX1
Amphimedon queenslandica
Trichoplax adhaerens
Nematostella vectensis
Hydra magnipapillata
Schistosoma mansoni
Trichinella spiralis
Pristionchus pacificus
Caenorhabditis brenneri
Caenorhabditis briggsae
Caenorhabditis remanei
Caenorhabditis japonica
Caenorhabditis elegans
Ixodes scapularis
Daphnia pulex
Pediculus humanus
Acyrthosiphon pisum
Tribolium castaneum
Nasonia vitripennis
Apis mellifera
Atta cephalotes
Bombyx mori
Danaus plexippus
Heliconius melpomene
Culex quinquefasciatus
Aedes aegypti
Anopheles gambiae
Anopheles darlingi
Drosophila virilis
Drosophila grimshawi
Drosophila willistoni
Drosophila pseudoobscura
Drosophila ananassae
Drosophila melanogaster
Drosophila yakuba
Strongylocentrotus purpuratus
Branchiostoma floridae
Ciona intestinalis
Ciona savignyi
Petromyzon marinus
Danio rerio
Gadus morhua
Oreochromis niloticus
Takifugu rubripes
Tetraodon nigroviridis
Gasterosteus aculeatus
Xiphophorus maculatus
Oryzias latipes
Latimeria chalumnae
Xenopus (Silurana) tropicalis
Pelodiscus sinensis
Anolis carolinensis
Taeniopygia guttata
Gallus gallus
Meleagris gallopavo
Ornithorhynchus anatinus
Monodelphis domestica
Sarcophilus harrisii
Macropus eugenii
Dasypus novemcinctus
Loxodonta africana
Echinops telfairi
Procavia capensis
Myotis lucifugus
Pteropus vampyrus
Sus scrofa
Tursiops truncatus
Bos taurus
Equus caballus
Felis catus
Canis lupus familiaris
Mustela putorius furo
Ailuropoda melanoleuca
Oryctolagus cuniculus
Cavia porcellus
Ictidomys tridecemlineatus
Dipodomys ordii
Rattus norvegicus
Mus musculus
Tupaia belangeri
Otolemur garnettii
Microcebus murinus
Tarsius syrichta
Callithrix jacchus
Macaca mulatta
Nomascus leucogenys
Pongo abelii
Gorilla gorilla
Pan troglodytes
Homo sapiens
Perkinsus marinus ATCC 50983
Tetrahymena thermophila
Paramecium tetraurelia strain d4 2
Neospora caninum Liverpool
Toxoplasma gondii
Cryptosporidium muris RN66
Cryptosporidium parvum Iowa II
Cryptosporidium hominis TU502
Theileria annulata strain Ankara
Theileria parva strain Muguga
Plasmodium falciparum
Plasmodium knowlesi
Plasmodium vivax
Plasmodium chabaudi
Plasmodium yoelii yoelii 17XNL
Plasmodium berghei
Phaeodactylum tricornutum
Thalassiosira pseudonana
Pythium ultimum
Albugo laibachii
Hyaloperonospora arabidopsidis
Phytophthora ramorum
Phytophthora infestans
Phytophthora sojae
Trypanosoma brucei
Trypanosoma cruzi strain CL Brener
Leishmania braziliensis MHOM BR 75 M2904
Leishmania mexicana MHOM GT 2001 U1103
Leishmania major
Leishmania donovani
Leishmania infantum JPCM5
Entamoeba histolytica
Entamoeba dispar SAW760
Dictyostelium discoideum
Dictyostelium purpureum
Trichomonas vaginalis G3
Trichomonas vaginalis
Naegleria gruberi
Giardia lamblia ATCC 50803
OG presence
OG absence
Inferred root
Dscore = 0.981
Pvalue = 5.01e−08
AdjPvalue = 5.96e−06

COG1304

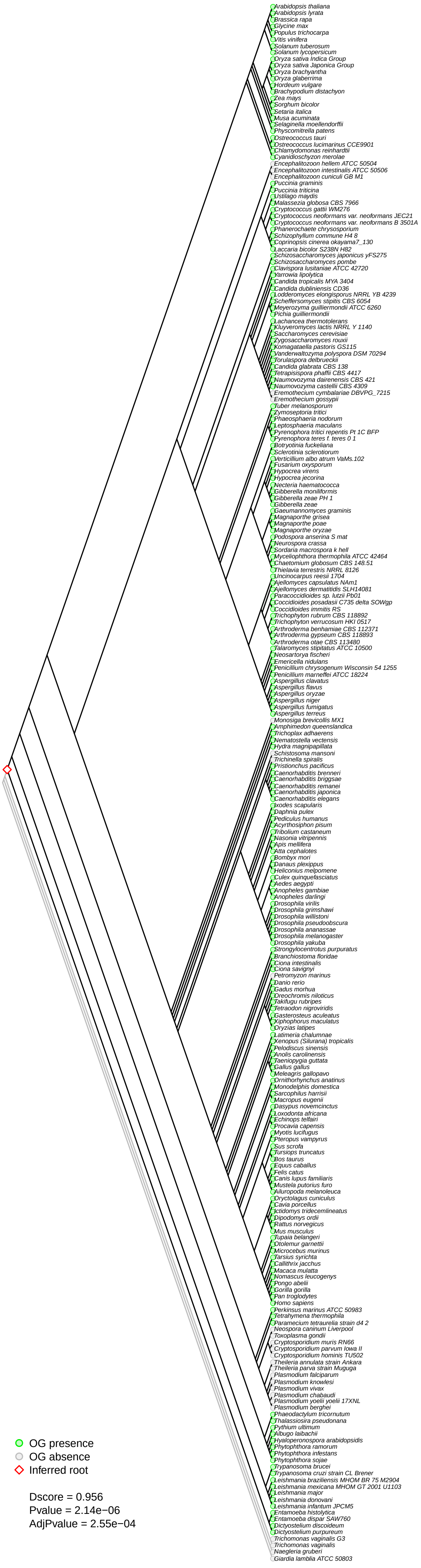

Arabidopsis thaliana
Arabidopsis lyrata
Brassica rapa
Glycine max
Populus trichocarpa
Vitis vinifera
Solanum tuberosum
Solanum lycopersicum
Oryza sativa Indica Group
Oryza sativa Japonica Group
Oryza brachyantha
Oryza glaberrima
Hordeum vulgare
Brachypodium distachyon
Zea mays
Sorghum bicolor
Setaria italica
Musa acuminata
Selaginella moellendorffii
Physcomitrella patens
Ostreococcus tauri
Ostreococcus lucimarinus CCE9901
Chlamydomonas reinhardtii
Cyanidioschyzon merolae
Encephalitozoon hellem ATCC 50504
Encephalitozoon intestinalis ATCC 50506
Encephalitozoon cuniculi GB M1
Puccinia graminis
Puccinia triticina
Ustilago maydis
Malassezia globosa CBS 7966
Cryptococcus gattii WM276
Cryptococcus neoformans var. neoformans JEC21
Cryptococcus neoformans var. neoformans B 3501A
Phanerochaete chrysosporium
Schizophyllum commune H4 8
Coprinopsis cinerea okayama7_130
Laccaria bicolor S238N H82
Schizosaccharomyces japonicus yFS275
Schizosaccharomyces pombe
Clavispora lusitaniae ATCC 42720
Yarrowia lipolytica
Candida tropicalis MYA 3404
Candida dubliniensis CD36
Lodderomyces elongisporus NRRL YB 4239
Scheffersomyces stipitis CBS 6054
Meyerozyma guilliermondii ATCC 6260
Pichia guilliermondii
Lachancea thermotolerans
Kluyveromyces lactis NRRL Y 1140
Saccharomyces cerevisiae
Zygosaccharomyces rouxii
Komagataella pastoris GS115
Vanderwaltozyma polyspora DSM 70294
Torulaspora delbrueckii
Candida glabrata CBS 138
Tetrapisispora phaffii CBS 4417
Naumovozyma dairenensis CBS 421
Naumovozyma castellii CBS 4309
Eremothecium cymbalariae DBVPG_7215
Eremothecium gossypii
Tuber melanosporum
Zymoseptoria tritici
Phaeosphaeria nodorum
Leptosphaeria maculans
Pyrenophora tritici repentis Pt 1C BFP
Pyrenophora teres f. teres 0 1
Botryotinia fuckeliana
Sclerotinia sclerotiorum
Verticillium albo atrum VaMs.102
Fusarium oxysporum
Hypocrea virens
Hypocrea jecorina
Nectria haematococca
Gibberella moniliformis
Gibberella zeae PH 1
Gibberella zeae
Gaeumannomyces graminis
Magnaporthe grisea
Magnaporthe poae
Magnaporthe oryzae
Podospora anserina S mat
Neurospora crassa
Sordaria macrospora k hell
Myceliophthora thermophila ATCC 42464
Chaetomium globosum CBS 148.51
Thielavia terrestris NRRL 8126
Uncinocarpus reesii 1704
Ajellomyces capsulatus NAm1
Ajellomyces dermatitidis SLH14081
Paracoccidioides sp. lutzii Pb01
Coccidioides posadasii C735 delta SOWgp
Coccidioides immitis RS
Trichophyton rubrum CBS 118892
Trichophyton verrucosum HKI 0517
Arthroderma benhamiae CBS 112371
Arthroderma gypseum CBS 118893
Arthroderma otae CBS 113480
Talaromyces stipitatus ATCC 10500
Neosartorya fischeri
Emericella nidulans
Penicillium chrysogenum Wisconsin 54 1255
Penicillium marneffei ATCC 18224
Aspergillus clavatus
Aspergillus flavus
Aspergillus oryzae
Aspergillus niger
Aspergillus fumigatus
Aspergillus terreus
Monosiga brevicollis MX1
Amphimedon queenslandica
Trichoplax adhaerens
Nematostella vectensis
Hydra magnipapillata
Schistosoma mansoni
Trichinella spiralis
Pristionchus pacificus
Caenorhabditis brenneri
Caenorhabditis briggsae
Caenorhabditis remanei
Caenorhabditis japonica
Caenorhabditis elegans
Ixodes scapularis
Daphnia pulex
Pediculus humanus
Acyrthosiphon pisum
Tribolium castaneum
Nasonia vitripennis
Apis mellifera
Atta cephalotes
Bombyx mori
Danaus plexippus
Heliconius melpomene
Culex quinquefasciatus
Aedes aegypti
Anopheles gambiae
Anopheles darlingi
Drosophila virilis
Drosophila grimshawi
Drosophila willistoni
Drosophila pseudoobscura
Drosophila ananassae
Drosophila melanogaster
Drosophila yakuba
Strongylocentrotus purpuratus
Branchiostoma floridae
Ciona intestinalis
Ciona savignyi
Petromyzon marinus
Danio rerio
Gadus morhua
Oreochromis niloticus
Takifugu rubripes
Tetraodon nigroviridis
Gasterosteus aculeatus
Xiphophorus maculatus
Oryzias latipes
Latimeria chalumnae
Xenopus (Silurana) tropicalis
Pelodiscus sinensis
Anolis carolinensis
Taeniopygia guttata
Gallus gallus
Meleagris gallopavo
Ornithorhynchus anatinus
Monodelphis domestica
Sarcophilus harrisii
Macropus eugenii
Dasypus novemcinctus
Loxodonta africana
Echinops telfairi
Procavia capensis
Myotis lucifugus
Pteropus vampyrus
Sus scrofa
Tursiops truncatus
Bos taurus
Equus caballus
Felis catus
Canis lupus familiaris
Mustela putorius furo
Ailuropoda melanoleuca
Oryctolagus cuniculus
Cavia porcellus
Ictidomys tridecemlineatus
Dipodomys ordii
Rattus norvegicus
Mus musculus
Tupaia belangeri
Otolemur garnettii
Microcebus murinus
Tarsius syrichta
Callithrix jacchus
Macaca mulatta
Nomascus leucogenys
Pongo abelii
Gorilla gorilla
Pan troglodytes
Homo sapiens
Perkinsus marinus ATCC 50983
Tetrahymena thermophila
Paramecium tetraurelia strain d4 2
Neospora caninum Liverpool
Toxoplasma gondii
Cryptosporidium muris RN66
Cryptosporidium parvum Iowa II
Cryptosporidium hominis TU502
Theileria annulata strain Ankara
Theileria parva strain Muguga
Plasmodium falciparum
Plasmodium knowlesi
Plasmodium vivax
Plasmodium chabaudi
Plasmodium yoelii yoelii 17XNL
Plasmodium berghei
Phaeodactylum tricornutum
Thalassiosira pseudonana
Pythium ultimum
Albugo laibachii
Hyaloperonospora arabidopsidis
Phytophthora ramorum
Phytophthora infestans
Phytophthora sojae
Trypanosoma brucei
Trypanosoma cruzi strain CL Brener
Leishmania braziliensis MHOM BR 75 M2904
Leishmania mexicana MHOM GT 2001 U1103
Leishmania major
Leishmania donovani
Leishmania infantum JPCM5
Entamoeba histolytica
Entamoeba dispar SAW760
Dictyostelium discoideum
Dictyostelium purpureum
Trichomonas vaginalis G3
Trichomonas vaginalis
Naegleria gruberi
Giardia lamblia ATCC 50803
OG presence
OG absence
Inferred root
Dscore = 0.956
Pvalue = 2.14e−06
AdjPvalue = 2.55e−04

COG1324

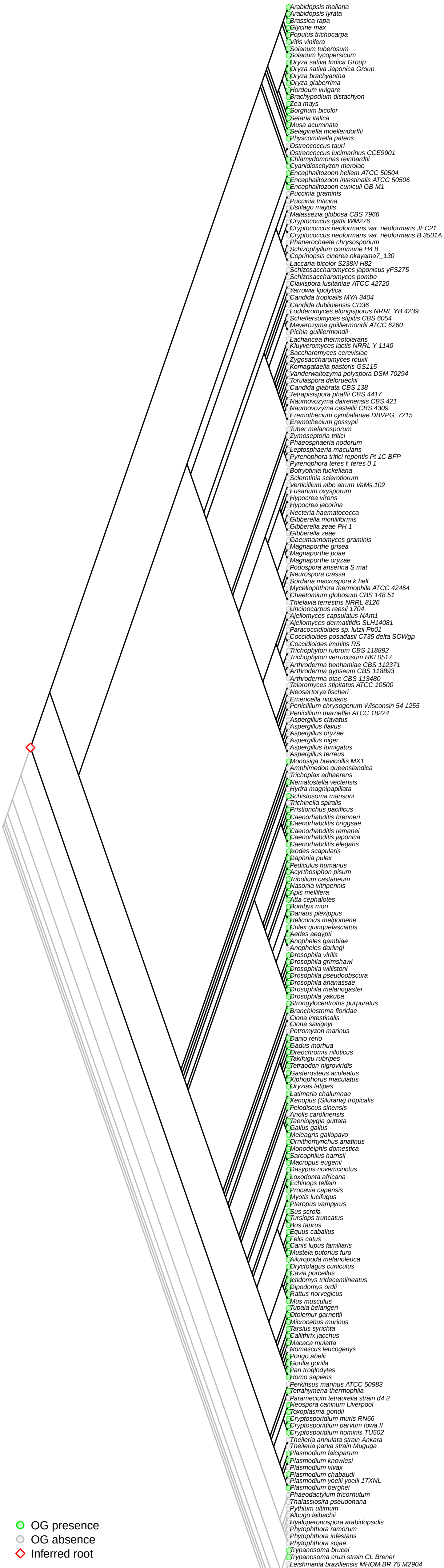

Dscore = 0.71
Pvalue = 4.58e−04
AdjPvalue = 5.46e−02

COG1528

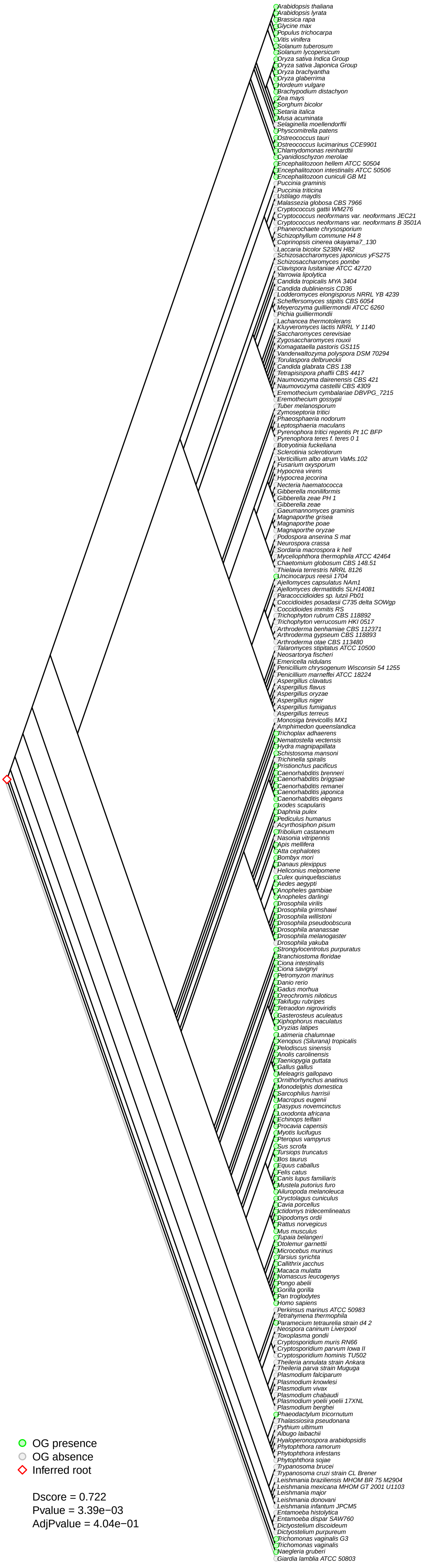
Arabidopsis thaliana
Arabidopsis lyrata
Brassica rapa
Glycine max
Populus trichocarpa
Vitis vinifera
Solanum tuberosum
Solanum lycopersicum
Oryza sativa Indica Group
Oryza sativa Japonica Group
Oryza brachyantha
Oryza glaberrima
Hordeum vulgare
Brachypodium distachyon
Zea mays
Sorghum bicolor
Setaria italica
Musa acuminata
Selaginella moellendorffii
Physcomitrella patens
Ostreococcus tauri
Ostreococcus lucimarinus CCE9901
Chlamydomonas reinhardtii
Cyanidioschyzon merolae
Encephalitozoon hellem ATCC 50504
Encephalitozoon intestinalis ATCC 50506
Encephalitozoon cuniculi GB M1
Puccinia graminis
Puccinia triticina
Ustilago maydis
Malassezia globosa CBS 7966
Cryptococcus gattii WM276
Cryptococcus neoformans var. neoformans JEC21
Cryptococcus neoformans var. neoformans B 3501A
Phanerochaete chrysosporium
Schizophyllum commune H4 8
Coprinopsis cinerea okayama7_130
Laccaria bicolor S238N H82
Schizosaccharomyces japonicus yFS275
Schizosaccharomyces pombe
Clavispora lusitaniae ATCC 42720
Yarrowia lipolytica
Candida tropicalis MYA 3404
Candida dubliniensis CD36
Lodderomyces elongisporus NRRL YB 4239
Scheffersomyces stipitis CBS 6054
Meyerozyma guilliermondii ATCC 6260
Pichia guilliermondii
Lachancea thermotolerans
Kluyveromyces lactis NRRL Y 1140
Saccharomyces cerevisiae
Zygosaccharomyces rouxii
Komagataella pastoris GS115
Vanderwaltozyma polyspora DSM 70294
Torulaspora delbrueckii
Candida glabrata CBS 138
Tetrapisispora phaffii CBS 4417
Naumovozyma dairenensis CBS 421
Naumovozyma castellii CBS 4309
Eremothecium cymbalariae DBVPG_7215
Eremothecium gossypii
Tuber melanosporum
Zymoseptoria tritici
Phaeosphaeria nodorum
Leptosphaeria maculans
Pyrenophora tritici repentis Pt 1C BFP
Pyrenophora teres f. teres 0 1
Botryotinia fuckeliana
Sclerotinia sclerotiorum
Verticillium albo atrum VaMs.102
Fusarium oxysporum
Hypocrea virens
Hypocrea jecorina
Nectria haematococca
Gibberella moniliformis
Gibberella zeae PH 1
Gibberella zeae
Gaeumannomyces graminis
Magnaporthe grisea
Magnaporthe poae
Magnaporthe oryzae
Podospora anserina S mat
Neurospora crassa
Sordaria macrospora k hell
Myceliophthora thermophila ATCC 42464
Chaetomium globosum CBS 148.51
Thielavia terrestris NRRL 8126
Uncinocarpus reesii 1704
Ajellomyces capsulatus NAm1
Ajellomyces dermatitidis SLH14081
Paracoccidioides sp. lutzii Pb01
Coccidioides posadasii C735 delta SOWgp
Coccidioides immitis RS
Trichophyton rubrum CBS 118892
Trichophyton verrucosum HKI 0517
Arthroderma benhamiae CBS 112371
Arthroderma gypseum CBS 118893
Arthroderma otae CBS 113480
Talaromyces stipitatus ATCC 10500
Neosartorya fischeri
Emericella nidulans
Penicillium chrysogenum Wisconsin 54 1255
Penicillium marneffei ATCC 18224
Aspergillus clavatus
Aspergillus flavus
Aspergillus oryzae
Aspergillus niger
Aspergillus fumigatus
Aspergillus terreus
Monosiga brevicollis MX1
Amphimedon queenslandica
Trichoplax adhaerens
Nematostella vectensis
Hydra magnipapillata
Schistosoma mansoni
Trichinella spiralis
Pristionchus pacificus
Caenorhabditis brenneri
Caenorhabditis briggsae
Caenorhabditis remanei
Caenorhabditis japonica
Caenorhabditis elegans
Ixodes scapularis
Daphnia pulex
Pediculus humanus
Acyrthosiphon pisum
Tribolium castaneum
Nasonia vitripennis
Apis mellifera
Atta cephalotes
Bombyx mori
Danaus plexippus
Heliconius melpomene
Culex quinquefasciatus
Aedes aegypti
Anopheles gambiae
Anopheles darlingi
Drosophila virilis
Drosophila grimshawi
Drosophila willistoni
Drosophila pseudoobscura
Drosophila ananassae
Drosophila melanogaster
Drosophila yakuba
Strongylocentrotus purpuratus
Branchiostoma floridae
Ciona intestinalis
Ciona savignyi
Petromyzon marinus
Danio rerio
Gadus morhua
Oreochromis niloticus
Takifugu rubripes
Tetraodon nigroviridis
Gasterosteus aculeatus
Xiphophorus maculatus
Oryzias latipes
Latimeria chalumnae
Xenopus (Silurana) tropicalis
Pelodiscus sinensis
Anolis carolinensis
Taeniopygia guttata
Gallus gallus
Meleagris gallopavo
Ornithorhynchus anatinus
Monodelphis domestica
Sarcophilus harrisii
Macropus eugenii
Dasypus novemcinctus
Loxodonta africana
Echinops telfairi
Procavia capensis
Myotis lucifugus
Pteropus vampyrus
Sus scrofa
Tursiops truncatus
Bos taurus
Equus caballus
Felis catus
Canis lupus familiaris
Mustela putorius furo
Ailuropoda melanoleuca
Oryctolagus cuniculus
Cavia porcellus
Ictidomys tridecemlineatus
Dipodomys ordii
Rattus norvegicus
Mus musculus
Tupaia belangeri
Otolemur garnettii
Microcebus murinus
Tarsius syrichta
Callithrix jacchus
Macaca mulatta
Nomascus leucogenys
Pongo abelii
Gorilla gorilla
Pan troglodytes
Homo sapiens
Perkinsus marinus ATCC 50983
Tetrahymena thermophila
Paramecium tetraurelia strain d4 2
Neospora caninum Liverpool
Toxoplasma gondii
Cryptosporidium muris RN66
Cryptosporidium parvum Iowa II
Cryptosporidium hominis TU502
Theileria annulata strain Ankara
Theileria parva strain Muguga
Plasmodium falciparum
Plasmodium knowlesi
Plasmodium vivax
Plasmodium chabaudi
Plasmodium yoelii yoelii 17XNL
Plasmodium berghei
Phaeodactylum tricornutum
Thalassiosira pseudonana
Pythium ultimum
Albugo laibachii
Hyaloperonospora arabidopsidis
Phytophthora ramorum
Phytophthora infestans
Phytophthora sojae
Trypanosoma brucei
Trypanosoma cruzi strain CL Brener
Leishmania braziliensis MHOM BR 75 M2904
Leishmania mexicana MHOM GT 2001 U1103
Leishmania major
Leishmania donovani
Leishmania infantum JPCM5
Entamoeba histolytica
Entamoeba dispar SAW760
Dictyostelium discoideum
Dictyostelium purpureum
Trichomonas vaginalis G3
Trichomonas vaginalis
Naegleria gruberi
Giardia lamblia ATCC 50803
OG presence
OG absence
Inferred root
Dscore = 0.722
Pvalue = 3.39e−03
AdjPvalue = 4.04e−01

COG2032

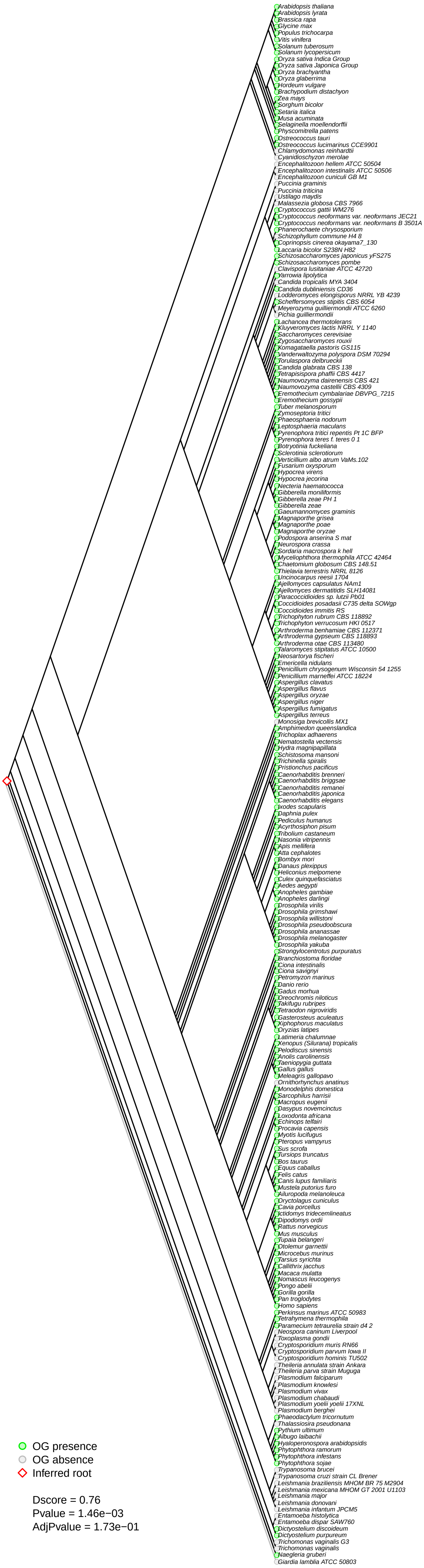
Arabidopsis thaliana
Arabidopsis lyrata
Brassica rapa
Glycine max
Populus trichocarpa
Vitis vinifera
Solanum tuberosum
Solanum lycopersicum
Oryza sativa Indica Group
Oryza sativa Japonica Group
Oryza brachyantha
Oryza glaberrima
Hordeum vulgare
Brachypodium distachyon
Zea mays
Sorghum bicolor
Setaria italica
Musa acuminata
Selaginella moellendorffii
Physcomitrella patens
Ostreococcus tauri
Ostreococcus lucimarinus CCE9901
Chlamydomonas reinhardtii
Cyanidioschyzon merolae
Encephalitozoon hellem ATCC 50504
Encephalitozoon intestinalis ATCC 50506
Encephalitozoon cuniculi GB M1
Puccinia graminis
Puccinia triticina
Ustilago maydis
Malassezia globosa CBS 7966
Cryptococcus gattii WM276
Cryptococcus neoformans var. neoformans JEC21
Cryptococcus neoformans var. neoformans B 3501A
Phanerochaete chrysosporium
Schizophyllum commune H4 8
Coprinopsis cinerea okayama7_130
Laccaria bicolor S238N H82
Schizosaccharomyces japonicus yFS275
Schizosaccharomyces pombe
Clavispora lusitaniae ATCC 42720
Yarrowia lipolytica
Candida tropicalis MYA 3404
Candida dubliniensis CD36
Lodderomyces elongisporus NRRL YB 4239
Scheffersomyces stipitis CBS 6054
Meyerozyma guilliermondii ATCC 6260
Pichia guilliermondii
Lachancea thermotolerans
Kluyveromyces lactis NRRL Y 1140
Saccharomyces cerevisiae
Zygosaccharomyces rouxii
Komagataella pastoris GS115
Vanderwaltozyma polyspora DSM 70294
Torulaspora delbrueckii
Candida glabrata CBS 138
Tetrapisispora phaffii CBS 4417
Naumovozyma dairenensis CBS 421
Naumovozyma castellii CBS 4309
Eremothecium cymbalariae DBVPG_7215
Eremothecium gossypii
Tuber melanosporum
Zymoseptoria tritici
Phaeosphaeria nodorum
Leptosphaeria maculans
Pyrenophora tritici repentis Pt 1C BFP
Pyrenophora teres f. teres 0 1
Botryotinia fuckeliana
Sclerotinia sclerotiorum
Verticillium albo atrum VaMs.102
Fusarium oxysporum
Hypocrea virens
Hypocrea jecorina
Nectria haematococca
Gibberella moniliformis
Gibberella zeae PH 1
Gibberella zeae
Gaeumannomyces graminis
Magnaporthe grisea
Magnaporthe poae
Magnaporthe oryzae
Podospora anserina S mat
Neurospora crassa
Sordaria macrospora k hell
Myceliophthora thermophila ATCC 42464
Chaetomium globosum CBS 148.51
Thielavia terrestris NRRL 8126
Uncinocarpus reesii 1704
Ajellomyces capsulatus NAm1
Ajellomyces dermatitidis SLH14081
Paracoccidioides sp. lutzii Pb01
Coccidioides posadasii C735 delta SOWgp
Coccidioides immitis RS
Trichophyton rubrum CBS 118892
Trichophyton verrucosum HKI 0517
Arthroderma benhamiae CBS 112371
Arthroderma gypseum CBS 118893
Arthroderma otae CBS 113480
Talaromyces stipitatus ATCC 10500
Neosartorya fischeri
Emericella nidulans
Penicillium chrysogenum Wisconsin 54 1255
Penicillium marneffei ATCC 18224
Aspergillus clavatus
Aspergillus flavus
Aspergillus oryzae
Aspergillus niger
Aspergillus fumigatus
Aspergillus terreus
Monosiga brevicollis MX1
Amphimedon queenslandica
Trichoplax adhaerens
Nematostella vectensis
Hydra magnipapillata
Schistosoma mansoni
Trichinella spiralis
Pristionchus pacificus
Caenorhabditis brenneri
Caenorhabditis briggsae
Caenorhabditis remanei
Caenorhabditis japonica
Caenorhabditis elegans
Ixodes scapularis
Daphnia pulex
Pediculus humanus
Acyrthosiphon pisum
Tribolium castaneum
Nasonia vitripennis
Apis mellifera
Atta cephalotes
Bombyx mori
Danaus plexippus
Heliconius melpomene
Culex quinquefasciatus
Aedes aegypti
Anopheles gambiae
Anopheles darlingi
Drosophila virilis
Drosophila grimshawi
Drosophila willistoni
Drosophila pseudoobscura
Drosophila ananassae
Drosophila melanogaster
Drosophila yakuba
Strongylocentrotus purpuratus
Branchiostoma floridae
Ciona intestinalis
Ciona savignyi
Petromyzon marinus
Danio rerio
Gadus morhua
Oreochromis niloticus
Takifugu rubripes
Tetraodon nigroviridis
Gasterosteus aculeatus
Xiphophorus maculatus
Oryzias latipes
Latimeria chalumnae
Xenopus (Silurana) tropicalis
Pelodiscus sinensis
Anolis carolinensis
Taeniopygia guttata
Gallus gallus
Meleagris gallopavo
Ornithorhynchus anatinus
Monodelphis domestica
Sarcophilus harrisii
Macropus eugenii
Dasypus novemcinctus
Loxodonta africana
Echinops telfairi
Procavia capensis
Myotis lucifugus
Pteropus vampyrus
Sus scrofa
Tursiops truncatus
Bos taurus
Equus caballus
Felis catus
Canis lupus familiaris
Mustela putorius furo
Ailuropoda melanoleuca
Oryctolagus cuniculus
Cavia porcellus
Ictidomys tridecemlineatus
Dipodomys ordii
Rattus norvegicus
Mus musculus
Tupaia belangeri
Otolemur garnettii
Microcebus murinus
Tarsius syrichta
Callithrix jacchus
Macaca mulatta
Nomascus leucogenys
Pongo abelii
Gorilla gorilla
Pan troglodytes
Homo sapiens
Perkinsus marinus ATCC 50983
Tetrahymena thermophila
Paramecium tetraurelia strain d4 2
Neospora caninum Liverpool
Toxoplasma gondii
Cryptosporidium muris RN66
Cryptosporidium parvum Iowa II
Cryptosporidium hominis TU502
Theileria annulata strain Ankara
Theileria parva strain Muguga
Plasmodium falciparum
Plasmodium knowlesi
Plasmodium vivax
Plasmodium chabaudi
Plasmodium yoelii yoelii 17XNL
Plasmodium berghei
Phaeodactylum tricornutum
Thalassiosira pseudonana
Pythium ultimum
Albugo laibachii
Hyaloperonospora arabidopsidis
Phytophthora ramorum
Phytophthora infestans
Phytophthora sojae
Trypanosoma brucei
Trypanosoma cruzi strain CL Brener
Leishmania braziliensis MHOM BR 75 M2904
Leishmania mexicana MHOM GT 2001 U1103
Leishmania major
Leishmania donovani
Leishmania infantum JPCM5
Entamoeba histolytica
Entamoeba dispar SAW760
Dictyostelium discoideum
Dictyostelium purpureum
Trichomonas vaginalis G3
Trichomonas vaginalis
Naegleria gruberi
Giardia lamblia ATCC 50803
OG presence
OG absence
Inferred root
Dscore = 0.76
Pvalue = 1.46e−03
AdjPvalue = 1.73e−01

COG2070

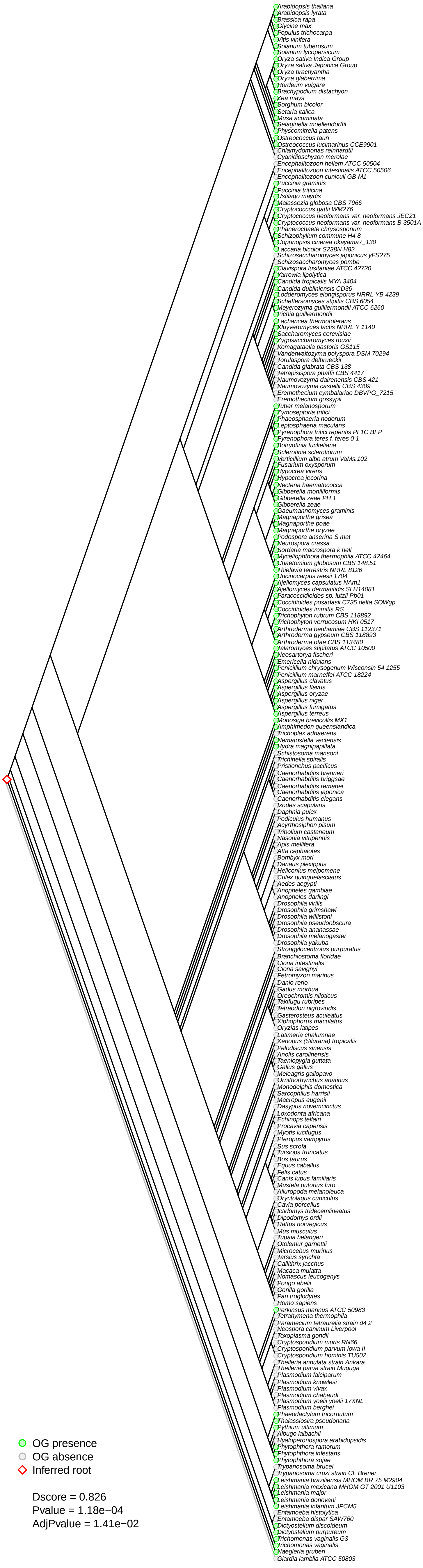

Arabidopsis thaliana
Arabidopsis lyrata
Brassica rapa
Glycine max
Populus trichocarpa
Vitis vinifera
Solanum tuberosum
Solanum lycopersicum
Oryza sativa Indica Group
Oryza sativa Japonica Group
Oryza brachyantha
Oryza glaberrima
Hordeum vulgare
Brachypodium distachyon
Zea mays
Sorghum bicolor
Setaria italica
Musa acuminata
Selaginella moellendorffii
Physcomitrella patens
Ostreococcus tauri
Ostreococcus lucimarinus CCE9901
Chlamydomonas reinhardtii
Cyanidioschyzon merolae
Encephalitozoon hellem ATCC 50504
Encephalitozoon intestinalis ATCC 50506
Encephalitozoon cuniculi GB M1
Puccinia graminis
Puccinia triticina
Ustilago maydis
Malassezia globosa CBS 7966
Cryptococcus gattii WM276
Cryptococcus neoformans var. neoformans JEC21
Cryptococcus neoformans var. neoformans B 3501A
Phanerochaete chrysosporium
Schizophyllum commune H4 8
Coprinopsis cinerea okayama7_130
Laccaria bicolor S238N H82
Schizosaccharomyces japonicus yFS275
Schizosaccharomyces pombe
Clavispora lusitaniae ATCC 42720
Yarrowia lipolytica
Candida tropicalis MYA 3404
Candida dubliniensis CD36
Lodderomyces elongisporus NRRL YB 4239
Scheffersomyces stipitis CBS 6054
Meyerozyma guilliermondii ATCC 6260
Pichia guilliermondii
Lachancea thermotolerans
Kluyveromyces lactis NRRL Y 1140
Saccharomyces cerevisiae
Zygosaccharomyces rouxii
Komagataella pastoris GS115
Vanderwaltozyma polyspora DSM 70294
Torulaspora delbrueckii
Candida glabrata CBS 138
Tetrapisispora phaffii CBS 4417
Naumovozyma dairenensis CBS 421
Naumovozyma castellii CBS 4309
Eremothecium cymbalariae DBVPG_7215
Eremothecium gossypii
Tuber melanosporum
Zymoseptoria tritici
Phaeosphaeria nodorum
Leptosphaeria maculans
Pyrenophora tritici repentis Pt 1C BFP
Pyrenophora teres f. teres 0 1
Botryotinia fuckeliana
Sclerotinia sclerotiorum
Verticillium albo atrum VaMs.102
Fusarium oxysporum
Hypocrea virens
Hypocrea jecorina
Nectria haematococca
Gibberella moniliformis
Gibberella zeae PH 1
Gibberella zeae
Gaeumannomyces graminis
Magnaporthe grisea
Magnaporthe poae
Magnaporthe oryzae
Podospora anserina S mat
Neurospora crassa
Sordaria macrospora k hell
Myceliophthora thermophila ATCC 42464
Chaetomium globosum CBS 148.51
Thielavia terrestris NRRL 8126
Uncinocarpus reesii 1704
Ajellomyces capsulatus NAm1
Ajellomyces dermatitidis SLH14081
Paracoccidioides sp. lutzii Pb01
Coccidioides posadasii C735 delta SOWgp
Coccidioides immitis RS
Trichophyton rubrum CBS 118892
Trichophyton verrucosum HKI 0517
Arthroderma benhamiae CBS 112371
Arthroderma gypseum CBS 118893
Arthroderma otae CBS 113480
Talaromyces stipitatus ATCC 10500
Neosartorya fischeri
Emericella nidulans
Penicillium chrysogenum Wisconsin 54 1255
Penicillium marneffei ATCC 18224
Aspergillus clavatus
Aspergillus flavus
Aspergillus oryzae
Aspergillus niger
Aspergillus fumigatus
Aspergillus terreus
Monosiga brevicollis MX1
Amphimedon queenslandica
Trichoplax adhaerens
Nematostella vectensis
Hydra magnipapillata
Schistosoma mansoni
Trichinella spiralis
Pristionchus pacificus
Caenorhabditis brenneri
Caenorhabditis briggsae
Caenorhabditis remanei
Caenorhabditis japonica
Caenorhabditis elegans
Ixodes scapularis
Daphnia pulex
Pediculus humanus
Acyrthosiphon pisum
Tribolium castaneum
Nasonia vitripennis
Apis mellifera
Atta cephalotes
Bombyx mori
Danaus plexippus
Heliconius melpomene
Culex quinquefasciatus
Aedes aegypti
Anopheles gambiae
Anopheles darlingi
Drosophila virilis
Drosophila grimshawi
Drosophila willistoni
Drosophila pseudoobscura
Drosophila ananassae
Drosophila melanogaster
Drosophila yakuba
Strongylocentrotus purpuratus
Branchiostoma floridae
Ciona intestinalis
Ciona savignyi
Petromyzon marinus
Danio rerio
Gadus morhua
Oreochromis niloticus
Takifugu rubripes
Tetraodon nigroviridis
Gasterosteus aculeatus
Xiphophorus maculatus
Oryzias latipes
Latimeria chalumnae
Xenopus (Silurana) tropicalis
Pelodiscus sinensis
Anolis carolinensis
Taeniopygia guttata
Gallus gallus
Meleagris gallopavo
Ornithorhynchus anatinus
Monodelphis domestica
Sarcophilus harrisii
Macropus eugenii
Dasypus novemcinctus
Loxodonta africana
Echinops telfairi
Procavia capensis
Myotis lucifugus
Pteropus vampyrus
Sus scrofa
Tursiops truncatus
Bos taurus
Equus caballus
Felis catus
Canis lupus familiaris
Mustela putorius furo
Ailuropoda melanoleuca
Oryctolagus cuniculus
Cavia porcellus
Ictidomys tridecemlineatus
Dipodomys ordii
Rattus norvegicus
Mus musculus
Tupaia belangeri
Otolemur garnettii
Microcebus murinus
Tarsius syrichta
Callithrix jacchus
Macaca mulatta
Nomascus leucogenys
Pongo abelii
Gorilla gorilla
Pan troglodytes
Homo sapiens
Perkinsus marinus ATCC 50983
Tetrahymena thermophila
Paramecium tetraurelia strain d4 2
Neospora caninum Liverpool
Toxoplasma gondii
Cryptosporidium muris RN66
Cryptosporidium parvum Iowa II
Cryptosporidium hominis TU502
Theileria annulata strain Ankara
Theileria parva strain Muguga
Plasmodium falciparum
Plasmodium knowlesi
Plasmodium vivax
Plasmodium chabaudi
Plasmodium yoelii yoelii 17XNL
Plasmodium berghei
Phaeodactylum tricornutum
Thalassiosira pseudonana
Pythium ultimum
Albugo laibachii
Hyaloperonospora arabidopsidis
Phytophthora ramorum
Phytophthora infestans
Phytophthora sojae
Trypanosoma brucei
Trypanosoma cruzi strain CL Brener
Leishmania braziliensis MHOM BR 75 M2904
Leishmania mexicana MHOM GT 2001 U1103
Leishmania major
Leishmania donovani
Leishmania infantum JPCM5
Entamoeba histolytica
Entamoeba dispar SAW760
Dictyostelium discoideum
Dictyostelium purpureum
Trichomonas vaginalis G3
Trichomonas vaginalis
Naegleria gruberi
Giardia lamblia ATCC 50803
OG presence
OG absence
Inferred root
Dscore = 0.826
Pvalue = 1.18e−04
AdjPvalue = 1.41e−02

COG2608

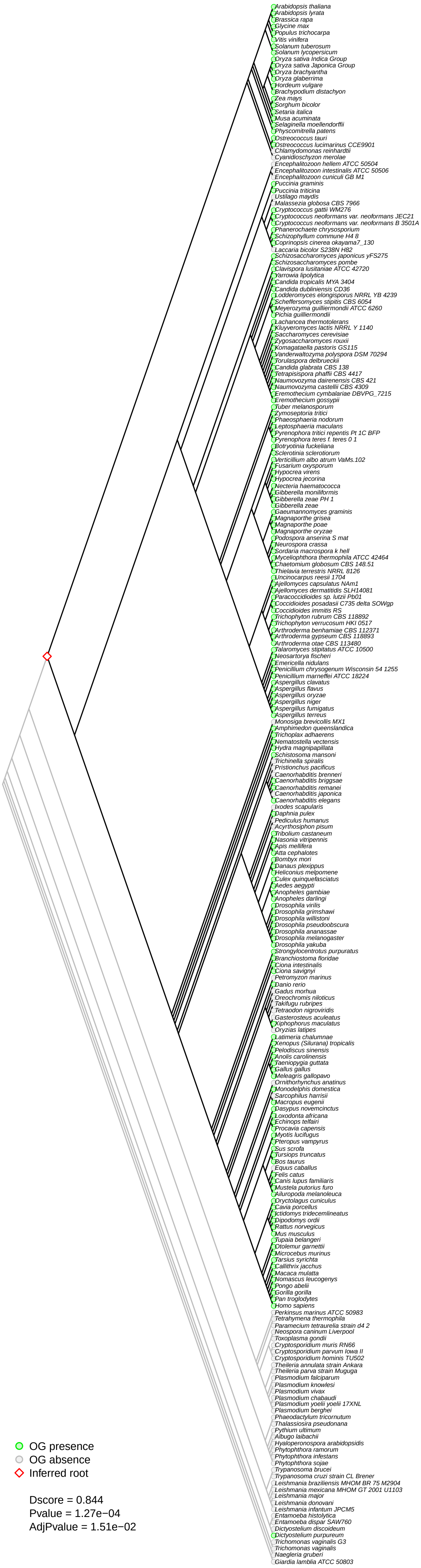
Arabidopsis thaliana
Arabidopsis lyrata
Brassica rapa
Glycine max
Populus trichocarpa
Vitis vinifera
Solanum tuberosum
Solanum lycopersicum
Oryza sativa Indica Group
Oryza sativa Japonica Group
Oryza brachyantha
Oryza glaberrima
Hordeum vulgare
Brachypodium distachyon
Zea mays
Sorghum bicolor
Setaria italica
Musa acuminata
Selaginella moellendorffii
Physcomitrella patens
Ostreococcus tauri
Ostreococcus lucimarinus CCE9901
Chlamydomonas reinhardtii
Cyanidioschyzon merolae
Encephalitozoon hellem ATCC 50504
Encephalitozoon intestinalis ATCC 50506
Encephalitozoon cuniculi GB M1
Puccinia graminis
Puccinia triticina
Ustilago maydis
Malassezia globosa CBS 7966
Cryptococcus gattii WM276
Cryptococcus neoformans var. neoformans JEC21
Cryptococcus neoformans var. neoformans B 3501A
Phanerochaete chrysosporium
Schizophyllum commune H4 8
Coprinopsis cinerea okayama7_130
Laccaria bicolor S238N H82
Schizosaccharomyces japonicus yFS275
Schizosaccharomyces pombe
Clavispora lusitaniae ATCC 42720
Yarrowia lipolytica
Candida tropicalis MYA 3404
Candida dubliniensis CD36
Lodderomyces elongisporus NRRL YB 4239
Scheffersomyces stipitis CBS 6054
Meyerozyma guilliermondii ATCC 6260
Pichia guilliermondii
Lachancea thermotolerans
Kluyveromyces lactis NRRL Y 1140
Saccharomyces cerevisiae
Zygosaccharomyces rouxii
Komagataella pastoris GS115
Vanderwaltozyma polyspora DSM 70294
Torulaspora delbrueckii
Candida glabrata CBS 138
Tetrapisispora phaffii CBS 4417
Naumovozyma dairenensis CBS 421
Naumovozyma castellii CBS 4309
Eremothecium cymbalariae DBVPG_7215
Eremothecium gossypii
Tuber melanosporum
Zymoseptoria tritici
Phaeosphaeria nodorum
Leptosphaeria maculans
Pyrenophora tritici repentis Pt 1C BFP
Pyrenophora teres f. teres 0 1
Botryotinia fuckeliana
Sclerotinia sclerotiorum
Verticillium albo atrum VaMs.102
Fusarium oxysporum
Hypocrea virens
Hypocrea jecorina
Nectria haematococca
Gibberella moniliformis
Gibberella zeae PH 1
Gibberella zeae
Gaeumannomyces graminis
Magnaporthe grisea
Magnaporthe poae
Magnaporthe oryzae
Podospora anserina S mat
Neurospora crassa
Sordaria macrospora k hell
Myceliophthora thermophila ATCC 42464
Chaetomium globosum CBS 148.51
Thielavia terrestris NRRL 8126
Uncinocarpus reesii 1704
Ajellomyces capsulatus NAm1
Ajellomyces dermatitidis SLH14081
Paracoccidioides sp. lutzii Pb01
Coccidioides posadasii C735 delta SOWgp
Coccidioides immitis RS
Trichophyton rubrum CBS 118892
Trichophyton verrucosum HKI 0517
Arthroderma benhamiae CBS 112371
Arthroderma gypseum CBS 118893
Arthroderma otae CBS 113480
Talaromyces stipitatus ATCC 10500
Neosartorya fischeri
Emericella nidulans
Penicillium chrysogenum Wisconsin 54 1255
Penicillium marneffei ATCC 18224
Aspergillus clavatus
Aspergillus flavus
Aspergillus oryzae
Aspergillus niger
Aspergillus fumigatus
Aspergillus terreus
Monosiga brevicollis MX1
Amphimedon queenslandica
Trichoplax adhaerens
Nematostella vectensis
Hydra magnipapillata
Schistosoma mansoni
Trichinella spiralis
Pristionchus pacificus
Caenorhabditis brenneri
Caenorhabditis briggsae
Caenorhabditis remanei
Caenorhabditis japonica
Caenorhabditis elegans
Ixodes scapularis
Daphnia pulex
Pediculus humanus
Acyrthosiphon pisum
Tribolium castaneum
Nasonia vitripennis
Apis mellifera
Atta cephalotes
Bombyx mori
Danaus plexippus
Heliconius melpomene
Culex quinquefasciatus
Aedes aegypti
Anopheles gambiae
Anopheles darlingi
Drosophila virilis
Drosophila grimshawi
Drosophila willistoni
Drosophila pseudoobscura
Drosophila ananassae
Drosophila melanogaster
Drosophila yakuba
Strongylocentrotus purpuratus
Branchiostoma floridae
Ciona intestinalis
Ciona savignyi
Petromyzon marinus
Danio rerio
Gadus morhua
Oreochromis niloticus
Takifugu rubripes
Tetraodon nigroviridis
Gasterosteus aculeatus
Xiphophorus maculatus
Oryzias latipes
Latimeria chalumnae
Xenopus (Silurana) tropicalis
Pelodiscus sinensis
Anolis carolinensis
Taeniopygia guttata
Gallus gallus
Meleagris gallopavo
Ornithorhynchus anatinus
Monodelphis domestica
Sarcophilus harrisii
Macropus eugenii
Dasypus novemcinctus
Loxodonta africana
Echinops telfairi
Procavia capensis
Myotis lucifugus
Pteropus vampyrus
Sus scrofa
Tursiops truncatus
Bos taurus
Equus caballus
Felis catus
Canis lupus familiaris
Mustela putorius furo
Ailuropoda melanoleuca
Oryctolagus cuniculus
Cavia porcellus
Ictidomys tridecemlineatus
Dipodomys ordii
Rattus norvegicus
Mus musculus
Tupaia belangeri
Otolemur garnettii
Microcebus murinus
Tarsius syrichta
Callithrix jacchus
Macaca mulatta
Nomascus leucogenys
Pongo abelii
Gorilla gorilla
Pan troglodytes
Homo sapiens
Perkinsus marinus ATCC 50983
Tetrahymena thermophila
Paramecium tetraurelia strain d4 2
Neospora caninum Liverpool
Toxoplasma gondii
Cryptosporidium muris RN66
Cryptosporidium parvum Iowa II
Cryptosporidium hominis TU502
Theileria annulata strain Ankara
Theileria parva strain Muguga
Plasmodium falciparum
Plasmodium knowlesi
Plasmodium vivax
Plasmodium chabaudi
Plasmodium yoelii yoelii 17XNL
Plasmodium berghei
Phaeodactylum tricornutum
Thalassiosira pseudonana
Pythium ultimum
Albugo laibachii
Hyaloperonospora arabidopsidis
Phytophthora ramorum
Phytophthora infestans
Phytophthora sojae
Trypanosoma brucei
Trypanosoma cruzi strain CL Brener
Leishmania braziliensis MHOM BR 75 M2904
Leishmania mexicana MHOM GT 2001 U1103
Leishmania major
Leishmania donovani
Leishmania infantum JPCM5
Entamoeba histolytica
Entamoeba dispar SAW760
Dictyostelium discoideum
Dictyostelium purpureum
Trichomonas vaginalis G3
Trichomonas vaginalis
Naegleria gruberi
Giardia lamblia ATCC 50803
OG presence
OG absence
Inferred root
Dscore = 0.844
Pvalue = 1.27e−04
AdjPvalue = 1.51e−02

COG3794

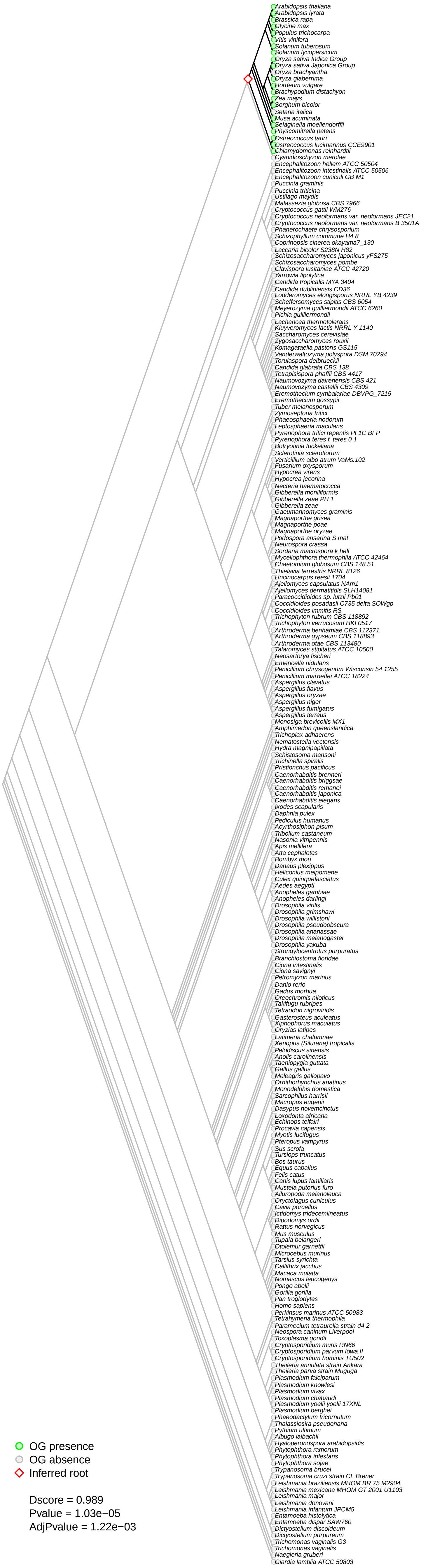

Arabidopsis thaliana
Arabidopsis lyrata
Brassica rapa
Glycine max
Populus trichocarpa
Vitis vinifera
Solanum tuberosum
Solanum lycopersicum
Oryza sativa Indica Group
Oryza sativa Japonica Group
Oryza brachyantha
Oryza glaberrima
Hordeum vulgare
Brachypodium distachyon
Zea mays
Sorghum bicolor
Setaria italica
Musa acuminata
Selaginella moellendorffii
Physcomitrella patens
Ostreococcus tauri
Ostreococcus lucimarinus CCE9901
Chlamydomonas reinhardtii
Cyanidioschyzon merolae
Encephalitozoon hellem ATCC 50504
Encephalitozoon intestinalis ATCC 50506
Encephalitozoon cuniculi GB M1
Puccinia graminis
Puccinia triticina
Ustilago maydis
Malassezia globosa CBS 7966
Cryptococcus gattii WM276
Cryptococcus neoformans var. neoformans JEC21
Cryptococcus neoformans var. neoformans B 3501A
Phanerochaete chrysosporium
Schizophyllum commune H4 8
Coprinopsis cinerea okayama7_130
Laccaria bicolor S238N H82
Schizosaccharomyces japonicus yFS275
Schizosaccharomyces pombe
Clavispora lusitaniae ATCC 42720
Yarrowia lipolytica
Candida tropicalis MYA 3404
Candida dubliniensis CD36
Lodderomyces elongisporus NRRL YB 4239
Scheffersomyces stipitis CBS 6054
Meyerozyma guilliermondii ATCC 6260
Pichia guilliermondii
Lachancea thermotolerans
Kluyveromyces lactis NRRL Y 1140
Saccharomyces cerevisiae
Zygosaccharomyces rouxii
Komagataella pastoris GS115
Vanderwaltozyma polyspora DSM 70294
Torulaspora delbrueckii
Candida glabrata CBS 138
Tetrapisispora phaffii CBS 4417
Naumovozyma dairenensis CBS 421
Naumovozyma castellii CBS 4309
Eremothecium cymbalariae DBVPG_7215
Eremothecium gossypii
Tuber melanosporum
Zymoseptoria tritici
Phaeosphaeria nodorum
Leptosphaeria maculans
Pyrenophora tritici repentis Pt 1C BFP
Pyrenophora teres f. teres 0 1
Botryotinia fuckeliana
Sclerotinia sclerotiorum
Verticillium albo atrum VaMs.102
Fusarium oxysporum
Hypocrea virens
Hypocrea jecorina
Nectria haematococca
Gibberella moniliformis
Gibberella zeae PH 1
Gibberella zeae
Gaeumannomyces graminis
Magnaporthe grisea
Magnaporthe poae
Magnaporthe oryzae
Podospora anserina S mat
Neurospora crassa
Sordaria macrospora k hell
Myceliophthora thermophila ATCC 42464
Chaetomium globosum CBS 148.51
Thielavia terrestris NRRL 8126
Uncinocarpus reesii 1704
Ajellomyces capsulatus NAm1
Ajellomyces dermatitidis SLH14081
Paracoccidioides sp. lutzii Pb01
Coccidioides posadasii C735 delta SOWgp
Coccidioides immitis RS
Trichophyton rubrum CBS 118892
Trichophyton verrucosum HKI 0517
Arthroderma benhamiae CBS 112371
Arthroderma gypseum CBS 118893
Arthroderma otae CBS 113480
Talaromyces stipitatus ATCC 10500
Neosartorya fischeri
Emericella nidulans
Penicillium chrysogenum Wisconsin 54 1255
Penicillium marneffei ATCC 18224
Aspergillus clavatus
Aspergillus flavus
Aspergillus oryzae
Aspergillus niger
Aspergillus fumigatus
Aspergillus terreus
Monosiga brevicollis MX1
Amphimedon queenslandica
Trichoplax adhaerens
Nematostella vectensis
Hydra magnipapillata
Schistosoma mansoni
Trichinella spiralis
Pristionchus pacificus
Caenorhabditis brenneri
Caenorhabditis briggsae
Caenorhabditis remanei
Caenorhabditis japonica
Caenorhabditis elegans
Ixodes scapularis
Daphnia pulex
Pediculus humanus
Acyrthosiphon pisum
Tribolium castaneum
Nasonia vitripennis
Apis mellifera
Atta cephalotes
Bombyx mori
Danaus plexippus
Heliconius melpomene
Culex quinquefasciatus
Aedes aegypti
Anopheles gambiae
Anopheles darlingi
Drosophila virilis
Drosophila grimshawi
Drosophila willistoni
Drosophila pseudoobscura
Drosophila ananassae
Drosophila melanogaster
Drosophila yakuba
Strongylocentrotus purpuratus
Branchiostoma floridae
Ciona intestinalis
Ciona savignyi
Petromyzon marinus
Danio rerio
Gadus morhua
Oreochromis niloticus
Takifugu rubripes
Tetraodon nigroviridis
Gasterosteus aculeatus
Xiphophorus maculatus
Oryzias latipes
Latimeria chalumnae
Xenopus (Silurana) tropicalis
Pelodiscus sinensis
Anolis carolinensis
Taeniopygia guttata
Gallus gallus
Meleagris gallopavo
Ornithorhynchus anatinus
Monodelphis domestica
Sarcophilus harrisii
Macropus eugenii
Dasypus novemcinctus
Loxodonta africana
Echinops telfairi
Procavia capensis
Myotis lucifugus
Pteropus vampyrus
Sus scrofa
Tursiops truncatus
Bos taurus
Equus caballus
Felis catus
Canis lupus familiaris
Mustela putorius furo
Ailuropoda melanoleuca
Oryctolagus cuniculus
Cavia porcellus
Ictidomys tridecemlineatus
Dipodomys ordii
Rattus norvegicus
Mus musculus
Tupaia belangeri
Otolemur garnettii
Microcebus murinus
Tarsius syrichta
Callithrix jacchus
Macaca mulatta
Nomascus leucogenys
Pongo abelii
Gorilla gorilla
Pan troglodytes
Homo sapiens
Perkinsus marinus ATCC 50983
Tetrahymena thermophila
Paramecium tetraurelia strain d4 2
Neospora caninum Liverpool
Toxoplasma gondii
Cryptosporidium muris RN66
Cryptosporidium parvum Iowa II
Cryptosporidium hominis TU502
Theileria annulata strain Ankara
Theileria parva strain Muguga
Plasmodium falciparum
Plasmodium knowlesi
Plasmodium vivax
Plasmodium chabaudi
Plasmodium yoelii yoelii 17XNL
Plasmodium berghei
Phaeodactylum tricornutum
Thalassiosira pseudonana
Pythium ultimum
Albugo laibachii
Hyaloperonospora arabidopsidis
Phytophthora ramorum
Phytophthora infestans
Phytophthora sojae
Trypanosoma brucei
Trypanosoma cruzi strain CL Brener
Leishmania braziliensis MHOM BR 75 M2904
Leishmania mexicana MHOM GT 2001 U1103
Leishmania major
Leishmania donovani
Leishmania infantum JPCM5
Entamoeba histolytica
Entamoeba dispar SAW760
Dictyostelium discoideum
Dictyostelium purpureum
Trichomonas vaginalis G3
Trichomonas vaginalis
Naegleria gruberi
Giardia lamblia ATCC 50803
OG presence
OG absence
Inferred root
Dscore = 0.989
Pvalue = 1.03e−05
AdjPvalue = 1.22e−03

COG4243

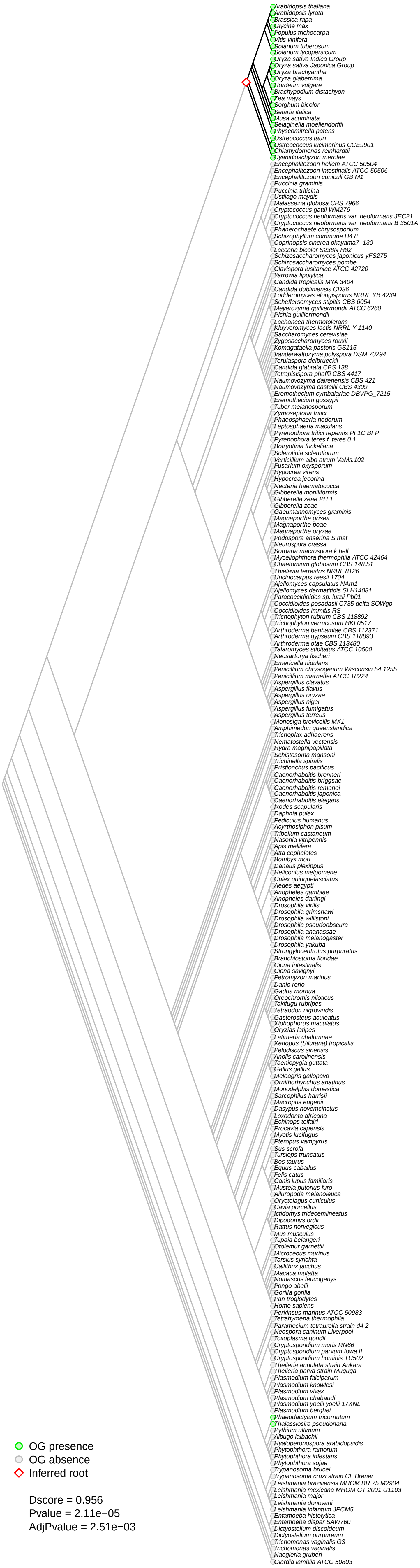
Arabidopsis thaliana
Arabidopsis lyrata
Brassica rapa
Glycine max
Populus trichocarpa
Vitis vinifera
Solanum tuberosum
Solanum lycopersicum
Oryza sativa Indica Group
Oryza sativa Japonica Group
Oryza brachyantha
Oryza glaberrima
Hordeum vulgare
Brachypodium distachyon
Zea mays
Sorghum bicolor
Setaria italica
Musa acuminata
Selaginella moellendorffii
Physcomitrella patens
Ostreococcus tauri
Ostreococcus lucimarinus CCE9901
Chlamydomonas reinhardtii
Cyanidioschyzon merolae
Encephalitozoon hellem ATCC 50504
Encephalitozoon intestinalis ATCC 50506
Encephalitozoon cuniculi GB M1
Puccinia graminis
Puccinia triticina
Ustilago maydis
Malassezia globosa CBS 7966
Cryptococcus gattii WM276
Cryptococcus neoformans var. neoformans JEC21
Cryptococcus neoformans var. neoformans B 3501A
Phanerochaete chrysosporium
Schizophyllum commune H4 8
Coprinopsis cinerea okayama7_130
Laccaria bicolor S238N H82
Schizosaccharomyces japonicus yFS275
Schizosaccharomyces pombe
Clavispora lusitaniae ATCC 42720
Yarrowia lipolytica
Candida tropicalis MYA 3404
Candida dubliniensis CD36
Lodderomyces elongisporus NRRL YB 4239
Scheffersomyces stipitis CBS 6054
Meyerozyma guilliermondii ATCC 6260
Pichia guilliermondii
Lachancea thermotolerans
Kluyveromyces lactis NRRL Y 1140
Saccharomyces cerevisiae
Zygosaccharomyces rouxii
Komagataella pastoris GS115
Vanderwaltozyma polyspora DSM 70294
Torulaspora delbrueckii
Candida glabrata CBS 138
Tetrapisispora phaffii CBS 4417
Naumovozyma dairenensis CBS 421
Naumovozyma castellii CBS 4309
Eremothecium cymbalariae DBVPG_7215
Eremothecium gossypii
Tuber melanosporum
Zymoseptoria tritici
Phaeosphaeria nodorum
Leptosphaeria maculans
Pyrenophora tritici repentis Pt 1C BFP
Pyrenophora teres f. teres 0 1
Botryotinia fuckeliana
Sclerotinia sclerotiorum
Verticillium albo atrum VaMs.102
Fusarium oxysporum
Hypocrea virens
Hypocrea jecorina
Necteria haematococca
Gibberella moniliformis
Gibberella zeae PH 1
Gibberella zeae
Gaeumannomyces graminis
Magnaporthe grisea
Magnaporthe poae
Magnaporthe oryzae
Podospora anserina S mat
Neurospora crassa
Sordaria macrospora k hell
Myceliophthora thermophila ATCC 42464
Chaetomium globosum CBS 148.51
Thielavia terrestris NRRL 8126
Uncinocarpus reesii 1704
Ajellomyces capsulatus NAm1
Ajellomyces dermatitidis SLH14081
Paracoccidioides sp. lutzii Pb01
Coccidioides posadasii C735 delta SOWgp
Coccidioides immitis RS
Trichophyton rubrum CBS 118892
Trichophyton verrucosum HKI 0517
Arthroderma benhamiae CBS 112371
Arthroderma gypseum CBS 118893
Arthroderma otae CBS 113480
Talaromyces stipitatus ATCC 10500
Neosartorya fischeri
Emericella nidulans
Penicillium chrysogenum Wisconsin 54 1255
Penicillium marneffei ATCC 18224
Aspergillus clavatus
Aspergillus flavus
Aspergillus oryzae
Aspergillus niger
Aspergillus fumigatus
Aspergillus terreus
Monosiga brevicollis MX1
Amphimedon queenslandica
Trichoplax adhaerens
Nematostella vectensis
Hydra magnipapillata
Schistosoma mansoni
Trichinella spiralis
Pristionchus pacificus
Caenorhabditis brenneri
Caenorhabditis briggsae
Caenorhabditis remanei
Caenorhabditis japonica
Caenorhabditis elegans
Ixodes scapularis
Daphnia pulex
Pediculus humanus
Acyrthosiphon pisum
Tribolium castaneum
Nasonia vitripennis
Apis mellifera
Atta cephalotes
Bombyx mori
Danaus plexippus
Heliconius melpomene
Culex quinquefasciatus
Aedes aegypti
Anopheles gambiae
Anopheles darlingi
Drosophila virilis
Drosophila grimshawi
Drosophila willistoni
Drosophila pseudoobscura
Drosophila ananassae
Drosophila melanogaster
Drosophila yakuba
Strongylocentrotus purpuratus
Branchiostoma floridae
Ciona intestinalis
Ciona savignyi
Petromyzon marinus
Danio rerio
Gadus morhua
Oreochromis niloticus
Takifugu rubripes
Tetraodon nigroviridis
Gasterosteus aculeatus
Xiphophorus maculatus
Oryzias latipes
Latimeria chalumnae
Xenopus (Silurana) tropicalis
Pelodiscus sinensis
Anolis carolinensis
Taeniopygia guttata
Gallus gallus
Meleagris gallopavo
Ornithorhynchus anatinus
Monodelphis domestica
Sarcophilus harrisii
Macropus eugenii
Dasypus novemcinctus
Loxodonta africana
Echinops telfairi
Procavia capensis
Myotis lucifugus
Pteropus vampyrus
Sus scrofa
Tursiops truncatus
Bos taurus
Equus caballus
Felis catus
Canis lupus familiaris
Mustela putorius furo
Ailuropoda melanoleuca
Oryctolagus cuniculus
Cavia porcellus
Ictidomys tridecemlineatus
Dipodomys ordii
Rattus norvegicus
Mus musculus
Tupaia belangeri
Otolemur garnettii
Microcebus murinus
Tarsius syrichta
Callithrix jacchus
Macaca mulatta
Nomascus leucogenys
Pongo abelii
Gorilla gorilla
Pan troglodytes
Homo sapiens
Perkinsus marinus ATCC 50983
Tetrahymena thermophila
Paramecium tetraurelia strain d4 2
Neospora caninum Liverpool
Toxoplasma gondii
Cryptosporidium muris RN66
Cryptosporidium parvum Iowa II
Cryptosporidium hominis TU502
Theileria annulata strain Ankara
Theileria parva strain Muguga
Plasmodium falciparum
Plasmodium knowlesi
Plasmodium vivax
Plasmodium chabaudi
Plasmodium yoelii yoelii 17XNL
Plasmodium berghei
Phaeodactylum tricornutum
Thalassiosira pseudonana
Pythium ultimum
Albugo laibachii
Hyaloperonospora arabidopsidis
Phytophthora ramorum
Phytophthora infestans
Phytophthora sojae
Trypanosoma brucei
Trypanosoma cruzi strain CL Brener
Leishmania braziliensis MHOM BR 75 M2904
Leishmania mexicana MHOM GT 2001 U1103
Leishmania major
Leishmania donovani
Leishmania infantum JPCM5
Entamoeba histolytica
Entamoeba dispar SAW760
Dictyostelium discoideum
Dictyostelium purpureum
Trichomonas vaginalis G3
Trichomonas vaginalis
Naegleria gruberi
Giardia lamblia ATCC 50803
OG presence
OG absence
Inferred root
Dscore = 0.956
Pvalue = 2.11e−05
AdjPvalue = 2.51e−03

COG5119

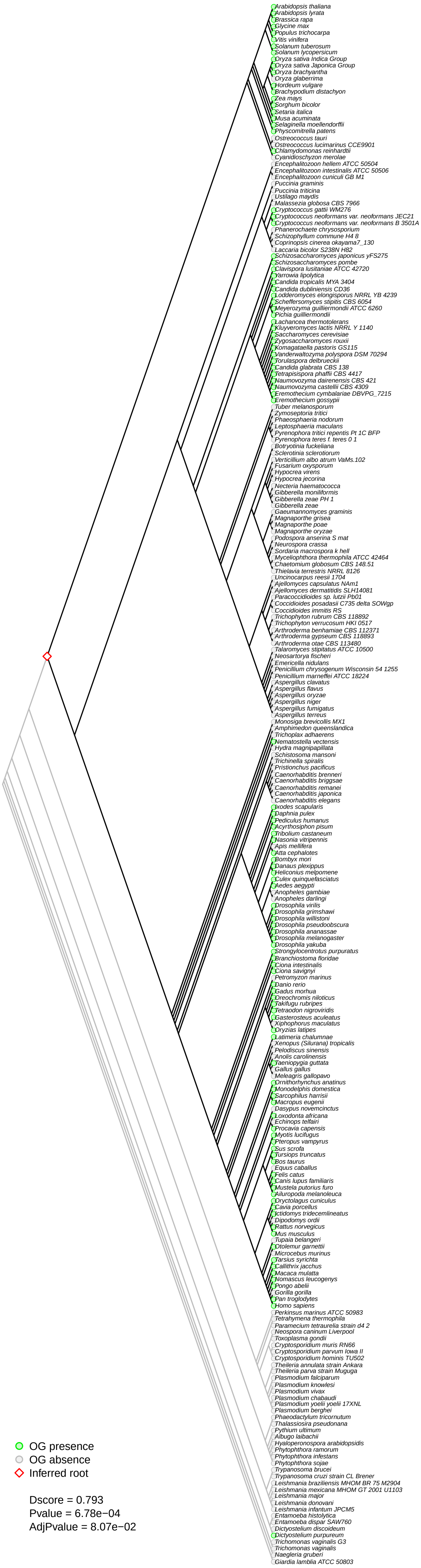

Arabidopsis thaliana
Arabidopsis lyrata
Brassica rapa
Glycine max
Populus trichocarpa
Vitis vinifera
Solanum tuberosum
Solanum lycopersicum
Oryza sativa Indica Group
Oryza sativa Japonica Group
Oryza brachyantha
Oryza glaberrima
Hordeum vulgare
Brachypodium distachyon
Zea mays
Sorghum bicolor
Setaria italica
Musa acuminata
Selaginella moellendorffii
Physcomitrella patens
Ostreococcus tauri
Ostreococcus lucimarinus CCE9901
Chlamydomonas reinhardtii
Cyanidioschyzon merolae
Encephalitozoon hellem ATCC 50504
Encephalitozoon intestinalis ATCC 50506
Encephalitozoon cuniculi GB M1
Puccinia graminis
Puccinia triticina
Ustilago maydis
Malassezia globosa CBS 7966
Cryptococcus gattii WM276
Cryptococcus neoformans var. neoformans JEC21
Cryptococcus neoformans var. neoformans B 3501A
Phanerochaete chrysosporium
Schizophyllum commune H4 8
Coprinopsis cinerea okayama7_130
Laccaria bicolor S238N H82
Schizosaccharomyces japonicus yFS275
Schizosaccharomyces pombe
Clavispora lusitaniae ATCC 42720
Yarrowia lipolytica
Candida tropicalis MYA 3404
Candida dubliniensis CD36
Lodderomyces elongisporus NRRL YB 4239
Scheffersomyces stipitis CBS 6054
Meyerozyma guilliermondii ATCC 6260
Pichia guilliermondii
Lachancea thermotolerans
Kluyveromyces lactis NRRL Y 1140
Saccharomyces cerevisiae
Zygosaccharomyces rouxii
Komagataella pastoris GS115
Vanderwaltozyma polyspora DSM 70294
Torulaspora delbrueckii
Candida glabrata CBS 138
Tetrapisispora phaffii CBS 4417
Naumovozyma dairenensis CBS 421
Naumovozyma castellii CBS 4309
Eremothecium cymbalariae DBVPG_7215
Eremothecium gossypii
Tuber melanosporum
Zymoseptoria tritici
Phaeosphaeria nodorum
Leptosphaeria maculans
Pyrenophora tritici repentis Pt 1C BFP
Pyrenophora teres f. teres 0 1
Botryotinia fuckeliana
Sclerotinia sclerotiorum
Verticillium albo atrum VaMs.102
Fusarium oxysporum
Hypocrea virens
Hypocrea jecorina
Nectria haematococca
Gibberella moniliformis
Gibberella zeae PH 1
Gibberella zeae
Gaeumannomyces graminis
Magnaporthe grisea
Magnaporthe poae
Magnaporthe oryzae
Podospora anserina S mat
Neurospora crassa
Sordaria macrospora k hell
Myceliophthora thermophila ATCC 42464
Chaetomium globosum CBS 148.51
Thielavia terrestris NRRL 8126
Uncinocarpus reesii 1704
Ajellomyces capsulatus NAm1
Ajellomyces dermatitidis SLH14081
Paracoccidioides sp. lutzii Pb01
Coccidioides posadasii C735 delta SOWgp
Coccidioides immitis RS
Trichophyton rubrum CBS 118892
Trichophyton verrucosum HKI 0517
Arthroderma benhamiae CBS 112371
Arthroderma gypseum CBS 118893
Arthroderma otae CBS 113480
Talaromyces stipitatus ATCC 10500
Neosartorya fischeri
Emericella nidulans
Penicillium chrysogenum Wisconsin 54 1255
Penicillium marneffei ATCC 18224
Aspergillus clavatus
Aspergillus flavus
Aspergillus oryzae
Aspergillus niger
Aspergillus fumigatus
Aspergillus terreus
Monosiga brevicollis MX1
Amphimedon queenslandica
Trichoplax adhaerens
Nematostella vectensis
Hydra magnipapillata
Schistosoma mansoni
Trichinella spiralis
Pristionchus pacificus
Caenorhabditis brenneri
Caenorhabditis briggsae
Caenorhabditis remanei
Caenorhabditis japonica
Caenorhabditis elegans
Ixodes scapularis
Daphnia pulex
Pediculus humanus
Acyrthosiphon pisum
Tribolium castaneum
Nasonia vitripennis
Apis mellifera
Atta cephalotes
Bombyx mori
Danaus plexippus
Heliconius melpomene
Culex quinquefasciatus
Aedes aegypti
Anopheles gambiae
Anopheles darlingi
Drosophila virilis
Drosophila grimshawi
Drosophila willistoni
Drosophila pseudoobscura
Drosophila ananassae
Drosophila melanogaster
Drosophila yakuba
Strongylocentrotus purpuratus
Branchiostoma floridae
Ciona intestinalis
Ciona savignyi
Petromyzon marinus
Danio rerio
Gadus morhua
Oreochromis niloticus
Takifugu rubripes
Tetraodon nigroviridis
Gasterosteus aculeatus
Xiphophorus maculatus
Oryzias latipes
Latimeria chalumnae
Xenopus (Silurana) tropicalis
Pelodiscus sinensis
Anolis carolinensis
Taeniopygia guttata
Gallus gallus
Meleagris gallopavo
Ornithorhynchus anatinus
Monodelphis domestica
Sarcophilus harrisii
Macropus eugenii
Dasypus novemcinctus
Loxodonta africana
Echinops telfairi
Procavia capensis
Myotis lucifugus
Pteropus vampyrus
Sus scrofa
Tursiops truncatus
Bos taurus
Equus caballus
Felis catus
Canis lupus familiaris
Mustela putorius furo
Ailuropoda melanoleuca
Oryctolagus cuniculus
Cavia porcellus
Ictidomys tridecemlineatus
Dipodomys ordii
Rattus norvegicus
Mus musculus
Tupaia belangeri
Otolemur garnettii
Microcebus murinus
Tarsius syrichta
Callithrix jacchus
Macaca mulatta
Nomascus leucogenys
Pongo abelii
Gorilla gorilla
Pan troglodytes
Homo sapiens
Perkinsus marinus ATCC 50983
Tetrahymena thermophila
Paramecium tetraurelia strain d4 2
Neospora caninum Liverpool
Toxoplasma gondii
Cryptosporidium muris RN66
Cryptosporidium parvum Iowa II
Cryptosporidium hominis TU502
Theileria annulata strain Ankara
Theileria parva strain Muguga
Plasmodium falciparum
Plasmodium knowlesi
Plasmodium vivax
Plasmodium chabaudi
Plasmodium yoelii yoelii 17XNL
Plasmodium berghei
Phaeodactylum tricornutum
Thalassiosira pseudonana
Pythium ultimum
Albugo laibachii
Hyaloperonospora arabidopsidis
Phytophthora ramorum
Phytophthora infestans
Phytophthora sojae
Trypanosoma brucei
Trypanosoma cruzi strain CL Brener
Leishmania braziliensis MHOM BR 75 M2904
Leishmania mexicana MHOM GT 2001 U1103
Leishmania major
Leishmania donovani
Leishmania infantum JPCM5
Entamoeba histolytica
Entamoeba dispar SAW760
Dictyostelium discoideum
Dictyostelium purpureum
Trichomonas vaginalis G3
Trichomonas vaginalis
Naegleria gruberi
Giardia lamblia ATCC 50803
OG presence
OG absence
Inferred root
Dscore = 0.793
Pvalue = 6.78e−04
AdjPvalue = 8.07e−02

COG5601

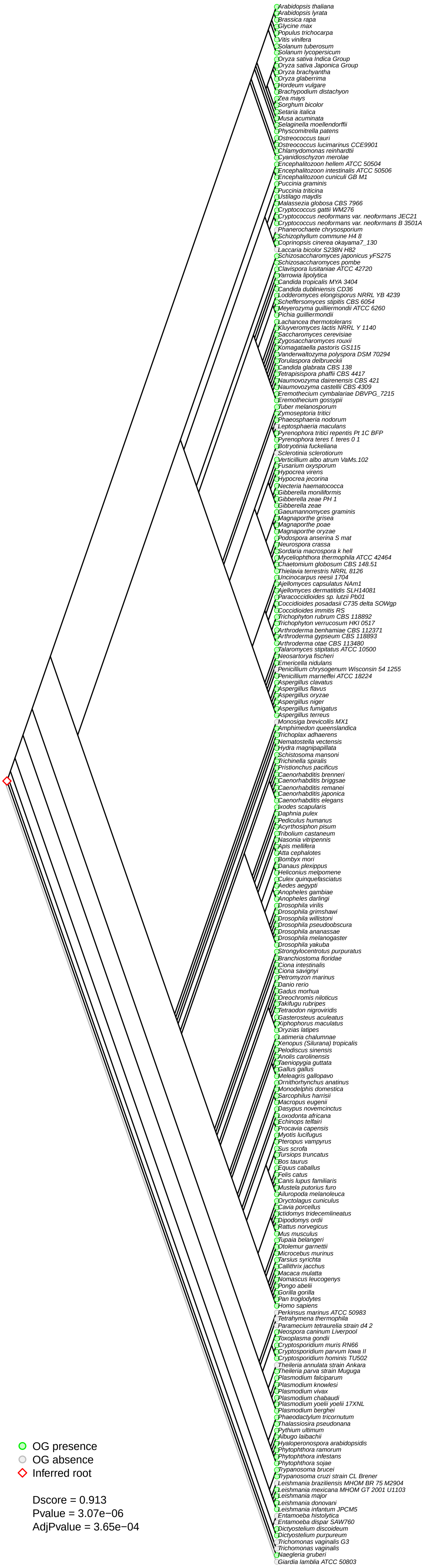

Dscore = 0.913
Pvalue = 3.07e−06
AdjPvalue = 3.65e−04

NOG00684

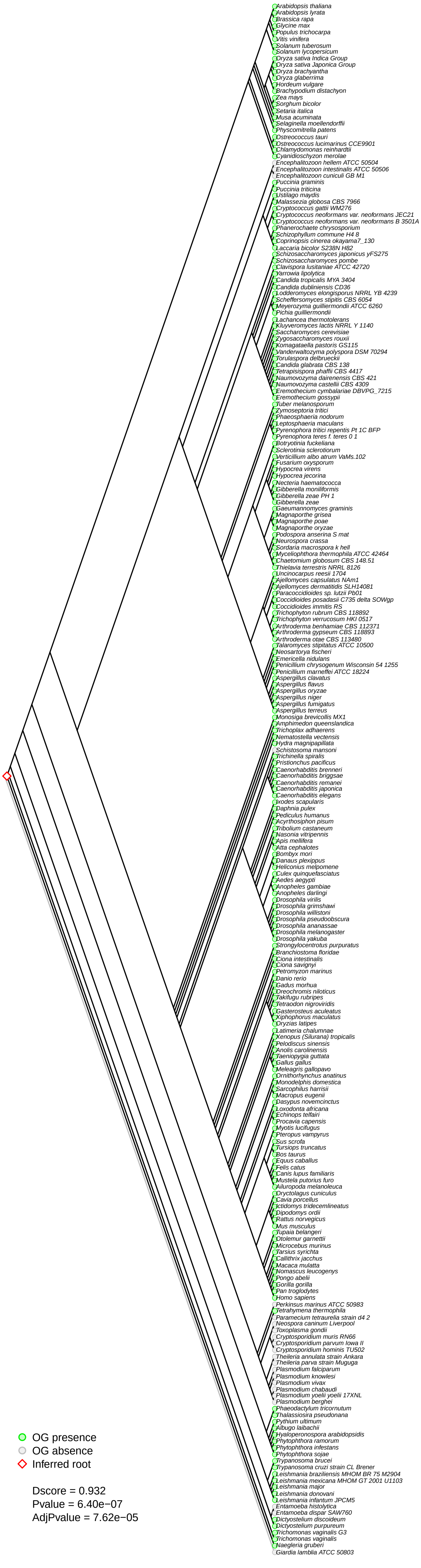

Dscore = 0.932
Pvalue = 6.40e−07
AdjPvalue = 7.62e−05

NOG01122

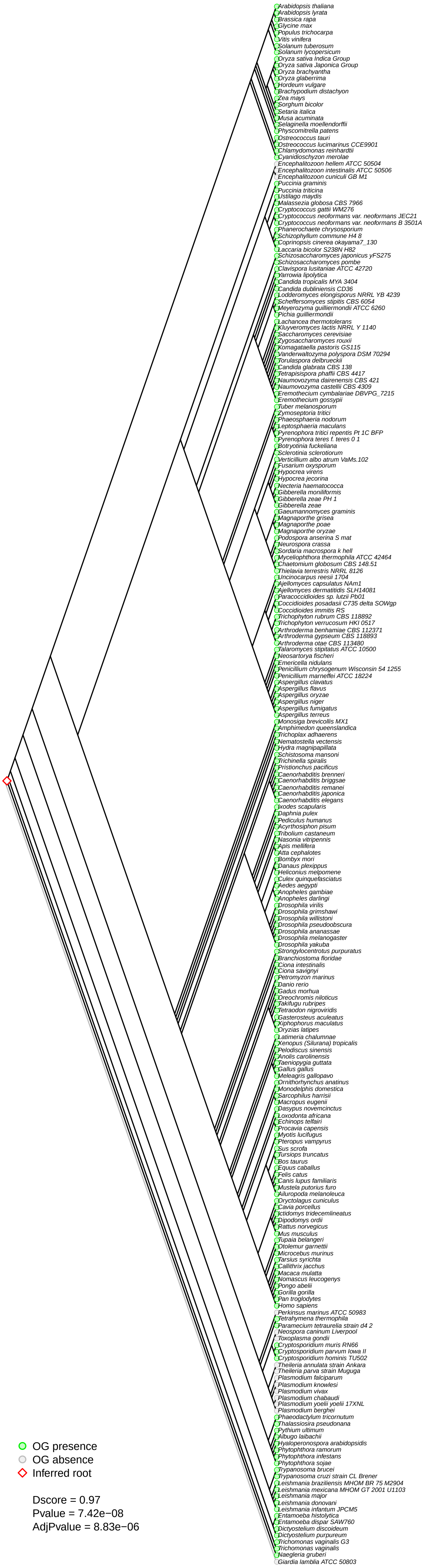

OG presence
OG absence
Inferred root

Dscore = 0.97
Pvalue = 7.42e−08
AdjPvalue = 8.83e−06

NOG02511

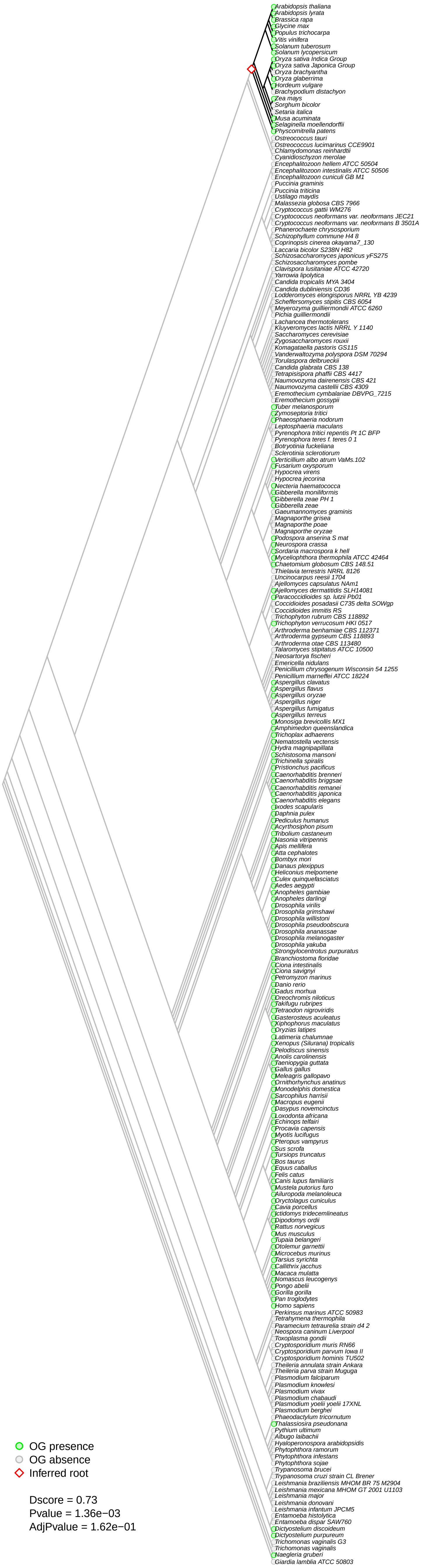

Dscore = 0.73
Pvalue = 1.36e−03
AdjPvalue = 1.62e−01

NOG04600

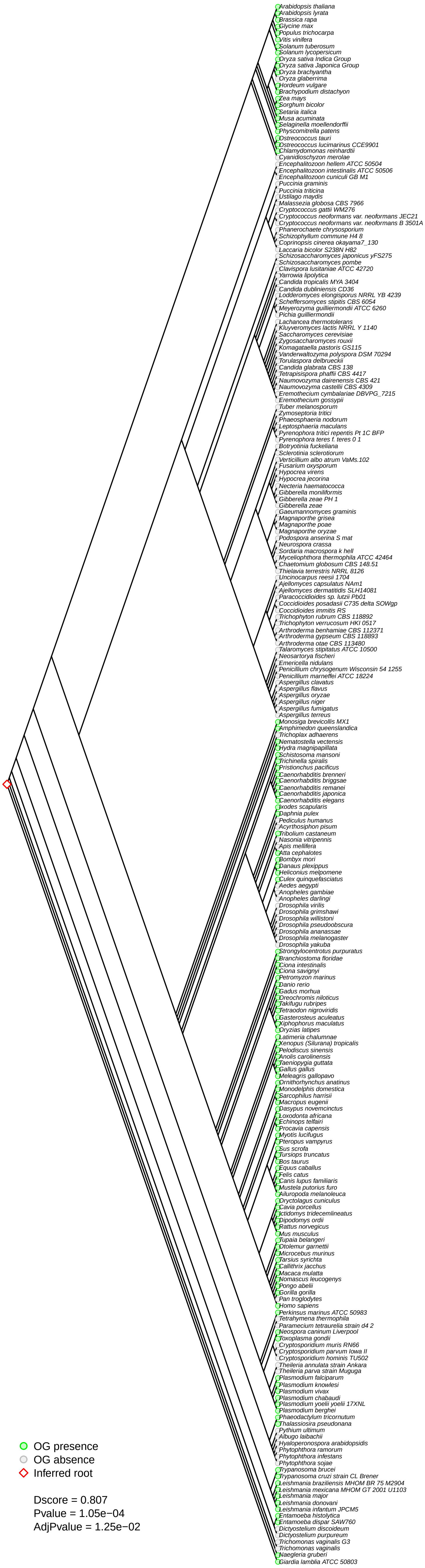
Arabidopsis thaliana
Arabidopsis lyrata
Brassica rapa
Glycine max
Populus trichocarpa
Vitis vinifera
Solanum tuberosum
Solanum lycopersicum
Oryza sativa Indica Group
Oryza sativa Japonica Group
Oryza brachyantha
Oryza glaberrima
Hordeum vulgare
Brachypodium distachyon
Zea mays
Sorghum bicolor
Setaria italica
Musa acuminata
Selaginella moellendorffii
Physcomitrella patens
Ostreococcus tauri
Ostreococcus lucimarinus CCE9901
Chlamydomonas reinhardtii
Cyanidioschyzon merolae
Encephalitozoon hellem ATCC 50504
Encephalitozoon intestinalis ATCC 50506
Encephalitozoon cuniculi GB M1
Puccinia graminis
Puccinia triticina
Ustilago maydis
Malassezia globosa CBS 7966
Cryptococcus gattii WM276
Cryptococcus neoformans var. neoformans JEC21
Cryptococcus neoformans var. neoformans B 3501A
Phanerochaete chrysosporium
Schizophyllum commune H4 8
Coprinopsis cinerea okayama7_130
Laccaria bicolor S238N H82
Schizosaccharomyces japonicus yFS275
Schizosaccharomyces pombe
Clavispora lusitaniae ATCC 42720
Yarrowia lipolytica
Candida tropicalis MYA 3404
Candida dubliniensis CD36
Lodderomyces elongisporus NRRL YB 4239
Scheffersomyces stipitis CBS 6054
Meyerozyma guilliermondii ATCC 6260
Pichia guilliermondii
Lachancea thermotolerans
Kluyveromyces lactis NRRL Y 1140
Saccharomyces cerevisiae
Zygosaccharomyces rouxii
Komagataella pastoris GS115
Vanderwaltozyma polyspora DSM 70294
Torulaspora delbrueckii
Candida glabrata CBS 138
Tetrapisispora phaffii CBS 4417
Naumovozyma dairenensis CBS 421
Naumovozyma castellii CBS 4309
Eremothecium cymbalariae DBVPG_7215
Eremothecium gossypii
Tuber melanosporum
Zymoseptoria tritici
Phaeosphaeria nodorum
Leptosphaeria maculans
Pyrenophora tritici repentis Pt 1C BFP
Pyrenophora teres f. teres 0 1
Botryotinia fuckeliana
Sclerotinia sclerotiorum
Verticillium albo atrum VaMs.102
Fusarium oxysporum
Hypocrea virens
Hypocrea jecorina
Nectria haematococca
Gibberella moniliformis
Gibberella zeae PH 1
Gibberella zeae
Gaeumannomyces graminis
Magnaporthe grisea
Magnaporthe poae
Magnaporthe oryzae
Podospora anserina S mat
Neurospora crassa
Sordaria macrospora k hell
Myceliophthora thermophila ATCC 42464
Chaetomium globosum CBS 148.51
Thielavia terrestris NRRL 8126
Uncinocarpus reesii 1704
Ajellomyces capsulatus NAm1
Ajellomyces dermatitidis SLH14081
Paracoccidioides sp. lutzii Pb01
Coccidioides posadasii C735 delta SOWgp
Coccidioides immitis RS
Trichophyton rubrum CBS 118892
Trichophyton verrucosum HKI 0517
Arthroderma benhamiae CBS 112371
Arthroderma gypseum CBS 118893
Arthroderma otae CBS 113480
Talaromyces stipitatus ATCC 10500
Neosartorya fischeri
Emericella nidulans
Penicillium chrysogenum Wisconsin 54 1255
Penicillium marneffei ATCC 18224
Aspergillus clavatus
Aspergillus flavus
Aspergillus oryzae
Aspergillus niger
Aspergillus fumigatus
Aspergillus terreus
Monosiga brevicollis MX1
Amphimedon queenslandica
Trichoplax adhaerens
Nematostella vectensis
Hydra magnipapillata
Schistosoma mansoni
Trichinella spiralis
Pristionchus pacificus
Caenorhabditis brenneri
Caenorhabditis briggsae
Caenorhabditis remanei
Caenorhabditis japonica
Caenorhabditis elegans
Ixodes scapularis
Daphnia pulex
Pediculus humanus
Acyrthosiphon pisum
Tribolium castaneum
Nasonia vitripennis
Apis mellifera
Atta cephalotes
Bombyx mori
Danaus plexippus
Heliconius melpomene
Culex quinquefasciatus
Aedes aegypti
Anopheles gambiae
Anopheles darlingi
Drosophila virilis
Drosophila grimshawi
Drosophila willistoni
Drosophila pseudoobscura
Drosophila ananassae
Drosophila melanogaster
Drosophila yakuba
Strongylocentrotus purpuratus
Branchiostoma floridae
Ciona intestinalis
Ciona savignyi
Petromyzon marinus
Danio rerio
Gadus morhua
Oreochromis niloticus
Takifugu rubripes
Tetraodon nigroviridis
Gasterosteus aculeatus
Xiphophorus maculatus
Oryzias latipes
Latimeria chalumnae
Xenopus (Silurana) tropicalis
Pelodiscus sinensis
Anolis carolinensis
Taeniopygia guttata
Gallus gallus
Meleagris gallopavo
Ornithorhynchus anatinus
Monodelphis domestica
Sarcophilus harrisii
Macropus eugenii
Dasypus novemcinctus
Loxodonta africana
Echinops telfairi
Procavia capensis
Myotis lucifugus
Pteropus vampyrus
Sus scrofa
Tursiops truncatus
Bos taurus
Equus caballus
Felis catus
Canis lupus familiaris
Mustela putorius furo
Ailuropoda melanoleuca
Oryctolagus cuniculus
Cavia porcellus
Ictidomys tridecemlineatus
Dipodomys ordii
Rattus norvegicus
Mus musculus
Tupaia belangeri
Otolemur garnettii
Microcebus murinus
Tarsius syrichta
Callithrix jacchus
Macaca mulatta
Nomascus leucogenys
Pongo abelii
Gorilla gorilla
Pan troglodytes
Homo sapiens
Perkinsus marinus ATCC 50983
Tetrahymena thermophila
Paramecium tetraurelia strain d4 2
Neospora caninum Liverpool
Toxoplasma gondii
Cryptosporidium muris RN66
Cryptosporidium parvum Iowa II
Cryptosporidium hominis TU502
Theileria annulata strain Ankara
Theileria parva strain Muguga
Plasmodium falciparum
Plasmodium knowlesi
Plasmodium vivax
Plasmodium chabaudi
Plasmodium yoelii yoelii 17XNL
Plasmodium berghei
Phaeodactylum tricornutum
Thalassiosira pseudonana
Pythium ultimum
Albugo laibachii
Hyaloperonospora arabidopsidis
Phytophthora ramorum
Phytophthora infestans
Phytophthora sojae
Trypanosoma brucei
Trypanosoma cruzi strain CL Brener
Leishmania braziliensis MHOM BR 75 M2904
Leishmania mexicana MHOM GT 2001 U1103
Leishmania major
Leishmania donovani
Leishmania infantum JPCM5
Entamoeba histolytica
Entamoeba dispar SAW760
Dictyostelium discoideum
Dictyostelium purpureum
Trichomonas vaginalis G3
Trichomonas vaginalis
Naegleria gruberi
Giardia lamblia ATCC 50803
OG presence
OG absence
Inferred root
Dscore = 0.807
Pvalue = 1.05e−04
AdjPvalue = 1.25e−02

NOG06748

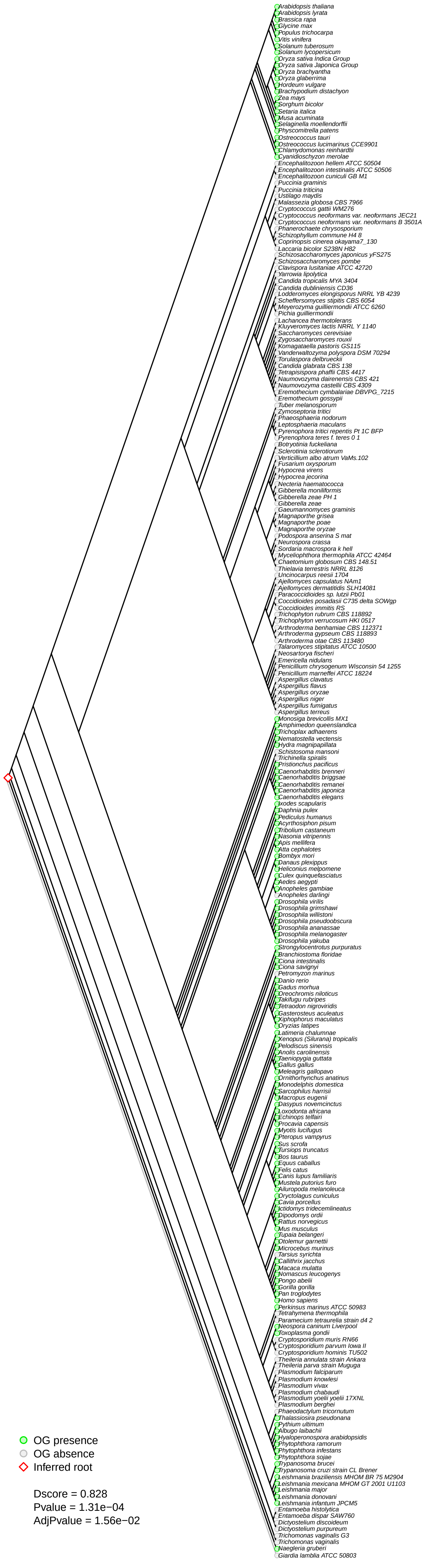

Arabidopsis thaliana
Arabidopsis lyrata
Brassica rapa
Glycine max
Populus trichocarpa
Vitis vinifera
Solanum tuberosum
Solanum lycopersicum
Oryza sativa Indica Group
Oryza sativa Japonica Group
Oryza brachyantha
Oryza glaberrima
Hordeum vulgare
Brachypodium distachyon
Zea mays
Sorghum bicolor
Setaria italica
Musa acuminata
Selaginella moellendorffii
Physcomitrella patens
Ostreococcus tauri
Ostreococcus lucimarinus CCE9901
Chlamydomonas reinhardtii
Cyanidioschyzon merolae
Encephalitozoon hellem ATCC 50504
Encephalitozoon intestinalis ATCC 50506
Encephalitozoon cuniculi GB M1
Puccinia graminis
Puccinia triticina
Ustilago maydis
Malassezia globosa CBS 7966
Cryptococcus gattii WM276
Cryptococcus neoformans var. neoformans JEC21
Cryptococcus neoformans var. neoformans B 3501A
Phanerochaete chrysosporium
Schizophyllum commune H4 8
Coprinopsis cinerea okayama7_130
Laccaria bicolor S238N H82
Schizosaccharomyces japonicus yFS275
Schizosaccharomyces pombe
Clavispora lusitaniae ATCC 42720
Yarrowia lipolytica
Candida tropicalis MYA 3404
Candida dubliniensis CD36
Lodderomyces elongisporus NRRL YB 4239
Scheffersomyces stipitis CBS 6054
Meyerozyma guilliermondii ATCC 6260
Pichia guilliermondii
Lachancea thermotolerans
Kluyveromyces lactis NRRL Y 1140
Saccharomyces cerevisiae
Zygosaccharomyces rouxii
Komagataella pastoris GS115
Vanderwaltozyma polyspora DSM 70294
Torulaspora delbrueckii
Candida glabrata CBS 138
Tetrapisispora phaffii CBS 4417
Naumovozyma dairenensis CBS 421
Naumovozyma castellii CBS 4309
Eremothecium cymbalariae DBVPG_7215
Eremothecium gossypii
Tuber melanosporum
Zymoseptoria tritici
Phaeosphaeria nodorum
Leptosphaeria maculans
Pyrenophora tritici repentis Pt 1C BFP
Pyrenophora teres f. teres 0 1
Botryotinia fuckeliana
Sclerotinia sclerotiorum
Verticillium albo atrum VaMs.102
Fusarium oxysporum
Hypocrea virens
Hypocrea jecorina
Necteria haematococca
Gibberella moniliformis
Gibberella zeae PH 1
Gibberella zeae
Gaeumannomyces graminis
Magnaporthe grisea
Magnaporthe poae
Magnaporthe oryzae
Podospora anserina S mat
Neurospora crassa
Sordaria macrospora k hell
Myceliophthora thermophila ATCC 42464
Chaetomium globosum CBS 148.51
Thielavia terrestris NRRL 8126
Uncinocarpus reesii 1704
Ajellomyces capsulatus NAm1
Ajellomyces dermatitidis SLH14081
Paracoccidioides sp. lutzii Pb01
Coccidioides posadasii C735 delta SOWgp
Coccidioides immitis RS
Trichophyton rubrum CBS 118892
Trichophyton verrucosum HKI 0517
Arthroderma benhamiae CBS 112371
Arthroderma gypseum CBS 118893
Arthroderma otae CBS 113480
Talaromyces stipitatus ATCC 10500
Neosartorya fischeri
Emericella nidulans
Penicillium chrysogenum Wisconsin 54 1255
Penicillium marneffei ATCC 18224
Aspergillus clavatus
Aspergillus flavus
Aspergillus oryzae
Aspergillus niger
Aspergillus fumigatus
Aspergillus terreus
Monosiga brevicollis MX1
Amphimedon queenslandica
Trichoplax adhaerens
Nematostella vectensis
Hydra magnipapillata
Schistosoma mansoni
Trichinella spiralis
Pristionchus pacificus
Caenorhabditis brenneri
Caenorhabditis briggsae
Caenorhabditis remanei
Caenorhabditis japonica
Caenorhabditis elegans
Ixodes scapularis
Daphnia pulex
Pediculus humanus
Acyrthosiphon pisum
Tribolium castaneum
Nasonia vitripennis
Apis mellifera
Atta cephalotes
Bombyx mori
Danaus plexippus
Heliconius melpomene
Culex quinquefasciatus
Aedes aegypti
Anopheles gambiae
Anopheles darlingi
Drosophila virilis
Drosophila grimshawi
Drosophila willistoni
Drosophila pseudoobscura
Drosophila ananassae
Drosophila melanogaster
Drosophila yakuba
Strongylocentrotus purpuratus
Branchiostoma floridae
Ciona intestinalis
Ciona savignyi
Petromyzon marinus
Danio rerio
Gadus morhua
Oreochromis niloticus
Takifugu rubripes
Tetraodon nigroviridis
Gasterosteus aculeatus
Xiphophorus maculatus
Oryzias latipes
Latimeria chalumnae
Xenopus (Silurana) tropicalis
Pelodiscus sinensis
Anolis carolinensis
Taeniopygia guttata
Gallus gallus
Meleagris gallopavo
Ornithorhynchus anatinus
Monodelphis domestica
Sarcophilus harrisii
Macropus eugenii
Dasypus novemcinctus
Loxodonta africana
Echinops telfairi
Procavia capensis
Myotis lucifugus
Pteropus vampyrus
Sus scrofa
Tursiops truncatus
Bos taurus
Equus caballus
Felis catus
Canis lupus familiaris
Mustela putorius furo
Ailuropoda melanoleuca
Oryctolagus cuniculus
Cavia porcellus
Ictidomys tridecemlineatus
Dipodomys ordii
Rattus norvegicus
Mus musculus
Tupaia belangeri
Otolemur garnettii
Microcebus murinus
Tarsius syrichta
Callithrix jacchus
Macaca mulatta
Nomascus leucogenys
Pongo abelii
Gorilla gorilla
Pan troglodytes
Homo sapiens
Perkinsus marinus ATCC 50983
Tetrahymena thermophila
Paramecium tetraurelia strain d4 2
Neospora caninum Liverpool
Toxoplasma gondii
Cryptosporidium muris RN66
Cryptosporidium parvum Iowa II
Cryptosporidium hominis TU502
Theileria annulata strain Ankara
Theileria parva strain Muguga
Plasmodium falciparum
Plasmodium knowlesi
Plasmodium vivax
Plasmodium chabaudi
Plasmodium yoelii yoelii 17XNL
Plasmodium berghei
Phaeodactylum tricornutum
Thalassiosira pseudonana
Pythium ultimum
Albugo laibachii
Hyaloperonospora arabidopsidis
Phytophthora ramorum
Phytophthora infestans
Phytophthora sojae
Trypanosoma brucei
Trypanosoma cruzi strain CL Brener
Leishmania braziliensis MHOM BR 75 M2904
Leishmania mexicana MHOM GT 2001 U1103
Leishmania major
Leishmania donovani
Leishmania infantum JPCM5
Entamoeba histolytica
Entamoeba dispar SAW760
Dictyostelium discoideum
Dictyostelium purpureum
Trichomonas vaginalis G3
Trichomonas vaginalis
Naegleria gruberi
Giardia lamblia ATCC 50803
OG presence
OG absence
Inferred root
Dscore = 0.828
Pvalue = 1.31e−04
AdjPvalue = 1.56e−02

NOG07211

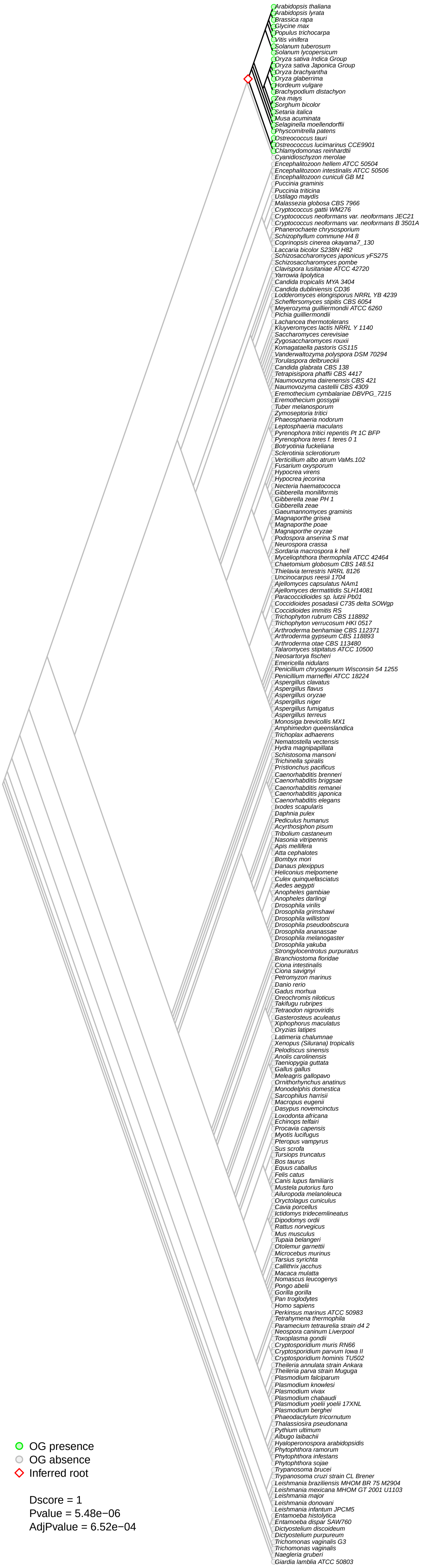
Arabidopsis thaliana
Arabidopsis lyrata
Brassica rapa
Glycine max
Populus trichocarpa
Vitis vinifera
Solanum tuberosum
Solanum lycopersicum
Oryza sativa Indica Group
Oryza sativa Japonica Group
Oryza brachyantha
Oryza glaberrima
Hordeum vulgare
Brachypodium distachyon
Zea mays
Sorghum bicolor
Setaria italica
Musa acuminata
Selaginella moellendorffii
Physcomitrella patens
Ostreococcus tauri
Ostreococcus lucimarinus CCE9901
Chlamydomonas reinhardtii
Cyanidioschyzon merolae
Encephalitozoon hellem ATCC 50504
Encephalitozoon intestinalis ATCC 50506
Encephalitozoon cuniculi GB M1
Puccinia graminis
Puccinia triticina
Ustilago maydis
Malassezia globosa CBS 7966
Cryptococcus gattii WM276
Cryptococcus neoformans var. neoformans JEC21
Cryptococcus neoformans var. neoformans B 3501A
Phanerochaete chrysosporium
Schizophyllum commune H4 8
Coprinopsis cinerea okayama7_130
Laccaria bicolor S238N H82
Schizosaccharomyces japonicus yFS275
Schizosaccharomyces pombe
Clavispora lusitaniae ATCC 42720
Yarrowia lipolytica
Candida tropicalis MYA 3404
Candida dubliniensis CD36
Lodderomyces elongisporus NRRL YB 4239
Scheffersomyces stipitis CBS 6054
Meyerozyma guilliermondii ATCC 6260
Pichia guilliermondii
Lachancea thermotolerans
Kluyveromyces lactis NRRL Y 1140
Saccharomyces cerevisiae
Zygosaccharomyces rouxii
Komagataella pastoris GS115
Vanderwaltozyma polyspora DSM 70294
Torulaspora delbrueckii
Candida glabrata CBS 138
Tetrapisispora phaffii CBS 4417
Naumovozyma dairenensis CBS 421
Naumovozyma castellii CBS 4309
Eremothecium cymbalariae DBVPG_7215
Eremothecium gossypii
Tuber melanosporum
Zymoseptoria tritici
Phaeosphaeria nodorum
Leptosphaeria maculans
Pyrenophora tritici repentis Pt 1C BFP
Pyrenophora teres f. teres 0 1
Botryotinia fuckeliana
Sclerotinia sclerotiorum
Verticillium albo atrum VaMs.102
Fusarium oxysporum
Hypocrea virens
Hypocrea jecorina
Necteria haematococca
Gibberella moniliformis
Gibberella zeae PH 1
Gibberella zeae
Gaeumannomyces graminis
Magnaporthe grisea
Magnaporthe poae
Magnaporthe oryzae
Podospora anserina S mat
Neurospora crassa
Sordaria macrospora k hell
Myceliophthora thermophila ATCC 42464
Chaetomium globosum CBS 148.51
Thielavia terrestris NRRL 8126
Uncinocarpus reesii 1704
Ajellomyces capsulatus NAm1
Ajellomyces dermatitidis SLH14081
Paracoccidioides sp. lutzii Pb01
Coccidioides posadasii C735 delta SOWgp
Coccidioides immitis RS
Trichophyton rubrum CBS 118892
Trichophyton verrucosum HKI 0517
Arthroderma benhamiae CBS 112371
Arthroderma gypseum CBS 118893
Arthroderma otae CBS 113480
Talaromyces stipitatus ATCC 10500
Neosartorya fischeri
Emericella nidulans
Penicillium chrysogenum Wisconsin 54 1255
Penicillium marneffei ATCC 18224
Aspergillus clavatus
Aspergillus flavus
Aspergillus oryzae
Aspergillus niger
Aspergillus fumigatus
Aspergillus terreus
Monosiga brevicollis MX1
Amphimedon queenslandica
Trichoplax adhaerens
Nematostella vectensis
Hydra magnipapillata
Schistosoma mansoni
Trichinella spiralis
Pristionchus pacificus
Caenorhabditis brenneri
Caenorhabditis briggsae
Caenorhabditis remanei
Caenorhabditis japonica
Caenorhabditis elegans
Ixodes scapularis
Daphnia pulex
Pediculus humanus
Acyrthosiphon pisum
Tribolium castaneum
Nasonia vitripennis
Apis mellifera
Atta cephalotes
Bombyx mori
Danaus plexippus
Heliconius melpomene
Culex quinquefasciatus
Aedes aegypti
Anopheles gambiae
Anopheles darlingi
Drosophila virilis
Drosophila grimshawi
Drosophila willistoni
Drosophila pseudoobscura
Drosophila ananassae
Drosophila melanogaster
Drosophila yakuba
Strongylocentrotus purpuratus
Branchiostoma floridae
Ciona intestinalis
Ciona savignyi
Petromyzon marinus
Danio rerio
Gadus morhua
Oreochromis niloticus
Takifugu rubripes
Tetraodon nigroviridis
Gasterosteus aculeatus
Xiphophorus maculatus
Oryzias latipes
Latimeria chalumnae
Xenopus (Silurana) tropicalis
Pelodiscus sinensis
Anolis carolinensis
Taeniopygia guttata
Gallus gallus
Meleagris gallopavo
Ornithorhynchus anatinus
Monodelphis domestica
Sarcophilus harrisii
Macropus eugenii
Dasypus novemcinctus
Loxodonta africana
Echinops telfairi
Procavia capensis
Myotis lucifugus
Pteropus vampyrus
Sus scrofa
Tursiops truncatus
Bos taurus
Equus caballus
Felis catus
Canis lupus familiaris
Mustela putorius furo
Ailuropoda melanoleuca
Oryctolagus cuniculus
Cavia porcellus
Ictidomys tridecemlineatus
Dipodomys ordii
Rattus norvegicus
Mus musculus
Tupaia belangeri
Otolemur garnettii
Microcebus murinus
Tarsius syrichta
Callithrix jacchus
Macaca mulatta
Nomascus leucogenys
Pongo abelii
Gorilla gorilla
Pan troglodytes
Homo sapiens
Perkinsus marinus ATCC 50983
Tetrahymena thermophila
Paramecium tetraurelia strain d4 2
Neospora caninum Liverpool
Toxoplasma gondii
Cryptosporidium muris RN66
Cryptosporidium parvum Iowa II
Cryptosporidium hominis TU502
Theileria annulata strain Ankara
Theileria parva strain Muguga
Plasmodium falciparum
Plasmodium knowlesi
Plasmodium vivax
Plasmodium chabaudi
Plasmodium yoelii yoelii 17XNL
Plasmodium berghei
Phaeodactylum tricornutum
Thalassiosira pseudonana
Pythium ultimum
Albugo laibachii
Hyaloperonospora arabidopsidis
Phytophthora ramorum
Phytophthora infestans
Phytophthora sojae
Trypanosoma brucei
Trypanosoma cruzi strain CL Brener
Leishmania braziliensis MHOM BR 75 M2904
Leishmania mexicana MHOM GT 2001 U1103
Leishmania major
Leishmania donovani
Leishmania infantum JPCM5
Entamoeba histolytica
Entamoeba dispar SAW760
Dictyostelium discoideum
Dictyostelium purpureum
Trichomonas vaginalis G3
Trichomonas vaginalis
Naegleria gruberi
Giardia lamblia ATCC 50803
OG presence
OG absence
Inferred root
Dscore = 1
Pvalue = 5.48e−06
AdjPvalue = 6.52e−04

NOG07766

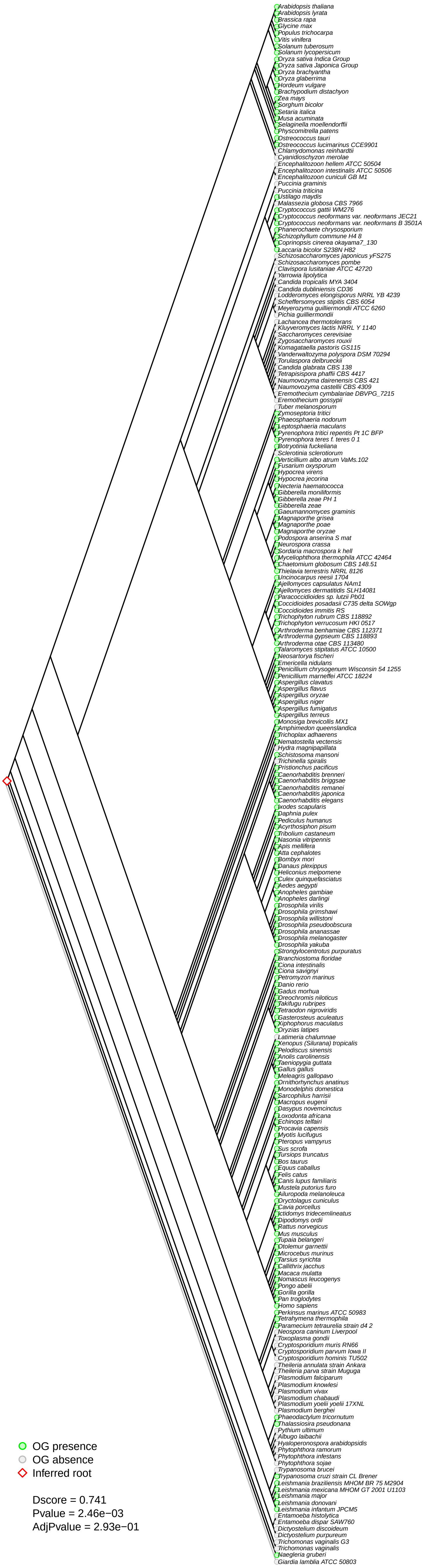

Arabidopsis thaliana
Arabidopsis lyrata
Brassica rapa
Glycine max
Populus trichocarpa
Vitis vinifera
Solanum tuberosum
Solanum lycopersicum
Oryza sativa Indica Group
Oryza sativa Japonica Group
Oryza brachyantha
Oryza glaberrima
Hordeum vulgare
Brachypodium distachyon
Zea mays
Sorghum bicolor
Setaria italica
Musa acuminata
Selaginella moellendorffii
Physcomitrella patens
Ostreococcus tauri
Ostreococcus lucimarinus CCE9901
Chlamydomonas reinhardtii
Cyanidioschyzon merolae
Encephalitozoon hellem ATCC 50504
Encephalitozoon intestinalis ATCC 50506
Encephalitozoon cuniculi GB M1
Puccinia graminis
Puccinia triticina
Ustilago maydis
Malassezia globosa CBS 7966
Cryptococcus gattii WM276
Cryptococcus neoformans var. neoformans JEC21
Cryptococcus neoformans var. neoformans B 3501A
Phanerochaete chrysosporium
Schizophyllum commune H4 8
Coprinopsis cinerea okayama7_130
Laccaria bicolor S238N H82
Schizosaccharomyces japonicus yFS275
Schizosaccharomyces pombe
Clavispora lusitaniae ATCC 42720
Yarrowia lipolytica
Candida tropicalis MYA 3404
Candida dubliniensis CD36
Lodderomyces elongisporus NRRL YB 4239
Scheffersomyces stipitis CBS 6054
Meyerozyma guilliermondii ATCC 6260
Pichia guilliermondii
Lachancea thermotolerans
Kluyveromyces lactis NRRL Y 1140
Saccharomyces cerevisiae
Zygosaccharomyces rouxii
Komagataella pastoris GS115
Vanderwaltozyma polyspora DSM 70294
Torulaspora delbrueckii
Candida glabrata CBS 138
Tetrapisispora phaffii CBS 4417
Naumovozyma dairenensis CBS 421
Naumovozyma castellii CBS 4309
Eremothecium cymbalariae DBVPG_7215
Eremothecium gossypii
Tuber melanosporum
Zymoseptoria tritici
Phaeosphaeria nodorum
Leptosphaeria maculans
Pyrenophora tritici repentis Pt 1C BFP
Pyrenophora teres f. teres 0 1
Botryotinia fuckeliana
Sclerotinia sclerotiorum
Verticillium albo atrum VaMs.102
Fusarium oxysporum
Hypocrea virens
Hypocrea jecorina
Nectria haematococca
Gibberella moniliformis
Gibberella zeae PH 1
Gibberella zeae
Gaeumannomyces graminis
Magnaporthe grisea
Magnaporthe poae
Magnaporthe oryzae
Podospora anserina S mat
Neurospora crassa
Sordaria macrospora k hell
Myceliophthora thermophila ATCC 42464
Chaetomium globosum CBS 148.51
Thielavia terrestris NRRL 8126
Uncinocarpus reesii 1704
Ajellomyces capsulatus NAm1
Ajellomyces dermatitidis SLH14081
Paracoccidioides sp. lutzii Pb01
Coccidioides posadasii C735 delta SOWgp
Coccidioides immitis RS
Trichophyton rubrum CBS 118892
Trichophyton verrucosum HKI 0517
Arthroderma benhamiae CBS 112371
Arthroderma gypseum CBS 118893
Arthroderma otae CBS 113480
Talaromyces stipitatus ATCC 10500
Neosartorya fischeri
Emericella nidulans
Penicillium chrysogenum Wisconsin 54 1255
Penicillium marneffei ATCC 18224
Aspergillus clavatus
Aspergillus flavus
Aspergillus oryzae
Aspergillus niger
Aspergillus fumigatus
Aspergillus terreus
Monosiga brevicollis MX1
Amphimedon queenslandica
Trichoplax adhaerens
Nematostella vectensis
Hydra magnipapillata
Schistosoma mansoni
Trichinella spiralis
Pristionchus pacificus
Caenorhabditis brenneri
Caenorhabditis briggsae
Caenorhabditis remanei
Caenorhabditis japonica
Caenorhabditis elegans
Ixodes scapularis
Daphnia pulex
Pediculus humanus
Acyrthosiphon pisum
Tribolium castaneum
Nasonia vitripennis
Apis mellifera
Atta cephalotes
Bombyx mori
Danaus plexippus
Heliconius melpomene
Culex quinquefasciatus
Aedes aegypti
Anopheles gambiae
Anopheles darlingi
Drosophila virilis
Drosophila grimshawi
Drosophila willistoni
Drosophila pseudoobscura
Drosophila ananassae
Drosophila melanogaster
Drosophila yakuba
Strongylocentrotus purpuratus
Branchiostoma floridae
Ciona intestinalis
Ciona savignyi
Petromyzon marinus
Danio rerio
Gadus morhua
Oreochromis niloticus
Takifugu rubripes
Tetraodon nigroviridis
Gasterosteus aculeatus
Xiphophorus maculatus
Oryzias latipes
Latimeria chalumnae
Xenopus (Silurana) tropicalis
Pelodiscus sinensis
Anolis carolinensis
Taeniopygia guttata
Gallus gallus
Meleagris gallopavo
Ornithorhynchus anatinus
Monodelphis domestica
Sarcophilus harrisii
Macropus eugenii
Dasypus novemcinctus
Loxodonta africana
Echinops telfairi
Procavia capensis
Myotis lucifugus
Pteropus vampyrus
Sus scrofa
Tursiops truncatus
Bos taurus
Equus caballus
Felis catus
Canis lupus familiaris
Mustela putorius furo
Ailuropoda melanoleuca
Oryctolagus cuniculus
Cavia porcellus
Ictidomys tridecemlineatus
Dipodomys ordii
Rattus norvegicus
Mus musculus
Tupaia belangeri
Otolemur garnettii
Microcebus murinus
Tarsius syrichta
Callithrix jacchus
Macaca mulatta
Nomascus leucogenys
Pongo abelii
Gorilla gorilla
Pan troglodytes
Homo sapiens
Perkinsus marinus ATCC 50983
Tetrahymena thermophila
Paramecium tetraurelia strain d4 2
Neospora caninum Liverpool
Toxoplasma gondii
Cryptosporidium muris RN66
Cryptosporidium parvum Iowa II
Cryptosporidium hominis TU502
Theileria annulata strain Ankara
Theileria parva strain Muguga
Plasmodium falciparum
Plasmodium knowlesi
Plasmodium vivax
Plasmodium chabaudi
Plasmodium yoelii yoelii 17XNL
Plasmodium berghei
Phaeodactylum tricornutum
Thalassiosira pseudonana
Pythium ultimum
Albugo laibachii
Hyaloperonospora arabidopsidis
Phytophthora ramorum
Phytophthora infestans
Phytophthora sojae
Trypanosoma brucei
Trypanosoma cruzi strain CL Brener
Leishmania braziliensis MHOM BR 75 M2904
Leishmania mexicana MHOM GT 2001 U1103
Leishmania major
Leishmania donovani
Leishmania infantum JPCM5
Entamoeba histolytica
Entamoeba dispar SAW760
Dictyostelium discoideum
Dictyostelium purpureum
Trichomonas vaginalis G3
Trichomonas vaginalis
Naegleria gruberi
Giardia lamblia ATCC 50803
OG presence
OG absence
Inferred root
Dscore = 0.741
Pvalue = 2.46e−03
AdjPvalue = 2.93e−01

NOG101992

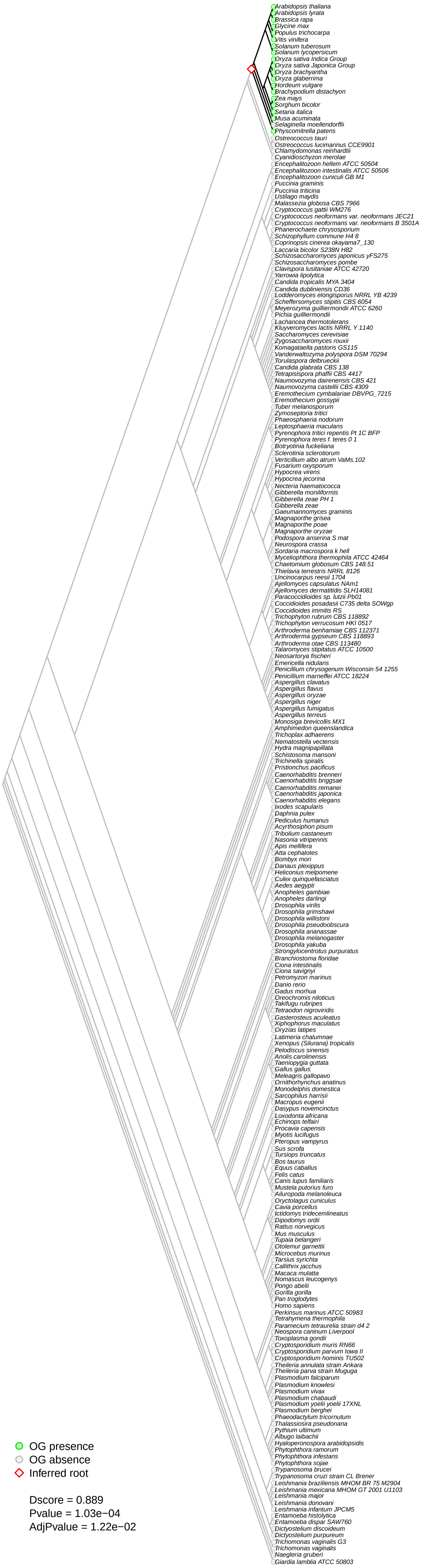
Arabidopsis thaliana
Arabidopsis lyrata
Brassica rapa
Glycine max
Populus trichocarpa
Vitis vinifera
Solanum tuberosum
Solanum lycopersicum
Oryza sativa Indica Group
Oryza sativa Japonica Group
Oryza brachyantha
Oryza glaberrima
Hordeum vulgare
Brachypodium distachyon
Zea mays
Sorghum bicolor
Setaria italica
Musa acuminata
Selaginella moellendorffii
Physcomitrella patens
Ostreococcus tauri
Ostreococcus lucimarinus CCE9901
Chlamydomonas reinhardtii
Cyanidioschyzon merolae
Encephalitozoon hellem ATCC 50504
Encephalitozoon intestinalis ATCC 50506
Encephalitozoon cuniculi GB M1
Puccinia graminis
Puccinia triticina
Ustilago maydis
Malassezia globosa CBS 7966
Cryptococcus gattii WM276
Cryptococcus neoformans var. neoformans JEC21
Cryptococcus neoformans var. neoformans B 3501A
Phanerochaete chrysosporium
Schizophyllum commune H4 8
Coprinopsis cinerea okayama7_130
Laccaria bicolor S238N H82
Schizosaccharomyces japonicus yFS275
Schizosaccharomyces pombe
Clavispora lusitaniae ATCC 42720
Yarrowia lipolytica
Candida tropicalis MYA 3404
Candida dubliniensis CD36
Lodderomyces elongisporus NRRL YB 4239
Scheffersomyces stipitis CBS 6054
Meyerozyma guilliermondii ATCC 6260
Pichia guilliermondii
Lachancea thermotolerans
Kluyveromyces lactis NRRL Y 1140
Saccharomyces cerevisiae
Zygosaccharomyces rouxii
Komagataella pastoris GS115
Vanderwaltozyma polyspora DSM 70294
Torulaspora delbrueckii
Candida glabrata CBS 138
Tetrapisispora phaffii CBS 4417
Naumovozyma dairenensis CBS 421
Naumovozyma castellii CBS 4309
Eremothecium cymbalariae DBVPG_7215
Eremothecium gossypii
Tuber melanosporum
Zymoseptoria tritici
Phaeosphaeria nodorum
Leptosphaeria maculans
Pyrenophora tritici repentis Pt 1C BFP
Pyrenophora teres f. teres 0 1
Botryotinia fuckeliana
Sclerotinia sclerotiorum
Verticillium albo atrum VaMs.102
Fusarium oxysporum
Hypocrea virens
Hypocrea jecorina
Nectria haematococca
Gibberella moniliformis
Gibberella zeae PH 1
Gibberella zeae
Gaeumannomyces graminis
Magnaporthe grisea
Magnaporthe poae
Magnaporthe oryzae
Podospora anserina S mat
Neurospora crassa
Sordaria macrospora k hell
Myceliophthora thermophila ATCC 42464
Chaetomium globosum CBS 148.51
Thielavia terrestris NRRL 8126
Uncinocarpus reesii 1704
Ajellomyces capsulatus NAm1
Ajellomyces dermatitidis SLH14081
Paracoccidioides sp. lutzii Pb01
Coccidioides posadasii C735 delta SOWgp
Coccidioides immitis RS
Trichophyton rubrum CBS 118892
Trichophyton verrucosum HKI 0517
Arthroderma benhamiae CBS 112371
Arthroderma gypseum CBS 118893
Arthroderma otae CBS 113480
Talaromyces stipitatus ATCC 10500
Neosartorya fischeri
Emericella nidulans
Penicillium chrysogenum Wisconsin 54 1255
Penicillium marneffei ATCC 18224
Aspergillus clavatus
Aspergillus flavus
Aspergillus oryzae
Aspergillus niger
Aspergillus fumigatus
Aspergillus terreus
Monosiga brevicollis MX1
Amphimedon queenslandica
Trichoplax adhaerens
Nematostella vectensis
Hydra magnipapillata
Schistosoma mansoni
Trichinella spiralis
Pristionchus pacificus
Caenorhabditis brenneri
Caenorhabditis briggsae
Caenorhabditis remanei
Caenorhabditis japonica
Caenorhabditis elegans
Ixodes scapularis
Daphnia pulex
Pediculus humanus
Acyrthosiphon pisum
Tribolium castaneum
Nasonia vitripennis
Apis mellifera
Atta cephalotes
Bombyx mori
Danaus plexippus
Heliconius melpomene
Culex quinquefasciatus
Aedes aegypti
Anopheles gambiae
Anopheles darlingi
Drosophila virilis
Drosophila grimshawi
Drosophila willistoni
Drosophila pseudoobscura
Drosophila ananassae
Drosophila melanogaster
Drosophila yakuba
Strongylocentrotus purpuratus
Branchiostoma floridae
Ciona intestinalis
Ciona savignyi
Petromyzon marinus
Danio rerio
Gadus morhua
Oreochromis niloticus
Takifugu rubripes
Tetraodon nigroviridis
Gasterosteus aculeatus
Xiphophorus maculatus
Oryzias latipes
Latimeria chalumnae
Xenopus (Silurana) tropicalis
Pelodiscus sinensis
Anolis carolinensis
Taeniopygia guttata
Gallus gallus
Meleagris gallopavo
Ornithorhynchus anatinus
Monodelphis domestica
Sarcophilus harrisii
Macropus eugenii
Dasypus novemcinctus
Loxodonta africana
Echinops telfairi
Procavia capensis
Myotis lucifugus
Pteropus vampyrus
Sus scrofa
Tursiops truncatus
Bos taurus
Equus caballus
Felis catus
Canis lupus familiaris
Mustela putorius furo
Ailuropoda melanoleuca
Oryctolagus cuniculus
Cavia porcellus
Ictidomys tridecemlineatus
Dipodomys ordii
Rattus norvegicus
Mus musculus
Tupaia belangeri
Otolemur garnettii
Microcebus murinus
Tarsius syrichta
Callithrix jacchus
Macaca mulatta
Nomascus leucogenys
Pongo abelii
Gorilla gorilla
Pan troglodytes
Homo sapiens
Perkinsus marinus ATCC 50983
Tetrahymena thermophila
Paramecium tetraurelia strain d4 2
Neospora caninum Liverpool
Toxoplasma gondii
Cryptosporidium muris RN66
Cryptosporidium parvum Iowa II
Cryptosporidium hominis TU502
Theileria annulata strain Ankara
Theileria parva strain Muguga
Plasmodium falciparum
Plasmodium knowlesi
Plasmodium vivax
Plasmodium chabaudi
Plasmodium yoelii yoelii 17XNL
Plasmodium berghei
Phaeodactylum tricornutum
Thalassiosira pseudonana
Pythium ultimum
Albugo laibachii
Hyaloperonospora arabidopsidis
Phytophthora ramorum
Phytophthora infestans
Phytophthora sojae
Trypanosoma brucei
Trypanosoma cruzi strain CL Brener
Leishmania braziliensis MHOM BR 75 M2904
Leishmania mexicana MHOM GT 2001 U1103
Leishmania major
Leishmania donovani
Leishmania infantum JPCM5
Entamoeba histolytica
Entamoeba dispar SAW760
Dictyostelium discoideum
Dictyostelium purpureum
Trichomonas vaginalis G3
Trichomonas vaginalis
Naegleria gruberi
Giardia lamblia ATCC 50803
OG presence
OG absence
Inferred root
Dscore = 0.889
Pvalue = 1.03e−04
AdjPvalue = 1.22e−02

NOG10297

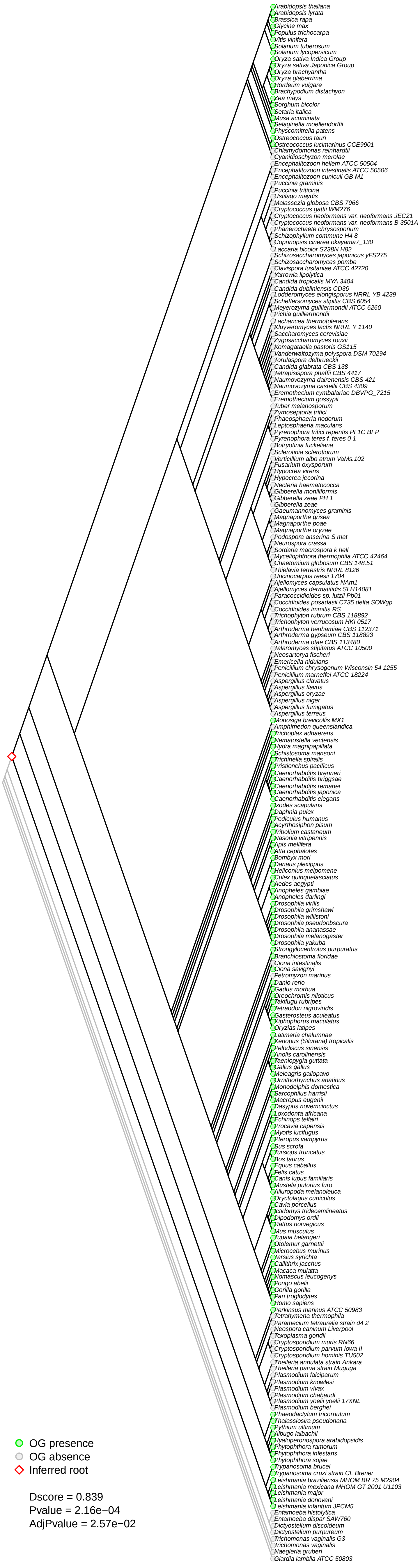

Arabidopsis thaliana
Arabidopsis lyrata
Brassica rapa
Glycine max
Populus trichocarpa
Vitis vinifera
Solanum tuberosum
Solanum lycopersicum
Oryza sativa Indica Group
Oryza sativa Japonica Group
Oryza brachyantha
Oryza glaberrima
Hordeum vulgare
Brachypodium distachyon
Zea mays
Sorghum bicolor
Setaria italica
Musa acuminata
Selaginella moellendorffii
Physcomitrella patens
Ostreococcus tauri
Ostreococcus lucimarinus CCE9901
Chlamydomonas reinhardtii
Cyanidioschyzon merolae
Encephalitozoon hellem ATCC 50504
Encephalitozoon intestinalis ATCC 50506
Encephalitozoon cuniculi GB M1
Puccinia graminis
Puccinia triticina
Ustilago maydis
Malassezia globosa CBS 7966
Cryptococcus gattii WM276
Cryptococcus neoformans var. neoformans JEC21
Cryptococcus neoformans var. neoformans B 3501A
Phanerochaete chrysosporium
Schizophyllum commune H4 8
Coprinopsis cinerea okayama7_130
Laccaria bicolor S238N H82
Schizosaccharomyces japonicus yFS275
Schizosaccharomyces pombe
Clavispora lusitaniae ATCC 42720
Yarrowia lipolytica
Candida tropicalis MYA 3404
Candida dubliniensis CD36
Lodderomyces elongisporus NRRL YB 4239
Scheffersomyces stipitis CBS 6054
Meyerozyma guilliermondii ATCC 6260
Pichia guilliermondii
Lachancea thermotolerans
Kluyveromyces lactis NRRL Y 1140
Saccharomyces cerevisiae
Zygosaccharomyces rouxii
Komagataella pastoris GS115
Vanderwaltozyma polyspora DSM 70294
Torulaspora delbrueckii
Candida glabrata CBS 138
Tetrapisispora phaffii CBS 4417
Naumovozyma dairenensis CBS 421
Naumovozyma castellii CBS 4309
Eremothecium cymbalariae DBVPG_7215
Eremothecium gossypii
Tuber melanosporum
Zymoseptoria tritici
Phaeosphaeria nodorum
Leptosphaeria maculans
Pyrenophora tritici repentis Pt 1C BFP
Pyrenophora teres f. teres 0 1
Botryotinia fuckeliana
Sclerotinia sclerotiorum
Verticillium albo atrum VaMs.102
Fusarium oxysporum
Hypocrea virens
Hypocrea jecorina
Necteria haematococca
Gibberella moniliformis
Gibberella zeae PH 1
Gibberella zeae
Gaeumannomyces graminis
Magnaporthe grisea
Magnaporthe poae
Magnaporthe oryzae
Podospora anserina S mat
Neurospora crassa
Sordaria macrospora k hell
Myceliophthora thermophila ATCC 42464
Chaetomium globosum CBS 148.51
Thielavia terrestris NRRL 8126
Uncinocarpus reesii 1704
Ajellomyces capsulatus NAm1
Ajellomyces dermatitidis SLH14081
Paracoccidioides sp. lutzii Pb01
Coccidioides posadasii C735 delta SOWgp
Coccidioides immitis RS
Trichophyton rubrum CBS 118892
Trichophyton verrucosum HKI 0517
Arthroderma benhamiae CBS 112371
Arthroderma gypseum CBS 118893
Arthroderma otae CBS 113480
Talaromyces stipitatus ATCC 10500
Neosartorya fischeri
Emericella nidulans
Penicillium chrysogenum Wisconsin 54 1255
Penicillium marneffei ATCC 18224
Aspergillus clavatus
Aspergillus flavus
Aspergillus oryzae
Aspergillus niger
Aspergillus fumigatus
Aspergillus terreus
Monosiga brevicollis MX1
Amphimedon queenslandica
Trichoplax adhaerens
Nematostella vectensis
Hydra magnipapillata
Schistosoma mansoni
Trichinella spiralis
Pristionchus pacificus
Caenorhabditis brenneri
Caenorhabditis briggsae
Caenorhabditis remanei
Caenorhabditis japonica
Caenorhabditis elegans
Ixodes scapularis
Daphnia pulex
Pediculus humanus
Acyrthosiphon pisum
Tribolium castaneum
Nasonia vitripennis
Apis mellifera
Atta cephalotes
Bombyx mori
Danaus plexippus
Heliconius melpomene
Culex quinquefasciatus
Aedes aegypti
Anopheles gambiae
Anopheles darlingi
Drosophila virilis
Drosophila grimshawi
Drosophila willistoni
Drosophila pseudoobscura
Drosophila ananassae
Drosophila melanogaster
Drosophila yakuba
Strongylocentrotus purpuratus
Branchiostoma floridae
Ciona intestinalis
Ciona savignyi
Petromyzon marinus
Danio rerio
Gadus morhua
Oreochromis niloticus
Takifugu rubripes
Tetraodon nigroviridis
Gasterosteus aculeatus
Xiphophorus maculatus
Oryzias latipes
Latimeria chalumnae
Xenopus (Silurana) tropicalis
Pelodiscus sinensis
Anolis carolinensis
Taeniopygia guttata
Gallus gallus
Meleagris gallopavo
Ornithorhynchus anatinus
Monodelphis domestica
Sarcophilus harrisii
Macropus eugenii
Dasypus novemcinctus
Loxodonta africana
Echinops telfairi
Procavia capensis
Myotis lucifugus
Pteropus vampyrus
Sus scrofa
Tursiops truncatus
Bos taurus
Equus caballus
Felis catus
Canis lupus familiaris
Mustela putorius furo
Ailuropoda melanoleuca
Oryctolagus cuniculus
Cavia porcellus
Ictidomys tridecemlineatus
Dipodomys ordii
Rattus norvegicus
Mus musculus
Tupaia belangeri
Otolemur garnettii
Microcebus murinus
Tarsius syrichta
Callithrix jacchus
Macaca mulatta
Nomascus leucogenys
Pongo abelii
Gorilla gorilla
Pan troglodytes
Homo sapiens
Perkinsus marinus ATCC 50983
Tetrahymena thermophila
Paramecium tetraurelia strain d4 2
Neospora caninum Liverpool
Toxoplasma gondii
Cryptosporidium muris RN66
Cryptosporidium parvum Iowa II
Cryptosporidium hominis TU502
Theileria annulata strain Ankara
Theileria parva strain Muguga
Plasmodium falciparum
Plasmodium knowlesi
Plasmodium vivax
Plasmodium chabaudi
Plasmodium yoelii yoelii 17XNL
Plasmodium berghei
Phaeodactylum tricornutum
Thalassiosira pseudonana
Pythium ultimum
Albugo laibachii
Hyaloperonospora arabidopsidis
Phytophthora ramorum
Phytophthora infestans
Phytophthora sojae
Trypanosoma brucei
Trypanosoma cruzi strain CL Brener
Leishmania braziliensis MHOM BR 75 M2904
Leishmania mexicana MHOM GT 2001 U1103
Leishmania major
Leishmania donovani
Leishmania infantum JPCM5
Entamoeba histolytica
Entamoeba dispar SAW760
Dictyostelium discoideum
Dictyostelium purpureum
Trichomonas vaginalis G3
Trichomonas vaginalis
Naegleria gruberi
Giardia lamblia ATCC 50803
OG presence
OG absence
Inferred root
Dscore = 0.839
Pvalue = 2.16e−04
AdjPvalue = 2.57e−02

NOG11768

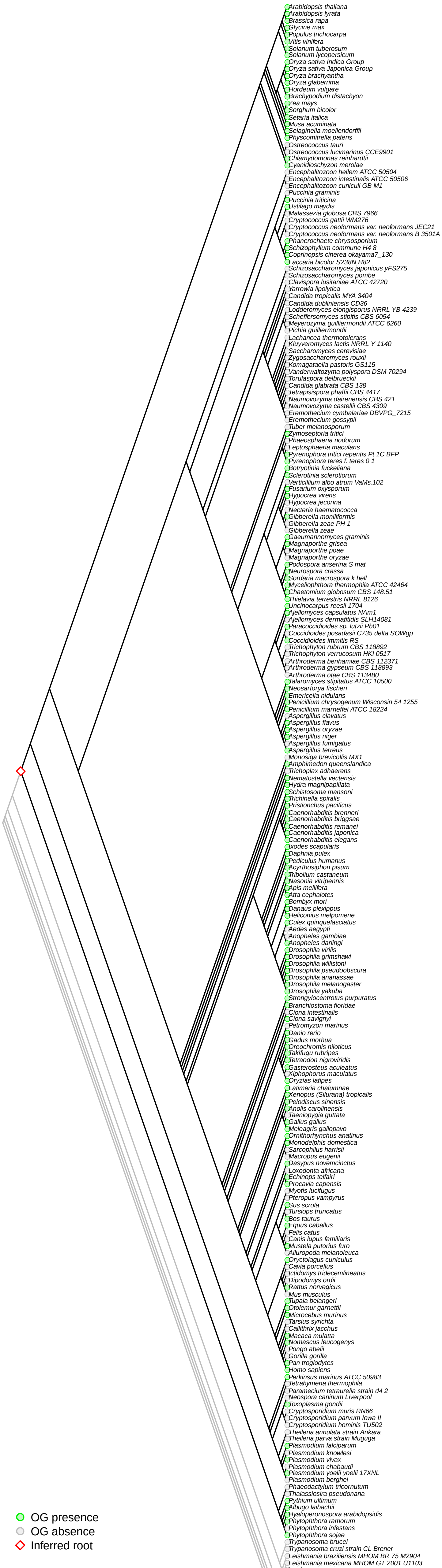

Dscore = 0.658
Pvalue = 7.58e−04
AdjPvalue = 9.02e−02

NOG121808

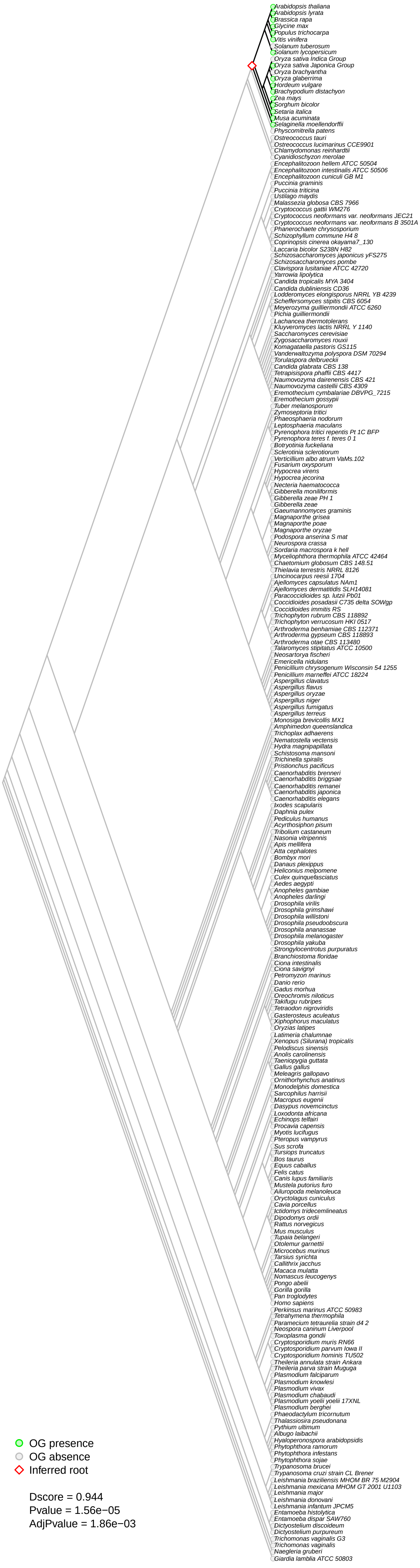

Arabidopsis thaliana
Arabidopsis lyrata
Brassica rapa
Glycine max
Populus trichocarpa
Vitis vinifera
Solanum tuberosum
Solanum lycopersicum
Oryza sativa Indica Group
Oryza sativa Japonica Group
Oryza brachyantha
Oryza glaberrima
Hordeum vulgare
Brachypodium distachyon
Zea mays
Sorghum bicolor
Setaria italica
Musa acuminata
Selaginella moellendorffii
Physcomitrella patens
Ostreococcus tauri
Ostreococcus lucimarinus CCE9901
Chlamydomonas reinhardtii
Cyanidioschyzon merolae
Encephalitozoon hellem ATCC 50504
Encephalitozoon intestinalis ATCC 50506
Encephalitozoon cuniculi GB M1
Puccinia graminis
Puccinia triticina
Ustilago maydis
Malassezia globosa CBS 7966
Cryptococcus gattii WM276
Cryptococcus neoformans var. neoformans JEC21
Cryptococcus neoformans var. neoformans B 3501A
Phanerochaete chrysosporium
Schizophyllum commune H4 8
Coprinopsis cinerea okayama7_130
Laccaria bicolor S238N H82
Schizosaccharomyces japonicus yFS275
Schizosaccharomyces pombe
Clavispora lusitaniae ATCC 42720
Yarrowia lipolytica
Candida tropicalis MYA 3404
Candida dubliniensis CD36
Lodderomyces elongisporus NRRL YB 4239
Scheffersomyces stipitis CBS 6054
Meyerozyma guilliermondii ATCC 6260
Pichia guilliermondii
Lachancea thermotolerans
Kluyveromyces lactis NRRL Y 1140
Saccharomyces cerevisiae
Zygosaccharomyces rouxii
Komagataella pastoris GS115
Vanderwaltozyma polyspora DSM 70294
Torulaspora delbrueckii
Candida glabrata CBS 138
Tetrapisispora phaffii CBS 4417
Naumovozyma dairenensis CBS 421
Naumovozyma castellii CBS 4309
Eremothecium cymbalariae DBVPG_7215
Eremothecium gossypii
Tuber melanosporum
Zymoseptoria tritici
Phaeosphaeria nodorum
Leptosphaeria maculans
Pyrenophora tritici repentis Pt 1C BFP
Pyrenophora teres f. teres 0 1
Botryotinia fuckeliana
Sclerotinia sclerotiorum
Verticillium albo atrum VaMs.102
Fusarium oxysporum
Hypocrea virens
Hypocrea jecorina
Nectria haematococca
Gibberella moniliformis
Gibberella zeae PH 1
Gibberella zeae
Gaeumannomyces graminis
Magnaporthe grisea
Magnaporthe poae
Magnaporthe oryzae
Podospora anserina S mat
Neurospora crassa
Sordaria macrospora k hell
Myceliophthora thermophila ATCC 42464
Chaetomium globosum CBS 148.51
Thielavia terrestris NRRL 8126
Uncinocarpus reesii 1704
Ajellomyces capsulatus NAm1
Ajellomyces dermatitidis SLH14081
Paracoccidioides sp. lutzii Pb01
Coccidioides posadasii C735 delta SOWgp
Coccidioides immitis RS
Trichophyton rubrum CBS 118892
Trichophyton verrucosum HKI 0517
Arthroderma benhamiae CBS 112371
Arthroderma gypseum CBS 118893
Arthroderma otae CBS 113480
Talaromyces stipitatus ATCC 10500
Neosartorya fischeri
Emericella nidulans
Penicillium chrysogenum Wisconsin 54 1255
Penicillium marneffei ATCC 18224
Aspergillus clavatus
Aspergillus flavus
Aspergillus oryzae
Aspergillus niger
Aspergillus fumigatus
Aspergillus terreus
Monosiga brevicollis MX1
Amphimedon queenslandica
Trichoplax adhaerens
Nematostella vectensis
Hydra magnipapillata
Schistosoma mansoni
Trichinella spiralis
Pristionchus pacificus
Caenorhabditis brenneri
Caenorhabditis briggsae
Caenorhabditis remanei
Caenorhabditis japonica
Caenorhabditis elegans
Ixodes scapularis
Daphnia pulex
Pediculus humanus
Acyrthosiphon pisum
Tribolium castaneum
Nasonia vitripennis
Apis mellifera
Atta cephalotes
Bombyx mori
Danaus plexippus
Heliconius melpomene
Culex quinquefasciatus
Aedes aegypti
Anopheles gambiae
Anopheles darlingi
Drosophila virilis
Drosophila grimshawi
Drosophila willistoni
Drosophila pseudoobscura
Drosophila ananassae
Drosophila melanogaster
Drosophila yakuba
Strongylocentrotus purpuratus
Branchiostoma floridae
Ciona intestinalis
Ciona savignyi
Petromyzon marinus
Danio rerio
Gadus morhua
Oreochromis niloticus
Takifugu rubripes
Tetraodon nigroviridis
Gasterosteus aculeatus
Xiphophorus maculatus
Oryzias latipes
Latimeria chalumnae
Xenopus (Silurana) tropicalis
Pelodiscus sinensis
Anolis carolinensis
Taeniopygia guttata
Gallus gallus
Meleagris gallopavo
Ornithorhynchus anatinus
Monodelphis domestica
Sarcophilus harrisii
Macropus eugenii
Dasypus novemcinctus
Loxodonta africana
Echinops telfairi
Procavia capensis
Myotis lucifugus
Pteropus vampyrus
Sus scrofa
Tursiops truncatus
Bos taurus
Equus caballus
Felis catus
Canis lupus familiaris
Mustela putorius furo
Ailuropoda melanoleuca
Oryctolagus cuniculus
Cavia porcellus
Ictidomys tridecemlineatus
Dipodomys ordii
Rattus norvegicus
Mus musculus
Tupaia belangeri
Otolemur garnettii
Microcebus murinus
Tarsius syrichta
Callithrix jacchus
Macaca mulatta
Nomascus leucogenys
Pongo abelii
Gorilla gorilla
Pan troglodytes
Homo sapiens
Perkinsus marinus ATCC 50983
Tetrahymena thermophila
Paramecium tetraurelia strain d4 2
Neospora caninum Liverpool
Toxoplasma gondii
Cryptosporidium muris RN66
Cryptosporidium parvum Iowa II
Cryptosporidium hominis TU502
Theileria annulata strain Ankara
Theileria parva strain Muguga
Plasmodium falciparum
Plasmodium knowlesi
Plasmodium vivax
Plasmodium chabaudi
Plasmodium yoelii yoelii 17XNL
Plasmodium berghei
Phaeodactylum tricornutum
Thalassiosira pseudonana
Pythium ultimum
Albugo laibachii
Hyaloperonospora arabidopsidis
Phytophthora ramorum
Phytophthora infestans
Phytophthora sojae
Trypanosoma brucei
Trypanosoma cruzi strain CL Brener
Leishmania braziliensis MHOM BR 75 M2904
Leishmania mexicana MHOM GT 2001 U1103
Leishmania major
Leishmania donovani
Leishmania infantum JPCM5
Entamoeba histolytica
Entamoeba dispar SAW760
Dictyostelium discoideum
Dictyostelium purpureum
Trichomonas vaginalis G3
Trichomonas vaginalis
Naegleria gruberi
Giardia lamblia ATCC 50803
OG presence
OG absence
Inferred root
Dscore = 0.944
Pvalue = 1.56e−05
AdjPvalue = 1.86e−03

NOG122617

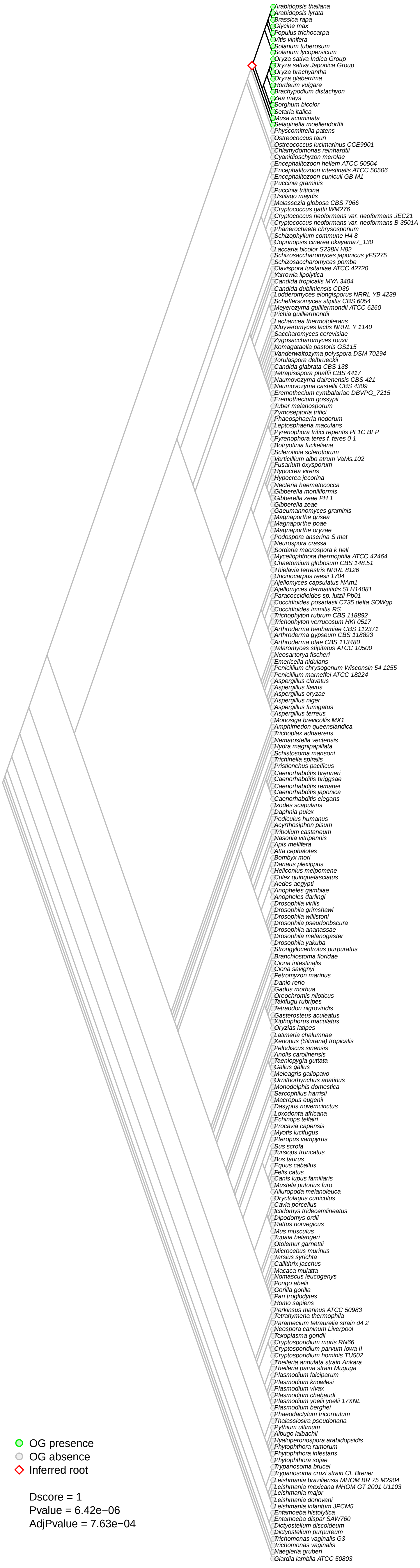

OG presence
OG absence
Inferred root

Dscore = 1
Pvalue = 6.42e−06
AdjPvalue = 7.63e−04

NOG123237

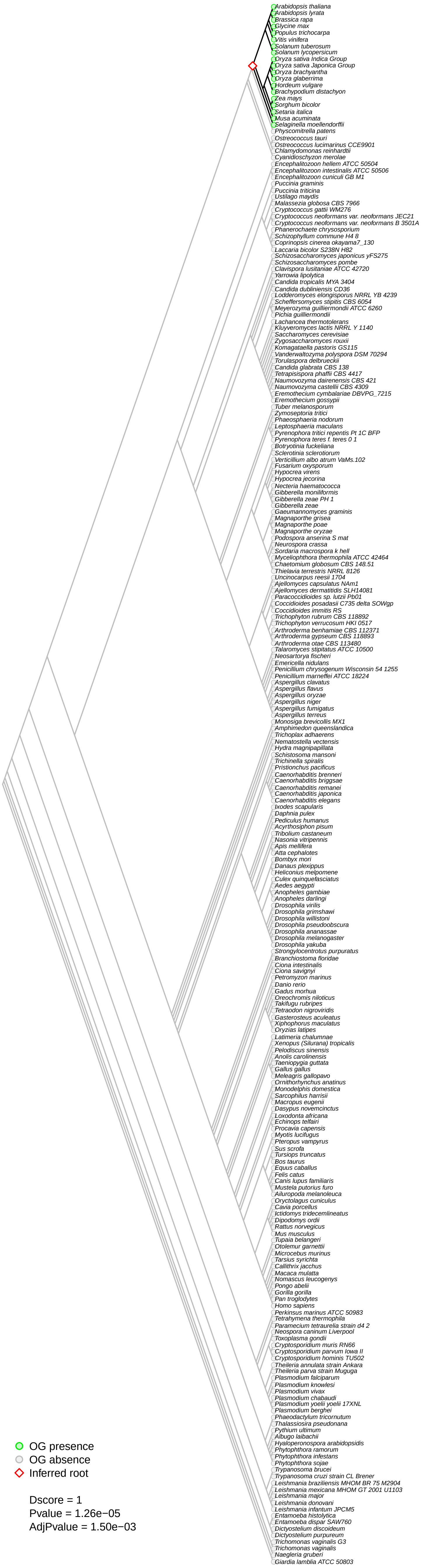

Arabidopsis thaliana
Arabidopsis lyrata
Brassica rapa
Glycine max
Populus trichocarpa
Vitis vinifera
Solanum tuberosum
Solanum lycopersicum
Oryza sativa Indica Group
Oryza sativa Japonica Group
Oryza brachyantha
Oryza glaberrima
Hordeum vulgare
Brachypodium distachyon
Zea mays
Sorghum bicolor
Setaria italica
Musa acuminata
Selaginella moellendorffii
Physcomitrella patens
Ostreococcus tauri
Ostreococcus lucimarinus CCE9901
Chlamydomonas reinhardtii
Cyanidioschyzon merolae
Encephalitozoon hellem ATCC 50504
Encephalitozoon intestinalis ATCC 50506
Encephalitozoon cuniculi GB M1
Puccinia graminis
Puccinia triticina
Ustilago maydis
Malassezia globosa CBS 7966
Cryptococcus gattii WM276
Cryptococcus neoformans var. neoformans JEC21
Cryptococcus neoformans var. neoformans B 3501A
Phanerochaete chrysosporium
Schizophyllum commune H4 8
Coprinopsis cinerea okayama7_130
Laccaria bicolor S238N H82
Schizosaccharomyces japonicus yFS275
Schizosaccharomyces pombe
Clavispora lusitaniae ATCC 42720
Yarrowia lipolytica
Candida tropicalis MYA 3404
Candida dubliniensis CD36
Lodderomyces elongisporus NRRL YB 4239
Scheffersomyces stipitis CBS 6054
Meyerozyma guilliermondii ATCC 6260
Pichia guilliermondii
Lachancea thermotolerans
Kluyveromyces lactis NRRL Y 1140
Saccharomyces cerevisiae
Zygosaccharomyces rouxii
Komagataella pastoris GS115
Vanderwaltozyma polyspora DSM 70294
Torulaspora delbrueckii
Candida glabrata CBS 138
Tetrapisispora phaffii CBS 4417
Naumovozyma dairenensis CBS 421
Naumovozyma castellii CBS 4309
Eremothecium cymbalariae DBVPG_7215
Eremothecium gossypii
Tuber melanosporum
Zymoseptoria tritici
Phaeosphaeria nodorum
Leptosphaeria maculans
Pyrenophora tritici repentis Pt 1C BFP
Pyrenophora teres f. teres 0 1
Botryotinia fuckeliana
Sclerotinia sclerotiorum
Verticillium albo atrum VaMs.102
Fusarium oxysporum
Hypocrea virens
Hypocrea jecorina
Necteria haematococca
Gibberella moniliformis
Gibberella zeae PH 1
Gibberella zeae
Gaeumannomyces graminis
Magnaporthe grisea
Magnaporthe poae
Magnaporthe oryzae
Podospora anserina S mat
Neurospora crassa
Sordaria macrospora k hell
Myceliophthora thermophila ATCC 42464
Chaetomium globosum CBS 148.51
Thielavia terrestris NRRL 8126
Uncinocarpus reesii 1704
Ajellomyces capsulatus NAm1
Ajellomyces dermatitidis SLH14081
Paracoccidioides sp. lutzii Pb01
Coccidioides posadasii C735 delta SOWgp
Coccidioides immitis RS
Trichophyton rubrum CBS 118892
Trichophyton verrucosum HKI 0517
Arthroderma benhamiae CBS 112371
Arthroderma gypseum CBS 118893
Arthroderma otae CBS 113480
Talaromyces stipitatus ATCC 10500
Neosartorya fischeri
Emericella nidulans
Penicillium chrysogenum Wisconsin 54 1255
Penicillium marneffei ATCC 18224
Aspergillus clavatus
Aspergillus flavus
Aspergillus oryzae
Aspergillus niger
Aspergillus fumigatus
Aspergillus terreus
Monosiga brevicollis MX1
Amphimedon queenslandica
Trichoplax adhaerens
Nematostella vectensis
Hydra magnipapillata
Schistosoma mansoni
Trichinella spiralis
Pristionchus pacificus
Caenorhabditis brenneri
Caenorhabditis briggsae
Caenorhabditis remanei
Caenorhabditis japonica
Caenorhabditis elegans
Ixodes scapularis
Daphnia pulex
Pediculus humanus
Acyrthosiphon pisum
Tribolium castaneum
Nasonia vitripennis
Apis mellifera
Atta cephalotes
Bombyx mori
Danaus plexippus
Heliconius melpomene
Culex quinquefasciatus
Aedes aegypti
Anopheles gambiae
Anopheles darlingi
Drosophila virilis
Drosophila grimshawi
Drosophila willistoni
Drosophila pseudoobscura
Drosophila ananassae
Drosophila melanogaster
Drosophila yakuba
Strongylocentrotus purpuratus
Branchiostoma floridae
Ciona intestinalis
Ciona savignyi
Petromyzon marinus
Danio rerio
Gadus morhua
Oreochromis niloticus
Takifugu rubripes
Tetraodon nigroviridis
Gasterosteus aculeatus
Xiphophorus maculatus
Oryzias latipes
Latimeria chalumnae
Xenopus (Silurana) tropicalis
Pelodiscus sinensis
Anolis carolinensis
Taeniopygia guttata
Gallus gallus
Meleagris gallopavo
Ornithorhynchus anatinus
Monodelphis domestica
Sarcophilus harrisii
Macropus eugenii
Dasypus novemcinctus
Loxodonta africana
Echinops telfairi
Procavia capensis
Myotis lucifugus
Pteropus vampyrus
Sus scrofa
Tursiops truncatus
Bos taurus
Equus caballus
Felis catus
Canis lupus familiaris
Mustela putorius furo
Ailuropoda melanoleuca
Oryctolagus cuniculus
Cavia porcellus
Ictidomys tridecemlineatus
Dipodomys ordii
Rattus norvegicus
Mus musculus
Tupaia belangeri
Otolemur garnettii
Microcebus murinus
Tarsius syrichta
Callithrix jacchus
Macaca mulatta
Nomascus leucogenys
Pongo abelii
Gorilla gorilla
Pan troglodytes
Homo sapiens
Perkinsus marinus ATCC 50983
Tetrahymena thermophila
Paramecium tetraurelia strain d4 2
Neospora caninum Liverpool
Toxoplasma gondii
Cryptosporidium muris RN66
Cryptosporidium parvum Iowa II
Cryptosporidium hominis TU502
Theileria annulata strain Ankara
Theileria parva strain Muguga
Plasmodium falciparum
Plasmodium knowlesi
Plasmodium vivax
Plasmodium chabaudi
Plasmodium yoelii yoelii 17XNL
Plasmodium berghei
Phaeodactylum tricornutum
Thalassiosira pseudonana
Pythium ultimum
Albugo laibachii
Hyaloperonospora arabidopsidis
Phytophthora ramorum
Phytophthora infestans
Phytophthora sojae
Trypanosoma brucei
Trypanosoma cruzi strain CL Brener
Leishmania braziliensis MHOM BR 75 M2904
Leishmania mexicana MHOM GT 2001 U1103
Leishmania major
Leishmania donovani
Leishmania infantum JPCM5
Entamoeba histolytica
Entamoeba dispar SAW760
Dictyostelium discoideum
Dictyostelium purpureum
Trichomonas vaginalis G3
Trichomonas vaginalis
Naegleria gruberi
Giardia lamblia ATCC 50803
OG presence
OG absence
Inferred root
Dscore = 1
Pvalue = 1.26e−05
AdjPvalue = 1.50e−03

NOG123538

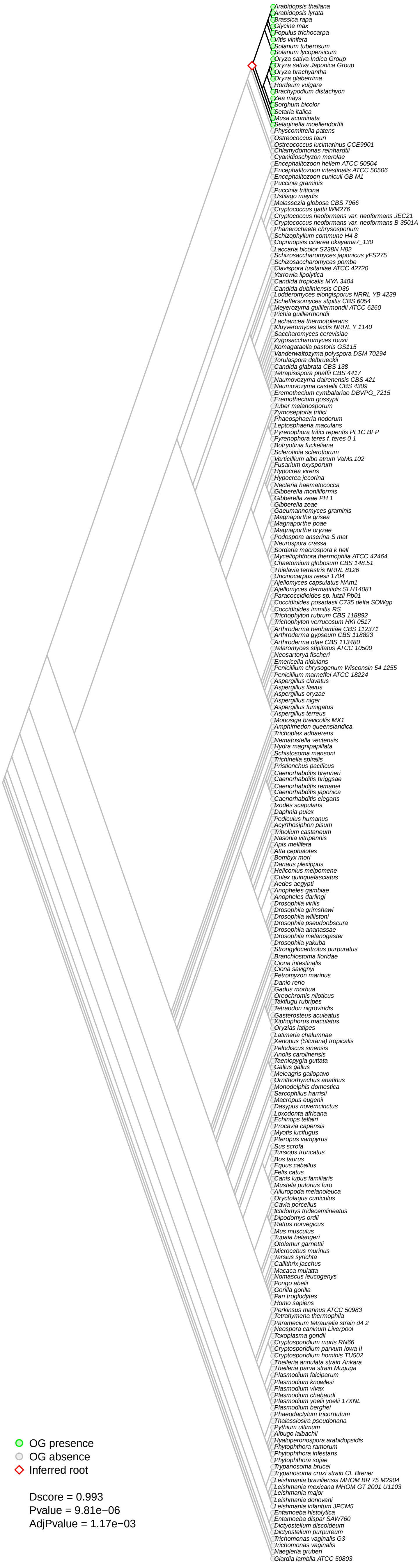

Arabidopsis thaliana
Arabidopsis lyrata
Brassica rapa
Glycine max
Populus trichocarpa
Vitis vinifera
Solanum tuberosum
Solanum lycopersicum
Oryza sativa Indica Group
Oryza sativa Japonica Group
Oryza brachyantha
Oryza glaberrima
Hordeum vulgare
Brachypodium distachyon
Zea mays
Sorghum bicolor
Setaria italica
Musa acuminata
Selaginella moellendorffii
Physcomitrella patens
Ostreococcus tauri
Ostreococcus lucimarinus CCE9901
Chlamydomonas reinhardtii
Cyanidioschyzon merolae
Encephalitozoon hellem ATCC 50504
Encephalitozoon intestinalis ATCC 50506
Encephalitozoon cuniculi GB M1
Puccinia graminis
Puccinia triticina
Ustilago maydis
Malassezia globosa CBS 7966
Cryptococcus gattii WM276
Cryptococcus neoformans var. neoformans JEC21
Cryptococcus neoformans var. neoformans B 3501A
Phanerochaete chrysosporium
Schizophyllum commune H4 8
Coprinopsis cinerea okayama7_130
Laccaria bicolor S238N H82
Schizosaccharomyces japonicus yFS275
Schizosaccharomyces pombe
Clavispora lusitaniae ATCC 42720
Yarrowia lipolytica
Candida tropicalis MYA 3404
Candida dubliniensis CD36
Lodderomyces elongisporus NRRL YB 4239
Scheffersomyces stipitis CBS 6054
Meyerozyma guilliermondii ATCC 6260
Pichia guilliermondii
Lachancea thermotolerans
Kluyveromyces lactis NRRL Y 1140
Saccharomyces cerevisiae
Zygosaccharomyces rouxii
Komagataella pastoris GS115
Vanderwaltozyma polyspora DSM 70294
Torulaspora delbrueckii
Candida glabrata CBS 138
Tetrapisispora phaffii CBS 4417
Naumovozyma dairenensis CBS 421
Naumovozyma castellii CBS 4309
Eremothecium cymbalariae DBVPG_7215
Eremothecium gossypii
Tuber melanosporum
Zymoseptoria tritici
Phaeosphaeria nodorum
Leptosphaeria maculans
Pyrenophora tritici repentis Pt 1C BFP
Pyrenophora teres f. teres 0 1
Botryotinia fuckeliana
Sclerotinia sclerotiorum
Verticillium albo atrum VaMs.102
Fusarium oxysporum
Hypocrea virens
Hypocrea jecorina
Nectria haematococca
Gibberella moniliformis
Gibberella zeae PH 1
Gibberella zeae
Gaeumannomyces graminis
Magnaporthe grisea
Magnaporthe poae
Magnaporthe oryzae
Podospora anserina S mat
Neurospora crassa
Sordaria macrospora k hell
Myceliophthora thermophila ATCC 42464
Chaetomium globosum CBS 148.51
Thielavia terrestris NRRL 8126
Uncinocarpus reesii 1704
Ajellomyces capsulatus NAm1
Ajellomyces dermatitidis SLH14081
Paracoccidioides sp. lutzii Pb01
Coccidioides posadasii C735 delta SOWgp
Coccidioides immitis RS
Trichophyton rubrum CBS 118892
Trichophyton verrucosum HKI 0517
Arthroderma benhamiae CBS 112371
Arthroderma gypseum CBS 118893
Arthroderma otae CBS 113480
Talaromyces stipitatus ATCC 10500
Neosartorya fischeri
Emericella nidulans
Penicillium chrysogenum Wisconsin 54 1255
Penicillium marneffei ATCC 18224
Aspergillus clavatus
Aspergillus flavus
Aspergillus oryzae
Aspergillus niger
Aspergillus fumigatus
Aspergillus terreus
Monosiga brevicollis MX1
Amphimedon queenslandica
Trichoplax adhaerens
Nematostella vectensis
Hydra magnipapillata
Schistosoma mansoni
Trichinella spiralis
Pristionchus pacificus
Caenorhabditis brenneri
Caenorhabditis briggsae
Caenorhabditis remanei
Caenorhabditis japonica
Caenorhabditis elegans
Ixodes scapularis
Daphnia pulex
Pediculus humanus
Acyrthosiphon pisum
Tribolium castaneum
Nasonia vitripennis
Apis mellifera
Atta cephalotes
Bombyx mori
Danaus plexippus
Heliconius melpomene
Culex quinquefasciatus
Aedes aegypti
Anopheles gambiae
Anopheles darlingi
Drosophila virilis
Drosophila grimshawi
Drosophila willistoni
Drosophila pseudoobscura
Drosophila ananassae
Drosophila melanogaster
Drosophila yakuba
Strongylocentrotus purpuratus
Branchiostoma floridae
Ciona intestinalis
Ciona savignyi
Petromyzon marinus
Danio rerio
Gadus morhua
Oreochromis niloticus
Takifugu rubripes
Tetraodon nigroviridis
Gasterosteus aculeatus
Xiphophorus maculatus
Oryzias latipes
Latimeria chalumnae
Xenopus (Silurana) tropicalis
Pelodiscus sinensis
Anolis carolinensis
Taeniopygia guttata
Gallus gallus
Meleagris gallopavo
Ornithorhynchus anatinus
Monodelphis domestica
Sarcophilus harrisii
Macropus eugenii
Dasypus novemcinctus
Loxodonta africana
Echinops telfairi
Procavia capensis
Myotis lucifugus
Pteropus vampyrus
Sus scrofa
Tursiops truncatus
Bos taurus
Equus caballus
Felis catus
Canis lupus familiaris
Mustela putorius furo
Ailuropoda melanoleuca
Oryctolagus cuniculus
Cavia porcellus
Ictidomys tridecemlineatus
Dipodomys ordii
Rattus norvegicus
Mus musculus
Tupaia belangeri
Otolemur garnettii
Microcebus murinus
Tarsius syrichta
Callithrix jacchus
Macaca mulatta
Nomascus leucogenys
Pongo abelii
Gorilla gorilla
Pan troglodytes
Homo sapiens
Perkinsus marinus ATCC 50983
Tetrahymena thermophila
Paramecium tetraurelia strain d4 2
Neospora caninum Liverpool
Toxoplasma gondii
Cryptosporidium muris RN66
Cryptosporidium parvum Iowa II
Cryptosporidium hominis TU502
Theileria annulata strain Ankara
Theileria parva strain Muguga
Plasmodium falciparum
Plasmodium knowlesi
Plasmodium vivax
Plasmodium chabaudi
Plasmodium yoelii yoelii 17XNL
Plasmodium berghei
Phaeodactylum tricornutum
Thalassiosira pseudonana
Pythium ultimum
Albugo laibachii
Hyaloperonospora arabidopsidis
Phytophthora ramorum
Phytophthora infestans
Phytophthora sojae
Trypanosoma brucei
Trypanosoma cruzi strain CL Brener
Leishmania braziliensis MHOM BR 75 M2904
Leishmania mexicana MHOM GT 2001 U1103
Leishmania major
Leishmania donovani
Leishmania infantum JPCM5
Entamoeba histolytica
Entamoeba dispar SAW760
Dictyostelium discoideum
Dictyostelium purpureum
Trichomonas vaginalis G3
Trichomonas vaginalis
Naegleria gruberi
Giardia lamblia ATCC 50803
OG presence
OG absence
Inferred root
Dscore = 0.993
Pvalue = 9.81e−06
AdjPvalue = 1.17e−03

NOG123647

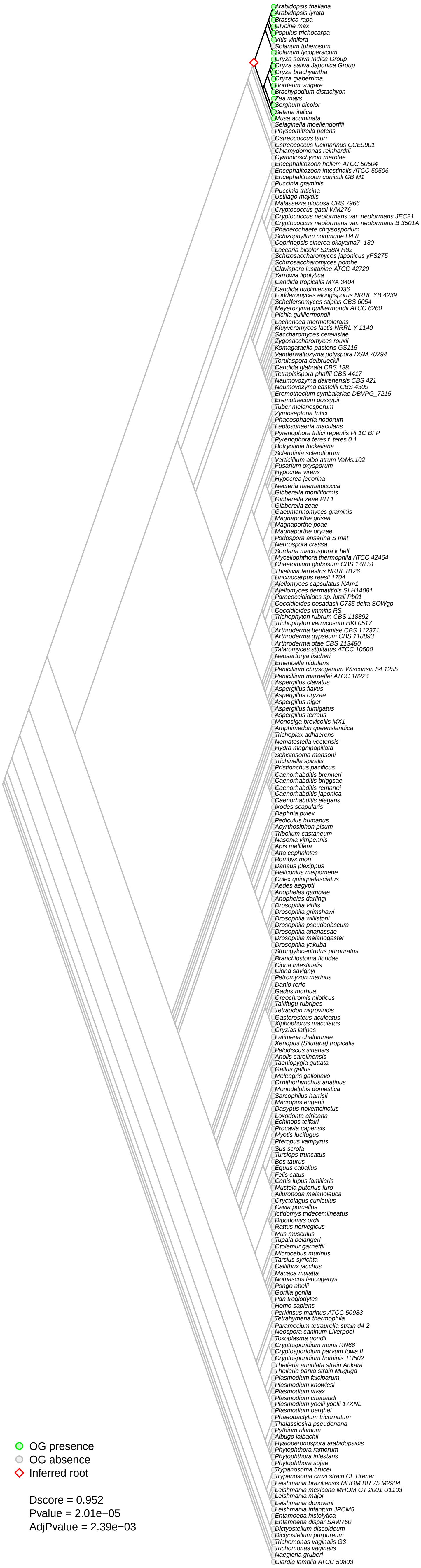
Arabidopsis thaliana
Arabidopsis lyrata
Brassica rapa
Glycine max
Populus trichocarpa
Vitis vinifera
Solanum tuberosum
Solanum lycopersicum
Oryza sativa Indica Group
Oryza sativa Japonica Group
Oryza brachyantha
Oryza glaberrima
Hordeum vulgare
Brachypodium distachyon
Zea mays
Sorghum bicolor
Setaria italica
Musa acuminata
Selaginella moellendorffii
Physcomitrella patens
Ostreococcus tauri
Ostreococcus lucimarinus CCE9901
Chlamydomonas reinhardtii
Cyanidioschyzon merolae
Encephalitozoon hellem ATCC 50504
Encephalitozoon intestinalis ATCC 50506
Encephalitozoon cuniculi GB M1
Puccinia graminis
Puccinia triticina
Ustilago maydis
Malassezia globosa CBS 7966
Cryptococcus gattii WM276
Cryptococcus neoformans var. neoformans JEC21
Cryptococcus neoformans var. neoformans B 3501A
Phanerochaete chrysosporium
Schizophyllum commune H4 8
Coprinopsis cinerea okayama7_130
Laccaria bicolor S238N H82
Schizosaccharomyces japonicus yFS275
Schizosaccharomyces pombe
Clavispora lusitaniae ATCC 42720
Yarrowia lipolytica
Candida tropicalis MYA 3404
Candida dubliniensis CD36
Lodderomyces elongisporus NRRL YB 4239
Scheffersomyces stipitis CBS 6054
Meyerozyma guilliermondii ATCC 6260
Pichia guilliermondii
Lachancea thermotolerans
Kluyveromyces lactis NRRL Y 1140
Saccharomyces cerevisiae
Zygosaccharomyces rouxii
Komagataella pastoris GS115
Vanderwaltozyma polyspora DSM 70294
Torulaspora delbrueckii
Candida glabrata CBS 138
Tetrapisispora phaffii CBS 4417
Naumovozyma dairenensis CBS 421
Naumovozyma castellii CBS 4309
Eremothecium cymbalariae DBVPG_7215
Eremothecium gossypii
Tuber melanosporum
Zymoseptoria tritici
Phaeosphaeria nodorum
Leptosphaeria maculans
Pyrenophora tritici repentis Pt 1C BFP
Pyrenophora teres f. teres 0 1
Botryotinia fuckeliana
Sclerotinia sclerotiorum
Verticillium albo atrum VaMs.102
Fusarium oxysporum
Hypocrea virens
Hypocrea jecorina
Nectria haematococca
Gibberella moniliformis
Gibberella zeae PH 1
Gibberella zeae
Gaeumannomyces graminis
Magnaporthe grisea
Magnaporthe poae
Magnaporthe oryzae
Podospora anserina S mat
Neurospora crassa
Sordaria macrospora k hell
Myceliophthora thermophila ATCC 42464
Chaetomium globosum CBS 148.51
Thielavia terrestris NRRL 8126
Uncinocarpus reesii 1704
Ajellomyces capsulatus NAm1
Ajellomyces dermatitidis SLH14081
Paracoccidioides sp. lutzii Pb01
Coccidioides posadasii C735 delta SOWgp
Coccidioides immitis RS
Trichophyton rubrum CBS 118892
Trichophyton verrucosum HKI 0517
Arthroderma benhamiae CBS 112371
Arthroderma gypseum CBS 118893
Arthroderma otae CBS 113480
Talaromyces stipitatus ATCC 10500
Neosartorya fischeri
Emericella nidulans
Penicillium chrysogenum Wisconsin 54 1255
Penicillium marneffei ATCC 18224
Aspergillus clavatus
Aspergillus flavus
Aspergillus oryzae
Aspergillus niger
Aspergillus fumigatus
Aspergillus terreus
Monosiga brevicollis MX1
Amphimedon queenslandica
Trichoplax adhaerens
Nematostella vectensis
Hydra magnipapillata
Schistosoma mansoni
Trichinella spiralis
Pristionchus pacificus
Caenorhabditis brenneri
Caenorhabditis briggsae
Caenorhabditis remanei
Caenorhabditis japonica
Caenorhabditis elegans
Ixodes scapularis
Daphnia pulex
Pediculus humanus
Acyrthosiphon pisum
Tribolium castaneum
Nasonia vitripennis
Apis mellifera
Atta cephalotes
Bombyx mori
Danaus plexippus
Heliconius melpomene
Culex quinquefasciatus
Aedes aegypti
Anopheles gambiae
Anopheles darlingi
Drosophila virilis
Drosophila grimshawi
Drosophila willistoni
Drosophila pseudoobscura
Drosophila ananassae
Drosophila melanogaster
Drosophila yakuba
Strongylocentrotus purpuratus
Branchiostoma floridae
Ciona intestinalis
Ciona savignyi
Petromyzon marinus
Danio rerio
Gadus morhua
Oreochromis niloticus
Takifugu rubripes
Tetraodon nigroviridis
Gasterosteus aculeatus
Xiphophorus maculatus
Oryzias latipes
Latimeria chalumnae
Xenopus (Silurana) tropicalis
Pelodiscus sinensis
Anolis carolinensis
Taeniopygia guttata
Gallus gallus
Meleagris gallopavo
Ornithorhynchus anatinus
Monodelphis domestica
Sarcophilus harrisii
Macropus eugenii
Dasypus novemcinctus
Loxodonta africana
Echinops telfairi
Procavia capensis
Myotis lucifugus
Pteropus vampyrus
Sus scrofa
Tursiops truncatus
Bos taurus
Equus caballus
Felis catus
Canis lupus familiaris
Mustela putorius furo
Ailuropoda melanoleuca
Oryctolagus cuniculus
Cavia porcellus
Ictidomys tridecemlineatus
Dipodomys ordii
Rattus norvegicus
Mus musculus
Tupaia belangeri
Otolemur garnettii
Microcebus murinus
Tarsius syrichta
Callithrix jacchus
Macaca mulatta
Nomascus leucogenys
Pongo abelii
Gorilla gorilla
Pan troglodytes
Homo sapiens
Perkinsus marinus ATCC 50983
Tetrahymena thermophila
Paramecium tetraurelia strain d4 2
Neospora caninum Liverpool
Toxoplasma gondii
Cryptosporidium muris RN66
Cryptosporidium parvum Iowa II
Cryptosporidium hominis TU502
Theileria annulata strain Ankara
Theileria parva strain Muguga
Plasmodium falciparum
Plasmodium knowlesi
Plasmodium vivax
Plasmodium chabaudi
Plasmodium yoelii yoelii 17XNL
Plasmodium berghei
Phaeodactylum tricornutum
Thalassiosira pseudonana
Pythium ultimum
Albugo laibachii
Hyaloperonospora arabidopsidis
Phytophthora ramorum
Phytophthora infestans
Phytophthora sojae
Trypanosoma brucei
Trypanosoma cruzi strain CL Brener
Leishmania braziliensis MHOM BR 75 M2904
Leishmania mexicana MHOM GT 2001 U1103
Leishmania major
Leishmania donovani
Leishmania infantum JPCM5
Entamoeba histolytica
Entamoeba dispar SAW760
Dictyostelium discoideum
Dictyostelium purpureum
Trichomonas vaginalis G3
Trichomonas vaginalis
Naegleria gruberi
Giardia lamblia ATCC 50803
OG presence
OG absence
Inferred root
Dscore = 0.952
Pvalue = 2.01e−05
AdjPvalue = 2.39e−03

NOG124136

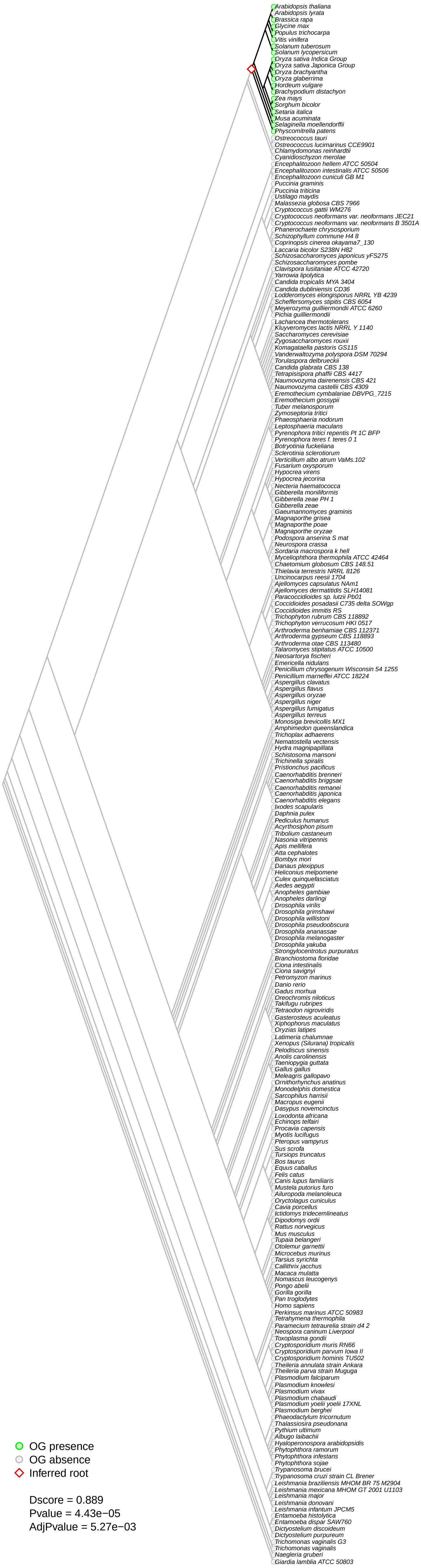

Arabidopsis thaliana
Arabidopsis lyrata
Brassica rapa
Glycine max
Populus trichocarpa
Vitis vinifera
Solanum tuberosum
Solanum lycopersicum
Oryza sativa Indica Group
Oryza sativa Japonica Group
Oryza brachyantha
Oryza glaberrima
Hordeum vulgare
Brachypodium distachyon
Zea mays
Sorghum bicolor
Setaria italica
Musa acuminata
Selaginella moellendorffii
Physcomitrella patens
Ostreococcus tauri
Ostreococcus lucimarinus CCE9901
Chlamydomonas reinhardtii
Cyanidioschyzon merolae
Encephalitozoon hellem ATCC 50504
Encephalitozoon intestinalis ATCC 50506
Encephalitozoon cuniculi GB M1
Puccinia graminis
Puccinia triticina
Ustilago maydis
Malassezia globosa CBS 7966
Cryptococcus gattii WM276
Cryptococcus neoformans var. neoformans JEC21
Cryptococcus neoformans var. neoformans B 3501A
Phanerochaete chrysosporium
Schizophyllum commune H4 8
Coprinopsis cinerea okayama7_130
Laccaria bicolor S238N H82
Schizosaccharomyces japonicus yFS275
Schizosaccharomyces pombe
Clavispora lusitaniae ATCC 42720
Yarrowia lipolytica
Candida tropicalis MYA 3404
Candida dubliniensis CD36
Lodderomyces elongisporus NRRL YB 4239
Scheffersomyces stipitis CBS 6054
Meyerozyma guilliermondii ATCC 6260
Pichia guilliermondii
Lachancea thermotolerans
Kluyveromyces lactis NRRL Y 1140
Saccharomyces cerevisiae
Zygosaccharomyces rouxii
Komagataella pastoris GS115
Vanderwaltozyma polyspora DSM 70294
Torulaspora delbrueckii
Candida glabrata CBS 138
Tetrapisispora phaffii CBS 4417
Naumovozyma dairenensis CBS 421
Naumovozyma castellii CBS 4309
Eremothecium cymbalariae DBVPG_7215
Eremothecium gossypii
Tuber melanosporum
Zymoseptoria tritici
Phaeosphaeria nodorum
Leptosphaeria maculans
Pyrenophora tritici repentis Pt 1C BFP
Pyrenophora teres f. teres 0 1
Botryotinia fuckeliana
Sclerotinia sclerotiorum
Verticillium albo atrum VaMs.102
Fusarium oxysporum
Hypocrea virens
Hypocrea jecorina
Nectria haematococca
Gibberella moniliformis
Gibberella zeae PH 1
Gibberella zeae
Gaeumannomyces graminis
Magnaporthe grisea
Magnaporthe poae
Magnaporthe oryzae
Podospora anserina S mat
Neurospora crassa
Sordaria macrospora k hell
Myceliophthora thermophila ATCC 42464
Chaetomium globosum CBS 148.51
Thielavia terrestris NRRL 8126
Uncinocarpus reesii 1704
Ajellomyces capsulatus NAm1
Ajellomyces dermatitidis SLH14081
Paracoccidioides sp. lutzii Pb01
Coccidioides posadasii C735 delta SOWgp
Coccidioides immitis RS
Trichophyton rubrum CBS 118892
Trichophyton verrucosum HKI 0517
Arthroderma benhamiae CBS 112371
Arthroderma gypseum CBS 118893
Arthroderma otae CBS 113480
Talaromyces stipitatus ATCC 10500
Neosartorya fischeri
Emericella nidulans
Penicillium chrysogenum Wisconsin 54 1255
Penicillium marneffei ATCC 18224
Aspergillus clavatus
Aspergillus flavus
Aspergillus oryzae
Aspergillus niger
Aspergillus fumigatus
Aspergillus terreus
Monosiga brevicollis MX1
Amphimedon queenslandica
Trichoplax adhaerens
Nematostella vectensis
Hydra magnipapillata
Schistosoma mansoni
Trichinella spiralis
Pristionchus pacificus
Caenorhabditis brenneri
Caenorhabditis briggsae
Caenorhabditis remanei
Caenorhabditis japonica
Caenorhabditis elegans
Ixodes scapularis
Daphnia pulex
Pediculus humanus
Acyrthosiphon pisum
Tribolium castaneum
Nasonia vitripennis
Apis mellifera
Atta cephalotes
Bombyx mori
Danaus plexippus
Heliconius melpomene
Culex quinquefasciatus
Aedes aegypti
Anopheles gambiae
Anopheles darlingi
Drosophila virilis
Drosophila grimshawi
Drosophila willistoni
Drosophila pseudoobscura
Drosophila ananassae
Drosophila melanogaster
Drosophila yakuba
Strongylocentrotus purpuratus
Branchiostoma floridae
Ciona intestinalis
Ciona savignyi
Petromyzon marinus
Danio rerio
Gadus morhua
Oreochromis niloticus
Takifugu rubripes
Tetraodon nigroviridis
Gasterosteus aculeatus
Xiphophorus maculatus
Oryzias latipes
Latimeria chalumnae
Xenopus (Silurana) tropicalis
Pelodiscus sinensis
Anolis carolinensis
Taeniopygia guttata
Gallus gallus
Meleagris gallopavo
Ornithorhynchus anatinus
Monodelphis domestica
Sarcophilus harrisii
Macropus eugenii
Dasypus novemcinctus
Loxodonta africana
Echinops telfairi
Procavia capensis
Myotis lucifugus
Pteropus vampyrus
Sus scrofa
Tursiops truncatus
Bos taurus
Equus caballus
Felis catus
Canis lupus familiaris
Mustela putorius furo
Ailuropoda melanoleuca
Oryctolagus cuniculus
Cavia porcellus
Ictidomys tridecemlineatus
Dipodomys ordii
Rattus norvegicus
Mus musculus
Tupaia belangeri
Otolemur garnettii
Microcebus murinus
Tarsius syrichta
Callithrix jacchus
Macaca mulatta
Nomascus leucogenys
Pongo abelii
Gorilla gorilla
Pan troglodytes
Homo sapiens
Perkinsus marinus ATCC 50983
Tetrahymena thermophila
Paramecium tetraurelia strain d4 2
Neospora caninum Liverpool
Toxoplasma gondii
Cryptosporidium muris RN66
Cryptosporidium parvum Iowa II
Cryptosporidium hominis TU502
Theileria annulata strain Ankara
Theileria parva strain Muguga
Plasmodium falciparum
Plasmodium knowlesi
Plasmodium vivax
Plasmodium chabaudi
Plasmodium yoelii yoelii 17XNL
Plasmodium berghei
Phaeodactylum tricornutum
Thalassiosira pseudonana
Pythium ultimum
Albugo laibachii
Hyaloperonospora arabidopsidis
Phytophthora ramorum
Phytophthora infestans
Phytophthora sojae
Trypanosoma brucei
Trypanosoma cruzi strain CL Brener
Leishmania braziliensis MHOM BR 75 M2904
Leishmania mexicana MHOM GT 2001 U1103
Leishmania major
Leishmania donovani
Leishmania infantum JPCM5
Entamoeba histolytica
Entamoeba dispar SAW760
Dictyostelium discoideum
Dictyostelium purpureum
Trichomonas vaginalis G3
Trichomonas vaginalis
Naegleria gruberi
Giardia lamblia ATCC 50803
OG presence
OG absence
Inferred root
Dscore = 0.889
Pvalue = 4.43e−05
AdjPvalue = 5.27e−03

NOG124919

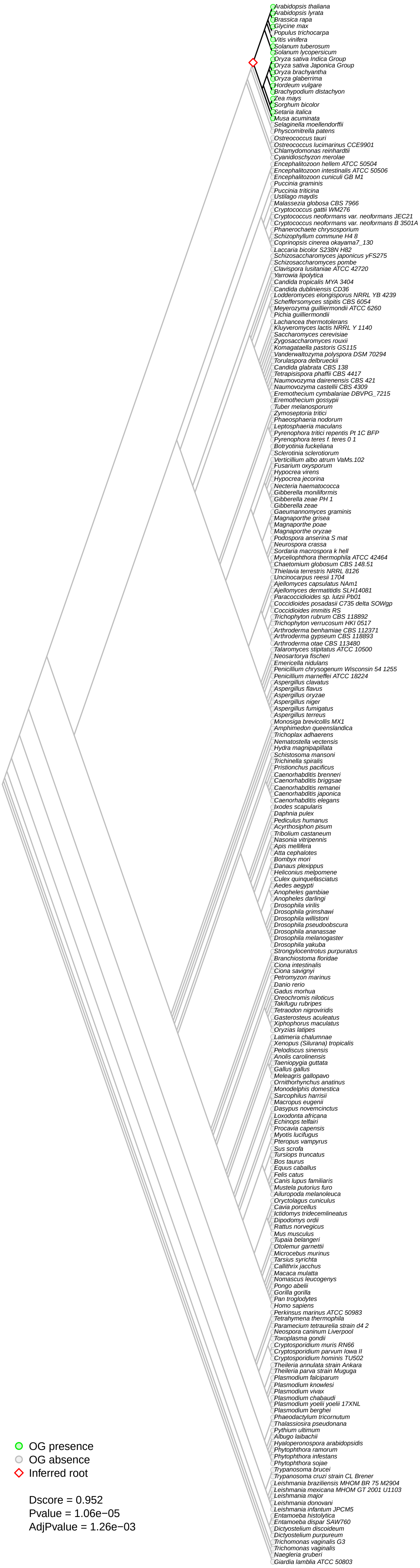

Dscore = 0.952
Pvalue = 1.06e−05
AdjPvalue = 1.26e−03

NOG125285

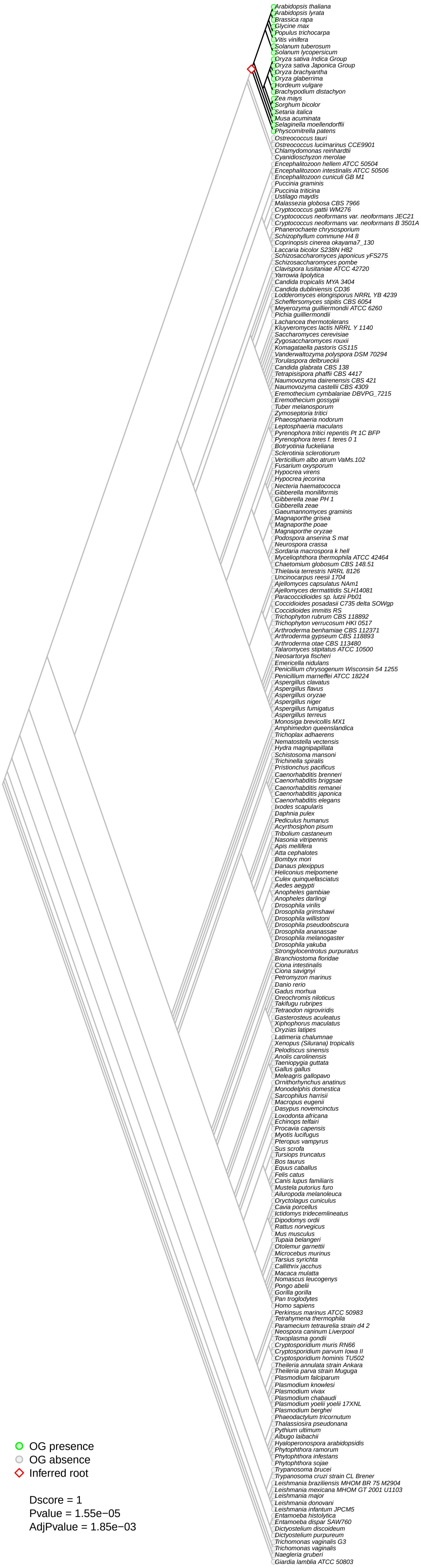
Arabidopsis thaliana
Arabidopsis lyrata
Brassica rapa
Glycine max
Populus trichocarpa
Vitis vinifera
Solanum tuberosum
Solanum lycopersicum
Oryza sativa Indica Group
Oryza sativa Japonica Group
Oryza brachyantha
Oryza glaberrima
Hordeum vulgare
Brachypodium distachyon
Zea mays
Sorghum bicolor
Setaria italica
Musa acuminata
Selaginella moellendorffii
Physcomitrella patens
Ostreococcus tauri
Ostreococcus lucimarinus CCE9901
Chlamydomonas reinhardtii
Cyanidioschyzon merolae
Encephalitozoon hellem ATCC 50504
Encephalitozoon intestinalis ATCC 50506
Encephalitozoon cuniculi GB M1
Puccinia graminis
Puccinia triticina
Ustilago maydis
Malassezia globosa CBS 7966
Cryptococcus gattii WM276
Cryptococcus neoformans var. neoformans JEC21
Cryptococcus neoformans var. neoformans B 3501A
Phanerochaete chrysosporium
Schizophyllum commune H4 8
Coprinopsis cinerea okayama7_130
Laccaria bicolor S238N H82
Schizosaccharomyces japonicus yFS275
Schizosaccharomyces pombe
Clavispora lusitaniae ATCC 42720
Yarrowia lipolytica
Candida tropicalis MYA 3404
Candida dubliniensis CD36
Lodderomyces elongisporus NRRL YB 4239
Scheffersomyces stipitis CBS 6054
Meyerozyma guilliermondii ATCC 6260
Pichia guilliermondii
Lachancea thermotolerans
Kluyveromyces lactis NRRL Y 1140
Saccharomyces cerevisiae
Zygosaccharomyces rouxii
Komagataella pastoris GS115
Vanderwaltozyma polyspora DSM 70294
Torulaspora delbrueckii
Candida glabrata CBS 138
Tetrapisispora phaffii CBS 4417
Naumovozyma dairenensis CBS 421
Naumovozyma castellii CBS 4309
Eremothecium cymbalariae DBVPG_7215
Eremothecium gossypii
Tuber melanosporum
Zymoseptoria tritici
Phaeosphaeria nodorum
Leptosphaeria maculans
Pyrenophora tritici repentis Pt 1C BFP
Pyrenophora teres f. teres 0 1
Botryotinia fuckeliana
Sclerotinia sclerotiorum
Verticillium albo atrum VaMs.102
Fusarium oxysporum
Hypocrea virens
Hypocrea jecorina
Nectria haematococca
Gibberella moniliformis
Gibberella zeae PH 1
Gibberella zeae
Gaeumannomyces graminis
Magnaporthe grisea
Magnaporthe poae
Magnaporthe oryzae
Podospora anserina S mat
Neurospora crassa
Sordaria macrospora k hell
Myceliophthora thermophila ATCC 42464
Chaetomium globosum CBS 148.51
Thielavia terrestris NRRL 8126
Uncinocarpus reesii 1704
Ajellomyces capsulatus NAm1
Ajellomyces dermatitidis SLH14081
Paracoccidioides sp. lutzii Pb01
Coccidioides posadasii C735 delta SOWgp
Coccidioides immitis RS
Trichophyton rubrum CBS 118892
Trichophyton verrucosum HKI 0517
Arthroderma benhamiae CBS 112371
Arthroderma gypseum CBS 118893
Arthroderma otae CBS 113480
Talaromyces stipitatus ATCC 10500
Neosartorya fischeri
Emericella nidulans
Penicillium chrysogenum Wisconsin 54 1255
Penicillium marneffei ATCC 18224
Aspergillus clavatus
Aspergillus flavus
Aspergillus oryzae
Aspergillus niger
Aspergillus fumigatus
Aspergillus terreus
Monosiga brevicollis MX1
Amphimedon queenslandica
Trichoplax adhaerens
Nematostella vectensis
Hydra magnipapillata
Schistosoma mansoni
Trichinella spiralis
Pristionchus pacificus
Caenorhabditis brenneri
Caenorhabditis briggsae
Caenorhabditis remanei
Caenorhabditis japonica
Caenorhabditis elegans
Ixodes scapularis
Daphnia pulex
Pediculus humanus
Acyrthosiphon pisum
Tribolium castaneum
Nasonia vitripennis
Apis mellifera
Atta cephalotes
Bombyx mori
Danaus plexippus
Heliconius melpomene
Culex quinquefasciatus
Aedes aegypti
Anopheles gambiae
Anopheles darlingi
Drosophila virilis
Drosophila grimshawi
Drosophila willistoni
Drosophila pseudoobscura
Drosophila ananassae
Drosophila melanogaster
Drosophila yakuba
Strongylocentrotus purpuratus
Branchiostoma floridae
Ciona intestinalis
Ciona savignyi
Petromyzon marinus
Danio rerio
Gadus morhua
Oreochromis niloticus
Takifugu rubripes
Tetraodon nigroviridis
Gasterosteus aculeatus
Xiphophorus maculatus
Oryzias latipes
Latimeria chalumnae
Xenopus (Silurana) tropicalis
Pelodiscus sinensis
Anolis carolinensis
Taeniopygia guttata
Gallus gallus
Meleagris gallopavo
Ornithorhynchus anatinus
Monodelphis domestica
Sarcophilus harrisii
Macropus eugenii
Dasypus novemcinctus
Loxodonta africana
Echinops telfairi
Procavia capensis
Myotis lucifugus
Pteropus vampyrus
Sus scrofa
Tursiops truncatus
Bos taurus
Equus caballus
Felis catus
Canis lupus familiaris
Mustela putorius furo
Ailuropoda melanoleuca
Oryctolagus cuniculus
Cavia porcellus
Ictidomys tridecemlineatus
Dipodomys ordii
Rattus norvegicus
Mus musculus
Tupaia belangeri
Otolemur garnettii
Microcebus murinus
Tarsius syrichta
Callithrix jacchus
Macaca mulatta
Nomascus leucogenys
Pongo abelii
Gorilla gorilla
Pan troglodytes
Homo sapiens
Perkinsus marinus ATCC 50983
Tetrahymena thermophila
Paramecium tetraurelia strain d4 2
Neospora caninum Liverpool
Toxoplasma gondii
Cryptosporidium muris RN66
Cryptosporidium parvum Iowa II
Cryptosporidium hominis TU502
Theileria annulata strain Ankara
Theileria parva strain Muguga
Plasmodium falciparum
Plasmodium knowlesi
Plasmodium vivax
Plasmodium chabaudi
Plasmodium yoelii yoelii 17XNL
Plasmodium berghei
Phaeodactylum tricornutum
Thalassiosira pseudonana
Pythium ultimum
Albugo laibachii
Hyaloperonospora arabidopsidis
Phytophthora ramorum
Phytophthora infestans
Phytophthora sojae
Trypanosoma brucei
Trypanosoma cruzi strain CL Brener
Leishmania braziliensis MHOM BR 75 M2904
Leishmania mexicana MHOM GT 2001 U1103
Leishmania major
Leishmania donovani
Leishmania infantum JPCM5
Entamoeba histolytica
Entamoeba dispar SAW760
Dictyostelium discoideum
Dictyostelium purpureum
Trichomonas vaginalis G3
Trichomonas vaginalis
Naegleria gruberi
Giardia lamblia ATCC 50803
OG presence
OG absence
Inferred root
Dscore = 1
Pvalue = 1.55e−05
AdjPvalue = 1.85e−03

NOG126611

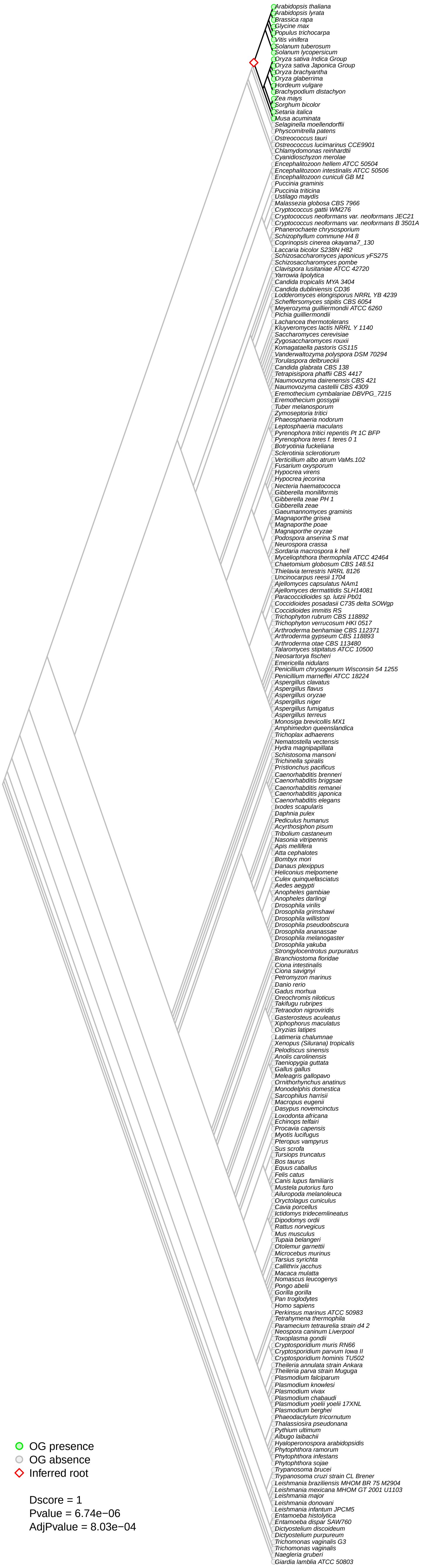
Arabidopsis thaliana
Arabidopsis lyrata
Brassica rapa
Glycine max
Populus trichocarpa
Vitis vinifera
Solanum tuberosum
Solanum lycopersicum
Oryza sativa Indica Group
Oryza sativa Japonica Group
Oryza brachyantha
Oryza glaberrima
Hordeum vulgare
Brachypodium distachyon
Zea mays
Sorghum bicolor
Setaria italica
Musa acuminata
Selaginella moellendorffii
Physcomitrella patens
Ostreococcus tauri
Ostreococcus lucimarinus CCE9901
Chlamydomonas reinhardtii
Cyanidioschyzon merolae
Encephalitozoon hellem ATCC 50504
Encephalitozoon intestinalis ATCC 50506
Encephalitozoon cuniculi GB M1
Puccinia graminis
Puccinia triticina
Ustilago maydis
Malassezia globosa CBS 7966
Cryptococcus gattii WM276
Cryptococcus neoformans var. neoformans JEC21
Cryptococcus neoformans var. neoformans B 3501A
Phanerochaete chrysosporium
Schizophyllum commune H4 8
Coprinopsis cinerea okayama7_130
Laccaria bicolor S238N H82
Schizosaccharomyces japonicus yFS275
Schizosaccharomyces pombe
Clavispora lusitaniae ATCC 42720
Yarrowia lipolytica
Candida tropicalis MYA 3404
Candida dubliniensis CD36
Lodderomyces elongisporus NRRL YB 4239
Scheffersomyces stipitis CBS 6054
Meyerozyma guilliermondii ATCC 6260
Pichia guilliermondii
Lachancea thermotolerans
Kluyveromyces lactis NRRL Y 1140
Saccharomyces cerevisiae
Zygosaccharomyces rouxii
Komagataella pastoris GS115
Vanderwaltozyma polyspora DSM 70294
Torulaspora delbrueckii
Candida glabrata CBS 138
Tetrapisispora phaffii CBS 4417
Naumovozyma dairenensis CBS 421
Naumovozyma castellii CBS 4309
Eremothecium cymbalariae DBVPG_7215
Eremothecium gossypii
Tuber melanosporum
Zymoseptoria tritici
Phaeosphaeria nodorum
Leptosphaeria maculans
Pyrenophora tritici repentis Pt 1C BFP
Pyrenophora teres f. teres 0 1
Botryotinia fuckeliana
Sclerotinia sclerotiorum
Verticillium albo atrum VaMs.102
Fusarium oxysporum
Hypocrea virens
Hypocrea jecorina
Nectria haematococca
Gibberella moniliformis
Gibberella zeae PH 1
Gibberella zeae
Gaeumannomyces graminis
Magnaporthe grisea
Magnaporthe poae
Magnaporthe oryzae
Podospora anserina S mat
Neurospora crassa
Sordaria macrospora k hell
Myceliophthora thermophila ATCC 42464
Chaetomium globosum CBS 148.51
Thielavia terrestris NRRL 8126
Uncinocarpus reesii 1704
Ajellomyces capsulatus NAm1
Ajellomyces dermatitidis SLH14081
Paracoccidioides sp. lutzii Pb01
Coccidioides posadasii C735 delta SOWgp
Coccidioides immitis RS
Trichophyton rubrum CBS 118892
Trichophyton verrucosum HKI 0517
Arthroderma benhamiae CBS 112371
Arthroderma gypseum CBS 118893
Arthroderma otae CBS 113480
Talaromyces stipitatus ATCC 10500
Neosartorya fischeri
Emericella nidulans
Penicillium chrysogenum Wisconsin 54 1255
Penicillium marneffei ATCC 18224
Aspergillus clavatus
Aspergillus flavus
Aspergillus oryzae
Aspergillus niger
Aspergillus fumigatus
Aspergillus terreus
Monosiga brevicollis MX1
Amphimedon queenslandica
Trichoplax adhaerens
Nematostella vectensis
Hydra magnipapillata
Schistosoma mansoni
Trichinella spiralis
Pristionchus pacificus
Caenorhabditis brenneri
Caenorhabditis briggsae
Caenorhabditis remanei
Caenorhabditis japonica
Caenorhabditis elegans
Ixodes scapularis
Daphnia pulex
Pediculus humanus
Acyrthosiphon pisum
Tribolium castaneum
Nasonia vitripennis
Apis mellifera
Atta cephalotes
Bombyx mori
Danaus plexippus
Heliconius melpomene
Culex quinquefasciatus
Aedes aegypti
Anopheles gambiae
Anopheles darlingi
Drosophila virilis
Drosophila grimshawi
Drosophila willistoni
Drosophila pseudoobscura
Drosophila ananassae
Drosophila melanogaster
Drosophila yakuba
Strongylocentrotus purpuratus
Branchiostoma floridae
Ciona intestinalis
Ciona savignyi
Petromyzon marinus
Danio rerio
Gadus morhua
Oreochromis niloticus
Takifugu rubripes
Tetraodon nigroviridis
Gasterosteus aculeatus
Xiphophorus maculatus
Oryzias latipes
Latimeria chalumnae
Xenopus (Silurana) tropicalis
Pelodiscus sinensis
Anolis carolinensis
Taeniopygia guttata
Gallus gallus
Meleagris gallopavo
Ornithorhynchus anatinus
Monodelphis domestica
Sarcophilus harrisii
Macropus eugenii
Dasypus novemcinctus
Loxodonta africana
Echinops telfairi
Procavia capensis
Myotis lucifugus
Pteropus vampyrus
Sus scrofa
Tursiops truncatus
Bos taurus
Equus caballus
Felis catus
Canis lupus familiaris
Mustela putorius furo
Ailuropoda melanoleuca
Oryctolagus cuniculus
Cavia porcellus
Ictidomys tridecemlineatus
Dipodomys ordii
Rattus norvegicus
Mus musculus
Tupaia belangeri
Otolemur garnettii
Microcebus murinus
Tarsius syrichta
Callithrix jacchus
Macaca mulatta
Nomascus leucogenys
Pongo abelii
Gorilla gorilla
Pan troglodytes
Homo sapiens
Perkinsus marinus ATCC 50983
Tetrahymena thermophila
Paramecium tetraurelia strain d4 2
Neospora caninum Liverpool
Toxoplasma gondii
Cryptosporidium muris RN66
Cryptosporidium parvum Iowa II
Cryptosporidium hominis TU502
Theileria annulata strain Ankara
Theileria parva strain Muguga
Plasmodium falciparum
Plasmodium knowlesi
Plasmodium vivax
Plasmodium chabaudi
Plasmodium yoelii yoelii 17XNL
Plasmodium berghei
Phaeodactylum tricornutum
Thalassiosira pseudonana
Pythium ultimum
Albugo laibachii
Hyaloperonospora arabidopsidis
Phytophthora ramorum
Phytophthora infestans
Phytophthora sojae
Trypanosoma brucei
Trypanosoma cruzi strain CL Brener
Leishmania braziliensis MHOM BR 75 M2904
Leishmania mexicana MHOM GT 2001 U1103
Leishmania major
Leishmania donovani
Leishmania infantum JPCM5
Entamoeba histolytica
Entamoeba dispar SAW760
Dictyostelium discoideum
Dictyostelium purpureum
Trichomonas vaginalis G3
Trichomonas vaginalis
Naegleria gruberi
Giardia lamblia ATCC 50803
OG presence
OG absence
Inferred root
Dscore = 1
Pvalue = 6.74e−06
AdjPvalue = 8.03e−04

NOG126859

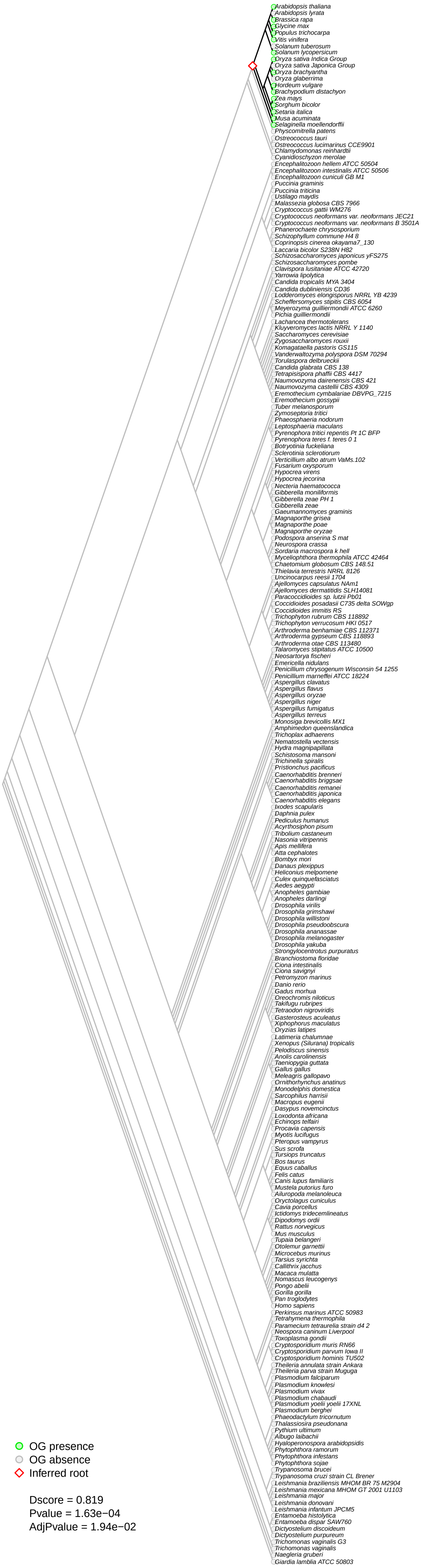
Arabidopsis thaliana
Arabidopsis lyrata
Brassica rapa
Glycine max
Populus trichocarpa
Vitis vinifera
Solanum tuberosum
Solanum lycopersicum
Oryza sativa Indica Group
Oryza sativa Japonica Group
Oryza brachyantha
Oryza glaberrima
Hordeum vulgare
Brachypodium distachyon
Zea mays
Sorghum bicolor
Setaria italica
Musa acuminata
Selaginella moellendorffii
Physcomitrella patens
Ostreococcus tauri
Ostreococcus lucimarinus CCE9901
Chlamydomonas reinhardtii
Cyanidioschyzon merolae
Encephalitozoon hellem ATCC 50504
Encephalitozoon intestinalis ATCC 50506
Encephalitozoon cuniculi GB M1
Puccinia graminis
Puccinia triticina
Ustilago maydis
Malassezia globosa CBS 7966
Cryptococcus gattii WM276
Cryptococcus neoformans var. neoformans JEC21
Cryptococcus neoformans var. neoformans B 3501A
Phanerochaete chrysosporium
Schizophyllum commune H4 8
Coprinopsis cinerea okayama7_130
Laccaria bicolor S238N H82
Schizosaccharomyces japonicus yFS275
Schizosaccharomyces pombe
Clavispora lusitaniae ATCC 42720
Yarrowia lipolytica
Candida tropicalis MYA 3404
Candida dubliniensis CD36
Lodderomyces elongisporus NRRL YB 4239
Scheffersomyces stipitis CBS 6054
Meyerozyma guilliermondii ATCC 6260
Pichia guilliermondii
Lachancea thermotolerans
Kluyveromyces lactis NRRL Y 1140
Saccharomyces cerevisiae
Zygosaccharomyces rouxii
Komagataella pastoris GS115
Vanderwaltozyma polyspora DSM 70294
Torulaspora delbrueckii
Candida glabrata CBS 138
Tetrapisispora phaffii CBS 4417
Naumovozyma dairenensis CBS 421
Naumovozyma castellii CBS 4309
Eremothecium cymbalariae DBVPG_7215
Eremothecium gossypii
Tuber melanosporum
Zymoseptoria tritici
Phaeosphaeria nodorum
Leptosphaeria maculans
Pyrenophora tritici repentis Pt 1C BFP
Pyrenophora teres f. teres 0 1
Botryotinia fuckeliana
Sclerotinia sclerotiorum
Verticillium albo atrum VaMs.102
Fusarium oxysporum
Hypocrea virens
Hypocrea jecorina
Nectria haematococca
Gibberella moniliformis
Gibberella zeae PH 1
Gibberella zeae
Gaeumannomyces graminis
Magnaporthe grisea
Magnaporthe poae
Magnaporthe oryzae
Podospora anserina S mat
Neurospora crassa
Sordaria macrospora k hell
Myceliophthora thermophila ATCC 42464
Chaetomium globosum CBS 148.51
Thielavia terrestris NRRL 8126
Uncinocarpus reesii 1704
Ajellomyces capsulatus NAm1
Ajellomyces dermatitidis SLH14081
Paracoccidioides sp. lutzii Pb01
Coccidioides posadasii C735 delta SOWgp
Coccidioides immitis RS
Trichophyton rubrum CBS 118892
Trichophyton verrucosum HKI 0517
Arthroderma benhamiae CBS 112371
Arthroderma gypseum CBS 118893
Arthroderma otae CBS 113480
Talaromyces stipitatus ATCC 10500
Neosartorya fischeri
Emericella nidulans
Penicillium chrysogenum Wisconsin 54 1255
Penicillium marneffei ATCC 18224
Aspergillus clavatus
Aspergillus flavus
Aspergillus oryzae
Aspergillus niger
Aspergillus fumigatus
Aspergillus terreus
Monosiga brevicollis MX1
Amphimedon queenslandica
Trichoplax adhaerens
Nematostella vectensis
Hydra magnipapillata
Schistosoma mansoni
Trichinella spiralis
Pristionchus pacificus
Caenorhabditis brenneri
Caenorhabditis briggsae
Caenorhabditis remanei
Caenorhabditis japonica
Caenorhabditis elegans
Ixodes scapularis
Daphnia pulex
Pediculus humanus
Acyrthosiphon pisum
Tribolium castaneum
Nasonia vitripennis
Apis mellifera
Atta cephalotes
Bombyx mori
Danaus plexippus
Heliconius melpomene
Culex quinquefasciatus
Aedes aegypti
Anopheles gambiae
Anopheles darlingi
Drosophila virilis
Drosophila grimshawi
Drosophila willistoni
Drosophila pseudoobscura
Drosophila ananassae
Drosophila melanogaster
Drosophila yakuba
Strongylocentrotus purpuratus
Branchiostoma floridae
Ciona intestinalis
Ciona savignyi
Petromyzon marinus
Danio rerio
Gadus morhua
Oreochromis niloticus
Takifugu rubripes
Tetraodon nigroviridis
Gasterosteus aculeatus
Xiphophorus maculatus
Oryzias latipes
Latimeria chalumnae
Xenopus (Silurana) tropicalis
Pelodiscus sinensis
Anolis carolinensis
Taeniopygia guttata
Gallus gallus
Meleagris gallopavo
Ornithorhynchus anatinus
Monodelphis domestica
Sarcophilus harrisii
Macropus eugenii
Dasypus novemcinctus
Loxodonta africana
Echinops telfairi
Procavia capensis
Myotis lucifugus
Pteropus vampyrus
Sus scrofa
Tursiops truncatus
Bos taurus
Equus caballus
Felis catus
Canis lupus familiaris
Mustela putorius furo
Ailuropoda melanoleuca
Oryctolagus cuniculus
Cavia porcellus
Ictidomys tridecemlineatus
Dipodomys ordii
Rattus norvegicus
Mus musculus
Tupaia belangeri
Otolemur garnettii
Microcebus murinus
Tarsius syrichta
Callithrix jacchus
Macaca mulatta
Nomascus leucogenys
Pongo abelii
Gorilla gorilla
Pan troglodytes
Homo sapiens
Perkinsus marinus ATCC 50983
Tetrahymena thermophila
Paramecium tetraurelia strain d4 2
Neospora caninum Liverpool
Toxoplasma gondii
Cryptosporidium muris RN66
Cryptosporidium parvum Iowa II
Cryptosporidium hominis TU502
Theileria annulata strain Ankara
Theileria parva strain Muguga
Plasmodium falciparum
Plasmodium knowlesi
Plasmodium vivax
Plasmodium chabaudi
Plasmodium yoelii yoelii 17XNL
Plasmodium berghei
Phaeodactylum tricornutum
Thalassiosira pseudonana
Pythium ultimum
Albugo laibachii
Hyaloperonospora arabidopsidis
Phytophthora ramorum
Phytophthora infestans
Phytophthora sojae
Trypanosoma brucei
Trypanosoma cruzi strain CL Brener
Leishmania braziliensis MHOM BR 75 M2904
Leishmania mexicana MHOM GT 2001 U1103
Leishmania major
Leishmania donovani
Leishmania infantum JPCM5
Entamoeba histolytica
Entamoeba dispar SAW760
Dictyostelium discoideum
Dictyostelium purpureum
Trichomonas vaginalis G3
Trichomonas vaginalis
Naegleria gruberi
Giardia lamblia ATCC 50803
OG presence
OG absence
Inferred root
Dscore = 0.819
Pvalue = 1.63e−04
AdjPvalue = 1.94e−02

NOG126963

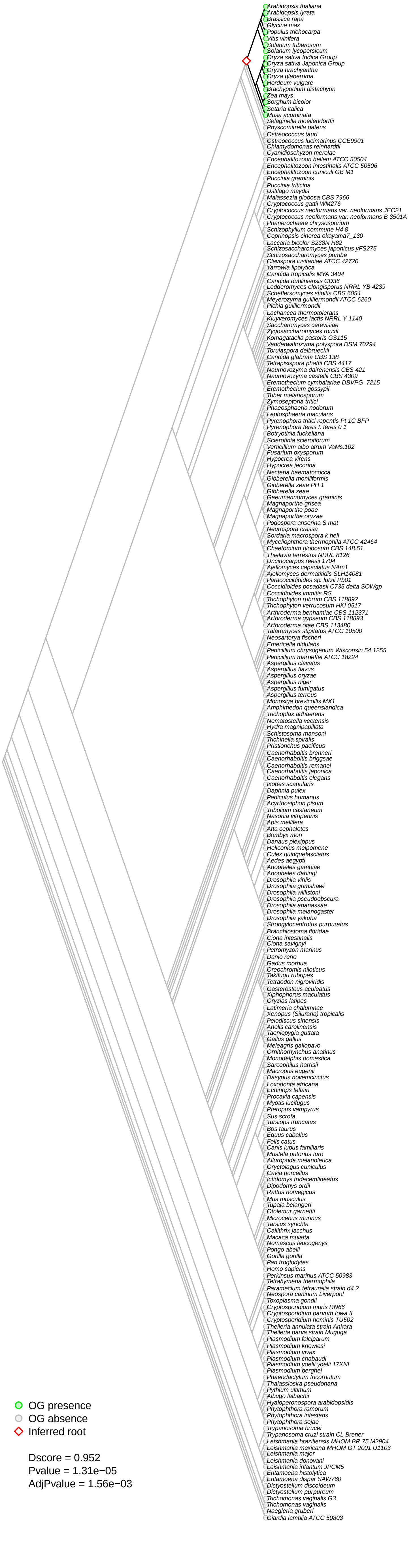
Arabidopsis thaliana
Arabidopsis lyrata
Brassica rapa
Glycine max
Populus trichocarpa
Vitis vinifera
Solanum tuberosum
Solanum lycopersicum
Oryza sativa Indica Group
Oryza sativa Japonica Group
Oryza brachyantha
Oryza glaberrima
Hordeum vulgare
Brachypodium distachyon
Zea mays
Sorghum bicolor
Setaria italica
Musa acuminata
Selaginella moellendorffii
Physcomitrella patens
Ostreococcus tauri
Ostreococcus lucimarinus CCE9901
Chlamydomonas reinhardtii
Cyanidioschyzon merolae
Encephalitozoon hellem ATCC 50504
Encephalitozoon intestinalis ATCC 50506
Encephalitozoon cuniculi GB M1
Puccinia graminis
Puccinia triticina
Ustilago maydis
Malassezia globosa CBS 7966
Cryptococcus gattii WM276
Cryptococcus neoformans var. neoformans JEC21
Cryptococcus neoformans var. neoformans B 3501A
Phanerochaete chrysosporium
Schizophyllum commune H4 8
Coprinopsis cinerea okayama7_130
Laccaria bicolor S238N H82
Schizosaccharomyces japonicus yFS275
Schizosaccharomyces pombe
Clavispora lusitaniae ATCC 42720
Yarrowia lipolytica
Candida tropicalis MYA 3404
Candida dubliniensis CD36
Lodderomyces elongisporus NRRL YB 4239
Scheffersomyces stipitis CBS 6054
Meyerozyma guilliermondii ATCC 6260
Pichia guilliermondii
Lachancea thermotolerans
Kluyveromyces lactis NRRL Y 1140
Saccharomyces cerevisiae
Zygosaccharomyces rouxii
Komagataella pastoris GS115
Vanderwaltozyma polyspora DSM 70294
Torulaspora delbrueckii
Candida glabrata CBS 138
Tetrapisispora phaffii CBS 4417
Naumovozyma dairenensis CBS 421
Naumovozyma castellii CBS 4309
Eremothecium cymbalariae DBVPG_7215
Eremothecium gossypii
Tuber melanosporum
Zymoseptoria tritici
Phaeosphaeria nodorum
Leptosphaeria maculans
Pyrenophora tritici repentis Pt 1C BFP
Pyrenophora teres f. teres 0 1
Botryotinia fuckeliana
Sclerotinia sclerotiorum
Verticillium albo atrum VaMs.102
Fusarium oxysporum
Hypocrea virens
Hypocrea jecorina
Nectria haematococca
Gibberella moniliformis
Gibberella zeae PH 1
Gibberella zeae
Gaeumannomyces graminis
Magnaporthe grisea
Magnaporthe poae
Magnaporthe oryzae
Podospora anserina S mat
Neurospora crassa
Sordaria macrospora k hell
Myceliophthora thermophila ATCC 42464
Chaetomium globosum CBS 148.51
Thielavia terrestris NRRL 8126
Uncinocarpus reesii 1704
Ajellomyces capsulatus NAm1
Ajellomyces dermatitidis SLH14081
Paracoccidioides sp. lutzii Pb01
Coccidioides posadasii C735 delta SOWgp
Coccidioides immitis RS
Trichophyton rubrum CBS 118892
Trichophyton verrucosum HKI 0517
Arthroderma benhamiae CBS 112371
Arthroderma gypseum CBS 118893
Arthroderma otae CBS 113480
Talaromyces stipitatus ATCC 10500
Neosartorya fischeri
Emericella nidulans
Penicillium chrysogenum Wisconsin 54 1255
Penicillium marneffei ATCC 18224
Aspergillus clavatus
Aspergillus flavus
Aspergillus oryzae
Aspergillus niger
Aspergillus fumigatus
Aspergillus terreus
Monosiga brevicollis MX1
Amphimedon queenslandica
Trichoplax adhaerens
Nematostella vectensis
Hydra magnipapillata
Schistosoma mansoni
Trichinella spiralis
Pristionchus pacificus
Caenorhabditis brenneri
Caenorhabditis briggsae
Caenorhabditis remanei
Caenorhabditis japonica
Caenorhabditis elegans
Ixodes scapularis
Daphnia pulex
Pediculus humanus
Acyrthosiphon pisum
Tribolium castaneum
Nasonia vitripennis
Apis mellifera
Atta cephalotes
Bombyx mori
Danaus plexippus
Heliconius melpomene
Culex quinquefasciatus
Aedes aegypti
Anopheles gambiae
Anopheles darlingi
Drosophila virilis
Drosophila grimshawi
Drosophila willistoni
Drosophila pseudoobscura
Drosophila ananassae
Drosophila melanogaster
Drosophila yakuba
Strongylocentrotus purpuratus
Branchiostoma floridae
Ciona intestinalis
Ciona savignyi
Petromyzon marinus
Danio rerio
Gadus morhua
Oreochromis niloticus
Takifugu rubripes
Tetraodon nigroviridis
Gasterosteus aculeatus
Xiphophorus maculatus
Oryzias latipes
Latimeria chalumnae
Xenopus (Silurana) tropicalis
Pelodiscus sinensis
Anolis carolinensis
Taeniopygia guttata
Gallus gallus
Meleagris gallopavo
Ornithorhynchus anatinus
Monodelphis domestica
Sarcophilus harrisii
Macropus eugenii
Dasypus novemcinctus
Loxodonta africana
Echinops telfairi
Procavia capensis
Myotis lucifugus
Pteropus vampyrus
Sus scrofa
Tursiops truncatus
Bos taurus
Equus caballus
Felis catus
Canis lupus familiaris
Mustela putorius furo
Ailuropoda melanoleuca
Oryctolagus cuniculus
Cavia porcellus
Ictidomys tridecemlineatus
Dipodomys ordii
Rattus norvegicus
Mus musculus
Tupaia belangeri
Otolemur garnettii
Microcebus murinus
Tarsius syrichta
Callithrix jacchus
Macaca mulatta
Nomascus leucogenys
Pongo abelii
Gorilla gorilla
Pan troglodytes
Homo sapiens
Perkinsus marinus ATCC 50983
Tetrahymena thermophila
Paramecium tetraurelia strain d4 2
Neospora caninum Liverpool
Toxoplasma gondii
Cryptosporidium muris RN66
Cryptosporidium parvum Iowa II
Cryptosporidium hominis TU502
Theileria annulata strain Ankara
Theileria parva strain Muguga
Plasmodium falciparum
Plasmodium knowlesi
Plasmodium vivax
Plasmodium chabaudi
Plasmodium yoelii yoelii 17XNL
Plasmodium berghei
Phaeodactylum tricornutum
Thalassiosira pseudonana
Pythium ultimum
Albugo laibachii
Hyaloperonospora arabidopsidis
Phytophthora ramorum
Phytophthora infestans
Phytophthora sojae
Trypanosoma brucei
Trypanosoma cruzi strain CL Brener
Leishmania braziliensis MHOM BR 75 M2904
Leishmania mexicana MHOM GT 2001 U1103
Leishmania major
Leishmania donovani
Leishmania infantum JPCM5
Entamoeba histolytica
Entamoeba dispar SAW760
Dictyostelium discoideum
Dictyostelium purpureum
Trichomonas vaginalis G3
Trichomonas vaginalis
Naegleria gruberi
Giardia lamblia ATCC 50803
OG presence
OG absence
Inferred root
Dscore = 0.952
Pvalue = 1.31e−05
AdjPvalue = 1.56e−03

NOG128070

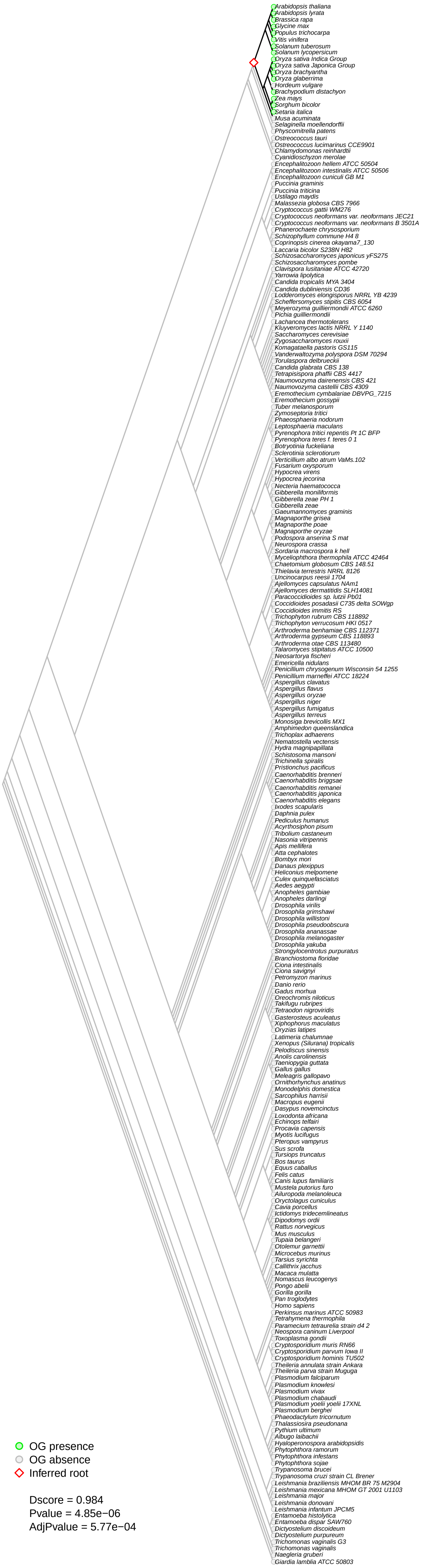
Arabidopsis thaliana
Arabidopsis lyrata
Brassica rapa
Glycine max
Populus trichocarpa
Vitis vinifera
Solanum tuberosum
Solanum lycopersicum
Oryza sativa Indica Group
Oryza sativa Japonica Group
Oryza brachyantha
Oryza glaberrima
Hordeum vulgare
Brachypodium distachyon
Zea mays
Sorghum bicolor
Setaria italica
Musa acuminata
Selaginella moellendorffii
Physcomitrella patens
Ostreococcus tauri
Ostreococcus lucimarinus CCE9901
Chlamydomonas reinhardtii
Cyanidioschyzon merolae
Encephalitozoon hellem ATCC 50504
Encephalitozoon intestinalis ATCC 50506
Encephalitozoon cuniculi GB M1
Puccinia graminis
Puccinia triticina
Ustilago maydis
Malassezia globosa CBS 7966
Cryptococcus gattii WM276
Cryptococcus neoformans var. neoformans JEC21
Cryptococcus neoformans var. neoformans B 3501A
Phanerochaete chrysosporium
Schizophyllum commune H4 8
Coprinopsis cinerea okayama7_130
Laccaria bicolor S238N H82
Schizosaccharomyces japonicus yFS275
Schizosaccharomyces pombe
Clavispora lusitaniae ATCC 42720
Yarrowia lipolytica
Candida tropicalis MYA 3404
Candida dubliniensis CD36
Lodderomyces elongisporus NRRL YB 4239
Scheffersomyces stipitis CBS 6054
Meyerozyma guilliermondii ATCC 6260
Pichia guilliermondii
Lachancea thermotolerans
Kluyveromyces lactis NRRL Y 1140
Saccharomyces cerevisiae
Zygosaccharomyces rouxii
Komagataella pastoris GS115
Vanderwaltozyma polyspora DSM 70294
Torulaspora delbrueckii
Candida glabrata CBS 138
Tetrapisispora phaffii CBS 4417
Naumovozyma dairenensis CBS 421
Naumovozyma castellii CBS 4309
Eremothecium cymbalariae DBVPG_7215
Eremothecium gossypii
Tuber melanosporum
Zymoseptoria tritici
Phaeosphaeria nodorum
Leptosphaeria maculans
Pyrenophora tritici repentis Pt 1C BFP
Pyrenophora teres f. teres 0 1
Botryotinia fuckeliana
Sclerotinia sclerotiorum
Verticillium albo atrum VaMs.102
Fusarium oxysporum
Hypocrea virens
Hypocrea jecorina
Nectria haematococca
Gibberella moniliformis
Gibberella zeae PH 1
Gibberella zeae
Gaeumannomyces graminis
Magnaporthe grisea
Magnaporthe poae
Magnaporthe oryzae
Podospora anserina S mat
Neurospora crassa
Sordaria macrospora k hell
Myceliophthora thermophila ATCC 42464
Chaetomium globosum CBS 148.51
Thielavia terrestris NRRL 8126
Uncinocarpus reesii 1704
Ajellomyces capsulatus NAm1
Ajellomyces dermatitidis SLH14081
Paracoccidioides sp. lutzii Pb01
Coccidioides posadasii C735 delta SOWgp
Coccidioides immitis RS
Trichophyton rubrum CBS 118892
Trichophyton verrucosum HKI 0517
Arthroderma benhamiae CBS 112371
Arthroderma gypseum CBS 118893
Arthroderma otae CBS 113480
Talaromyces stipitatus ATCC 10500
Neosartorya fischeri
Emericella nidulans
Penicillium chrysogenum Wisconsin 54 1255
Penicillium marneffei ATCC 18224
Aspergillus clavatus
Aspergillus flavus
Aspergillus oryzae
Aspergillus niger
Aspergillus fumigatus
Aspergillus terreus
Monosiga brevicollis MX1
Amphimedon queenslandica
Trichoplax adhaerens
Nematostella vectensis
Hydra magnipapillata
Schistosoma mansoni
Trichinella spiralis
Pristionchus pacificus
Caenorhabditis brenneri
Caenorhabditis briggsae
Caenorhabditis remanei
Caenorhabditis japonica
Caenorhabditis elegans
Ixodes scapularis
Daphnia pulex
Pediculus humanus
Acyrthosiphon pisum
Tribolium castaneum
Nasonia vitripennis
Apis mellifera
Atta cephalotes
Bombyx mori
Danaus plexippus
Heliconius melpomene
Culex quinquefasciatus
Aedes aegypti
Anopheles gambiae
Anopheles darlingi
Drosophila virilis
Drosophila grimshawi
Drosophila willistoni
Drosophila pseudoobscura
Drosophila ananassae
Drosophila melanogaster
Drosophila yakuba
Strongylocentrotus purpuratus
Branchiostoma floridae
Ciona intestinalis
Ciona savignyi
Petromyzon marinus
Danio rerio
Gadus morhua
Oreochromis niloticus
Takifugu rubripes
Tetraodon nigroviridis
Gasterosteus aculeatus
Xiphophorus maculatus
Oryzias latipes
Latimeria chalumnae
Xenopus (Silurana) tropicalis
Pelodiscus sinensis
Anolis carolinensis
Taeniopygia guttata
Gallus gallus
Meleagris gallopavo
Ornithorhynchus anatinus
Monodelphis domestica
Sarcophilus harrisii
Macropus eugenii
Dasypus novemcinctus
Loxodonta africana
Echinops telfairi
Procavia capensis
Myotis lucifugus
Pteropus vampyrus
Sus scrofa
Tursiops truncatus
Bos taurus
Equus caballus
Felis catus
Canis lupus familiaris
Mustela putorius furo
Ailuropoda melanoleuca
Oryctolagus cuniculus
Cavia porcellus
Ictidomys tridecemlineatus
Dipodomys ordii
Rattus norvegicus
Mus musculus
Tupaia belangeri
Otolemur garnettii
Microcebus murinus
Tarsius syrichta
Callithrix jacchus
Macaca mulatta
Nomascus leucogenys
Pongo abelii
Gorilla gorilla
Pan troglodytes
Homo sapiens
Perkinsus marinus ATCC 50983
Tetrahymena thermophila
Paramecium tetraurelia strain d4 2
Neospora caninum Liverpool
Toxoplasma gondii
Cryptosporidium muris RN66
Cryptosporidium parvum Iowa II
Cryptosporidium hominis TU502
Theileria annulata strain Ankara
Theileria parva strain Muguga
Plasmodium falciparum
Plasmodium knowlesi
Plasmodium vivax
Plasmodium chabaudi
Plasmodium yoelii yoelii 17XNL
Plasmodium berghei
Phaeodactylum tricornutum
Thalassiosira pseudonana
Pythium ultimum
Albugo laibachii
Hyaloperonospora arabidopsidis
Phytophthora ramorum
Phytophthora infestans
Phytophthora sojae
Trypanosoma brucei
Trypanosoma cruzi strain CL Brener
Leishmania braziliensis MHOM BR 75 M2904
Leishmania mexicana MHOM GT 2001 U1103
Leishmania major
Leishmania donovani
Leishmania infantum JPCM5
Entamoeba histolytica
Entamoeba dispar SAW760
Dictyostelium discoideum
Dictyostelium purpureum
Trichomonas vaginalis G3
Trichomonas vaginalis
Naegleria gruberi
Giardia lamblia ATCC 50803
OG presence
OG absence
Inferred root
Dscore = 0.984
Pvalue = 4.85e−06
AdjPvalue = 5.77e−04

NOG128539

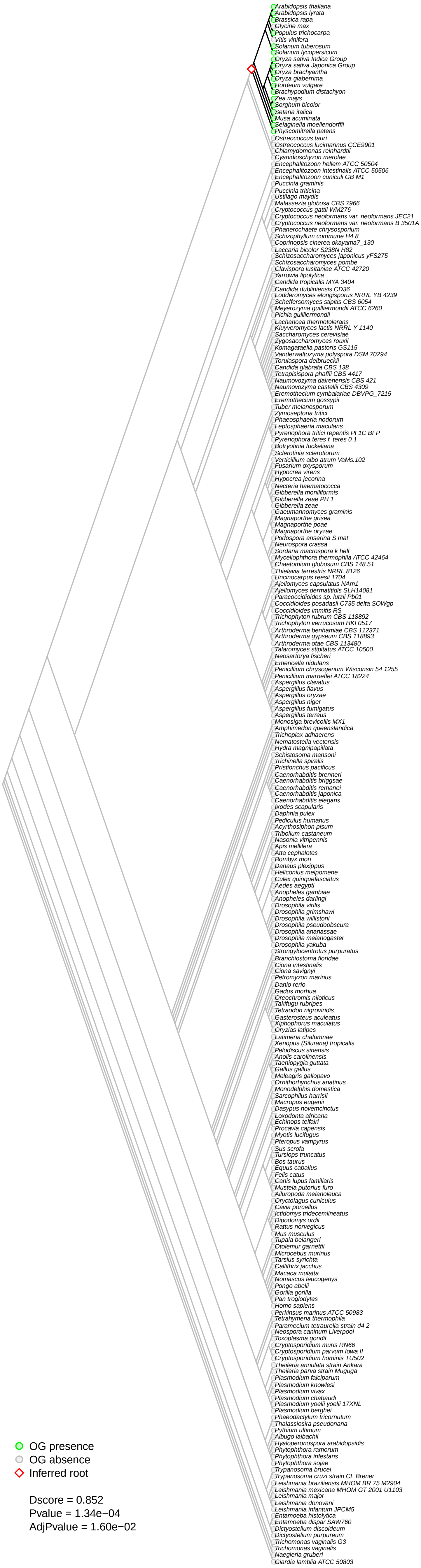
Arabidopsis thaliana
Arabidopsis lyrata
Brassica rapa
Glycine max
Populus trichocarpa
Vitis vinifera
Solanum tuberosum
Solanum lycopersicum
Oryza sativa Indica Group
Oryza sativa Japonica Group
Oryza brachyantha
Oryza glaberrima
Hordeum vulgare
Brachypodium distachyon
Zea mays
Sorghum bicolor
Setaria italica
Musa acuminata
Selaginella moellendorffii
Physcomitrella patens
Ostreococcus tauri
Ostreococcus lucimarinus CCE9901
Chlamydomonas reinhardtii
Cyanidioschyzon merolae
Encephalitozoon hellem ATCC 50504
Encephalitozoon intestinalis ATCC 50506
Encephalitozoon cuniculi GB M1
Puccinia graminis
Puccinia triticina
Ustilago maydis
Malassezia globosa CBS 7966
Cryptococcus gattii WM276
Cryptococcus neoformans var. neoformans JEC21
Cryptococcus neoformans var. neoformans B 3501A
Phanerochaete chrysosporium
Schizophyllum commune H4 8
Coprinopsis cinerea okayama7_130
Laccaria bicolor S238N H82
Schizosaccharomyces japonicus yFS275
Schizosaccharomyces pombe
Clavispora lusitaniae ATCC 42720
Yarrowia lipolytica
Candida tropicalis MYA 3404
Candida dubliniensis CD36
Lodderomyces elongisporus NRRL YB 4239
Scheffersomyces stipitis CBS 6054
Meyerozyma guilliermondii ATCC 6260
Pichia guilliermondii
Lachancea thermotolerans
Kluyveromyces lactis NRRL Y 1140
Saccharomyces cerevisiae
Zygosaccharomyces rouxii
Komagataella pastoris GS115
Vanderwaltozyma polyspora DSM 70294
Torulaspora delbrueckii
Candida glabrata CBS 138
Tetrapisispora phaffii CBS 4417
Naumovozyma dairenensis CBS 421
Naumovozyma castellii CBS 4309
Eremothecium cymbalariae DBVPG_7215
Eremothecium gossypii
Tuber melanosporum
Zymoseptoria tritici
Phaeosphaeria nodorum
Leptosphaeria maculans
Pyrenophora tritici repentis Pt 1C BFP
Pyrenophora teres f. teres 0 1
Botryotinia fuckeliana
Sclerotinia sclerotiorum
Verticillium albo atrum VaMs.102
Fusarium oxysporum
Hypocrea virens
Hypocrea jecorina
Nectria haematococca
Gibberella moniliformis
Gibberella zeae PH 1
Gibberella zeae
Gaeumannomyces graminis
Magnaporthe grisea
Magnaporthe poae
Magnaporthe oryzae
Podospora anserina S mat
Neurospora crassa
Sordaria macrospora k hell
Myceliophthora thermophila ATCC 42464
Chaetomium globosum CBS 148.51
Thielavia terrestris NRRL 8126
Uncinocarpus reesii 1704
Ajellomyces capsulatus NAm1
Ajellomyces dermatitidis SLH14081
Paracoccidioides sp. lutzii Pb01
Coccidioides posadasii C735 delta SOWgp
Coccidioides immitis RS
Trichophyton rubrum CBS 118892
Trichophyton verrucosum HKI 0517
Arthroderma benhamiae CBS 112371
Arthroderma gypseum CBS 118893
Arthroderma otae CBS 113480
Talaromyces stipitatus ATCC 10500
Neosartorya fischeri
Emericella nidulans
Penicillium chrysogenum Wisconsin 54 1255
Penicillium marneffei ATCC 18224
Aspergillus clavatus
Aspergillus flavus
Aspergillus oryzae
Aspergillus niger
Aspergillus fumigatus
Aspergillus terreus
Monosiga brevicollis MX1
Amphimedon queenslandica
Trichoplax adhaerens
Nematostella vectensis
Hydra magnipapillata
Schistosoma mansoni
Trichinella spiralis
Pristionchus pacificus
Caenorhabditis brenneri
Caenorhabditis briggsae
Caenorhabditis remanei
Caenorhabditis japonica
Caenorhabditis elegans
Ixodes scapularis
Daphnia pulex
Pediculus humanus
Acyrthosiphon pisum
Tribolium castaneum
Nasonia vitripennis
Apis mellifera
Atta cephalotes
Bombyx mori
Danaus plexippus
Heliconius melpomene
Culex quinquefasciatus
Aedes aegypti
Anopheles gambiae
Anopheles darlingi
Drosophila virilis
Drosophila grimshawi
Drosophila willistoni
Drosophila pseudoobscura
Drosophila ananassae
Drosophila melanogaster
Drosophila yakuba
Strongylocentrotus purpuratus
Branchiostoma floridae
Ciona intestinalis
Ciona savignyi
Petromyzon marinus
Danio rerio
Gadus morhua
Oreochromis niloticus
Takifugu rubripes
Tetraodon nigroviridis
Gasterosteus aculeatus
Xiphophorus maculatus
Oryzias latipes
Latimeria chalumnae
Xenopus (Silurana) tropicalis
Pelodiscus sinensis
Anolis carolinensis
Taeniopygia guttata
Gallus gallus
Meleagris gallopavo
Ornithorhynchus anatinus
Monodelphis domestica
Sarcophilus harrisii
Macropus eugenii
Dasypus novemcinctus
Loxodonta africana
Echinops telfairi
Procavia capensis
Myotis lucifugus
Pteropus vampyrus
Sus scrofa
Tursiops truncatus
Bos taurus
Equus caballus
Felis catus
Canis lupus familiaris
Mustela putorius furo
Ailuropoda melanoleuca
Oryctolagus cuniculus
Cavia porcellus
Ictidomys tridecemlineatus
Dipodomys ordii
Rattus norvegicus
Mus musculus
Tupaia belangeri
Otolemur garnettii
Microcebus murinus
Tarsius syrichta
Callithrix jacchus
Macaca mulatta
Nomascus leucogenys
Pongo abelii
Gorilla gorilla
Pan troglodytes
Homo sapiens
Perkinsus marinus ATCC 50983
Tetrahymena thermophila
Paramecium tetraurelia strain d4 2
Neospora caninum Liverpool
Toxoplasma gondii
Cryptosporidium muris RN66
Cryptosporidium parvum Iowa II
Cryptosporidium hominis TU502
Theileria annulata strain Ankara
Theileria parva strain Muguga
Plasmodium falciparum
Plasmodium knowlesi
Plasmodium vivax
Plasmodium chabaudi
Plasmodium yoelii yoelii 17XNL
Plasmodium berghei
Phaeodactylum tricornutum
Thalassiosira pseudonana
Pythium ultimum
Albugo laibachii
Hyaloperonospora arabidopsidis
Phytophthora ramorum
Phytophthora infestans
Phytophthora sojae
Trypanosoma brucei
Trypanosoma cruzi strain CL Brener
Leishmania braziliensis MHOM BR 75 M2904
Leishmania mexicana MHOM GT 2001 U1103
Leishmania major
Leishmania donovani
Leishmania infantum JPCM5
Entamoeba histolytica
Entamoeba dispar SAW760
Dictyostelium discoideum
Dictyostelium purpureum
Trichomonas vaginalis G3
Trichomonas vaginalis
Naegleria gruberi
Giardia lamblia ATCC 50803
OG presence
OG absence
Inferred root
Dscore = 0.852
Pvalue = 1.34e−04
AdjPvalue = 1.60e−02

NOG129341

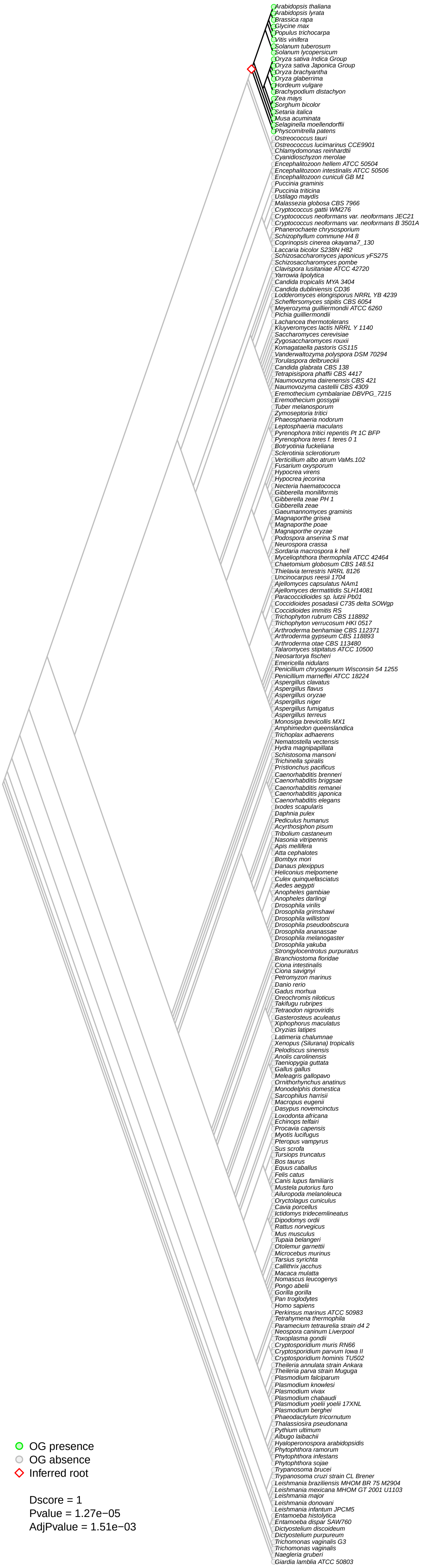

Arabidopsis thaliana
Arabidopsis lyrata
Brassica rapa
Glycine max
Populus trichocarpa
Vitis vinifera
Solanum tuberosum
Solanum lycopersicum
Oryza sativa Indica Group
Oryza sativa Japonica Group
Oryza brachyantha
Oryza glaberrima
Hordeum vulgare
Brachypodium distachyon
Zea mays
Sorghum bicolor
Setaria italica
Musa acuminata
Selaginella moellendorffii
Physcomitrella patens
Ostreococcus tauri
Ostreococcus lucimarinus CCE9901
Chlamydomonas reinhardtii
Cyanidioschyzon merolae
Encephalitozoon hellem ATCC 50504
Encephalitozoon intestinalis ATCC 50506
Encephalitozoon cuniculi GB M1
Puccinia graminis
Puccinia triticina
Ustilago maydis
Malassezia globosa CBS 7966
Cryptococcus gattii WM276
Cryptococcus neoformans var. neoformans JEC21
Cryptococcus neoformans var. neoformans B 3501A
Phanerochaete chrysosporium
Schizophyllum commune H4 8
Coprinopsis cinerea okayama7_130
Laccaria bicolor S238N H82
Schizosaccharomyces japonicus yFS275
Schizosaccharomyces pombe
Clavispora lusitaniae ATCC 42720
Yarrowia lipolytica
Candida tropicalis MYA 3404
Candida dubliniensis CD36
Lodderomyces elongisporus NRRL YB 4239
Scheffersomyces stipitis CBS 6054
Meyerozyma guilliermondii ATCC 6260
Pichia guilliermondii
Lachancea thermotolerans
Kluyveromyces lactis NRRL Y 1140
Saccharomyces cerevisiae
Zygosaccharomyces rouxii
Komagataella pastoris GS115
Vanderwaltozyma polyspora DSM 70294
Torulaspora delbrueckii
Candida glabrata CBS 138
Tetrapisispora phaffii CBS 4417
Naumovozyma dairenensis CBS 421
Naumovozyma castellii CBS 4309
Eremothecium cymbalariae DBVPG_7215
Eremothecium gossypii
Tuber melanosporum
Zymoseptoria tritici
Phaeosphaeria nodorum
Leptosphaeria maculans
Pyrenophora tritici repentis Pt 1C BFP
Pyrenophora teres f. teres 0 1
Botryotinia fuckeliana
Sclerotinia sclerotiorum
Verticillium albo atrum VaMs.102
Fusarium oxysporum
Hypocrea virens
Hypocrea jecorina
Nectria haematococca
Gibberella moniliformis
Gibberella zeae PH 1
Gibberella zeae
Gaeumannomyces graminis
Magnaporthe grisea
Magnaporthe poae
Magnaporthe oryzae
Podospora anserina S mat
Neurospora crassa
Sordaria macrospora k hell
Myceliophthora thermophila ATCC 42464
Chaetomium globosum CBS 148.51
Thielavia terrestris NRRL 8126
Uncinocarpus reesii 1704
Ajellomyces capsulatus NAm1
Ajellomyces dermatitidis SLH14081
Paracoccidioides sp. lutzii Pb01
Coccidioides posadasii C735 delta SOWgp
Coccidioides immitis RS
Trichophyton rubrum CBS 118892
Trichophyton verrucosum HKI 0517
Arthroderma benhamiae CBS 112371
Arthroderma gypseum CBS 118893
Arthroderma otae CBS 113480
Talaromyces stipitatus ATCC 10500
Neosartorya fischeri
Emericella nidulans
Penicillium chrysogenum Wisconsin 54 1255
Penicillium marneffei ATCC 18224
Aspergillus clavatus
Aspergillus flavus
Aspergillus oryzae
Aspergillus niger
Aspergillus fumigatus
Aspergillus terreus
Monosiga brevicollis MX1
Amphimedon queenslandica
Trichoplax adhaerens
Nematostella vectensis
Hydra magnipapillata
Schistosoma mansoni
Trichinella spiralis
Pristionchus pacificus
Caenorhabditis brenneri
Caenorhabditis briggsae
Caenorhabditis remanei
Caenorhabditis japonica
Caenorhabditis elegans
Ixodes scapularis
Daphnia pulex
Pediculus humanus
Acyrthosiphon pisum
Tribolium castaneum
Nasonia vitripennis
Apis mellifera
Atta cephalotes
Bombyx mori
Danaus plexippus
Heliconius melpomene
Culex quinquefasciatus
Aedes aegypti
Anopheles gambiae
Anopheles darlingi
Drosophila virilis
Drosophila grimshawi
Drosophila willistoni
Drosophila pseudoobscura
Drosophila ananassae
Drosophila melanogaster
Drosophila yakuba
Strongylocentrotus purpuratus
Branchiostoma floridae
Ciona intestinalis
Ciona savignyi
Petromyzon marinus
Danio rerio
Gadus morhua
Oreochromis niloticus
Takifugu rubripes
Tetraodon nigroviridis
Gasterosteus aculeatus
Xiphophorus maculatus
Oryzias latipes
Latimeria chalumnae
Xenopus (Silurana) tropicalis
Pelodiscus sinensis
Anolis carolinensis
Taeniopygia guttata
Gallus gallus
Meleagris gallopavo
Ornithorhynchus anatinus
Monodelphis domestica
Sarcophilus harrisii
Macropus eugenii
Dasypus novemcinctus
Loxodonta africana
Echinops telfairi
Procavia capensis
Myotis lucifugus
Pteropus vampyrus
Sus scrofa
Tursiops truncatus
Bos taurus
Equus caballus
Felis catus
Canis lupus familiaris
Mustela putorius furo
Ailuropoda melanoleuca
Oryctolagus cuniculus
Cavia porcellus
Ictidomys tridecemlineatus
Dipodomys ordii
Rattus norvegicus
Mus musculus
Tupaia belangeri
Otolemur garnettii
Microcebus murinus
Tarsius syrichta
Callithrix jacchus
Macaca mulatta
Nomascus leucogenys
Pongo abelii
Gorilla gorilla
Pan troglodytes
Homo sapiens
Perkinsus marinus ATCC 50983
Tetrahymena thermophila
Paramecium tetraurelia strain d4 2
Neospora caninum Liverpool
Toxoplasma gondii
Cryptosporidium muris RN66
Cryptosporidium parvum Iowa II
Cryptosporidium hominis TU502
Theileria annulata strain Ankara
Theileria parva strain Muguga
Plasmodium falciparum
Plasmodium knowlesi
Plasmodium vivax
Plasmodium chabaudi
Plasmodium yoelii yoelii 17XNL
Plasmodium berghei
Phaeodactylum tricornutum
Thalassiosira pseudonana
Pythium ultimum
Albugo laibachii
Hyaloperonospora arabidopsidis
Phytophthora ramorum
Phytophthora infestans
Phytophthora sojae
Trypanosoma brucei
Trypanosoma cruzi strain CL Brener
Leishmania braziliensis MHOM BR 75 M2904
Leishmania mexicana MHOM GT 2001 U1103
Leishmania major
Leishmania donovani
Leishmania infantum JPCM5
Entamoeba histolytica
Entamoeba dispar SAW760
Dictyostelium discoideum
Dictyostelium purpureum
Trichomonas vaginalis G3
Trichomonas vaginalis
Naegleria gruberi
Giardia lamblia ATCC 50803
OG presence
OG absence
Inferred root
Dscore = 1
Pvalue = 1.27e−05
AdjPvalue = 1.51e−03

NOG129430

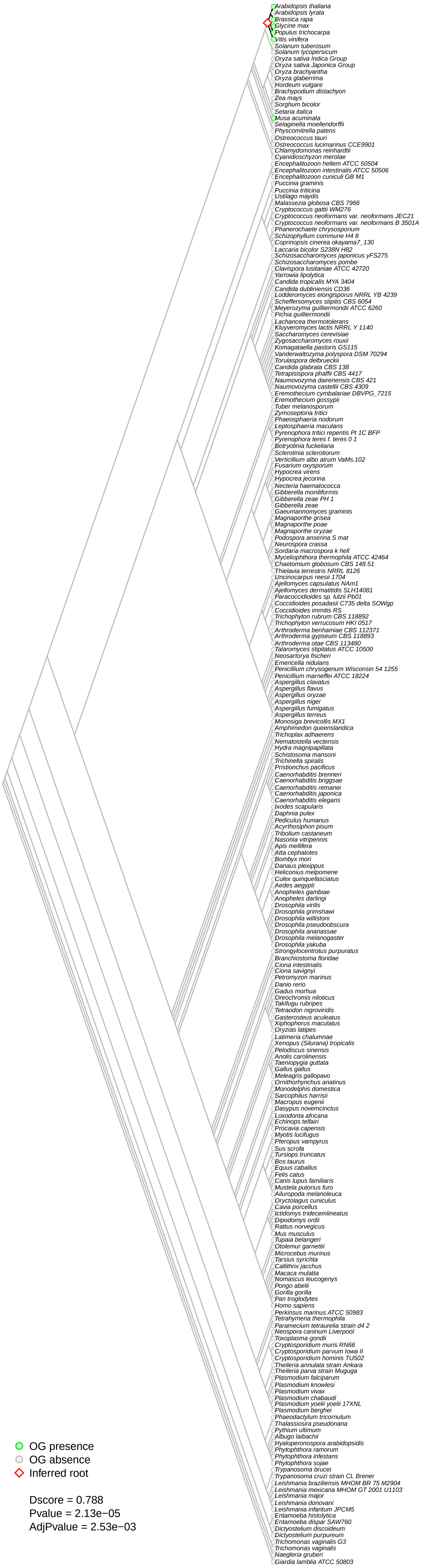

OG presence
OG absence
Inferred root

Dscore = 0.788
Pvalue = 2.13e−05
AdjPvalue = 2.53e−03

NOG129978

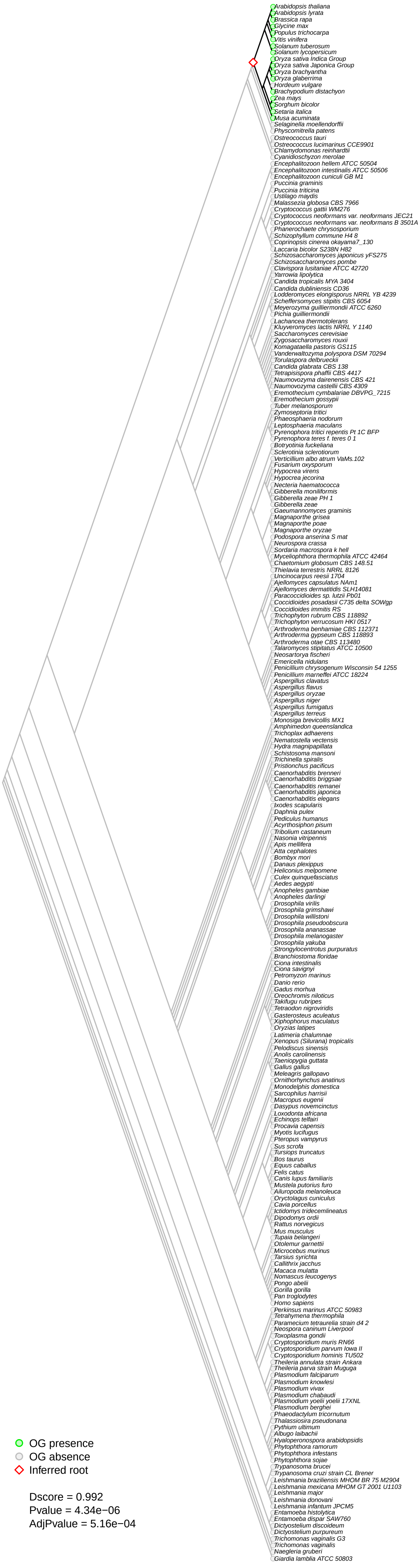

Arabidopsis thaliana
Arabidopsis lyrata
Brassica rapa
Glycine max
Populus trichocarpa
Vitis vinifera
Solanum tuberosum
Solanum lycopersicum
Oryza sativa Indica Group
Oryza sativa Japonica Group
Oryza brachyantha
Oryza glaberrima
Hordeum vulgare
Brachypodium distachyon
Zea mays
Sorghum bicolor
Setaria italica
Musa acuminata
Selaginella moellendorffii
Physcomitrella patens
Ostreococcus tauri
Ostreococcus lucimarinus CCE9901
Chlamydomonas reinhardtii
Cyanidioschyzon merolae
Encephalitozoon hellem ATCC 50504
Encephalitozoon intestinalis ATCC 50506
Encephalitozoon cuniculi GB M1
Puccinia graminis
Puccinia triticina
Ustilago maydis
Malassezia globosa CBS 7966
Cryptococcus gattii WM276
Cryptococcus neoformans var. neoformans JEC21
Cryptococcus neoformans var. neoformans B 3501A
Phanerochaete chrysosporium
Schizophyllum commune H4 8
Coprinopsis cinerea okayama7_130
Laccaria bicolor S238N H82
Schizosaccharomyces japonicus yFS275
Schizosaccharomyces pombe
Clavispora lusitaniae ATCC 42720
Yarrowia lipolytica
Candida tropicalis MYA 3404
Candida dubliniensis CD36
Lodderomyces elongisporus NRRL YB 4239
Scheffersomyces stipitis CBS 6054
Meyerozyma guilliermondii ATCC 6260
Pichia guilliermondii
Lachancea thermotolerans
Kluyveromyces lactis NRRL Y 1140
Saccharomyces cerevisiae
Zygosaccharomyces rouxii
Komagataella pastoris GS115
Vanderwaltozyma polyspora DSM 70294
Torulaspora delbrueckii
Candida glabrata CBS 138
Tetrapisispora phaffii CBS 4417
Naumovozyma dairenensis CBS 421
Naumovozyma castellii CBS 4309
Eremothecium cymbalariae DBVPG_7215
Eremothecium gossypii
Tuber melanosporum
Zymoseptoria tritici
Phaeosphaeria nodorum
Leptosphaeria maculans
Pyrenophora tritici repentis Pt 1C BFP
Pyrenophora teres f. teres 0 1
Botryotinia fuckeliana
Sclerotinia sclerotiorum
Verticillium albo atrum VaMs.102
Fusarium oxysporum
Hypocrea virens
Hypocrea jecorina
Necteria haematococca
Gibberella moniliformis
Gibberella zeae PH 1
Gibberella zeae
Gaeumannomyces graminis
Magnaporthe grisea
Magnaporthe poae
Magnaporthe oryzae
Podospora anserina S mat
Neurospora crassa
Sordaria macrospora k hell
Myceliophthora thermophila ATCC 42464
Chaetomium globosum CBS 148.51
Thielavia terrestris NRRL 8126
Uncinocarpus reesii 1704
Ajellomyces capsulatus NAm1
Ajellomyces dermatitidis SLH14081
Paracoccidioides sp. lutzii Pb01
Coccidioides posadasii C735 delta SOWgp
Coccidioides immitis RS
Trichophyton rubrum CBS 118892
Trichophyton verrucosum HKI 0517
Arthroderma benhamiae CBS 112371
Arthroderma gypseum CBS 118893
Arthroderma otae CBS 113480
Talaromyces stipitatus ATCC 10500
Neosartorya fischeri
Emericella nidulans
Penicillium chrysogenum Wisconsin 54 1255
Penicillium marneffei ATCC 18224
Aspergillus clavatus
Aspergillus flavus
Aspergillus oryzae
Aspergillus niger
Aspergillus fumigatus
Aspergillus terreus
Monosiga brevicollis MX1
Amphimedon queenslandica
Trichoplax adhaerens
Nematostella vectensis
Hydra magnipapillata
Schistosoma mansoni
Trichinella spiralis
Pristionchus pacificus
Caenorhabditis brenneri
Caenorhabditis briggsae
Caenorhabditis remanei
Caenorhabditis japonica
Caenorhabditis elegans
Ixodes scapularis
Daphnia pulex
Pediculus humanus
Acyrthosiphon pisum
Tribolium castaneum
Nasonia vitripennis
Apis mellifera
Atta cephalotes
Bombyx mori
Danaus plexippus
Heliconius melpomene
Culex quinquefasciatus
Aedes aegypti
Anopheles gambiae
Anopheles darlingi
Drosophila virilis
Drosophila grimshawi
Drosophila willistoni
Drosophila pseudoobscura
Drosophila ananassae
Drosophila melanogaster
Drosophila yakuba
Strongylocentrotus purpuratus
Branchiostoma floridae
Ciona intestinalis
Ciona savignyi
Petromyzon marinus
Danio rerio
Gadus morhua
Oreochromis niloticus
Takifugu rubripes
Tetraodon nigroviridis
Gasterosteus aculeatus
Xiphophorus maculatus
Oryzias latipes
Latimeria chalumnae
Xenopus (Silurana) tropicalis
Pelodiscus sinensis
Anolis carolinensis
Taeniopygia guttata
Gallus gallus
Meleagris gallopavo
Ornithorhynchus anatinus
Monodelphis domestica
Sarcophilus harrisii
Macropus eugenii
Dasypus novemcinctus
Loxodonta africana
Echinops telfairi
Procavia capensis
Myotis lucifugus
Pteropus vampyrus
Sus scrofa
Tursiops truncatus
Bos taurus
Equus caballus
Felis catus
Canis lupus familiaris
Mustela putorius furo
Ailuropoda melanoleuca
Oryctolagus cuniculus
Cavia porcellus
Ictidomys tridecemlineatus
Dipodomys ordii
Rattus norvegicus
Mus musculus
Tupaia belangeri
Otolemur garnettii
Microcebus murinus
Tarsius syrichta
Callithrix jacchus
Macaca mulatta
Nomascus leucogenys
Pongo abelii
Gorilla gorilla
Pan troglodytes
Homo sapiens
Perkinsus marinus ATCC 50983
Tetrahymena thermophila
Paramecium tetraurelia strain d4 2
Neospora caninum Liverpool
Toxoplasma gondii
Cryptosporidium muris RN66
Cryptosporidium parvum Iowa II
Cryptosporidium hominis TU502
Theileria annulata strain Ankara
Theileria parva strain Muguga
Plasmodium falciparum
Plasmodium knowlesi
Plasmodium vivax
Plasmodium chabaudi
Plasmodium yoelii yoelii 17XNL
Plasmodium berghei
Phaeodactylum tricornutum
Thalassiosira pseudonana
Pythium ultimum
Albugo laibachii
Hyaloperonospora arabidopsidis
Phytophthora ramorum
Phytophthora infestans
Phytophthora sojae
Trypanosoma brucei
Trypanosoma cruzi strain CL Brener
Leishmania braziliensis MHOM BR 75 M2904
Leishmania mexicana MHOM GT 2001 U1103
Leishmania major
Leishmania donovani
Leishmania infantum JPCM5
Entamoeba histolytica
Entamoeba dispar SAW760
Dictyostelium discoideum
Dictyostelium purpureum
Trichomonas vaginalis G3
Trichomonas vaginalis
Naegleria gruberi
Giardia lamblia ATCC 50803
OG presence
OG absence
Inferred root
Dscore = 0.992
Pvalue = 4.34e−06
AdjPvalue = 5.16e−04

NOG130232

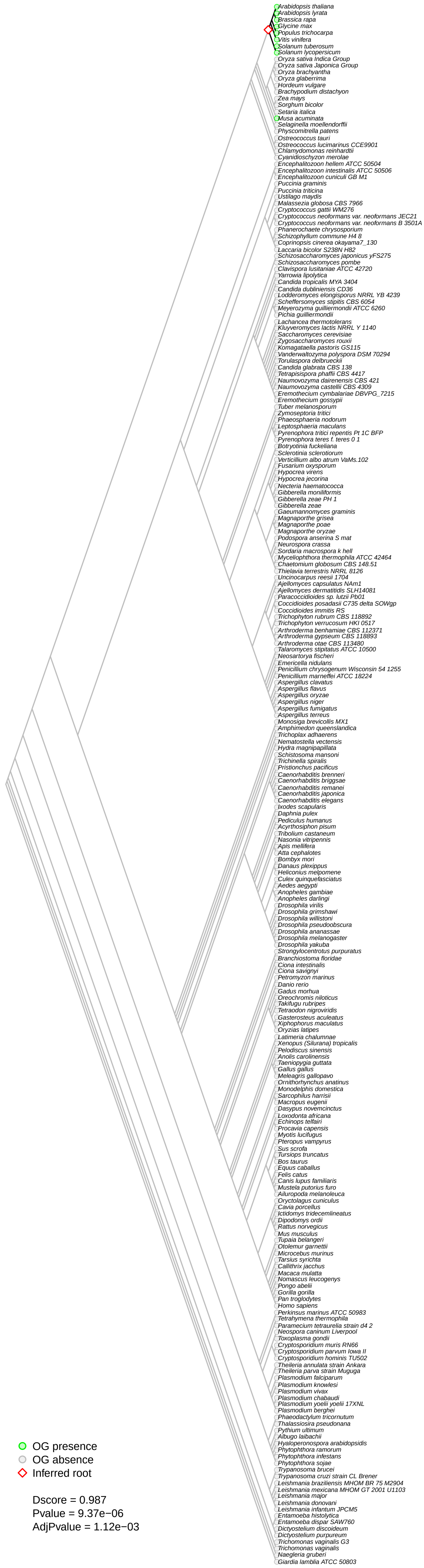

Arabidopsis thaliana
Arabidopsis lyrata
Brassica rapa
Glycine max
Populus trichocarpa
Vitis vinifera
Solanum tuberosum
Solanum lycopersicum
Oryza sativa Indica Group
Oryza sativa Japonica Group
Oryza brachyantha
Oryza glaberrima
Hordeum vulgare
Brachypodium distachyon
Zea mays
Sorghum bicolor
Setaria italica
Musa acuminata
Selaginella moellendorffii
Physcomitrella patens
Ostreococcus tauri
Ostreococcus lucimarinus CCE9901
Chlamydomonas reinhardtii
Cyanidioschyzon merolae
Encephalitozoon hellem ATCC 50504
Encephalitozoon intestinalis ATCC 50506
Encephalitozoon cuniculi GB M1
Puccinia graminis
Puccinia triticina
Ustilago maydis
Malassezia globosa CBS 7966
Cryptococcus gattii WM276
Cryptococcus neoformans var. neoformans JEC21
Cryptococcus neoformans var. neoformans B 3501A
Phanerochaete chrysosporium
Schizophyllum commune H4 8
Coprinopsis cinerea okayama7_130
Laccaria bicolor S238N H82
Schizosaccharomyces japonicus yFS275
Schizosaccharomyces pombe
Clavispora lusitaniae ATCC 42720
Yarrowia lipolytica
Candida tropicalis MYA 3404
Candida dubliniensis CD36
Lodderomyces elongisporus NRRL YB 4239
Scheffersomyces stipitis CBS 6054
Meyerozyma guilliermondii ATCC 6260
Pichia guilliermondii
Lachancea thermotolerans
Kluyveromyces lactis NRRL Y 1140
Saccharomyces cerevisiae
Zygosaccharomyces rouxii
Komagataella pastoris GS115
Vanderwaltozyma polyspora DSM 70294
Torulaspora delbrueckii
Candida glabrata CBS 138
Tetrapisispora phaffii CBS 4417
Naumovozyma dairenensis CBS 421
Naumovozyma castellii CBS 4309
Eremothecium cymbalariae DBVPG_7215
Eremothecium gossypii
Tuber melanosporum
Zymoseptoria tritici
Phaeosphaeria nodorum
Leptosphaeria maculans
Pyrenophora tritici repentis Pt 1C BFP
Pyrenophora teres f. teres 0 1
Botryotinia fuckeliana
Sclerotinia sclerotiorum
Verticillium albo atrum VaMs.102
Fusarium oxysporum
Hypocrea virens
Hypocrea jecorina
Nectria haematococca
Gibberella moniliformis
Gibberella zeae PH 1
Gibberella zeae
Gaeumannomyces graminis
Magnaporthe grisea
Magnaporthe poae
Magnaporthe oryzae
Podospora anserina S mat
Neurospora crassa
Sordaria macrospora k hell
Myceliophthora thermophila ATCC 42464
Chaetomium globosum CBS 148.51
Thielavia terrestris NRRL 8126
Uncinocarpus reesii 1704
Ajellomyces capsulatus NAm1
Ajellomyces dermatitidis SLH14081
Paracoccidioides sp. lutzii Pb01
Coccidioides posadasii C735 delta SOWgp
Coccidioides immitis RS
Trichophyton rubrum CBS 118892
Trichophyton verrucosum HKI 0517
Arthroderma benhamiae CBS 112371
Arthroderma gypseum CBS 118893
Arthroderma otae CBS 113480
Talaromyces stipitatus ATCC 10500
Neosartorya fischeri
Emericella nidulans
Penicillium chrysogenum Wisconsin 54 1255
Penicillium marneffei ATCC 18224
Aspergillus clavatus
Aspergillus flavus
Aspergillus oryzae
Aspergillus niger
Aspergillus fumigatus
Aspergillus terreus
Monosiga brevicollis MX1
Amphimedon queenslandica
Trichoplax adhaerens
Nematostella vectensis
Hydra magnipapillata
Schistosoma mansoni
Trichinella spiralis
Pristionchus pacificus
Caenorhabditis brenneri
Caenorhabditis briggsae
Caenorhabditis remanei
Caenorhabditis japonica
Caenorhabditis elegans
Ixodes scapularis
Daphnia pulex
Pediculus humanus
Acyrthosiphon pisum
Tribolium castaneum
Nasonia vitripennis
Apis mellifera
Atta cephalotes
Bombyx mori
Danaus plexippus
Heliconius melpomene
Culex quinquefasciatus
Aedes aegypti
Anopheles gambiae
Anopheles darlingi
Drosophila virilis
Drosophila grimshawi
Drosophila willistoni
Drosophila pseudoobscura
Drosophila ananassae
Drosophila melanogaster
Drosophila yakuba
Strongylocentrotus purpuratus
Branchiostoma floridae
Ciona intestinalis
Ciona savignyi
Petromyzon marinus
Danio rerio
Gadus morhua
Oreochromis niloticus
Takifugu rubripes
Tetraodon nigroviridis
Gasterosteus aculeatus
Xiphophorus maculatus
Oryzias latipes
Latimeria chalumnae
Xenopus (Silurana) tropicalis
Pelodiscus sinensis
Anolis carolinensis
Taeniopygia guttata
Gallus gallus
Meleagris gallopavo
Ornithorhynchus anatinus
Monodelphis domestica
Sarcophilus harrisii
Macropus eugenii
Dasypus novemcinctus
Loxodonta africana
Echinops telfairi
Procavia capensis
Myotis lucifugus
Pteropus vampyrus
Sus scrofa
Tursiops truncatus
Bos taurus
Equus caballus
Felis catus
Canis lupus familiaris
Mustela putorius furo
Ailuropoda melanoleuca
Oryctolagus cuniculus
Cavia porcellus
Ictidomys tridecemlineatus
Dipodomys ordii
Rattus norvegicus
Mus musculus
Tupaia belangeri
Otolemur garnettii
Microcebus murinus
Tarsius syrichta
Callithrix jacchus
Macaca mulatta
Nomascus leucogenys
Pongo abelii
Gorilla gorilla
Pan troglodytes
Homo sapiens
Perkinsus marinus ATCC 50983
Tetrahymena thermophila
Paramecium tetraurelia strain d4 2
Neospora caninum Liverpool
Toxoplasma gondii
Cryptosporidium muris RN66
Cryptosporidium parvum Iowa II
Cryptosporidium hominis TU502
Theileria annulata strain Ankara
Theileria parva strain Muguga
Plasmodium falciparum
Plasmodium knowlesi
Plasmodium vivax
Plasmodium chabaudi
Plasmodium yoelii yoelii 17XNL
Plasmodium berghei
Phaeodactylum tricornutum
Thalassiosira pseudonana
Pythium ultimum
Albugo laibachii
Hyaloperonospora arabidopsidis
Phytophthora ramorum
Phytophthora infestans
Phytophthora sojae
Trypanosoma brucei
Trypanosoma cruzi strain CL Brener
Leishmania braziliensis MHOM BR 75 M2904
Leishmania mexicana MHOM GT 2001 U1103
Leishmania major
Leishmania donovani
Leishmania infantum JPCM5
Entamoeba histolytica
Entamoeba dispar SAW760
Dictyostelium discoideum
Dictyostelium purpureum
Trichomonas vaginalis G3
Trichomonas vaginalis
Naegleria gruberi
Giardia lamblia ATCC 50803
OG presence
OG absence
Inferred root
Dscore = 0.987
Pvalue = 9.37e−06
AdjPvalue = 1.12e−03

NOG130428

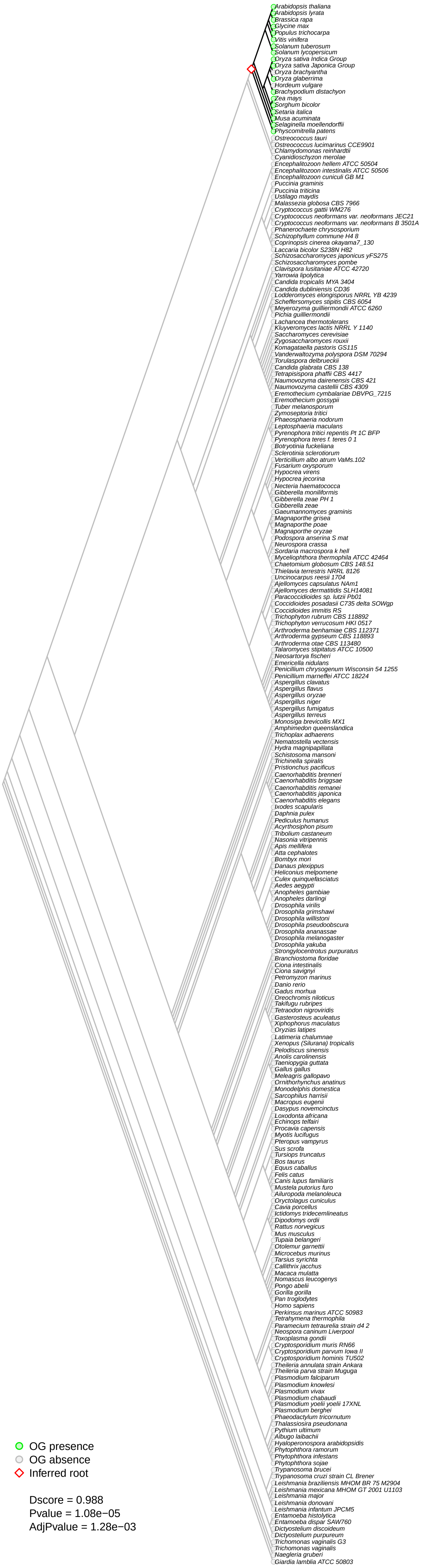

Arabidopsis thaliana
Arabidopsis lyrata
Brassica rapa
Glycine max
Populus trichocarpa
Vitis vinifera
Solanum tuberosum
Solanum lycopersicum
Oryza sativa Indica Group
Oryza sativa Japonica Group
Oryza brachyantha
Oryza glaberrima
Hordeum vulgare
Brachypodium distachyon
Zea mays
Sorghum bicolor
Setaria italica
Musa acuminata
Selaginella moellendorffii
Physcomitrella patens
Ostreococcus tauri
Ostreococcus lucimarinus CCE9901
Chlamydomonas reinhardtii
Cyanidioschyzon merolae
Encephalitozoon hellem ATCC 50504
Encephalitozoon intestinalis ATCC 50506
Encephalitozoon cuniculi GB M1
Puccinia graminis
Puccinia triticina
Ustilago maydis
Malassezia globosa CBS 7966
Cryptococcus gattii WM276
Cryptococcus neoformans var. neoformans JEC21
Cryptococcus neoformans var. neoformans B 3501A
Phanerochaete chrysosporium
Schizophyllum commune H4 8
Coprinopsis cinerea okayama7_130
Laccaria bicolor S238N H82
Schizosaccharomyces japonicus yFS275
Schizosaccharomyces pombe
Clavispora lusitaniae ATCC 42720
Yarrowia lipolytica
Candida tropicalis MYA 3404
Candida dubliniensis CD36
Lodderomyces elongisporus NRRL YB 4239
Scheffersomyces stipitis CBS 6054
Meyerozyma guilliermondii ATCC 6260
Pichia guilliermondii
Lachancea thermotolerans
Kluyveromyces lactis NRRL Y 1140
Saccharomyces cerevisiae
Zygosaccharomyces rouxii
Komagataella pastoris GS115
Vanderwaltozyma polyspora DSM 70294
Torulaspora delbrueckii
Candida glabrata CBS 138
Tetrapisispora phaffii CBS 4417
Naumovozyma dairenensis CBS 421
Naumovozyma castellii CBS 4309
Eremothecium cymbalariae DBVPG_7215
Eremothecium gossypii
Tuber melanosporum
Zymoseptoria tritici
Phaeosphaeria nodorum
Leptosphaeria maculans
Pyrenophora tritici repentis Pt 1C BFP
Pyrenophora teres f. teres 0 1
Botryotinia fuckeliana
Sclerotinia sclerotiorum
Verticillium albo atrum VaMs.102
Fusarium oxysporum
Hypocrea virens
Hypocrea jecorina
Nectria haematococca
Gibberella moniliformis
Gibberella zeae PH 1
Gibberella zeae
Gaeumannomyces graminis
Magnaporthe grisea
Magnaporthe poae
Magnaporthe oryzae
Podospora anserina S mat
Neurospora crassa
Sordaria macrospora k hell
Myceliophthora thermophila ATCC 42464
Chaetomium globosum CBS 148.51
Thielavia terrestris NRRL 8126
Uncinocarpus reesii 1704
Ajellomyces capsulatus NAm1
Ajellomyces dermatitidis SLH14081
Paracoccidioides sp. lutzii Pb01
Coccidioides posadasii C735 delta SOWgp
Coccidioides immitis RS
Trichophyton rubrum CBS 118892
Trichophyton verrucosum HKI 0517
Arthroderma benhamiae CBS 112371
Arthroderma gypseum CBS 118893
Arthroderma otae CBS 113480
Talaromyces stipitatus ATCC 10500
Neosartorya fischeri
Emericella nidulans
Penicillium chrysogenum Wisconsin 54 1255
Penicillium marneffei ATCC 18224
Aspergillus clavatus
Aspergillus flavus
Aspergillus oryzae
Aspergillus niger
Aspergillus fumigatus
Aspergillus terreus
Monosiga brevicollis MX1
Amphimedon queenslandica
Trichoplax adhaerens
Nematostella vectensis
Hydra magnipapillata
Schistosoma mansoni
Trichinella spiralis
Pristionchus pacificus
Caenorhabditis brenneri
Caenorhabditis briggsae
Caenorhabditis remanei
Caenorhabditis japonica
Caenorhabditis elegans
Ixodes scapularis
Daphnia pulex
Pediculus humanus
Acyrthosiphon pisum
Tribolium castaneum
Nasonia vitripennis
Apis mellifera
Atta cephalotes
Bombyx mori
Danaus plexippus
Heliconius melpomene
Culex quinquefasciatus
Aedes aegypti
Anopheles gambiae
Anopheles darlingi
Drosophila virilis
Drosophila grimshawi
Drosophila willistoni
Drosophila pseudoobscura
Drosophila ananassae
Drosophila melanogaster
Drosophila yakuba
Strongylocentrotus purpuratus
Branchiostoma floridae
Ciona intestinalis
Ciona savignyi
Petromyzon marinus
Danio rerio
Gadus morhua
Oreochromis niloticus
Takifugu rubripes
Tetraodon nigroviridis
Gasterosteus aculeatus
Xiphophorus maculatus
Oryzias latipes
Latimeria chalumnae
Xenopus (Silurana) tropicalis
Pelodiscus sinensis
Anolis carolinensis
Taeniopygia guttata
Gallus gallus
Meleagris gallopavo
Ornithorhynchus anatinus
Monodelphis domestica
Sarcophilus harrisii
Macropus eugenii
Dasypus novemcinctus
Loxodonta africana
Echinops telfairi
Procavia capensis
Myotis lucifugus
Pteropus vampyrus
Sus scrofa
Tursiops truncatus
Bos taurus
Equus caballus
Felis catus
Canis lupus familiaris
Mustela putorius furo
Ailuropoda melanoleuca
Oryctolagus cuniculus
Cavia porcellus
Ictidomys tridecemlineatus
Dipodomys ordii
Rattus norvegicus
Mus musculus
Tupaia belangeri
Otolemur garnettii
Microcebus murinus
Tarsius syrichta
Callithrix jacchus
Macaca mulatta
Nomascus leucogenys
Pongo abelii
Gorilla gorilla
Pan troglodytes
Homo sapiens
Perkinsus marinus ATCC 50983
Tetrahymena thermophila
Paramecium tetraurelia strain d4 2
Neospora caninum Liverpool
Toxoplasma gondii
Cryptosporidium muris RN66
Cryptosporidium parvum Iowa II
Cryptosporidium hominis TU502
Theileria annulata strain Ankara
Theileria parva strain Muguga
Plasmodium falciparum
Plasmodium knowlesi
Plasmodium vivax
Plasmodium chabaudi
Plasmodium yoelii yoelii 17XNL
Plasmodium berghei
Phaeodactylum tricornutum
Thalassiosira pseudonana
Pythium ultimum
Albugo laibachii
Hyaloperonospora arabidopsidis
Phytophthora ramorum
Phytophthora infestans
Phytophthora sojae
Trypanosoma brucei
Trypanosoma cruzi strain CL Brener
Leishmania braziliensis MHOM BR 75 M2904
Leishmania mexicana MHOM GT 2001 U1103
Leishmania major
Leishmania donovani
Leishmania infantum JPCM5
Entamoeba histolytica
Entamoeba dispar SAW760
Dictyostelium discoideum
Dictyostelium purpureum
Trichomonas vaginalis G3
Trichomonas vaginalis
Naegleria gruberi
Giardia lamblia ATCC 50803
OG presence
OG absence
Inferred root
Dscore = 0.988
Pvalue = 1.08e−05
AdjPvalue = 1.28e−03

NOG13059

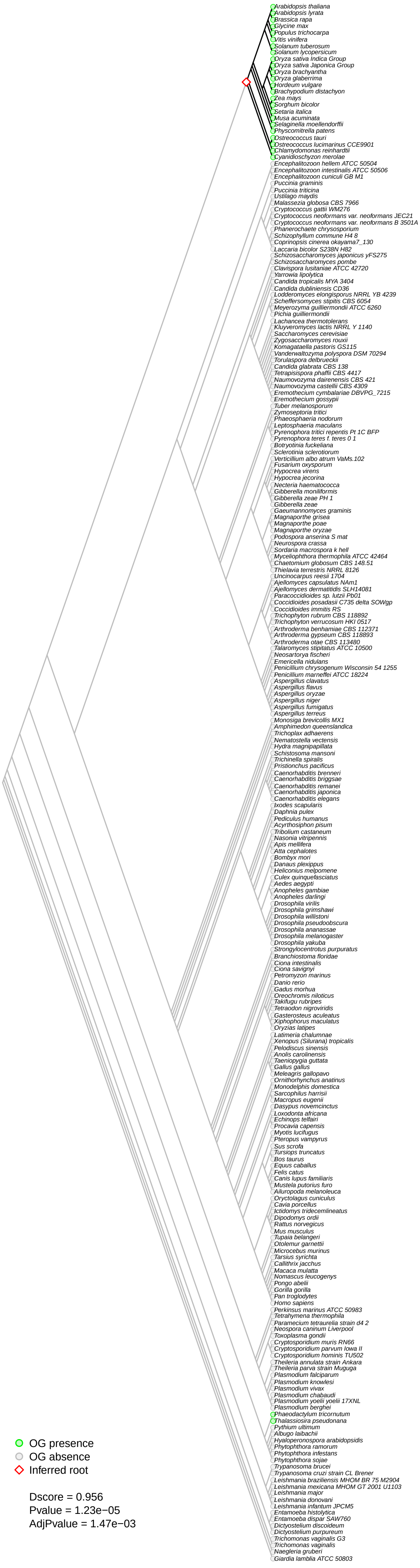

Arabidopsis thaliana
Arabidopsis lyrata
Brassica rapa
Glycine max
Populus trichocarpa
Vitis vinifera
Solanum tuberosum
Solanum lycopersicum
Oryza sativa Indica Group
Oryza sativa Japonica Group
Oryza brachyantha
Oryza glaberrima
Hordeum vulgare
Brachypodium distachyon
Zea mays
Sorghum bicolor
Setaria italica
Musa acuminata
Selaginella moellendorffii
Physcomitrella patens
Ostreococcus tauri
Ostreococcus lucimarinus CCE9901
Chlamydomonas reinhardtii
Cyanidioschyzon merolae
Encephalitozoon hellem ATCC 50504
Encephalitozoon intestinalis ATCC 50506
Encephalitozoon cuniculi GB M1
Puccinia graminis
Puccinia triticina
Ustilago maydis
Malassezia globosa CBS 7966
Cryptococcus gattii WM276
Cryptococcus neoformans var. neoformans JEC21
Cryptococcus neoformans var. neoformans B 3501A
Phanerochaete chrysosporium
Schizophyllum commune H4 8
Coprinopsis cinerea okayama7_130
Laccaria bicolor S238N H82
Schizosaccharomyces japonicus yFS275
Schizosaccharomyces pombe
Clavispora lusitaniae ATCC 42720
Yarrowia lipolytica
Candida tropicalis MYA 3404
Candida dubliniensis CD36
Lodderomyces elongisporus NRRL YB 4239
Scheffersomyces stipitis CBS 6054
Meyerozyma guilliermondii ATCC 6260
Pichia guilliermondii
Lachancea thermotolerans
Kluyveromyces lactis NRRL Y 1140
Saccharomyces cerevisiae
Zygosaccharomyces rouxii
Komagataella pastoris GS115
Vanderwaltozyma polyspora DSM 70294
Torulaspora delbrueckii
Candida glabrata CBS 138
Tetrapisispora phaffii CBS 4417
Naumovozyma dairenensis CBS 421
Naumovozyma castellii CBS 4309
Eremothecium cymbalariae DBVPG_7215
Eremothecium gossypii
Tuber melanosporum
Zymoseptoria tritici
Phaeosphaeria nodorum
Leptosphaeria maculans
Pyrenophora tritici repentis Pt 1C BFP
Pyrenophora teres f. teres 0 1
Botryotinia fuckeliana
Sclerotinia sclerotiorum
Verticillium albo atrum VaMs.102
Fusarium oxysporum
Hypocrea virens
Hypocrea jecorina
Nectria haematococca
Gibberella moniliformis
Gibberella zeae PH 1
Gibberella zeae
Gaeumannomyces graminis
Magnaporthe grisea
Magnaporthe poae
Magnaporthe oryzae
Podospora anserina S mat
Neurospora crassa
Sordaria macrospora k hell
Myceliophthora thermophila ATCC 42464
Chaetomium globosum CBS 148.51
Thielavia terrestris NRRL 8126
Uncinocarpus reesii 1704
Ajellomyces capsulatus NAm1
Ajellomyces dermatitidis SLH14081
Paracoccidioides sp. lutzii Pb01
Coccidioides posadasii C735 delta SOWgp
Coccidioides immitis RS
Trichophyton rubrum CBS 118892
Trichophyton verrucosum HKI 0517
Arthroderma benhamiae CBS 112371
Arthroderma gypseum CBS 118893
Arthroderma otae CBS 113480
Talaromyces stipitatus ATCC 10500
Neosartorya fischeri
Emericella nidulans
Penicillium chrysogenum Wisconsin 54 1255
Penicillium marneffei ATCC 18224
Aspergillus clavatus
Aspergillus flavus
Aspergillus oryzae
Aspergillus niger
Aspergillus fumigatus
Aspergillus terreus
Monosiga brevicollis MX1
Amphimedon queenslandica
Trichoplax adhaerens
Nematostella vectensis
Hydra magnipapillata
Schistosoma mansoni
Trichinella spiralis
Pristionchus pacificus
Caenorhabditis brenneri
Caenorhabditis briggsae
Caenorhabditis remanei
Caenorhabditis japonica
Caenorhabditis elegans
Ixodes scapularis
Daphnia pulex
Pediculus humanus
Acyrthosiphon pisum
Tribolium castaneum
Nasonia vitripennis
Apis mellifera
Atta cephalotes
Bombyx mori
Danaus plexippus
Heliconius melpomene
Culex quinquefasciatus
Aedes aegypti
Anopheles gambiae
Anopheles darlingi
Drosophila virilis
Drosophila grimshawi
Drosophila willistoni
Drosophila pseudoobscura
Drosophila ananassae
Drosophila melanogaster
Drosophila yakuba
Strongylocentrotus purpuratus
Branchiostoma floridae
Ciona intestinalis
Ciona savignyi
Petromyzon marinus
Danio rerio
Gadus morhua
Oreochromis niloticus
Takifugu rubripes
Tetraodon nigroviridis
Gasterosteus aculeatus
Xiphophorus maculatus
Oryzias latipes
Latimeria chalumnae
Xenopus (Silurana) tropicalis
Pelodiscus sinensis
Anolis carolinensis
Taeniopygia guttata
Gallus gallus
Meleagris gallopavo
Ornithorhynchus anatinus
Monodelphis domestica
Sarcophilus harrisii
Macropus eugenii
Dasypus novemcinctus
Loxodonta africana
Echinops telfairi
Procavia capensis
Myotis lucifugus
Pteropus vampyrus
Sus scrofa
Tursiops truncatus
Bos taurus
Equus caballus
Felis catus
Canis lupus familiaris
Mustela putorius furo
Ailuropoda melanoleuca
Oryctolagus cuniculus
Cavia porcellus
Ictidomys tridecemlineatus
Dipodomys ordii
Rattus norvegicus
Mus musculus
Tupaia belangeri
Otolemur garnettii
Microcebus murinus
Tarsius syrichta
Callithrix jacchus
Macaca mulatta
Nomascus leucogenys
Pongo abelii
Gorilla gorilla
Pan troglodytes
Homo sapiens
Perkinsus marinus ATCC 50983
Tetrahymena thermophila
Paramecium tetraurelia strain d4 2
Neospora caninum Liverpool
Toxoplasma gondii
Cryptosporidium muris RN66
Cryptosporidium parvum Iowa II
Cryptosporidium hominis TU502
Theileria annulata strain Ankara
Theileria parva strain Muguga
Plasmodium falciparum
Plasmodium knowlesi
Plasmodium vivax
Plasmodium chabaudi
Plasmodium yoelii yoelii 17XNL
Plasmodium berghei
Phaeodactylum tricornutum
Thalassiosira pseudonana
Pythium ultimum
Albugo laibachii
Hyaloperonospora arabidopsidis
Phytophthora ramorum
Phytophthora infestans
Phytophthora sojae
Trypanosoma brucei
Trypanosoma cruzi strain CL Brener
Leishmania braziliensis MHOM BR 75 M2904
Leishmania mexicana MHOM GT 2001 U1103
Leishmania major
Leishmania donovani
Leishmania infantum JPCM5
Entamoeba histolytica
Entamoeba dispar SAW760
Dictyostelium discoideum
Dictyostelium purpureum
Trichomonas vaginalis G3
Trichomonas vaginalis
Naegleria gruberi
Giardia lamblia ATCC 50803
OG presence
OG absence
Inferred root
Dscore = 0.956
Pvalue = 1.23e−05
AdjPvalue = 1.47e−03

NOG130945

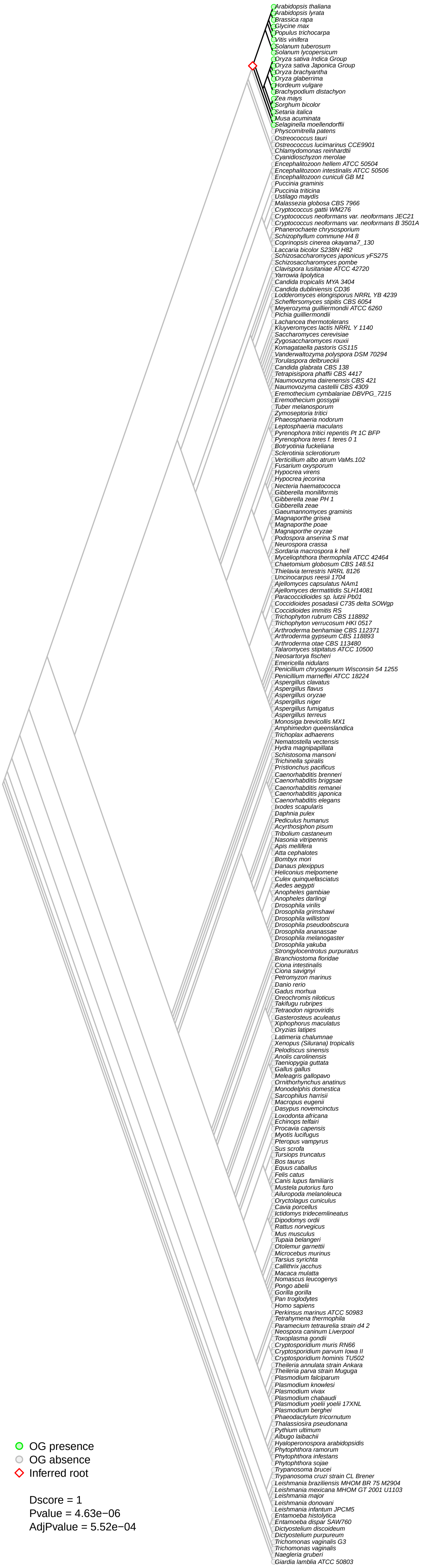

OG presence
OG absence
Inferred root

Dscore = 1
Pvalue = 4.63e−06
AdjPvalue = 5.52e−04

NOG131107

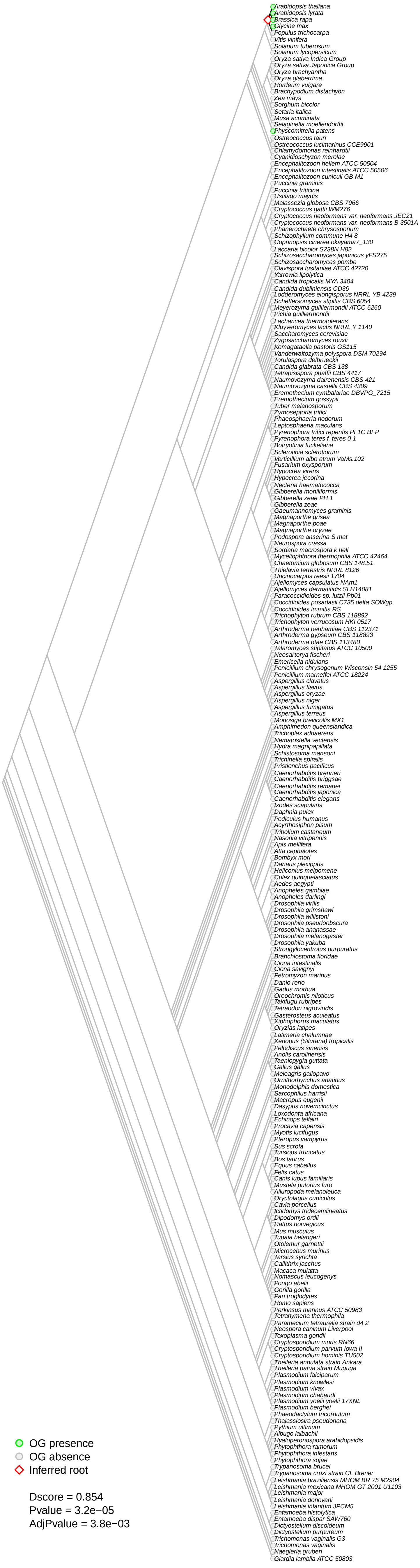

Dscore = 0.854
Pvalue = 3.2e−05
AdjPvalue = 3.8e−03

NOG131444

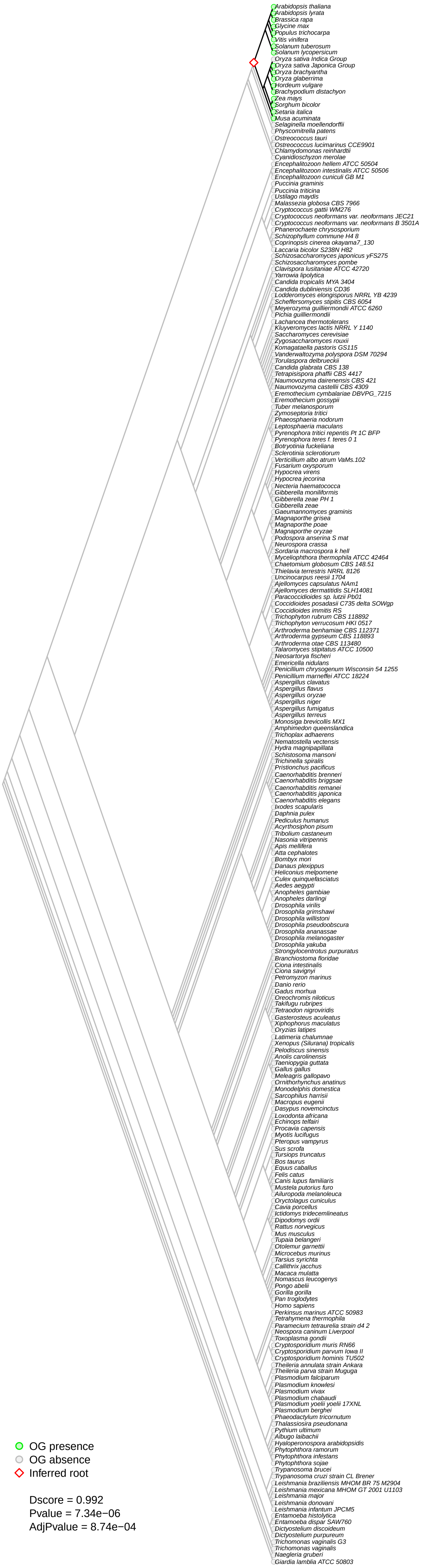
Arabidopsis thaliana
Arabidopsis lyrata
Brassica rapa
Glycine max
Populus trichocarpa
Vitis vinifera
Solanum tuberosum
Solanum lycopersicum
Oryza sativa Indica Group
Oryza sativa Japonica Group
Oryza brachyantha
Oryza glaberrima
Hordeum vulgare
Brachypodium distachyon
Zea mays
Sorghum bicolor
Setaria italica
Musa acuminata
Selaginella moellendorffii
Physcomitrella patens
Ostreococcus tauri
Ostreococcus lucimarinus CCE9901
Chlamydomonas reinhardtii
Cyanidioschyzon merolae
Encephalitozoon hellem ATCC 50504
Encephalitozoon intestinalis ATCC 50506
Encephalitozoon cuniculi GB M1
Puccinia graminis
Puccinia triticina
Ustilago maydis
Malassezia globosa CBS 7966
Cryptococcus gattii WM276
Cryptococcus neoformans var. neoformans JEC21
Cryptococcus neoformans var. neoformans B 3501A
Phanerochaete chrysosporium
Schizophyllum commune H4 8
Coprinopsis cinerea okayama7_130
Laccaria bicolor S238N H82
Schizosaccharomyces japonicus yFS275
Schizosaccharomyces pombe
Clavispora lusitaniae ATCC 42720
Yarrowia lipolytica
Candida tropicalis MYA 3404
Candida dubliniensis CD36
Lodderomyces elongisporus NRRL YB 4239
Scheffersomyces stipitis CBS 6054
Meyerozyma guilliermondii ATCC 6260
Pichia guilliermondii
Lachancea thermotolerans
Kluyveromyces lactis NRRL Y 1140
Saccharomyces cerevisiae
Zygosaccharomyces rouxii
Komagataella pastoris GS115
Vanderwaltozyma polyspora DSM 70294
Torulaspora delbrueckii
Candida glabrata CBS 138
Tetrapisispora phaffii CBS 4417
Naumovozyma dairenensis CBS 421
Naumovozyma castellii CBS 4309
Eremothecium cymbalariae DBVPG_7215
Eremothecium gossypii
Tuber melanosporum
Zymoseptoria tritici
Phaeosphaeria nodorum
Leptosphaeria maculans
Pyrenophora tritici repentis Pt 1C BFP
Pyrenophora teres f. teres 0 1
Botryotinia fuckeliana
Sclerotinia sclerotiorum
Verticillium albo atrum VaMs.102
Fusarium oxysporum
Hypocrea virens
Hypocrea jecorina
Nectria haematococca
Gibberella moniliformis
Gibberella zeae PH 1
Gibberella zeae
Gaeumannomyces graminis
Magnaporthe grisea
Magnaporthe poae
Magnaporthe oryzae
Podospora anserina S mat
Neurospora crassa
Sordaria macrospora k hell
Myceliophthora thermophila ATCC 42464
Chaetomium globosum CBS 148.51
Thielavia terrestris NRRL 8126
Uncinocarpus reesii 1704
Ajellomyces capsulatus NAm1
Ajellomyces dermatitidis SLH14081
Paracoccidioides sp. lutzii Pb01
Coccidioides posadasii C735 delta SOWgp
Coccidioides immitis RS
Trichophyton rubrum CBS 118892
Trichophyton verrucosum HKI 0517
Arthroderma benhamiae CBS 112371
Arthroderma gypseum CBS 118893
Arthroderma otae CBS 113480
Talaromyces stipitatus ATCC 10500
Neosartorya fischeri
Emericella nidulans
Penicillium chrysogenum Wisconsin 54 1255
Penicillium marneffei ATCC 18224
Aspergillus clavatus
Aspergillus flavus
Aspergillus oryzae
Aspergillus niger
Aspergillus fumigatus
Aspergillus terreus
Monosiga brevicollis MX1
Amphimedon queenslandica
Trichoplax adhaerens
Nematostella vectensis
Hydra magnipapillata
Schistosoma mansoni
Trichinella spiralis
Pristionchus pacificus
Caenorhabditis brenneri
Caenorhabditis briggsae
Caenorhabditis remanei
Caenorhabditis japonica
Caenorhabditis elegans
Ixodes scapularis
Daphnia pulex
Pediculus humanus
Acyrthosiphon pisum
Tribolium castaneum
Nasonia vitripennis
Apis mellifera
Atta cephalotes
Bombyx mori
Danaus plexippus
Heliconius melpomene
Culex quinquefasciatus
Aedes aegypti
Anopheles gambiae
Anopheles darlingi
Drosophila virilis
Drosophila grimshawi
Drosophila willistoni
Drosophila pseudoobscura
Drosophila ananassae
Drosophila melanogaster
Drosophila yakuba
Strongylocentrotus purpuratus
Branchiostoma floridae
Ciona intestinalis
Ciona savignyi
Petromyzon marinus
Danio rerio
Gadus morhua
Oreochromis niloticus
Takifugu rubripes
Tetraodon nigroviridis
Gasterosteus aculeatus
Xiphophorus maculatus
Oryzias latipes
Latimeria chalumnae
Xenopus (Silurana) tropicalis
Pelodiscus sinensis
Anolis carolinensis
Taeniopygia guttata
Gallus gallus
Meleagris gallopavo
Ornithorhynchus anatinus
Monodelphis domestica
Sarcophilus harrisii
Macropus eugenii
Dasypus novemcinctus
Loxodonta africana
Echinops telfairi
Procavia capensis
Myotis lucifugus
Pteropus vampyrus
Sus scrofa
Tursiops truncatus
Bos taurus
Equus caballus
Felis catus
Canis lupus familiaris
Mustela putorius furo
Ailuropoda melanoleuca
Oryctolagus cuniculus
Cavia porcellus
Ictidomys tridecemlineatus
Dipodomys ordii
Rattus norvegicus
Mus musculus
Tupaia belangeri
Otolemur garnettii
Microcebus murinus
Tarsius syrichta
Callithrix jacchus
Macaca mulatta
Nomascus leucogenys
Pongo abelii
Gorilla gorilla
Pan troglodytes
Homo sapiens
Perkinsus marinus ATCC 50983
Tetrahymena thermophila
Paramecium tetraurelia strain d4 2
Neospora caninum Liverpool
Toxoplasma gondii
Cryptosporidium muris RN66
Cryptosporidium parvum Iowa II
Cryptosporidium hominis TU502
Theileria annulata strain Ankara
Theileria parva strain Muguga
Plasmodium falciparum
Plasmodium knowlesi
Plasmodium vivax
Plasmodium chabaudi
Plasmodium yoelii yoelii 17XNL
Plasmodium berghei
Phaeodactylum tricornutum
Thalassiosira pseudonana
Pythium ultimum
Albugo laibachii
Hyaloperonospora arabidopsidis
Phytophthora ramorum
Phytophthora infestans
Phytophthora sojae
Trypanosoma brucei
Trypanosoma cruzi strain CL Brener
Leishmania braziliensis MHOM BR 75 M2904
Leishmania mexicana MHOM GT 2001 U1103
Leishmania major
Leishmania donovani
Leishmania infantum JPCM5
Entamoeba histolytica
Entamoeba dispar SAW760
Dictyostelium discoideum
Dictyostelium purpureum
Trichomonas vaginalis G3
Trichomonas vaginalis
Naegleria gruberi
Giardia lamblia ATCC 50803
OG presence
OG absence
Inferred root
Dscore = 0.992
Pvalue = 7.34e−06
AdjPvalue = 8.74e−04

NOG131641

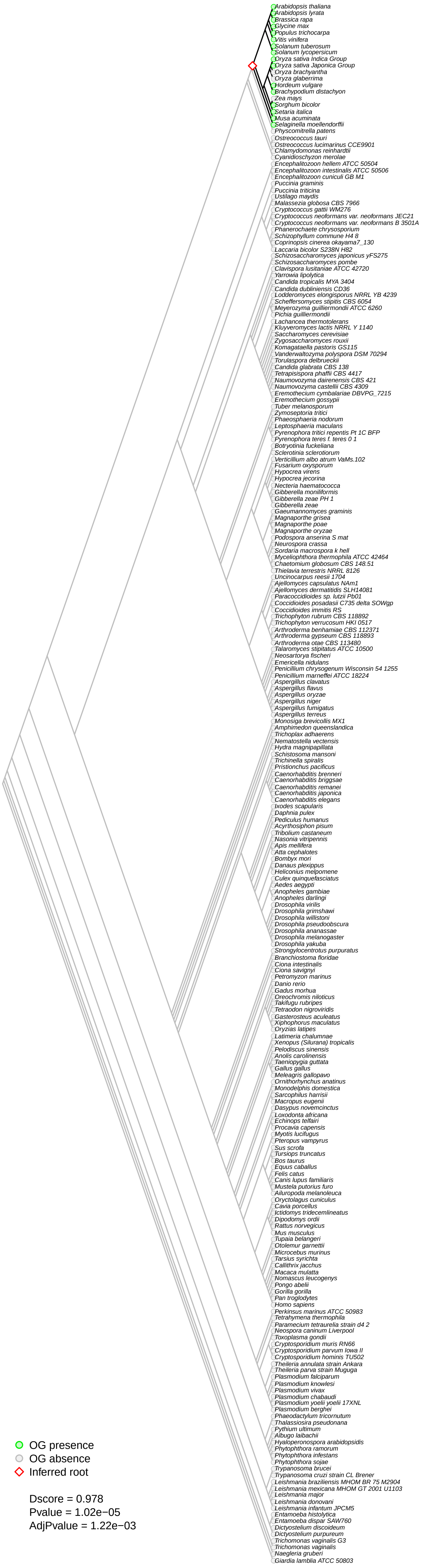

Arabidopsis thaliana
Arabidopsis lyrata
Brassica rapa
Glycine max
Populus trichocarpa
Vitis vinifera
Solanum tuberosum
Solanum lycopersicum
Oryza sativa Indica Group
Oryza sativa Japonica Group
Oryza brachyantha
Oryza glaberrima
Hordeum vulgare
Brachypodium distachyon
Zea mays
Sorghum bicolor
Setaria italica
Musa acuminata
Selaginella moellendorffii
Physcomitrella patens
Ostreococcus tauri
Ostreococcus lucimarinus CCE9901
Chlamydomonas reinhardtii
Cyanidioschyzon merolae
Encephalitozoon hellem ATCC 50504
Encephalitozoon intestinalis ATCC 50506
Encephalitozoon cuniculi GB M1
Puccinia graminis
Puccinia triticina
Ustilago maydis
Malassezia globosa CBS 7966
Cryptococcus gattii WM276
Cryptococcus neoformans var. neoformans JEC21
Cryptococcus neoformans var. neoformans B 3501A
Phanerochaete chrysosporium
Schizophyllum commune H4 8
Coprinopsis cinerea okayama7_130
Laccaria bicolor S238N H82
Schizosaccharomyces japonicus yFS275
Schizosaccharomyces pombe
Clavispora lusitaniae ATCC 42720
Yarrowia lipolytica
Candida tropicalis MYA 3404
Candida dubliniensis CD36
Lodderomyces elongisporus NRRL YB 4239
Scheffersomyces stipitis CBS 6054
Meyerozyma guilliermondii ATCC 6260
Pichia guilliermondii
Lachancea thermotolerans
Kluyveromyces lactis NRRL Y 1140
Saccharomyces cerevisiae
Zygosaccharomyces rouxii
Komagataella pastoris GS115
Vanderwaltozyma polyspora DSM 70294
Torulaspora delbrueckii
Candida glabrata CBS 138
Tetrapisispora phaffii CBS 4417
Naumovozyma dairenensis CBS 421
Naumovozyma castellii CBS 4309
Eremothecium cymbalariae DBVPG_7215
Eremothecium gossypii
Tuber melanosporum
Zymoseptoria tritici
Phaeosphaeria nodorum
Leptosphaeria maculans
Pyrenophora tritici repentis Pt 1C BFP
Pyrenophora teres f. teres 0 1
Botryotinia fuckeliana
Sclerotinia sclerotiorum
Verticillium albo atrum VaMs.102
Fusarium oxysporum
Hypocrea virens
Hypocrea jecorina
Nectria haematococca
Gibberella moniliformis
Gibberella zeae PH 1
Gibberella zeae
Gaeumannomyces graminis
Magnaporthe grisea
Magnaporthe poae
Magnaporthe oryzae
Podospora anserina S mat
Neurospora crassa
Sordaria macrospora k hell
Myceliophthora thermophila ATCC 42464
Chaetomium globosum CBS 148.51
Thielavia terrestris NRRL 8126
Uncinocarpus reesii 1704
Ajellomyces capsulatus NAm1
Ajellomyces dermatitidis SLH14081
Paracoccidioides sp. lutzii Pb01
Coccidioides posadasii C735 delta SOWgp
Coccidioides immitis RS
Trichophyton rubrum CBS 118892
Trichophyton verrucosum HKI 0517
Arthroderma benhamiae CBS 112371
Arthroderma gypseum CBS 118893
Arthroderma otae CBS 113480
Talaromyces stipitatus ATCC 10500
Neosartorya fischeri
Emericella nidulans
Penicillium chrysogenum Wisconsin 54 1255
Penicillium marneffei ATCC 18224
Aspergillus clavatus
Aspergillus flavus
Aspergillus oryzae
Aspergillus niger
Aspergillus fumigatus
Aspergillus terreus
Monosiga brevicollis MX1
Amphimedon queenslandica
Trichoplax adhaerens
Nematostella vectensis
Hydra magnipapillata
Schistosoma mansoni
Trichinella spiralis
Pristionchus pacificus
Caenorhabditis brenneri
Caenorhabditis briggsae
Caenorhabditis remanei
Caenorhabditis japonica
Caenorhabditis elegans
Ixodes scapularis
Daphnia pulex
Pediculus humanus
Acyrthosiphon pisum
Tribolium castaneum
Nasonia vitripennis
Apis mellifera
Atta cephalotes
Bombyx mori
Danaus plexippus
Heliconius melpomene
Culex quinquefasciatus
Aedes aegypti
Anopheles gambiae
Anopheles darlingi
Drosophila virilis
Drosophila grimshawi
Drosophila willistoni
Drosophila pseudoobscura
Drosophila ananassae
Drosophila melanogaster
Drosophila yakuba
Strongylocentrotus purpuratus
Branchiostoma floridae
Ciona intestinalis
Ciona savignyi
Petromyzon marinus
Danio rerio
Gadus morhua
Oreochromis niloticus
Takifugu rubripes
Tetraodon nigroviridis
Gasterosteus aculeatus
Xiphophorus maculatus
Oryzias latipes
Latimeria chalumnae
Xenopus (Silurana) tropicalis
Pelodiscus sinensis
Anolis carolinensis
Taeniopygia guttata
Gallus gallus
Meleagris gallopavo
Ornithorhynchus anatinus
Monodelphis domestica
Sarcophilus harrisii
Macropus eugenii
Dasypus novemcinctus
Loxodonta africana
Echinops telfairi
Procavia capensis
Myotis lucifugus
Pteropus vampyrus
Sus scrofa
Tursiops truncatus
Bos taurus
Equus caballus
Felis catus
Canis lupus familiaris
Mustela putorius furo
Ailuropoda melanoleuca
Oryctolagus cuniculus
Cavia porcellus
Ictidomys tridecemlineatus
Dipodomys ordii
Rattus norvegicus
Mus musculus
Tupaia belangeri
Otolemur garnettii
Microcebus murinus
Tarsius syrichta
Callithrix jacchus
Macaca mulatta
Nomascus leucogenys
Pongo abelii
Gorilla gorilla
Pan troglodytes
Homo sapiens
Perkinsus marinus ATCC 50983
Tetrahymena thermophila
Paramecium tetraurelia strain d4 2
Neospora caninum Liverpool
Toxoplasma gondii
Cryptosporidium muris RN66
Cryptosporidium parvum Iowa II
Cryptosporidium hominis TU502
Theileria annulata strain Ankara
Theileria parva strain Muguga
Plasmodium falciparum
Plasmodium knowlesi
Plasmodium vivax
Plasmodium chabaudi
Plasmodium yoelii yoelii 17XNL
Plasmodium berghei
Phaeodactylum tricornutum
Thalassiosira pseudonana
Pythium ultimum
Albugo laibachii
Hyaloperonospora arabidopsidis
Phytophthora ramorum
Phytophthora infestans
Phytophthora sojae
Trypanosoma brucei
Trypanosoma cruzi strain CL Brener
Leishmania braziliensis MHOM BR 75 M2904
Leishmania mexicana MHOM GT 2001 U1103
Leishmania major
Leishmania donovani
Leishmania infantum JPCM5
Entamoeba histolytica
Entamoeba dispar SAW760
Dictyostelium discoideum
Dictyostelium purpureum
Trichomonas vaginalis G3
Trichomonas vaginalis
Naegleria gruberi
Giardia lamblia ATCC 50803
OG presence
OG absence
Inferred root
Dscore = 0.978
Pvalue = 1.02e−05
AdjPvalue = 1.22e−03

NOG131772

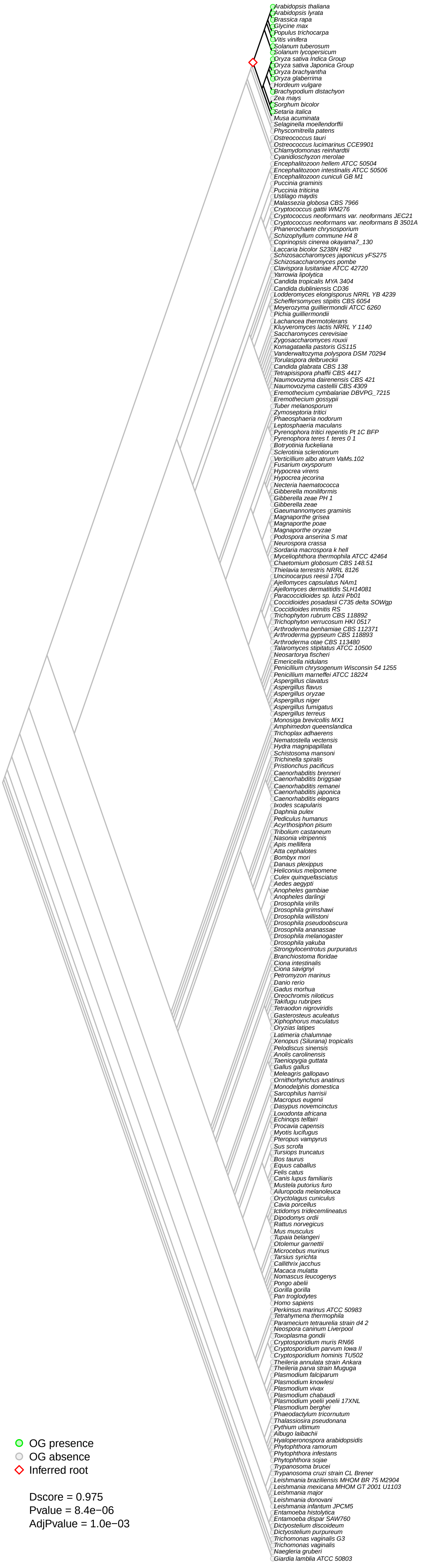

Arabidopsis thaliana
Arabidopsis lyrata
Brassica rapa
Glycine max
Populus trichocarpa
Vitis vinifera
Solanum tuberosum
Solanum lycopersicum
Oryza sativa Indica Group
Oryza sativa Japonica Group
Oryza brachyantha
Oryza glaberrima
Hordeum vulgare
Brachypodium distachyon
Zea mays
Sorghum bicolor
Setaria italica
Musa acuminata
Selaginella moellendorffii
Physcomitrella patens
Ostreococcus tauri
Ostreococcus lucimarinus CCE9901
Chlamydomonas reinhardtii
Cyanidioschyzon merolae
Encephalitozoon hellem ATCC 50504
Encephalitozoon intestinalis ATCC 50506
Encephalitozoon cuniculi GB M1
Puccinia graminis
Puccinia triticina
Ustilago maydis
Malassezia globosa CBS 7966
Cryptococcus gattii WM276
Cryptococcus neoformans var. neoformans JEC21
Cryptococcus neoformans var. neoformans B 3501A
Phanerochaete chrysosporium
Schizophyllum commune H4 8
Coprinopsis cinerea okayama7_130
Laccaria bicolor S238N H82
Schizosaccharomyces japonicus yFS275
Schizosaccharomyces pombe
Clavispora lusitaniae ATCC 42720
Yarrowia lipolytica
Candida tropicalis MYA 3404
Candida dubliniensis CD36
Lodderomyces elongisporus NRRL YB 4239
Scheffersomyces stipitis CBS 6054
Meyerozyma guilliermondii ATCC 6260
Pichia guilliermondii
Lachancea thermotolerans
Kluyveromyces lactis NRRL Y 1140
Saccharomyces cerevisiae
Zygosaccharomyces rouxii
Komagataella pastoris GS115
Vanderwaltozyma polyspora DSM 70294
Torulaspora delbrueckii
Candida glabrata CBS 138
Tetrapisispora phaffii CBS 4417
Naumovozyma dairenensis CBS 421
Naumovozyma castellii CBS 4309
Eremothecium cymbalariae DBVPG_7215
Eremothecium gossypii
Tuber melanosporum
Zymoseptoria tritici
Phaeosphaeria nodorum
Leptosphaeria maculans
Pyrenophora tritici repentis Pt 1C BFP
Pyrenophora teres f. teres 0 1
Botryotinia fuckeliana
Sclerotinia sclerotiorum
Verticillium albo atrum VaMs.102
Fusarium oxysporum
Hypocrea virens
Hypocrea jecorina
Necteria haematococca
Gibberella moniliformis
Gibberella zeae PH 1
Gibberella zeae
Gaeumannomyces graminis
Magnaporthe grisea
Magnaporthe poae
Magnaporthe oryzae
Podospora anserina S mat
Neurospora crassa
Sordaria macrospora k hell
Myceliophthora thermophila ATCC 42464
Chaetomium globosum CBS 148.51
Thielavia terrestris NRRL 8126
Uncinocarpus reesii 1704
Ajellomyces capsulatus NAm1
Ajellomyces dermatitidis SLH14081
Paracoccidioides sp. lutzii Pb01
Coccidioides posadasii C735 delta SOWgp
Coccidioides immitis RS
Trichophyton rubrum CBS 118892
Trichophyton verrucosum HKI 0517
Arthroderma benhamiae CBS 112371
Arthroderma gypseum CBS 118893
Arthroderma otae CBS 113480
Talaromyces stipitatus ATCC 10500
Neosartorya fischeri
Emericella nidulans
Penicillium chrysogenum Wisconsin 54 1255
Penicillium marneffei ATCC 18224
Aspergillus clavatus
Aspergillus flavus
Aspergillus oryzae
Aspergillus niger
Aspergillus fumigatus
Aspergillus terreus
Monosiga brevicollis MX1
Amphimedon queenslandica
Trichoplax adhaerens
Nematostella vectensis
Hydra magnipapillata
Schistosoma mansoni
Trichinella spiralis
Pristionchus pacificus
Caenorhabditis brenneri
Caenorhabditis briggsae
Caenorhabditis remanei
Caenorhabditis japonica
Caenorhabditis elegans
Ixodes scapularis
Daphnia pulex
Pediculus humanus
Acyrthosiphon pisum
Tribolium castaneum
Nasonia vitripennis
Apis mellifera
Atta cephalotes
Bombyx mori
Danaus plexippus
Heliconius melpomene
Culex quinquefasciatus
Aedes aegypti
Anopheles gambiae
Anopheles darlingi
Drosophila virilis
Drosophila grimshawi
Drosophila willistoni
Drosophila pseudoobscura
Drosophila ananassae
Drosophila melanogaster
Drosophila yakuba
Strongylocentrotus purpuratus
Branchiostoma floridae
Ciona intestinalis
Ciona savignyi
Petromyzon marinus
Danio rerio
Gadus morhua
Oreochromis niloticus
Takifugu rubripes
Tetraodon nigroviridis
Gasterosteus aculeatus
Xiphophorus maculatus
Oryzias latipes
Latimeria chalumnae
Xenopus (Silurana) tropicalis
Pelodiscus sinensis
Anolis carolinensis
Taeniopygia guttata
Gallus gallus
Meleagris gallopavo
Ornithorhynchus anatinus
Monodelphis domestica
Sarcophilus harrisii
Macropus eugenii
Dasypus novemcinctus
Loxodonta africana
Echinops telfairi
Procavia capensis
Myotis lucifugus
Pteropus vampyrus
Sus scrofa
Tursiops truncatus
Bos taurus
Equus caballus
Felis catus
Canis lupus familiaris
Mustela putorius furo
Ailuropoda melanoleuca
Oryctolagus cuniculus
Cavia porcellus
Ictidomys tridecemlineatus
Dipodomys ordii
Rattus norvegicus
Mus musculus
Tupaia belangeri
Otolemur garnettii
Microcebus murinus
Tarsius syrichta
Callithrix jacchus
Macaca mulatta
Nomascus leucogenys
Pongo abelii
Gorilla gorilla
Pan troglodytes
Homo sapiens
Perkinsus marinus ATCC 50983
Tetrahymena thermophila
Paramecium tetraurelia strain d4 2
Neospora caninum Liverpool
Toxoplasma gondii
Cryptosporidium muris RN66
Cryptosporidium parvum Iowa II
Cryptosporidium hominis TU502
Theileria annulata strain Ankara
Theileria parva strain Muguga
Plasmodium falciparum
Plasmodium knowlesi
Plasmodium vivax
Plasmodium chabaudi
Plasmodium yoelii yoelii 17XNL
Plasmodium berghei
Phaeodactylum tricornutum
Thalassiosira pseudonana
Pythium ultimum
Albugo laibachii
Hyaloperonospora arabidopsidis
Phytophthora ramorum
Phytophthora infestans
Phytophthora sojae
Trypanosoma brucei
Trypanosoma cruzi strain CL Brener
Leishmania braziliensis MHOM BR 75 M2904
Leishmania mexicana MHOM GT 2001 U1103
Leishmania major
Leishmania donovani
Leishmania infantum JPCM5
Entamoeba histolytica
Entamoeba dispar SAW760
Dictyostelium discoideum
Dictyostelium purpureum
Trichomonas vaginalis G3
Trichomonas vaginalis
Naegleria gruberi
Giardia lamblia ATCC 50803
OG presence
OG absence
Inferred root
Dscore = 0.975
Pvalue = 8.4e−06
AdjPvalue = 1.0e−03

NOG131810

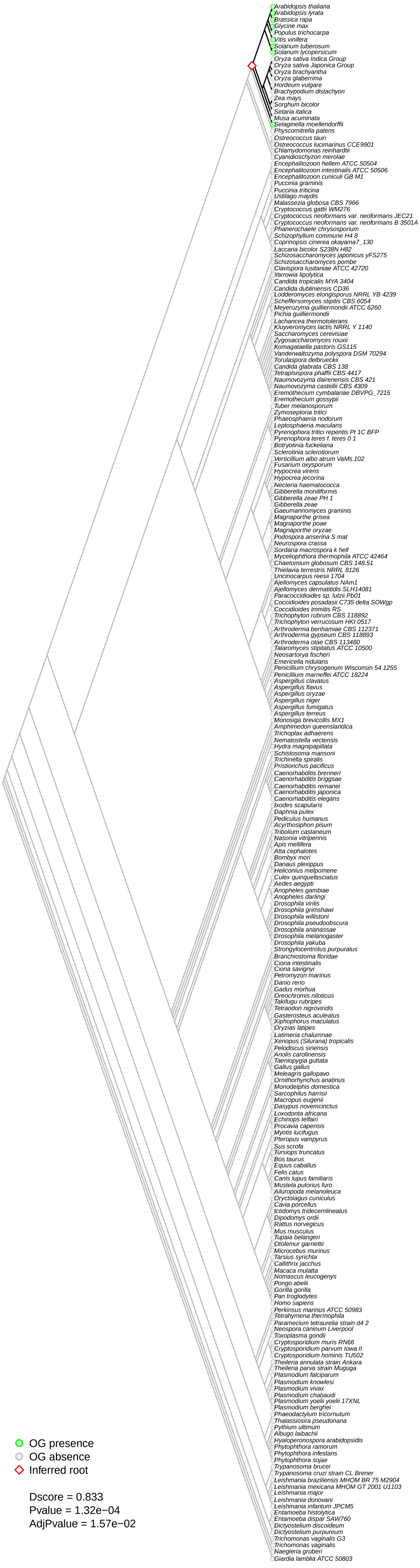
Arabidopsis thaliana
Arabidopsis lyrata
Brassica rapa
Glycine max
Populus trichocarpa
Vitis vinifera
Solanum tuberosum
Solanum lycopersicum
Oryza sativa Indica Group
Oryza sativa Japonica Group
Oryza brachyantha
Oryza glaberrima
Hordeum vulgare
Brachypodium distachyon
Zea mays
Sorghum bicolor
Setaria italica
Musa acuminata
Selaginella moellendorffii
Physcomitrella patens
Ostreococcus tauri
Ostreococcus lucimarinus CCE9901
Chlamydomonas reinhardtii
Cyanidioschyzon merolae
Encephalitozoon hellem ATCC 50504
Encephalitozoon intestinalis ATCC 50506
Encephalitozoon cuniculi GB M1
Puccinia graminis
Puccinia triticina
Ustilago maydis
Malassezia globosa CBS 7966
Cryptococcus gattii WM276
Cryptococcus neoformans var. neoformans JEC21
Cryptococcus neoformans var. neoformans B 3501A
Phanerochaete chrysosporium
Schizophyllum commune H4 8
Coprinopsis cinerea okayama7_130
Laccaria bicolor S238N H82
Schizosaccharomyces japonicus yFS275
Schizosaccharomyces pombe
Clavispora lusitaniae ATCC 42720
Yarrowia lipolytica
Candida tropicalis MYA 3404
Candida dubliniensis CD36
Lodderomyces elongisporus NRRL YB 4239
Scheffersomyces stipitis CBS 6054
Meyerozyma guilliermondii ATCC 6260
Pichia guilliermondii
Lachancea thermotolerans
Kluyveromyces lactis NRRL Y 1140
Saccharomyces cerevisiae
Zygosaccharomyces rouxii
Komagataella pastoris GS115
Vanderwaltozyma polyspora DSM 70294
Torulaspora delbrueckii
Candida glabrata CBS 138
Tetrapisispora phaffii CBS 4417
Naumovozyma dairenensis CBS 421
Naumovozyma castellii CBS 4309
Eremothecium cymbalariae DBVPG_7215
Eremothecium gossypii
Tuber melanosporum
Zymoseptoria tritici
Phaeosphaeria nodorum
Leptosphaeria maculans
Pyrenophora tritici repentis Pt 1C BFP
Pyrenophora teres f. teres 0 1
Botryotinia fuckeliana
Sclerotinia sclerotiorum
Verticillium albo atrum VaMs.102
Fusarium oxysporum
Hypocrea virens
Hypocrea jecorina
Nectria haematococca
Gibberella moniliformis
Gibberella zeae PH 1
Gibberella zeae
Gaeumannomyces graminis
Magnaporthe grisea
Magnaporthe poae
Magnaporthe oryzae
Podospora anserina S mat
Neurospora crassa
Sordaria macrospora k hell
Myceliophthora thermophila ATCC 42464
Chaetomium globosum CBS 148.51
Thielavia terrestris NRRL 8126
Uncinocarpus reesii 1704
Ajellomyces capsulatus NAm1
Ajellomyces dermatitidis SLH14081
Paracoccidioides sp. lutzii Pb01
Coccidioides posadasii C735 delta SOWgp
Coccidioides immitis RS
Trichophyton rubrum CBS 118892
Trichophyton verrucosum HKI 0517
Arthroderma benhamiae CBS 112371
Arthroderma gypseum CBS 118893
Arthroderma otae CBS 113480
Talaromyces stipitatus ATCC 10500
Neosartorya fischeri
Emericella nidulans
Penicillium chrysogenum Wisconsin 54 1255
Penicillium marneffei ATCC 18224
Aspergillus clavatus
Aspergillus flavus
Aspergillus oryzae
Aspergillus niger
Aspergillus fumigatus
Aspergillus terreus
Monosiga brevicollis MX1
Amphimedon queenslandica
Trichoplax adhaerens
Nematostella vectensis
Hydra magnipapillata
Schistosoma mansoni
Trichinella spiralis
Pristionchus pacificus
Caenorhabditis brenneri
Caenorhabditis briggsae
Caenorhabditis remanei
Caenorhabditis japonica
Caenorhabditis elegans
Ixodes scapularis
Daphnia pulex
Pediculus humanus
Acyrthosiphon pisum
Tribolium castaneum
Nasonia vitripennis
Apis mellifera
Atta cephalotes
Bombyx mori
Danaus plexippus
Heliconius melpomene
Culex quinquefasciatus
Aedes aegypti
Anopheles gambiae
Anopheles darlingi
Drosophila virilis
Drosophila grimshawi
Drosophila willistoni
Drosophila pseudoobscura
Drosophila ananassae
Drosophila melanogaster
Drosophila yakuba
Strongylocentrotus purpuratus
Branchiostoma floridae
Ciona intestinalis
Ciona savignyi
Petromyzon marinus
Danio rerio
Gadus morhua
Oreochromis niloticus
Takifugu rubripes
Tetraodon nigroviridis
Gasterosteus aculeatus
Xiphophorus maculatus
Oryzias latipes
Latimeria chalumnae
Xenopus (Silurana) tropicalis
Pelodiscus sinensis
Anolis carolinensis
Taeniopygia guttata
Gallus gallus
Meleagris gallopavo
Ornithorhynchus anatinus
Monodelphis domestica
Sarcophilus harrisii
Macropus eugenii
Dasypus novemcinctus
Loxodonta africana
Echinops telfairi
Procavia capensis
Myotis lucifugus
Pteropus vampyrus
Sus scrofa
Tursiops truncatus
Bos taurus
Equus caballus
Felis catus
Canis lupus familiaris
Mustela putorius furo
Ailuropoda melanoleuca
Oryctolagus cuniculus
Cavia porcellus
Ictidomys tridecemlineatus
Dipodomys ordii
Rattus norvegicus
Mus musculus
Tupaia belangeri
Otolemur garnettii
Microcebus murinus
Tarsius syrichta
Callithrix jacchus
Macaca mulatta
Nomascus leucogenys
Pongo abelii
Gorilla gorilla
Pan troglodytes
Homo sapiens
Perkinsus marinus ATCC 50983
Tetrahymena thermophila
Paramecium tetraurelia strain d4 2
Neospora caninum Liverpool
Toxoplasma gondii
Cryptosporidium muris RN66
Cryptosporidium parvum Iowa II
Cryptosporidium hominis TU502
Theileria annulata strain Ankara
Theileria parva strain Muguga
Plasmodium falciparum
Plasmodium knowlesi
Plasmodium vivax
Plasmodium chabaudi
Plasmodium yoelii yoelii 17XNL
Plasmodium berghei
Phaeodactylum tricornutum
Thalassiosira pseudonana
Pythium ultimum
Albugo laibachii
Hyaloperonospora arabidopsidis
Phytophthora ramorum
Phytophthora infestans
Phytophthora sojae
Trypanosoma brucei
Trypanosoma cruzi strain CL Brener
Leishmania braziliensis MHOM BR 75 M2904
Leishmania mexicana MHOM GT 2001 U1103
Leishmania major
Leishmania donovani
Leishmania infantum JPCM5
Entamoeba histolytica
Entamoeba dispar SAW760
Dictyostelium discoideum
Dictyostelium purpureum
Trichomonas vaginalis G3
Trichomonas vaginalis
Naegleria gruberi
Giardia lamblia ATCC 50803
OG presence
OG absence
Inferred root
Dscore = 0.833
Pvalue = 1.32e−04
AdjPvalue = 1.57e−02

NOG13193

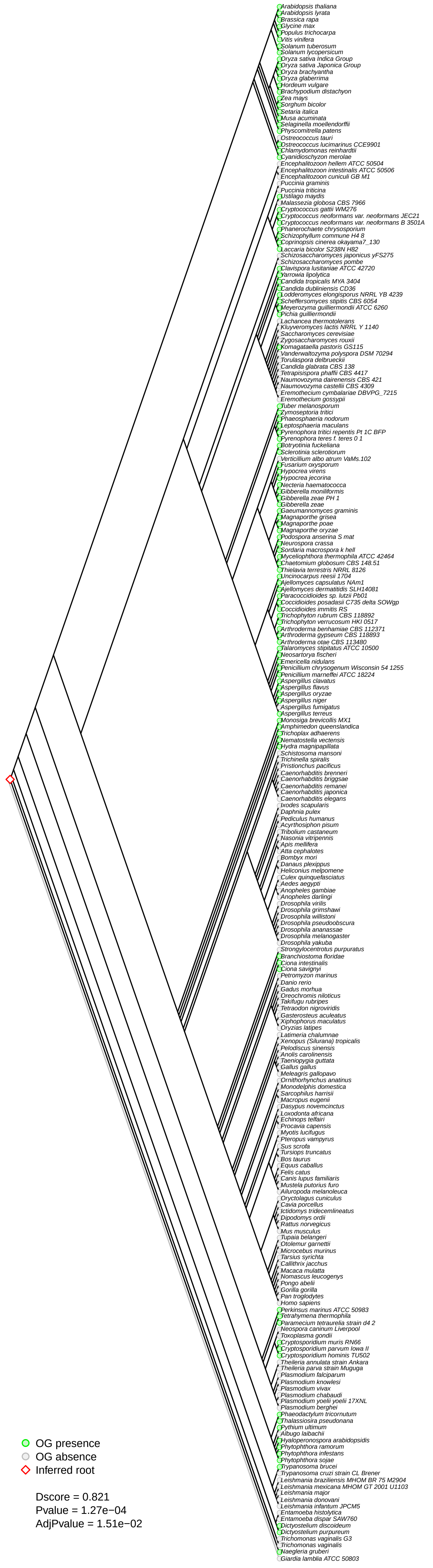
Arabidopsis thaliana
Arabidopsis lyrata
Brassica rapa
Glycine max
Populus trichocarpa
Vitis vinifera
Solanum tuberosum
Solanum lycopersicum
Oryza sativa Indica Group
Oryza sativa Japonica Group
Oryza brachyantha
Oryza glaberrima
Hordeum vulgare
Brachypodium distachyon
Zea mays
Sorghum bicolor
Setaria italica
Musa acuminata
Selaginella moellendorffii
Physcomitrella patens
Ostreococcus tauri
Ostreococcus lucimarinus CCE9901
Chlamydomonas reinhardtii
Cyanidioschyzon merolae
Encephalitozoon hellem ATCC 50504
Encephalitozoon intestinalis ATCC 50506
Encephalitozoon cuniculi GB M1
Puccinia graminis
Puccinia triticina
Ustilago maydis
Malassezia globosa CBS 7966
Cryptococcus gattii WM276
Cryptococcus neoformans var. neoformans JEC21
Cryptococcus neoformans var. neoformans B 3501A
Phanerochaete chrysosporium
Schizophyllum commune H4 8
Coprinopsis cinerea okayama7_130
Laccaria bicolor S238N H82
Schizosaccharomyces japonicus yFS275
Schizosaccharomyces pombe
Clavispora lusitaniae ATCC 42720
Yarrowia lipolytica
Candida tropicalis MYA 3404
Candida dubliniensis CD36
Lodderomyces elongisporus NRRL YB 4239
Scheffersomyces stipitis CBS 6054
Meyerozyma guilliermondii ATCC 6260
Pichia guilliermondii
Lachancea thermotolerans
Kluyveromyces lactis NRRL Y 1140
Saccharomyces cerevisiae
Zygosaccharomyces rouxii
Komagataella pastoris GS115
Vanderwaltozyma polyspora DSM 70294
Torulaspora delbrueckii
Candida glabrata CBS 138
Tetrapisispora phaffii CBS 4417
Naumovozyma dairenensis CBS 421
Naumovozyma castellii CBS 4309
Eremothecium cymbalariae DBVPG_7215
Eremothecium gossypii
Tuber melanosporum
Zymoseptoria tritici
Phaeosphaeria nodorum
Leptosphaeria maculans
Pyrenophora tritici repentis Pt 1C BFP
Pyrenophora teres f. teres 0 1
Botryotinia fuckeliana
Sclerotinia sclerotiorum
Verticillium albo atrum VaMs.102
Fusarium oxysporum
Hypocrea virens
Hypocrea jecorina
Necteria haematococca
Gibberella moniliformis
Gibberella zeae PH 1
Gibberella zeae
Gaeumannomyces graminis
Magnaporthe grisea
Magnaporthe poae
Magnaporthe oryzae
Podospora anserina S mat
Neurospora crassa
Sordaria macrospora k hell
Myceliophthora thermophila ATCC 42464
Chaetomium globosum CBS 148.51
Thielavia terrestris NRRL 8126
Uncinocarpus reesii 1704
Ajellomyces capsulatus NAm1
Ajellomyces dermatitidis SLH14081
Paracoccidioides sp. lutzii Pb01
Coccidioides posadasii C735 delta SOWgp
Coccidioides immitis RS
Trichophyton rubrum CBS 118892
Trichophyton verrucosum HKI 0517
Arthroderma benhamiae CBS 112371
Arthroderma gypseum CBS 118893
Arthroderma otae CBS 113480
Talaromyces stipitatus ATCC 10500
Neosartorya fischeri
Emericella nidulans
Penicillium chrysogenum Wisconsin 54 1255
Penicillium marneffei ATCC 18224
Aspergillus clavatus
Aspergillus flavus
Aspergillus oryzae
Aspergillus niger
Aspergillus fumigatus
Aspergillus terreus
Monosiga brevicollis MX1
Amphimedon queenslandica
Trichoplax adhaerens
Nematostella vectensis
Hydra magnipapillata
Schistosoma mansoni
Trichinella spiralis
Pristionchus pacificus
Caenorhabditis brenneri
Caenorhabditis briggsae
Caenorhabditis remanei
Caenorhabditis japonica
Caenorhabditis elegans
Ixodes scapularis
Daphnia pulex
Pediculus humanus
Acyrthosiphon pisum
Tribolium castaneum
Nasonia vitripennis
Apis mellifera
Atta cephalotes
Bombyx mori
Danaus plexippus
Heliconius melpomene
Culex quinquefasciatus
Aedes aegypti
Anopheles gambiae
Anopheles darlingi
Drosophila virilis
Drosophila grimshawi
Drosophila willistoni
Drosophila pseudoobscura
Drosophila ananassae
Drosophila melanogaster
Drosophila yakuba
Strongylocentrotus purpuratus
Branchiostoma floridae
Ciona intestinalis
Ciona savignyi
Petromyzon marinus
Danio rerio
Gadus morhua
Oreochromis niloticus
Takifugu rubripes
Tetraodon nigroviridis
Gasterosteus aculeatus
Xiphophorus maculatus
Oryzias latipes
Latimeria chalumnae
Xenopus (Silurana) tropicalis
Pelodiscus sinensis
Anolis carolinensis
Taeniopygia guttata
Gallus gallus
Meleagris gallopavo
Ornithorhynchus anatinus
Monodelphis domestica
Sarcophilus harrisii
Macropus eugenii
Dasypus novemcinctus
Loxodonta africana
Echinops telfairi
Procavia capensis
Myotis lucifugus
Pteropus vampyrus
Sus scrofa
Tursiops truncatus
Bos taurus
Equus caballus
Felis catus
Canis lupus familiaris
Mustela putorius furo
Ailuropoda melanoleuca
Oryctolagus cuniculus
Cavia porcellus
Ictidomys tridecemlineatus
Dipodomys ordii
Rattus norvegicus
Mus musculus
Tupaia belangeri
Otolemur garnettii
Microcebus murinus
Tarsius syrichta
Callithrix jacchus
Macaca mulatta
Nomascus leucogenys
Pongo abelii
Gorilla gorilla
Pan troglodytes
Homo sapiens
Perkinsus marinus ATCC 50983
Tetrahymena thermophila
Paramecium tetraurelia strain d4 2
Neospora caninum Liverpool
Toxoplasma gondii
Cryptosporidium muris RN66
Cryptosporidium parvum Iowa II
Cryptosporidium hominis TU502
Theileria annulata strain Ankara
Theileria parva strain Muguga
Plasmodium falciparum
Plasmodium knowlesi
Plasmodium vivax
Plasmodium chabaudi
Plasmodium yoelii yoelii 17XNL
Plasmodium berghei
Phaeodactylum tricornutum
Thalassiosira pseudonana
Pythium ultimum
Albugo laibachii
Hyaloperonospora arabidopsidis
Phytophthora ramorum
Phytophthora infestans
Phytophthora sojae
Trypanosoma brucei
Trypanosoma cruzi strain CL Brener
Leishmania braziliensis MHOM BR 75 M2904
Leishmania mexicana MHOM GT 2001 U1103
Leishmania major
Leishmania donovani
Leishmania infantum JPCM5
Entamoeba histolytica
Entamoeba dispar SAW760
Dictyostelium discoideum
Dictyostelium purpureum
Trichomonas vaginalis G3
Trichomonas vaginalis
Naegleria gruberi
Giardia lamblia ATCC 50803
OG presence
OG absence
Inferred root
Dscore = 0.821
Pvalue = 1.27e−04
AdjPvalue = 1.51e−02

NOG133099

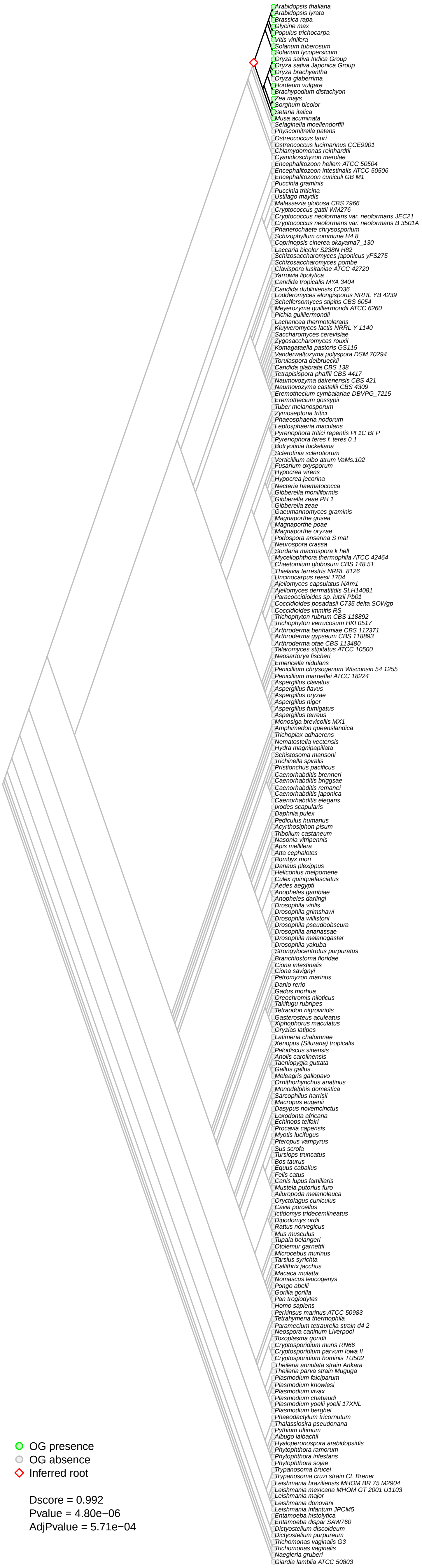

Arabidopsis thaliana
Arabidopsis lyrata
Brassica rapa
Glycine max
Populus trichocarpa
Vitis vinifera
Solanum tuberosum
Solanum lycopersicum
Oryza sativa Indica Group
Oryza sativa Japonica Group
Oryza brachyantha
Oryza glaberrima
Hordeum vulgare
Brachypodium distachyon
Zea mays
Sorghum bicolor
Setaria italica
Musa acuminata
Selaginella moellendorffii
Physcomitrella patens
Ostreococcus tauri
Ostreococcus lucimarinus CCE9901
Chlamydomonas reinhardtii
Cyanidioschyzon merolae
Encephalitozoon hellem ATCC 50504
Encephalitozoon intestinalis ATCC 50506
Encephalitozoon cuniculi GB M1
Puccinia graminis
Puccinia triticina
Ustilago maydis
Malassezia globosa CBS 7966
Cryptococcus gattii WM276
Cryptococcus neoformans var. neoformans JEC21
Cryptococcus neoformans var. neoformans B 3501A
Phanerochaete chrysosporium
Schizophyllum commune H4 8
Coprinopsis cinerea okayama7_130
Laccaria bicolor S238N H82
Schizosaccharomyces japonicus yFS275
Schizosaccharomyces pombe
Clavispora lusitaniae ATCC 42720
Yarrowia lipolytica
Candida tropicalis MYA 3404
Candida dubliniensis CD36
Lodderomyces elongisporus NRRL YB 4239
Scheffersomyces stipitis CBS 6054
Meyerozyma guilliermondii ATCC 6260
Pichia guilliermondii
Lachancea thermotolerans
Kluyveromyces lactis NRRL Y 1140
Saccharomyces cerevisiae
Zygosaccharomyces rouxii
Komagataella pastoris GS115
Vanderwaltozyma polyspora DSM 70294
Torulaspora delbrueckii
Candida glabrata CBS 138
Tetrapisispora phaffii CBS 4417
Naumovozyma dairenensis CBS 421
Naumovozyma castellii CBS 4309
Eremothecium cymbalariae DBVPG_7215
Eremothecium gossypii
Tuber melanosporum
Zymoseptoria tritici
Phaeosphaeria nodorum
Leptosphaeria maculans
Pyrenophora tritici repentis Pt 1C BFP
Pyrenophora teres f. teres 0 1
Botryotinia fuckeliana
Sclerotinia sclerotiorum
Verticillium albo atrum VaMs.102
Fusarium oxysporum
Hypocrea virens
Hypocrea jecorina
Nectria haematococca
Gibberella moniliformis
Gibberella zeae PH 1
Gibberella zeae
Gaeumannomyces graminis
Magnaporthe grisea
Magnaporthe poae
Magnaporthe oryzae
Podospora anserina S mat
Neurospora crassa
Sordaria macrospora k hell
Myceliophthora thermophila ATCC 42464
Chaetomium globosum CBS 148.51
Thielavia terrestris NRRL 8126
Uncinocarpus reesii 1704
Ajellomyces capsulatus NAm1
Ajellomyces dermatitidis SLH14081
Paracoccidioides sp. lutzii Pb01
Coccidioides posadasii C735 delta SOWgp
Coccidioides immitis RS
Trichophyton rubrum CBS 118892
Trichophyton verrucosum HKI 0517
Arthroderma benhamiae CBS 112371
Arthroderma gypseum CBS 118893
Arthroderma otae CBS 113480
Talaromyces stipitatus ATCC 10500
Neosartorya fischeri
Emericella nidulans
Penicillium chrysogenum Wisconsin 54 1255
Penicillium marneffei ATCC 18224
Aspergillus clavatus
Aspergillus flavus
Aspergillus oryzae
Aspergillus niger
Aspergillus fumigatus
Aspergillus terreus
Monosiga brevicollis MX1
Amphimedon queenslandica
Trichoplax adhaerens
Nematostella vectensis
Hydra magnipapillata
Schistosoma mansoni
Trichinella spiralis
Pristionchus pacificus
Caenorhabditis brenneri
Caenorhabditis briggsae
Caenorhabditis remanei
Caenorhabditis japonica
Caenorhabditis elegans
Ixodes scapularis
Daphnia pulex
Pediculus humanus
Acyrthosiphon pisum
Tribolium castaneum
Nasonia vitripennis
Apis mellifera
Atta cephalotes
Bombyx mori
Danaus plexippus
Heliconius melpomene
Culex quinquefasciatus
Aedes aegypti
Anopheles gambiae
Anopheles darlingi
Drosophila virilis
Drosophila grimshawi
Drosophila willistoni
Drosophila pseudoobscura
Drosophila ananassae
Drosophila melanogaster
Drosophila yakuba
Strongylocentrotus purpuratus
Branchiostoma floridae
Ciona intestinalis
Ciona savignyi
Petromyzon marinus
Danio rerio
Gadus morhua
Oreochromis niloticus
Takifugu rubripes
Tetraodon nigroviridis
Gasterosteus aculeatus
Xiphophorus maculatus
Oryzias latipes
Latimeria chalumnae
Xenopus (Silurana) tropicalis
Pelodiscus sinensis
Anolis carolinensis
Taeniopygia guttata
Gallus gallus
Meleagris gallopavo
Ornithorhynchus anatinus
Monodelphis domestica
Sarcophilus harrisii
Macropus eugenii
Dasypus novemcinctus
Loxodonta africana
Echinops telfairi
Procavia capensis
Myotis lucifugus
Pteropus vampyrus
Sus scrofa
Tursiops truncatus
Bos taurus
Equus caballus
Felis catus
Canis lupus familiaris
Mustela putorius furo
Ailuropoda melanoleuca
Oryctolagus cuniculus
Cavia porcellus
Ictidomys tridecemlineatus
Dipodomys ordii
Rattus norvegicus
Mus musculus
Tupaia belangeri
Otolemur garnettii
Microcebus murinus
Tarsius syrichta
Callithrix jacchus
Macaca mulatta
Nomascus leucogenys
Pongo abelii
Gorilla gorilla
Pan troglodytes
Homo sapiens
Perkinsus marinus ATCC 50983
Tetrahymena thermophila
Paramecium tetraurelia strain d4 2
Neospora caninum Liverpool
Toxoplasma gondii
Cryptosporidium muris RN66
Cryptosporidium parvum Iowa II
Cryptosporidium hominis TU502
Theileria annulata strain Ankara
Theileria parva strain Muguga
Plasmodium falciparum
Plasmodium knowlesi
Plasmodium vivax
Plasmodium chabaudi
Plasmodium yoelii yoelii 17XNL
Plasmodium berghei
Phaeodactylum tricornutum
Thalassiosira pseudonana
Pythium ultimum
Albugo laibachii
Hyaloperonospora arabidopsidis
Phytophthora ramorum
Phytophthora infestans
Phytophthora sojae
Trypanosoma brucei
Trypanosoma cruzi strain CL Brener
Leishmania braziliensis MHOM BR 75 M2904
Leishmania mexicana MHOM GT 2001 U1103
Leishmania major
Leishmania donovani
Leishmania infantum JPCM5
Entamoeba histolytica
Entamoeba dispar SAW760
Dictyostelium discoideum
Dictyostelium purpureum
Trichomonas vaginalis G3
Trichomonas vaginalis
Naegleria gruberi
Giardia lamblia ATCC 50803
OG presence
OG absence
Inferred root
Dscore = 0.992
Pvalue = 4.80e−06
AdjPvalue = 5.71e−04

NOG135096

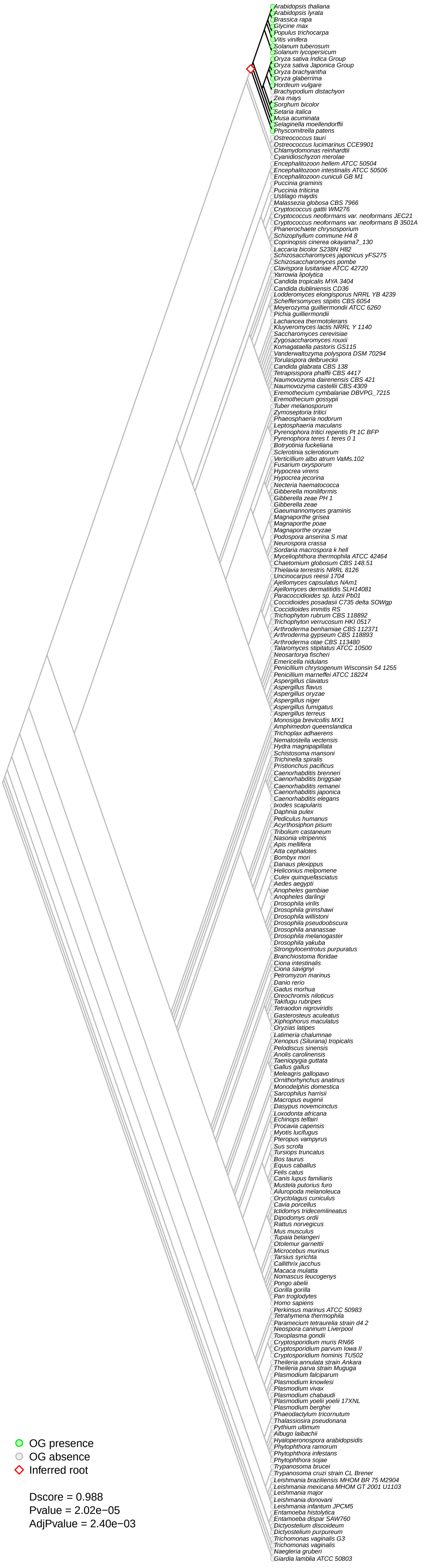
Arabidopsis thaliana
Arabidopsis lyrata
Brassica rapa
Glycine max
Populus trichocarpa
Vitis vinifera
Solanum tuberosum
Solanum lycopersicum
Oryza sativa Indica Group
Oryza sativa Japonica Group
Oryza brachyantha
Oryza glaberrima
Hordeum vulgare
Brachypodium distachyon
Zea mays
Sorghum bicolor
Setaria italica
Musa acuminata
Selaginella moellendorffii
Physcomitrella patens
Ostreococcus tauri
Ostreococcus lucimarinus CCE9901
Chlamydomonas reinhardtii
Cyanidioschyzon merolae
Encephalitozoon hellem ATCC 50504
Encephalitozoon intestinalis ATCC 50506
Encephalitozoon cuniculi GB M1
Puccinia graminis
Puccinia triticina
Ustilago maydis
Malassezia globosa CBS 7966
Cryptococcus gattii WM276
Cryptococcus neoformans var. neoformans JEC21
Cryptococcus neoformans var. neoformans B 3501A
Phanerochaete chrysosporium
Schizophyllum commune H4 8
Coprinopsis cinerea okayama7_130
Laccaria bicolor S238N H82
Schizosaccharomyces japonicus yFS275
Schizosaccharomyces pombe
Clavispora lusitaniae ATCC 42720
Yarrowia lipolytica
Candida tropicalis MYA 3404
Candida dubliniensis CD36
Lodderomyces elongisporus NRRL YB 4239
Scheffersomyces stipitis CBS 6054
Meyerozyma guilliermondii ATCC 6260
Pichia guilliermondii
Lachancea thermotolerans
Kluyveromyces lactis NRRL Y 1140
Saccharomyces cerevisiae
Zygosaccharomyces rouxii
Komagataella pastoris GS115
Vanderwaltozyma polyspora DSM 70294
Torulaspora delbrueckii
Candida glabrata CBS 138
Tetrapisispora phaffii CBS 4417
Naumovozyma dairenensis CBS 421
Naumovozyma castellii CBS 4309
Eremothecium cymbalariae DBVPG_7215
Eremothecium gossypii
Tuber melanosporum
Zymoseptoria tritici
Phaeosphaeria nodorum
Leptosphaeria maculans
Pyrenophora tritici repentis Pt 1C BFP
Pyrenophora teres f. teres 0 1
Botryotinia fuckeliana
Sclerotinia sclerotiorum
Verticillium albo atrum VaMs.102
Fusarium oxysporum
Hypocrea virens
Hypocrea jecorina
Necteria haematococca
Gibberella moniliformis
Gibberella zeae PH 1
Gibberella zeae
Gaeumannomyces graminis
Magnaporthe grisea
Magnaporthe poae
Magnaporthe oryzae
Podospora anserina S mat
Neurospora crassa
Sordaria macrospora k hell
Myceliophthora thermophila ATCC 42464
Chaetomium globosum CBS 148.51
Thielavia terrestris NRRL 8126
Uncinocarpus reesii 1704
Ajellomyces capsulatus NAm1
Ajellomyces dermatitidis SLH14081
Paracoccidioides sp. lutzii Pb01
Coccidioides posadasii C735 delta SOWgp
Coccidioides immitis RS
Trichophyton rubrum CBS 118892
Trichophyton verrucosum HKI 0517
Arthroderma benhamiae CBS 112371
Arthroderma gypseum CBS 118893
Arthroderma otae CBS 113480
Talaromyces stipitatus ATCC 10500
Neosartorya fischeri
Emericella nidulans
Penicillium chrysogenum Wisconsin 54 1255
Penicillium marneffei ATCC 18224
Aspergillus clavatus
Aspergillus flavus
Aspergillus oryzae
Aspergillus niger
Aspergillus fumigatus
Aspergillus terreus
Monosiga brevicollis MX1
Amphimedon queenslandica
Trichoplax adhaerens
Nematostella vectensis
Hydra magnipapillata
Schistosoma mansoni
Trichinella spiralis
Pristionchus pacificus
Caenorhabditis brenneri
Caenorhabditis briggsae
Caenorhabditis remanei
Caenorhabditis japonica
Caenorhabditis elegans
Ixodes scapularis
Daphnia pulex
Pediculus humanus
Acyrthosiphon pisum
Tribolium castaneum
Nasonia vitripennis
Apis mellifera
Atta cephalotes
Bombyx mori
Danaus plexippus
Heliconius melpomene
Culex quinquefasciatus
Aedes aegypti
Anopheles gambiae
Anopheles darlingi
Drosophila virilis
Drosophila grimshawi
Drosophila willistoni
Drosophila pseudoobscura
Drosophila ananassae
Drosophila melanogaster
Drosophila yakuba
Strongylocentrotus purpuratus
Branchiostoma floridae
Ciona intestinalis
Ciona savignyi
Petromyzon marinus
Danio rerio
Gadus morhua
Oreochromis niloticus
Takifugu rubripes
Tetraodon nigroviridis
Gasterosteus aculeatus
Xiphophorus maculatus
Oryzias latipes
Latimeria chalumnae
Xenopus (Silurana) tropicalis
Pelodiscus sinensis
Anolis carolinensis
Taeniopygia guttata
Gallus gallus
Meleagris gallopavo
Ornithorhynchus anatinus
Monodelphis domestica
Sarcophilus harrisii
Macropus eugenii
Dasypus novemcinctus
Loxodonta africana
Echinops telfairi
Procavia capensis
Myotis lucifugus
Pteropus vampyrus
Sus scrofa
Tursiops truncatus
Bos taurus
Equus caballus
Felis catus
Canis lupus familiaris
Mustela putorius furo
Ailuropoda melanoleuca
Oryctolagus cuniculus
Cavia porcellus
Ictidomys tridecemlineatus
Dipodomys ordii
Rattus norvegicus
Mus musculus
Tupaia belangeri
Otolemur garnettii
Microcebus murinus
Tarsius syrichta
Callithrix jacchus
Macaca mulatta
Nomascus leucogenys
Pongo abelii
Gorilla gorilla
Pan troglodytes
Homo sapiens
Perkinsus marinus ATCC 50983
Tetrahymena thermophila
Paramecium tetraurelia strain d4 2
Neospora caninum Liverpool
Toxoplasma gondii
Cryptosporidium muris RN66
Cryptosporidium parvum Iowa II
Cryptosporidium hominis TU502
Theileria annulata strain Ankara
Theileria parva strain Muguga
Plasmodium falciparum
Plasmodium knowlesi
Plasmodium vivax
Plasmodium chabaudi
Plasmodium yoelii yoelii 17XNL
Plasmodium berghei
Phaeodactylum tricornutum
Thalassiosira pseudonana
Pythium ultimum
Albugo laibachii
Hyaloperonospora arabidopsidis
Phytophthora ramorum
Phytophthora infestans
Phytophthora sojae
Trypanosoma brucei
Trypanosoma cruzi strain CL Brener
Leishmania braziliensis MHOM BR 75 M2904
Leishmania mexicana MHOM GT 2001 U1103
Leishmania major
Leishmania donovani
Leishmania infantum JPCM5
Entamoeba histolytica
Entamoeba dispar SAW760
Dictyostelium discoideum
Dictyostelium purpureum
Trichomonas vaginalis G3
Trichomonas vaginalis
Naegleria gruberi
Giardia lamblia ATCC 50803
OG presence
OG absence
Inferred root
Dscore = 0.988
Pvalue = 2.02e−05
AdjPvalue = 2.40e−03

NOG136237

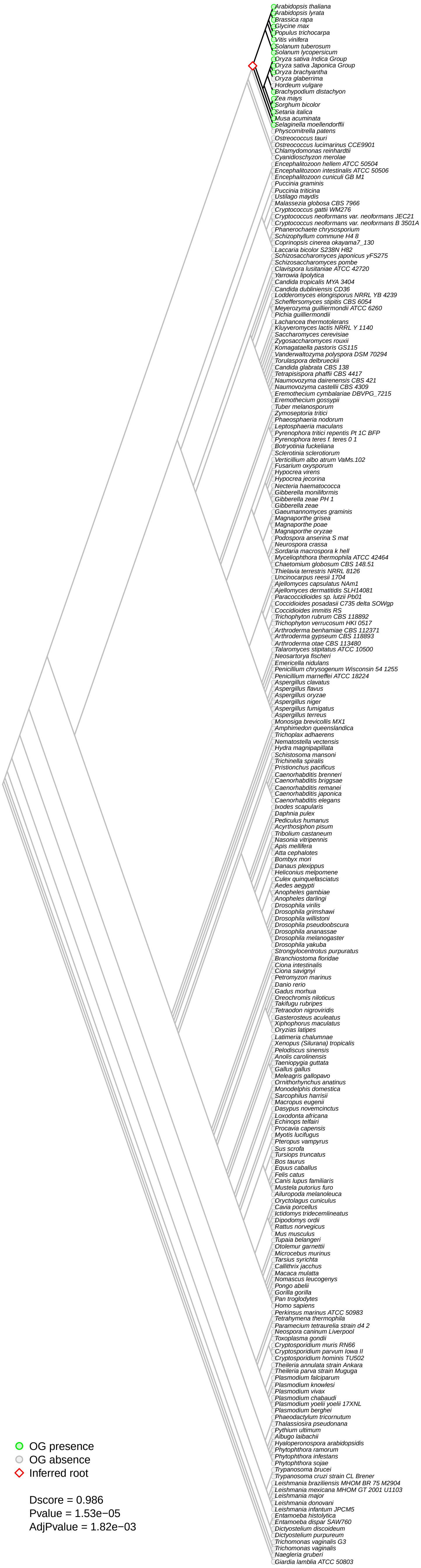
Arabidopsis thaliana
Arabidopsis lyrata
Brassica rapa
Glycine max
Populus trichocarpa
Vitis vinifera
Solanum tuberosum
Solanum lycopersicum
Oryza sativa Indica Group
Oryza sativa Japonica Group
Oryza brachyantha
Oryza glaberrima
Hordeum vulgare
Brachypodium distachyon
Zea mays
Sorghum bicolor
Setaria italica
Musa acuminata
Selaginella moellendorffii
Physcomitrella patens
Ostreococcus tauri
Ostreococcus lucimarinus CCE9901
Chlamydomonas reinhardtii
Cyanidioschyzon merolae
Encephalitozoon hellem ATCC 50504
Encephalitozoon intestinalis ATCC 50506
Encephalitozoon cuniculi GB M1
Puccinia graminis
Puccinia triticina
Ustilago maydis
Malassezia globosa CBS 7966
Cryptococcus gattii WM276
Cryptococcus neoformans var. neoformans JEC21
Cryptococcus neoformans var. neoformans B 3501A
Phanerochaete chrysosporium
Schizophyllum commune H4 8
Coprinopsis cinerea okayama7_130
Laccaria bicolor S238N H82
Schizosaccharomyces japonicus yFS275
Schizosaccharomyces pombe
Clavispora lusitaniae ATCC 42720
Yarrowia lipolytica
Candida tropicalis MYA 3404
Candida dubliniensis CD36
Lodderomyces elongisporus NRRL YB 4239
Scheffersomyces stipitis CBS 6054
Meyerozyma guilliermondii ATCC 6260
Pichia guilliermondii
Lachancea thermotolerans
Kluyveromyces lactis NRRL Y 1140
Saccharomyces cerevisiae
Zygosaccharomyces rouxii
Komagataella pastoris GS115
Vanderwaltozyma polyspora DSM 70294
Torulaspora delbrueckii
Candida glabrata CBS 138
Tetrapisispora phaffii CBS 4417
Naumovozyma dairenensis CBS 421
Naumovozyma castellii CBS 4309
Eremothecium cymbalariae DBVPG_7215
Eremothecium gossypii
Tuber melanosporum
Zymoseptoria tritici
Phaeosphaeria nodorum
Leptosphaeria maculans
Pyrenophora tritici repentis Pt 1C BFP
Pyrenophora teres f. teres 0 1
Botryotinia fuckeliana
Sclerotinia sclerotiorum
Verticillium albo atrum VaMs.102
Fusarium oxysporum
Hypocrea virens
Hypocrea jecorina
Nectria haematococca
Gibberella moniliformis
Gibberella zeae PH 1
Gibberella zeae
Gaeumannomyces graminis
Magnaporthe grisea
Magnaporthe poae
Magnaporthe oryzae
Podospora anserina S mat
Neurospora crassa
Sordaria macrospora k hell
Myceliophthora thermophila ATCC 42464
Chaetomium globosum CBS 148.51
Thielavia terrestris NRRL 8126
Uncinocarpus reesii 1704
Ajellomyces capsulatus NAm1
Ajellomyces dermatitidis SLH14081
Paracoccidioides sp. lutzii Pb01
Coccidioides posadasii C735 delta SOWgp
Coccidioides immitis RS
Trichophyton rubrum CBS 118892
Trichophyton verrucosum HKI 0517
Arthroderma benhamiae CBS 112371
Arthroderma gypseum CBS 118893
Arthroderma otae CBS 113480
Talaromyces stipitatus ATCC 10500
Neosartorya fischeri
Emericella nidulans
Penicillium chrysogenum Wisconsin 54 1255
Penicillium marneffei ATCC 18224
Aspergillus clavatus
Aspergillus flavus
Aspergillus oryzae
Aspergillus niger
Aspergillus fumigatus
Aspergillus terreus
Monosiga brevicollis MX1
Amphimedon queenslandica
Trichoplax adhaerens
Nematostella vectensis
Hydra magnipapillata
Schistosoma mansoni
Trichinella spiralis
Pristionchus pacificus
Caenorhabditis brenneri
Caenorhabditis briggsae
Caenorhabditis remanei
Caenorhabditis japonica
Caenorhabditis elegans
Ixodes scapularis
Daphnia pulex
Pediculus humanus
Acyrthosiphon pisum
Tribolium castaneum
Nasonia vitripennis
Apis mellifera
Atta cephalotes
Bombyx mori
Danaus plexippus
Heliconius melpomene
Culex quinquefasciatus
Aedes aegypti
Anopheles gambiae
Anopheles darlingi
Drosophila virilis
Drosophila grimshawi
Drosophila willistoni
Drosophila pseudoobscura
Drosophila ananassae
Drosophila melanogaster
Drosophila yakuba
Strongylocentrotus purpuratus
Branchiostoma floridae
Ciona intestinalis
Ciona savignyi
Petromyzon marinus
Danio rerio
Gadus morhua
Oreochromis niloticus
Takifugu rubripes
Tetraodon nigroviridis
Gasterosteus aculeatus
Xiphophorus maculatus
Oryzias latipes
Latimeria chalumnae
Xenopus (Silurana) tropicalis
Pelodiscus sinensis
Anolis carolinensis
Taeniopygia guttata
Gallus gallus
Meleagris gallopavo
Ornithorhynchus anatinus
Monodelphis domestica
Sarcophilus harrisii
Macropus eugenii
Dasypus novemcinctus
Loxodonta africana
Echinops telfairi
Procavia capensis
Myotis lucifugus
Pteropus vampyrus
Sus scrofa
Tursiops truncatus
Bos taurus
Equus caballus
Felis catus
Canis lupus familiaris
Mustela putorius furo
Ailuropoda melanoleuca
Oryctolagus cuniculus
Cavia porcellus
Ictidomys tridecemlineatus
Dipodomys ordii
Rattus norvegicus
Mus musculus
Tupaia belangeri
Otolemur garnettii
Microcebus murinus
Tarsius syrichta
Callithrix jacchus
Macaca mulatta
Nomascus leucogenys
Pongo abelii
Gorilla gorilla
Pan troglodytes
Homo sapiens
Perkinsus marinus ATCC 50983
Tetrahymena thermophila
Paramecium tetraurelia strain d4 2
Neospora caninum Liverpool
Toxoplasma gondii
Cryptosporidium muris RN66
Cryptosporidium parvum Iowa II
Cryptosporidium hominis TU502
Theileria annulata strain Ankara
Theileria parva strain Muguga
Plasmodium falciparum
Plasmodium knowlesi
Plasmodium vivax
Plasmodium chabaudi
Plasmodium yoelii yoelii 17XNL
Plasmodium berghei
Phaeodactylum tricornutum
Thalassiosira pseudonana
Pythium ultimum
Albugo laibachii
Hyaloperonospora arabidopsidis
Phytophthora ramorum
Phytophthora infestans
Phytophthora sojae
Trypanosoma brucei
Trypanosoma cruzi strain CL Brener
Leishmania braziliensis MHOM BR 75 M2904
Leishmania mexicana MHOM GT 2001 U1103
Leishmania major
Leishmania donovani
Leishmania infantum JPCM5
Entamoeba histolytica
Entamoeba dispar SAW760
Dictyostelium discoideum
Dictyostelium purpureum
Trichomonas vaginalis G3
Trichomonas vaginalis
Naegleria gruberi
Giardia lamblia ATCC 50803
OG presence
OG absence
Inferred root
Dscore = 0.986
Pvalue = 1.53e−05
AdjPvalue = 1.82e−03

NOG140340

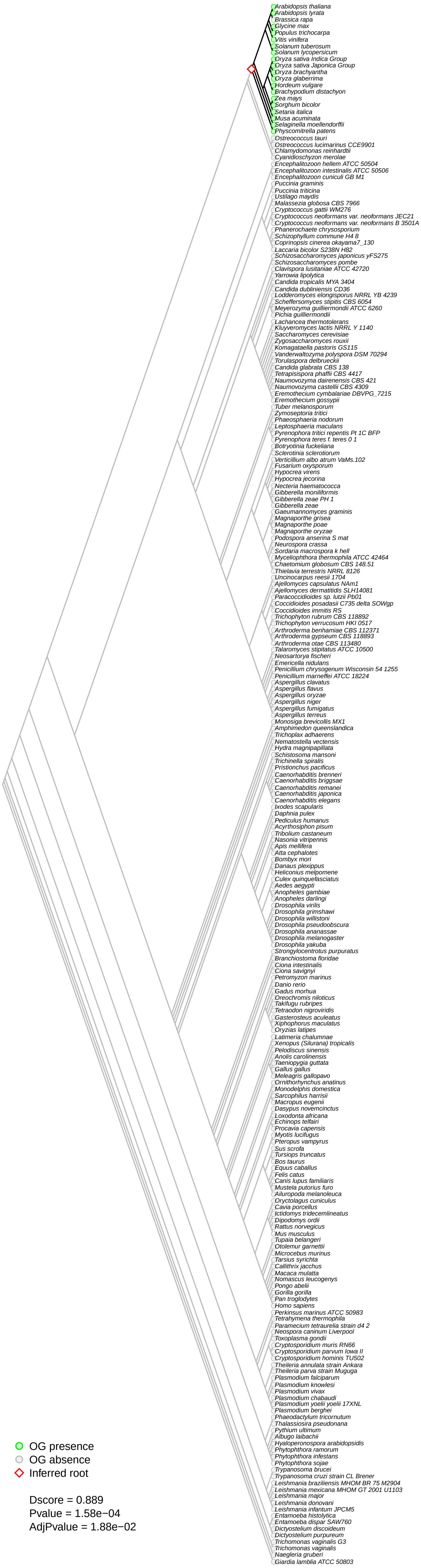

Arabidopsis thaliana
Arabidopsis lyrata
Brassica rapa
Glycine max
Populus trichocarpa
Vitis vinifera
Solanum tuberosum
Solanum lycopersicum
Oryza sativa Indica Group
Oryza sativa Japonica Group
Oryza brachyantha
Oryza glaberrima
Hordeum vulgare
Brachypodium distachyon
Zea mays
Sorghum bicolor
Setaria italica
Musa acuminata
Selaginella moellendorffii
Physcomitrella patens
Ostreococcus tauri
Ostreococcus lucimarinus CCE9901
Chlamydomonas reinhardtii
Cyanidioschyzon merolae
Encephalitozoon hellem ATCC 50504
Encephalitozoon intestinalis ATCC 50506
Encephalitozoon cuniculi GB M1
Puccinia graminis
Puccinia triticina
Ustilago maydis
Malassezia globosa CBS 7966
Cryptococcus gattii WM276
Cryptococcus neoformans var. neoformans JEC21
Cryptococcus neoformans var. neoformans B 3501A
Phanerochaete chrysosporium
Schizophyllum commune H4 8
Coprinopsis cinerea okayama7_130
Laccaria bicolor S238N H82
Schizosaccharomyces japonicus yFS275
Schizosaccharomyces pombe
Clavispora lusitaniae ATCC 42720
Yarrowia lipolytica
Candida tropicalis MYA 3404
Candida dubliniensis CD36
Lodderomyces elongisporus NRRL YB 4239
Scheffersomyces stipitis CBS 6054
Meyerozyma guilliermondii ATCC 6260
Pichia guilliermondii
Lachancea thermotolerans
Kluyveromyces lactis NRRL Y 1140
Saccharomyces cerevisiae
Zygosaccharomyces rouxii
Komagataella pastoris GS115
Vanderwaltozyma polyspora DSM 70294
Torulaspora delbrueckii
Candida glabrata CBS 138
Tetrapisispora phaffii CBS 4417
Naumovozyma dairenensis CBS 421
Naumovozyma castellii CBS 4309
Eremothecium cymbalariae DBVPG_7215
Eremothecium gossypii
Tuber melanosporum
Zymoseptoria tritici
Phaeosphaeria nodorum
Leptosphaeria maculans
Pyrenophora tritici repentis Pt 1C BFP
Pyrenophora teres f. teres 0 1
Botryotinia fuckeliana
Sclerotinia sclerotiorum
Verticillium albo atrum VaMs.102
Fusarium oxysporum
Hypocrea virens
Hypocrea jecorina
Nectria haematococca
Gibberella moniliformis
Gibberella zeae PH 1
Gibberella zeae
Gaeumannomyces graminis
Magnaporthe grisea
Magnaporthe poae
Magnaporthe oryzae
Podospora anserina S mat
Neurospora crassa
Sordaria macrospora k hell
Myceliophthora thermophila ATCC 42464
Chaetomium globosum CBS 148.51
Thielavia terrestris NRRL 8126
Uncinocarpus reesii 1704
Ajellomyces capsulatus NAm1
Ajellomyces dermatitidis SLH14081
Paracoccidioides sp. lutzii Pb01
Coccidioides posadasii C735 delta SOWgp
Coccidioides immitis RS
Trichophyton rubrum CBS 118892
Trichophyton verrucosum HKI 0517
Arthroderma benhamiae CBS 112371
Arthroderma gypseum CBS 118893
Arthroderma otae CBS 113480
Talaromyces stipitatus ATCC 10500
Neosartorya fischeri
Emericella nidulans
Penicillium chrysogenum Wisconsin 54 1255
Penicillium marneffei ATCC 18224
Aspergillus clavatus
Aspergillus flavus
Aspergillus oryzae
Aspergillus niger
Aspergillus fumigatus
Aspergillus terreus
Monosiga brevicollis MX1
Amphimedon queenslandica
Trichoplax adhaerens
Nematostella vectensis
Hydra magnipapillata
Schistosoma mansoni
Trichinella spiralis
Pristionchus pacificus
Caenorhabditis brenneri
Caenorhabditis briggsae
Caenorhabditis remanei
Caenorhabditis japonica
Caenorhabditis elegans
Ixodes scapularis
Daphnia pulex
Pediculus humanus
Acyrthosiphon pisum
Tribolium castaneum
Nasonia vitripennis
Apis mellifera
Atta cephalotes
Bombyx mori
Danaus plexippus
Heliconius melpomene
Culex quinquefasciatus
Aedes aegypti
Anopheles gambiae
Anopheles darlingi
Drosophila virilis
Drosophila grimshawi
Drosophila willistoni
Drosophila pseudoobscura
Drosophila ananassae
Drosophila melanogaster
Drosophila yakuba
Strongylocentrotus purpuratus
Branchiostoma floridae
Ciona intestinalis
Ciona savignyi
Petromyzon marinus
Danio rerio
Gadus morhua
Oreochromis niloticus
Takifugu rubripes
Tetraodon nigroviridis
Gasterosteus aculeatus
Xiphophorus maculatus
Oryzias latipes
Latimeria chalumnae
Xenopus (Silurana) tropicalis
Pelodiscus sinensis
Anolis carolinensis
Taeniopygia guttata
Gallus gallus
Meleagris gallopavo
Ornithorhynchus anatinus
Monodelphis domestica
Sarcophilus harrisii
Macropus eugenii
Dasypus novemcinctus
Loxodonta africana
Echinops telfairi
Procavia capensis
Myotis lucifugus
Pteropus vampyrus
Sus scrofa
Tursiops truncatus
Bos taurus
Equus caballus
Felis catus
Canis lupus familiaris
Mustela putorius furo
Ailuropoda melanoleuca
Oryctolagus cuniculus
Cavia porcellus
Ictidomys tridecemlineatus
Dipodomys ordii
Rattus norvegicus
Mus musculus
Tupaia belangeri
Otolemur garnettii
Microcebus murinus
Tarsius syrichta
Callithrix jacchus
Macaca mulatta
Nomascus leucogenys
Pongo abelii
Gorilla gorilla
Pan troglodytes
Homo sapiens
Perkinsus marinus ATCC 50983
Tetrahymena thermophila
Paramecium tetraurelia strain d4 2
Neospora caninum Liverpool
Toxoplasma gondii
Cryptosporidium muris RN66
Cryptosporidium parvum Iowa II
Cryptosporidium hominis TU502
Theileria annulata strain Ankara
Theileria parva strain Muguga
Plasmodium falciparum
Plasmodium knowlesi
Plasmodium vivax
Plasmodium chabaudi
Plasmodium yoelii yoelii 17XNL
Plasmodium berghei
Phaeodactylum tricornutum
Thalassiosira pseudonana
Pythium ultimum
Albugo laibachii
Hyaloperonospora arabidopsidis
Phytophthora ramorum
Phytophthora infestans
Phytophthora sojae
Trypanosoma brucei
Trypanosoma cruzi strain CL Brener
Leishmania braziliensis MHOM BR 75 M2904
Leishmania mexicana MHOM GT 2001 U1103
Leishmania major
Leishmania donovani
Leishmania infantum JPCM5
Entamoeba histolytica
Entamoeba dispar SAW760
Dictyostelium discoideum
Dictyostelium purpureum
Trichomonas vaginalis G3
Trichomonas vaginalis
Naegleria gruberi
Giardia lamblia ATCC 50803
OG presence
OG absence
Inferred root
Dscore = 0.889
Pvalue = 1.58e−04
AdjPvalue = 1.88e−02

NOG141489

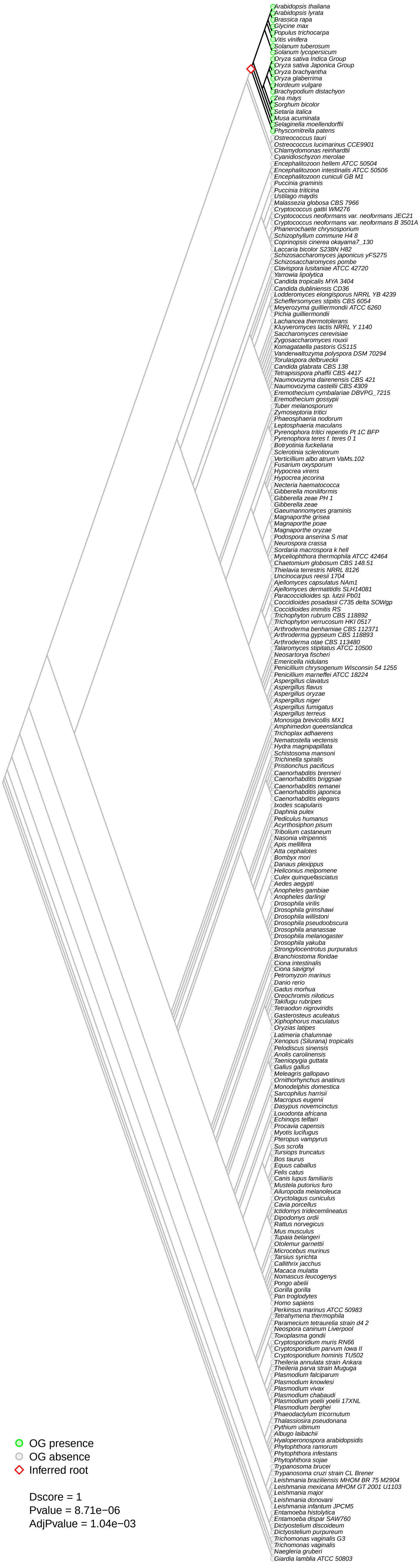

Arabidopsis thaliana
Arabidopsis lyrata
Brassica rapa
Glycine max
Populus trichocarpa
Vitis vinifera
Solanum tuberosum
Solanum lycopersicum
Oryza sativa Indica Group
Oryza sativa Japonica Group
Oryza brachyantha
Oryza glaberrima
Hordeum vulgare
Brachypodium distachyon
Zea mays
Sorghum bicolor
Setaria italica
Musa acuminata
Selaginella moellendorffii
Physcomitrella patens
Ostreococcus tauri
Ostreococcus lucimarinus CCE9901
Chlamydomonas reinhardtii
Cyanidioschyzon merolae
Encephalitozoon hellem ATCC 50504
Encephalitozoon intestinalis ATCC 50506
Encephalitozoon cuniculi GB M1
Puccinia graminis
Puccinia triticina
Ustilago maydis
Malassezia globosa CBS 7966
Cryptococcus gattii WM276
Cryptococcus neoformans var. neoformans JEC21
Cryptococcus neoformans var. neoformans B 3501A
Phanerochaete chrysosporium
Schizophyllum commune H4 8
Coprinopsis cinerea okayama7_130
Laccaria bicolor S238N H82
Schizosaccharomyces japonicus yFS275
Schizosaccharomyces pombe
Clavispora lusitaniae ATCC 42720
Yarrowia lipolytica
Candida tropicalis MYA 3404
Candida dubliniensis CD36
Lodderomyces elongisporus NRRL YB 4239
Scheffersomyces stipitis CBS 6054
Meyerozyma guilliermondii ATCC 6260
Pichia guilliermondii
Lachancea thermotolerans
Kluyveromyces lactis NRRL Y 1140
Saccharomyces cerevisiae
Zygosaccharomyces rouxii
Komagataella pastoris GS115
Vanderwaltozyma polyspora DSM 70294
Torulaspora delbrueckii
Candida glabrata CBS 138
Tetrapisispora phaffii CBS 4417
Naumovozyma dairenensis CBS 421
Naumovozyma castellii CBS 4309
Eremothecium cymbalariae DBVPG_7215
Eremothecium gossypii
Tuber melanosporum
Zymoseptoria tritici
Phaeosphaeria nodorum
Leptosphaeria maculans
Pyrenophora tritici repentis Pt 1C BFP
Pyrenophora teres f. teres 0 1
Botryotinia fuckeliana
Sclerotinia sclerotiorum
Verticillium albo atrum VaMs.102
Fusarium oxysporum
Hypocrea virens
Hypocrea jecorina
Nectria haematococca
Gibberella moniliformis
Gibberella zeae PH 1
Gibberella zeae
Gaeumannomyces graminis
Magnaporthe grisea
Magnaporthe poae
Magnaporthe oryzae
Podospora anserina S mat
Neurospora crassa
Sordaria macrospora k hell
Myceliophthora thermophila ATCC 42464
Chaetomium globosum CBS 148.51
Thielavia terrestris NRRL 8126
Uncinocarpus reesii 1704
Ajellomyces capsulatus NAm1
Ajellomyces dermatitidis SLH14081
Paracoccidioides sp. lutzii Pb01
Coccidioides posadasii C735 delta SOWgp
Coccidioides immitis RS
Trichophyton rubrum CBS 118892
Trichophyton verrucosum HKI 0517
Arthroderma benhamiae CBS 112371
Arthroderma gypseum CBS 118893
Arthroderma otae CBS 113480
Talaromyces stipitatus ATCC 10500
Neosartorya fischeri
Emericella nidulans
Penicillium chrysogenum Wisconsin 54 1255
Penicillium marneffei ATCC 18224
Aspergillus clavatus
Aspergillus flavus
Aspergillus oryzae
Aspergillus niger
Aspergillus fumigatus
Aspergillus terreus
Monosiga brevicollis MX1
Amphimedon queenslandica
Trichoplax adhaerens
Nematostella vectensis
Hydra magnipapillata
Schistosoma mansoni
Trichinella spiralis
Pristionchus pacificus
Caenorhabditis brenneri
Caenorhabditis briggsae
Caenorhabditis remanei
Caenorhabditis japonica
Caenorhabditis elegans
Ixodes scapularis
Daphnia pulex
Pediculus humanus
Acyrthosiphon pisum
Tribolium castaneum
Nasonia vitripennis
Apis mellifera
Atta cephalotes
Bombyx mori
Danaus plexippus
Heliconius melpomene
Culex quinquefasciatus
Aedes aegypti
Anopheles gambiae
Anopheles darlingi
Drosophila virilis
Drosophila grimshawi
Drosophila willistoni
Drosophila pseudoobscura
Drosophila ananassae
Drosophila melanogaster
Drosophila yakuba
Strongylocentrotus purpuratus
Branchiostoma floridae
Ciona intestinalis
Ciona savignyi
Petromyzon marinus
Danio rerio
Gadus morhua
Oreochromis niloticus
Takifugu rubripes
Tetraodon nigroviridis
Gasterosteus aculeatus
Xiphophorus maculatus
Oryzias latipes
Latimeria chalumnae
Xenopus (Silurana) tropicalis
Pelodiscus sinensis
Anolis carolinensis
Taeniopygia guttata
Gallus gallus
Meleagris gallopavo
Ornithorhynchus anatinus
Monodelphis domestica
Sarcophilus harrisii
Macropus eugenii
Dasypus novemcinctus
Loxodonta africana
Echinops telfairi
Procavia capensis
Myotis lucifugus
Pteropus vampyrus
Sus scrofa
Tursiops truncatus
Bos taurus
Equus caballus
Felis catus
Canis lupus familiaris
Mustela putorius furo
Ailuropoda melanoleuca
Oryctolagus cuniculus
Cavia porcellus
Ictidomys tridecemlineatus
Dipodomys ordii
Rattus norvegicus
Mus musculus
Tupaia belangeri
Otolemur garnettii
Microcebus murinus
Tarsius syrichta
Callithrix jacchus
Macaca mulatta
Nomascus leucogenys
Pongo abelii
Gorilla gorilla
Pan troglodytes
Homo sapiens
Perkinsus marinus ATCC 50983
Tetrahymena thermophila
Paramecium tetraurelia strain d4 2
Neospora caninum Liverpool
Toxoplasma gondii
Cryptosporidium muris RN66
Cryptosporidium parvum Iowa II
Cryptosporidium hominis TU502
Theileria annulata strain Ankara
Theileria parva strain Muguga
Plasmodium falciparum
Plasmodium knowlesi
Plasmodium vivax
Plasmodium chabaudi
Plasmodium yoelii yoelii 17XNL
Plasmodium berghei
Phaeodactylum tricornutum
Thalassiosira pseudonana
Pythium ultimum
Albugo laibachii
Hyaloperonospora arabidopsidis
Phytophthora ramorum
Phytophthora infestans
Phytophthora sojae
Trypanosoma brucei
Trypanosoma cruzi strain CL Brener
Leishmania braziliensis MHOM BR 75 M2904
Leishmania mexicana MHOM GT 2001 U1103
Leishmania major
Leishmania donovani
Leishmania infantum JPCM5
Entamoeba histolytica
Entamoeba dispar SAW760
Dictyostelium discoideum
Dictyostelium purpureum
Trichomonas vaginalis G3
Trichomonas vaginalis
Naegleria gruberi
Giardia lamblia ATCC 50803
OG presence
OG absence
Inferred root
Dscore = 1
Pvalue = 8.71e−06
AdjPvalue = 1.04e−03

NOG142960

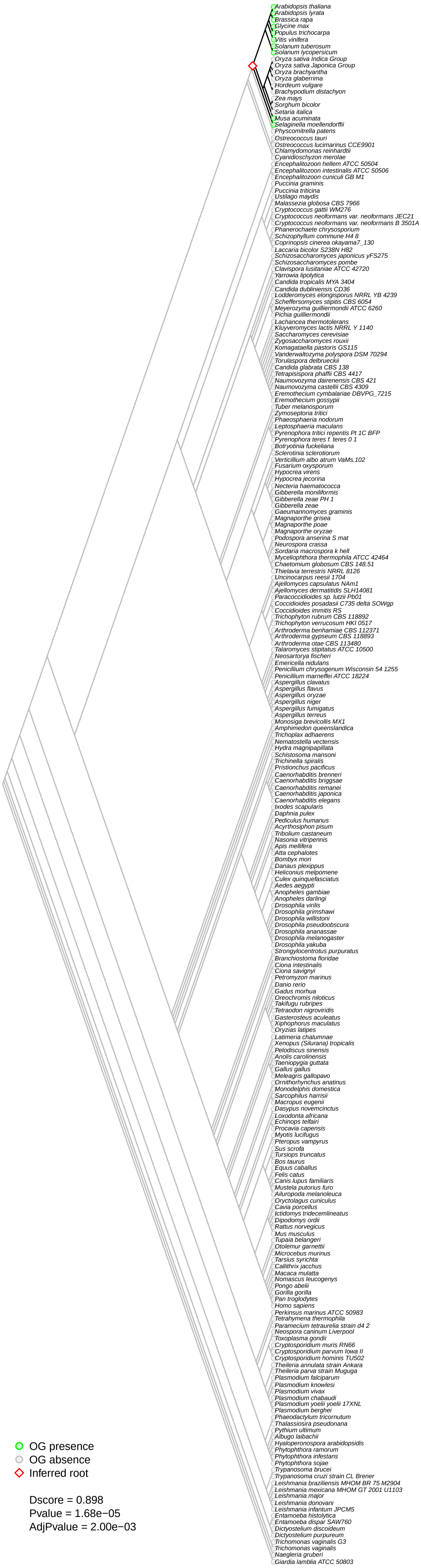
Arabidopsis thaliana
Arabidopsis lyrata
Brassica rapa
Glycine max
Populus trichocarpa
Vitis vinifera
Solanum tuberosum
Solanum lycopersicum
Oryza sativa Indica Group
Oryza sativa Japonica Group
Oryza brachyantha
Oryza glaberrima
Hordeum vulgare
Brachypodium distachyon
Zea mays
Sorghum bicolor
Setaria italica
Musa acuminata
Selaginella moellendorffii
Physcomitrella patens
Ostreococcus tauri
Ostreococcus lucimarinus CCE9901
Chlamydomonas reinhardtii
Cyanidioschyzon merolae
Encephalitozoon hellem ATCC 50504
Encephalitozoon intestinalis ATCC 50506
Encephalitozoon cuniculi GB M1
Puccinia graminis
Puccinia triticina
Ustilago maydis
Malassezia globosa CBS 7966
Cryptococcus gattii WM276
Cryptococcus neoformans var. neoformans JEC21
Cryptococcus neoformans var. neoformans B 3501A
Phanerochaete chrysosporium
Schizophyllum commune H4 8
Coprinopsis cinerea okayama7_130
Laccaria bicolor S238N H82
Schizosaccharomyces japonicus yFS275
Schizosaccharomyces pombe
Clavispora lusitaniae ATCC 42720
Yarrowia lipolytica
Candida tropicalis MYA 3404
Candida dubliniensis CD36
Lodderomyces elongisporus NRRL YB 4239
Scheffersomyces stipitis CBS 6054
Meyerozyma guilliermondii ATCC 6260
Pichia guilliermondii
Lachancea thermotolerans
Kluyveromyces lactis NRRL Y 1140
Saccharomyces cerevisiae
Zygosaccharomyces rouxii
Komagataella pastoris GS115
Vanderwaltozyma polyspora DSM 70294
Torulaspora delbrueckii
Candida glabrata CBS 138
Tetrapisispora phaffii CBS 4417
Naumovozyma dairenensis CBS 421
Naumovozyma castellii CBS 4309
Eremothecium cymbalariae DBVPG_7215
Eremothecium gossypii
Tuber melanosporum
Zymoseptoria tritici
Phaeosphaeria nodorum
Leptosphaeria maculans
Pyrenophora tritici repentis Pt 1C BFP
Pyrenophora teres f. teres 0 1
Botryotinia fuckeliana
Sclerotinia sclerotiorum
Verticillium albo atrum VaMs.102
Fusarium oxysporum
Hypocrea virens
Hypocrea jecorina
Nectria haematococca
Gibberella moniliformis
Gibberella zeae PH 1
Gibberella zeae
Gaeumannomyces graminis
Magnaporthe grisea
Magnaporthe poae
Magnaporthe oryzae
Podospora anserina S mat
Neurospora crassa
Sordaria macrospora k hell
Myceliophthora thermophila ATCC 42464
Chaetomium globosum CBS 148.51
Thielavia terrestris NRRL 8126
Uncinocarpus reesii 1704
Ajellomyces capsulatus NAm1
Ajellomyces dermatitidis SLH14081
Paracoccidioides sp. lutzii Pb01
Coccidioides posadasii C735 delta SOWgp
Coccidioides immitis RS
Trichophyton rubrum CBS 118892
Trichophyton verrucosum HKI 0517
Arthroderma benhamiae CBS 112371
Arthroderma gypseum CBS 118893
Arthroderma otae CBS 113480
Talaromyces stipitatus ATCC 10500
Neosartorya fischeri
Emericella nidulans
Penicillium chrysogenum Wisconsin 54 1255
Penicillium marneffei ATCC 18224
Aspergillus clavatus
Aspergillus flavus
Aspergillus oryzae
Aspergillus niger
Aspergillus fumigatus
Aspergillus terreus
Monosiga brevicollis MX1
Amphimedon queenslandica
Trichoplax adhaerens
Nematostella vectensis
Hydra magnipapillata
Schistosoma mansoni
Trichinella spiralis
Pristionchus pacificus
Caenorhabditis brenneri
Caenorhabditis briggsae
Caenorhabditis remanei
Caenorhabditis japonica
Caenorhabditis elegans
Ixodes scapularis
Daphnia pulex
Pediculus humanus
Acyrthosiphon pisum
Tribolium castaneum
Nasonia vitripennis
Apis mellifera
Atta cephalotes
Bombyx mori
Danaus plexippus
Heliconius melpomene
Culex quinquefasciatus
Aedes aegypti
Anopheles gambiae
Anopheles darlingi
Drosophila virilis
Drosophila grimshawi
Drosophila willistoni
Drosophila pseudoobscura
Drosophila ananassae
Drosophila melanogaster
Drosophila yakuba
Strongylocentrotus purpuratus
Branchiostoma floridae
Ciona intestinalis
Ciona savignyi
Petromyzon marinus
Danio rerio
Gadus morhua
Oreochromis niloticus
Takifugu rubripes
Tetraodon nigroviridis
Gasterosteus aculeatus
Xiphophorus maculatus
Oryzias latipes
Latimeria chalumnae
Xenopus (Silurana) tropicalis
Pelodiscus sinensis
Anolis carolinensis
Taeniopygia guttata
Gallus gallus
Meleagris gallopavo
Ornithorhynchus anatinus
Monodelphis domestica
Sarcophilus harrisii
Macropus eugenii
Dasypus novemcinctus
Loxodonta africana
Echinops telfairi
Procavia capensis
Myotis lucifugus
Pteropus vampyrus
Sus scrofa
Tursiops truncatus
Bos taurus
Equus caballus
Felis catus
Canis lupus familiaris
Mustela putorius furo
Ailuropoda melanoleuca
Oryctolagus cuniculus
Cavia porcellus
Ictidomys tridecemlineatus
Dipodomys ordii
Rattus norvegicus
Mus musculus
Tupaia belangeri
Otolemur garnettii
Microcebus murinus
Tarsius syrichta
Callithrix jacchus
Macaca mulatta
Nomascus leucogenys
Pongo abelii
Gorilla gorilla
Pan troglodytes
Homo sapiens
Perkinsus marinus ATCC 50983
Tetrahymena thermophila
Paramecium tetraurelia strain d4 2
Neospora caninum Liverpool
Toxoplasma gondii
Cryptosporidium muris RN66
Cryptosporidium parvum Iowa II
Cryptosporidium hominis TU502
Theileria annulata strain Ankara
Theileria parva strain Muguga
Plasmodium falciparum
Plasmodium knowlesi
Plasmodium vivax
Plasmodium chabaudi
Plasmodium yoelii yoelii 17XNL
Plasmodium berghei
Phaeodactylum tricornutum
Thalassiosira pseudonana
Pythium ultimum
Albugo laibachii
Hyaloperonospora arabidopsidis
Phytophthora ramorum
Phytophthora infestans
Phytophthora sojae
Trypanosoma brucei
Trypanosoma cruzi strain CL Brener
Leishmania braziliensis MHOM BR 75 M2904
Leishmania mexicana MHOM GT 2001 U1103
Leishmania major
Leishmania donovani
Leishmania infantum JPCM5
Entamoeba histolytica
Entamoeba dispar SAW760
Dictyostelium discoideum
Dictyostelium purpureum
Trichomonas vaginalis G3
Trichomonas vaginalis
Naegleria gruberi
Giardia lamblia ATCC 50803
OG presence
OG absence
Inferred root
Dscore = 0.898
Pvalue = 1.68e−05
AdjPvalue = 2.00e−03

NOG143076

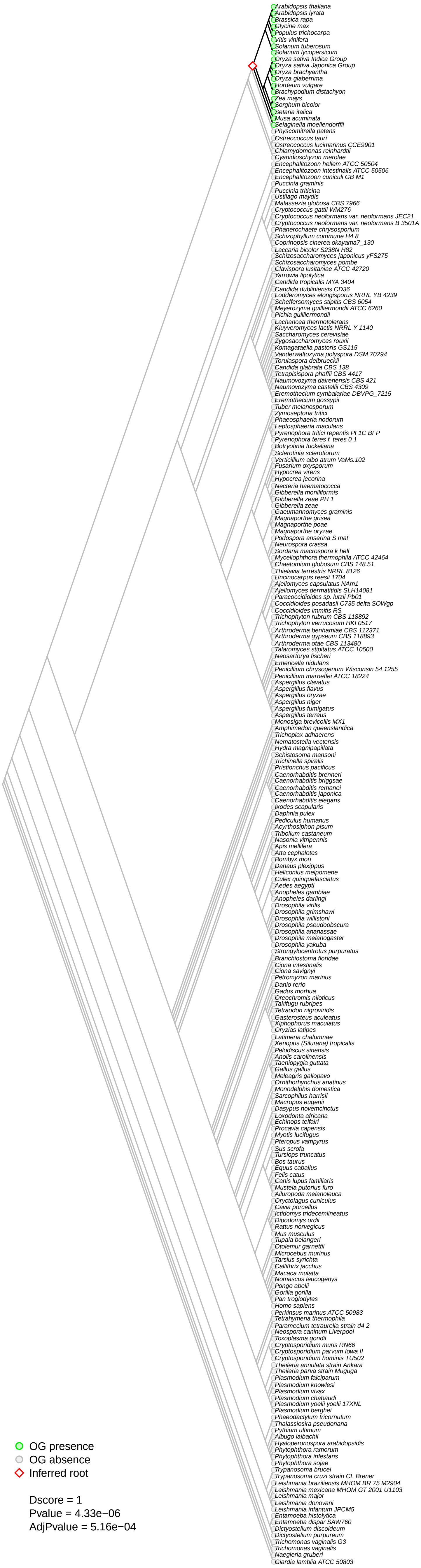

OG presence
OG absence
Inferred root

Dscore = 1
Pvalue = 4.33e−06
AdjPvalue = 5.16e−04

NOG143140

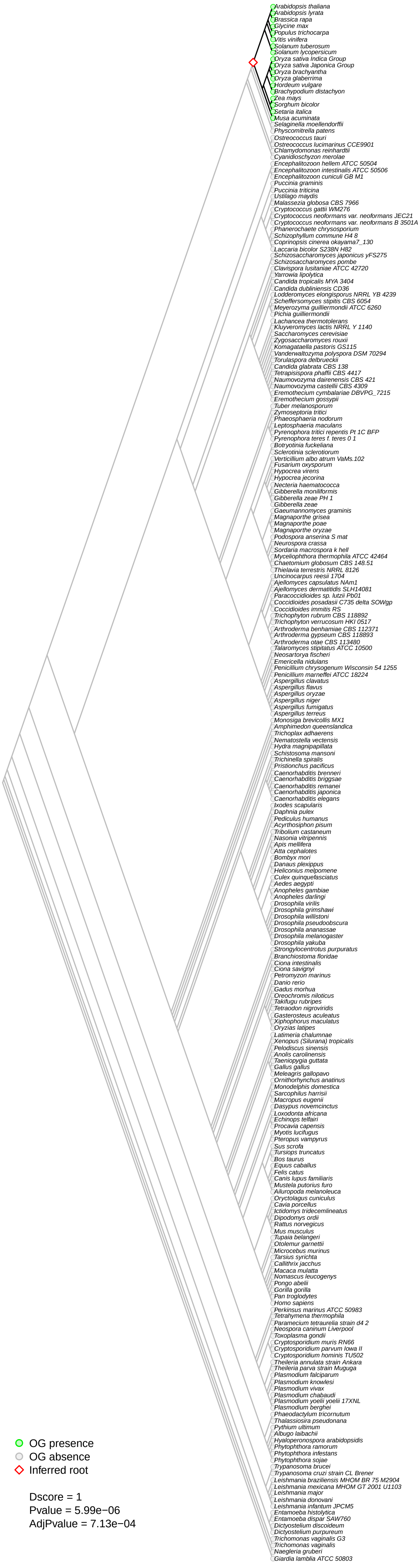

Dscore = 1
Pvalue = 5.99e−06
AdjPvalue = 7.13e−04

NOG143703

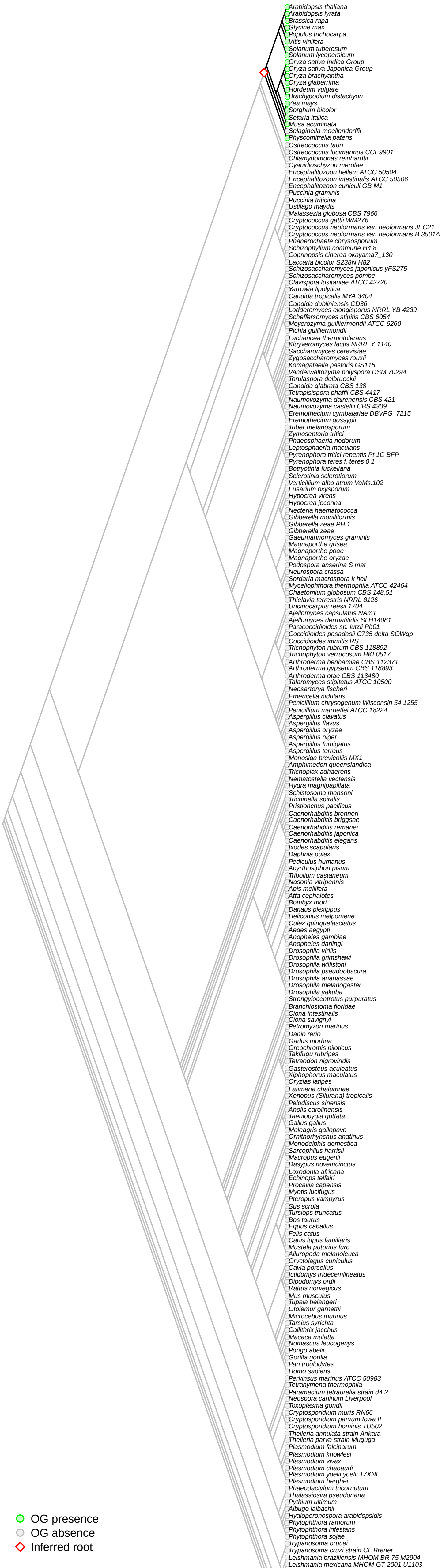

Dscore = 0.889
Pvalue = 4.82e−05
AdjPvalue = 5.73e−03

NOG144070

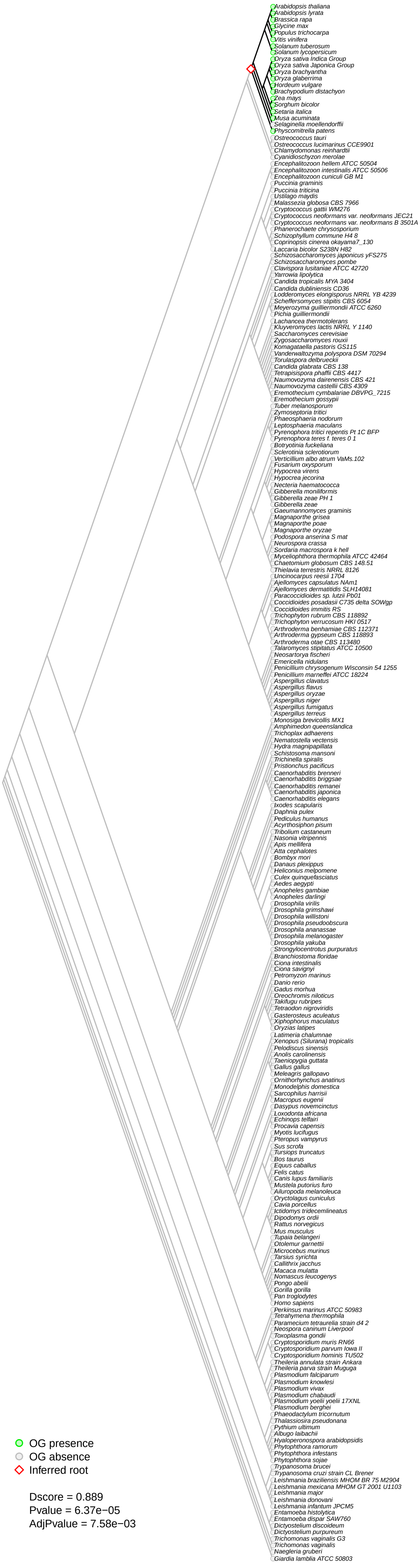

Arabidopsis thaliana
Arabidopsis lyrata
Brassica rapa
Glycine max
Populus trichocarpa
Vitis vinifera
Solanum tuberosum
Solanum lycopersicum
Oryza sativa Indica Group
Oryza sativa Japonica Group
Oryza brachyantha
Oryza glaberrima
Hordeum vulgare
Brachypodium distachyon
Zea mays
Sorghum bicolor
Setaria italica
Musa acuminata
Selaginella moellendorffii
Physcomitrella patens
Ostreococcus tauri
Ostreococcus lucimarinus CCE9901
Chlamydomonas reinhardtii
Cyanidioschyzon merolae
Encephalitozoon hellem ATCC 50504
Encephalitozoon intestinalis ATCC 50506
Encephalitozoon cuniculi GB M1
Puccinia graminis
Puccinia triticina
Ustilago maydis
Malassezia globosa CBS 7966
Cryptococcus gattii WM276
Cryptococcus neoformans var. neoformans JEC21
Cryptococcus neoformans var. neoformans B 3501A
Phanerochaete chrysosporium
Schizophyllum commune H4 8
Coprinopsis cinerea okayama7_130
Laccaria bicolor S238N H82
Schizosaccharomyces japonicus yFS275
Schizosaccharomyces pombe
Clavispora lusitaniae ATCC 42720
Yarrowia lipolytica
Candida tropicalis MYA 3404
Candida dubliniensis CD36
Lodderomyces elongisporus NRRL YB 4239
Scheffersomyces stipitis CBS 6054
Meyerozyma guilliermondii ATCC 6260
Pichia guilliermondii
Lachancea thermotolerans
Kluyveromyces lactis NRRL Y 1140
Saccharomyces cerevisiae
Zygosaccharomyces rouxii
Komagataella pastoris GS115
Vanderwaltozyma polyspora DSM 70294
Torulaspora delbrueckii
Candida glabrata CBS 138
Tetrapisispora phaffii CBS 4417
Naumovozyma dairenensis CBS 421
Naumovozyma castellii CBS 4309
Eremothecium cymbalariae DBVPG_7215
Eremothecium gossypii
Tuber melanosporum
Zymoseptoria tritici
Phaeosphaeria nodorum
Leptosphaeria maculans
Pyrenophora tritici repentis Pt 1C BFP
Pyrenophora teres f. teres 0 1
Botryotinia fuckeliana
Sclerotinia sclerotiorum
Verticillium albo atrum VaMs.102
Fusarium oxysporum
Hypocrea virens
Hypocrea jecorina
Nectria haematococca
Gibberella moniliformis
Gibberella zeae PH 1
Gibberella zeae
Gaeumannomyces graminis
Magnaporthe grisea
Magnaporthe poae
Magnaporthe oryzae
Podospora anserina S mat
Neurospora crassa
Sordaria macrospora k hell
Myceliophthora thermophila ATCC 42464
Chaetomium globosum CBS 148.51
Thielavia terrestris NRRL 8126
Uncinocarpus reesii 1704
Ajellomyces capsulatus NAm1
Ajellomyces dermatitidis SLH14081
Paracoccidioides sp. lutzii Pb01
Coccidioides posadasii C735 delta SOWgp
Coccidioides immitis RS
Trichophyton rubrum CBS 118892
Trichophyton verrucosum HKI 0517
Arthroderma benhamiae CBS 112371
Arthroderma gypseum CBS 118893
Arthroderma otae CBS 113480
Talaromyces stipitatus ATCC 10500
Neosartorya fischeri
Emericella nidulans
Penicillium chrysogenum Wisconsin 54 1255
Penicillium marneffei ATCC 18224
Aspergillus clavatus
Aspergillus flavus
Aspergillus oryzae
Aspergillus niger
Aspergillus fumigatus
Aspergillus terreus
Monosiga brevicollis MX1
Amphimedon queenslandica
Trichoplax adhaerens
Nematostella vectensis
Hydra magnipapillata
Schistosoma mansoni
Trichinella spiralis
Pristionchus pacificus
Caenorhabditis brenneri
Caenorhabditis briggsae
Caenorhabditis remanei
Caenorhabditis japonica
Caenorhabditis elegans
Ixodes scapularis
Daphnia pulex
Pediculus humanus
Acyrthosiphon pisum
Tribolium castaneum
Nasonia vitripennis
Apis mellifera
Atta cephalotes
Bombyx mori
Danaus plexippus
Heliconius melpomene
Culex quinquefasciatus
Aedes aegypti
Anopheles gambiae
Anopheles darlingi
Drosophila virilis
Drosophila grimshawi
Drosophila willistoni
Drosophila pseudoobscura
Drosophila ananassae
Drosophila melanogaster
Drosophila yakuba
Strongylocentrotus purpuratus
Branchiostoma floridae
Ciona intestinalis
Ciona savignyi
Petromyzon marinus
Danio rerio
Gadus morhua
Oreochromis niloticus
Takifugu rubripes
Tetraodon nigroviridis
Gasterosteus aculeatus
Xiphophorus maculatus
Oryzias latipes
Latimeria chalumnae
Xenopus (Silurana) tropicalis
Pelodiscus sinensis
Anolis carolinensis
Taeniopygia guttata
Gallus gallus
Meleagris gallopavo
Ornithorhynchus anatinus
Monodelphis domestica
Sarcophilus harrisii
Macropus eugenii
Dasypus novemcinctus
Loxodonta africana
Echinops telfairi
Procavia capensis
Myotis lucifugus
Pteropus vampyrus
Sus scrofa
Tursiops truncatus
Bos taurus
Equus caballus
Felis catus
Canis lupus familiaris
Mustela putorius furo
Ailuropoda melanoleuca
Oryctolagus cuniculus
Cavia porcellus
Ictidomys tridecemlineatus
Dipodomys ordii
Rattus norvegicus
Mus musculus
Tupaia belangeri
Otolemur garnettii
Microcebus murinus
Tarsius syrichta
Callithrix jacchus
Macaca mulatta
Nomascus leucogenys
Pongo abelii
Gorilla gorilla
Pan troglodytes
Homo sapiens
Perkinsus marinus ATCC 50983
Tetrahymena thermophila
Paramecium tetraurelia strain d4 2
Neospora caninum Liverpool
Toxoplasma gondii
Cryptosporidium muris RN66
Cryptosporidium parvum Iowa II
Cryptosporidium hominis TU502
Theileria annulata strain Ankara
Theileria parva strain Muguga
Plasmodium falciparum
Plasmodium knowlesi
Plasmodium vivax
Plasmodium chabaudi
Plasmodium yoelii yoelii 17XNL
Plasmodium berghei
Phaeodactylum tricornutum
Thalassiosira pseudonana
Pythium ultimum
Albugo laibachii
Hyaloperonospora arabidopsidis
Phytophthora ramorum
Phytophthora infestans
Phytophthora sojae
Trypanosoma brucei
Trypanosoma cruzi strain CL Brener
Leishmania braziliensis MHOM BR 75 M2904
Leishmania mexicana MHOM GT 2001 U1103
Leishmania major
Leishmania donovani
Leishmania infantum JPCM5
Entamoeba histolytica
Entamoeba dispar SAW760
Dictyostelium discoideum
Dictyostelium purpureum
Trichomonas vaginalis G3
Trichomonas vaginalis
Naegleria gruberi
Giardia lamblia ATCC 50803
OG presence
OG absence
Inferred root
Dscore = 0.889
Pvalue = 6.37e−05
AdjPvalue = 7.58e−03

NOG145474

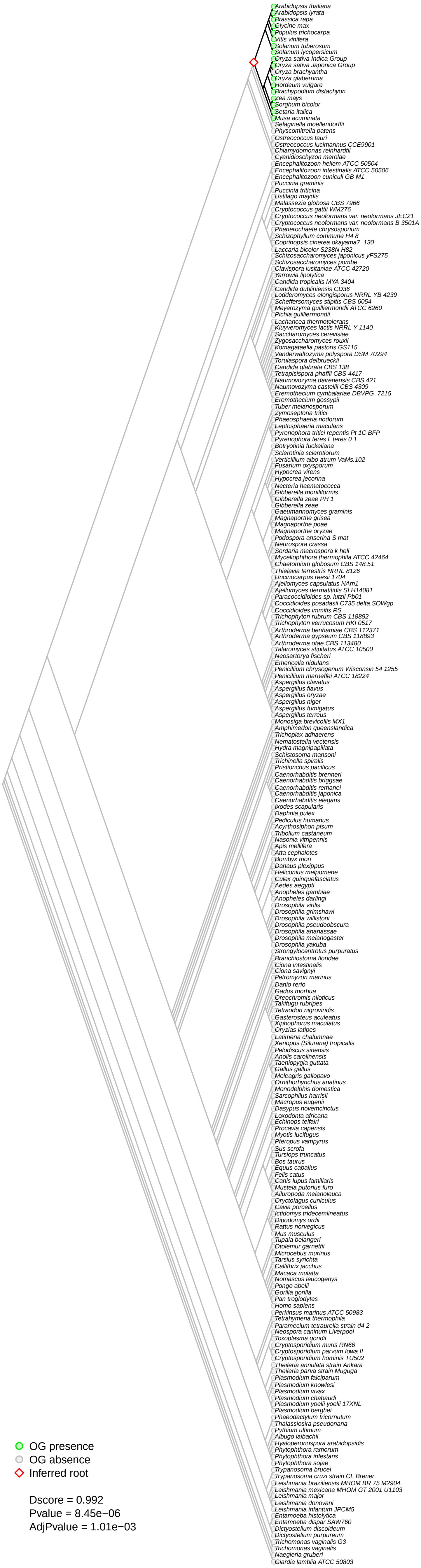

Arabidopsis thaliana
Arabidopsis lyrata
Brassica rapa
Glycine max
Populus trichocarpa
Vitis vinifera
Solanum tuberosum
Solanum lycopersicum
Oryza sativa Indica Group
Oryza sativa Japonica Group
Oryza brachyantha
Oryza glaberrima
Hordeum vulgare
Brachypodium distachyon
Zea mays
Sorghum bicolor
Setaria italica
Musa acuminata
Selaginella moellendorffii
Physcomitrella patens
Ostreococcus tauri
Ostreococcus lucimarinus CCE9901
Chlamydomonas reinhardtii
Cyanidioschyzon merolae
Encephalitozoon hellem ATCC 50504
Encephalitozoon intestinalis ATCC 50506
Encephalitozoon cuniculi GB M1
Puccinia graminis
Puccinia triticina
Ustilago maydis
Malassezia globosa CBS 7966
Cryptococcus gattii WM276
Cryptococcus neoformans var. neoformans JEC21
Cryptococcus neoformans var. neoformans B 3501A
Phanerochaete chrysosporium
Schizophyllum commune H4 8
Coprinopsis cinerea okayama7_130
Laccaria bicolor S238N H82
Schizosaccharomyces japonicus yFS275
Schizosaccharomyces pombe
Clavispora lusitaniae ATCC 42720
Yarrowia lipolytica
Candida tropicalis MYA 3404
Candida dubliniensis CD36
Lodderomyces elongisporus NRRL YB 4239
Scheffersomyces stipitis CBS 6054
Meyerozyma guilliermondii ATCC 6260
Pichia guilliermondii
Lachancea thermotolerans
Kluyveromyces lactis NRRL Y 1140
Saccharomyces cerevisiae
Zygosaccharomyces rouxii
Komagataella pastoris GS115
Vanderwaltozyma polyspora DSM 70294
Torulaspora delbrueckii
Candida glabrata CBS 138
Tetrapisispora phaffii CBS 4417
Naumovozyma dairenensis CBS 421
Naumovozyma castellii CBS 4309
Eremothecium cymbalariae DBVPG_7215
Eremothecium gossypii
Tuber melanosporum
Zymoseptoria tritici
Phaeosphaeria nodorum
Leptosphaeria maculans
Pyrenophora tritici repentis Pt 1C BFP
Pyrenophora teres f. teres 0 1
Botryotinia fuckeliana
Sclerotinia sclerotiorum
Verticillium albo atrum VaMs.102
Fusarium oxysporum
Hypocrea virens
Hypocrea jecorina
Nectria haematococca
Gibberella moniliformis
Gibberella zeae PH 1
Gibberella zeae
Gaeumannomyces graminis
Magnaporthe grisea
Magnaporthe poae
Magnaporthe oryzae
Podospora anserina S mat
Neurospora crassa
Sordaria macrospora k hell
Myceliophthora thermophila ATCC 42464
Chaetomium globosum CBS 148.51
Thielavia terrestris NRRL 8126
Uncinocarpus reesii 1704
Ajellomyces capsulatus NAm1
Ajellomyces dermatitidis SLH14081
Paracoccidioides sp. lutzii Pb01
Coccidioides posadasii C735 delta SOWgp
Coccidioides immitis RS
Trichophyton rubrum CBS 118892
Trichophyton verrucosum HKI 0517
Arthroderma benhamiae CBS 112371
Arthroderma gypseum CBS 118893
Arthroderma otae CBS 113480
Talaromyces stipitatus ATCC 10500
Neosartorya fischeri
Emericella nidulans
Penicillium chrysogenum Wisconsin 54 1255
Penicillium marneffei ATCC 18224
Aspergillus clavatus
Aspergillus flavus
Aspergillus oryzae
Aspergillus niger
Aspergillus fumigatus
Aspergillus terreus
Monosiga brevicollis MX1
Amphimedon queenslandica
Trichoplax adhaerens
Nematostella vectensis
Hydra magnipapillata
Schistosoma mansoni
Trichinella spiralis
Pristionchus pacificus
Caenorhabditis brenneri
Caenorhabditis briggsae
Caenorhabditis remanei
Caenorhabditis japonica
Caenorhabditis elegans
Ixodes scapularis
Daphnia pulex
Pediculus humanus
Acyrthosiphon pisum
Tribolium castaneum
Nasonia vitripennis
Apis mellifera
Atta cephalotes
Bombyx mori
Danaus plexippus
Heliconius melpomene
Culex quinquefasciatus
Aedes aegypti
Anopheles gambiae
Anopheles darlingi
Drosophila virilis
Drosophila grimshawi
Drosophila willistoni
Drosophila pseudoobscura
Drosophila ananassae
Drosophila melanogaster
Drosophila yakuba
Strongylocentrotus purpuratus
Branchiostoma floridae
Ciona intestinalis
Ciona savignyi
Petromyzon marinus
Danio rerio
Gadus morhua
Oreochromis niloticus
Takifugu rubripes
Tetraodon nigroviridis
Gasterosteus aculeatus
Xiphophorus maculatus
Oryzias latipes
Latimeria chalumnae
Xenopus (Silurana) tropicalis
Pelodiscus sinensis
Anolis carolinensis
Taeniopygia guttata
Gallus gallus
Meleagris gallopavo
Ornithorhynchus anatinus
Monodelphis domestica
Sarcophilus harrisii
Macropus eugenii
Dasypus novemcinctus
Loxodonta africana
Echinops telfairi
Procavia capensis
Myotis lucifugus
Pteropus vampyrus
Sus scrofa
Tursiops truncatus
Bos taurus
Equus caballus
Felis catus
Canis lupus familiaris
Mustela putorius furo
Ailuropoda melanoleuca
Oryctolagus cuniculus
Cavia porcellus
Ictidomys tridecemlineatus
Dipodomys ordii
Rattus norvegicus
Mus musculus
Tupaia belangeri
Otolemur garnettii
Microcebus murinus
Tarsius syrichta
Callithrix jacchus
Macaca mulatta
Nomascus leucogenys
Pongo abelii
Gorilla gorilla
Pan troglodytes
Homo sapiens
Perkinsus marinus ATCC 50983
Tetrahymena thermophila
Paramecium tetraurelia strain d4 2
Neospora caninum Liverpool
Toxoplasma gondii
Cryptosporidium muris RN66
Cryptosporidium parvum Iowa II
Cryptosporidium hominis TU502
Theileria annulata strain Ankara
Theileria parva strain Muguga
Plasmodium falciparum
Plasmodium knowlesi
Plasmodium vivax
Plasmodium chabaudi
Plasmodium yoelii yoelii 17XNL
Plasmodium berghei
Phaeodactylum tricornutum
Thalassiosira pseudonana
Pythium ultimum
Albugo laibachii
Hyaloperonospora arabidopsidis
Phytophthora ramorum
Phytophthora infestans
Phytophthora sojae
Trypanosoma brucei
Trypanosoma cruzi strain CL Brener
Leishmania braziliensis MHOM BR 75 M2904
Leishmania mexicana MHOM GT 2001 U1103
Leishmania major
Leishmania donovani
Leishmania infantum JPCM5
Entamoeba histolytica
Entamoeba dispar SAW760
Dictyostelium discoideum
Dictyostelium purpureum
Trichomonas vaginalis G3
Trichomonas vaginalis
Naegleria gruberi
Giardia lamblia ATCC 50803
OG presence
OG absence
Inferred root
Dscore = 0.992
Pvalue = 8.45e−06
AdjPvalue = 1.01e−03

NOG146607

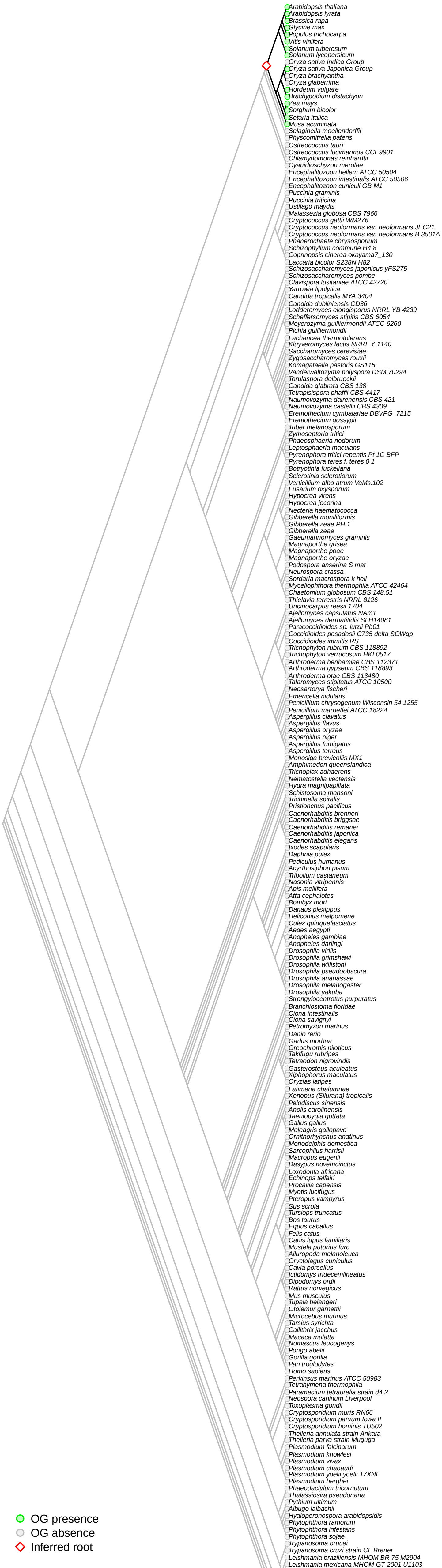

Dscore = 0.975
Pvalue = 5.10e−06
AdjPvalue = 6.07e−04

NOG147190

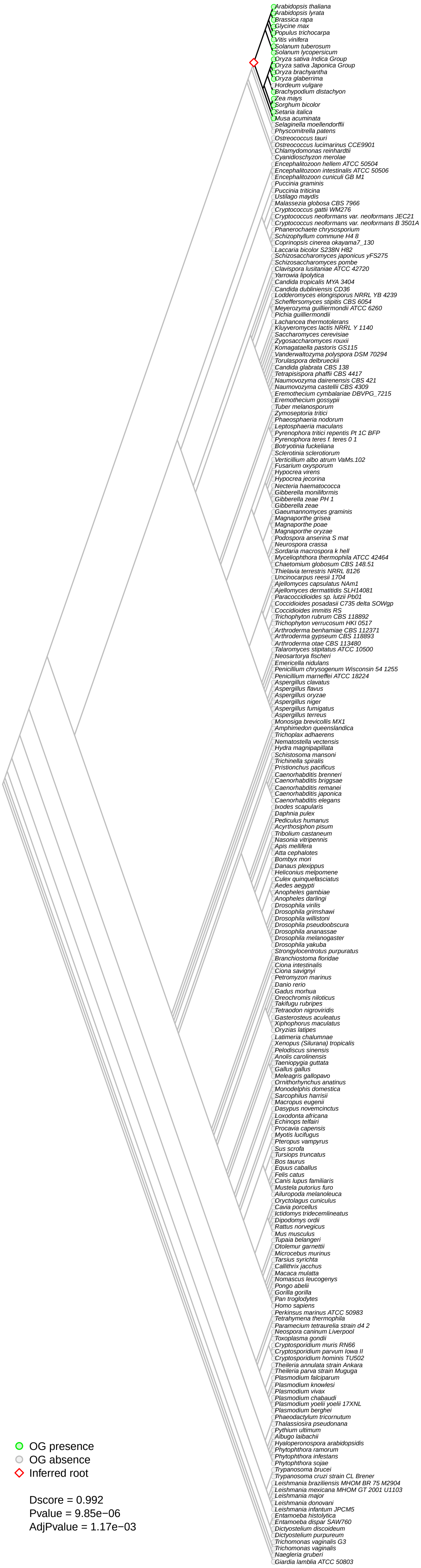

Dscore = 0.992
Pvalue = 9.85e−06
AdjPvalue = 1.17e−03

NOG148203

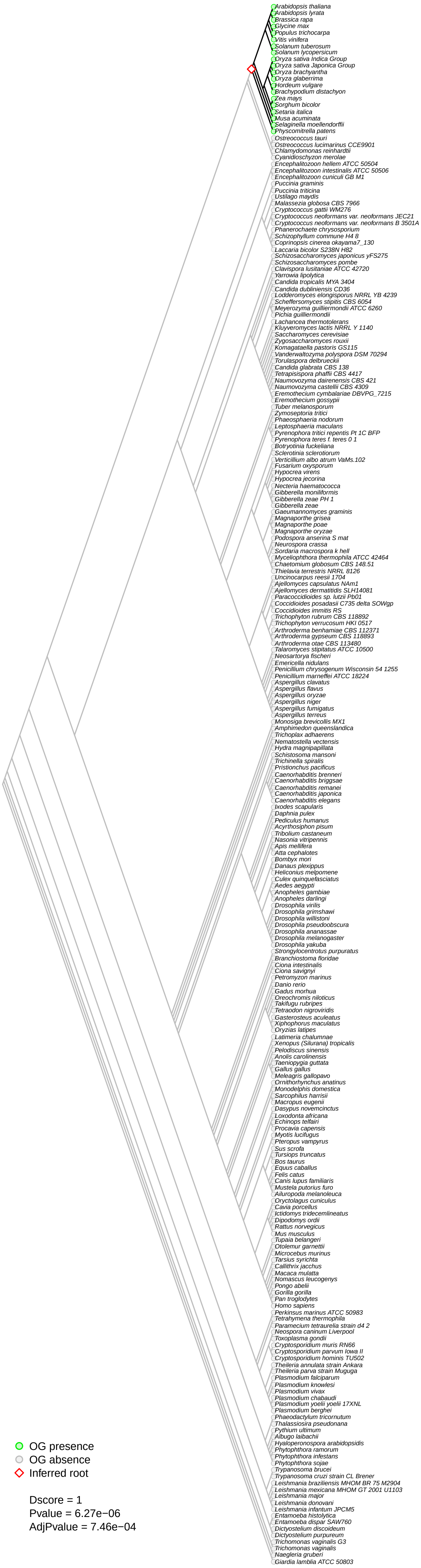
Arabidopsis thaliana
Arabidopsis lyrata
Brassica rapa
Glycine max
Populus trichocarpa
Vitis vinifera
Solanum tuberosum
Solanum lycopersicum
Oryza sativa Indica Group
Oryza sativa Japonica Group
Oryza brachyantha
Oryza glaberrima
Hordeum vulgare
Brachypodium distachyon
Zea mays
Sorghum bicolor
Setaria italica
Musa acuminata
Selaginella moellendorffii
Physcomitrella patens
Ostreococcus tauri
Ostreococcus lucimarinus CCE9901
Chlamydomonas reinhardtii
Cyanidioschyzon merolae
Encephalitozoon hellem ATCC 50504
Encephalitozoon intestinalis ATCC 50506
Encephalitozoon cuniculi GB M1
Puccinia graminis
Puccinia triticina
Ustilago maydis
Malassezia globosa CBS 7966
Cryptococcus gattii WM276
Cryptococcus neoformans var. neoformans JEC21
Cryptococcus neoformans var. neoformans B 3501A
Phanerochaete chrysosporium
Schizophyllum commune H4 8
Coprinopsis cinerea okayama7_130
Laccaria bicolor S238N H82
Schizosaccharomyces japonicus yFS275
Schizosaccharomyces pombe
Clavispora lusitaniae ATCC 42720
Yarrowia lipolytica
Candida tropicalis MYA 3404
Candida dubliniensis CD36
Lodderomyces elongisporus NRRL YB 4239
Scheffersomyces stipitis CBS 6054
Meyerozyma guilliermondii ATCC 6260
Pichia guilliermondii
Lachancea thermotolerans
Kluyveromyces lactis NRRL Y 1140
Saccharomyces cerevisiae
Zygosaccharomyces rouxii
Komagataella pastoris GS115
Vanderwaltozyma polyspora DSM 70294
Torulaspora delbrueckii
Candida glabrata CBS 138
Tetrapisispora phaffii CBS 4417
Naumovozyma dairenensis CBS 421
Naumovozyma castellii CBS 4309
Eremothecium cymbalariae DBVPG_7215
Eremothecium gossypii
Tuber melanosporum
Zymoseptoria tritici
Phaeosphaeria nodorum
Leptosphaeria maculans
Pyrenophora tritici repentis Pt 1C BFP
Pyrenophora teres f. teres 0 1
Botryotinia fuckeliana
Sclerotinia sclerotiorum
Verticillium albo atrum VaMs.102
Fusarium oxysporum
Hypocrea virens
Hypocrea jecorina
Nectria haematococca
Gibberella moniliformis
Gibberella zeae PH 1
Gibberella zeae
Gaeumannomyces graminis
Magnaporthe grisea
Magnaporthe poae
Magnaporthe oryzae
Podospora anserina S mat
Neurospora crassa
Sordaria macrospora k hell
Myceliophthora thermophila ATCC 42464
Chaetomium globosum CBS 148.51
Thielavia terrestris NRRL 8126
Uncinocarpus reesii 1704
Ajellomyces capsulatus NAm1
Ajellomyces dermatitidis SLH14081
Paracoccidioides sp. lutzii Pb01
Coccidioides posadasii C735 delta SOWgp
Coccidioides immitis RS
Trichophyton rubrum CBS 118892
Trichophyton verrucosum HKI 0517
Arthroderma benhamiae CBS 112371
Arthroderma gypseum CBS 118893
Arthroderma otae CBS 113480
Talaromyces stipitatus ATCC 10500
Neosartorya fischeri
Emericella nidulans
Penicillium chrysogenum Wisconsin 54 1255
Penicillium marneffei ATCC 18224
Aspergillus clavatus
Aspergillus flavus
Aspergillus oryzae
Aspergillus niger
Aspergillus fumigatus
Aspergillus terreus
Monosiga brevicollis MX1
Amphimedon queenslandica
Trichoplax adhaerens
Nematostella vectensis
Hydra magnipapillata
Schistosoma mansoni
Trichinella spiralis
Pristionchus pacificus
Caenorhabditis brenneri
Caenorhabditis briggsae
Caenorhabditis remanei
Caenorhabditis japonica
Caenorhabditis elegans
Ixodes scapularis
Daphnia pulex
Pediculus humanus
Acyrthosiphon pisum
Tribolium castaneum
Nasonia vitripennis
Apis mellifera
Atta cephalotes
Bombyx mori
Danaus plexippus
Heliconius melpomene
Culex quinquefasciatus
Aedes aegypti
Anopheles gambiae
Anopheles darlingi
Drosophila virilis
Drosophila grimshawi
Drosophila willistoni
Drosophila pseudoobscura
Drosophila ananassae
Drosophila melanogaster
Drosophila yakuba
Strongylocentrotus purpuratus
Branchiostoma floridae
Ciona intestinalis
Ciona savignyi
Petromyzon marinus
Danio rerio
Gadus morhua
Oreochromis niloticus
Takifugu rubripes
Tetraodon nigroviridis
Gasterosteus aculeatus
Xiphophorus maculatus
Oryzias latipes
Latimeria chalumnae
Xenopus (Silurana) tropicalis
Pelodiscus sinensis
Anolis carolinensis
Taeniopygia guttata
Gallus gallus
Meleagris gallopavo
Ornithorhynchus anatinus
Monodelphis domestica
Sarcophilus harrisii
Macropus eugenii
Dasypus novemcinctus
Loxodonta africana
Echinops telfairi
Procavia capensis
Myotis lucifugus
Pteropus vampyrus
Sus scrofa
Tursiops truncatus
Bos taurus
Equus caballus
Felis catus
Canis lupus familiaris
Mustela putorius furo
Ailuropoda melanoleuca
Oryctolagus cuniculus
Cavia porcellus
Ictidomys tridecemlineatus
Dipodomys ordii
Rattus norvegicus
Mus musculus
Tupaia belangeri
Otolemur garnettii
Microcebus murinus
Tarsius syrichta
Callithrix jacchus
Macaca mulatta
Nomascus leucogenys
Pongo abelii
Gorilla gorilla
Pan troglodytes
Homo sapiens
Perkinsus marinus ATCC 50983
Tetrahymena thermophila
Paramecium tetraurelia strain d4 2
Neospora caninum Liverpool
Toxoplasma gondii
Cryptosporidium muris RN66
Cryptosporidium parvum Iowa II
Cryptosporidium hominis TU502
Theileria annulata strain Ankara
Theileria parva strain Muguga
Plasmodium falciparum
Plasmodium knowlesi
Plasmodium vivax
Plasmodium chabaudi
Plasmodium yoelii yoelii 17XNL
Plasmodium berghei
Phaeodactylum tricornutum
Thalassiosira pseudonana
Pythium ultimum
Albugo laibachii
Hyaloperonospora arabidopsidis
Phytophthora ramorum
Phytophthora infestans
Phytophthora sojae
Trypanosoma brucei
Trypanosoma cruzi strain CL Brener
Leishmania braziliensis MHOM BR 75 M2904
Leishmania mexicana MHOM GT 2001 U1103
Leishmania major
Leishmania donovani
Leishmania infantum JPCM5
Entamoeba histolytica
Entamoeba dispar SAW760
Dictyostelium discoideum
Dictyostelium purpureum
Trichomonas vaginalis G3
Trichomonas vaginalis
Naegleria gruberi
Giardia lamblia ATCC 50803
OG presence
OG absence
Inferred root
Dscore = 1
Pvalue = 6.27e−06
AdjPvalue = 7.46e−04

NOG148585

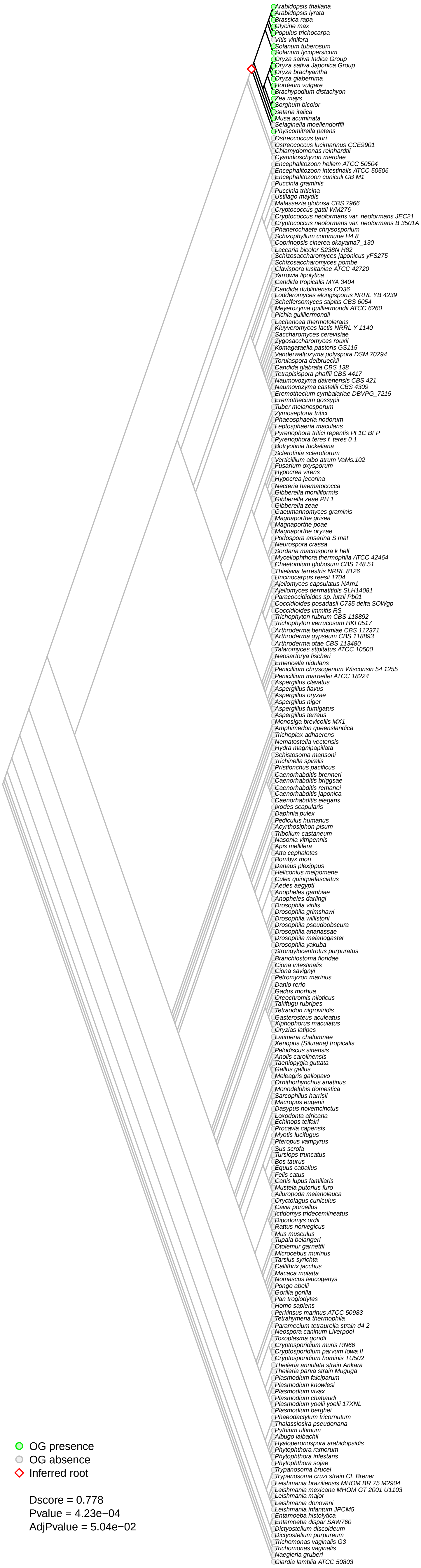

Arabidopsis thaliana
Arabidopsis lyrata
Brassica rapa
Glycine max
Populus trichocarpa
Vitis vinifera
Solanum tuberosum
Solanum lycopersicum
Oryza sativa Indica Group
Oryza sativa Japonica Group
Oryza brachyantha
Oryza glaberrima
Hordeum vulgare
Brachypodium distachyon
Zea mays
Sorghum bicolor
Setaria italica
Musa acuminata
Selaginella moellendorffii
Physcomitrella patens
Ostreococcus tauri
Ostreococcus lucimarinus CCE9901
Chlamydomonas reinhardtii
Cyanidioschyzon merolae
Encephalitozoon hellem ATCC 50504
Encephalitozoon intestinalis ATCC 50506
Encephalitozoon cuniculi GB M1
Puccinia graminis
Puccinia triticina
Ustilago maydis
Malassezia globosa CBS 7966
Cryptococcus gattii WM276
Cryptococcus neoformans var. neoformans JEC21
Cryptococcus neoformans var. neoformans B 3501A
Phanerochaete chrysosporium
Schizophyllum commune H4 8
Coprinopsis cinerea okayama7_130
Laccaria bicolor S238N H82
Schizosaccharomyces japonicus yFS275
Schizosaccharomyces pombe
Clavispora lusitaniae ATCC 42720
Yarrowia lipolytica
Candida tropicalis MYA 3404
Candida dubliniensis CD36
Lodderomyces elongisporus NRRL YB 4239
Scheffersomyces stipitis CBS 6054
Meyerozyma guilliermondii ATCC 6260
Pichia guilliermondii
Lachancea thermotolerans
Kluyveromyces lactis NRRL Y 1140
Saccharomyces cerevisiae
Zygosaccharomyces rouxii
Komagataella pastoris GS115
Vanderwaltozyma polyspora DSM 70294
Torulaspora delbrueckii
Candida glabrata CBS 138
Tetrapisispora phaffii CBS 4417
Naumovozyma dairenensis CBS 421
Naumovozyma castellii CBS 4309
Eremothecium cymbalariae DBVPG_7215
Eremothecium gossypii
Tuber melanosporum
Zymoseptoria tritici
Phaeosphaeria nodorum
Leptosphaeria maculans
Pyrenophora tritici repentis Pt 1C BFP
Pyrenophora teres f. teres 0 1
Botryotinia fuckeliana
Sclerotinia sclerotiorum
Verticillium albo atrum VaMs.102
Fusarium oxysporum
Hypocrea virens
Hypocrea jecorina
Nectria haematococca
Gibberella moniliformis
Gibberella zeae PH 1
Gibberella zeae
Gaeumannomyces graminis
Magnaporthe grisea
Magnaporthe poae
Magnaporthe oryzae
Podospora anserina S mat
Neurospora crassa
Sordaria macrospora k hell
Myceliophthora thermophila ATCC 42464
Chaetomium globosum CBS 148.51
Thielavia terrestris NRRL 8126
Uncinocarpus reesii 1704
Ajellomyces capsulatus NAm1
Ajellomyces dermatitidis SLH14081
Paracoccidioides sp. lutzii Pb01
Coccidioides posadasii C735 delta SOWgp
Coccidioides immitis RS
Trichophyton rubrum CBS 118892
Trichophyton verrucosum HKI 0517
Arthroderma benhamiae CBS 112371
Arthroderma gypseum CBS 118893
Arthroderma otae CBS 113480
Talaromyces stipitatus ATCC 10500
Neosartorya fischeri
Emericella nidulans
Penicillium chrysogenum Wisconsin 54 1255
Penicillium marneffei ATCC 18224
Aspergillus clavatus
Aspergillus flavus
Aspergillus oryzae
Aspergillus niger
Aspergillus fumigatus
Aspergillus terreus
Monosiga brevicollis MX1
Amphimedon queenslandica
Trichoplax adhaerens
Nematostella vectensis
Hydra magnipapillata
Schistosoma mansoni
Trichinella spiralis
Pristionchus pacificus
Caenorhabditis brenneri
Caenorhabditis briggsae
Caenorhabditis remanei
Caenorhabditis japonica
Caenorhabditis elegans
Ixodes scapularis
Daphnia pulex
Pediculus humanus
Acyrthosiphon pisum
Tribolium castaneum
Nasonia vitripennis
Apis mellifera
Atta cephalotes
Bombyx mori
Danaus plexippus
Heliconius melpomene
Culex quinquefasciatus
Aedes aegypti
Anopheles gambiae
Anopheles darlingi
Drosophila virilis
Drosophila grimshawi
Drosophila willistoni
Drosophila pseudoobscura
Drosophila ananassae
Drosophila melanogaster
Drosophila yakuba
Strongylocentrotus purpuratus
Branchiostoma floridae
Ciona intestinalis
Ciona savignyi
Petromyzon marinus
Danio rerio
Gadus morhua
Oreochromis niloticus
Takifugu rubripes
Tetraodon nigroviridis
Gasterosteus aculeatus
Xiphophorus maculatus
Oryzias latipes
Latimeria chalumnae
Xenopus (Silurana) tropicalis
Pelodiscus sinensis
Anolis carolinensis
Taeniopygia guttata
Gallus gallus
Meleagris gallopavo
Ornithorhynchus anatinus
Monodelphis domestica
Sarcophilus harrisii
Macropus eugenii
Dasypus novemcinctus
Loxodonta africana
Echinops telfairi
Procavia capensis
Myotis lucifugus
Pteropus vampyrus
Sus scrofa
Tursiops truncatus
Bos taurus
Equus caballus
Felis catus
Canis lupus familiaris
Mustela putorius furo
Ailuropoda melanoleuca
Oryctolagus cuniculus
Cavia porcellus
Ictidomys tridecemlineatus
Dipodomys ordii
Rattus norvegicus
Mus musculus
Tupaia belangeri
Otolemur garnettii
Microcebus murinus
Tarsius syrichta
Callithrix jacchus
Macaca mulatta
Nomascus leucogenys
Pongo abelii
Gorilla gorilla
Pan troglodytes
Homo sapiens
Perkinsus marinus ATCC 50983
Tetrahymena thermophila
Paramecium tetraurelia strain d4 2
Neospora caninum Liverpool
Toxoplasma gondii
Cryptosporidium muris RN66
Cryptosporidium parvum Iowa II
Cryptosporidium hominis TU502
Theileria annulata strain Ankara
Theileria parva strain Muguga
Plasmodium falciparum
Plasmodium knowlesi
Plasmodium vivax
Plasmodium chabaudi
Plasmodium yoelii yoelii 17XNL
Plasmodium berghei
Phaeodactylum tricornutum
Thalassiosira pseudonana
Pythium ultimum
Albugo laibachii
Hyaloperonospora arabidopsidis
Phytophthora ramorum
Phytophthora infestans
Phytophthora sojae
Trypanosoma brucei
Trypanosoma cruzi strain CL Brener
Leishmania braziliensis MHOM BR 75 M2904
Leishmania mexicana MHOM GT 2001 U1103
Leishmania major
Leishmania donovani
Leishmania infantum JPCM5
Entamoeba histolytica
Entamoeba dispar SAW760
Dictyostelium discoideum
Dictyostelium purpureum
Trichomonas vaginalis G3
Trichomonas vaginalis
Naegleria gruberi
Giardia lamblia ATCC 50803
OG presence
OG absence
Inferred root
Dscore = 0.778
Pvalue = 4.23e−04
AdjPvalue = 5.04e−02

NOG150593

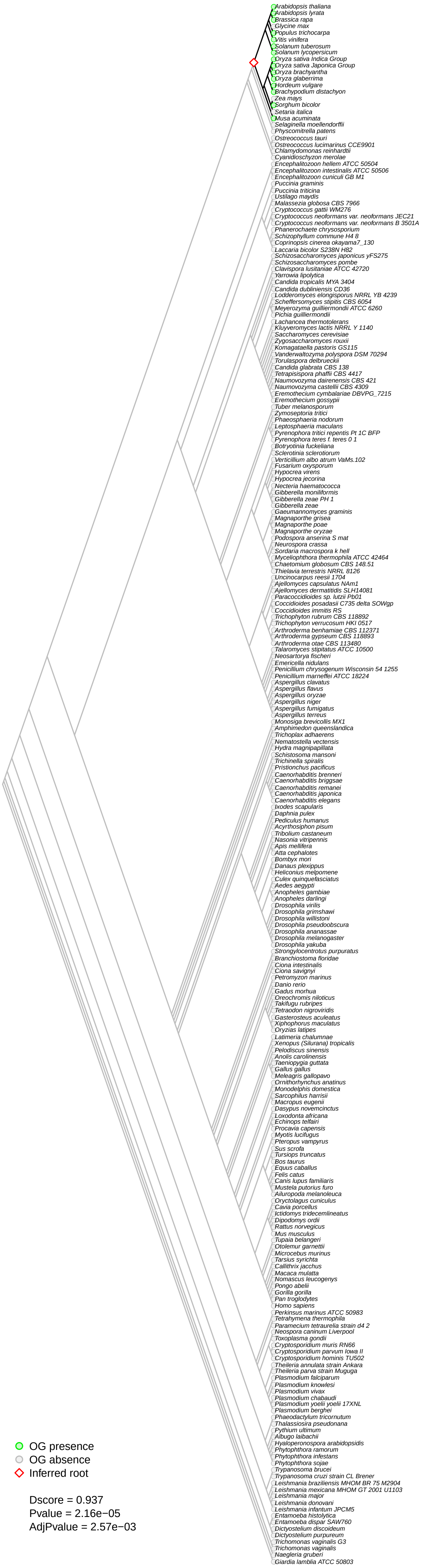

Dscore = 0.937
Pvalue = 2.16e−05
AdjPvalue = 2.57e−03

NOG151739

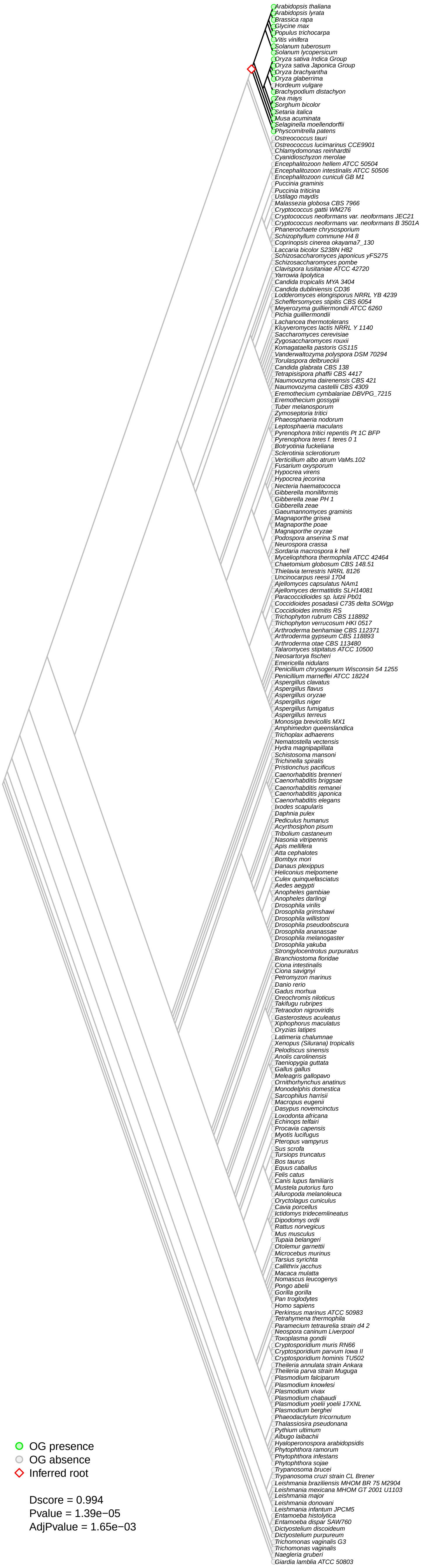

Arabidopsis thaliana
Arabidopsis lyrata
Brassica rapa
Glycine max
Populus trichocarpa
Vitis vinifera
Solanum tuberosum
Solanum lycopersicum
Oryza sativa Indica Group
Oryza sativa Japonica Group
Oryza brachyantha
Oryza glaberrima
Hordeum vulgare
Brachypodium distachyon
Zea mays
Sorghum bicolor
Setaria italica
Musa acuminata
Selaginella moellendorffii
Physcomitrella patens
Ostreococcus tauri
Ostreococcus lucimarinus CCE9901
Chlamydomonas reinhardtii
Cyanidioschyzon merolae
Encephalitozoon hellem ATCC 50504
Encephalitozoon intestinalis ATCC 50506
Encephalitozoon cuniculi GB M1
Puccinia graminis
Puccinia triticina
Ustilago maydis
Malassezia globosa CBS 7966
Cryptococcus gattii WM276
Cryptococcus neoformans var. neoformans JEC21
Cryptococcus neoformans var. neoformans B 3501A
Phanerochaete chrysosporium
Schizophyllum commune H4 8
Coprinopsis cinerea okayama7_130
Laccaria bicolor S238N H82
Schizosaccharomyces japonicus yFS275
Schizosaccharomyces pombe
Clavispora lusitaniae ATCC 42720
Yarrowia lipolytica
Candida tropicalis MYA 3404
Candida dubliniensis CD36
Lodderomyces elongisporus NRRL YB 4239
Scheffersomyces stipitis CBS 6054
Meyerozyma guilliermondii ATCC 6260
Pichia guilliermondii
Lachancea thermotolerans
Kluyveromyces lactis NRRL Y 1140
Saccharomyces cerevisiae
Zygosaccharomyces rouxii
Komagataella pastoris GS115
Vanderwaltozyma polyspora DSM 70294
Torulaspora delbrueckii
Candida glabrata CBS 138
Tetrapisispora phaffii CBS 4417
Naumovozyma dairenensis CBS 421
Naumovozyma castellii CBS 4309
Eremothecium cymbalariae DBVPG_7215
Eremothecium gossypii
Tuber melanosporum
Zymoseptoria tritici
Phaeosphaeria nodorum
Leptosphaeria maculans
Pyrenophora tritici repentis Pt 1C BFP
Pyrenophora teres f. teres 0 1
Botryotinia fuckeliana
Sclerotinia sclerotiorum
Verticillium albo atrum VaMs.102
Fusarium oxysporum
Hypocrea virens
Hypocrea jecorina
Necteria haematococca
Gibberella moniliformis
Gibberella zeae PH 1
Gibberella zeae
Gaeumannomyces graminis
Magnaporthe grisea
Magnaporthe poae
Magnaporthe oryzae
Podospora anserina S mat
Neurospora crassa
Sordaria macrospora k hell
Myceliophthora thermophila ATCC 42464
Chaetomium globosum CBS 148.51
Thielavia terrestris NRRL 8126
Uncinocarpus reesii 1704
Ajellomyces capsulatus NAm1
Ajellomyces dermatitidis SLH14081
Paracoccidioides sp. lutzii Pb01
Coccidioides posadasii C735 delta SOWgp
Coccidioides immitis RS
Trichophyton rubrum CBS 118892
Trichophyton verrucosum HKI 0517
Arthroderma benhamiae CBS 112371
Arthroderma gypseum CBS 118893
Arthroderma otae CBS 113480
Talaromyces stipitatus ATCC 10500
Neosartorya fischeri
Emericella nidulans
Penicillium chrysogenum Wisconsin 54 1255
Penicillium marneffei ATCC 18224
Aspergillus clavatus
Aspergillus flavus
Aspergillus oryzae
Aspergillus niger
Aspergillus fumigatus
Aspergillus terreus
Monosiga brevicollis MX1
Amphimedon queenslandica
Trichoplax adhaerens
Nematostella vectensis
Hydra magnipapillata
Schistosoma mansoni
Trichinella spiralis
Pristionchus pacificus
Caenorhabditis brenneri
Caenorhabditis briggsae
Caenorhabditis remanei
Caenorhabditis japonica
Caenorhabditis elegans
Ixodes scapularis
Daphnia pulex
Pediculus humanus
Acyrthosiphon pisum
Tribolium castaneum
Nasonia vitripennis
Apis mellifera
Atta cephalotes
Bombyx mori
Danaus plexippus
Heliconius melpomene
Culex quinquefasciatus
Aedes aegypti
Anopheles gambiae
Anopheles darlingi
Drosophila virilis
Drosophila grimshawi
Drosophila willistoni
Drosophila pseudoobscura
Drosophila ananassae
Drosophila melanogaster
Drosophila yakuba
Strongylocentrotus purpuratus
Branchiostoma floridae
Ciona intestinalis
Ciona savignyi
Petromyzon marinus
Danio rerio
Gadus morhua
Oreochromis niloticus
Takifugu rubripes
Tetraodon nigroviridis
Gasterosteus aculeatus
Xiphophorus maculatus
Oryzias latipes
Latimeria chalumnae
Xenopus (Silurana) tropicalis
Pelodiscus sinensis
Anolis carolinensis
Taeniopygia guttata
Gallus gallus
Meleagris gallopavo
Ornithorhynchus anatinus
Monodelphis domestica
Sarcophilus harrisii
Macropus eugenii
Dasypus novemcinctus
Loxodonta africana
Echinops telfairi
Procavia capensis
Myotis lucifugus
Pteropus vampyrus
Sus scrofa
Tursiops truncatus
Bos taurus
Equus caballus
Felis catus
Canis lupus familiaris
Mustela putorius furo
Ailuropoda melanoleuca
Oryctolagus cuniculus
Cavia porcellus
Ictidomys tridecemlineatus
Dipodomys ordii
Rattus norvegicus
Mus musculus
Tupaia belangeri
Otolemur garnettii
Microcebus murinus
Tarsius syrichta
Callithrix jacchus
Macaca mulatta
Nomascus leucogenys
Pongo abelii
Gorilla gorilla
Pan troglodytes
Homo sapiens
Perkinsus marinus ATCC 50983
Tetrahymena thermophila
Paramecium tetraurelia strain d4 2
Neospora caninum Liverpool
Toxoplasma gondii
Cryptosporidium muris RN66
Cryptosporidium parvum Iowa II
Cryptosporidium hominis TU502
Theileria annulata strain Ankara
Theileria parva strain Muguga
Plasmodium falciparum
Plasmodium knowlesi
Plasmodium vivax
Plasmodium chabaudi
Plasmodium yoelii yoelii 17XNL
Plasmodium berghei
Phaeodactylum tricornutum
Thalassiosira pseudonana
Pythium ultimum
Albugo laibachii
Hyaloperonospora arabidopsidis
Phytophthora ramorum
Phytophthora infestans
Phytophthora sojae
Trypanosoma brucei
Trypanosoma cruzi strain CL Brener
Leishmania braziliensis MHOM BR 75 M2904
Leishmania mexicana MHOM GT 2001 U1103
Leishmania major
Leishmania donovani
Leishmania infantum JPCM5
Entamoeba histolytica
Entamoeba dispar SAW760
Dictyostelium discoideum
Dictyostelium purpureum
Trichomonas vaginalis G3
Trichomonas vaginalis
Naegleria gruberi
Giardia lamblia ATCC 50803
OG presence
OG absence
Inferred root
Dscore = 0.994
Pvalue = 1.39e−05
AdjPvalue = 1.65e−03

NOG151858

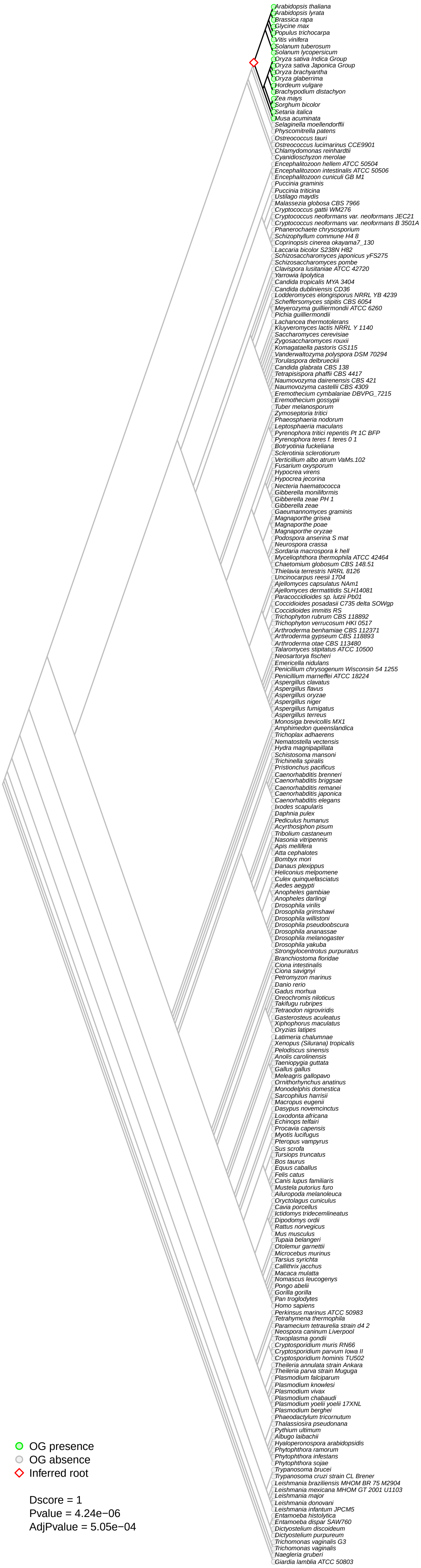

Arabidopsis thaliana
Arabidopsis lyrata
Brassica rapa
Glycine max
Populus trichocarpa
Vitis vinifera
Solanum tuberosum
Solanum lycopersicum
Oryza sativa Indica Group
Oryza sativa Japonica Group
Oryza brachyantha
Oryza glaberrima
Hordeum vulgare
Brachypodium distachyon
Zea mays
Sorghum bicolor
Setaria italica
Musa acuminata
Selaginella moellendorffii
Physcomitrella patens
Ostreococcus tauri
Ostreococcus lucimarinus CCE9901
Chlamydomonas reinhardtii
Cyanidioschyzon merolae
Encephalitozoon hellem ATCC 50504
Encephalitozoon intestinalis ATCC 50506
Encephalitozoon cuniculi GB M1
Puccinia graminis
Puccinia triticina
Ustilago maydis
Malassezia globosa CBS 7966
Cryptococcus gattii WM276
Cryptococcus neoformans var. neoformans JEC21
Cryptococcus neoformans var. neoformans B 3501A
Phanerochaete chrysosporium
Schizophyllum commune H4 8
Coprinopsis cinerea okayama7_130
Laccaria bicolor S238N H82
Schizosaccharomyces japonicus yFS275
Schizosaccharomyces pombe
Clavispora lusitaniae ATCC 42720
Yarrowia lipolytica
Candida tropicalis MYA 3404
Candida dubliniensis CD36
Lodderomyces elongisporus NRRL YB 4239
Scheffersomyces stipitis CBS 6054
Meyerozyma guilliermondii ATCC 6260
Pichia guilliermondii
Lachancea thermotolerans
Kluyveromyces lactis NRRL Y 1140
Saccharomyces cerevisiae
Zygosaccharomyces rouxii
Komagataella pastoris GS115
Vanderwaltozyma polyspora DSM 70294
Torulaspora delbrueckii
Candida glabrata CBS 138
Tetrapisispora phaffii CBS 4417
Naumovozyma dairenensis CBS 421
Naumovozyma castellii CBS 4309
Eremothecium cymbalariae DBVPG_7215
Eremothecium gossypii
Tuber melanosporum
Zymoseptoria tritici
Phaeosphaeria nodorum
Leptosphaeria maculans
Pyrenophora tritici repentis Pt 1C BFP
Pyrenophora teres f. teres 0 1
Botryotinia fuckeliana
Sclerotinia sclerotiorum
Verticillium albo atrum VaMs.102
Fusarium oxysporum
Hypocrea virens
Hypocrea jecorina
Nectria haematococca
Gibberella moniliformis
Gibberella zeae PH 1
Gibberella zeae
Gaeumannomyces graminis
Magnaporthe grisea
Magnaporthe poae
Magnaporthe oryzae
Podospora anserina S mat
Neurospora crassa
Sordaria macrospora k hell
Myceliophthora thermophila ATCC 42464
Chaetomium globosum CBS 148.51
Thielavia terrestris NRRL 8126
Uncinocarpus reesii 1704
Ajellomyces capsulatus NAm1
Ajellomyces dermatitidis SLH14081
Paracoccidioides sp. lutzii Pb01
Coccidioides posadasii C735 delta SOWgp
Coccidioides immitis RS
Trichophyton rubrum CBS 118892
Trichophyton verrucosum HKI 0517
Arthroderma benhamiae CBS 112371
Arthroderma gypseum CBS 118893
Arthroderma otae CBS 113480
Talaromyces stipitatus ATCC 10500
Neosartorya fischeri
Emericella nidulans
Penicillium chrysogenum Wisconsin 54 1255
Penicillium marneffei ATCC 18224
Aspergillus clavatus
Aspergillus flavus
Aspergillus oryzae
Aspergillus niger
Aspergillus fumigatus
Aspergillus terreus
Monosiga brevicollis MX1
Amphimedon queenslandica
Trichoplax adhaerens
Nematostella vectensis
Hydra magnipapillata
Schistosoma mansoni
Trichinella spiralis
Pristionchus pacificus
Caenorhabditis brenneri
Caenorhabditis briggsae
Caenorhabditis remanei
Caenorhabditis japonica
Caenorhabditis elegans
Ixodes scapularis
Daphnia pulex
Pediculus humanus
Acyrthosiphon pisum
Tribolium castaneum
Nasonia vitripennis
Apis mellifera
Atta cephalotes
Bombyx mori
Danaus plexippus
Heliconius melpomene
Culex quinquefasciatus
Aedes aegypti
Anopheles gambiae
Anopheles darlingi
Drosophila virilis
Drosophila grimshawi
Drosophila willistoni
Drosophila pseudoobscura
Drosophila ananassae
Drosophila melanogaster
Drosophila yakuba
Strongylocentrotus purpuratus
Branchiostoma floridae
Ciona intestinalis
Ciona savignyi
Petromyzon marinus
Danio rerio
Gadus morhua
Oreochromis niloticus
Takifugu rubripes
Tetraodon nigroviridis
Gasterosteus aculeatus
Xiphophorus maculatus
Oryzias latipes
Latimeria chalumnae
Xenopus (Silurana) tropicalis
Pelodiscus sinensis
Anolis carolinensis
Taeniopygia guttata
Gallus gallus
Meleagris gallopavo
Ornithorhynchus anatinus
Monodelphis domestica
Sarcophilus harrisii
Macropus eugenii
Dasypus novemcinctus
Loxodonta africana
Echinops telfairi
Procavia capensis
Myotis lucifugus
Pteropus vampyrus
Sus scrofa
Tursiops truncatus
Bos taurus
Equus caballus
Felis catus
Canis lupus familiaris
Mustela putorius furo
Ailuropoda melanoleuca
Oryctolagus cuniculus
Cavia porcellus
Ictidomys tridecemlineatus
Dipodomys ordii
Rattus norvegicus
Mus musculus
Tupaia belangeri
Otolemur garnettii
Microcebus murinus
Tarsius syrichta
Callithrix jacchus
Macaca mulatta
Nomascus leucogenys
Pongo abelii
Gorilla gorilla
Pan troglodytes
Homo sapiens
Perkinsus marinus ATCC 50983
Tetrahymena thermophila
Paramecium tetraurelia strain d4 2
Neospora caninum Liverpool
Toxoplasma gondii
Cryptosporidium muris RN66
Cryptosporidium parvum Iowa II
Cryptosporidium hominis TU502
Theileria annulata strain Ankara
Theileria parva strain Muguga
Plasmodium falciparum
Plasmodium knowlesi
Plasmodium vivax
Plasmodium chabaudi
Plasmodium yoelii yoelii 17XNL
Plasmodium berghei
Phaeodactylum tricornutum
Thalassiosira pseudonana
Pythium ultimum
Albugo laibachii
Hyaloperonospora arabidopsidis
Phytophthora ramorum
Phytophthora infestans
Phytophthora sojae
Trypanosoma brucei
Trypanosoma cruzi strain CL Brener
Leishmania braziliensis MHOM BR 75 M2904
Leishmania mexicana MHOM GT 2001 U1103
Leishmania major
Leishmania donovani
Leishmania infantum JPCM5
Entamoeba histolytica
Entamoeba dispar SAW760
Dictyostelium discoideum
Dictyostelium purpureum
Trichomonas vaginalis G3
Trichomonas vaginalis
Naegleria gruberi
Giardia lamblia ATCC 50803
OG presence
OG absence
Inferred root
Dscore = 1
Pvalue = 4.24e−06
AdjPvalue = 5.05e−04

NOG152378

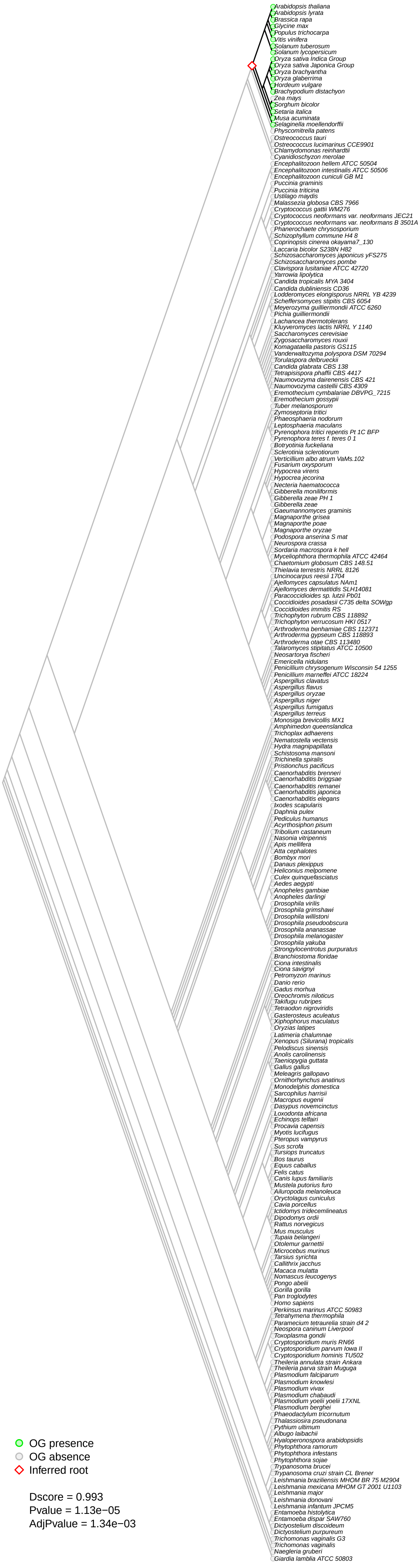
Arabidopsis thaliana
Arabidopsis lyrata
Brassica rapa
Glycine max
Populus trichocarpa
Vitis vinifera
Solanum tuberosum
Solanum lycopersicum
Oryza sativa Indica Group
Oryza sativa Japonica Group
Oryza brachyantha
Oryza glaberrima
Hordeum vulgare
Brachypodium distachyon
Zea mays
Sorghum bicolor
Setaria italica
Musa acuminata
Selaginella moellendorffii
Physcomitrella patens
Ostreococcus tauri
Ostreococcus lucimarinus CCE9901
Chlamydomonas reinhardtii
Cyanidioschyzon merolae
Encephalitozoon hellem ATCC 50504
Encephalitozoon intestinalis ATCC 50506
Encephalitozoon cuniculi GB M1
Puccinia graminis
Puccinia triticina
Ustilago maydis
Malassezia globosa CBS 7966
Cryptococcus gattii WM276
Cryptococcus neoformans var. neoformans JEC21
Cryptococcus neoformans var. neoformans B 3501A
Phanerochaete chrysosporium
Schizophyllum commune H4 8
Coprinopsis cinerea okayama7_130
Laccaria bicolor S238N H82
Schizosaccharomyces japonicus yFS275
Schizosaccharomyces pombe
Clavispora lusitaniae ATCC 42720
Yarrowia lipolytica
Candida tropicalis MYA 3404
Candida dubliniensis CD36
Lodderomyces elongisporus NRRL YB 4239
Scheffersomyces stipitis CBS 6054
Meyerozyma guilliermondii ATCC 6260
Pichia guilliermondii
Lachancea thermotolerans
Kluyveromyces lactis NRRL Y 1140
Saccharomyces cerevisiae
Zygosaccharomyces rouxii
Komagataella pastoris GS115
Vanderwaltozyma polyspora DSM 70294
Torulaspora delbrueckii
Candida glabrata CBS 138
Tetrapisispora phaffii CBS 4417
Naumovozyma dairenensis CBS 421
Naumovozyma castellii CBS 4309
Eremothecium cymbalariae DBVPG_7215
Eremothecium gossypii
Tuber melanosporum
Zymoseptoria tritici
Phaeosphaeria nodorum
Leptosphaeria maculans
Pyrenophora tritici repentis Pt 1C BFP
Pyrenophora teres f. teres 0 1
Botryotinia fuckeliana
Sclerotinia sclerotiorum
Verticillium albo atrum VaMs.102
Fusarium oxysporum
Hypocrea virens
Hypocrea jecorina
Nectria haematococca
Gibberella moniliformis
Gibberella zeae PH 1
Gibberella zeae
Gaeumannomyces graminis
Magnaporthe grisea
Magnaporthe poae
Magnaporthe oryzae
Podospora anserina S mat
Neurospora crassa
Sordaria macrospora k hell
Myceliophthora thermophila ATCC 42464
Chaetomium globosum CBS 148.51
Thielavia terrestris NRRL 8126
Uncinocarpus reesii 1704
Ajellomyces capsulatus NAm1
Ajellomyces dermatitidis SLH14081
Paracoccidioides sp. lutzii Pb01
Coccidioides posadasii C735 delta SOWgp
Coccidioides immitis RS
Trichophyton rubrum CBS 118892
Trichophyton verrucosum HKI 0517
Arthroderma benhamiae CBS 112371
Arthroderma gypseum CBS 118893
Arthroderma otae CBS 113480
Talaromyces stipitatus ATCC 10500
Neosartorya fischeri
Emericella nidulans
Penicillium chrysogenum Wisconsin 54 1255
Penicillium marneffei ATCC 18224
Aspergillus clavatus
Aspergillus flavus
Aspergillus oryzae
Aspergillus niger
Aspergillus fumigatus
Aspergillus terreus
Monosiga brevicollis MX1
Amphimedon queenslandica
Trichoplax adhaerens
Nematostella vectensis
Hydra magnipapillata
Schistosoma mansoni
Trichinella spiralis
Pristionchus pacificus
Caenorhabditis brenneri
Caenorhabditis briggsae
Caenorhabditis remanei
Caenorhabditis japonica
Caenorhabditis elegans
Ixodes scapularis
Daphnia pulex
Pediculus humanus
Acyrthosiphon pisum
Tribolium castaneum
Nasonia vitripennis
Apis mellifera
Atta cephalotes
Bombyx mori
Danaus plexippus
Heliconius melpomene
Culex quinquefasciatus
Aedes aegypti
Anopheles gambiae
Anopheles darlingi
Drosophila virilis
Drosophila grimshawi
Drosophila willistoni
Drosophila pseudoobscura
Drosophila ananassae
Drosophila melanogaster
Drosophila yakuba
Strongylocentrotus purpuratus
Branchiostoma floridae
Ciona intestinalis
Ciona savignyi
Petromyzon marinus
Danio rerio
Gadus morhua
Oreochromis niloticus
Takifugu rubripes
Tetraodon nigroviridis
Gasterosteus aculeatus
Xiphophorus maculatus
Oryzias latipes
Latimeria chalumnae
Xenopus (Silurana) tropicalis
Pelodiscus sinensis
Anolis carolinensis
Taeniopygia guttata
Gallus gallus
Meleagris gallopavo
Ornithorhynchus anatinus
Monodelphis domestica
Sarcophilus harrisii
Macropus eugenii
Dasypus novemcinctus
Loxodonta africana
Echinops telfairi
Procavia capensis
Myotis lucifugus
Pteropus vampyrus
Sus scrofa
Tursiops truncatus
Bos taurus
Equus caballus
Felis catus
Canis lupus familiaris
Mustela putorius furo
Ailuropoda melanoleuca
Oryctolagus cuniculus
Cavia porcellus
Ictidomys tridecemlineatus
Dipodomys ordii
Rattus norvegicus
Mus musculus
Tupaia belangeri
Otolemur garnettii
Microcebus murinus
Tarsius syrichta
Callithrix jacchus
Macaca mulatta
Nomascus leucogenys
Pongo abelii
Gorilla gorilla
Pan troglodytes
Homo sapiens
Perkinsus marinus ATCC 50983
Tetrahymena thermophila
Paramecium tetraurelia strain d4 2
Neospora caninum Liverpool
Toxoplasma gondii
Cryptosporidium muris RN66
Cryptosporidium parvum Iowa II
Cryptosporidium hominis TU502
Theileria annulata strain Ankara
Theileria parva strain Muguga
Plasmodium falciparum
Plasmodium knowlesi
Plasmodium vivax
Plasmodium chabaudi
Plasmodium yoelii yoelii 17XNL
Plasmodium berghei
Phaeodactylum tricornutum
Thalassiosira pseudonana
Pythium ultimum
Albugo laibachii
Hyaloperonospora arabidopsidis
Phytophthora ramorum
Phytophthora infestans
Phytophthora sojae
Trypanosoma brucei
Trypanosoma cruzi strain CL Brener
Leishmania braziliensis MHOM BR 75 M2904
Leishmania mexicana MHOM GT 2001 U1103
Leishmania major
Leishmania donovani
Leishmania infantum JPCM5
Entamoeba histolytica
Entamoeba dispar SAW760
Dictyostelium discoideum
Dictyostelium purpureum
Trichomonas vaginalis G3
Trichomonas vaginalis
Naegleria gruberi
Giardia lamblia ATCC 50803
OG presence
OG absence
Inferred root
Dscore = 0.993
Pvalue = 1.13e−05
AdjPvalue = 1.34e−03

NOG157462

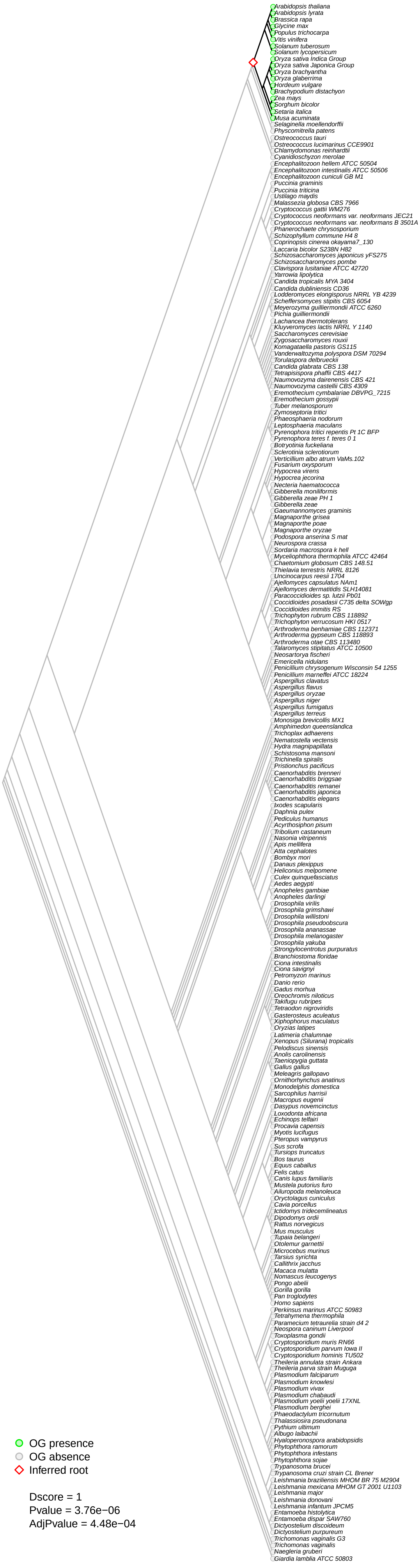
Arabidopsis thaliana
Arabidopsis lyrata
Brassica rapa
Glycine max
Populus trichocarpa
Vitis vinifera
Solanum tuberosum
Solanum lycopersicum
Oryza sativa Indica Group
Oryza sativa Japonica Group
Oryza brachyantha
Oryza glaberrima
Hordeum vulgare
Brachypodium distachyon
Zea mays
Sorghum bicolor
Setaria italica
Musa acuminata
Selaginella moellendorffii
Physcomitrella patens
Ostreococcus tauri
Ostreococcus lucimarinus CCE9901
Chlamydomonas reinhardtii
Cyanidioschyzon merolae
Encephalitozoon hellem ATCC 50504
Encephalitozoon intestinalis ATCC 50506
Encephalitozoon cuniculi GB M1
Puccinia graminis
Puccinia triticina
Ustilago maydis
Malassezia globosa CBS 7966
Cryptococcus gattii WM276
Cryptococcus neoformans var. neoformans JEC21
Cryptococcus neoformans var. neoformans B 3501A
Phanerochaete chrysosporium
Schizophyllum commune H 8
Coprinopsis cinerea okayama7_130
Laccaria bicolor S238N H82
Schizosaccharomyces japonicus yFS275
Schizosaccharomyces pombe
Clavispora lusitaniae ATCC 42720
Yarrowia lipolytica
Candida tropicalis MYA 3404
Candida dubliniensis CD36
Lodderomyces elongisporus NRRL YB 4239
Scheffersomyces stipitis CBS 6054
Meyerozyma guilliermondii ATCC 6260
Pichia guilliermondii
Lachancea thermotolerans
Kluyveromyces lactis NRRL Y 1140
Saccharomyces cerevisiae
Zygosaccharomyces rouxii
Komagataella pastoris GS115
Vanderwaltozyma polyspora DSM 70294
Torulaspora delbrueckii
Candida glabrata CBS 138
Tetrapisispora phaffii CBS 4417
Naumovozyma dairenensis CBS 421
Naumovozyma castellii CBS 4309
Eremothecium cymbalariae DBVPG_7215
Eremothecium gossypii
Tuber melanosporum
Zymoseptoria tritici
Phaeosphaeria nodorum
Leptosphaeria maculans
Pyrenophora tritici repentis Pt 1C BFP
Pyrenophora teres f. teres 0 1
Botryotinia fuckeliana
Sclerotinia sclerotiorum
Verticillium albo atrum VaMs.102
Fusarium oxysporum
Hypocrea virens
Hypocrea jecorina
Nectria haematococca
Gibberella moniliformis
Gibberella zeae PH 1
Gibberella zeae
Gaeumannomyces graminis
Magnaporthe grisea
Magnaporthe poae
Magnaporthe oryzae
Podospora anserina S mat
Neurospora crassa
Sordaria macrospora k hell
Myceliophthora thermophila ATCC 42464
Chaetomium globosum CBS 148.51
Thielavia terrestris NRRL 8126
Uncinocarpus reesii 1704
Ajellomyces capsulatus NAm1
Ajellomyces dermatitidis SLH14081
Paracoccidioides sp. lutzii Pb01
Coccidioides posadasii C735 delta SOWgp
Coccidioides immitis RS
Trichophyton rubrum CBS 118892
Trichophyton verrucosum HKI 0517
Arthroderma benhamiae CBS 112371
Arthroderma gypseum CBS 118893
Arthroderma otae CBS 113480
Talaromyces stipitatus ATCC 10500
Neosartorya fischeri
Emericella nidulans
Penicillium chrysogenum Wisconsin 54 1255
Penicillium marneffei ATCC 18224
Aspergillus clavatus
Aspergillus flavus
Aspergillus oryzae
Aspergillus niger
Aspergillus fumigatus
Aspergillus terreus
Monosiga brevicollis MX1
Amphimedon queenslandica
Trichoplax adhaerens
Nematostella vectensis
Hydra magnipapillata
Schistosoma mansoni
Trichinella spiralis
Pristionchus pacificus
Caenorhabditis brenneri
Caenorhabditis briggsae
Caenorhabditis remanei
Caenorhabditis japonica
Caenorhabditis elegans
Ixodes scapularis
Daphnia pulex
Pediculus humanus
Acyrthosiphon pisum
Tribolium castaneum
Nasonia vitripennis
Apis mellifera
Atta cephalotes
Bombyx mori
Danaus plexippus
Heliconius melpomene
Culex quinquefasciatus
Aedes aegypti
Anopheles gambiae
Anopheles darlingi
Drosophila virilis
Drosophila grimshawi
Drosophila willistoni
Drosophila pseudoobscura
Drosophila ananassae
Drosophila melanogaster
Drosophila yakuba
Strongylocentrotus purpuratus
Branchiostoma floridae
Ciona intestinalis
Ciona savignyi
Petromyzon marinus
Danio rerio
Gadus morhua
Oreochromis niloticus
Takifugu rubripes
Tetraodon nigroviridis
Gasterosteus aculeatus
Xiphophorus maculatus
Oryzias latipes
Latimeria chalumnae
Xenopus (Silurana) tropicalis
Pelodiscus sinensis
Anolis carolinensis
Taeniopygia guttata
Gallus gallus
Meleagris gallopavo
Ornithorhynchus anatinus
Monodelphis domestica
Sarcophilus harrisii
Macropus eugenii
Dasypus novemcinctus
Loxodonta africana
Echinops telfairi
Procavia capensis
Myotis lucifugus
Pteropus vampyrus
Sus scrofa
Tursiops truncatus
Bos taurus
Equus caballus
Felis catus
Canis lupus familiaris
Mustela putorius furo
Ailuropoda melanoleuca
Oryctolagus cuniculus
Cavia porcellus
Ictidomys tridecemlineatus
Dipodomys ordii
Rattus norvegicus
Mus musculus
Tupaia belangeri
Otolemur garnettii
Microcebus murinus
Tarsius syrichta
Callithrix jacchus
Macaca mulatta
Nomascus leucogenys
Pongo abelii
Gorilla gorilla
Pan troglodytes
Homo sapiens
Perkinsus marinus ATCC 50983
Tetrahymena thermophila
Paramecium tetraurelia strain d4 2
Neospora caninum Liverpool
Toxoplasma gondii
Cryptosporidium muris RN66
Cryptosporidium parvum Iowa II
Cryptosporidium hominis TU502
Theileria annulata strain Ankara
Theileria parva strain Muguga
Plasmodium falciparum
Plasmodium knowlesi
Plasmodium vivax
Plasmodium chabaudi
Plasmodium yoelii yoelii 17XNL
Plasmodium berghei
Phaeodactylum tricornutum
Thalassiosira pseudonana
Pythium ultimum
Albugo laibachii
Hyaloperonospora arabidopsidis
Phytophthora ramorum
Phytophthora infestans
Phytophthora sojae
Trypanosoma brucei
Trypanosoma cruzi strain CL Brener
Leishmania braziliensis MHOM BR 75 M2904
Leishmania mexicana MHOM GT 2001 U1103
Leishmania major
Leishmania donovani
Leishmania infantum JPCM5
Entamoeba histolytica
Entamoeba dispar SAW760
Dictyostelium discoideum
Dictyostelium purpureum
Trichomonas vaginalis G3
Trichomonas vaginalis
Naegleria gruberi
Giardia lamblia ATCC 50803
OG presence
OG absence
Inferred root
Dscore = 1
Pvalue = 3.76e−06
AdjPvalue = 4.48e−04

NOG158362

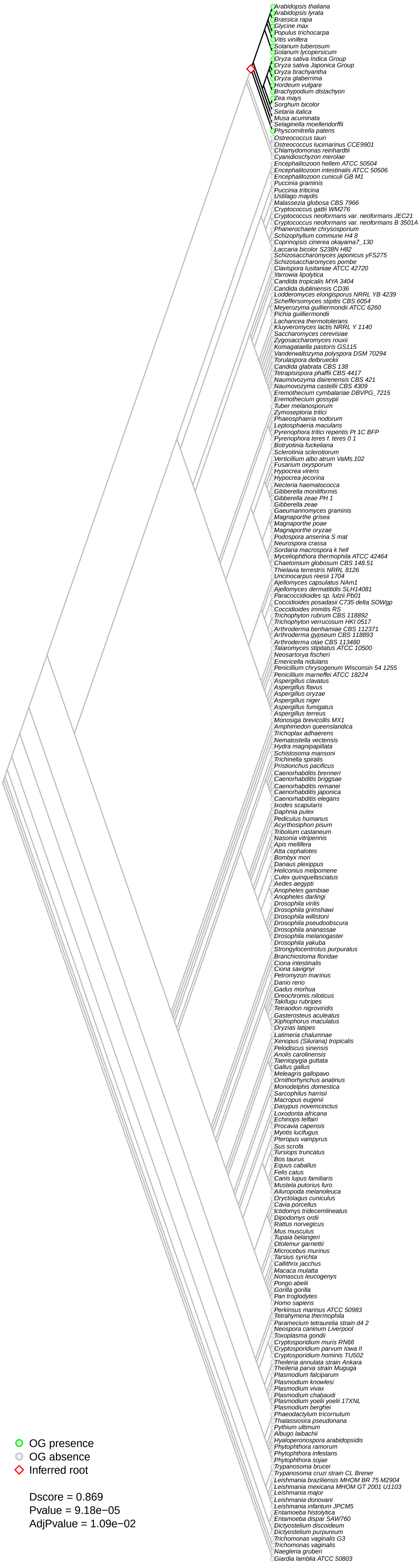

Arabidopsis thaliana
Arabidopsis lyrata
Brassica rapa
Glycine max
Populus trichocarpa
Vitis vinifera
Solanum tuberosum
Solanum lycopersicum
Oryza sativa Indica Group
Oryza sativa Japonica Group
Oryza brachyantha
Oryza glaberrima
Hordeum vulgare
Brachypodium distachyon
Zea mays
Sorghum bicolor
Setaria italica
Musa acuminata
Selaginella moellendorffii
Physcomitrella patens
Ostreococcus tauri
Ostreococcus lucimarinus CCE9901
Chlamydomonas reinhardtii
Cyanidioschyzon merolae
Encephalitozoon hellem ATCC 50504
Encephalitozoon intestinalis ATCC 50506
Encephalitozoon cuniculi GB M1
Puccinia graminis
Puccinia triticina
Ustilago maydis
Malassezia globosa CBS 7966
Cryptococcus gattii WM276
Cryptococcus neoformans var. neoformans JEC21
Cryptococcus neoformans var. neoformans B 3501A
Phanerochaete chrysosporium
Schizophyllum commune H4 8
Coprinopsis cinerea okayama7_130
Laccaria bicolor S238N H82
Schizosaccharomyces japonicus yFS275
Schizosaccharomyces pombe
Clavispora lusitaniae ATCC 42720
Yarrowia lipolytica
Candida tropicalis MYA 3404
Candida dubliniensis CD36
Lodderomyces elongisporus NRRL YB 4239
Scheffersomyces stipitis CBS 6054
Meyerozyma guilliermondii ATCC 6260
Pichia guilliermondii
Lachancea thermotolerans
Kluyveromyces lactis NRRL Y 1140
Saccharomyces cerevisiae
Zygosaccharomyces rouxii
Komagataella pastoris GS115
Vanderwaltozyma polyspora DSM 70294
Torulaspora delbrueckii
Candida glabrata CBS 138
Tetrapisispora phaffii CBS 4417
Naumovozyma dairenensis CBS 421
Naumovozyma castellii CBS 4309
Eremothecium cymbalariae DBVPG_7215
Eremothecium gossypii
Tuber melanosporum
Zymoseptoria tritici
Phaeosphaeria nodorum
Leptosphaeria maculans
Pyrenophora tritici repentis Pt 1C BFP
Pyrenophora teres f. teres 0 1
Botryotinia fuckeliana
Sclerotinia sclerotiorum
Verticillium albo atrum VaMs.102
Fusarium oxysporum
Hypocrea virens
Hypocrea jecorina
Nectria haematococca
Gibberella moniliformis
Gibberella zeae PH 1
Gibberella zeae
Gaeumannomyces graminis
Magnaporthe grisea
Magnaporthe poae
Magnaporthe oryzae
Podospora anserina S mat
Neurospora crassa
Sordaria macrospora k hell
Myceliophthora thermophila ATCC 42464
Chaetomium globosum CBS 148.51
Thielavia terrestris NRRL 8126
Uncinocarpus reesii 1704
Ajellomyces capsulatus NAm1
Ajellomyces dermatitidis SLH14081
Paracoccidioides sp. lutzii Pb01
Coccidioides posadasii C735 delta SOWgp
Coccidioides immitis RS
Trichophyton rubrum CBS 118892
Trichophyton verrucosum HKI 0517
Arthroderma benhamiae CBS 112371
Arthroderma gypseum CBS 118893
Arthroderma otae CBS 113480
Talaromyces stipitatus ATCC 10500
Neosartorya fischeri
Emericella nidulans
Penicillium chrysogenum Wisconsin 54 1255
Penicillium marneffei ATCC 18224
Aspergillus clavatus
Aspergillus flavus
Aspergillus oryzae
Aspergillus niger
Aspergillus fumigatus
Aspergillus terreus
Monosiga brevicollis MX1
Amphimedon queenslandica
Trichoplax adhaerens
Nematostella vectensis
Hydra magnipapillata
Schistosoma mansoni
Trichinella spiralis
Pristionchus pacificus
Caenorhabditis brenneri
Caenorhabditis briggsae
Caenorhabditis remanei
Caenorhabditis japonica
Caenorhabditis elegans
Ixodes scapularis
Daphnia pulex
Pediculus humanus
Acyrthosiphon pisum
Tribolium castaneum
Nasonia vitripennis
Apis mellifera
Atta cephalotes
Bombyx mori
Danaus plexippus
Heliconius melpomene
Culex quinquefasciatus
Aedes aegypti
Anopheles gambiae
Anopheles darlingi
Drosophila virilis
Drosophila grimshawi
Drosophila willistoni
Drosophila pseudoobscura
Drosophila ananassae
Drosophila melanogaster
Drosophila yakuba
Strongylocentrotus purpuratus
Branchiostoma floridae
Ciona intestinalis
Ciona savignyi
Petromyzon marinus
Danio rerio
Gadus morhua
Oreochromis niloticus
Takifugu rubripes
Tetraodon nigroviridis
Gasterosteus aculeatus
Xiphophorus maculatus
Oryzias latipes
Latimeria chalumnae
Xenopus (Silurana) tropicalis
Pelodiscus sinensis
Anolis carolinensis
Taeniopygia guttata
Gallus gallus
Meleagris gallopavo
Ornithorhynchus anatinus
Monodelphis domestica
Sarcophilus harrisii
Macropus eugenii
Dasypus novemcinctus
Loxodonta africana
Echinops telfairi
Procavia capensis
Myotis lucifugus
Pteropus vampyrus
Sus scrofa
Tursiops truncatus
Bos taurus
Equus caballus
Felis catus
Canis lupus familiaris
Mustela putorius furo
Ailuropoda melanoleuca
Oryctolagus cuniculus
Cavia porcellus
Ictidomys tridecemlineatus
Dipodomys ordii
Rattus norvegicus
Mus musculus
Tupaia belangeri
Otolemur garnettii
Microcebus murinus
Tarsius syrichta
Callithrix jacchus
Macaca mulatta
Nomascus leucogenys
Pongo abelii
Gorilla gorilla
Pan troglodytes
Homo sapiens
Perkinsus marinus ATCC 50983
Tetrahymena thermophila
Paramecium tetraurelia strain d4 2
Neospora caninum Liverpool
Toxoplasma gondii
Cryptosporidium muris RN66
Cryptosporidium parvum Iowa II
Cryptosporidium hominis TU502
Theileria annulata strain Ankara
Theileria parva strain Muguga
Plasmodium falciparum
Plasmodium knowlesi
Plasmodium vivax
Plasmodium chabaudi
Plasmodium yoelii yoelii 17XNL
Plasmodium berghei
Phaeodactylum tricornutum
Thalassiosira pseudonana
Pythium ultimum
Albugo laibachii
Hyaloperonospora arabidopsidis
Phytophthora ramorum
Phytophthora infestans
Phytophthora sojae
Trypanosoma brucei
Trypanosoma cruzi strain CL Brener
Leishmania braziliensis MHOM BR 75 M2904
Leishmania mexicana MHOM GT 2001 U1103
Leishmania major
Leishmania donovani
Leishmania infantum JPCM5
Entamoeba histolytica
Entamoeba dispar SAW760
Dictyostelium discoideum
Dictyostelium purpureum
Trichomonas vaginalis G3
Trichomonas vaginalis
Naegleria gruberi
Giardia lamblia ATCC 50803
OG presence
OG absence
Inferred root
Dscore = 0.869
Pvalue = 9.18e−05
AdjPvalue = 1.09e−02

NOG18420

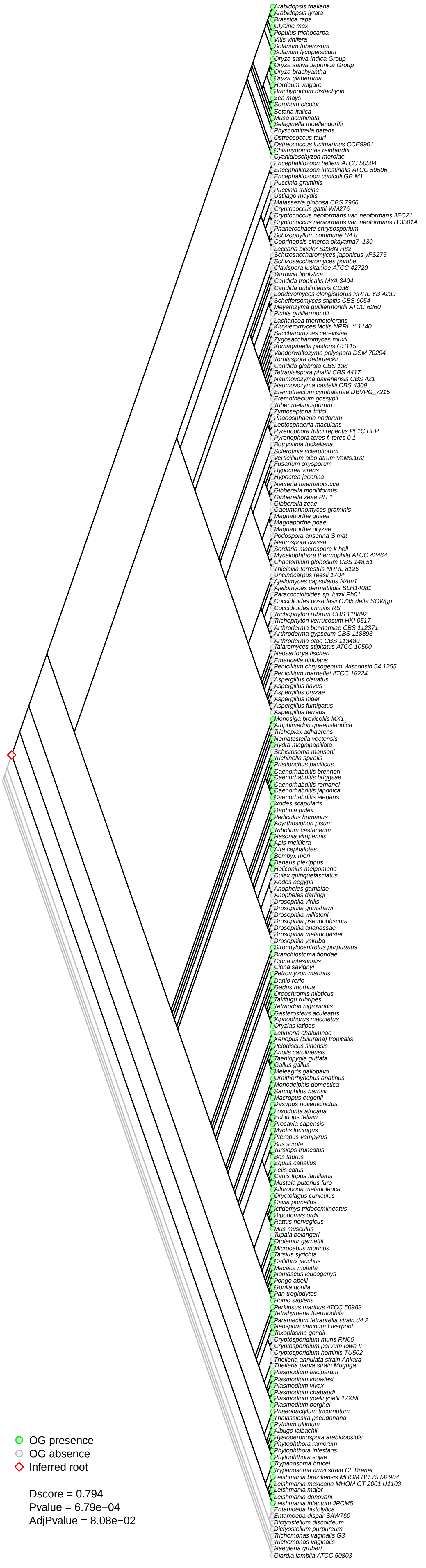
Arabidopsis thaliana
Arabidopsis lyrata
Brassica rapa
Glycine max
Populus trichocarpa
Vitis vinifera
Solanum tuberosum
Solanum lycopersicum
Oryza sativa Indica Group
Oryza sativa Japonica Group
Oryza brachyantha
Oryza glaberrima
Hordeum vulgare
Brachypodium distachyon
Zea mays
Sorghum bicolor
Setaria italica
Musa acuminata
Selaginella moellendorffii
Physcomitrella patens
Ostreococcus tauri
Ostreococcus lucimarinus CCE9901
Chlamydomonas reinhardtii
Cyanidioschyzon merolae
Encephalitozoon hellem ATCC 50504
Encephalitozoon intestinalis ATCC 50506
Encephalitozoon cuniculi GB M1
Puccinia graminis
Puccinia triticina
Ustilago maydis
Malassezia globosa CBS 7966
Cryptococcus gattii WM276
Cryptococcus neoformans var. neoformans JEC21
Cryptococcus neoformans var. neoformans B 3501A
Phanerochaete chrysosporium
Schizophyllum commune H4 8
Coprinopsis cinerea okayama7_130
Laccaria bicolor S238N H82
Schizosaccharomyces japonicus yFS275
Schizosaccharomyces pombe
Clavispora lusitaniae ATCC 42720
Yarrowia lipolytica
Candida tropicalis MYA 3404
Candida dubliniensis CD36
Lodderomyces elongisporus NRRL YB 4239
Scheffersomyces stipitis CBS 6054
Meyerozyma guilliermondii ATCC 6260
Pichia guilliermondii
Lachancea thermotolerans
Kluyveromyces lactis NRRL Y 1140
Saccharomyces cerevisiae
Zygosaccharomyces rouxii
Komagataella pastoris GS115
Vanderwaltozyma polyspora DSM 70294
Torulaspora delbrueckii
Candida glabrata CBS 138
Tetrapisispora phaffii CBS 4417
Naumovozyma dairenensis CBS 421
Naumovozyma castellii CBS 4309
Eremothecium cymbalariae DBVPG_7215
Eremothecium gossypii
Tuber melanosporum
Zymoseptoria tritici
Phaeosphaeria nodorum
Leptosphaeria maculans
Pyrenophora tritici repentis Pt 1C BFP
Pyrenophora teres f. teres 0 1
Botryotinia fuckeliana
Sclerotinia sclerotiorum
Verticillium albo atrum VaMs.102
Fusarium oxysporum
Hypocrea virens
Hypocrea jecorina
Nectria haematococca
Gibberella moniliformis
Gibberella zeae PH 1
Gibberella zeae
Gaeumannomyces graminis
Magnaporthe grisea
Magnaporthe poae
Magnaporthe oryzae
Podospora anserina S mat
Neurospora crassa
Sordaria macrospora k hell
Myceliophthora thermophila ATCC 42464
Chaetomium globosum CBS 148.51
Thielavia terrestris NRRL 8126
Uncinocarpus reesii 1704
Ajellomyces capsulatus NAm1
Ajellomyces dermatitidis SLH14081
Paracoccidioides sp. lutzii Pb01
Coccidioides posadasii C735 delta SOWgp
Coccidioides immitis RS
Trichophyton rubrum CBS 118892
Trichophyton verrucosum HKI 0517
Arthroderma benhamiae CBS 112371
Arthroderma gypseum CBS 118893
Arthroderma otae CBS 113480
Talaromyces stipitatus ATCC 10500
Neosartorya fischeri
Emericella nidulans
Penicillium chrysogenum Wisconsin 54 1255
Penicillium marneffei ATCC 18224
Aspergillus clavatus
Aspergillus flavus
Aspergillus oryzae
Aspergillus niger
Aspergillus fumigatus
Aspergillus terreus
Monosiga brevicollis MX1
Amphimedon queenslandica
Trichoplax adhaerens
Nematostella vectensis
Hydra magnipapillata
Schistosoma mansoni
Trichinella spiralis
Pristionchus pacificus
Caenorhabditis brenneri
Caenorhabditis briggsae
Caenorhabditis remanei
Caenorhabditis japonica
Caenorhabditis elegans
Ixodes scapularis
Daphnia pulex
Pediculus humanus
Acyrthosiphon pisum
Tribolium castaneum
Nasonia vitripennis
Apis mellifera
Atta cephalotes
Bombyx mori
Danaus plexippus
Heliconius melpomene
Culex quinquefasciatus
Aedes aegypti
Anopheles gambiae
Anopheles darlingi
Drosophila virilis
Drosophila grimshawi
Drosophila willistoni
Drosophila pseudoobscura
Drosophila ananassae
Drosophila melanogaster
Drosophila yakuba
Strongylocentrotus purpuratus
Branchiostoma floridae
Ciona intestinalis
Ciona savignyi
Petromyzon marinus
Danio rerio
Gadus morhua
Oreochromis niloticus
Takifugu rubripes
Tetraodon nigroviridis
Gasterosteus aculeatus
Xiphophorus maculatus
Oryzias latipes
Latimeria chalumnae
Xenopus (Silurana) tropicalis
Pelodiscus sinensis
Anolis carolinensis
Taeniopygia guttata
Gallus gallus
Meleagris gallopavo
Ornithorhynchus anatinus
Monodelphis domestica
Sarcophilus harrisii
Macropus eugenii
Dasypus novemcinctus
Loxodonta africana
Echinops telfairi
Procavia capensis
Myotis lucifugus
Pteropus vampyrus
Sus scrofa
Tursiops truncatus
Bos taurus
Equus caballus
Felis catus
Canis lupus familiaris
Mustela putorius furo
Ailuropoda melanoleuca
Oryctolagus cuniculus
Cavia porcellus
Ictidomys tridecemlineatus
Dipodomys ordii
Rattus norvegicus
Mus musculus
Tupaia belangeri
Otolemur garnettii
Microcebus murinus
Tarsius syrichta
Callithrix jacchus
Macaca mulatta
Nomascus leucogenys
Pongo abelii
Gorilla gorilla
Pan troglodytes
Homo sapiens
Perkinsus marinus ATCC 50983
Tetrahymena thermophila
Paramecium tetraurelia strain d4 2
Neospora caninum Liverpool
Toxoplasma gondii
Cryptosporidium muris RN66
Cryptosporidium parvum Iowa II
Cryptosporidium hominis TU502
Theileria annulata strain Ankara
Theileria parva strain Muguga
Plasmodium falciparum
Plasmodium knowlesi
Plasmodium vivax
Plasmodium chabaudi
Plasmodium yoelii yoelii 17XNL
Plasmodium berghei
Phaeodactylum tricornutum
Thalassiosira pseudonana
Pythium ultimum
Albugo laibachii
Hyaloperonospora arabidopsidis
Phytophthora ramorum
Phytophthora infestans
Phytophthora sojae
Trypanosoma brucei
Trypanosoma cruzi strain CL Brener
Leishmania braziliensis MHOM BR 75 M2904
Leishmania mexicana MHOM GT 2001 U1103
Leishmania major
Leishmania donovani
Leishmania infantum JPCM5
Entamoeba histolytica
Entamoeba dispar SAW760
Dictyostelium discoideum
Dictyostelium purpureum
Trichomonas vaginalis G3
Trichomonas vaginalis
Naegleria gruberi
Giardia lamblia ATCC 50803
OG presence
OG absence
Inferred root
Dscore = 0.794
Pvalue = 6.79e−04
AdjPvalue = 8.08e−02

NOG185758

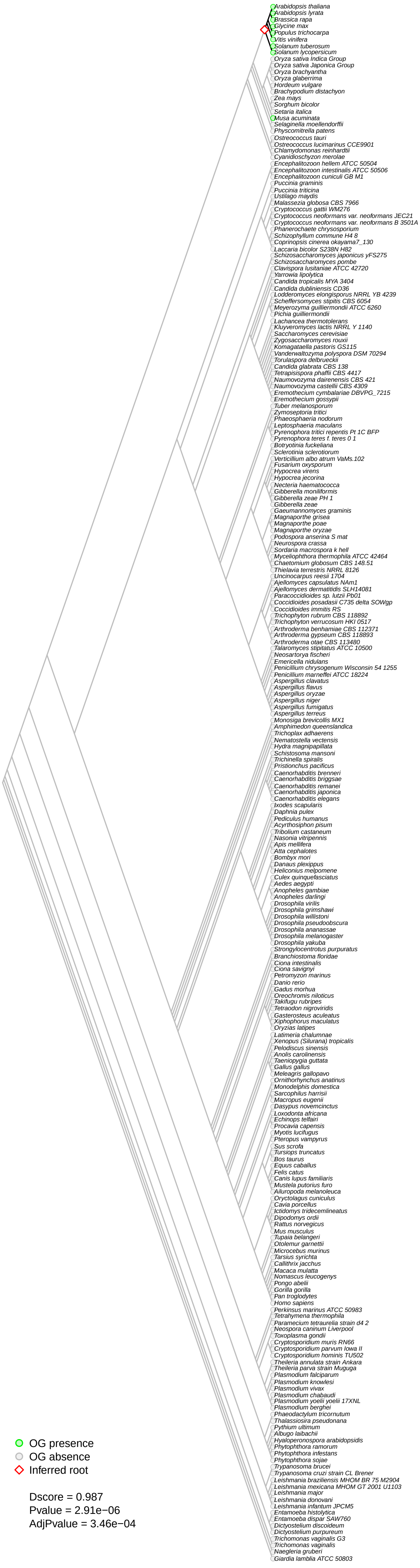

Arabidopsis thaliana
Arabidopsis lyrata
Brassica rapa
Glycine max
Populus trichocarpa
Vitis vinifera
Solanum tuberosum
Solanum lycopersicum
Oryza sativa Indica Group
Oryza sativa Japonica Group
Oryza brachyantha
Oryza glaberrima
Hordeum vulgare
Brachypodium distachyon
Zea mays
Sorghum bicolor
Setaria italica
Musa acuminata
Selaginella moellendorffii
Physcomitrella patens
Ostreococcus tauri
Ostreococcus lucimarinus CCE9901
Chlamydomonas reinhardtii
Cyanidioschyzon merolae
Encephalitozoon hellem ATCC 50504
Encephalitozoon intestinalis ATCC 50506
Encephalitozoon cuniculi GB M1
Puccinia graminis
Puccinia triticina
Ustilago maydis
Malassezia globosa CBS 7966
Cryptococcus gattii WM276
Cryptococcus neoformans var. neoformans JEC21
Cryptococcus neoformans var. neoformans B 3501A
Phanerochaete chrysosporium
Schizophyllum commune H4 8
Coprinopsis cinerea okayama7_130
Laccaria bicolor S238N H82
Schizosaccharomyces japonicus yFS275
Schizosaccharomyces pombe
Clavispora lusitaniae ATCC 42720
Yarrowia lipolytica
Candida tropicalis MYA 3404
Candida dubliniensis CD36
Lodderomyces elongisporus NRRL YB 4239
Scheffersomyces stipitis CBS 6054
Meyerozyma guilliermondii ATCC 6260
Pichia guilliermondii
Lachancea thermotolerans
Kluyveromyces lactis NRRL Y 1140
Saccharomyces cerevisiae
Zygosaccharomyces rouxii
Komagataella pastoris GS115
Vanderwaltozyma polyspora DSM 70294
Torulaspora delbrueckii
Candida glabrata CBS 138
Tetrapisispora phaffii CBS 4417
Naumovozyma dairenensis CBS 421
Naumovozyma castellii CBS 4309
Eremothecium cymbalariae DBVPG_7215
Eremothecium gossypii
Tuber melanosporum
Zymoseptoria tritici
Phaeosphaeria nodorum
Leptosphaeria maculans
Pyrenophora tritici repentis Pt 1C BFP
Pyrenophora teres f. teres 0 1
Botryotinia fuckeliana
Sclerotinia sclerotiorum
Verticillium albo atrum VaMs.102
Fusarium oxysporum
Hypocrea virens
Hypocrea jecorina
Nectria haematococca
Gibberella moniliformis
Gibberella zeae PH 1
Gibberella zeae
Gaeumannomyces graminis
Magnaporthe grisea
Magnaporthe poae
Magnaporthe oryzae
Podospora anserina S mat
Neurospora crassa
Sordaria macrospora k hell
Myceliophthora thermophila ATCC 42464
Chaetomium globosum CBS 148.51
Thielavia terrestris NRRL 8126
Uncinocarpus reesii 1704
Ajellomyces capsulatus NAm1
Ajellomyces dermatitidis SLH14081
Paracoccidioides sp. lutzii Pb01
Coccidioides posadasii C735 delta SOWgp
Coccidioides immitis RS
Trichophyton rubrum CBS 118892
Trichophyton verrucosum HKI 0517
Arthroderma benhamiae CBS 112371
Arthroderma gypseum CBS 118893
Arthroderma otae CBS 113480
Talaromyces stipitatus ATCC 10500
Neosartorya fischeri
Emericella nidulans
Penicillium chrysogenum Wisconsin 54 1255
Penicillium marneffei ATCC 18224
Aspergillus clavatus
Aspergillus flavus
Aspergillus oryzae
Aspergillus niger
Aspergillus fumigatus
Aspergillus terreus
Monosiga brevicollis MX1
Amphimedon queenslandica
Trichoplax adhaerens
Nematostella vectensis
Hydra magnipapillata
Schistosoma mansoni
Trichinella spiralis
Pristionchus pacificus
Caenorhabditis brenneri
Caenorhabditis briggsae
Caenorhabditis remanei
Caenorhabditis japonica
Caenorhabditis elegans
Ixodes scapularis
Daphnia pulex
Pediculus humanus
Acyrthosiphon pisum
Tribolium castaneum
Nasonia vitripennis
Apis mellifera
Atta cephalotes
Bombyx mori
Danaus plexippus
Heliconius melpomene
Culex quinquefasciatus
Aedes aegypti
Anopheles gambiae
Anopheles darlingi
Drosophila virilis
Drosophila grimshawi
Drosophila willistoni
Drosophila pseudoobscura
Drosophila ananassae
Drosophila melanogaster
Drosophila yakuba
Strongylocentrotus purpuratus
Branchiostoma floridae
Ciona intestinalis
Ciona savignyi
Petromyzon marinus
Danio rerio
Gadus morhua
Oreochromis niloticus
Takifugu rubripes
Tetraodon nigroviridis
Gasterosteus aculeatus
Xiphophorus maculatus
Oryzias latipes
Latimeria chalumnae
Xenopus (Silurana) tropicalis
Pelodiscus sinensis
Anolis carolinensis
Taeniopygia guttata
Gallus gallus
Meleagris gallopavo
Ornithorhynchus anatinus
Monodelphis domestica
Sarcophilus harrisii
Macropus eugenii
Dasypus novemcinctus
Loxodonta africana
Echinops telfairi
Procavia capensis
Myotis lucifugus
Pteropus vampyrus
Sus scrofa
Tursiops truncatus
Bos taurus
Equus caballus
Felis catus
Canis lupus familiaris
Mustela putorius furo
Ailuropoda melanoleuca
Oryctolagus cuniculus
Cavia porcellus
Ictidomys tridecemlineatus
Dipodomys ordii
Rattus norvegicus
Mus musculus
Tupaia belangeri
Otolemur garnettii
Microcebus murinus
Tarsius syrichta
Callithrix jacchus
Macaca mulatta
Nomascus leucogenys
Pongo abelii
Gorilla gorilla
Pan troglodytes
Homo sapiens
Perkinsus marinus ATCC 50983
Tetrahymena thermophila
Paramecium tetraurelia strain d4 2
Neospora caninum Liverpool
Toxoplasma gondii
Cryptosporidium muris RN66
Cryptosporidium parvum Iowa II
Cryptosporidium hominis TU502
Theileria annulata strain Ankara
Theileria parva strain Muguga
Plasmodium falciparum
Plasmodium knowlesi
Plasmodium vivax
Plasmodium chabaudi
Plasmodium yoelii yoelii 17XNL
Plasmodium berghei
Phaeodactylum tricornutum
Thalassiosira pseudonana
Pythium ultimum
Albugo laibachii
Hyaloperonospora arabidopsidis
Phytophthora ramorum
Phytophthora infestans
Phytophthora sojae
Trypanosoma brucei
Trypanosoma cruzi strain CL Brener
Leishmania braziliensis MHOM BR 75 M2904
Leishmania mexicana MHOM GT 2001 U1103
Leishmania major
Leishmania donovani
Leishmania infantum JPCM5
Entamoeba histolytica
Entamoeba dispar SAW760
Dictyostelium discoideum
Dictyostelium purpureum
Trichomonas vaginalis G3
Trichomonas vaginalis
Naegleria gruberi
Giardia lamblia ATCC 50803
OG presence
OG absence
Inferred root
Dscore = 0.987
Pvalue = 2.91e−06
AdjPvalue = 3.46e−04

NOG191381

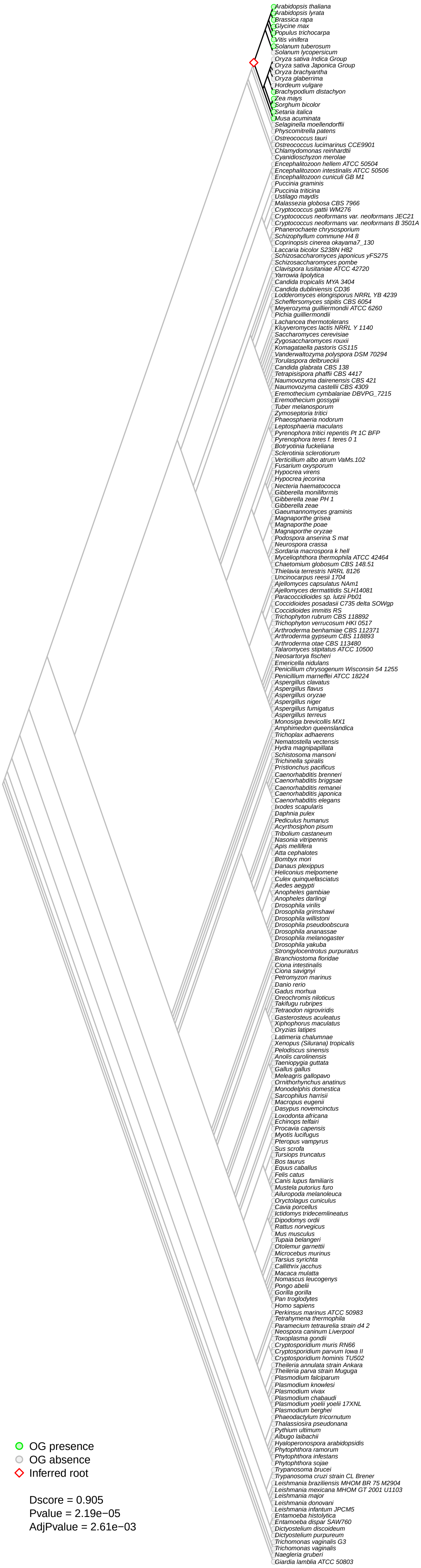

Dscore = 0.905
Pvalue = 2.19e−05
AdjPvalue = 2.61e−03

NOG19163

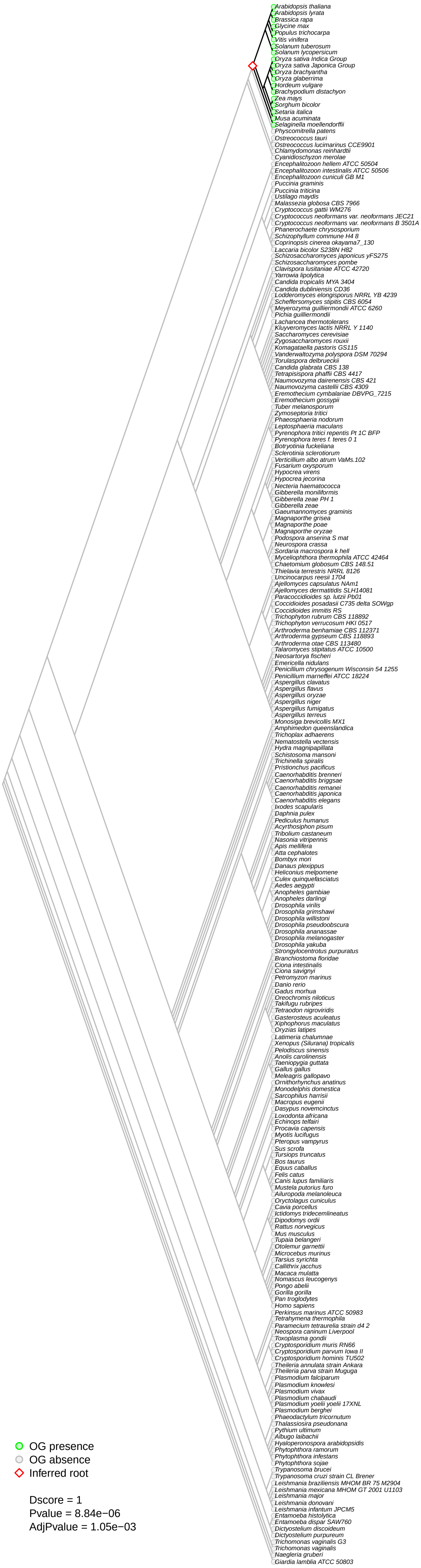
Arabidopsis thaliana
Arabidopsis lyrata
Brassica rapa
Glycine max
Populus trichocarpa
Vitis vinifera
Solanum tuberosum
Solanum lycopersicum
Oryza sativa Indica Group
Oryza sativa Japonica Group
Oryza brachyantha
Oryza glaberrima
Hordeum vulgare
Brachypodium distachyon
Zea mays
Sorghum bicolor
Setaria italica
Musa acuminata
Selaginella moellendorffii
Physcomitrella patens
Ostreococcus tauri
Ostreococcus lucimarinus CCE9901
Chlamydomonas reinhardtii
Cyanidioschyzon merolae
Encephalitozoon hellem ATCC 50504
Encephalitozoon intestinalis ATCC 50506
Encephalitozoon cuniculi GB M1
Puccinia graminis
Puccinia triticina
Ustilago maydis
Malassezia globosa CBS 7966
Cryptococcus gattii WM276
Cryptococcus neoformans var. neoformans JEC21
Cryptococcus neoformans var. neoformans B 3501A
Phanerochaete chrysosporium
Schizophyllum commune H4 8
Coprinopsis cinerea okayama7_130
Laccaria bicolor S238N H82
Schizosaccharomyces japonicus yFS275
Schizosaccharomyces pombe
Clavispora lusitaniae ATCC 42720
Yarrowia lipolytica
Candida tropicalis MYA 3404
Candida dubliniensis CD36
Lodderomyces elongisporus NRRL YB 4239
Scheffersomyces stipitis CBS 6054
Meyerozyma guilliermondii ATCC 6260
Pichia guilliermondii
Lachancea thermotolerans
Kluyveromyces lactis NRRL Y 1140
Saccharomyces cerevisiae
Zygosaccharomyces rouxii
Komagataella pastoris GS115
Vanderwaltozyma polyspora DSM 70294
Torulaspora delbrueckii
Candida glabrata CBS 138
Tetrapisispora phaffii CBS 4417
Naumovozyma dairenensis CBS 421
Naumovozyma castellii CBS 4309
Eremothecium cymbalariae DBVPG_7215
Eremothecium gossypii
Tuber melanosporum
Zymoseptoria tritici
Phaeosphaeria nodorum
Leptosphaeria maculans
Pyrenophora tritici repentis Pt 1C BFP
Pyrenophora teres f. teres 0 1
Botryotinia fuckeliana
Sclerotinia sclerotiorum
Verticillium albo atrum VaMs.102
Fusarium oxysporum
Hypocrea virens
Hypocrea jecorina
Nectria haematococca
Gibberella moniliformis
Gibberella zeae PH 1
Gibberella zeae
Gaeumannomyces graminis
Magnaporthe grisea
Magnaporthe poae
Magnaporthe oryzae
Podospora anserina S mat
Neurospora crassa
Sordaria macrospora k hell
Myceliophthora thermophila ATCC 42464
Chaetomium globosum CBS 148.51
Thielavia terrestris NRRL 8126
Uncinocarpus reesii 1704
Ajellomyces capsulatus NAm1
Ajellomyces dermatitidis SLH14081
Paracoccidioides sp. lutzii Pb01
Coccidioides posadasii C735 delta SOWgp
Coccidioides immitis RS
Trichophyton rubrum CBS 118892
Trichophyton verrucosum HKI 0517
Arthroderma benhamiae CBS 112371
Arthroderma gypseum CBS 118893
Arthroderma otae CBS 113480
Talaromyces stipitatus ATCC 10500
Neosartorya fischeri
Emericella nidulans
Penicillium chrysogenum Wisconsin 54 1255
Penicillium marneffei ATCC 18224
Aspergillus clavatus
Aspergillus flavus
Aspergillus oryzae
Aspergillus niger
Aspergillus fumigatus
Aspergillus terreus
Monosiga brevicollis MX1
Amphimedon queenslandica
Trichoplax adhaerens
Nematostella vectensis
Hydra magnipapillata
Schistosoma mansoni
Trichinella spiralis
Pristionchus pacificus
Caenorhabditis brenneri
Caenorhabditis briggsae
Caenorhabditis remanei
Caenorhabditis japonica
Caenorhabditis elegans
Ixodes scapularis
Daphnia pulex
Pediculus humanus
Acyrthosiphon pisum
Tribolium castaneum
Nasonia vitripennis
Apis mellifera
Atta cephalotes
Bombyx mori
Danaus plexippus
Heliconius melpomene
Culex quinquefasciatus
Aedes aegypti
Anopheles gambiae
Anopheles darlingi
Drosophila virilis
Drosophila grimshawi
Drosophila willistoni
Drosophila pseudoobscura
Drosophila ananassae
Drosophila melanogaster
Drosophila yakuba
Strongylocentrotus purpuratus
Branchiostoma floridae
Ciona intestinalis
Ciona savignyi
Petromyzon marinus
Danio rerio
Gadus morhua
Oreochromis niloticus
Takifugu rubripes
Tetraodon nigroviridis
Gasterosteus aculeatus
Xiphophorus maculatus
Oryzias latipes
Latimeria chalumnae
Xenopus (Silurana) tropicalis
Pelodiscus sinensis
Anolis carolinensis
Taeniopygia guttata
Gallus gallus
Meleagris gallopavo
Ornithorhynchus anatinus
Monodelphis domestica
Sarcophilus harrisii
Macropus eugenii
Dasypus novemcinctus
Loxodonta africana
Echinops telfairi
Procavia capensis
Myotis lucifugus
Pteropus vampyrus
Sus scrofa
Tursiops truncatus
Bos taurus
Equus caballus
Felis catus
Canis lupus familiaris
Mustela putorius furo
Ailuropoda melanoleuca
Oryctolagus cuniculus
Cavia porcellus
Ictidomys tridecemlineatus
Dipodomys ordii
Rattus norvegicus
Mus musculus
Tupaia belangeri
Otolemur garnettii
Microcebus murinus
Tarsius syrichta
Callithrix jacchus
Macaca mulatta
Nomascus leucogenys
Pongo abelii
Gorilla gorilla
Pan troglodytes
Homo sapiens
Perkinsus marinus ATCC 50983
Tetrahymena thermophila
Paramecium tetraurelia strain d4 2
Neospora caninum Liverpool
Toxoplasma gondii
Cryptosporidium muris RN66
Cryptosporidium parvum Iowa II
Cryptosporidium hominis TU502
Theileria annulata strain Ankara
Theileria parva strain Muguga
Plasmodium falciparum
Plasmodium knowlesi
Plasmodium vivax
Plasmodium chabaudi
Plasmodium yoelii yoelii 17XNL
Plasmodium berghei
Phaeodactylum tricornutum
Thalassiosira pseudonana
Pythium ultimum
Albugo laibachii
Hyaloperonospora arabidopsidis
Phytophthora ramorum
Phytophthora infestans
Phytophthora sojae
Trypanosoma brucei
Trypanosoma cruzi strain CL Brener
Leishmania braziliensis MHOM BR 75 M2904
Leishmania mexicana MHOM GT 2001 U1103
Leishmania major
Leishmania donovani
Leishmania infantum JPCM5
Entamoeba histolytica
Entamoeba dispar SAW760
Dictyostelium discoideum
Dictyostelium purpureum
Trichomonas vaginalis G3
Trichomonas vaginalis
Naegleria gruberi
Giardia lamblia ATCC 50803
OG presence
OG absence
Inferred root
Dscore = 1
Pvalue = 8.84e−06
AdjPvalue = 1.05e−03

NOG19390

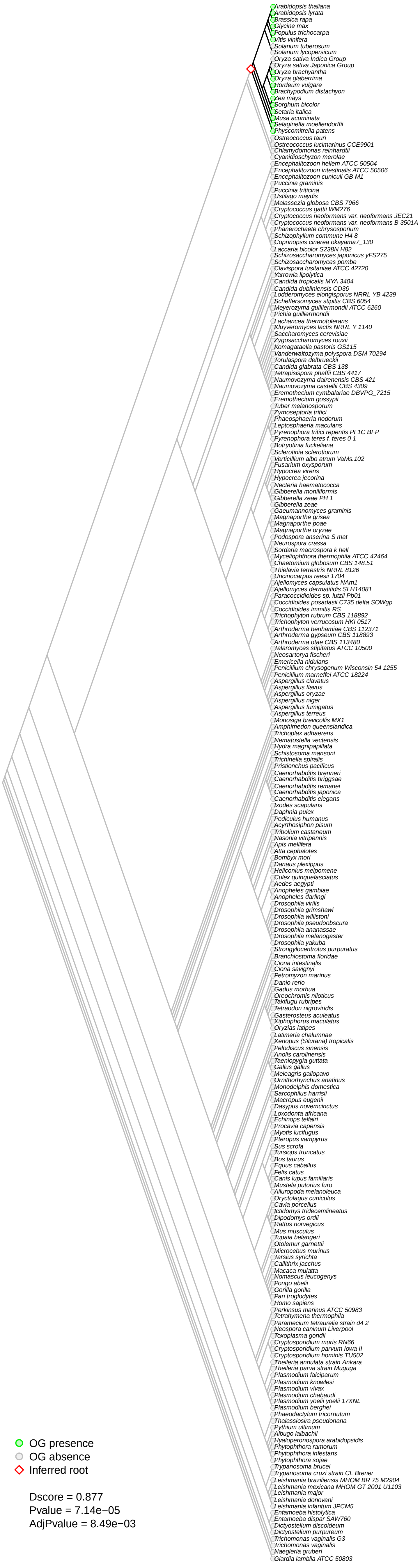

Arabidopsis thaliana
Arabidopsis lyrata
Brassica rapa
Glycine max
Populus trichocarpa
Vitis vinifera
Solanum tuberosum
Solanum lycopersicum
Oryza sativa Indica Group
Oryza sativa Japonica Group
Oryza brachyantha
Oryza glaberrima
Hordeum vulgare
Brachypodium distachyon
Zea mays
Sorghum bicolor
Setaria italica
Musa acuminata
Selaginella moellendorffii
Physcomitrella patens
Ostreococcus tauri
Ostreococcus lucimarinus CCE9901
Chlamydomonas reinhardtii
Cyanidioschyzon merolae
Encephalitozoon hellem ATCC 50504
Encephalitozoon intestinalis ATCC 50506
Encephalitozoon cuniculi GB M1
Puccinia graminis
Puccinia triticina
Ustilago maydis
Malassezia globosa CBS 7966
Cryptococcus gattii WM276
Cryptococcus neoformans var. neoformans JEC21
Cryptococcus neoformans var. neoformans B 3501A
Phanerochaete chrysosporium
Schizophyllum commune H4 8
Coprinopsis cinerea okayama7_130
Laccaria bicolor S238N H82
Schizosaccharomyces japonicus yFS275
Schizosaccharomyces pombe
Clavispora lusitaniae ATCC 42720
Yarrowia lipolytica
Candida tropicalis MYA 3404
Candida dubliniensis CD36
Lodderomyces elongisporus NRRL YB 4239
Scheffersomyces stipitis CBS 6054
Meyerozyma guilliermondii ATCC 6260
Pichia guilliermondii
Lachancea thermotolerans
Kluyveromyces lactis NRRL Y 1140
Saccharomyces cerevisiae
Zygosaccharomyces rouxii
Komagataella pastoris GS115
Vanderwaltozyma polyspora DSM 70294
Torulaspora delbrueckii
Candida glabrata CBS 138
Tetrapisispora phaffii CBS 4417
Naumovozyma dairenensis CBS 421
Naumovozyma castellii CBS 4309
Eremothecium cymbalariae DBVPG_7215
Eremothecium gossypii
Tuber melanosporum
Zymoseptoria tritici
Phaeosphaeria nodorum
Leptosphaeria maculans
Pyrenophora tritici repentis Pt 1C BFP
Pyrenophora teres f. teres 0 1
Botryotinia fuckeliana
Sclerotinia sclerotiorum
Verticillium albo atrum VaMs.102
Fusarium oxysporum
Hypocrea virens
Hypocrea jecorina
Nectria haematococca
Gibberella moniliformis
Gibberella zeae PH 1
Gibberella zeae
Gaeumannomyces graminis
Magnaporthe grisea
Magnaporthe poae
Magnaporthe oryzae
Podospora anserina S mat
Neurospora crassa
Sordaria macrospora k hell
Myceliophthora thermophila ATCC 42464
Chaetomium globosum CBS 148.51
Thielavia terrestris NRRL 8126
Uncinocarpus reesii 1704
Ajellomyces capsulatus NAm1
Ajellomyces dermatitidis SLH14081
Paracoccidioides sp. lutzii Pb01
Coccidioides posadasii C735 delta SOWgp
Coccidioides immitis RS
Trichophyton rubrum CBS 118892
Trichophyton verrucosum HKI 0517
Arthroderma benhamiae CBS 112371
Arthroderma gypseum CBS 118893
Arthroderma otae CBS 113480
Talaromyces stipitatus ATCC 10500
Neosartorya fischeri
Emericella nidulans
Penicillium chrysogenum Wisconsin 54 1255
Penicillium marneffei ATCC 18224
Aspergillus clavatus
Aspergillus flavus
Aspergillus oryzae
Aspergillus niger
Aspergillus fumigatus
Aspergillus terreus
Monosiga brevicollis MX1
Amphimedon queenslandica
Trichoplax adhaerens
Nematostella vectensis
Hydra magnipapillata
Schistosoma mansoni
Trichinella spiralis
Pristionchus pacificus
Caenorhabditis brenneri
Caenorhabditis briggsae
Caenorhabditis remanei
Caenorhabditis japonica
Caenorhabditis elegans
Ixodes scapularis
Daphnia pulex
Pediculus humanus
Acyrthosiphon pisum
Tribolium castaneum
Nasonia vitripennis
Apis mellifera
Atta cephalotes
Bombyx mori
Danaus plexippus
Heliconius melpomene
Culex quinquefasciatus
Aedes aegypti
Anopheles gambiae
Anopheles darlingi
Drosophila virilis
Drosophila grimshawi
Drosophila willistoni
Drosophila pseudoobscura
Drosophila ananassae
Drosophila melanogaster
Drosophila yakuba
Strongylocentrotus purpuratus
Branchiostoma floridae
Ciona intestinalis
Ciona savignyi
Petromyzon marinus
Danio rerio
Gadus morhua
Oreochromis niloticus
Takifugu rubripes
Tetraodon nigroviridis
Gasterosteus aculeatus
Xiphophorus maculatus
Oryzias latipes
Latimeria chalumnae
Xenopus (Silurana) tropicalis
Pelodiscus sinensis
Anolis carolinensis
Taeniopygia guttata
Gallus gallus
Meleagris gallopavo
Ornithorhynchus anatinus
Monodelphis domestica
Sarcophilus harrisii
Macropus eugenii
Dasypus novemcinctus
Loxodonta africana
Echinops telfairi
Procavia capensis
Myotis lucifugus
Pteropus vampyrus
Sus scrofa
Tursiops truncatus
Bos taurus
Equus caballus
Felis catus
Canis lupus familiaris
Mustela putorius furo
Ailuropoda melanoleuca
Oryctolagus cuniculus
Cavia porcellus
Ictidomys tridecemlineatus
Dipodomys ordii
Rattus norvegicus
Mus musculus
Tupaia belangeri
Otolemur garnettii
Microcebus murinus
Tarsius syrichta
Callithrix jacchus
Macaca mulatta
Nomascus leucogenys
Pongo abelii
Gorilla gorilla
Pan troglodytes
Homo sapiens
Perkinsus marinus ATCC 50983
Tetrahymena thermophila
Paramecium tetraurelia strain d4 2
Neospora caninum Liverpool
Toxoplasma gondii
Cryptosporidium muris RN66
Cryptosporidium parvum Iowa II
Cryptosporidium hominis TU502
Theileria annulata strain Ankara
Theileria parva strain Muguga
Plasmodium falciparum
Plasmodium knowlesi
Plasmodium vivax
Plasmodium chabaudi
Plasmodium yoelii yoelii 17XNL
Plasmodium berghei
Phaeodactylum tricornutum
Thalassiosira pseudonana
Pythium ultimum
Albugo laibachii
Hyaloperonospora arabidopsidis
Phytophthora ramorum
Phytophthora infestans
Phytophthora sojae
Trypanosoma brucei
Trypanosoma cruzi strain CL Brener
Leishmania braziliensis MHOM BR 75 M2904
Leishmania mexicana MHOM GT 2001 U1103
Leishmania major
Leishmania donovani
Leishmania infantum JPCM5
Entamoeba histolytica
Entamoeba dispar SAW760
Dictyostelium discoideum
Dictyostelium purpureum
Trichomonas vaginalis G3
Trichomonas vaginalis
Naegleria gruberi
Giardia lamblia ATCC 50803
OG presence
OG absence
Inferred root
Dscore = 0.877
Pvalue = 7.14e−05
AdjPvalue = 8.49e−03

NOG20146

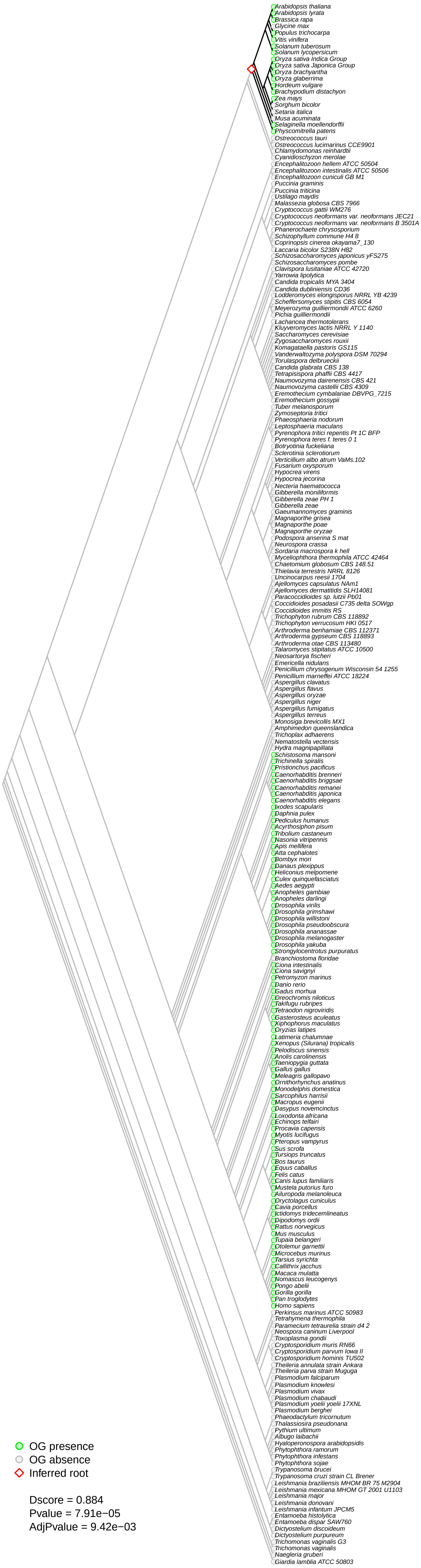

Arabidopsis thaliana
Arabidopsis lyrata
Brassica rapa
Glycine max
Populus trichocarpa
Vitis vinifera
Solanum tuberosum
Solanum lycopersicum
Oryza sativa Indica Group
Oryza sativa Japonica Group
Oryza brachyantha
Oryza glaberrima
Hordeum vulgare
Brachypodium distachyon
Zea mays
Sorghum bicolor
Setaria italica
Musa acuminata
Selaginella moellendorffii
Physcomitrella patens
Ostreococcus tauri
Ostreococcus lucimarinus CCE9901
Chlamydomonas reinhardtii
Cyanidioschyzon merolae
Encephalitozoon hellem ATCC 50504
Encephalitozoon intestinalis ATCC 50506
Encephalitozoon cuniculi GB M1
Puccinia graminis
Puccinia triticina
Ustilago maydis
Malassezia globosa CBS 7966
Cryptococcus gattii WM276
Cryptococcus neoformans var. neoformans JEC21
Cryptococcus neoformans var. neoformans B 3501A
Phanerochaete chrysosporium
Schizophyllum commune H4 8
Coprinopsis cinerea okayama7_130
Laccaria bicolor S238N H82
Schizosaccharomyces japonicus yFS275
Schizosaccharomyces pombe
Clavispora lusitaniae ATCC 42720
Yarrowia lipolytica
Candida tropicalis MYA 3404
Candida dubliniensis CD36
Lodderomyces elongisporus NRRL YB 4239
Scheffersomyces stipitis CBS 6054
Meyerozyma guilliermondii ATCC 6260
Pichia guilliermondii
Lachancea thermotolerans
Kluyveromyces lactis NRRL Y 1140
Saccharomyces cerevisiae
Zygosaccharomyces rouxii
Komagataella pastoris GS115
Vanderwaltozyma polyspora DSM 70294
Torulaspora delbrueckii
Candida glabrata CBS 138
Tetrapisispora phaffii CBS 4417
Naumovozyma dairenensis CBS 421
Naumovozyma castellii CBS 4309
Eremothecium cymbalariae DBVPG_7215
Eremothecium gossypii
Tuber melanosporum
Zymoseptoria tritici
Phaeosphaeria nodorum
Leptosphaeria maculans
Pyrenophora tritici repentis Pt 1C BFP
Pyrenophora teres f. teres 0 1
Botryotinia fuckeliana
Sclerotinia sclerotiorum
Verticillium albo atrum VaMs.102
Fusarium oxysporum
Hypocrea virens
Hypocrea jecorina
Nectria haematococca
Gibberella moniliformis
Gibberella zeae PH 1
Gibberella zeae
Gaeumannomyces graminis
Magnaporthe grisea
Magnaporthe poae
Magnaporthe oryzae
Podospora anserina S mat
Neurospora crassa
Sordaria macrospora k hell
Myceliophthora thermophila ATCC 42464
Chaetomium globosum CBS 148.51
Thielavia terrestris NRRL 8126
Uncinocarpus reesii 1704
Ajellomyces capsulatus NAm1
Ajellomyces dermatitidis SLH14081
Paracoccidioides sp. lutzii Pb01
Coccidioides posadasii C735 delta SOWgp
Coccidioides immitis RS
Trichophyton rubrum CBS 118892
Trichophyton verrucosum HKI 0517
Arthroderma benhamiae CBS 112371
Arthroderma gypseum CBS 118893
Arthroderma otae CBS 113480
Talaromyces stipitatus ATCC 10500
Neosartorya fischeri
Emericella nidulans
Penicillium chrysogenum Wisconsin 54 1255
Penicillium marneffei ATCC 18224
Aspergillus clavatus
Aspergillus flavus
Aspergillus oryzae
Aspergillus niger
Aspergillus fumigatus
Aspergillus terreus
Monosiga brevicollis MX1
Amphimedon queenslandica
Trichoplax adhaerens
Nematostella vectensis
Hydra magnipapillata
Schistosoma mansoni
Trichinella spiralis
Pristionchus pacificus
Caenorhabditis brenneri
Caenorhabditis briggsae
Caenorhabditis remanei
Caenorhabditis japonica
Caenorhabditis elegans
Ixodes scapularis
Daphnia pulex
Pediculus humanus
Acyrthosiphon pisum
Tribolium castaneum
Nasonia vitripennis
Apis mellifera
Atta cephalotes
Bombyx mori
Danaus plexippus
Heliconius melpomene
Culex quinquefasciatus
Aedes aegypti
Anopheles gambiae
Anopheles darlingi
Drosophila virilis
Drosophila grimshawi
Drosophila willistoni
Drosophila pseudoobscura
Drosophila ananassae
Drosophila melanogaster
Drosophila yakuba
Strongylocentrotus purpuratus
Branchiostoma floridae
Ciona intestinalis
Ciona savignyi
Petromyzon marinus
Danio rerio
Gadus morhua
Oreochromis niloticus
Takifugu rubripes
Tetraodon nigroviridis
Gasterosteus aculeatus
Xiphophorus maculatus
Oryzias latipes
Latimeria chalumnae
Xenopus (Silurana) tropicalis
Pelodiscus sinensis
Anolis carolinensis
Taeniopygia guttata
Gallus gallus
Meleagris gallopavo
Ornithorhynchus anatinus
Monodelphis domestica
Sarcophilus harrisii
Macropus eugenii
Dasypus novemcinctus
Loxodonta africana
Echinops telfairi
Procavia capensis
Myotis lucifugus
Pteropus vampyrus
Sus scrofa
Tursiops truncatus
Bos taurus
Equus caballus
Felis catus
Canis lupus familiaris
Mustela putorius furo
Ailuropoda melanoleuca
Oryctolagus cuniculus
Cavia porcellus
Ictidomys tridecemlineatus
Dipodomys ordii
Rattus norvegicus
Mus musculus
Tupaia belangeri
Otolemur garnettii
Microcebus murinus
Tarsius syrichta
Callithrix jacchus
Macaca mulatta
Nomascus leucogenys
Pongo abelii
Gorilla gorilla
Pan troglodytes
Homo sapiens
Perkinsus marinus ATCC 50983
Tetrahymena thermophila
Paramecium tetraurelia strain d4 2
Neospora caninum Liverpool
Toxoplasma gondii
Cryptosporidium muris RN66
Cryptosporidium parvum Iowa II
Cryptosporidium hominis TU502
Theileria annulata strain Ankara
Theileria parva strain Muguga
Plasmodium falciparum
Plasmodium knowlesi
Plasmodium vivax
Plasmodium chabaudi
Plasmodium yoelii yoelii 17XNL
Plasmodium berghei
Phaeodactylum tricornutum
Thalassiosira pseudonana
Pythium ultimum
Albugo laibachii
Hyaloperonospora arabidopsidis
Phytophthora ramorum
Phytophthora infestans
Phytophthora sojae
Trypanosoma brucei
Trypanosoma cruzi strain CL Brener
Leishmania braziliensis MHOM BR 75 M2904
Leishmania mexicana MHOM GT 2001 U1103
Leishmania major
Leishmania donovani
Leishmania infantum JPCM5
Entamoeba histolytica
Entamoeba dispar SAW760
Dictyostelium discoideum
Dictyostelium purpureum
Trichomonas vaginalis G3
Trichomonas vaginalis
Naegleria gruberi
Giardia lamblia ATCC 50803
OG presence
OG absence
Inferred root
Dscore = 0.884
Pvalue = 7.91e−05
AdjPvalue = 9.42e−03

NOG232612

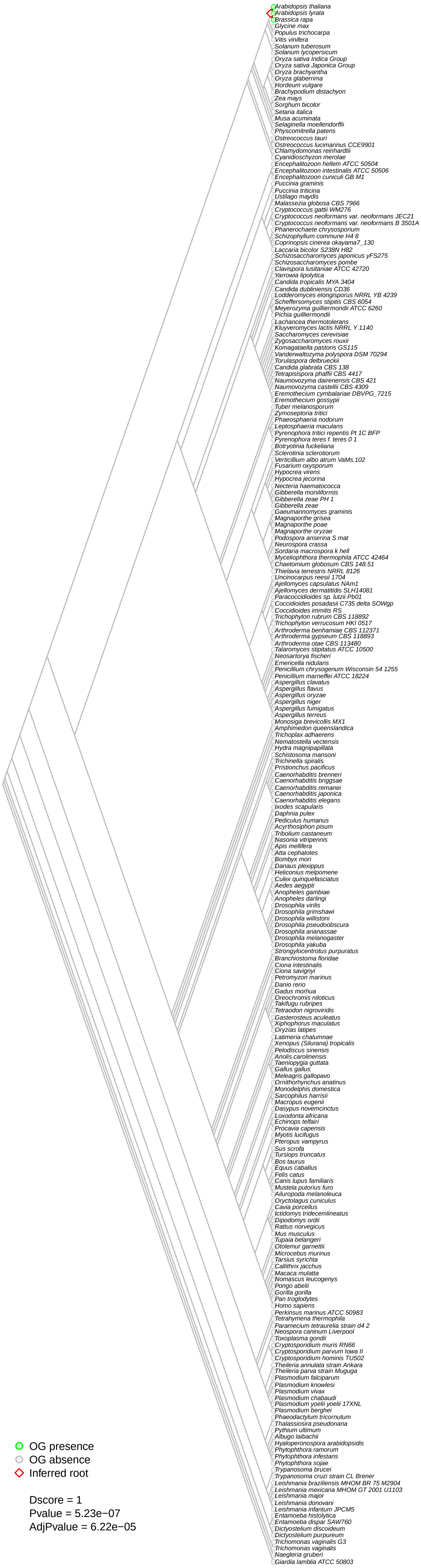
Arabidopsis thaliana
Arabidopsis lyrata
Brassica rapa
Glycine max
Populus trichocarpa
Vitis vinifera
Solanum tuberosum
Solanum lycopersicum
Oryza sativa Indica Group
Oryza sativa Japonica Group
Oryza brachyantha
Oryza glaberrima
Hordeum vulgare
Brachypodium distachyon
Zea mays
Sorghum bicolor
Setaria italica
Musa acuminata
Selaginella moellendorffii
Physcomitrella patens
Ostreococcus tauri
Ostreococcus lucimarinus CCE9901
Chlamydomonas reinhardtii
Cyanidioschyzon merolae
Encephalitozoon hellem ATCC 50504
Encephalitozoon intestinalis ATCC 50506
Encephalitozoon cuniculi GB M1
Puccinia graminis
Puccinia triticina
Ustilago maydis
Malassezia globosa CBS 7966
Cryptococcus gattii WM276
Cryptococcus neoformans var. neoformans JEC21
Cryptococcus neoformans var. neoformans B 3501A
Phanerochaete chrysosporium
Schizophyllum commune H4 8
Coprinopsis cinerea okayama7_130
Laccaria bicolor S238N H82
Schizosaccharomyces japonicus yFS275
Schizosaccharomyces pombe
Clavispora lusitaniae ATCC 42720
Yarrowia lipolytica
Candida tropicalis MYA 3404
Candida dubliniensis CD36
Lodderomyces elongisporus NRRL YB 4239
Scheffersomyces stipitis CBS 6054
Meyerozyma guilliermondii ATCC 6260
Pichia guilliermondii
Lachancea thermotolerans
Kluyveromyces lactis NRRL Y 1140
Saccharomyces cerevisiae
Zygosaccharomyces rouxii
Komagataella pastoris GS115
Vanderwaltozyma polyspora DSM 70294
Torulaspora delbrueckii
Candida glabrata CBS 138
Tetrapisispora phaffii CBS 4417
Naumovozyma dairenensis CBS 421
Naumovozyma castellii CBS 4309
Eremothecium cymbalariae DBVPG_7215
Eremothecium gossypii
Tuber melanosporum
Zymoseptoria tritici
Phaeosphaeria nodorum
Leptosphaeria maculans
Pyrenophora tritici repentis Pt 1C BFP
Pyrenophora teres f. teres 0 1
Botryotinia fuckeliana
Sclerotinia sclerotiorum
Verticillium albo atrum VaMs.102
Fusarium oxysporum
Hypocrea virens
Hypocrea jecorina
Nectria haematococca
Gibberella moniliformis
Gibberella zeae PH 1
Gibberella zeae
Gaeumannomyces graminis
Magnaporthe grisea
Magnaporthe poae
Magnaporthe oryzae
Podospora anserina S mat
Neurospora crassa
Sordaria macrospora k hell
Myceliophthora thermophila ATCC 42464
Chaetomium globosum CBS 148.51
Thielavia terrestris NRRL 8126
Uncinocarpus reesii 1704
Ajellomyces capsulatus NAm1
Ajellomyces dermatitidis SLH14081
Paracoccidioides sp. lutzii Pb01
Coccidioides posadasii C735 delta SOWgp
Coccidioides immitis RS
Trichophyton rubrum CBS 118892
Trichophyton verrucosum HKI 0517
Arthroderma benhamiae CBS 112371
Arthroderma gypseum CBS 118893
Arthroderma otae CBS 113480
Talaromyces stipitatus ATCC 10500
Neosartorya fischeri
Emericella nidulans
Penicillium chrysogenum Wisconsin 54 1255
Penicillium marneffei ATCC 18224
Aspergillus clavatus
Aspergillus flavus
Aspergillus oryzae
Aspergillus niger
Aspergillus fumigatus
Aspergillus terreus
Monosiga brevicollis MX1
Amphimedon queenslandica
Trichoplax adhaerens
Nematostella vectensis
Hydra magnipapillata
Schistosoma mansoni
Trichinella spiralis
Pristionchus pacificus
Caenorhabditis brenneri
Caenorhabditis briggsae
Caenorhabditis remanei
Caenorhabditis japonica
Caenorhabditis elegans
Ixodes scapularis
Daphnia pulex
Pediculus humanus
Acyrthosiphon pisum
Tribolium castaneum
Nasonia vitripennis
Apis mellifera
Atta cephalotes
Bombyx mori
Danaus plexippus
Heliconius melpomene
Culex quinquefasciatus
Aedes aegypti
Anopheles gambiae
Anopheles darlingi
Drosophila virilis
Drosophila grimshawi
Drosophila willistoni
Drosophila pseudoobscura
Drosophila ananassae
Drosophila melanogaster
Drosophila yakuba
Strongylocentrotus purpuratus
Branchiostoma floridae
Ciona intestinalis
Ciona savignyi
Petromyzon marinus
Danio rerio
Gadus morhua
Oreochromis niloticus
Takifugu rubripes
Tetraodon nigroviridis
Gasterosteus aculeatus
Xiphophorus maculatus
Oryzias latipes
Latimeria chalumnae
Xenopus (Silurana) tropicalis
Pelodiscus sinensis
Anolis carolinensis
Taeniopygia guttata
Gallus gallus
Meleagris gallopavo
Ornithorhynchus anatinus
Monodelphis domestica
Sarcophilus harrisii
Macropus eugenii
Dasypus novemcinctus
Loxodonta africana
Echinops telfairi
Procavia capensis
Myotis lucifugus
Pteropus vampyrus
Sus scrofa
Tursiops truncatus
Bos taurus
Equus caballus
Felis catus
Canis lupus familiaris
Mustela putorius furo
Ailuropoda melanoleuca
Oryctolagus cuniculus
Cavia porcellus
Ictidomys tridecemlineatus
Dipodomys ordii
Rattus norvegicus
Mus musculus
Tupaia belangeri
Otolemur garnettii
Microcebus murinus
Tarsius syrichta
Callithrix jacchus
Macaca mulatta
Nomascus leucogenys
Pongo abelii
Gorilla gorilla
Pan troglodytes
Homo sapiens
Perkinsus marinus ATCC 50983
Tetrahymena thermophila
Paramecium tetraurelia strain d4 2
Neospora caninum Liverpool
Toxoplasma gondii
Cryptosporidium muris RN66
Cryptosporidium parvum Iowa II
Cryptosporidium hominis TU502
Theileria annulata strain Ankara
Theileria parva strain Muguga
Plasmodium falciparum
Plasmodium knowlesi
Plasmodium vivax
Plasmodium chabaudi
Plasmodium yoelii yoelii 17XNL
Plasmodium berghei
Phaeodactylum tricornutum
Thalassiosira pseudonana
Pythium ultimum
Albugo laibachii
Hyaloperonospora arabidopsidis
Phytophthora ramorum
Phytophthora infestans
Phytophthora sojae
Trypanosoma brucei
Trypanosoma cruzi strain CL Brener
Leishmania braziliensis MHOM BR 75 M2904
Leishmania mexicana MHOM GT 2001 U1103
Leishmania major
Leishmania donovani
Leishmania infantum JPCM5
Entamoeba histolytica
Entamoeba dispar SAW760
Dictyostelium discoideum
Dictyostelium purpureum
Trichomonas vaginalis G3
Trichomonas vaginalis
Naegleria gruberi
Giardia lamblia ATCC 50803
OG presence
OG absence
Inferred root
Dscore = 1
Pvalue = 5.23e−07
AdjPvalue = 6.22e−05

NOG232879

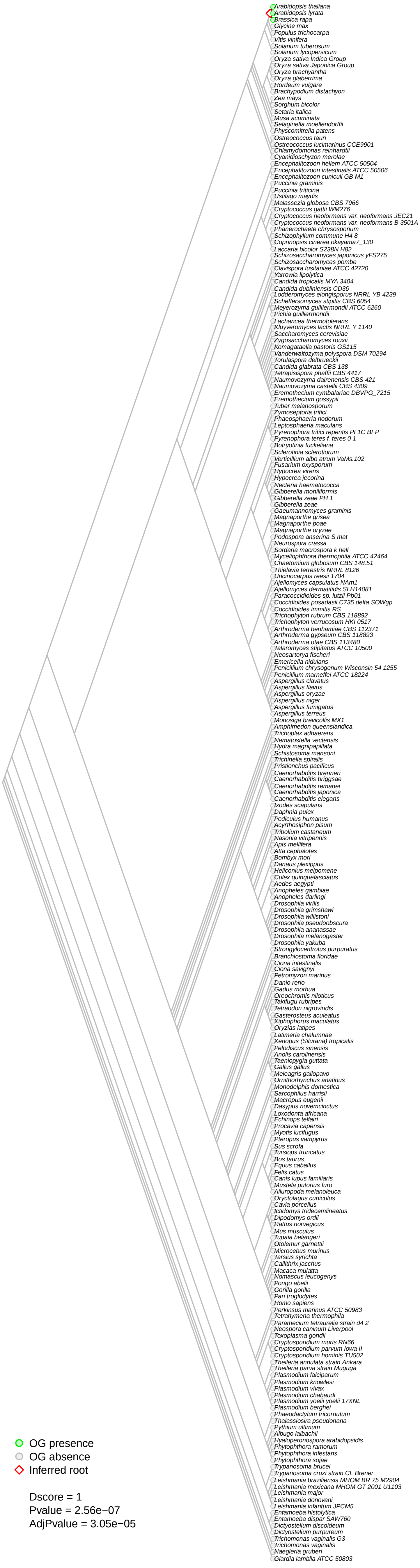

Arabidopsis thaliana
Arabidopsis lyrata
Brassica rapa
Glycine max
Populus trichocarpa
Vitis vinifera
Solanum tuberosum
Solanum lycopersicum
Oryza sativa Indica Group
Oryza sativa Japonica Group
Oryza brachyantha
Oryza glaberrima
Hordeum vulgare
Brachypodium distachyon
Zea mays
Sorghum bicolor
Setaria italica
Musa acuminata
Selaginella moellendorffii
Physcomitrella patens
Ostreococcus tauri
Ostreococcus lucimarinus CCE9901
Chlamydomonas reinhardtii
Cyanidioschyzon merolae
Encephalitozoon hellem ATCC 50504
Encephalitozoon intestinalis ATCC 50506
Encephalitozoon cuniculi GB M1
Puccinia graminis
Puccinia triticina
Ustilago maydis
Malassezia globosa CBS 7966
Cryptococcus gattii WM276
Cryptococcus neoformans var. neoformans JEC21
Cryptococcus neoformans var. neoformans B 3501A
Phanerochaete chrysosporium
Schizophyllum commune H4 8
Coprinopsis cinerea okayama7_130
Laccaria bicolor S238N H82
Schizosaccharomyces japonicus yFS275
Schizosaccharomyces pombe
Clavispora lusitaniae ATCC 42720
Yarrowia lipolytica
Candida tropicalis MYA 3404
Candida dubliniensis CD36
Lodderomyces elongisporus NRRL YB 4239
Scheffersomyces stipitis CBS 6054
Meyerozyma guilliermondii ATCC 6260
Pichia guilliermondii
Lachancea thermotolerans
Kluyveromyces lactis NRRL Y 1140
Saccharomyces cerevisiae
Zygosaccharomyces rouxii
Komagataella pastoris GS115
Vanderwaltozyma polyspora DSM 70294
Torulaspora delbrueckii
Candida glabrata CBS 138
Tetrapisispora phaffii CBS 4417
Naumovozyma dairenensis CBS 421
Naumovozyma castellii CBS 4309
Eremothecium cymbalariae DBVPG_7215
Eremothecium gossypii
Tuber melanosporum
Zymoseptoria tritici
Phaeosphaeria nodorum
Leptosphaeria maculans
Pyrenophora tritici repentis Pt 1C BFP
Pyrenophora teres f. teres 0 1
Botryotinia fuckeliana
Sclerotinia sclerotiorum
Verticillium albo atrum VaMs.102
Fusarium oxysporum
Hypocrea virens
Hypocrea jecorina
Nectria haematococca
Gibberella moniliformis
Gibberella zeae PH 1
Gibberella zeae
Gaeumannomyces graminis
Magnaporthe grisea
Magnaporthe poae
Magnaporthe oryzae
Podospora anserina S mat
Neurospora crassa
Sordaria macrospora k hell
Myceliophthora thermophila ATCC 42464
Chaetomium globosum CBS 148.51
Thielavia terrestris NRRL 8126
Uncinocarpus reesii 1704
Ajellomyces capsulatus NAm1
Ajellomyces dermatitidis SLH14081
Paracoccidioides sp. lutzii Pb01
Coccidioides posadasii C735 delta SOWgp
Coccidioides immitis RS
Trichophyton rubrum CBS 118892
Trichophyton verrucosum HKI 0517
Arthroderma benhamiae CBS 112371
Arthroderma gypseum CBS 118893
Arthroderma otae CBS 113480
Talaromyces stipitatus ATCC 10500
Neosartorya fischeri
Emericella nidulans
Penicillium chrysogenum Wisconsin 54 1255
Penicillium marneffei ATCC 18224
Aspergillus clavatus
Aspergillus flavus
Aspergillus oryzae
Aspergillus niger
Aspergillus fumigatus
Aspergillus terreus
Monosiga brevicollis MX1
Amphimedon queenslandica
Trichoplax adhaerens
Nematostella vectensis
Hydra magnipapillata
Schistosoma mansoni
Trichinella spiralis
Pristionchus pacificus
Caenorhabditis brenneri
Caenorhabditis briggsae
Caenorhabditis remanei
Caenorhabditis japonica
Caenorhabditis elegans
Ixodes scapularis
Daphnia pulex
Pediculus humanus
Acyrthosiphon pisum
Tribolium castaneum
Nasonia vitripennis
Apis mellifera
Atta cephalotes
Bombyx mori
Danaus plexippus
Heliconius melpomene
Culex quinquefasciatus
Aedes aegypti
Anopheles gambiae
Anopheles darlingi
Drosophila virilis
Drosophila grimshawi
Drosophila willistoni
Drosophila pseudoobscura
Drosophila ananassae
Drosophila melanogaster
Drosophila yakuba
Strongylocentrotus purpuratus
Branchiostoma floridae
Ciona intestinalis
Ciona savignyi
Petromyzon marinus
Danio rerio
Gadus morhua
Oreochromis niloticus
Takifugu rubripes
Tetraodon nigroviridis
Gasterosteus aculeatus
Xiphophorus maculatus
Oryzias latipes
Latimeria chalumnae
Xenopus (Silurana) tropicalis
Pelodiscus sinensis
Anolis carolinensis
Taeniopygia guttata
Gallus gallus
Meleagris gallopavo
Ornithorhynchus anatinus
Monodelphis domestica
Sarcophilus harrisii
Macropus eugenii
Dasypus novemcinctus
Loxodonta africana
Echinops telfairi
Procavia capensis
Myotis lucifugus
Pteropus vampyrus
Sus scrofa
Tursiops truncatus
Bos taurus
Equus caballus
Felis catus
Canis lupus familiaris
Mustela putorius furo
Ailuropoda melanoleuca
Oryctolagus cuniculus
Cavia porcellus
Ictidomys tridecemlineatus
Dipodomys ordii
Rattus norvegicus
Mus musculus
Tupaia belangeri
Otolemur garnettii
Microcebus murinus
Tarsius syrichta
Callithrix jacchus
Macaca mulatta
Nomascus leucogenys
Pongo abelii
Gorilla gorilla
Pan troglodytes
Homo sapiens
Perkinsus marinus ATCC 50983
Tetrahymena thermophila
Paramecium tetraurelia strain d4 2
Neospora caninum Liverpool
Toxoplasma gondii
Cryptosporidium muris RN66
Cryptosporidium parvum Iowa II
Cryptosporidium hominis TU502
Theileria annulata strain Ankara
Theileria parva strain Muguga
Plasmodium falciparum
Plasmodium knowlesi
Plasmodium vivax
Plasmodium chabaudi
Plasmodium yoelii yoelii 17XNL
Plasmodium berghei
Phaeodactylum tricornutum
Thalassiosira pseudonana
Pythium ultimum
Albugo laibachii
Hyaloperonospora arabidopsidis
Phytophthora ramorum
Phytophthora infestans
Phytophthora sojae
Trypanosoma brucei
Trypanosoma cruzi strain CL Brener
Leishmania braziliensis MHOM BR 75 M2904
Leishmania mexicana MHOM GT 2001 U1103
Leishmania major
Leishmania donovani
Leishmania infantum JPCM5
Entamoeba histolytica
Entamoeba dispar SAW760
Dictyostelium discoideum
Dictyostelium purpureum
Trichomonas vaginalis G3
Trichomonas vaginalis
Naegleria gruberi
Giardia lamblia ATCC 50803
OG presence
OG absence
Inferred root
Dscore = 1
Pvalue = 2.56e−07
AdjPvalue = 3.05e−05

NOG232889

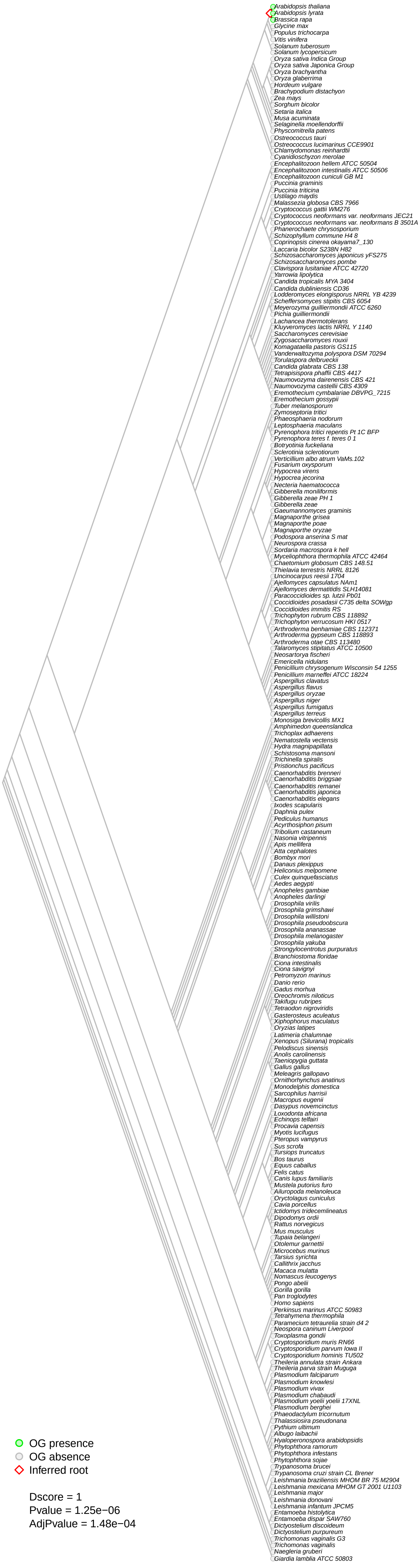

Arabidopsis thaliana
Arabidopsis lyrata
Brassica rapa
Glycine max
Populus trichocarpa
Vitis vinifera
Solanum tuberosum
Solanum lycopersicum
Oryza sativa Indica Group
Oryza sativa Japonica Group
Oryza brachyantha
Oryza glaberrima
Hordeum vulgare
Brachypodium distachyon
Zea mays
Sorghum bicolor
Setaria italica
Musa acuminata
Selaginella moellendorffii
Physcomitrella patens
Ostreococcus tauri
Ostreococcus lucimarinus CCE9901
Chlamydomonas reinhardtii
Cyanidioschyzon merolae
Encephalitozoon hellem ATCC 50504
Encephalitozoon intestinalis ATCC 50506
Encephalitozoon cuniculi GB M1
Puccinia graminis
Puccinia triticina
Ustilago maydis
Malassezia globosa CBS 7966
Cryptococcus gattii WM276
Cryptococcus neoformans var. neoformans JEC21
Cryptococcus neoformans var. neoformans B 3501A
Phanerochaete chrysosporium
Schizophyllum commune H4 8
Coprinopsis cinerea okayama7_130
Laccaria bicolor S238N H82
Schizosaccharomyces japonicus yFS275
Schizosaccharomyces pombe
Clavispora lusitaniae ATCC 42720
Yarrowia lipolytica
Candida tropicalis MYA 3404
Candida dubliniensis CD36
Lodderomyces elongisporus NRRL YB 4239
Scheffersomyces stipitis CBS 6054
Meyerozyma guilliermondii ATCC 6260
Pichia guilliermondii
Lachancea thermotolerans
Kluyveromyces lactis NRRL Y 1140
Saccharomyces cerevisiae
Zygosaccharomyces rouxii
Komagataella pastoris GS115
Vanderwaltozyma polyspora DSM 70294
Torulaspora delbrueckii
Candida glabrata CBS 138
Tetrapisispora phaffii CBS 4417
Naumovozyma dairenensis CBS 421
Naumovozyma castellii CBS 4309
Eremothecium cymbalariae DBVPG_7215
Eremothecium gossypii
Tuber melanosporum
Zymoseptoria tritici
Phaeosphaeria nodorum
Leptosphaeria maculans
Pyrenophora tritici repentis Pt 1C BFP
Pyrenophora teres f. teres 0 1
Botryotinia fuckeliana
Sclerotinia sclerotiorum
Verticillium albo atrum VaMs.102
Fusarium oxysporum
Hypocrea virens
Hypocrea jecorina
Necteria haematococca
Gibberella moniliformis
Gibberella zeae PH 1
Gibberella zeae
Gaeumannomyces graminis
Magnaporthe grisea
Magnaporthe poae
Magnaporthe oryzae
Podospora anserina S mat
Neurospora crassa
Sordaria macrospora k hell
Myceliophthora thermophila ATCC 42464
Chaetomium globosum CBS 148.51
Thielavia terrestris NRRL 8126
Uncinocarpus reesii 1704
Ajellomyces capsulatus NAm1
Ajellomyces dermatitidis SLH14081
Paracoccidioides sp. lutzii Pb01
Coccidioides posadasii C735 delta SOWgp
Coccidioides immitis RS
Trichophyton rubrum CBS 118892
Trichophyton verrucosum HKI 0517
Arthroderma benhamiae CBS 112371
Arthroderma gypseum CBS 118893
Arthroderma otae CBS 113480
Talaromyces stipitatus ATCC 10500
Neosartorya fischeri
Emericella nidulans
Penicillium chrysogenum Wisconsin 54 1255
Penicillium marneffei ATCC 18224
Aspergillus clavatus
Aspergillus flavus
Aspergillus oryzae
Aspergillus niger
Aspergillus fumigatus
Aspergillus terreus
Monosiga brevicollis MX1
Amphimedon queenslandica
Trichoplax adhaerens
Nematostella vectensis
Hydra magnipapillata
Schistosoma mansoni
Trichinella spiralis
Pristionchus pacificus
Caenorhabditis brenneri
Caenorhabditis briggsae
Caenorhabditis remanei
Caenorhabditis japonica
Caenorhabditis elegans
Ixodes scapularis
Daphnia pulex
Pediculus humanus
Acyrthosiphon pisum
Tribolium castaneum
Nasonia vitripennis
Apis mellifera
Atta cephalotes
Bombyx mori
Danaus plexippus
Heliconius melpomene
Culex quinquefasciatus
Aedes aegypti
Anopheles gambiae
Anopheles darlingi
Drosophila virilis
Drosophila grimshawi
Drosophila willistoni
Drosophila pseudoobscura
Drosophila ananassae
Drosophila melanogaster
Drosophila yakuba
Strongylocentrotus purpuratus
Branchiostoma floridae
Ciona intestinalis
Ciona savignyi
Petromyzon marinus
Danio rerio
Gadus morhua
Oreochromis niloticus
Takifugu rubripes
Tetraodon nigroviridis
Gasterosteus aculeatus
Xiphophorus maculatus
Oryzias latipes
Latimeria chalumnae
Xenopus (Silurana) tropicalis
Pelodiscus sinensis
Anolis carolinensis
Taeniopygia guttata
Gallus gallus
Meleagris gallopavo
Ornithorhynchus anatinus
Monodelphis domestica
Sarcophilus harrisii
Macropus eugenii
Dasypus novemcinctus
Loxodonta africana
Echinops telfairi
Procavia capensis
Myotis lucifugus
Pteropus vampyrus
Sus scrofa
Tursiops truncatus
Bos taurus
Equus caballus
Felis catus
Canis lupus familiaris
Mustela putorius furo
Ailuropoda melanoleuca
Oryctolagus cuniculus
Cavia porcellus
Ictidomys tridecemlineatus
Dipodomys ordii
Rattus norvegicus
Mus musculus
Tupaia belangeri
Otolemur garnettii
Microcebus murinus
Tarsius syrichta
Callithrix jacchus
Macaca mulatta
Nomascus leucogenys
Pongo abelii
Gorilla gorilla
Pan troglodytes
Homo sapiens
Perkinsus marinus ATCC 50983
Tetrahymena thermophila
Paramecium tetraurelia strain d4 2
Neospora caninum Liverpool
Toxoplasma gondii
Cryptosporidium muris RN66
Cryptosporidium parvum Iowa II
Cryptosporidium hominis TU502
Theileria annulata strain Ankara
Theileria parva strain Muguga
Plasmodium falciparum
Plasmodium knowlesi
Plasmodium vivax
Plasmodium chabaudi
Plasmodium yoelii yoelii 17XNL
Plasmodium berghei
Phaeodactylum tricornutum
Thalassiosira pseudonana
Pythium ultimum
Albugo laibachii
Hyaloperonospora arabidopsidis
Phytophthora ramorum
Phytophthora infestans
Phytophthora sojae
Trypanosoma brucei
Trypanosoma cruzi strain CL Brener
Leishmania braziliensis MHOM BR 75 M2904
Leishmania mexicana MHOM GT 2001 U1103
Leishmania major
Leishmania donovani
Leishmania infantum JPCM5
Entamoeba histolytica
Entamoeba dispar SAW760
Dictyostelium discoideum
Dictyostelium purpureum
Trichomonas vaginalis G3
Trichomonas vaginalis
Naegleria gruberi
Giardia lamblia ATCC 50803
OG presence
OG absence
Inferred root
Dscore = 1
Pvalue = 1.25e−06
AdjPvalue = 1.48e−04

NOG232894

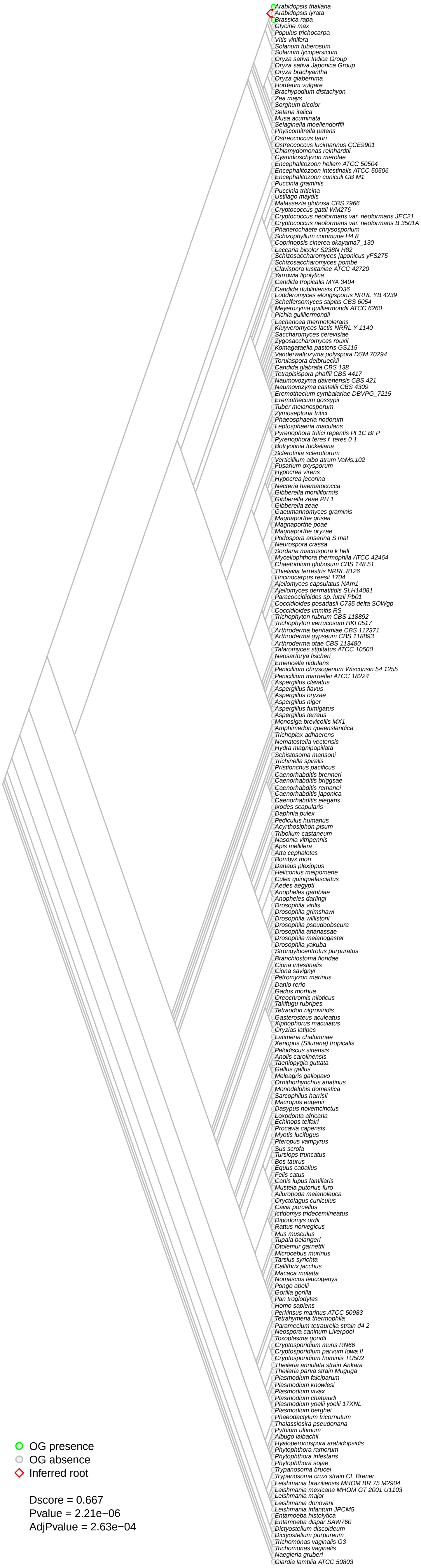
Arabidopsis thaliana
Arabidopsis lyrata
Brassica rapa
Glycine max
Populus trichocarpa
Vitis vinifera
Solanum tuberosum
Solanum lycopersicum
Oryza sativa Indica Group
Oryza sativa Japonica Group
Oryza brachyantha
Oryza glaberrima
Hordeum vulgare
Brachypodium distachyon
Zea mays
Sorghum bicolor
Setaria italica
Musa acuminata
Selaginella moellendorffii
Physcomitrella patens
Ostreococcus tauri
Ostreococcus lucimarinus CCE9901
Chlamydomonas reinhardtii
Cyanidioschyzon merolae
Encephalitozoon hellem ATCC 50504
Encephalitozoon intestinalis ATCC 50506
Encephalitozoon cuniculi GB M1
Puccinia graminis
Puccinia triticina
Ustilago maydis
Malassezia globosa CBS 7966
Cryptococcus gattii WM276
Cryptococcus neoformans var. neoformans JEC21
Cryptococcus neoformans var. neoformans B 3501A
Phanerochaete chrysosporium
Schizophyllum commune H4 8
Coprinopsis cinerea okayama7_130
Laccaria bicolor S238N H82
Schizosaccharomyces japonicus yFS275
Schizosaccharomyces pombe
Clavispora lusitaniae ATCC 42720
Yarrowia lipolytica
Candida tropicalis MYA 3404
Candida dubliniensis CD36
Lodderomyces elongisporus NRRL YB 4239
Scheffersomyces stipitis CBS 6054
Meyerozyma guilliermondii ATCC 6260
Pichia guilliermondii
Lachancea thermotolerans
Kluyveromyces lactis NRRL Y 1140
Saccharomyces cerevisiae
Zygosaccharomyces rouxii
Komagataella pastoris GS115
Vanderwaltozyma polyspora DSM 70294
Torulaspora delbrueckii
Candida glabrata CBS 138
Tetrapisispora phaffii CBS 4417
Naumovozyma dairenensis CBS 421
Naumovozyma castellii CBS 4309
Eremothecium cymbalariae DBVPG_7215
Eremothecium gossypii
Tuber melanosporum
Zymoseptoria tritici
Phaeosphaeria nodorum
Leptosphaeria maculans
Pyrenophora tritici repentis Pt 1C BFP
Pyrenophora teres f. teres 0 1
Botryotinia fuckeliana
Sclerotinia sclerotiorum
Verticillium albo atrum VaMs.102
Fusarium oxysporum
Hypocrea virens
Hypocrea jecorina
Necteria haematococca
Gibberella moniliformis
Gibberella zeae PH 1
Gibberella zeae
Gaeumannomyces graminis
Magnaporthe grisea
Magnaporthe poae
Magnaporthe oryzae
Podospora anserina S mat
Neurospora crassa
Sordaria macrospora k hell
Myceliophthora thermophila ATCC 42464
Chaetomium globosum CBS 148.51
Thielavia terrestris NRRL 8126
Uncinocarpus reesii 1704
Ajellomyces capsulatus NAm1
Ajellomyces dermatitidis SLH14081
Paracoccidioides sp. lutzii Pb01
Coccidioides posadasii C735 delta SOWgp
Coccidioides immitis RS
Trichophyton rubrum CBS 118892
Trichophyton verrucosum HKI 0517
Arthroderma benhamiae CBS 112371
Arthroderma gypseum CBS 118893
Arthroderma otae CBS 113480
Talaromyces stipitatus ATCC 10500
Neosartorya fischeri
Emericella nidulans
Penicillium chrysogenum Wisconsin 54 1255
Penicillium marneffei ATCC 18224
Aspergillus clavatus
Aspergillus flavus
Aspergillus oryzae
Aspergillus niger
Aspergillus fumigatus
Aspergillus terreus
Monosiga brevicollis MX1
Amphimedon queenslandica
Trichoplax adhaerens
Nematostella vectensis
Hydra magnipapillata
Schistosoma mansoni
Trichinella spiralis
Pristionchus pacificus
Caenorhabditis brenneri
Caenorhabditis briggsae
Caenorhabditis remanei
Caenorhabditis japonica
Caenorhabditis elegans
Ixodes scapularis
Daphnia pulex
Pediculus humanus
Acyrthosiphon pisum
Tribolium castaneum
Nasonia vitripennis
Apis mellifera
Atta cephalotes
Bombyx mori
Danaus plexippus
Heliconius melpomene
Culex quinquefasciatus
Aedes aegypti
Anopheles gambiae
Anopheles darlingi
Drosophila virilis
Drosophila grimshawi
Drosophila willistoni
Drosophila pseudoobscura
Drosophila ananassae
Drosophila melanogaster
Drosophila yakuba
Strongylocentrotus purpuratus
Branchiostoma floridae
Ciona intestinalis
Ciona savignyi
Petromyzon marinus
Danio rerio
Gadus morhua
Oreochromis niloticus
Takifugu rubripes
Tetraodon nigroviridis
Gasterosteus aculeatus
Xiphophorus maculatus
Oryzias latipes
Latimeria chalumnae
Xenopus (Silurana) tropicalis
Pelodiscus sinensis
Anolis carolinensis
Taeniopygia guttata
Gallus gallus
Meleagris gallopavo
Ornithorhynchus anatinus
Monodelphis domestica
Sarcophilus harrisii
Macropus eugenii
Dasypus novemcinctus
Loxodonta africana
Echinops telfairi
Procavia capensis
Myotis lucifugus
Pteropus vampyrus
Sus scrofa
Tursiops truncatus
Bos taurus
Equus caballus
Felis catus
Canis lupus familiaris
Mustela putorius furo
Ailuropoda melanoleuca
Oryctolagus cuniculus
Cavia porcellus
Ictidomys tridecemlineatus
Dipodomys ordii
Rattus norvegicus
Mus musculus
Tupaia belangeri
Otolemur garnettii
Microcebus murinus
Tarsius syrichta
Callithrix jacchus
Macaca mulatta
Nomascus leucogenys
Pongo abelii
Gorilla gorilla
Pan troglodytes
Homo sapiens
Perkinsus marinus ATCC 50983
Tetrahymena thermophila
Paramecium tetraurelia strain d4 2
Neospora caninum Liverpool
Toxoplasma gondii
Cryptosporidium muris RN66
Cryptosporidium parvum Iowa II
Cryptosporidium hominis TU502
Theileria annulata strain Ankara
Theileria parva strain Muguga
Plasmodium falciparum
Plasmodium knowlesi
Plasmodium vivax
Plasmodium chabaudi
Plasmodium yoelii yoelii 17XNL
Plasmodium berghei
Phaeodactylum tricornutum
Thalassiosira pseudonana
Pythium ultimum
Albugo laibachii
Hyaloperonospora arabidopsidis
Phytophthora ramorum
Phytophthora infestans
Phytophthora sojae
Trypanosoma brucei
Trypanosoma cruzi strain CL Brener
Leishmania braziliensis MHOM BR 75 M2904
Leishmania mexicana MHOM GT 2001 U1103
Leishmania major
Leishmania donovani
Leishmania infantum JPCM5
Entamoeba histolytica
Entamoeba dispar SAW760
Dictyostelium discoideum
Dictyostelium purpureum
Trichomonas vaginalis G3
Trichomonas vaginalis
Naegleria gruberi
Giardia lamblia ATCC 50803
OG presence
OG absence
Inferred root
Dscore = 0.667
Pvalue = 2.21e−06
AdjPvalue = 2.63e−04

NOG233164

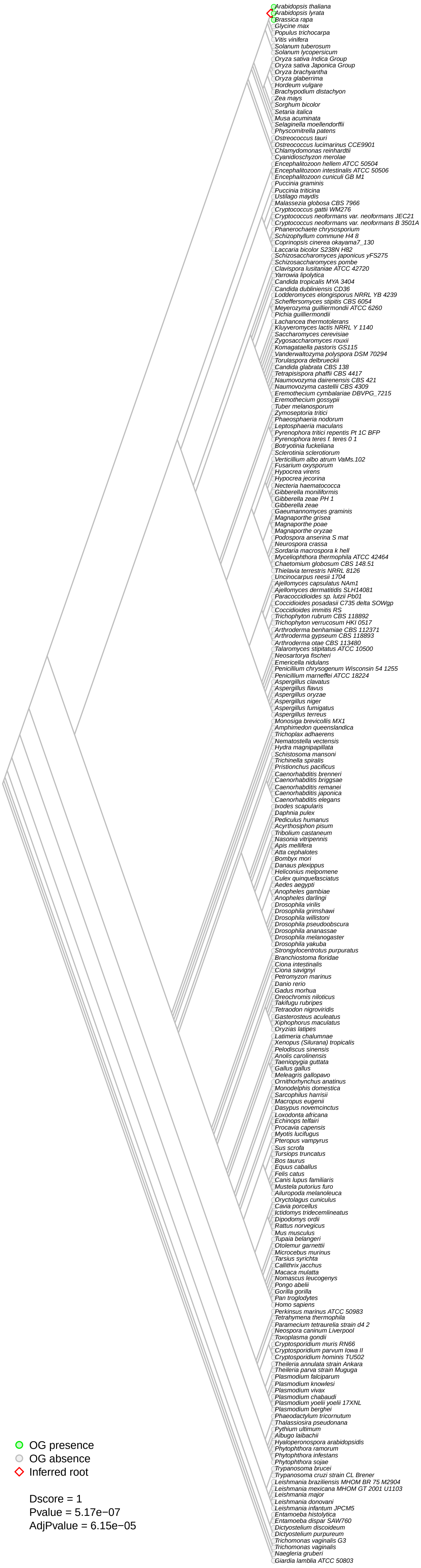
Arabidopsis thaliana
Arabidopsis lyrata
Brassica rapa
Glycine max
Populus trichocarpa
Vitis vinifera
Solanum tuberosum
Solanum lycopersicum
Oryza sativa Indica Group
Oryza sativa Japonica Group
Oryza brachyantha
Oryza glaberrima
Hordeum vulgare
Brachypodium distachyon
Zea mays
Sorghum bicolor
Setaria italica
Musa acuminata
Selaginella moellendorffii
Physcomitrella patens
Ostreococcus tauri
Ostreococcus lucimarinus CCE9901
Chlamydomonas reinhardtii
Cyanidioschyzon merolae
Encephalitozoon hellem ATCC 50504
Encephalitozoon intestinalis ATCC 50506
Encephalitozoon cuniculi GB M1
Puccinia graminis
Puccinia triticina
Ustilago maydis
Malassezia globosa CBS 7966
Cryptococcus gattii WM276
Cryptococcus neoformans var. neoformans JEC21
Cryptococcus neoformans var. neoformans B 3501A
Phanerochaete chrysosporium
Schizophyllum commune H4 8
Coprinopsis cinerea okayama7_130
Laccaria bicolor S238N H82
Schizosaccharomyces japonicus yFS275
Schizosaccharomyces pombe
Clavispora lusitaniae ATCC 42720
Yarrowia lipolytica
Candida tropicalis MYA 3404
Candida dubliniensis CD36
Lodderomyces elongisporus NRRL YB 4239
Scheffersomyces stipitis CBS 6054
Meyerozyma guilliermondii ATCC 6260
Pichia guilliermondii
Lachancea thermotolerans
Kluyveromyces lactis NRRL Y 1140
Saccharomyces cerevisiae
Zygosaccharomyces rouxii
Komagataella pastoris GS115
Vanderwaltozyma polyspora DSM 70294
Torulaspora delbrueckii
Candida glabrata CBS 138
Tetrapisispora phaffii CBS 4417
Naumovozyma dairenensis CBS 421
Naumovozyma castellii CBS 4309
Eremothecium cymbalariae DBVPG_7215
Eremothecium gossypii
Tuber melanosporum
Zymoseptoria tritici
Phaeosphaeria nodorum
Leptosphaeria maculans
Pyrenophora tritici repentis Pt 1C BFP
Pyrenophora teres f. teres 0 1
Botryotinia fuckeliana
Sclerotinia sclerotiorum
Verticillium albo atrum VaMs.102
Fusarium oxysporum
Hypocrea virens
Hypocrea jecorina
Nectria haematococca
Gibberella moniliformis
Gibberella zeae PH 1
Gibberella zeae
Gaeumannomyces graminis
Magnaporthe grisea
Magnaporthe poae
Magnaporthe oryzae
Podospora anserina S mat
Neurospora crassa
Sordaria macrospora k hell
Myceliophthora thermophila ATCC 42464
Chaetomium globosum CBS 148.51
Thielavia terrestris NRRL 8126
Uncinocarpus reesii 1704
Ajellomyces capsulatus NAm1
Ajellomyces dermatitidis SLH14081
Paracoccidioides sp. lutzii Pb01
Coccidioides posadasii C735 delta SOWgp
Coccidioides immitis RS
Trichophyton rubrum CBS 118892
Trichophyton verrucosum HKI 0517
Arthroderma benhamiae CBS 112371
Arthroderma gypseum CBS 118893
Arthroderma otae CBS 113480
Talaromyces stipitatus ATCC 10500
Neosartorya fischeri
Emericella nidulans
Penicillium chrysogenum Wisconsin 54 1255
Penicillium marneffei ATCC 18224
Aspergillus clavatus
Aspergillus flavus
Aspergillus oryzae
Aspergillus niger
Aspergillus fumigatus
Aspergillus terreus
Monosiga brevicollis MX1
Amphimedon queenslandica
Trichoplax adhaerens
Nematostella vectensis
Hydra magnipapillata
Schistosoma mansoni
Trichinella spiralis
Pristionchus pacificus
Caenorhabditis brenneri
Caenorhabditis briggsae
Caenorhabditis remanei
Caenorhabditis japonica
Caenorhabditis elegans
Ixodes scapularis
Daphnia pulex
Pediculus humanus
Acyrthosiphon pisum
Tribolium castaneum
Nasonia vitripennis
Apis mellifera
Atta cephalotes
Bombyx mori
Danaus plexippus
Heliconius melpomene
Culex quinquefasciatus
Aedes aegypti
Anopheles gambiae
Anopheles darlingi
Drosophila virilis
Drosophila grimshawi
Drosophila willistoni
Drosophila pseudoobscura
Drosophila ananassae
Drosophila melanogaster
Drosophila yakuba
Strongylocentrotus purpuratus
Branchiostoma floridae
Ciona intestinalis
Ciona savignyi
Petromyzon marinus
Danio rerio
Gadus morhua
Oreochromis niloticus
Takifugu rubripes
Tetraodon nigroviridis
Gasterosteus aculeatus
Xiphophorus maculatus
Oryzias latipes
Latimeria chalumnae
Xenopus (Silurana) tropicalis
Pelodiscus sinensis
Anolis carolinensis
Taeniopygia guttata
Gallus gallus
Meleagris gallopavo
Ornithorhynchus anatinus
Monodelphis domestica
Sarcophilus harrisii
Macropus eugenii
Dasypus novemcinctus
Loxodonta africana
Echinops telfairi
Procavia capensis
Myotis lucifugus
Pteropus vampyrus
Sus scrofa
Tursiops truncatus
Bos taurus
Equus caballus
Felis catus
Canis lupus familiaris
Mustela putorius furo
Ailuropoda melanoleuca
Oryctolagus cuniculus
Cavia porcellus
Ictidomys tridecemlineatus
Dipodomys ordii
Rattus norvegicus
Mus musculus
Tupaia belangeri
Otolemur garnettii
Microcebus murinus
Tarsius syrichta
Callithrix jacchus
Macaca mulatta
Nomascus leucogenys
Pongo abelii
Gorilla gorilla
Pan troglodytes
Homo sapiens
Perkinsus marinus ATCC 50983
Tetrahymena thermophila
Paramecium tetraurelia strain d4 2
Neospora caninum Liverpool
Toxoplasma gondii
Cryptosporidium muris RN66
Cryptosporidium parvum Iowa II
Cryptosporidium hominis TU502
Theileria annulata strain Ankara
Theileria parva strain Muguga
Plasmodium falciparum
Plasmodium knowlesi
Plasmodium vivax
Plasmodium chabaudi
Plasmodium yoelii yoelii 17XNL
Plasmodium berghei
Phaeodactylum tricornutum
Thalassiosira pseudonana
Pythium ultimum
Albugo laibachii
Hyaloperonospora arabidopsidis
Phytophthora ramorum
Phytophthora infestans
Phytophthora sojae
Trypanosoma brucei
Trypanosoma cruzi strain CL Brener
Leishmania braziliensis MHOM BR 75 M2904
Leishmania mexicana MHOM GT 2001 U1103
Leishmania major
Leishmania donovani
Leishmania infantum JPCM5
Entamoeba histolytica
Entamoeba dispar SAW760
Dictyostelium discoideum
Dictyostelium purpureum
Trichomonas vaginalis G3
Trichomonas vaginalis
Naegleria gruberi
Giardia lamblia ATCC 50803
OG presence
OG absence
Inferred root
Dscore = 1
Pvalue = 5.17e−07
AdjPvalue = 6.15e−05

NOG234501

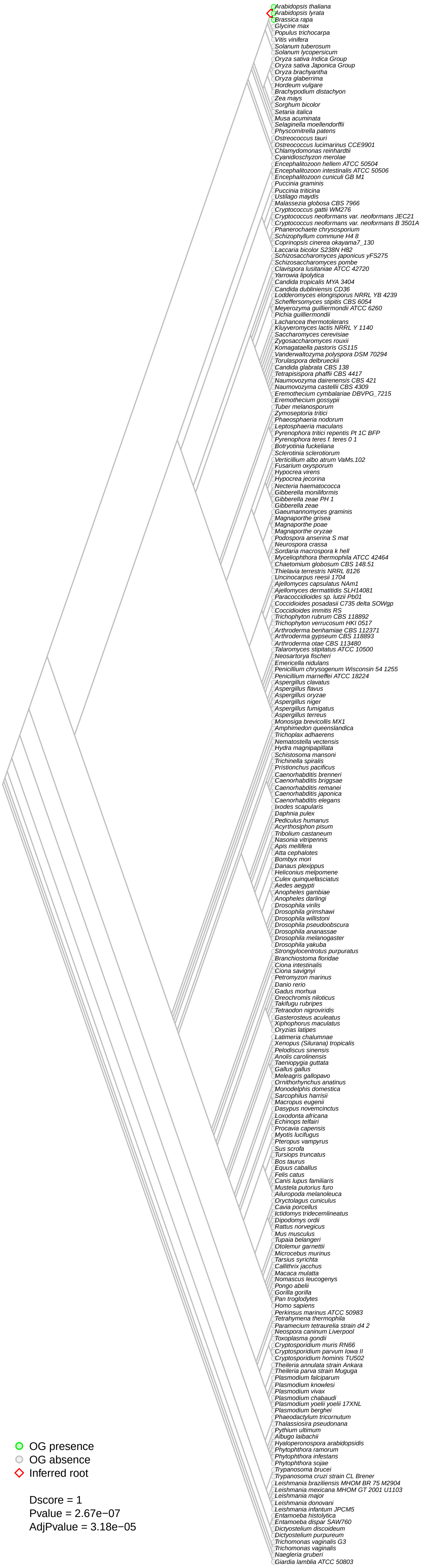
Arabidopsis thaliana
Arabidopsis lyrata
Brassica rapa
Glycine max
Populus trichocarpa
Vitis vinifera
Solanum tuberosum
Solanum lycopersicum
Oryza sativa Indica Group
Oryza sativa Japonica Group
Oryza brachyantha
Oryza glaberrima
Hordeum vulgare
Brachypodium distachyon
Zea mays
Sorghum bicolor
Setaria italica
Musa acuminata
Selaginella moellendorffii
Physcomitrella patens
Ostreococcus tauri
Ostreococcus lucimarinus CCE9901
Chlamydomonas reinhardtii
Cyanidioschyzon merolae
Encephalitozoon hellem ATCC 50504
Encephalitozoon intestinalis ATCC 50506
Encephalitozoon cuniculi GB M1
Puccinia graminis
Puccinia triticina
Ustilago maydis
Malassezia globosa CBS 7966
Cryptococcus gattii WM276
Cryptococcus neoformans var. neoformans JEC21
Cryptococcus neoformans var. neoformans B 3501A
Phanerochaete chrysosporium
Schizophyllum commune H4 8
Coprinopsis cinerea okayama7_130
Laccaria bicolor S238N H82
Schizosaccharomyces japonicus yFS275
Schizosaccharomyces pombe
Clavispora lusitaniae ATCC 42720
Yarrowia lipolytica
Candida tropicalis MYA 3404
Candida dubliniensis CD36
Lodderomyces elongisporus NRRL YB 4239
Scheffersomyces stipitis CBS 6054
Meyerozyma guilliermondii ATCC 6260
Pichia guilliermondii
Lachancea thermotolerans
Kluyveromyces lactis NRRL Y 1140
Saccharomyces cerevisiae
Zygosaccharomyces rouxii
Komagataella pastoris GS115
Vanderwaltozyma polyspora DSM 70294
Torulaspora delbrueckii
Candida glabrata CBS 138
Tetrapisispora phaffii CBS 4417
Naumovozyma dairenensis CBS 421
Naumovozyma castellii CBS 4309
Eremothecium cymbalariae DBVPG_7215
Eremothecium gossypii
Tuber melanosporum
Zymoseptoria tritici
Phaeosphaeria nodorum
Leptosphaeria maculans
Pyrenophora tritici repentis Pt 1C BFP
Pyrenophora teres f. teres 0 1
Botryotinia fuckeliana
Sclerotinia sclerotiorum
Verticillium albo atrum VaMs.102
Fusarium oxysporum
Hypocrea virens
Hypocrea jecorina
Nectria haematococca
Gibberella moniliformis
Gibberella zeae PH 1
Gibberella zeae
Gaeumannomyces graminis
Magnaporthe grisea
Magnaporthe poae
Magnaporthe oryzae
Podospora anserina S mat
Neurospora crassa
Sordaria macrospora k hell
Myceliophthora thermophila ATCC 42464
Chaetomium globosum CBS 148.51
Thielavia terrestris NRRL 8126
Uncinocarpus reesii 1704
Ajellomyces capsulatus NAm1
Ajellomyces dermatitidis SLH14081
Paracoccidioides sp. lutzii Pb01
Coccidioides posadasii C735 delta SOWgp
Coccidioides immitis RS
Trichophyton rubrum CBS 118892
Trichophyton verrucosum HKI 0517
Arthroderma benhamiae CBS 112371
Arthroderma gypseum CBS 118893
Arthroderma otae CBS 113480
Talaromyces stipitatus ATCC 10500
Neosartorya fischeri
Emericella nidulans
Penicillium chrysogenum Wisconsin 54 1255
Penicillium marneffei ATCC 18224
Aspergillus clavatus
Aspergillus flavus
Aspergillus oryzae
Aspergillus niger
Aspergillus fumigatus
Aspergillus terreus
Monosiga brevicollis MX1
Amphimedon queenslandica
Trichoplax adhaerens
Nematostella vectensis
Hydra magnipapillata
Schistosoma mansoni
Trichinella spiralis
Pristionchus pacificus
Caenorhabditis brenneri
Caenorhabditis briggsae
Caenorhabditis remanei
Caenorhabditis japonica
Caenorhabditis elegans
Ixodes scapularis
Daphnia pulex
Pediculus humanus
Acyrthosiphon pisum
Tribolium castaneum
Nasonia vitripennis
Apis mellifera
Atta cephalotes
Bombyx mori
Danaus plexippus
Heliconius melpomene
Culex quinquefasciatus
Aedes aegypti
Anopheles gambiae
Anopheles darlingi
Drosophila virilis
Drosophila grimshawi
Drosophila willistoni
Drosophila pseudoobscura
Drosophila ananassae
Drosophila melanogaster
Drosophila yakuba
Strongylocentrotus purpuratus
Branchiostoma floridae
Ciona intestinalis
Ciona savignyi
Petromyzon marinus
Danio rerio
Gadus morhua
Oreochromis niloticus
Takifugu rubripes
Tetraodon nigroviridis
Gasterosteus aculeatus
Xiphophorus maculatus
Oryzias latipes
Latimeria chalumnae
Xenopus (Silurana) tropicalis
Pelodiscus sinensis
Anolis carolinensis
Taeniopygia guttata
Gallus gallus
Meleagris gallopavo
Ornithorhynchus anatinus
Monodelphis domestica
Sarcophilus harrisii
Macropus eugenii
Dasypus novemcinctus
Loxodonta africana
Echinops telfairi
Procavia capensis
Myotis lucifugus
Pteropus vampyrus
Sus scrofa
Tursiops truncatus
Bos taurus
Equus caballus
Felis catus
Canis lupus familiaris
Mustela putorius furo
Ailuropoda melanoleuca
Oryctolagus cuniculus
Cavia porcellus
Ictidomys tridecemlineatus
Dipodomys ordii
Rattus norvegicus
Mus musculus
Tupaia belangeri
Otolemur garnettii
Microcebus murinus
Tarsius syrichta
Callithrix jacchus
Macaca mulatta
Nomascus leucogenys
Pongo abelii
Gorilla gorilla
Pan troglodytes
Homo sapiens
Perkinsus marinus ATCC 50983
Tetrahymena thermophila
Paramecium tetraurelia strain d4 2
Neospora caninum Liverpool
Toxoplasma gondii
Cryptosporidium muris RN66
Cryptosporidium parvum Iowa II
Cryptosporidium hominis TU502
Theileria annulata strain Ankara
Theileria parva strain Muguga
Plasmodium falciparum
Plasmodium knowlesi
Plasmodium vivax
Plasmodium chabaudi
Plasmodium yoelii yoelii 17XNL
Plasmodium berghei
Phaeodactylum tricornutum
Thalassiosira pseudonana
Pythium ultimum
Albugo laibachii
Hyaloperonospora arabidopsidis
Phytophthora ramorum
Phytophthora infestans
Phytophthora sojae
Trypanosoma brucei
Trypanosoma cruzi strain CL Brener
Leishmania braziliensis MHOM BR 75 M2904
Leishmania mexicana MHOM GT 2001 U1103
Leishmania major
Leishmania donovani
Leishmania infantum JPCM5
Entamoeba histolytica
Entamoeba dispar SAW760
Dictyostelium discoideum
Dictyostelium purpureum
Trichomonas vaginalis G3
Trichomonas vaginalis
Naegleria gruberi
Giardia lamblia ATCC 50803
OG presence
OG absence
Inferred root
Dscore = 1
Pvalue = 2.67e−07
AdjPvalue = 3.18e−05

NOG234820

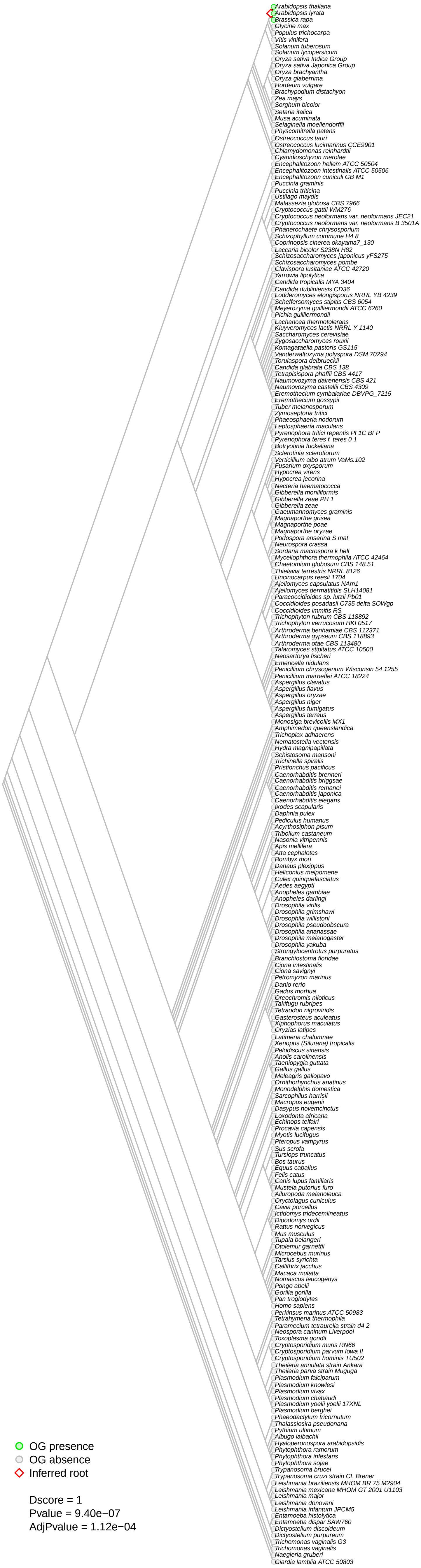
Arabidopsis thaliana
Arabidopsis lyrata
Brassica rapa
Glycine max
Populus trichocarpa
Vitis vinifera
Solanum tuberosum
Solanum lycopersicum
Oryza sativa Indica Group
Oryza sativa Japonica Group
Oryza brachyantha
Oryza glaberrima
Hordeum vulgare
Brachypodium distachyon
Zea mays
Sorghum bicolor
Setaria italica
Musa acuminata
Selaginella moellendorffii
Physcomitrella patens
Ostreococcus tauri
Ostreococcus lucimarinus CCE9901
Chlamydomonas reinhardtii
Cyanidioschyzon merolae
Encephalitozoon hellem ATCC 50504
Encephalitozoon intestinalis ATCC 50506
Encephalitozoon cuniculi GB M1
Puccinia graminis
Puccinia triticina
Ustilago maydis
Malassezia globosa CBS 7966
Cryptococcus gattii WM276
Cryptococcus neoformans var. neoformans JEC21
Cryptococcus neoformans var. neoformans B 3501A
Phanerochaete chrysosporium
Schizophyllum commune H4 8
Coprinopsis cinerea okayama7_130
Laccaria bicolor S238N H82
Schizosaccharomyces japonicus yFS275
Schizosaccharomyces pombe
Clavispora lusitaniae ATCC 42720
Yarrowia lipolytica
Candida tropicalis MYA 3404
Candida dubliniensis CD36
Lodderomyces elongisporus NRRL YB 4239
Scheffersomyces stipitis CBS 6054
Meyerozyma guilliermondii ATCC 6260
Pichia guilliermondii
Lachancea thermotolerans
Kluyveromyces lactis NRRL Y 1140
Saccharomyces cerevisiae
Zygosaccharomyces rouxii
Komagataella pastoris GS115
Vanderwaltozyma polyspora DSM 70294
Torulaspora delbrueckii
Candida glabrata CBS 138
Tetrapisispora phaffii CBS 4417
Naumovozyma dairenensis CBS 421
Naumovozyma castellii CBS 4309
Eremothecium cymbalariae DBVPG_7215
Eremothecium gossypii
Tuber melanosporum
Zymoseptoria tritici
Phaeosphaeria nodorum
Leptosphaeria maculans
Pyrenophora tritici repentis Pt 1C BFP
Pyrenophora teres f. teres 0 1
Botryotinia fuckeliana
Sclerotinia sclerotiorum
Verticillium albo atrum VaMs.102
Fusarium oxysporum
Hypocrea virens
Hypocrea jecorina
Nectria haematococca
Gibberella moniliformis
Gibberella zeae PH 1
Gibberella zeae
Gaeumannomyces graminis
Magnaporthe grisea
Magnaporthe poae
Magnaporthe oryzae
Podospora anserina S mat
Neurospora crassa
Sordaria macrospora k hell
Myceliophthora thermophila ATCC 42464
Chaetomium globosum CBS 148.51
Thielavia terrestris NRRL 8126
Uncinocarpus reesii 1704
Ajellomyces capsulatus NAm1
Ajellomyces dermatitidis SLH14081
Paracoccidioides sp. lutzii Pb01
Coccidioides posadasii C735 delta SOWgp
Coccidioides immitis RS
Trichophyton rubrum CBS 118892
Trichophyton verrucosum HKI 0517
Arthroderma benhamiae CBS 112371
Arthroderma gypseum CBS 118893
Arthroderma otae CBS 113480
Talaromyces stipitatus ATCC 10500
Neosartorya fischeri
Emericella nidulans
Penicillium chrysogenum Wisconsin 54 1255
Penicillium marneffei ATCC 18224
Aspergillus clavatus
Aspergillus flavus
Aspergillus oryzae
Aspergillus niger
Aspergillus fumigatus
Aspergillus terreus
Monosiga brevicollis MX1
Amphimedon queenslandica
Trichoplax adhaerens
Nematostella vectensis
Hydra magnipapillata
Schistosoma mansoni
Trichinella spiralis
Pristionchus pacificus
Caenorhabditis brenneri
Caenorhabditis briggsae
Caenorhabditis remanei
Caenorhabditis japonica
Caenorhabditis elegans
Ixodes scapularis
Daphnia pulex
Pediculus humanus
Acyrthosiphon pisum
Tribolium castaneum
Nasonia vitripennis
Apis mellifera
Atta cephalotes
Bombyx mori
Danaus plexippus
Heliconius melpomene
Culex quinquefasciatus
Aedes aegypti
Anopheles gambiae
Anopheles darlingi
Drosophila virilis
Drosophila grimshawi
Drosophila willistoni
Drosophila pseudoobscura
Drosophila ananassae
Drosophila melanogaster
Drosophila yakuba
Strongylocentrotus purpuratus
Branchiostoma floridae
Ciona intestinalis
Ciona savignyi
Petromyzon marinus
Danio rerio
Gadus morhua
Oreochromis niloticus
Takifugu rubripes
Tetraodon nigroviridis
Gasterosteus aculeatus
Xiphophorus maculatus
Oryzias latipes
Latimeria chalumnae
Xenopus (Silurana) tropicalis
Pelodiscus sinensis
Anolis carolinensis
Taeniopygia guttata
Gallus gallus
Meleagris gallopavo
Ornithorhynchus anatinus
Monodelphis domestica
Sarcophilus harrisii
Macropus eugenii
Dasypus novemcinctus
Loxodonta africana
Echinops telfairi
Procavia capensis
Myotis lucifugus
Pteropus vampyrus
Sus scrofa
Tursiops truncatus
Bos taurus
Equus caballus
Felis catus
Canis lupus familiaris
Mustela putorius furo
Ailuropoda melanoleuca
Oryctolagus cuniculus
Cavia porcellus
Ictidomys tridecemlineatus
Dipodomys ordii
Rattus norvegicus
Mus musculus
Tupaia belangeri
Otolemur garnettii
Microcebus murinus
Tarsius syrichta
Callithrix jacchus
Macaca mulatta
Nomascus leucogenys
Pongo abelii
Gorilla gorilla
Pan troglodytes
Homo sapiens
Perkinsus marinus ATCC 50983
Tetrahymena thermophila
Paramecium tetraurelia strain d4 2
Neospora caninum Liverpool
Toxoplasma gondii
Cryptosporidium muris RN66
Cryptosporidium parvum Iowa II
Cryptosporidium hominis TU502
Theileria annulata strain Ankara
Theileria parva strain Muguga
Plasmodium falciparum
Plasmodium knowlesi
Plasmodium vivax
Plasmodium chabaudi
Plasmodium yoelii yoelii 17XNL
Plasmodium berghei
Phaeodactylum tricornutum
Thalassiosira pseudonana
Pythium ultimum
Albugo laibachii
Hyaloperonospora arabidopsidis
Phytophthora ramorum
Phytophthora infestans
Phytophthora sojae
Trypanosoma brucei
Trypanosoma cruzi strain CL Brener
Leishmania braziliensis MHOM BR 75 M2904
Leishmania mexicana MHOM GT 2001 U1103
Leishmania major
Leishmania donovani
Leishmania infantum JPCM5
Entamoeba histolytica
Entamoeba dispar SAW760
Dictyostelium discoideum
Dictyostelium purpureum
Trichomonas vaginalis G3
Trichomonas vaginalis
Naegleria gruberi
Giardia lamblia ATCC 50803
OG presence
OG absence
Inferred root
Dscore = 1
Pvalue = 9.40e−07
AdjPvalue = 1.12e−04

NOG235401

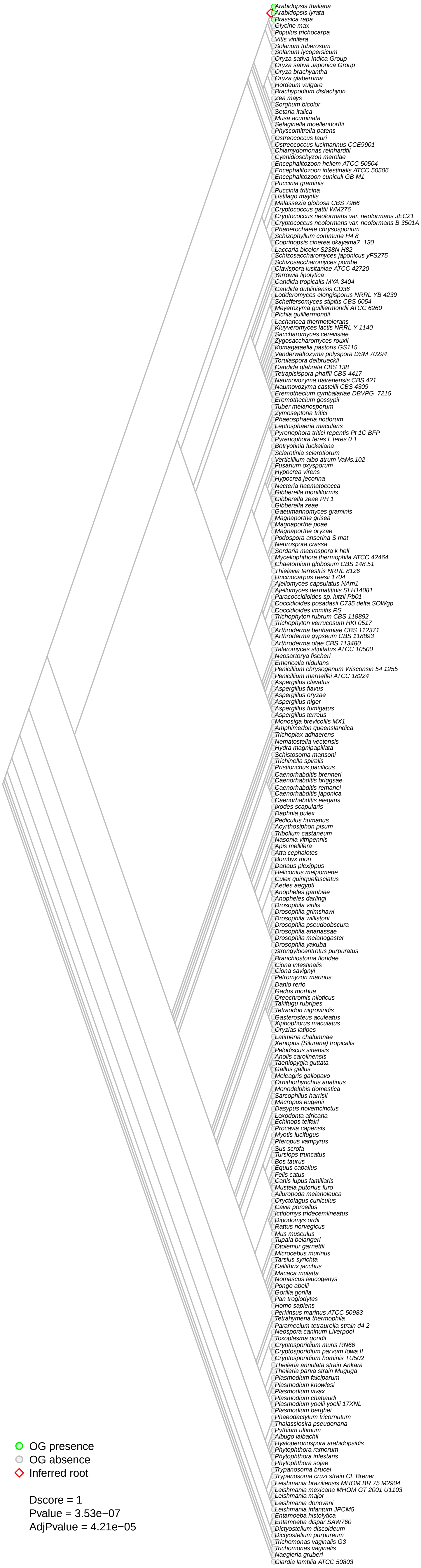

Arabidopsis thaliana
Arabidopsis lyrata
Brassica rapa
Glycine max
Populus trichocarpa
Vitis vinifera
Solanum tuberosum
Solanum lycopersicum
Oryza sativa Indica Group
Oryza sativa Japonica Group
Oryza brachyantha
Oryza glaberrima
Hordeum vulgare
Brachypodium distachyon
Zea mays
Sorghum bicolor
Setaria italica
Musa acuminata
Selaginella moellendorffii
Physcomitrella patens
Ostreococcus tauri
Ostreococcus lucimarinus CCE9901
Chlamydomonas reinhardtii
Cyanidioschyzon merolae
Encephalitozoon hellem ATCC 50504
Encephalitozoon intestinalis ATCC 50506
Encephalitozoon cuniculi GB M1
Puccinia graminis
Puccinia triticina
Ustilago maydis
Malassezia globosa CBS 7966
Cryptococcus gattii WM276
Cryptococcus neoformans var. neoformans JEC21
Cryptococcus neoformans var. neoformans B 3501A
Phanerochaete chrysosporium
Schizophyllum commune H4 8
Coprinopsis cinerea okayama7_130
Laccaria bicolor S238N H82
Schizosaccharomyces japonicus yFS275
Schizosaccharomyces pombe
Clavispora lusitaniae ATCC 42720
Yarrowia lipolytica
Candida tropicalis MYA 3404
Candida dubliniensis CD36
Lodderomyces elongisporus NRRL YB 4239
Scheffersomyces stipitis CBS 6054
Meyerozyma guilliermondii ATCC 6260
Pichia guilliermondii
Lachancea thermotolerans
Kluyveromyces lactis NRRL Y 1140
Saccharomyces cerevisiae
Zygosaccharomyces rouxii
Komagataella pastoris GS115
Vanderwaltozyma polyspora DSM 70294
Torulaspora delbrueckii
Candida glabrata CBS 138
Tetrapisispora phaffii CBS 4417
Naumovozyma dairenensis CBS 421
Naumovozyma castellii CBS 4309
Eremothecium cymbalariae DBVPG_7215
Eremothecium gossypii
Tuber melanosporum
Zymoseptoria tritici
Phaeosphaeria nodorum
Leptosphaeria maculans
Pyrenophora tritici repentis Pt 1C BFP
Pyrenophora teres f. teres 0 1
Botryotinia fuckeliana
Sclerotinia sclerotiorum
Verticillium albo atrum VaMs.102
Fusarium oxysporum
Hypocrea virens
Hypocrea jecorina
Nectria haematococca
Gibberella moniliformis
Gibberella zeae PH 1
Gibberella zeae
Gaeumannomyces graminis
Magnaporthe grisea
Magnaporthe poae
Magnaporthe oryzae
Podospora anserina S mat
Neurospora crassa
Sordaria macrospora k hell
Myceliophthora thermophila ATCC 42464
Chaetomium globosum CBS 148.51
Thielavia terrestris NRRL 8126
Uncinocarpus reesii 1704
Ajellomyces capsulatus NAm1
Ajellomyces dermatitidis SLH14081
Paracoccidioides sp. lutzii Pb01
Coccidioides posadasii C735 delta SOWgp
Coccidioides immitis RS
Trichophyton rubrum CBS 118892
Trichophyton verrucosum HKI 0517
Arthroderma benhamiae CBS 112371
Arthroderma gypseum CBS 118893
Arthroderma otae CBS 113480
Talaromyces stipitatus ATCC 10500
Neosartorya fischeri
Emericella nidulans
Penicillium chrysogenum Wisconsin 54 1255
Penicillium marneffei ATCC 18224
Aspergillus clavatus
Aspergillus flavus
Aspergillus oryzae
Aspergillus niger
Aspergillus fumigatus
Aspergillus terreus
Monosiga brevicollis MX1
Amphimedon queenslandica
Trichoplax adhaerens
Nematostella vectensis
Hydra magnipapillata
Schistosoma mansoni
Trichinella spiralis
Pristionchus pacificus
Caenorhabditis brenneri
Caenorhabditis briggsae
Caenorhabditis remanei
Caenorhabditis japonica
Caenorhabditis elegans
Ixodes scapularis
Daphnia pulex
Pediculus humanus
Acyrthosiphon pisum
Tribolium castaneum
Nasonia vitripennis
Apis mellifera
Atta cephalotes
Bombyx mori
Danaus plexippus
Heliconius melpomene
Culex quinquefasciatus
Aedes aegypti
Anopheles gambiae
Anopheles darlingi
Drosophila virilis
Drosophila grimshawi
Drosophila willistoni
Drosophila pseudoobscura
Drosophila ananassae
Drosophila melanogaster
Drosophila yakuba
Strongylocentrotus purpuratus
Branchiostoma floridae
Ciona intestinalis
Ciona savignyi
Petromyzon marinus
Danio rerio
Gadus morhua
Oreochromis niloticus
Takifugu rubripes
Tetraodon nigroviridis
Gasterosteus aculeatus
Xiphophorus maculatus
Oryzias latipes
Latimeria chalumnae
Xenopus (Silurana) tropicalis
Pelodiscus sinensis
Anolis carolinensis
Taeniopygia guttata
Gallus gallus
Meleagris gallopavo
Ornithorhynchus anatinus
Monodelphis domestica
Sarcophilus harrisii
Macropus eugenii
Dasypus novemcinctus
Loxodonta africana
Echinops telfairi
Procavia capensis
Myotis lucifugus
Pteropus vampyrus
Sus scrofa
Tursiops truncatus
Bos taurus
Equus caballus
Felis catus
Canis lupus familiaris
Mustela putorius furo
Ailuropoda melanoleuca
Oryctolagus cuniculus
Cavia porcellus
Ictidomys tridecemlineatus
Dipodomys ordii
Rattus norvegicus
Mus musculus
Tupaia belangeri
Otolemur garnettii
Microcebus murinus
Tarsius syrichta
Callithrix jacchus
Macaca mulatta
Nomascus leucogenys
Pongo abelii
Gorilla gorilla
Pan troglodytes
Homo sapiens
Perkinsus marinus ATCC 50983
Tetrahymena thermophila
Paramecium tetraurelia strain d4 2
Neospora caninum Liverpool
Toxoplasma gondii
Cryptosporidium muris RN66
Cryptosporidium parvum Iowa II
Cryptosporidium hominis TU502
Theileria annulata strain Ankara
Theileria parva strain Muguga
Plasmodium falciparum
Plasmodium knowlesi
Plasmodium vivax
Plasmodium chabaudi
Plasmodium yoelii yoelii 17XNL
Plasmodium berghei
Phaeodactylum tricornutum
Thalassiosira pseudonana
Pythium ultimum
Albugo laibachii
Hyaloperonospora arabidopsidis
Phytophthora ramorum
Phytophthora infestans
Phytophthora sojae
Trypanosoma brucei
Trypanosoma cruzi strain CL Brener
Leishmania braziliensis MHOM BR 75 M2904
Leishmania mexicana MHOM GT 2001 U1103
Leishmania major
Leishmania donovani
Leishmania infantum JPCM5
Entamoeba histolytica
Entamoeba dispar SAW760
Dictyostelium discoideum
Dictyostelium purpureum
Trichomonas vaginalis G3
Trichomonas vaginalis
Naegleria gruberi
Giardia lamblia ATCC 50803
OG presence
OG absence
Inferred root
Dscore = 1
Pvalue = 3.53e−07
AdjPvalue = 4.21e−05

NOG236110

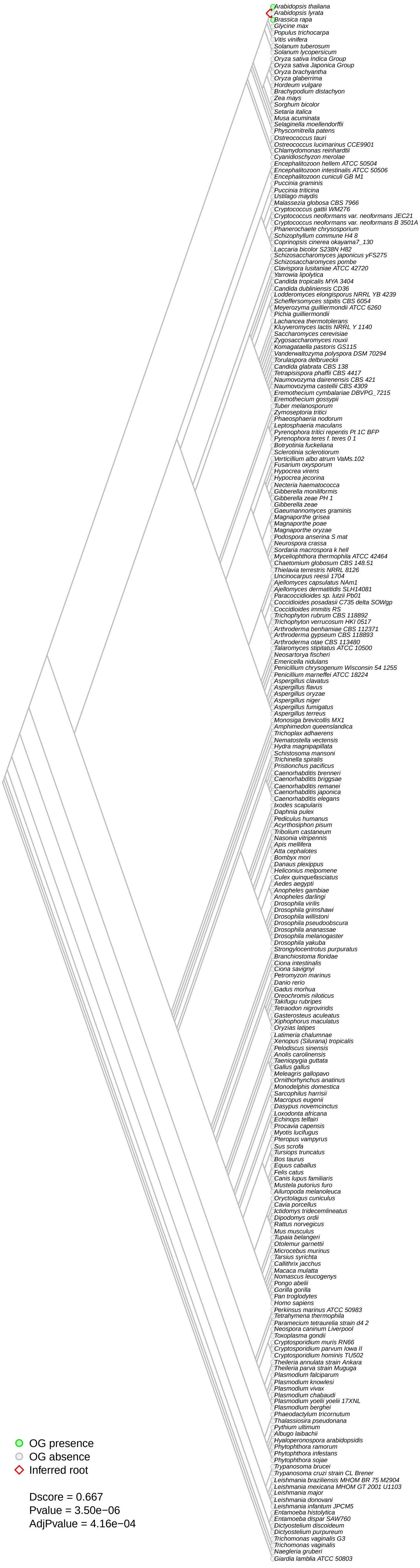
Arabidopsis thaliana
Arabidopsis lyrata
Brassica rapa
Glycine max
Populus trichocarpa
Vitis vinifera
Solanum tuberosum
Solanum lycopersicum
Oryza sativa Indica Group
Oryza sativa Japonica Group
Oryza brachyantha
Oryza glaberrima
Hordeum vulgare
Brachypodium distachyon
Zea mays
Sorghum bicolor
Setaria italica
Musa acuminata
Selaginella moellendorffii
Physcomitrella patens
Ostreococcus tauri
Ostreococcus lucimarinus CCE9901
Chlamydomonas reinhardtii
Cyanidioschyzon merolae
Encephalitozoon hellem ATCC 50504
Encephalitozoon intestinalis ATCC 50506
Encephalitozoon cuniculi GB M1
Puccinia graminis
Puccinia triticina
Ustilago maydis
Malassezia globosa CBS 7966
Cryptococcus gattii WM276
Cryptococcus neoformans var. neoformans JEC21
Cryptococcus neoformans var. neoformans B 3501A
Phanerochaete chrysosporium
Schizophyllum commune H4 8
Coprinopsis cinerea okayama7_130
Laccaria bicolor S238N H82
Schizosaccharomyces japonicus yFS275
Schizosaccharomyces pombe
Clavispora lusitaniae ATCC 42720
Yarrowia lipolytica
Candida tropicalis MYA 3404
Candida dubliniensis CD36
Lodderomyces elongisporus NRRL YB 4239
Scheffersomyces stipitis CBS 6054
Meyerozyma guilliermondii ATCC 6260
Pichia guilliermondii
Lachancea thermotolerans
Kluyveromyces lactis NRRL Y 1140
Saccharomyces cerevisiae
Zygosaccharomyces rouxii
Komagataella pastoris GS115
Vanderwaltozyma polyspora DSM 70294
Torulaspora delbrueckii
Candida glabrata CBS 138
Tetrapisispora phaffii CBS 4417
Naumovozyma dairenensis CBS 421
Naumovozyma castellii CBS 4309
Eremothecium cymbalariae DBVPG_7215
Eremothecium gossypii
Tuber melanosporum
Zymoseptoria tritici
Phaeosphaeria nodorum
Leptosphaeria maculans
Pyrenophora tritici repentis Pt 1C BFP
Pyrenophora teres f. teres 0 1
Botryotinia fuckeliana
Sclerotinia sclerotiorum
Verticillium albo atrum VaMs.102
Fusarium oxysporum
Hypocrea virens
Hypocrea jecorina
Nectria haematococca
Gibberella moniliformis
Gibberella zeae PH 1
Gibberella zeae
Gaeumannomyces graminis
Magnaporthe grisea
Magnaporthe poae
Magnaporthe oryzae
Podospora anserina S mat
Neurospora crassa
Sordaria macrospora k hell
Myceliophthora thermophila ATCC 42464
Chaetomium globosum CBS 148.51
Thielavia terrestris NRRL 8126
Uncinocarpus reesii 1704
Ajellomyces capsulatus NAm1
Ajellomyces dermatitidis SLH14081
Paracoccidioides sp. lutzii Pb01
Coccidioides posadasii C735 delta SOWgp
Coccidioides immitis RS
Trichophyton rubrum CBS 118892
Trichophyton verrucosum HKI 0517
Arthroderma benhamiae CBS 112371
Arthroderma gypseum CBS 118893
Arthroderma otae CBS 113480
Talaromyces stipitatus ATCC 10500
Neosartorya fischeri
Emericella nidulans
Penicillium chrysogenum Wisconsin 54 1255
Penicillium marneffei ATCC 18224
Aspergillus clavatus
Aspergillus flavus
Aspergillus oryzae
Aspergillus niger
Aspergillus fumigatus
Aspergillus terreus
Monosiga brevicollis MX1
Amphimedon queenslandica
Trichoplax adhaerens
Nematostella vectensis
Hydra magnipapillata
Schistosoma mansoni
Trichinella spiralis
Pristionchus pacificus
Caenorhabditis brenneri
Caenorhabditis briggsae
Caenorhabditis remanei
Caenorhabditis japonica
Caenorhabditis elegans
Ixodes scapularis
Daphnia pulex
Pediculus humanus
Acyrthosiphon pisum
Tribolium castaneum
Nasonia vitripennis
Apis mellifera
Atta cephalotes
Bombyx mori
Danaus plexippus
Heliconius melpomene
Culex quinquefasciatus
Aedes aegypti
Anopheles gambiae
Anopheles darlingi
Drosophila virilis
Drosophila grimshawi
Drosophila willistoni
Drosophila pseudoobscura
Drosophila ananassae
Drosophila melanogaster
Drosophila yakuba
Strongylocentrotus purpuratus
Branchiostoma floridae
Ciona intestinalis
Ciona savignyi
Petromyzon marinus
Danio rerio
Gadus morhua
Oreochromis niloticus
Takifugu rubripes
Tetraodon nigroviridis
Gasterosteus aculeatus
Xiphophorus maculatus
Oryzias latipes
Latimeria chalumnae
Xenopus (Silurana) tropicalis
Pelodiscus sinensis
Anolis carolinensis
Taeniopygia guttata
Gallus gallus
Meleagris gallopavo
Ornithorhynchus anatinus
Monodelphis domestica
Sarcophilus harrisii
Macropus eugenii
Dasypus novemcinctus
Loxodonta africana
Echinops telfairi
Procavia capensis
Myotis lucifugus
Pteropus vampyrus
Sus scrofa
Tursiops truncatus
Bos taurus
Equus caballus
Felis catus
Canis lupus familiaris
Mustela putorius furo
Ailuropoda melanoleuca
Oryctolagus cuniculus
Cavia porcellus
Ictidomys tridecemlineatus
Dipodomys ordii
Rattus norvegicus
Mus musculus
Tupaia belangeri
Otolemur garnettii
Microcebus murinus
Tarsius syrichta
Callithrix jacchus
Macaca mulatta
Nomascus leucogenys
Pongo abelii
Gorilla gorilla
Pan troglodytes
Homo sapiens
Perkinsus marinus ATCC 50983
Tetrahymena thermophila
Paramecium tetraurelia strain d4 2
Neospora caninum Liverpool
Toxoplasma gondii
Cryptosporidium muris RN66
Cryptosporidium parvum Iowa II
Cryptosporidium hominis TU502
Theileria annulata strain Ankara
Theileria parva strain Muguga
Plasmodium falciparum
Plasmodium knowlesi
Plasmodium vivax
Plasmodium chabaudi
Plasmodium yoelii yoelii 17XNL
Plasmodium berghei
Phaeodactylum tricornutum
Thalassiosira pseudonana
Pythium ultimum
Albugo laibachii
Hyaloperonospora arabidopsidis
Phytophthora ramorum
Phytophthora infestans
Phytophthora sojae
Trypanosoma brucei
Trypanosoma cruzi strain CL Brener
Leishmania braziliensis MHOM BR 75 M2904
Leishmania mexicana MHOM GT 2001 U1103
Leishmania major
Leishmania donovani
Leishmania infantum JPCM5
Entamoeba histolytica
Entamoeba dispar SAW760
Dictyostelium discoideum
Dictyostelium purpureum
Trichomonas vaginalis G3
Trichomonas vaginalis
Naegleria gruberi
Giardia lamblia ATCC 50803
OG presence
OG absence
Inferred root
Dscore = 0.667
Pvalue = 3.50e−06
AdjPvalue = 4.16e−04

NOG236115

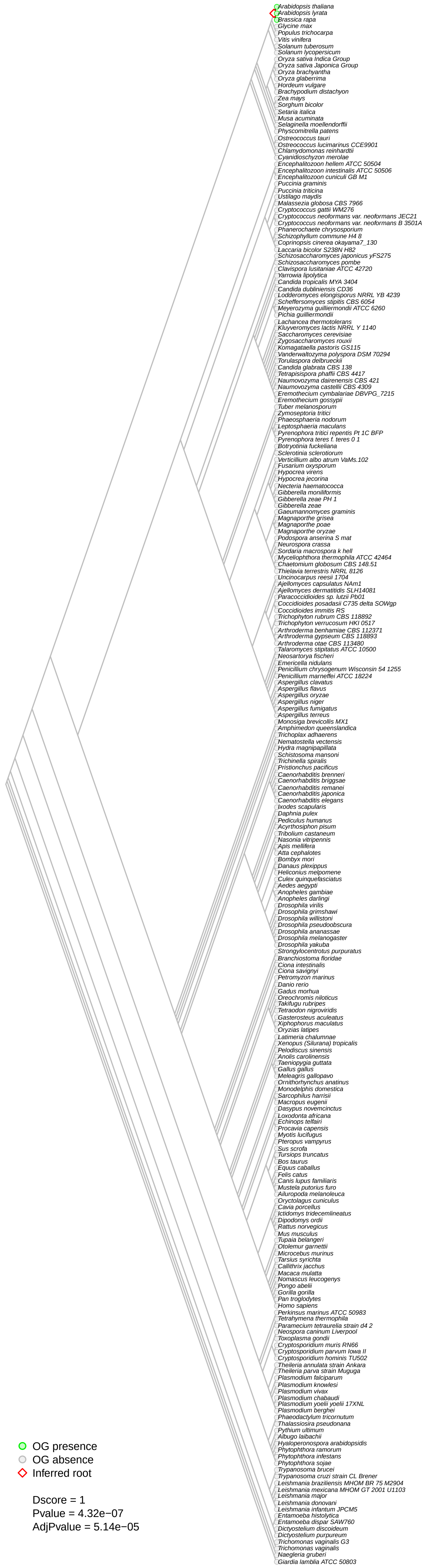
Arabidopsis thaliana
Arabidopsis lyrata
Brassica rapa
Glycine max
Populus trichocarpa
Vitis vinifera
Solanum tuberosum
Solanum lycopersicum
Oryza sativa Indica Group
Oryza sativa Japonica Group
Oryza brachyantha
Oryza glaberrima
Hordeum vulgare
Brachypodium distachyon
Zea mays
Sorghum bicolor
Setaria italica
Musa acuminata
Selaginella moellendorffii
Physcomitrella patens
Ostreococcus tauri
Ostreococcus lucimarinus CCE9901
Chlamydomonas reinhardtii
Cyanidioschyzon merolae
Encephalitozoon hellem ATCC 50504
Encephalitozoon intestinalis ATCC 50506
Encephalitozoon cuniculi GB M1
Puccinia graminis
Puccinia triticina
Ustilago maydis
Malassezia globosa CBS 7966
Cryptococcus gattii WM276
Cryptococcus neoformans var. neoformans JEC21
Cryptococcus neoformans var. neoformans B 3501A
Phanerochaete chrysosporium
Schizophyllum commune H4 8
Coprinopsis cinerea okayama7_130
Laccaria bicolor S238N H82
Schizosaccharomyces japonicus yFS275
Schizosaccharomyces pombe
Clavispora lusitaniae ATCC 42720
Yarrowia lipolytica
Candida tropicalis MYA 3404
Candida dubliniensis CD36
Lodderomyces elongisporus NRRL YB 4239
Scheffersomyces stipitis CBS 6054
Meyerozyma guilliermondii ATCC 6260
Pichia guilliermondii
Lachancea thermotolerans
Kluyveromyces lactis NRRL Y 1140
Saccharomyces cerevisiae
Zygosaccharomyces rouxii
Komagataella pastoris GS115
Vanderwaltozyma polyspora DSM 70294
Torulaspora delbrueckii
Candida glabrata CBS 138
Tetrapisispora phaffii CBS 4417
Naumovozyma dairenensis CBS 421
Naumovozyma castellii CBS 4309
Eremothecium cymbalariae DBVPG_7215
Eremothecium gossypii
Tuber melanosporum
Zymoseptoria tritici
Phaeosphaeria nodorum
Leptosphaeria maculans
Pyrenophora tritici repentis Pt 1C BFP
Pyrenophora teres f. teres 0 1
Botryotinia fuckeliana
Sclerotinia sclerotiorum
Verticillium albo atrum VaMs.102
Fusarium oxysporum
Hypocrea virens
Hypocrea jecorina
Nectria haematococca
Gibberella moniliformis
Gibberella zeae PH 1
Gibberella zeae
Gaeumannomyces graminis
Magnaporthe grisea
Magnaporthe poae
Magnaporthe oryzae
Podospora anserina S mat
Neurospora crassa
Sordaria macrospora k hell
Myceliophthora thermophila ATCC 42464
Chaetomium globosum CBS 148.51
Thielavia terrestris NRRL 8126
Uncinocarpus reesii 1704
Ajellomyces capsulatus NAm1
Ajellomyces dermatitidis SLH14081
Paracoccidioides sp. lutzii Pb01
Coccidioides posadasii C735 delta SOWgp
Coccidioides immitis RS
Trichophyton rubrum CBS 118892
Trichophyton verrucosum HKI 0517
Arthroderma benhamiae CBS 112371
Arthroderma gypseum CBS 118893
Arthroderma otae CBS 113480
Talaromyces stipitatus ATCC 10500
Neosartorya fischeri
Emericella nidulans
Penicillium chrysogenum Wisconsin 54 1255
Penicillium marneffei ATCC 18224
Aspergillus clavatus
Aspergillus flavus
Aspergillus oryzae
Aspergillus niger
Aspergillus fumigatus
Aspergillus terreus
Monosiga brevicollis MX1
Amphimedon queenslandica
Trichoplax adhaerens
Nematostella vectensis
Hydra magnipapillata
Schistosoma mansoni
Trichinella spiralis
Pristionchus pacificus
Caenorhabditis brenneri
Caenorhabditis briggsae
Caenorhabditis remanei
Caenorhabditis japonica
Caenorhabditis elegans
Ixodes scapularis
Daphnia pulex
Pediculus humanus
Acyrthosiphon pisum
Tribolium castaneum
Nasonia vitripennis
Apis mellifera
Atta cephalotes
Bombyx mori
Danaus plexippus
Heliconius melpomene
Culex quinquefasciatus
Aedes aegypti
Anopheles gambiae
Anopheles darlingi
Drosophila virilis
Drosophila grimshawi
Drosophila willistoni
Drosophila pseudoobscura
Drosophila ananassae
Drosophila melanogaster
Drosophila yakuba
Strongylocentrotus purpuratus
Branchiostoma floridae
Ciona intestinalis
Ciona savignyi
Petromyzon marinus
Danio rerio
Gadus morhua
Oreochromis niloticus
Takifugu rubripes
Tetraodon nigroviridis
Gasterosteus aculeatus
Xiphophorus maculatus
Oryzias latipes
Latimeria chalumnae
Xenopus (Silurana) tropicalis
Pelodiscus sinensis
Anolis carolinensis
Taeniopygia guttata
Gallus gallus
Meleagris gallopavo
Ornithorhynchus anatinus
Monodelphis domestica
Sarcophilus harrisii
Macropus eugenii
Dasypus novemcinctus
Loxodonta africana
Echinops telfairi
Procavia capensis
Myotis lucifugus
Pteropus vampyrus
Sus scrofa
Tursiops truncatus
Bos taurus
Equus caballus
Felis catus
Canis lupus familiaris
Mustela putorius furo
Ailuropoda melanoleuca
Oryctolagus cuniculus
Cavia porcellus
Ictidomys tridecemlineatus
Dipodomys ordii
Rattus norvegicus
Mus musculus
Tupaia belangeri
Otolemur garnettii
Microcebus murinus
Tarsius syrichta
Callithrix jacchus
Macaca mulatta
Nomascus leucogenys
Pongo abelii
Gorilla gorilla
Pan troglodytes
Homo sapiens
Perkinsus marinus ATCC 50983
Tetrahymena thermophila
Paramecium tetraurelia strain d4 2
Neospora caninum Liverpool
Toxoplasma gondii
Cryptosporidium muris RN66
Cryptosporidium parvum Iowa II
Cryptosporidium hominis TU502
Theileria annulata strain Ankara
Theileria parva strain Muguga
Plasmodium falciparum
Plasmodium knowlesi
Plasmodium vivax
Plasmodium chabaudi
Plasmodium yoelii yoelii 17XNL
Plasmodium berghei
Phaeodactylum tricornutum
Thalassiosira pseudonana
Pythium ultimum
Albugo laibachii
Hyaloperonospora arabidopsidis
Phytophthora ramorum
Phytophthora infestans
Phytophthora sojae
Trypanosoma brucei
Trypanosoma cruzi strain CL Brener
Leishmania braziliensis MHOM BR 75 M2904
Leishmania mexicana MHOM GT 2001 U1103
Leishmania major
Leishmania donovani
Leishmania infantum JPCM5
Entamoeba histolytica
Entamoeba dispar SAW760
Dictyostelium discoideum
Dictyostelium purpureum
Trichomonas vaginalis G3
Trichomonas vaginalis
Naegleria gruberi
Giardia lamblia ATCC 50803
OG presence
OG absence
Inferred root
Dscore = 1
Pvalue = 4.32e−07
AdjPvalue = 5.14e−05

NOG237024

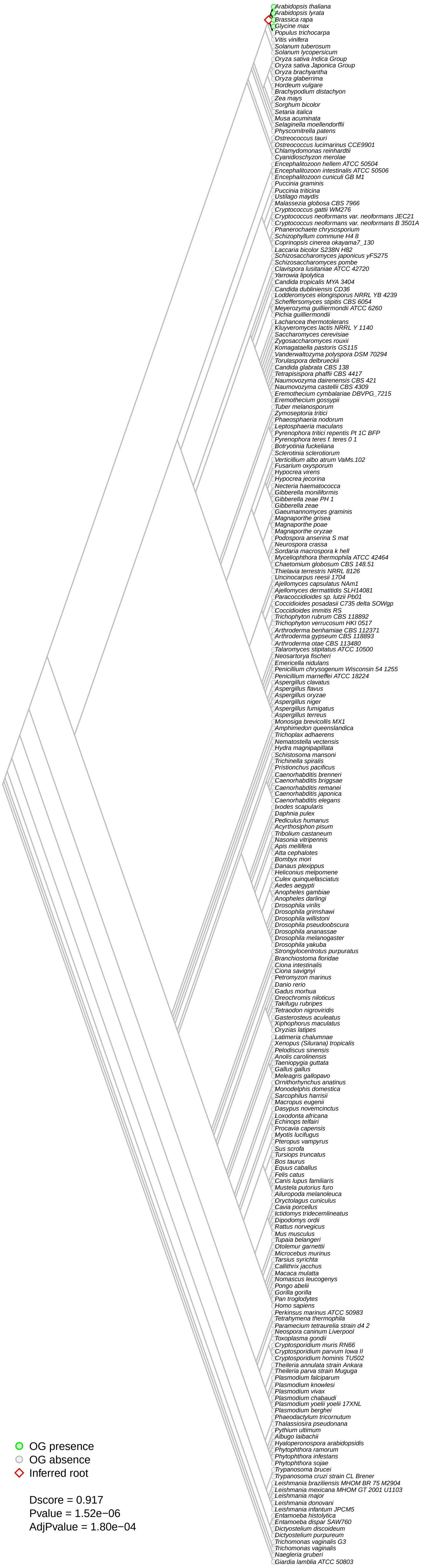

Arabidopsis thaliana
Arabidopsis lyrata
Brassica rapa
Glycine max
Populus trichocarpa
Vitis vinifera
Solanum tuberosum
Solanum lycopersicum
Oryza sativa Indica Group
Oryza sativa Japonica Group
Oryza brachyantha
Oryza glaberrima
Hordeum vulgare
Brachypodium distachyon
Zea mays
Sorghum bicolor
Setaria italica
Musa acuminata
Selaginella moellendorffii
Physcomitrella patens
Ostreococcus tauri
Ostreococcus lucimarinus CCE9901
Chlamydomonas reinhardtii
Cyanidioschyzon merolae
Encephalitozoon hellem ATCC 50504
Encephalitozoon intestinalis ATCC 50506
Encephalitozoon cuniculi GB M1
Puccinia graminis
Puccinia triticina
Ustilago maydis
Malassezia globosa CBS 7966
Cryptococcus gattii WM276
Cryptococcus neoformans var. neoformans JEC21
Cryptococcus neoformans var. neoformans B 3501A
Phanerochaete chrysosporium
Schizophyllum commune H4 8
Coprinopsis cinerea okayama7_130
Laccaria bicolor S238N H82
Schizosaccharomyces japonicus yFS275
Schizosaccharomyces pombe
Clavispora lusitaniae ATCC 42720
Yarrowia lipolytica
Candida tropicalis MYA 3404
Candida dubliniensis CD36
Lodderomyces elongisporus NRRL YB 4239
Scheffersomyces stipitis CBS 6054
Meyerozyma guilliermondii ATCC 6260
Pichia guilliermondii
Lachancea thermotolerans
Kluyveromyces lactis NRRL Y 1140
Saccharomyces cerevisiae
Zygosaccharomyces rouxii
Komagataella pastoris GS115
Vanderwaltozyma polyspora DSM 70294
Torulaspora delbrueckii
Candida glabrata CBS 138
Tetrapisispora phaffii CBS 4417
Naumovozyma dairenensis CBS 421
Naumovozyma castellii CBS 4309
Eremothecium cymbalariae DBVPG_7215
Eremothecium gossypii
Tuber melanosporum
Zymoseptoria tritici
Phaeosphaeria nodorum
Leptosphaeria maculans
Pyrenophora tritici repentis Pt 1C BFP
Pyrenophora teres f. teres 0 1
Botryotinia fuckeliana
Sclerotinia sclerotiorum
Verticillium albo atrum VaMs.102
Fusarium oxysporum
Hypocrea virens
Hypocrea jecorina
Necteria haematococca
Gibberella moniliformis
Gibberella zeae PH 1
Gibberella zeae
Gaeumannomyces graminis
Magnaporthe grisea
Magnaporthe poae
Magnaporthe oryzae
Podospora anserina S mat
Neurospora crassa
Sordaria macrospora k hell
Myceliophthora thermophila ATCC 42464
Chaetomium globosum CBS 148.51
Thielavia terrestris NRRL 8126
Uncinocarpus reesii 1704
Ajellomyces capsulatus NAm1
Ajellomyces dermatitidis SLH14081
Paracoccidioides sp. lutzii Pb01
Coccidioides posadasii C735 delta SOWgp
Coccidioides immitis RS
Trichophyton rubrum CBS 118892
Trichophyton verrucosum HKI 0517
Arthroderma benhamiae CBS 112371
Arthroderma gypseum CBS 118893
Arthroderma otae CBS 113480
Talaromyces stipitatus ATCC 10500
Neosartorya fischeri
Emericella nidulans
Penicillium chrysogenum Wisconsin 54 1255
Penicillium marneffei ATCC 18224
Aspergillus clavatus
Aspergillus flavus
Aspergillus oryzae
Aspergillus niger
Aspergillus fumigatus
Aspergillus terreus
Monosiga brevicollis MX1
Amphimedon queenslandica
Trichoplax adhaerens
Nematostella vectensis
Hydra magnipapillata
Schistosoma mansoni
Trichinella spiralis
Pristionchus pacificus
Caenorhabditis brenneri
Caenorhabditis briggsae
Caenorhabditis remanei
Caenorhabditis japonica
Caenorhabditis elegans
Ixodes scapularis
Daphnia pulex
Pediculus humanus
Acyrthosiphon pisum
Tribolium castaneum
Nasonia vitripennis
Apis mellifera
Atta cephalotes
Bombyx mori
Danaus plexippus
Heliconius melpomene
Culex quinquefasciatus
Aedes aegypti
Anopheles gambiae
Anopheles darlingi
Drosophila virilis
Drosophila grimshawi
Drosophila willistoni
Drosophila pseudoobscura
Drosophila ananassae
Drosophila melanogaster
Drosophila yakuba
Strongylocentrotus purpuratus
Branchiostoma floridae
Ciona intestinalis
Ciona savignyi
Petromyzon marinus
Danio rerio
Gadus morhua
Oreochromis niloticus
Takifugu rubripes
Tetraodon nigroviridis
Gasterosteus aculeatus
Xiphophorus maculatus
Oryzias latipes
Latimeria chalumnae
Xenopus (Silurana) tropicalis
Pelodiscus sinensis
Anolis carolinensis
Taeniopygia guttata
Gallus gallus
Meleagris gallopavo
Ornithorhynchus anatinus
Monodelphis domestica
Sarcophilus harrisii
Macropus eugenii
Dasypus novemcinctus
Loxodonta africana
Echinops telfairi
Procavia capensis
Myotis lucifugus
Pteropus vampyrus
Sus scrofa
Tursiops truncatus
Bos taurus
Equus caballus
Felis catus
Canis lupus familiaris
Mustela putorius furo
Ailuropoda melanoleuca
Oryctolagus cuniculus
Cavia porcellus
Ictidomys tridecemlineatus
Dipodomys ordii
Rattus norvegicus
Mus musculus
Tupaia belangeri
Otolemur garnettii
Microcebus murinus
Tarsius syrichta
Callithrix jacchus
Macaca mulatta
Nomascus leucogenys
Pongo abelii
Gorilla gorilla
Pan troglodytes
Homo sapiens
Perkinsus marinus ATCC 50983
Tetrahymena thermophila
Paramecium tetraurelia strain d4 2
Neospora caninum Liverpool
Toxoplasma gondii
Cryptosporidium muris RN66
Cryptosporidium parvum Iowa II
Cryptosporidium hominis TU502
Theileria annulata strain Ankara
Theileria parva strain Muguga
Plasmodium falciparum
Plasmodium knowlesi
Plasmodium vivax
Plasmodium chabaudi
Plasmodium yoelii yoelii 17XNL
Plasmodium berghei
Phaeodactylum tricornutum
Thalassiosira pseudonana
Pythium ultimum
Albugo laibachii
Hyaloperonospora arabidopsidis
Phytophthora ramorum
Phytophthora infestans
Phytophthora sojae
Trypanosoma brucei
Trypanosoma cruzi strain CL Brener
Leishmania braziliensis MHOM BR 75 M2904
Leishmania mexicana MHOM GT 2001 U1103
Leishmania major
Leishmania donovani
Leishmania infantum JPCM5
Entamoeba histolytica
Entamoeba dispar SAW760
Dictyostelium discoideum
Dictyostelium purpureum
Trichomonas vaginalis G3
Trichomonas vaginalis
Naegleria gruberi
Giardia lamblia ATCC 50803
OG presence
OG absence
Inferred root
Dscore = 0.917
Pvalue = 1.52e−06
AdjPvalue = 1.80e−04

NOG24542

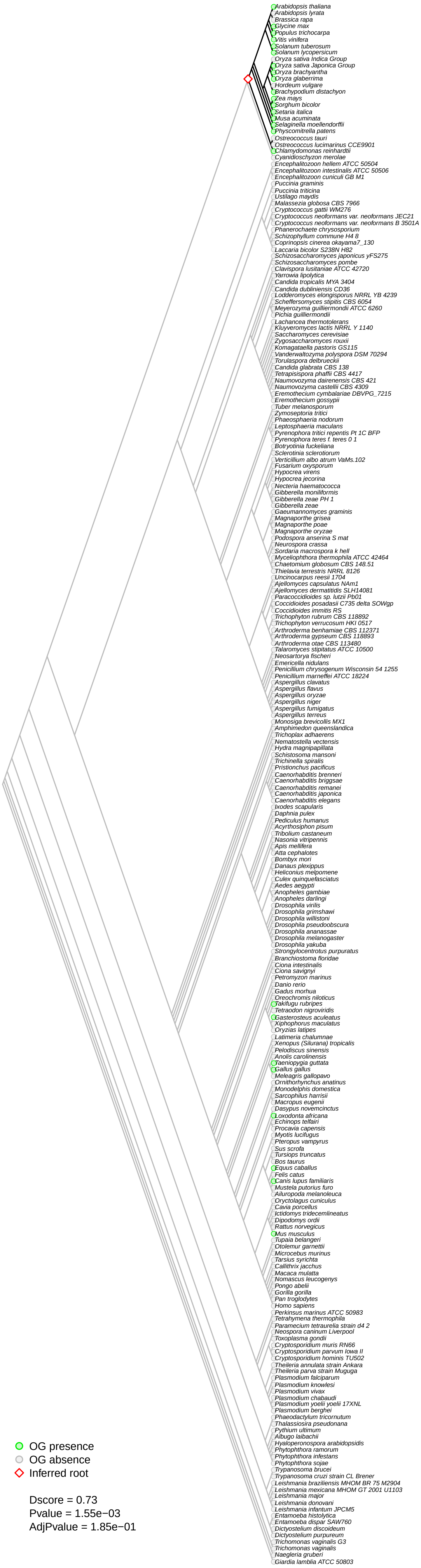
Arabidopsis thaliana
Arabidopsis lyrata
Brassica rapa
Glycine max
Populus trichocarpa
Vitis vinifera
Solanum tuberosum
Solanum lycopersicum
Oryza sativa Indica Group
Oryza sativa Japonica Group
Oryza brachyantha
Oryza glaberrima
Hordeum vulgare
Brachypodium distachyon
Zea mays
Sorghum bicolor
Setaria italica
Musa acuminata
Selaginella moellendorffii
Physcomitrella patens
Ostreococcus tauri
Ostreococcus lucimarinus CCE9901
Chlamydomonas reinhardtii
Cyanidioschyzon merolae
Encephalitozoon hellem ATCC 50504
Encephalitozoon intestinalis ATCC 50506
Encephalitozoon cuniculi GB M1
Puccinia graminis
Puccinia triticina
Ustilago maydis
Malassezia globosa CBS 7966
Cryptococcus gattii WM276
Cryptococcus neoformans var. neoformans JEC21
Cryptococcus neoformans var. neoformans B 3501A
Phanerochaete chrysosporium
Schizophyllum commune H4 8
Coprinopsis cinerea okayama7_130
Laccaria bicolor S238N H82
Schizosaccharomyces japonicus yFS275
Schizosaccharomyces pombe
Clavispora lusitaniae ATCC 42720
Yarrowia lipolytica
Candida tropicalis MYA 3404
Candida dubliniensis CD36
Lodderomyces elongisporus NRRL YB 4239
Scheffersomyces stipitis CBS 6054
Meyerozyma guilliermondii ATCC 6260
Pichia guilliermondii
Lachancea thermotolerans
Kluyveromyces lactis NRRL Y 1140
Saccharomyces cerevisiae
Zygosaccharomyces rouxii
Komagataella pastoris GS115
Vanderwaltozyma polyspora DSM 70294
Torulaspora delbrueckii
Candida glabrata CBS 138
Tetrapisispora phaffii CBS 4417
Naumovozyma dairenensis CBS 421
Naumovozyma castellii CBS 4309
Eremothecium cymbalariae DBVPG_7215
Eremothecium gossypii
Tuber melanosporum
Zymoseptoria tritici
Phaeosphaeria nodorum
Leptosphaeria maculans
Pyrenophora tritici repentis Pt 1C BFP
Pyrenophora teres f. teres 0 1
Botryotinia fuckeliana
Sclerotinia sclerotiorum
Verticillium albo atrum VaMs.102
Fusarium oxysporum
Hypocrea virens
Hypocrea jecorina
Nectria haematococca
Gibberella moniliformis
Gibberella zeae PH 1
Gibberella zeae
Gaeumannomyces graminis
Magnaporthe grisea
Magnaporthe poae
Magnaporthe oryzae
Podospora anserina S mat
Neurospora crassa
Sordaria macrospora k hell
Myceliophthora thermophila ATCC 42464
Chaetomium globosum CBS 148.51
Thielavia terrestris NRRL 8126
Uncinocarpus reesii 1704
Ajellomyces capsulatus NAm1
Ajellomyces dermatitidis SLH14081
Paracoccidioides sp. lutzii Pb01
Coccidioides posadasii C735 delta SOWgp
Coccidioides immitis RS
Trichophyton rubrum CBS 118892
Trichophyton verrucosum HKI 0517
Arthroderma benhamiae CBS 112371
Arthroderma gypseum CBS 118893
Arthroderma otae CBS 113480
Talaromyces stipitatus ATCC 10500
Neosartorya fischeri
Emericella nidulans
Penicillium chrysogenum Wisconsin 54 1255
Penicillium marneffei ATCC 18224
Aspergillus clavatus
Aspergillus flavus
Aspergillus oryzae
Aspergillus niger
Aspergillus fumigatus
Aspergillus terreus
Monosiga brevicollis MX1
Amphimedon queenslandica
Trichoplax adhaerens
Nematostella vectensis
Hydra magnipapillata
Schistosoma mansoni
Trichinella spiralis
Pristionchus pacificus
Caenorhabditis brenneri
Caenorhabditis briggsae
Caenorhabditis remanei
Caenorhabditis japonica
Caenorhabditis elegans
Ixodes scapularis
Daphnia pulex
Pediculus humanus
Acyrthosiphon pisum
Tribolium castaneum
Nasonia vitripennis
Apis mellifera
Atta cephalotes
Bombyx mori
Danaus plexippus
Heliconius melpomene
Culex quinquefasciatus
Aedes aegypti
Anopheles gambiae
Anopheles darlingi
Drosophila virilis
Drosophila grimshawi
Drosophila willistoni
Drosophila pseudoobscura
Drosophila ananassae
Drosophila melanogaster
Drosophila yakuba
Strongylocentrotus purpuratus
Branchiostoma floridae
Ciona intestinalis
Ciona savignyi
Petromyzon marinus
Danio rerio
Gadus morhua
Oreochromis niloticus
Takifugu rubripes
Tetraodon nigroviridis
Gasterosteus aculeatus
Xiphophorus maculatus
Oryzias latipes
Latimeria chalumnae
Xenopus (Silurana) tropicalis
Pelodiscus sinensis
Anolis carolinensis
Taeniopygia guttata
Gallus gallus
Meleagris gallopavo
Ornithorhynchus anatinus
Monodelphis domestica
Sarcophilus harrisii
Macropus eugenii
Dasypus novemcinctus
Loxodonta africana
Echinops telfairi
Procavia capensis
Myotis lucifugus
Pteropus vampyrus
Sus scrofa
Tursiops truncatus
Bos taurus
Equus caballus
Felis catus
Canis lupus familiaris
Mustela putorius furo
Ailuropoda melanoleuca
Oryctolagus cuniculus
Cavia porcellus
Ictidomys tridecemlineatus
Dipodomys ordii
Rattus norvegicus
Mus musculus
Tupaia belangeri
Otolemur garnettii
Microcebus murinus
Tarsius syrichta
Callithrix jacchus
Macaca mulatta
Nomascus leucogenys
Pongo abelii
Gorilla gorilla
Pan troglodytes
Homo sapiens
Perkinsus marinus ATCC 50983
Tetrahymena thermophila
Paramecium tetraurelia strain d4 2
Neospora caninum Liverpool
Toxoplasma gondii
Cryptosporidium muris RN66
Cryptosporidium parvum Iowa II
Cryptosporidium hominis TU502
Theileria annulata strain Ankara
Theileria parva strain Muguga
Plasmodium falciparum
Plasmodium knowlesi
Plasmodium vivax
Plasmodium chabaudi
Plasmodium yoelii yoelii 17XNL
Plasmodium berghei
Phaeodactylum tricornutum
Thalassiosira pseudonana
Pythium ultimum
Albugo laibachii
Hyaloperonospora arabidopsidis
Phytophthora ramorum
Phytophthora infestans
Phytophthora sojae
Trypanosoma brucei
Trypanosoma cruzi strain CL Brener
Leishmania braziliensis MHOM BR 75 M2904
Leishmania mexicana MHOM GT 2001 U1103
Leishmania major
Leishmania donovani
Leishmania infantum JPCM5
Entamoeba histolytica
Entamoeba dispar SAW760
Dictyostelium discoideum
Dictyostelium purpureum
Trichomonas vaginalis G3
Trichomonas vaginalis
Naegleria gruberi
Giardia lamblia ATCC 50803
OG presence
OG absence
Inferred root
Dscore = 0.73
Pvalue = 1.55e−03
AdjPvalue = 1.85e−01

NOG24739

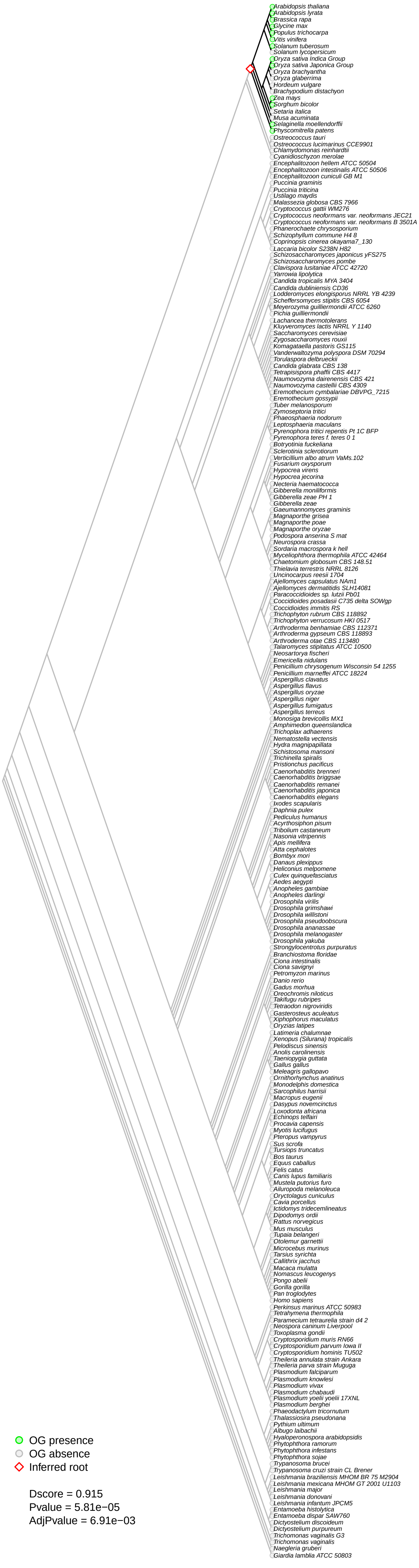

Dscore = 0.915
Pvalue = 5.81e−05
AdjPvalue = 6.91e−03

NOG26018

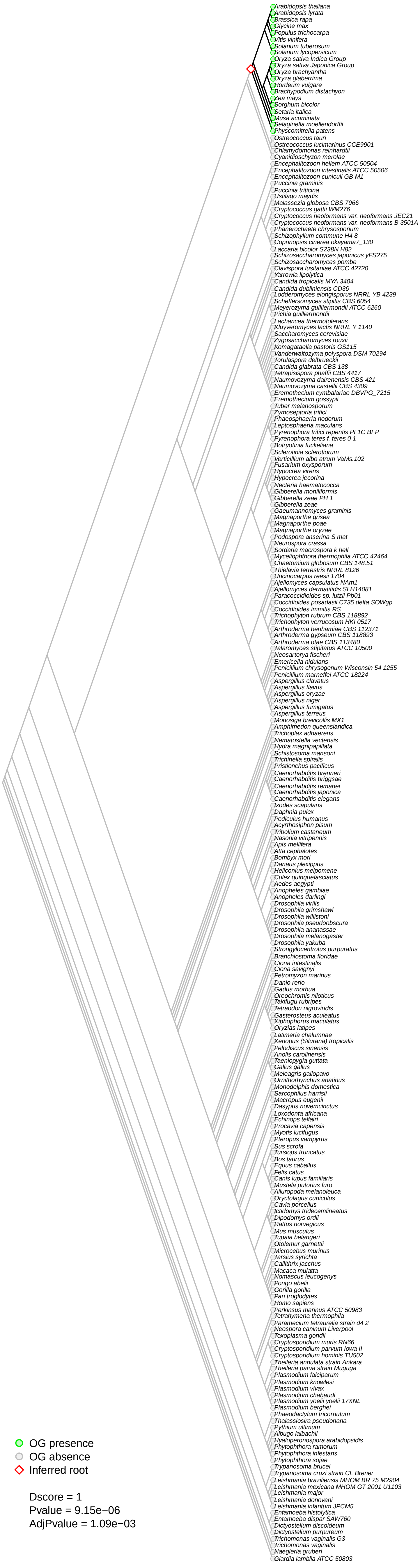
Arabidopsis thaliana
Arabidopsis lyrata
Brassica rapa
Glycine max
Populus trichocarpa
Vitis vinifera
Solanum tuberosum
Solanum lycopersicum
Oryza sativa Indica Group
Oryza sativa Japonica Group
Oryza brachyantha
Oryza glaberrima
Hordeum vulgare
Brachypodium distachyon
Zea mays
Sorghum bicolor
Setaria italica
Musa acuminata
Selaginella moellendorffii
Physcomitrella patens
Ostreococcus tauri
Ostreococcus lucimarinus CCE9901
Chlamydomonas reinhardtii
Cyanidioschyzon merolae
Encephalitozoon hellem ATCC 50504
Encephalitozoon intestinalis ATCC 50506
Encephalitozoon cuniculi GB M1
Puccinia graminis
Puccinia triticina
Ustilago maydis
Malassezia globosa CBS 7966
Cryptococcus gattii WM276
Cryptococcus neoformans var. neoformans JEC21
Cryptococcus neoformans var. neoformans B 3501A
Phanerochaete chrysosporium
Schizophyllum commune H4 8
Coprinopsis cinerea okayama7_130
Laccaria bicolor S238N H82
Schizosaccharomyces japonicus yFS275
Schizosaccharomyces pombe
Clavispora lusitaniae ATCC 42720
Yarrowia lipolytica
Candida tropicalis MYA 3404
Candida dubliniensis CD36
Lodderomyces elongisporus NRRL YB 4239
Scheffersomyces stipitis CBS 6054
Meyerozyma guilliermondii ATCC 6260
Pichia guilliermondii
Lachancea thermotolerans
Kluyveromyces lactis NRRL Y 1140
Saccharomyces cerevisiae
Zygosaccharomyces rouxii
Komagataella pastoris GS115
Vanderwaltozyma polyspora DSM 70294
Torulaspora delbrueckii
Candida glabrata CBS 138
Tetrapisispora phaffii CBS 4417
Naumovozyma dairenensis CBS 421
Naumovozyma castellii CBS 4309
Eremothecium cymbalariae DBVPG_7215
Eremothecium gossypii
Tuber melanosporum
Zymoseptoria tritici
Phaeosphaeria nodorum
Leptosphaeria maculans
Pyrenophora tritici repentis Pt 1C BFP
Pyrenophora teres f. teres 0 1
Botryotinia fuckeliana
Sclerotinia sclerotiorum
Verticillium albo atrum VaMs.102
Fusarium oxysporum
Hypocrea virens
Hypocrea jecorina
Nectria haematococca
Gibberella moniliformis
Gibberella zeae PH 1
Gibberella zeae
Gaeumannomyces graminis
Magnaporthe grisea
Magnaporthe poae
Magnaporthe oryzae
Podospora anserina S mat
Neurospora crassa
Sordaria macrospora k hell
Myceliophthora thermophila ATCC 42464
Chaetomium globosum CBS 148.51
Thielavia terrestris NRRL 8126
Uncinocarpus reesii 1704
Ajellomyces capsulatus NAm1
Ajellomyces dermatitidis SLH14081
Paracoccidioides sp. lutzii Pb01
Coccidioides posadasii C735 delta SOWgp
Coccidioides immitis RS
Trichophyton rubrum CBS 118892
Trichophyton verrucosum HKI 0517
Arthroderma benhamiae CBS 112371
Arthroderma gypseum CBS 118893
Arthroderma otae CBS 113480
Talaromyces stipitatus ATCC 10500
Neosartorya fischeri
Emericella nidulans
Penicillium chrysogenum Wisconsin 54 1255
Penicillium marneffei ATCC 18224
Aspergillus clavatus
Aspergillus flavus
Aspergillus oryzae
Aspergillus niger
Aspergillus fumigatus
Aspergillus terreus
Monosiga brevicollis MX1
Amphimedon queenslandica
Trichoplax adhaerens
Nematostella vectensis
Hydra magnipapillata
Schistosoma mansoni
Trichinella spiralis
Pristionchus pacificus
Caenorhabditis brenneri
Caenorhabditis briggsae
Caenorhabditis remanei
Caenorhabditis japonica
Caenorhabditis elegans
Ixodes scapularis
Daphnia pulex
Pediculus humanus
Acyrthosiphon pisum
Tribolium castaneum
Nasonia vitripennis
Apis mellifera
Atta cephalotes
Bombyx mori
Danaus plexippus
Heliconius melpomene
Culex quinquefasciatus
Aedes aegypti
Anopheles gambiae
Anopheles darlingi
Drosophila virilis
Drosophila grimshawi
Drosophila willistoni
Drosophila pseudoobscura
Drosophila ananassae
Drosophila melanogaster
Drosophila yakuba
Strongylocentrotus purpuratus
Branchiostoma floridae
Ciona intestinalis
Ciona savignyi
Petromyzon marinus
Danio rerio
Gadus morhua
Oreochromis niloticus
Takifugu rubripes
Tetraodon nigroviridis
Gasterosteus aculeatus
Xiphophorus maculatus
Oryzias latipes
Latimeria chalumnae
Xenopus (Silurana) tropicalis
Pelodiscus sinensis
Anolis carolinensis
Taeniopygia guttata
Gallus gallus
Meleagris gallopavo
Ornithorhynchus anatinus
Monodelphis domestica
Sarcophilus harrisii
Macropus eugenii
Dasypus novemcinctus
Loxodonta africana
Echinops telfairi
Procavia capensis
Myotis lucifugus
Pteropus vampyrus
Sus scrofa
Tursiops truncatus
Bos taurus
Equus caballus
Felis catus
Canis lupus familiaris
Mustela putorius furo
Ailuropoda melanoleuca
Oryctolagus cuniculus
Cavia porcellus
Ictidomys tridecemlineatus
Dipodomys ordii
Rattus norvegicus
Mus musculus
Tupaia belangeri
Otolemur garnettii
Microcebus murinus
Tarsius syrichta
Callithrix jacchus
Macaca mulatta
Nomascus leucogenys
Pongo abelii
Gorilla gorilla
Pan troglodytes
Homo sapiens
Perkinsus marinus ATCC 50983
Tetrahymena thermophila
Paramecium tetraurelia strain d4 2
Neospora caninum Liverpool
Toxoplasma gondii
Cryptosporidium muris RN66
Cryptosporidium parvum Iowa II
Cryptosporidium hominis TU502
Theileria annulata strain Ankara
Theileria parva strain Muguga
Plasmodium falciparum
Plasmodium knowlesi
Plasmodium vivax
Plasmodium chabaudi
Plasmodium yoelii yoelii 17XNL
Plasmodium berghei
Phaeodactylum tricornutum
Thalassiosira pseudonana
Pythium ultimum
Albugo laibachii
Hyaloperonospora arabidopsidis
Phytophthora ramorum
Phytophthora infestans
Phytophthora sojae
Trypanosoma brucei
Trypanosoma cruzi strain CL Brener
Leishmania braziliensis MHOM BR 75 M2904
Leishmania mexicana MHOM GT 2001 U1103
Leishmania major
Leishmania donovani
Leishmania infantum JPCM5
Entamoeba histolytica
Entamoeba dispar SAW760
Dictyostelium discoideum
Dictyostelium purpureum
Trichomonas vaginalis G3
Trichomonas vaginalis
Naegleria gruberi
Giardia lamblia ATCC 50803
OG presence
OG absence
Inferred root
Dscore = 1
Pvalue = 9.15e−06
AdjPvalue = 1.09e−03

NOG269739

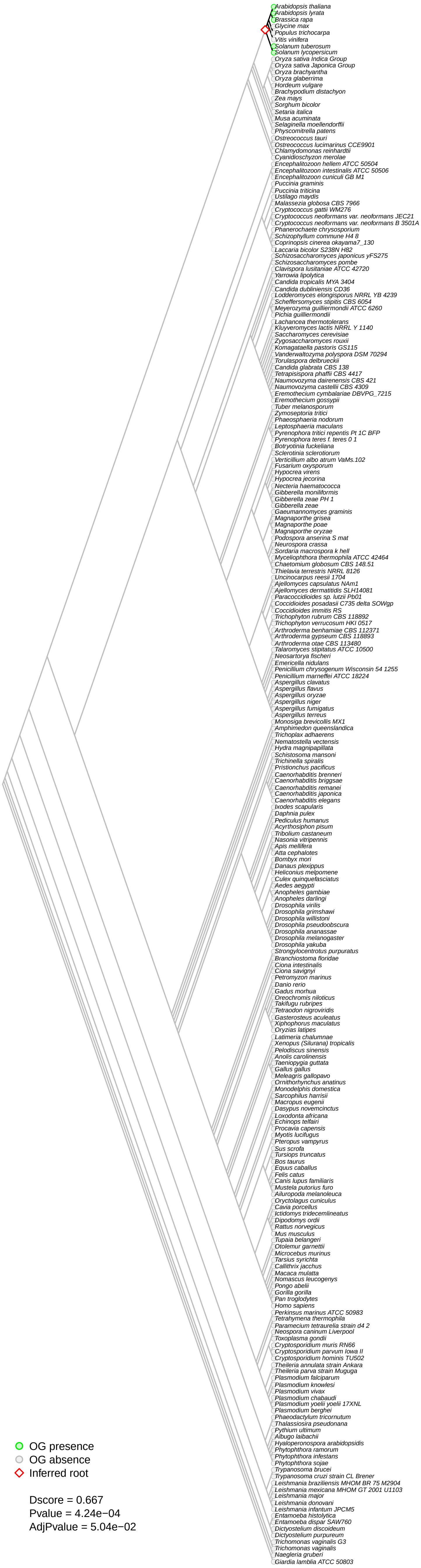

OG presence
OG absence
Inferred root

Dscore = 0.667
Pvalue = 4.24e−04
AdjPvalue = 5.04e−02

NOG271274

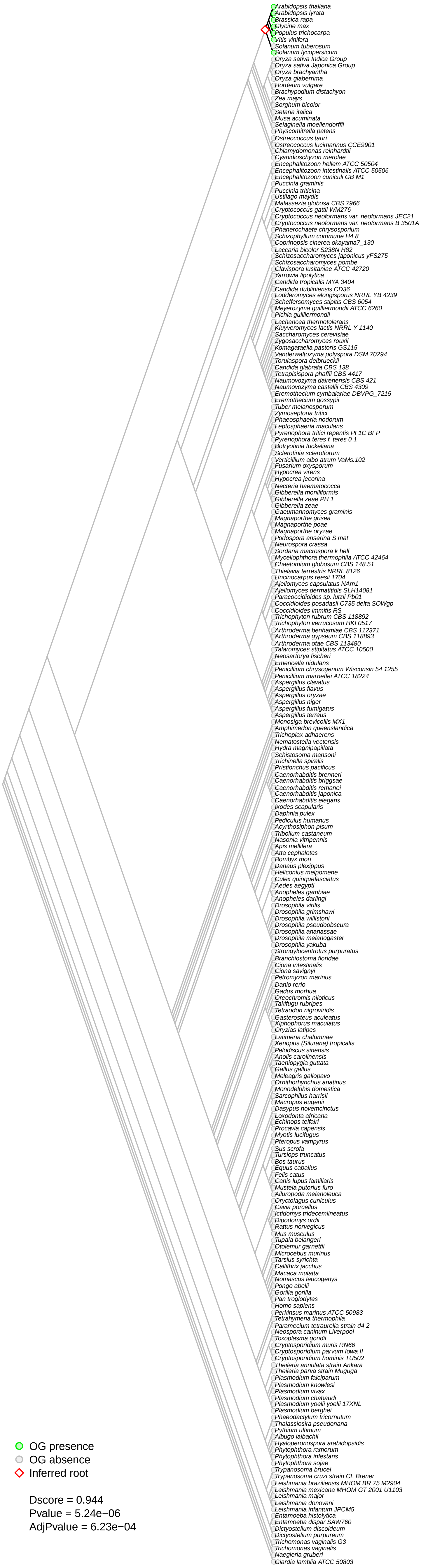

OG presence
OG absence
Inferred root

Dscore = 0.944
Pvalue = 5.24e−06
AdjPvalue = 6.23e−04

NOG272155

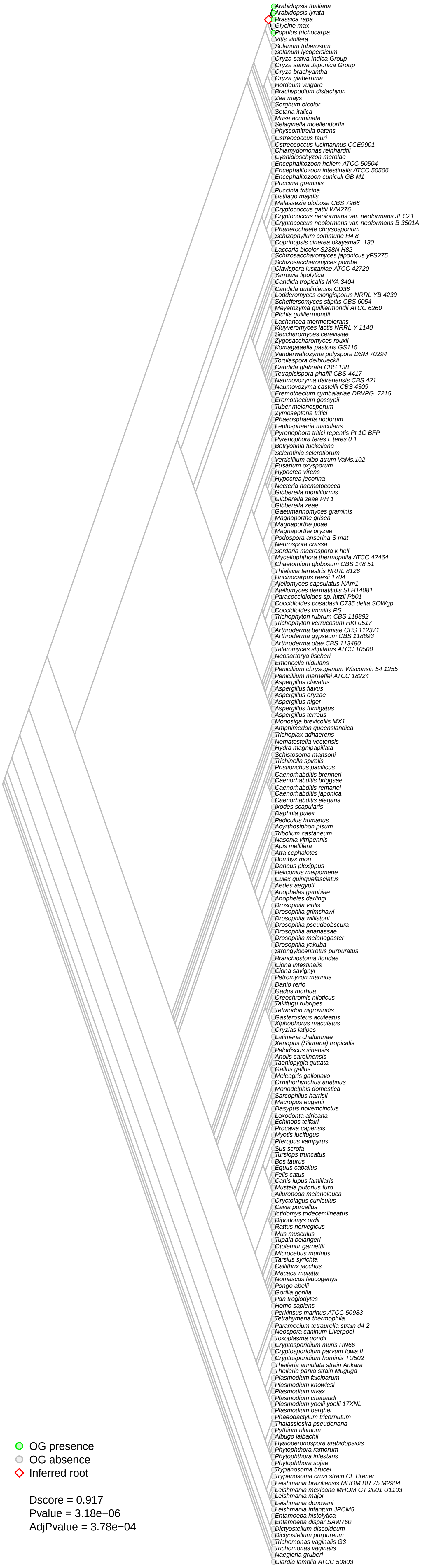

OG presence
OG absence
Inferred root

Dscore = 0.917
Pvalue = 3.18e−06
AdjPvalue = 3.78e−04

NOG275802

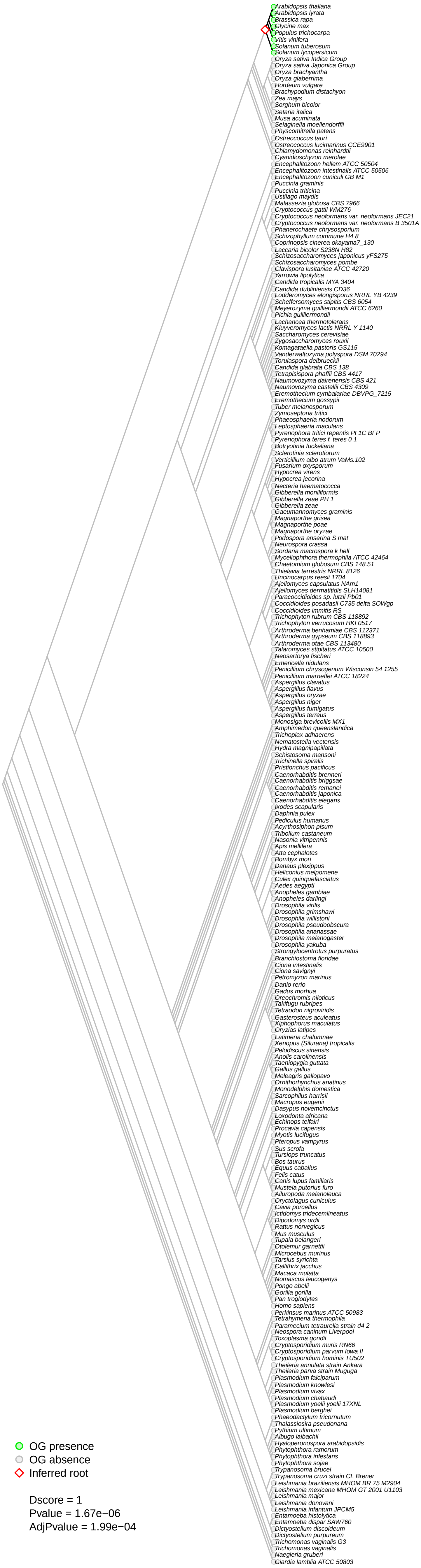

Arabidopsis thaliana
Arabidopsis lyrata
Brassica rapa
Glycine max
Populus trichocarpa
Vitis vinifera
Solanum tuberosum
Solanum lycopersicum
Oryza sativa Indica Group
Oryza sativa Japonica Group
Oryza brachyantha
Oryza glaberrima
Hordeum vulgare
Brachypodium distachyon
Zea mays
Sorghum bicolor
Setaria italica
Musa acuminata
Selaginella moellendorffii
Physcomitrella patens
Ostreococcus tauri
Ostreococcus lucimarinus CCE9901
Chlamydomonas reinhardtii
Cyanidioschyzon merolae
Encephalitozoon hellem ATCC 50504
Encephalitozoon intestinalis ATCC 50506
Encephalitozoon cuniculi GB M1
Puccinia graminis
Puccinia triticina
Ustilago maydis
Malassezia globosa CBS 7966
Cryptococcus gattii WM276
Cryptococcus neoformans var. neoformans JEC21
Cryptococcus neoformans var. neoformans B 3501A
Phanerochaete chrysosporium
Schizophyllum commune H4 8
Coprinopsis cinerea okayama7_130
Laccaria bicolor S238N H82
Schizosaccharomyces japonicus yFS275
Schizosaccharomyces pombe
Clavispora lusitaniae ATCC 42720
Yarrowia lipolytica
Candida tropicalis MYA 3404
Candida dubliniensis CD36
Lodderomyces elongisporus NRRL YB 4239
Scheffersomyces stipitis CBS 6054
Meyerozyma guilliermondii ATCC 6260
Pichia guilliermondii
Lachancea thermotolerans
Kluyveromyces lactis NRRL Y 1140
Saccharomyces cerevisiae
Zygosaccharomyces rouxii
Komagataella pastoris GS115
Vanderwaltozyma polyspora DSM 70294
Torulaspora delbrueckii
Candida glabrata CBS 138
Tetrapisispora phaffii CBS 4417
Naumovozyma dairenensis CBS 421
Naumovozyma castellii CBS 4309
Eremothecium cymbalariae DBVPG_7215
Eremothecium gossypii
Tuber melanosporum
Zymoseptoria tritici
Phaeosphaeria nodorum
Leptosphaeria maculans
Pyrenophora tritici repentis Pt 1C BFP
Pyrenophora teres f. teres 0 1
Botryotinia fuckeliana
Sclerotinia sclerotiorum
Verticillium albo atrum VaMs.102
Fusarium oxysporum
Hypocrea virens
Hypocrea jecorina
Nectria haematococca
Gibberella moniliformis
Gibberella zeae PH 1
Gibberella zeae
Gaeumannomyces graminis
Magnaporthe grisea
Magnaporthe poae
Magnaporthe oryzae
Podospora anserina S mat
Neurospora crassa
Sordaria macrospora k hell
Myceliophthora thermophila ATCC 42464
Chaetomium globosum CBS 148.51
Thielavia terrestris NRRL 8126
Uncinocarpus reesii 1704
Ajellomyces capsulatus NAm1
Ajellomyces dermatitidis SLH14081
Paracoccidioides sp. lutzii Pb01
Coccidioides posadasii C735 delta SOWgp
Coccidioides immitis RS
Trichophyton rubrum CBS 118892
Trichophyton verrucosum HKI 0517
Arthroderma benhamiae CBS 112371
Arthroderma gypseum CBS 118893
Arthroderma otae CBS 113480
Talaromyces stipitatus ATCC 10500
Neosartorya fischeri
Emericella nidulans
Penicillium chrysogenum Wisconsin 54 1255
Penicillium marneffei ATCC 18224
Aspergillus clavatus
Aspergillus flavus
Aspergillus oryzae
Aspergillus niger
Aspergillus fumigatus
Aspergillus terreus
Monosiga brevicollis MX1
Amphimedon queenslandica
Trichoplax adhaerens
Nematostella vectensis
Hydra magnipapillata
Schistosoma mansoni
Trichinella spiralis
Pristionchus pacificus
Caenorhabditis brenneri
Caenorhabditis briggsae
Caenorhabditis remanei
Caenorhabditis japonica
Caenorhabditis elegans
Ixodes scapularis
Daphnia pulex
Pediculus humanus
Acyrthosiphon pisum
Tribolium castaneum
Nasonia vitripennis
Apis mellifera
Atta cephalotes
Bombyx mori
Danaus plexippus
Heliconius melpomene
Culex quinquefasciatus
Aedes aegypti
Anopheles gambiae
Anopheles darlingi
Drosophila virilis
Drosophila grimshawi
Drosophila willistoni
Drosophila pseudoobscura
Drosophila ananassae
Drosophila melanogaster
Drosophila yakuba
Strongylocentrotus purpuratus
Branchiostoma floridae
Ciona intestinalis
Ciona savignyi
Petromyzon marinus
Danio rerio
Gadus morhua
Oreochromis niloticus
Takifugu rubripes
Tetraodon nigroviridis
Gasterosteus aculeatus
Xiphophorus maculatus
Oryzias latipes
Latimeria chalumnae
Xenopus (Silurana) tropicalis
Pelodiscus sinensis
Anolis carolinensis
Taeniopygia guttata
Gallus gallus
Meleagris gallopavo
Ornithorhynchus anatinus
Monodelphis domestica
Sarcophilus harrisii
Macropus eugenii
Dasypus novemcinctus
Loxodonta africana
Echinops telfairi
Procavia capensis
Myotis lucifugus
Pteropus vampyrus
Sus scrofa
Tursiops truncatus
Bos taurus
Equus caballus
Felis catus
Canis lupus familiaris
Mustela putorius furo
Ailuropoda melanoleuca
Oryctolagus cuniculus
Cavia porcellus
Ictidomys tridecemlineatus
Dipodomys ordii
Rattus norvegicus
Mus musculus
Tupaia belangeri
Otolemur garnettii
Microcebus murinus
Tarsius syrichta
Callithrix jacchus
Macaca mulatta
Nomascus leucogenys
Pongo abelii
Gorilla gorilla
Pan troglodytes
Homo sapiens
Perkinsus marinus ATCC 50983
Tetrahymena thermophila
Paramecium tetraurelia strain d4 2
Neospora caninum Liverpool
Toxoplasma gondii
Cryptosporidium muris RN66
Cryptosporidium parvum Iowa II
Cryptosporidium hominis TU502
Theileria annulata strain Ankara
Theileria parva strain Muguga
Plasmodium falciparum
Plasmodium knowlesi
Plasmodium vivax
Plasmodium chabaudi
Plasmodium yoelii yoelii 17XNL
Plasmodium berghei
Phaeodactylum tricornutum
Thalassiosira pseudonana
Pythium ultimum
Albugo laibachii
Hyaloperonospora arabidopsidis
Phytophthora ramorum
Phytophthora infestans
Phytophthora sojae
Trypanosoma brucei
Trypanosoma cruzi strain CL Brener
Leishmania braziliensis MHOM BR 75 M2904
Leishmania mexicana MHOM GT 2001 U1103
Leishmania major
Leishmania donovani
Leishmania infantum JPCM5
Entamoeba histolytica
Entamoeba dispar SAW760
Dictyostelium discoideum
Dictyostelium purpureum
Trichomonas vaginalis G3
Trichomonas vaginalis
Naegleria gruberi
Giardia lamblia ATCC 50803
OG presence
OG absence
Inferred root
Dscore = 1
Pvalue = 1.67e−06
AdjPvalue = 1.99e−04

NOG276879

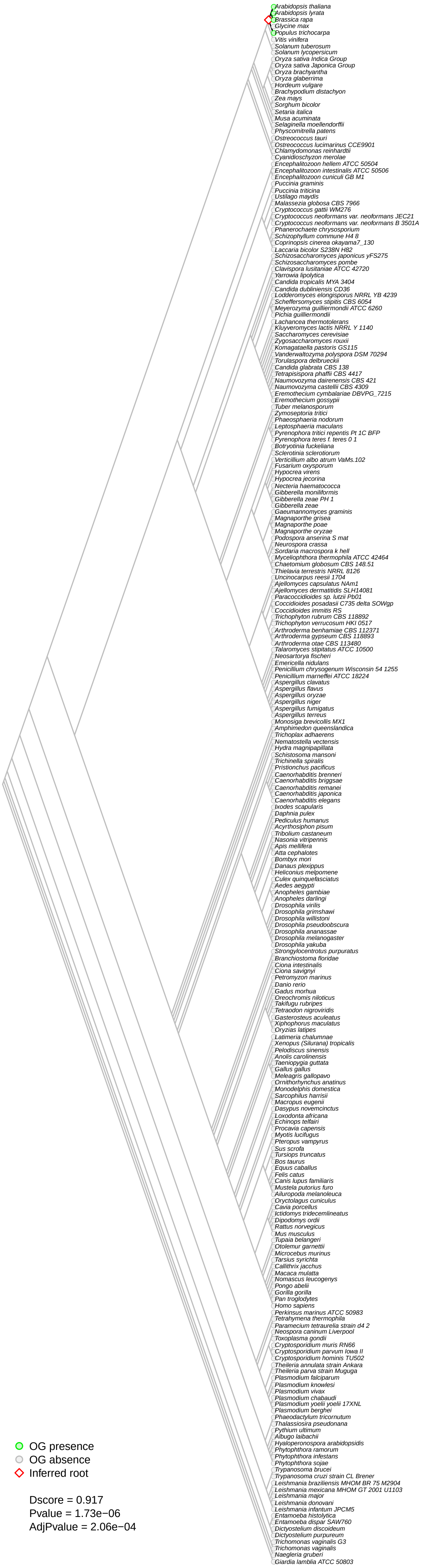

Dscore = 0.917
Pvalue = 1.73e−06
AdjPvalue = 2.06e−04

NOG277769

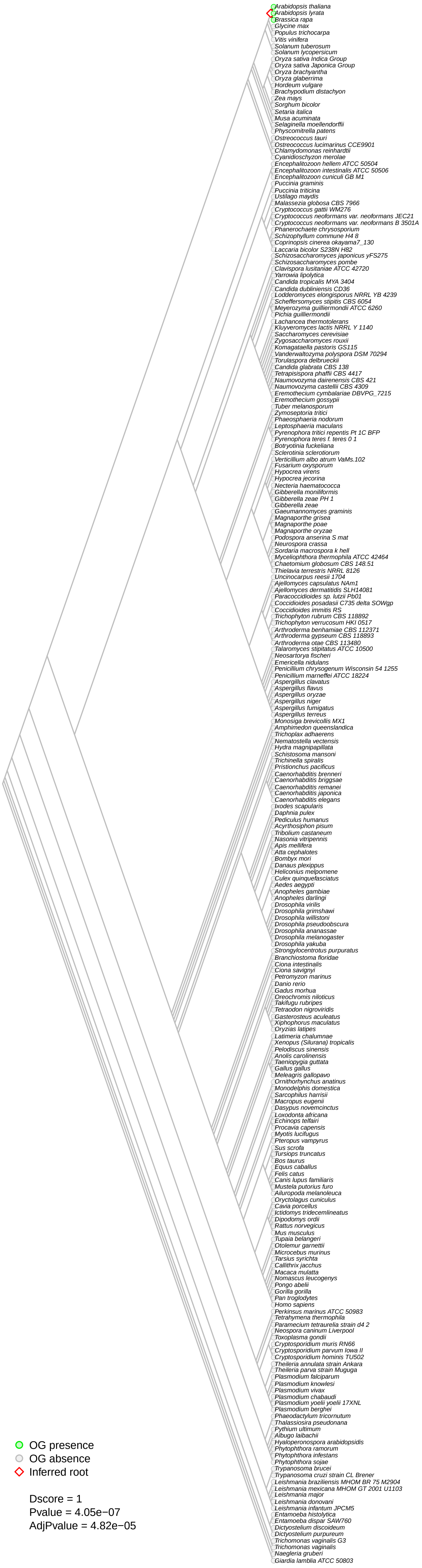

Arabidopsis thaliana
Arabidopsis lyrata
Brassica rapa
Glycine max
Populus trichocarpa
Vitis vinifera
Solanum tuberosum
Solanum lycopersicum
Oryza sativa Indica Group
Oryza sativa Japonica Group
Oryza brachyantha
Oryza glaberrima
Hordeum vulgare
Brachypodium distachyon
Zea mays
Sorghum bicolor
Setaria italica
Musa acuminata
Selaginella moellendorffii
Physcomitrella patens
Ostreococcus tauri
Ostreococcus lucimarinus CCE9901
Chlamydomonas reinhardtii
Cyanidioschyzon merolae
Encephalitozoon hellem ATCC 50504
Encephalitozoon intestinalis ATCC 50506
Encephalitozoon cuniculi GB M1
Puccinia graminis
Puccinia triticina
Ustilago maydis
Malassezia globosa CBS 7966
Cryptococcus gattii WM276
Cryptococcus neoformans var. neoformans JEC21
Cryptococcus neoformans var. neoformans B 3501A
Phanerochaete chrysosporium
Schizophyllum commune H4 8
Coprinopsis cinerea okayama7_130
Laccaria bicolor S238N H82
Schizosaccharomyces japonicus yFS275
Schizosaccharomyces pombe
Clavispora lusitaniae ATCC 42720
Yarrowia lipolytica
Candida tropicalis MYA 3404
Candida dubliniensis CD36
Lodderomyces elongisporus NRRL YB 4239
Scheffersomyces stipitis CBS 6054
Meyerozyma guilliermondii ATCC 6260
Pichia guilliermondii
Lachancea thermotolerans
Kluyveromyces lactis NRRL Y 1140
Saccharomyces cerevisiae
Zygosaccharomyces rouxii
Komagataella pastoris GS115
Vanderwaltozyma polyspora DSM 70294
Torulaspora delbrueckii
Candida glabrata CBS 138
Tetrapisispora phaffii CBS 4417
Naumovozyma dairenensis CBS 421
Naumovozyma castellii CBS 4309
Eremothecium cymbalariae DBVPG_7215
Eremothecium gossypii
Tuber melanosporum
Zymoseptoria tritici
Phaeosphaeria nodorum
Leptosphaeria maculans
Pyrenophora tritici repentis Pt 1C BFP
Pyrenophora teres f. teres 0 1
Botryotinia fuckeliana
Sclerotinia sclerotiorum
Verticillium albo atrum VaMs.102
Fusarium oxysporum
Hypocrea virens
Hypocrea jecorina
Nectria haematococca
Gibberella moniliformis
Gibberella zeae PH 1
Gibberella zeae
Gaeumannomyces graminis
Magnaporthe grisea
Magnaporthe poae
Magnaporthe oryzae
Podospora anserina S mat
Neurospora crassa
Sordaria macrospora k hell
Myceliophthora thermophila ATCC 42464
Chaetomium globosum CBS 148.51
Thielavia terrestris NRRL 8126
Uncinocarpus reesii 1704
Ajellomyces capsulatus NAm1
Ajellomyces dermatitidis SLH14081
Paracoccidioides sp. lutzii Pb01
Coccidioides posadasii C735 delta SOWgp
Coccidioides immitis RS
Trichophyton rubrum CBS 118892
Trichophyton verrucosum HKI 0517
Arthroderma benhamiae CBS 112371
Arthroderma gypseum CBS 118893
Arthroderma otae CBS 113480
Talaromyces stipitatus ATCC 10500
Neosartorya fischeri
Emericella nidulans
Penicillium chrysogenum Wisconsin 54 1255
Penicillium marneffei ATCC 18224
Aspergillus clavatus
Aspergillus flavus
Aspergillus oryzae
Aspergillus niger
Aspergillus fumigatus
Aspergillus terreus
Monosiga brevicollis MX1
Amphimedon queenslandica
Trichoplax adhaerens
Nematostella vectensis
Hydra magnipapillata
Schistosoma mansoni
Trichinella spiralis
Pristionchus pacificus
Caenorhabditis brenneri
Caenorhabditis briggsae
Caenorhabditis remanei
Caenorhabditis japonica
Caenorhabditis elegans
Ixodes scapularis
Daphnia pulex
Pediculus humanus
Acyrthosiphon pisum
Tribolium castaneum
Nasonia vitripennis
Apis mellifera
Atta cephalotes
Bombyx mori
Danaus plexippus
Heliconius melpomene
Culex quinquefasciatus
Aedes aegypti
Anopheles gambiae
Anopheles darlingi
Drosophila virilis
Drosophila grimshawi
Drosophila willistoni
Drosophila pseudoobscura
Drosophila ananassae
Drosophila melanogaster
Drosophila yakuba
Strongylocentrotus purpuratus
Branchiostoma floridae
Ciona intestinalis
Ciona savignyi
Petromyzon marinus
Danio rerio
Gadus morhua
Oreochromis niloticus
Takifugu rubripes
Tetraodon nigroviridis
Gasterosteus aculeatus
Xiphophorus maculatus
Oryzias latipes
Latimeria chalumnae
Xenopus (Silurana) tropicalis
Pelodiscus sinensis
Anolis carolinensis
Taeniopygia guttata
Gallus gallus
Meleagris gallopavo
Ornithorhynchus anatinus
Monodelphis domestica
Sarcophilus harrisii
Macropus eugenii
Dasypus novemcinctus
Loxodonta africana
Echinops telfairi
Procavia capensis
Myotis lucifugus
Pteropus vampyrus
Sus scrofa
Tursiops truncatus
Bos taurus
Equus caballus
Felis catus
Canis lupus familiaris
Mustela putorius furo
Ailuropoda melanoleuca
Oryctolagus cuniculus
Cavia porcellus
Ictidomys tridecemlineatus
Dipodomys ordii
Rattus norvegicus
Mus musculus
Tupaia belangeri
Otolemur garnettii
Microcebus murinus
Tarsius syrichta
Callithrix jacchus
Macaca mulatta
Nomascus leucogenys
Pongo abelii
Gorilla gorilla
Pan troglodytes
Homo sapiens
Perkinsus marinus ATCC 50983
Tetrahymena thermophila
Paramecium tetraurelia strain d4 2
Neospora caninum Liverpool
Toxoplasma gondii
Cryptosporidium muris RN66
Cryptosporidium parvum Iowa II
Cryptosporidium hominis TU502
Theileria annulata strain Ankara
Theileria parva strain Muguga
Plasmodium falciparum
Plasmodium knowlesi
Plasmodium vivax
Plasmodium chabaudi
Plasmodium yoelii yoelii 17XNL
Plasmodium berghei
Phaeodactylum tricornutum
Thalassiosira pseudonana
Pythium ultimum
Albugo laibachii
Hyaloperonospora arabidopsidis
Phytophthora ramorum
Phytophthora infestans
Phytophthora sojae
Trypanosoma brucei
Trypanosoma cruzi strain CL Brener
Leishmania braziliensis MHOM BR 75 M2904
Leishmania mexicana MHOM GT 2001 U1103
Leishmania major
Leishmania donovani
Leishmania infantum JPCM5
Entamoeba histolytica
Entamoeba dispar SAW760
Dictyostelium discoideum
Dictyostelium purpureum
Trichomonas vaginalis G3
Trichomonas vaginalis
Naegleria gruberi
Giardia lamblia ATCC 50803
OG presence
OG absence
Inferred root
Dscore = 1
Pvalue = 4.05e−07
AdjPvalue = 4.82e−05

NOG29116

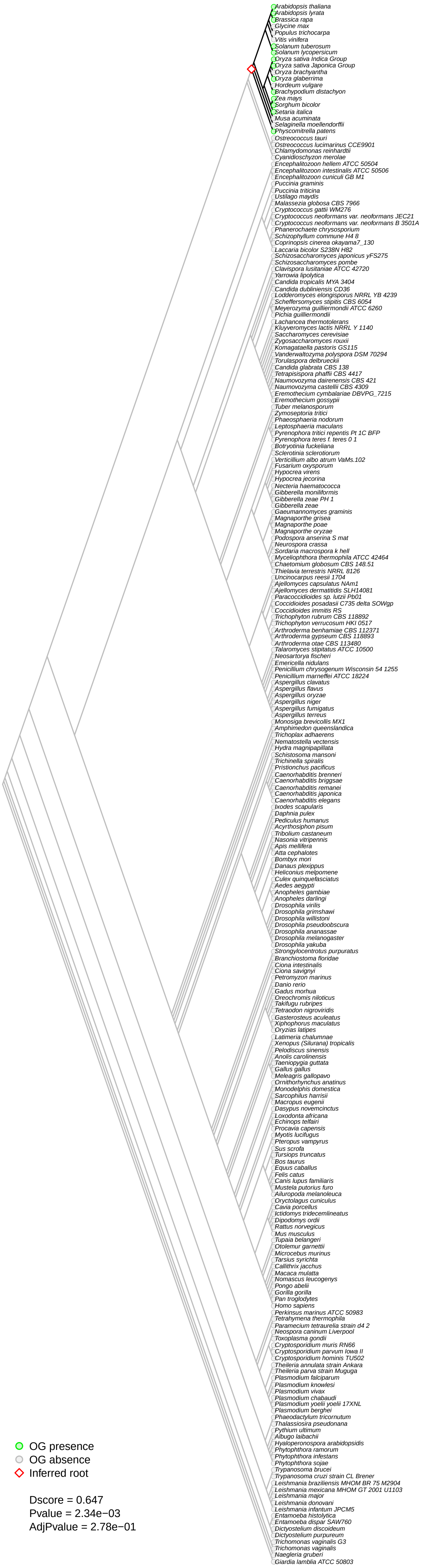

Arabidopsis thaliana
Arabidopsis lyrata
Brassica rapa
Glycine max
Populus trichocarpa
Vitis vinifera
Solanum tuberosum
Solanum lycopersicum
Oryza sativa Indica Group
Oryza sativa Japonica Group
Oryza brachyantha
Oryza glaberrima
Hordeum vulgare
Brachypodium distachyon
Zea mays
Sorghum bicolor
Setaria italica
Musa acuminata
Selaginella moellendorffii
Physcomitrella patens
Ostreococcus tauri
Ostreococcus lucimarinus CCE9901
Chlamydomonas reinhardtii
Cyanidioschyzon merolae
Encephalitozoon hellem ATCC 50504
Encephalitozoon intestinalis ATCC 50506
Encephalitozoon cuniculi GB M1
Puccinia graminis
Puccinia triticina
Ustilago maydis
Malassezia globosa CBS 7966
Cryptococcus gattii WM276
Cryptococcus neoformans var. neoformans JEC21
Cryptococcus neoformans var. neoformans B 3501A
Phanerochaete chrysosporium
Schizophyllum commune H4 8
Coprinopsis cinerea okayama7_130
Laccaria bicolor S238N H82
Schizosaccharomyces japonicus yFS275
Schizosaccharomyces pombe
Clavispora lusitaniae ATCC 42720
Yarrowia lipolytica
Candida tropicalis MYA 3404
Candida dubliniensis CD36
Lodderomyces elongisporus NRRL YB 4239
Scheffersomyces stipitis CBS 6054
Meyerozyma guilliermondii ATCC 6260
Pichia guilliermondii
Lachancea thermotolerans
Kluyveromyces lactis NRRL Y 1140
Saccharomyces cerevisiae
Zygosaccharomyces rouxii
Komagataella pastoris GS115
Vanderwaltozyma polyspora DSM 70294
Torulaspora delbrueckii
Candida glabrata CBS 138
Tetrapisispora phaffii CBS 4417
Naumovozyma dairenensis CBS 421
Naumovozyma castellii CBS 4309
Eremothecium cymbalariae DBVPG_7215
Eremothecium gossypii
Tuber melanosporum
Zymoseptoria tritici
Phaeosphaeria nodorum
Leptosphaeria maculans
Pyrenophora tritici repentis Pt 1C BFP
Pyrenophora teres f. teres 0 1
Botryotinia fuckeliana
Sclerotinia sclerotiorum
Verticillium albo atrum VaMs.102
Fusarium oxysporum
Hypocrea virens
Hypocrea jecorina
Nectria haematococca
Gibberella moniliformis
Gibberella zeae PH 1
Gibberella zeae
Gaeumannomyces graminis
Magnaporthe grisea
Magnaporthe poae
Magnaporthe oryzae
Podospora anserina S mat
Neurospora crassa
Sordaria macrospora k hell
Myceliophthora thermophila ATCC 42464
Chaetomium globosum CBS 148.51
Thielavia terrestris NRRL 8126
Uncinocarpus reesii 1704
Ajellomyces capsulatus NAm1
Ajellomyces dermatitidis SLH14081
Paracoccidioides sp. lutzii Pb01
Coccidioides posadasii C735 delta SOWgp
Coccidioides immitis RS
Trichophyton rubrum CBS 118892
Trichophyton verrucosum HKI 0517
Arthroderma benhamiae CBS 112371
Arthroderma gypseum CBS 118893
Arthroderma otae CBS 113480
Talaromyces stipitatus ATCC 10500
Neosartorya fischeri
Emericella nidulans
Penicillium chrysogenum Wisconsin 54 1255
Penicillium marneffei ATCC 18224
Aspergillus clavatus
Aspergillus flavus
Aspergillus oryzae
Aspergillus niger
Aspergillus fumigatus
Aspergillus terreus
Monosiga brevicollis MX1
Amphimedon queenslandica
Trichoplax adhaerens
Nematostella vectensis
Hydra magnipapillata
Schistosoma mansoni
Trichinella spiralis
Pristionchus pacificus
Caenorhabditis brenneri
Caenorhabditis briggsae
Caenorhabditis remanei
Caenorhabditis japonica
Caenorhabditis elegans
Ixodes scapularis
Daphnia pulex
Pediculus humanus
Acyrthosiphon pisum
Tribolium castaneum
Nasonia vitripennis
Apis mellifera
Atta cephalotes
Bombyx mori
Danaus plexippus
Heliconius melpomene
Culex quinquefasciatus
Aedes aegypti
Anopheles gambiae
Anopheles darlingi
Drosophila virilis
Drosophila grimshawi
Drosophila willistoni
Drosophila pseudoobscura
Drosophila ananassae
Drosophila melanogaster
Drosophila yakuba
Strongylocentrotus purpuratus
Branchiostoma floridae
Ciona intestinalis
Ciona savignyi
Petromyzon marinus
Danio rerio
Gadus morhua
Oreochromis niloticus
Takifugu rubripes
Tetraodon nigroviridis
Gasterosteus aculeatus
Xiphophorus maculatus
Oryzias latipes
Latimeria chalumnae
Xenopus (Silurana) tropicalis
Pelodiscus sinensis
Anolis carolinensis
Taeniopygia guttata
Gallus gallus
Meleagris gallopavo
Ornithorhynchus anatinus
Monodelphis domestica
Sarcophilus harrisii
Macropus eugenii
Dasypus novemcinctus
Loxodonta africana
Echinops telfairi
Procavia capensis
Myotis lucifugus
Pteropus vampyrus
Sus scrofa
Tursiops truncatus
Bos taurus
Equus caballus
Felis catus
Canis lupus familiaris
Mustela putorius furo
Ailuropoda melanoleuca
Oryctolagus cuniculus
Cavia porcellus
Ictidomys tridecemlineatus
Dipodomys ordii
Rattus norvegicus
Mus musculus
Tupaia belangeri
Otolemur garnettii
Microcebus murinus
Tarsius syrichta
Callithrix jacchus
Macaca mulatta
Nomascus leucogenys
Pongo abelii
Gorilla gorilla
Pan troglodytes
Homo sapiens
Perkinsus marinus ATCC 50983
Tetrahymena thermophila
Paramecium tetraurelia strain d4 2
Neospora caninum Liverpool
Toxoplasma gondii
Cryptosporidium muris RN66
Cryptosporidium parvum Iowa II
Cryptosporidium hominis TU502
Theileria annulata strain Ankara
Theileria parva strain Muguga
Plasmodium falciparum
Plasmodium knowlesi
Plasmodium vivax
Plasmodium chabaudi
Plasmodium yoelii yoelii 17XNL
Plasmodium berghei
Phaeodactylum tricornutum
Thalassiosira pseudonana
Pythium ultimum
Albugo laibachii
Hyaloperonospora arabidopsidis
Phytophthora ramorum
Phytophthora infestans
Phytophthora sojae
Trypanosoma brucei
Trypanosoma cruzi strain CL Brener
Leishmania braziliensis MHOM BR 75 M2904
Leishmania mexicana MHOM GT 2001 U1103
Leishmania major
Leishmania donovani
Leishmania infantum JPCM5
Entamoeba histolytica
Entamoeba dispar SAW760
Dictyostelium discoideum
Dictyostelium purpureum
Trichomonas vaginalis G3
Trichomonas vaginalis
Naegleria gruberi
Giardia lamblia ATCC 50803
OG presence
OG absence
Inferred root
Dscore = 0.647
Pvalue = 2.34e−03
AdjPvalue = 2.78e−01

NOG29576

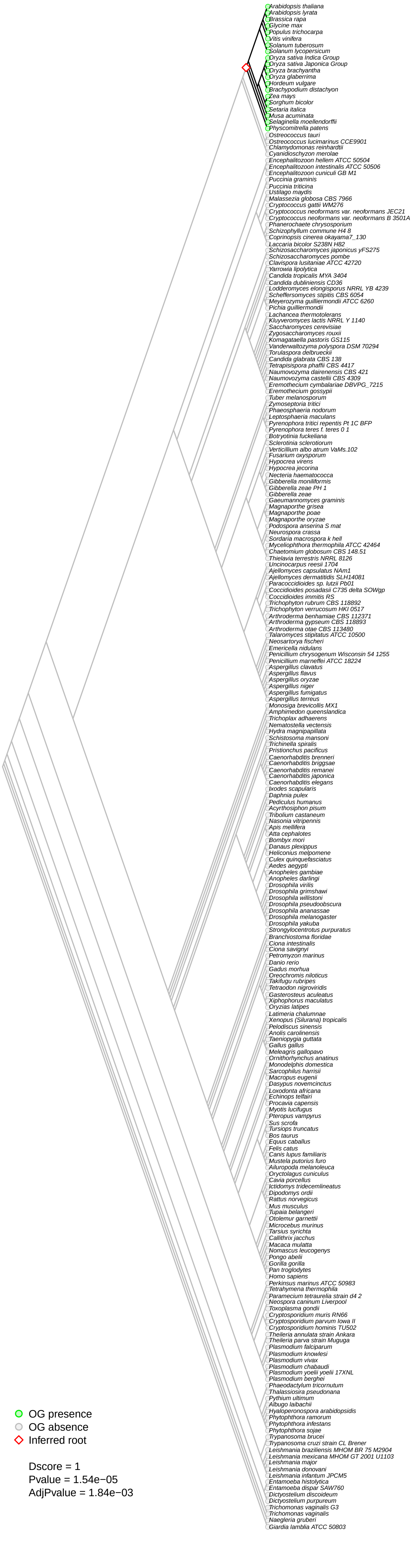
Arabidopsis thaliana
Arabidopsis lyrata
Brassica rapa
Glycine max
Populus trichocarpa
Vitis vinifera
Solanum tuberosum
Solanum lycopersicum
Oryza sativa Indica Group
Oryza sativa Japonica Group
Oryza brachyantha
Oryza glaberrima
Hordeum vulgare
Brachypodium distachyon
Zea mays
Sorghum bicolor
Setaria italica
Musa acuminata
Selaginella moellendorffii
Physcomitrella patens
Ostreococcus tauri
Ostreococcus lucimarinus CCE9901
Chlamydomonas reinhardtii
Cyanidioschyzon merolae
Encephalitozoon hellem ATCC 50504
Encephalitozoon intestinalis ATCC 50506
Encephalitozoon cuniculi GB M1
Puccinia graminis
Puccinia triticina
Ustilago maydis
Malassezia globosa CBS 7966
Cryptococcus gattii WM276
Cryptococcus neoformans var. neoformans JEC21
Cryptococcus neoformans var. neoformans B 3501A
Phanerochaete chrysosporium
Schizophyllum commune H4 8
Coprinopsis cinerea okayama7_130
Laccaria bicolor S238N H82
Schizosaccharomyces japonicus yFS275
Schizosaccharomyces pombe
Clavispora lusitaniae ATCC 42720
Yarrowia lipolytica
Candida tropicalis MYA 3404
Candida dubliniensis CD36
Lodderomyces elongisporus NRRL YB 4239
Scheffersomyces stipitis CBS 6054
Meyerozyma guilliermondii ATCC 6260
Pichia guilliermondii
Lachancea thermotolerans
Kluyveromyces lactis NRRL Y 1140
Saccharomyces cerevisiae
Zygosaccharomyces rouxii
Komagataella pastoris GS115
Vanderwaltozyma polyspora DSM 70294
Torulaspora delbrueckii
Candida glabrata CBS 138
Tetrapisispora phaffii CBS 4417
Naumovozyma dairenensis CBS 421
Naumovozyma castellii CBS 4309
Eremothecium cymbalariae DBVPG_7215
Eremothecium gossypii
Tuber melanosporum
Zymoseptoria tritici
Phaeosphaeria nodorum
Leptosphaeria maculans
Pyrenophora tritici repentis Pt 1C BFP
Pyrenophora teres f. teres 0 1
Botryotinia fuckeliana
Sclerotinia sclerotiorum
Verticillium albo atrum VaMs.102
Fusarium oxysporum
Hypocrea virens
Hypocrea jecorina
Nectria haematococca
Gibberella moniliformis
Gibberella zeae PH 1
Gibberella zeae
Gaeumannomyces graminis
Magnaporthe grisea
Magnaporthe poae
Magnaporthe oryzae
Podospora anserina S mat
Neurospora crassa
Sordaria macrospora k hell
Myceliophthora thermophila ATCC 42464
Chaetomium globosum CBS 148.51
Thielavia terrestris NRRL 8126
Uncinocarpus reesii 1704
Ajellomyces capsulatus NAm1
Ajellomyces dermatitidis SLH14081
Paracoccidioides sp. lutzii Pb01
Coccidioides posadasii C735 delta SOWgp
Coccidioides immitis RS
Trichophyton rubrum CBS 118892
Trichophyton verrucosum HKI 0517
Arthroderma benhamiae CBS 112371
Arthroderma gypseum CBS 118893
Arthroderma otae CBS 113480
Talaromyces stipitatus ATCC 10500
Neosartorya fischeri
Emericella nidulans
Penicillium chrysogenum Wisconsin 54 1255
Penicillium marneffei ATCC 18224
Aspergillus clavatus
Aspergillus flavus
Aspergillus oryzae
Aspergillus niger
Aspergillus fumigatus
Aspergillus terreus
Monosiga brevicollis MX1
Amphimedon queenslandica
Trichoplax adhaerens
Nematostella vectensis
Hydra magnipapillata
Schistosoma mansoni
Trichinella spiralis
Pristionchus pacificus
Caenorhabditis brenneri
Caenorhabditis briggsae
Caenorhabditis remanei
Caenorhabditis japonica
Caenorhabditis elegans
Ixodes scapularis
Daphnia pulex
Pediculus humanus
Acyrthosiphon pisum
Tribolium castaneum
Nasonia vitripennis
Apis mellifera
Atta cephalotes
Bombyx mori
Danaus plexippus
Heliconius melpomene
Culex quinquefasciatus
Aedes aegypti
Anopheles gambiae
Anopheles darlingi
Drosophila virilis
Drosophila grimshawi
Drosophila willistoni
Drosophila pseudoobscura
Drosophila ananassae
Drosophila melanogaster
Drosophila yakuba
Strongylocentrotus purpuratus
Branchiostoma floridae
Ciona intestinalis
Ciona savignyi
Petromyzon marinus
Danio rerio
Gadus morhua
Oreochromis niloticus
Takifugu rubripes
Tetraodon nigroviridis
Gasterosteus aculeatus
Xiphophorus maculatus
Oryzias latipes
Latimeria chalumnae
Xenopus (Silurana) tropicalis
Pelodiscus sinensis
Anolis carolinensis
Taeniopygia guttata
Gallus gallus
Meleagris gallopavo
Ornithorhynchus anatinus
Monodelphis domestica
Sarcophilus harrisii
Macropus eugenii
Dasypus novemcinctus
Loxodonta africana
Echinops telfairi
Procavia capensis
Myotis lucifugus
Pteropus vampyrus
Sus scrofa
Tursiops truncatus
Bos taurus
Equus caballus
Felis catus
Canis lupus familiaris
Mustela putorius furo
Ailuropoda melanoleuca
Oryctolagus cuniculus
Cavia porcellus
Ictidomys tridecemlineatus
Dipodomys ordii
Rattus norvegicus
Mus musculus
Tupaia belangeri
Otolemur garnettii
Microcebus murinus
Tarsius syrichta
Callithrix jacchus
Macaca mulatta
Nomascus leucogenys
Pongo abelii
Gorilla gorilla
Pan troglodytes
Homo sapiens
Perkinsus marinus ATCC 50983
Tetrahymena thermophila
Paramecium tetraurelia strain d4 2
Neospora caninum Liverpool
Toxoplasma gondii
Cryptosporidium muris RN66
Cryptosporidium parvum Iowa II
Cryptosporidium hominis TU502
Theileria annulata strain Ankara
Theileria parva strain Muguga
Plasmodium falciparum
Plasmodium knowlesi
Plasmodium vivax
Plasmodium chabaudi
Plasmodium yoelii yoelii 17XNL
Plasmodium berghei
Phaeodactylum tricornutum
Thalassiosira pseudonana
Pythium ultimum
Albugo laibachii
Hyaloperonospora arabidopsidis
Phytophthora ramorum
Phytophthora infestans
Phytophthora sojae
Trypanosoma brucei
Trypanosoma cruzi strain CL Brener
Leishmania braziliensis MHOM BR 75 M2904
Leishmania mexicana MHOM GT 2001 U1103
Leishmania major
Leishmania donovani
Leishmania infantum JPCM5
Entamoeba histolytica
Entamoeba dispar SAW760
Dictyostelium discoideum
Dictyostelium purpureum
Trichomonas vaginalis G3
Trichomonas vaginalis
Naegleria gruberi
Giardia lamblia ATCC 50803
OG presence
OG absence
Inferred root
Dscore = 1
Pvalue = 1.54e−05
AdjPvalue = 1.84e−03

NOG33768

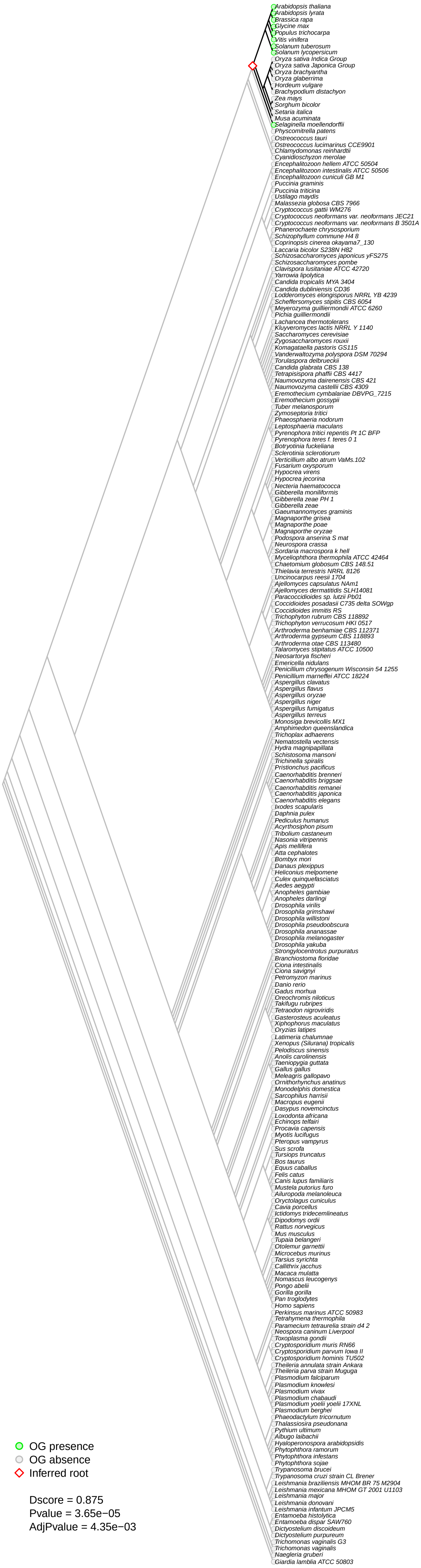

OG presence
OG absence
Inferred root

Dscore = 0.875
Pvalue = 3.65e−05
AdjPvalue = 4.35e−03

NOG35174

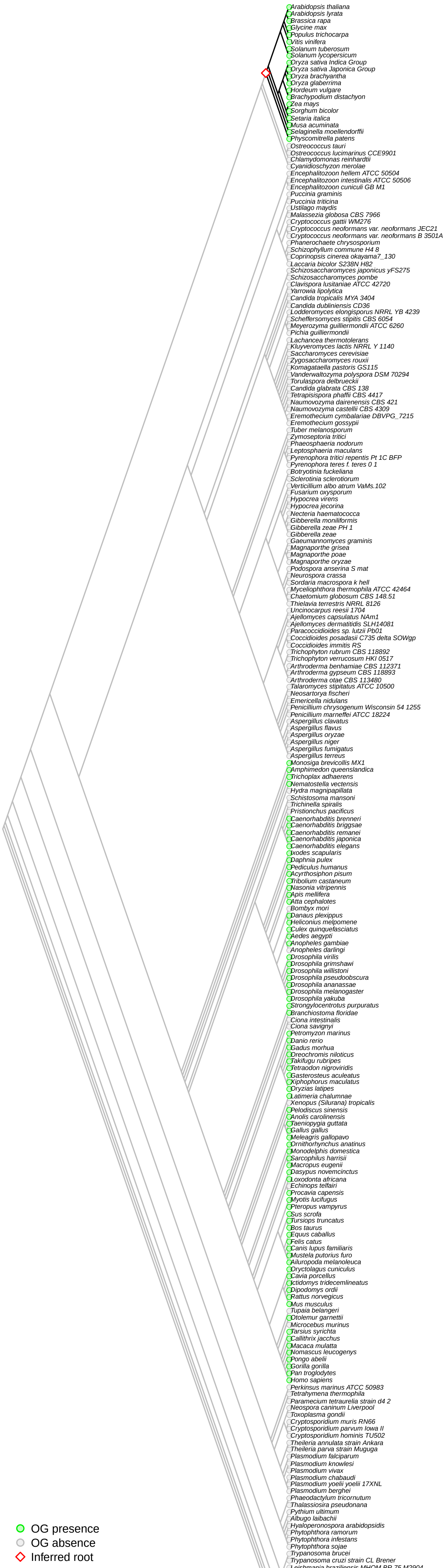

Dscore = 0.944
Pvalue = 1.91e−05
AdjPvalue = 2.27e−03

NOG45847

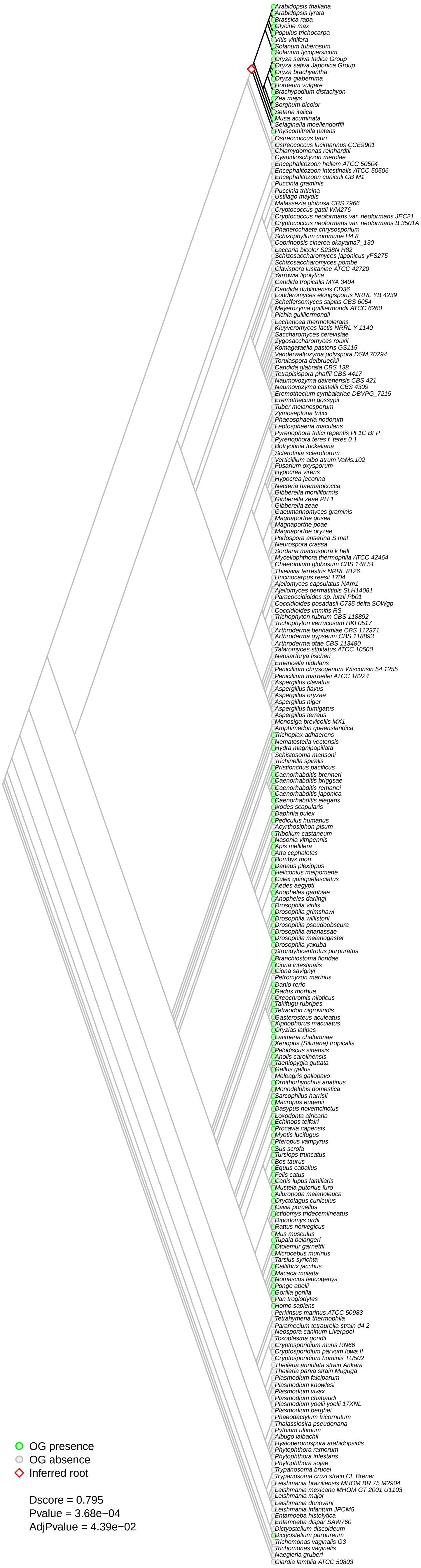
Arabidopsis thaliana
Arabidopsis lyrata
Brassica rapa
Glycine max
Populus trichocarpa
Vitis vinifera
Solanum tuberosum
Solanum lycopersicum
Oryza sativa Indica Group
Oryza sativa Japonica Group
Oryza brachyantha
Oryza glaberrima
Hordeum vulgare
Brachypodium distachyon
Zea mays
Sorghum bicolor
Setaria italica
Musa acuminata
Selaginella moellendorffii
Physcomitrella patens
Ostreococcus tauri
Ostreococcus lucimarinus CCE9901
Chlamydomonas reinhardtii
Cyanidioschyzon merolae
Encephalitozoon hellem ATCC 50504
Encephalitozoon intestinalis ATCC 50506
Encephalitozoon cuniculi GB M1
Puccinia graminis
Puccinia triticina
Ustilago maydis
Malassezia globosa CBS 7966
Cryptococcus gattii WM276
Cryptococcus neoformans var. neoformans JEC21
Cryptococcus neoformans var. neoformans B 3501A
Phanerochaete chrysosporium
Schizophyllum commune H4 8
Coprinopsis cinerea okayama7_130
Laccaria bicolor S238N H82
Schizosaccharomyces japonicus yFS275
Schizosaccharomyces pombe
Clavispora lusitaniae ATCC 42720
Yarrowia lipolytica
Candida tropicalis MYA 3404
Candida dubliniensis CD36
Lodderomyces elongisporus NRRL YB 4239
Scheffersomyces stipitis CBS 6054
Meyerozyma guilliermondii ATCC 6260
Pichia guilliermondii
Lachancea thermotolerans
Kluyveromyces lactis NRRL Y 1140
Saccharomyces cerevisiae
Zygosaccharomyces rouxii
Komagataella pastoris GS115
Vanderwaltozyma polyspora DSM 70294
Torulaspora delbrueckii
Candida glabrata CBS 138
Tetrapisispora phaffii CBS 4417
Naumovozyma dairenensis CBS 421
Naumovozyma castellii CBS 4309
Eremothecium cymbalariae DBVPG_7215
Eremothecium gossypii
Tuber melanosporum
Zymoseptoria tritici
Phaeosphaeria nodorum
Leptosphaeria maculans
Pyrenophora tritici repentis Pt 1C BFP
Pyrenophora teres f. teres 0 1
Botryotinia fuckeliana
Sclerotinia sclerotiorum
Verticillium albo atrum VaMs.102
Fusarium oxysporum
Hypocrea virens
Hypocrea jecorina
Nectria haematococca
Gibberella moniliformis
Gibberella zeae PH 1
Gibberella zeae
Gaeumannomyces graminis
Magnaporthe grisea
Magnaporthe poae
Magnaporthe oryzae
Podospora anserina S mat
Neurospora crassa
Sordaria macrospora k hell
Myceliophthora thermophila ATCC 42464
Chaetomium globosum CBS 148.51
Thielavia terrestris NRRL 8126
Uncinocarpus reesii 1704
Ajellomyces capsulatus NAm1
Ajellomyces dermatitidis SLH14081
Paracoccidioides sp. lutzii Pb01
Coccidioides posadasii C735 delta SOWgp
Coccidioides immitis RS
Trichophyton rubrum CBS 118892
Trichophyton verrucosum HKI 0517
Arthroderma benhamiae CBS 112371
Arthroderma gypseum CBS 118893
Arthroderma otae CBS 113480
Talaromyces stipitatus ATCC 10500
Neosartorya fischeri
Emericella nidulans
Penicillium chrysogenum Wisconsin 54 1255
Penicillium marneffei ATCC 18224
Aspergillus clavatus
Aspergillus flavus
Aspergillus oryzae
Aspergillus niger
Aspergillus fumigatus
Aspergillus terreus
Monosiga brevicollis MX1
Amphimedon queenslandica
Trichoplax adhaerens
Nematostella vectensis
Hydra magnipapillata
Schistosoma mansoni
Trichinella spiralis
Pristionchus pacificus
Caenorhabditis brenneri
Caenorhabditis briggsae
Caenorhabditis remanei
Caenorhabditis japonica
Caenorhabditis elegans
Ixodes scapularis
Daphnia pulex
Pediculus humanus
Acyrthosiphon pisum
Tribolium castaneum
Nasonia vitripennis
Apis mellifera
Atta cephalotes
Bombyx mori
Danaus plexippus
Heliconius melpomene
Culex quinquefasciatus
Aedes aegypti
Anopheles gambiae
Anopheles darlingi
Drosophila virilis
Drosophila grimshawi
Drosophila willistoni
Drosophila pseudoobscura
Drosophila ananassae
Drosophila melanogaster
Drosophila yakuba
Strongylocentrotus purpuratus
Branchiostoma floridae
Ciona intestinalis
Ciona savignyi
Petromyzon marinus
Danio rerio
Gadus morhua
Oreochromis niloticus
Takifugu rubripes
Tetraodon nigroviridis
Gasterosteus aculeatus
Xiphophorus maculatus
Oryzias latipes
Latimeria chalumnae
Xenopus (Silurana) tropicalis
Pelodiscus sinensis
Anolis carolinensis
Taeniopygia guttata
Gallus gallus
Meleagris gallopavo
Ornithorhynchus anatinus
Monodelphis domestica
Sarcophilus harrisii
Macropus eugenii
Dasypus novemcinctus
Loxodonta africana
Echinops telfairi
Procavia capensis
Myotis lucifugus
Pteropus vampyrus
Sus scrofa
Tursiops truncatus
Bos taurus
Equus caballus
Felis catus
Canis lupus familiaris
Mustela putorius furo
Ailuropoda melanoleuca
Oryctolagus cuniculus
Cavia porcellus
Ictidomys tridecemlineatus
Dipodomys ordii
Rattus norvegicus
Mus musculus
Tupaia belangeri
Otolemur garnettii
Microcebus murinus
Tarsius syrichta
Callithrix jacchus
Macaca mulatta
Nomascus leucogenys
Pongo abelii
Gorilla gorilla
Pan troglodytes
Homo sapiens
Perkinsus marinus ATCC 50983
Tetrahymena thermophila
Paramecium tetraurelia strain d4 2
Neospora caninum Liverpool
Toxoplasma gondii
Cryptosporidium muris RN66
Cryptosporidium parvum Iowa II
Cryptosporidium hominis TU502
Theileria annulata strain Ankara
Theileria parva strain Muguga
Plasmodium falciparum
Plasmodium knowlesi
Plasmodium vivax
Plasmodium chabaudi
Plasmodium yoelii yoelii 17XNL
Plasmodium berghei
Phaeodactylum tricornutum
Thalassiosira pseudonana
Pythium ultimum
Albugo laibachii
Hyaloperonospora arabidopsidis
Phytophthora ramorum
Phytophthora infestans
Phytophthora sojae
Trypanosoma brucei
Trypanosoma cruzi strain CL Brener
Leishmania braziliensis MHOM BR 75 M2904
Leishmania mexicana MHOM GT 2001 U1103
Leishmania major
Leishmania donovani
Leishmania infantum JPCM5
Entamoeba histolytica
Entamoeba dispar SAW760
Dictyostelium discoideum
Dictyostelium purpureum
Trichomonas vaginalis G3
Trichomonas vaginalis
Naegleria gruberi
Giardia lamblia ATCC 50803
OG presence
OG absence
Inferred root
Dscore = 0.795
Pvalue = 3.68e−04
AdjPvalue = 4.39e−02

NOG53024

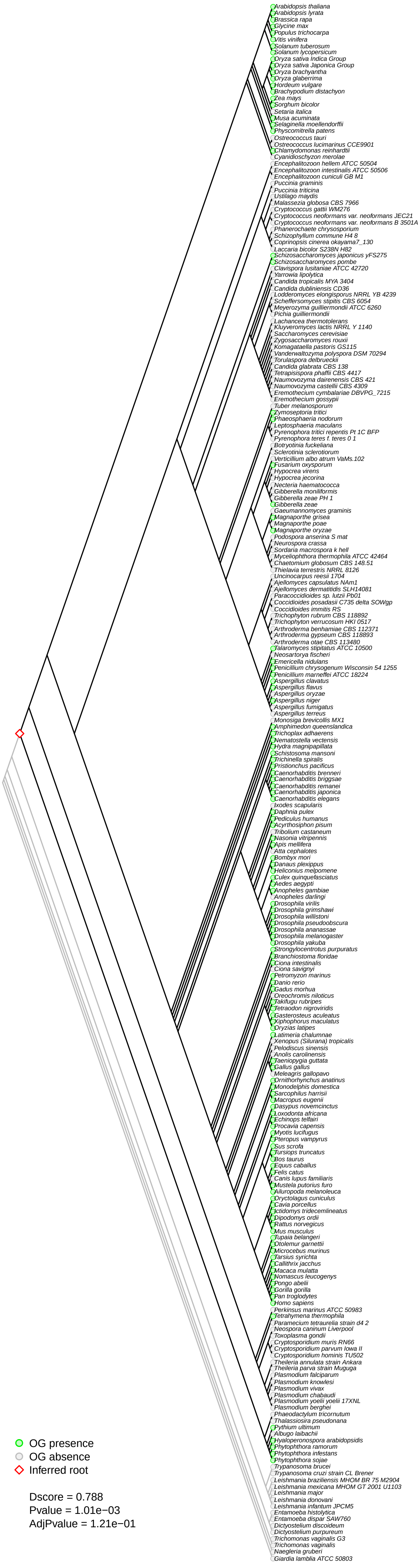

Arabidopsis thaliana
Arabidopsis lyrata
Brassica rapa
Glycine max
Populus trichocarpa
Vitis vinifera
Solanum tuberosum
Solanum lycopersicum
Oryza sativa Indica Group
Oryza sativa Japonica Group
Oryza brachyantha
Oryza glaberrima
Hordeum vulgare
Brachypodium distachyon
Zea mays
Sorghum bicolor
Setaria italica
Musa acuminata
Selaginella moellendorffii
Physcomitrella patens
Ostreococcus tauri
Ostreococcus lucimarinus CCE9901
Chlamydomonas reinhardtii
Cyanidioschyzon merolae
Encephalitozoon hellem ATCC 50504
Encephalitozoon intestinalis ATCC 50506
Encephalitozoon cuniculi GB M1
Puccinia graminis
Puccinia triticina
Ustilago maydis
Malassezia globosa CBS 7966
Cryptococcus gattii WM276
Cryptococcus neoformans var. neoformans JEC21
Cryptococcus neoformans var. neoformans B 3501A
Phanerochaete chrysosporium
Schizophyllum commune H4 8
Coprinopsis cinerea okayama7_130
Laccaria bicolor S238N H82
Schizosaccharomyces japonicus yFS275
Schizosaccharomyces pombe
Clavispora lusitaniae ATCC 42720
Yarrowia lipolytica
Candida tropicalis MYA 3404
Candida dubliniensis CD36
Lodderomyces elongisporus NRRL YB 4239
Scheffersomyces stipitis CBS 6054
Meyerozyma guilliermondii ATCC 6260
Pichia guilliermondii
Lachancea thermotolerans
Kluyveromyces lactis NRRL Y 1140
Saccharomyces cerevisiae
Zygosaccharomyces rouxii
Komagataella pastoris GS115
Vanderwaltozyma polyspora DSM 70294
Torulaspora delbrueckii
Candida glabrata CBS 138
Tetrapisispora phaffii CBS 4417
Naumovozyma dairenensis CBS 421
Naumovozyma castellii CBS 4309
Eremothecium cymbalariae DBVPG_7215
Eremothecium gossypii
Tuber melanosporum
Zymoseptoria tritici
Phaeosphaeria nodorum
Leptosphaeria maculans
Pyrenophora tritici repentis Pt 1C BFP
Pyrenophora teres f. teres 0 1
Botryotinia fuckeliana
Sclerotinia sclerotiorum
Verticillium albo atrum VaMs.102
Fusarium oxysporum
Hypocrea virens
Hypocrea jecorina
Nectria haematococca
Gibberella moniliformis
Gibberella zeae PH 1
Gibberella zeae
Gaeumannomyces graminis
Magnaporthe grisea
Magnaporthe poae
Magnaporthe oryzae
Podospora anserina S mat
Neurospora crassa
Sordaria macrospora k hell
Myceliophthora thermophila ATCC 42464
Chaetomium globosum CBS 148.51
Thielavia terrestris NRRL 8126
Uncinocarpus reesii 1704
Ajellomyces capsulatus NAm1
Ajellomyces dermatitidis SLH14081
Paracoccidioides sp. lutzii Pb01
Coccidioides posadasii C735 delta SOWgp
Coccidioides immitis RS
Trichophyton rubrum CBS 118892
Trichophyton verrucosum HKI 0517
Arthroderma benhamiae CBS 112371
Arthroderma gypseum CBS 118893
Arthroderma otae CBS 113480
Talaromyces stipitatus ATCC 10500
Neosartorya fischeri
Emericella nidulans
Penicillium chrysogenum Wisconsin 54 1255
Penicillium marneffei ATCC 18224
Aspergillus clavatus
Aspergillus flavus
Aspergillus oryzae
Aspergillus niger
Aspergillus fumigatus
Aspergillus terreus
Monosiga brevicollis MX1
Amphimedon queenslandica
Trichoplax adhaerens
Nematostella vectensis
Hydra magnipapillata
Schistosoma mansoni
Trichinella spiralis
Pristionchus pacificus
Caenorhabditis brenneri
Caenorhabditis briggsae
Caenorhabditis remanei
Caenorhabditis japonica
Caenorhabditis elegans
Ixodes scapularis
Daphnia pulex
Pediculus humanus
Acyrthosiphon pisum
Tribolium castaneum
Nasonia vitripennis
Apis mellifera
Atta cephalotes
Bombyx mori
Danaus plexippus
Heliconius melpomene
Culex quinquefasciatus
Aedes aegypti
Anopheles gambiae
Anopheles darlingi
Drosophila virilis
Drosophila grimshawi
Drosophila willistoni
Drosophila pseudoobscura
Drosophila ananassae
Drosophila melanogaster
Drosophila yakuba
Strongylocentrotus purpuratus
Branchiostoma floridae
Ciona intestinalis
Ciona savignyi
Petromyzon marinus
Danio rerio
Gadus morhua
Oreochromis niloticus
Takifugu rubripes
Tetraodon nigroviridis
Gasterosteus aculeatus
Xiphophorus maculatus
Oryzias latipes
Latimeria chalumnae
Xenopus (Silurana) tropicalis
Pelodiscus sinensis
Anolis carolinensis
Taeniopygia guttata
Gallus gallus
Meleagris gallopavo
Ornithorhynchus anatinus
Monodelphis domestica
Sarcophilus harrisii
Macropus eugenii
Dasypus novemcinctus
Loxodonta africana
Echinops telfairi
Procavia capensis
Myotis lucifugus
Pteropus vampyrus
Sus scrofa
Tursiops truncatus
Bos taurus
Equus caballus
Felis catus
Canis lupus familiaris
Mustela putorius furo
Ailuropoda melanoleuca
Oryctolagus cuniculus
Cavia porcellus
Ictidomys tridecemlineatus
Dipodomys ordii
Rattus norvegicus
Mus musculus
Tupaia belangeri
Otolemur garnettii
Microcebus murinus
Tarsius syrichta
Callithrix jacchus
Macaca mulatta
Nomascus leucogenys
Pongo abelii
Gorilla gorilla
Pan troglodytes
Homo sapiens
Perkinsus marinus ATCC 50983
Tetrahymena thermophila
Paramecium tetraurelia strain d4 2
Neospora caninum Liverpool
Toxoplasma gondii
Cryptosporidium muris RN66
Cryptosporidium parvum Iowa II
Cryptosporidium hominis TU502
Theileria annulata strain Ankara
Theileria parva strain Muguga
Plasmodium falciparum
Plasmodium knowlesi
Plasmodium vivax
Plasmodium chabaudi
Plasmodium yoelii yoelii 17XNL
Plasmodium berghei
Phaeodactylum tricornutum
Thalassiosira pseudonana
Pythium ultimum
Albugo laibachii
Hyaloperonospora arabidopsidis
Phytophthora ramorum
Phytophthora infestans
Phytophthora sojae
Trypanosoma brucei
Trypanosoma cruzi strain CL Brener
Leishmania braziliensis MHOM BR 75 M2904
Leishmania mexicana MHOM GT 2001 U1103
Leishmania major
Leishmania donovani
Leishmania infantum JPCM5
Entamoeba histolytica
Entamoeba dispar SAW760
Dictyostelium discoideum
Dictyostelium purpureum
Trichomonas vaginalis G3
Trichomonas vaginalis
Naegleria gruberi
Giardia lamblia ATCC 50803
OG presence
OG absence
Inferred root
Dscore = 0.788
Pvalue = 1.01e−03
AdjPvalue = 1.21e−01

NOG58286

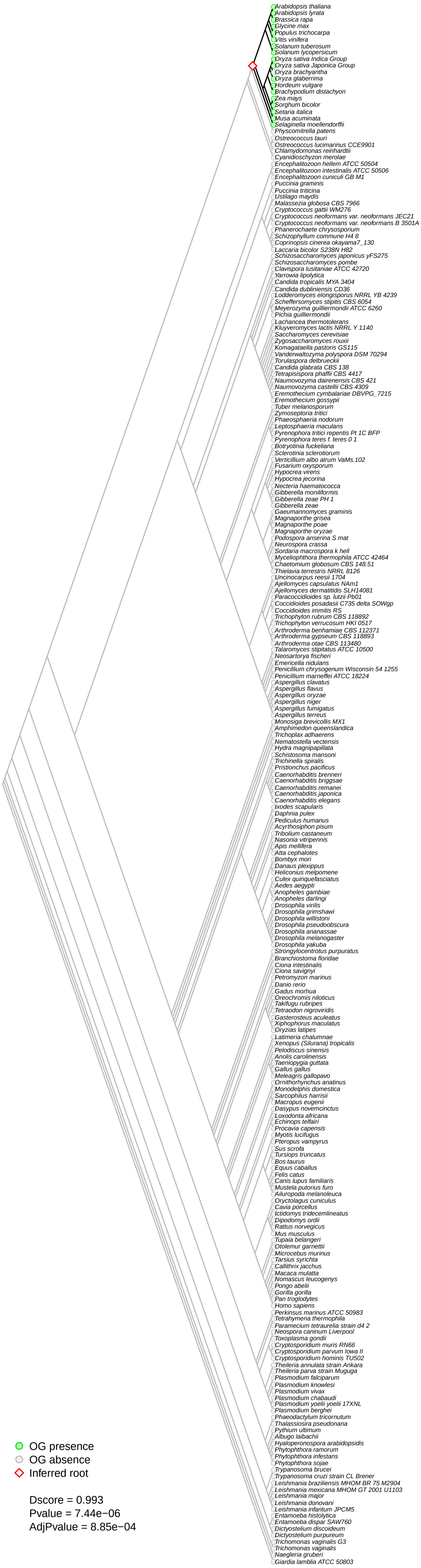
Arabidopsis thaliana
Arabidopsis lyrata
Brassica rapa
Glycine max
Populus trichocarpa
Vitis vinifera
Solanum tuberosum
Solanum lycopersicum
Oryza sativa Indica Group
Oryza sativa Japonica Group
Oryza brachyantha
Oryza glaberrima
Hordeum vulgare
Brachypodium distachyon
Zea mays
Sorghum bicolor
Setaria italica
Musa acuminata
Selaginella moellendorffii
Physcomitrella patens
Ostreococcus tauri
Ostreococcus lucimarinus CCE9901
Chlamydomonas reinhardtii
Cyanidioschyzon merolae
Encephalitozoon hellem ATCC 50504
Encephalitozoon intestinalis ATCC 50506
Encephalitozoon cuniculi GB M1
Puccinia graminis
Puccinia triticina
Ustilago maydis
Malassezia globosa CBS 7966
Cryptococcus gattii WM276
Cryptococcus neoformans var. neoformans JEC21
Cryptococcus neoformans var. neoformans B 3501A
Phanerochaete chrysosporium
Schizophyllum commune H4 8
Coprinopsis cinerea okayama7_130
Laccaria bicolor S238N H82
Schizosaccharomyces japonicus yFS275
Schizosaccharomyces pombe
Clavispora lusitaniae ATCC 42720
Yarrowia lipolytica
Candida tropicalis MYA 3404
Candida dubliniensis CD36
Lodderomyces elongisporus NRRL YB 4239
Scheffersomyces stipitis CBS 6054
Meyerozyma guilliermondii ATCC 6260
Pichia guilliermondii
Lachancea thermotolerans
Kluyveromyces lactis NRRL Y 1140
Saccharomyces cerevisiae
Zygosaccharomyces rouxii
Komagataella pastoris GS115
Vanderwaltozyma polyspora DSM 70294
Torulaspora delbrueckii
Candida glabrata CBS 138
Tetrapisispora phaffii CBS 4417
Naumovozyma dairenensis CBS 421
Naumovozyma castellii CBS 4309
Eremothecium cymbalariae DBVPG_7215
Eremothecium gossypii
Tuber melanosporum
Zymoseptoria tritici
Phaeosphaeria nodorum
Leptosphaeria maculans
Pyrenophora tritici repentis Pt 1C BFP
Pyrenophora teres f. teres 0 1
Botryotinia fuckeliana
Sclerotinia sclerotiorum
Verticillium albo atrum VaMs.102
Fusarium oxysporum
Hypocrea virens
Hypocrea jecorina
Nectria haematococca
Gibberella moniliformis
Gibberella zeae PH 1
Gibberella zeae
Gaeumannomyces graminis
Magnaporthe grisea
Magnaporthe poae
Magnaporthe oryzae
Podospora anserina S mat
Neurospora crassa
Sordaria macrospora k hell
Myceliophthora thermophila ATCC 42464
Chaetomium globosum CBS 148.51
Thielavia terrestris NRRL 8126
Uncinocarpus reesii 1704
Ajellomyces capsulatus NAm1
Ajellomyces dermatitidis SLH14081
Paracoccidioides sp. lutzii Pb01
Coccidioides posadasii C735 delta SOWgp
Coccidioides immitis RS
Trichophyton rubrum CBS 118892
Trichophyton verrucosum HKI 0517
Arthroderma benhamiae CBS 112371
Arthroderma gypseum CBS 118893
Arthroderma otae CBS 113480
Talaromyces stipitatus ATCC 10500
Neosartorya fischeri
Emericella nidulans
Penicillium chrysogenum Wisconsin 54 1255
Penicillium marneffei ATCC 18224
Aspergillus clavatus
Aspergillus flavus
Aspergillus oryzae
Aspergillus niger
Aspergillus fumigatus
Aspergillus terreus
Monosiga brevicollis MX1
Amphimedon queenslandica
Trichoplax adhaerens
Nematostella vectensis
Hydra magnipapillata
Schistosoma mansoni
Trichinella spiralis
Pristionchus pacificus
Caenorhabditis brenneri
Caenorhabditis briggsae
Caenorhabditis remanei
Caenorhabditis japonica
Caenorhabditis elegans
Ixodes scapularis
Daphnia pulex
Pediculus humanus
Acyrthosiphon pisum
Tribolium castaneum
Nasonia vitripennis
Apis mellifera
Atta cephalotes
Bombyx mori
Danaus plexippus
Heliconius melpomene
Culex quinquefasciatus
Aedes aegypti
Anopheles gambiae
Anopheles darlingi
Drosophila virilis
Drosophila grimshawi
Drosophila willistoni
Drosophila pseudoobscura
Drosophila ananassae
Drosophila melanogaster
Drosophila yakuba
Strongylocentrotus purpuratus
Branchiostoma floridae
Ciona intestinalis
Ciona savignyi
Petromyzon marinus
Danio rerio
Gadus morhua
Oreochromis niloticus
Takifugu rubripes
Tetraodon nigroviridis
Gasterosteus aculeatus
Xiphophorus maculatus
Oryzias latipes
Latimeria chalumnae
Xenopus (Silurana) tropicalis
Pelodiscus sinensis
Anolis carolinensis
Taeniopygia guttata
Gallus gallus
Meleagris gallopavo
Ornithorhynchus anatinus
Monodelphis domestica
Sarcophilus harrisii
Macropus eugenii
Dasypus novemcinctus
Loxodonta africana
Echinops telfairi
Procavia capensis
Myotis lucifugus
Pteropus vampyrus
Sus scrofa
Tursiops truncatus
Bos taurus
Equus caballus
Felis catus
Canis lupus familiaris
Mustela putorius furo
Ailuropoda melanoleuca
Oryctolagus cuniculus
Cavia porcellus
Ictidomys tridecemlineatus
Dipodomys ordii
Rattus norvegicus
Mus musculus
Tupaia belangeri
Otolemur garnettii
Microcebus murinus
Tarsius syrichta
Callithrix jacchus
Macaca mulatta
Nomascus leucogenys
Pongo abelii
Gorilla gorilla
Pan troglodytes
Homo sapiens
Perkinsus marinus ATCC 50983
Tetrahymena thermophila
Paramecium tetraurelia strain d4 2
Neospora caninum Liverpool
Toxoplasma gondii
Cryptosporidium muris RN66
Cryptosporidium parvum Iowa II
Cryptosporidium hominis TU502
Theileria annulata strain Ankara
Theileria parva strain Muguga
Plasmodium falciparum
Plasmodium knowlesi
Plasmodium vivax
Plasmodium chabaudi
Plasmodium yoelii yoelii 17XNL
Plasmodium berghei
Phaeodactylum tricornutum
Thalassiosira pseudonana
Pythium ultimum
Albugo laibachii
Hyaloperonospora arabidopsidis
Phytophthora ramorum
Phytophthora infestans
Phytophthora sojae
Trypanosoma brucei
Trypanosoma cruzi strain CL Brener
Leishmania braziliensis MHOM BR 75 M2904
Leishmania mexicana MHOM GT 2001 U1103
Leishmania major
Leishmania donovani
Leishmania infantum JPCM5
Entamoeba histolytica
Entamoeba dispar SAW760
Dictyostelium discoideum
Dictyostelium purpureum
Trichomonas vaginalis G3
Trichomonas vaginalis
Naegleria gruberi
Giardia lamblia ATCC 50803
OG presence
OG absence
Inferred root
Dscore = 0.993
Pvalue = 7.44e−06
AdjPvalue = 8.85e−04

NOG66170

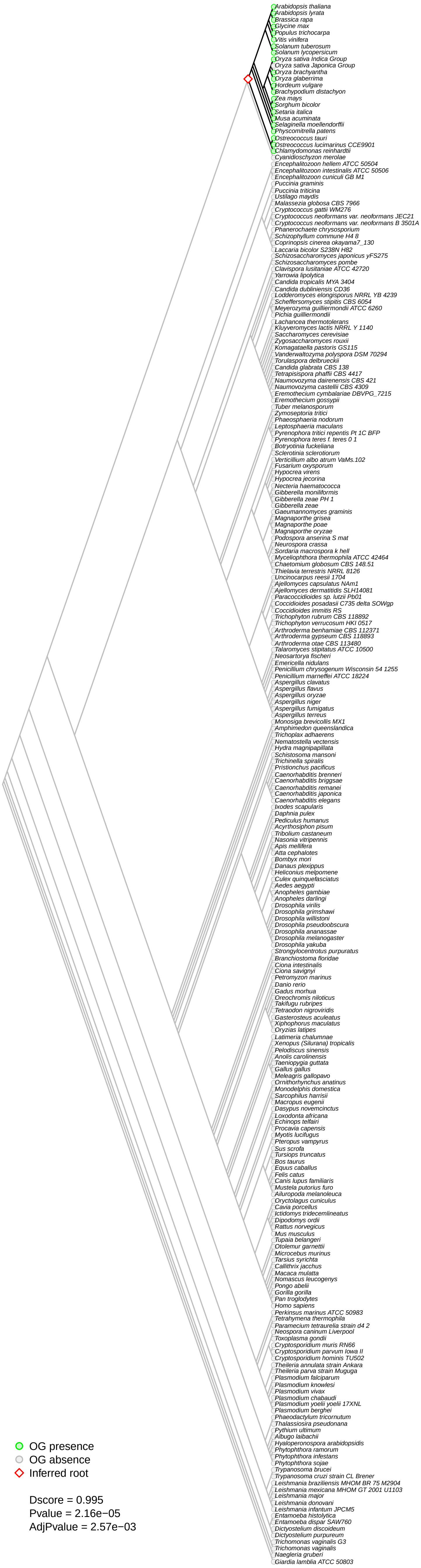
Arabidopsis thaliana
Arabidopsis lyrata
Brassica rapa
Glycine max
Populus trichocarpa
Vitis vinifera
Solanum tuberosum
Solanum lycopersicum
Oryza sativa Indica Group
Oryza sativa Japonica Group
Oryza brachyantha
Oryza glaberrima
Hordeum vulgare
Brachypodium distachyon
Zea mays
Sorghum bicolor
Setaria italica
Musa acuminata
Selaginella moellendorffii
Physcomitrella patens
Ostreococcus tauri
Ostreococcus lucimarinus CCE9901
Chlamydomonas reinhardtii
Cyanidioschyzon merolae
Encephalitozoon hellem ATCC 50504
Encephalitozoon intestinalis ATCC 50506
Encephalitozoon cuniculi GB M1
Puccinia graminis
Puccinia triticina
Ustilago maydis
Malassezia globosa CBS 7966
Cryptococcus gattii WM276
Cryptococcus neoformans var. neoformans JEC21
Cryptococcus neoformans var. neoformans B 3501A
Phanerochaete chrysosporium
Schizophyllum commune H4 8
Coprinopsis cinerea okayama7_130
Laccaria bicolor S238N H82
Schizosaccharomyces japonicus yFS275
Schizosaccharomyces pombe
Clavispora lusitaniae ATCC 42720
Yarrowia lipolytica
Candida tropicalis MYA 3404
Candida dubliniensis CD36
Lodderomyces elongisporus NRRL YB 4239
Scheffersomyces stipitis CBS 6054
Meyerozyma guilliermondii ATCC 6260
Pichia guilliermondii
Lachancea thermotolerans
Kluyveromyces lactis NRRL Y 1140
Saccharomyces cerevisiae
Zygosaccharomyces rouxii
Komagataella pastoris GS115
Vanderwaltozyma polyspora DSM 70294
Torulaspora delbrueckii
Candida glabrata CBS 138
Tetrapisispora phaffii CBS 4417
Naumovozyma dairenensis CBS 421
Naumovozyma castellii CBS 4309
Eremothecium cymbalariae DBVPG_7215
Eremothecium gossypii
Tuber melanosporum
Zymoseptoria tritici
Phaeosphaeria nodorum
Leptosphaeria maculans
Pyrenophora tritici repentis Pt 1C BFP
Pyrenophora teres f. teres 0 1
Botryotinia fuckeliana
Sclerotinia sclerotiorum
Verticillium albo atrum VaMs.102
Fusarium oxysporum
Hypocrea virens
Hypocrea jecorina
Nectria haematococca
Gibberella moniliformis
Gibberella zeae PH 1
Gibberella zeae
Gaeumannomyces graminis
Magnaporthe grisea
Magnaporthe poae
Magnaporthe oryzae
Podospora anserina S mat
Neurospora crassa
Sordaria macrospora k hell
Myceliophthora thermophila ATCC 42464
Chaetomium globosum CBS 148.51
Thielavia terrestris NRRL 8126
Uncinocarpus reesii 1704
Ajellomyces capsulatus NAm1
Ajellomyces dermatitidis SLH14081
Paracoccidioides sp. lutzii Pb01
Coccidioides posadasii C735 delta SOWgp
Coccidioides immitis RS
Trichophyton rubrum CBS 118892
Trichophyton verrucosum HKI 0517
Arthroderma benhamiae CBS 112371
Arthroderma gypseum CBS 118893
Arthroderma otae CBS 113480
Talaromyces stipitatus ATCC 10500
Neosartorya fischeri
Emericella nidulans
Penicillium chrysogenum Wisconsin 54 1255
Penicillium marneffei ATCC 18224
Aspergillus clavatus
Aspergillus flavus
Aspergillus oryzae
Aspergillus niger
Aspergillus fumigatus
Aspergillus terreus
Monosiga brevicollis MX1
Amphimedon queenslandica
Trichoplax adhaerens
Nematostella vectensis
Hydra magnipapillata
Schistosoma mansoni
Trichinella spiralis
Pristionchus pacificus
Caenorhabditis brenneri
Caenorhabditis briggsae
Caenorhabditis remanei
Caenorhabditis japonica
Caenorhabditis elegans
Ixodes scapularis
Daphnia pulex
Pediculus humanus
Acyrthosiphon pisum
Tribolium castaneum
Nasonia vitripennis
Apis mellifera
Atta cephalotes
Bombyx mori
Danaus plexippus
Heliconius melpomene
Culex quinquefasciatus
Aedes aegypti
Anopheles gambiae
Anopheles darlingi
Drosophila virilis
Drosophila grimshawi
Drosophila willistoni
Drosophila pseudoobscura
Drosophila ananassae
Drosophila melanogaster
Drosophila yakuba
Strongylocentrotus purpuratus
Branchiostoma floridae
Ciona intestinalis
Ciona savignyi
Petromyzon marinus
Danio rerio
Gadus morhua
Oreochromis niloticus
Takifugu rubripes
Tetraodon nigroviridis
Gasterosteus aculeatus
Xiphophorus maculatus
Oryzias latipes
Latimeria chalumnae
Xenopus (Silurana) tropicalis
Pelodiscus sinensis
Anolis carolinensis
Taeniopygia guttata
Gallus gallus
Meleagris gallopavo
Ornithorhynchus anatinus
Monodelphis domestica
Sarcophilus harrisii
Macropus eugenii
Dasypus novemcinctus
Loxodonta africana
Echinops telfairi
Procavia capensis
Myotis lucifugus
Pteropus vampyrus
Sus scrofa
Tursiops truncatus
Bos taurus
Equus caballus
Felis catus
Canis lupus familiaris
Mustela putorius furo
Ailuropoda melanoleuca
Oryctolagus cuniculus
Cavia porcellus
Ictidomys tridecemlineatus
Dipodomys ordii
Rattus norvegicus
Mus musculus
Tupaia belangeri
Otolemur garnettii
Microcebus murinus
Tarsius syrichta
Callithrix jacchus
Macaca mulatta
Nomascus leucogenys
Pongo abelii
Gorilla gorilla
Pan troglodytes
Homo sapiens
Perkinsus marinus ATCC 50983
Tetrahymena thermophila
Paramecium tetraurelia strain d4 2
Neospora caninum Liverpool
Toxoplasma gondii
Cryptosporidium muris RN66
Cryptosporidium parvum Iowa II
Cryptosporidium hominis TU502
Theileria annulata strain Ankara
Theileria parva strain Muguga
Plasmodium falciparum
Plasmodium knowlesi
Plasmodium vivax
Plasmodium chabaudi
Plasmodium yoelii yoelii 17XNL
Plasmodium berghei
Phaeodactylum tricornutum
Thalassiosira pseudonana
Pythium ultimum
Albugo laibachii
Hyaloperonospora arabidopsidis
Phytophthora ramorum
Phytophthora infestans
Phytophthora sojae
Trypanosoma brucei
Trypanosoma cruzi strain CL Brener
Leishmania braziliensis MHOM BR 75 M2904
Leishmania mexicana MHOM GT 2001 U1103
Leishmania major
Leishmania donovani
Leishmania infantum JPCM5
Entamoeba histolytica
Entamoeba dispar SAW760
Dictyostelium discoideum
Dictyostelium purpureum
Trichomonas vaginalis G3
Trichomonas vaginalis
Naegleria gruberi
Giardia lamblia ATCC 50803
OG presence
OG absence
Inferred root
Dscore = 0.995
Pvalue = 2.16e−05
AdjPvalue = 2.57e−03

NOG75667

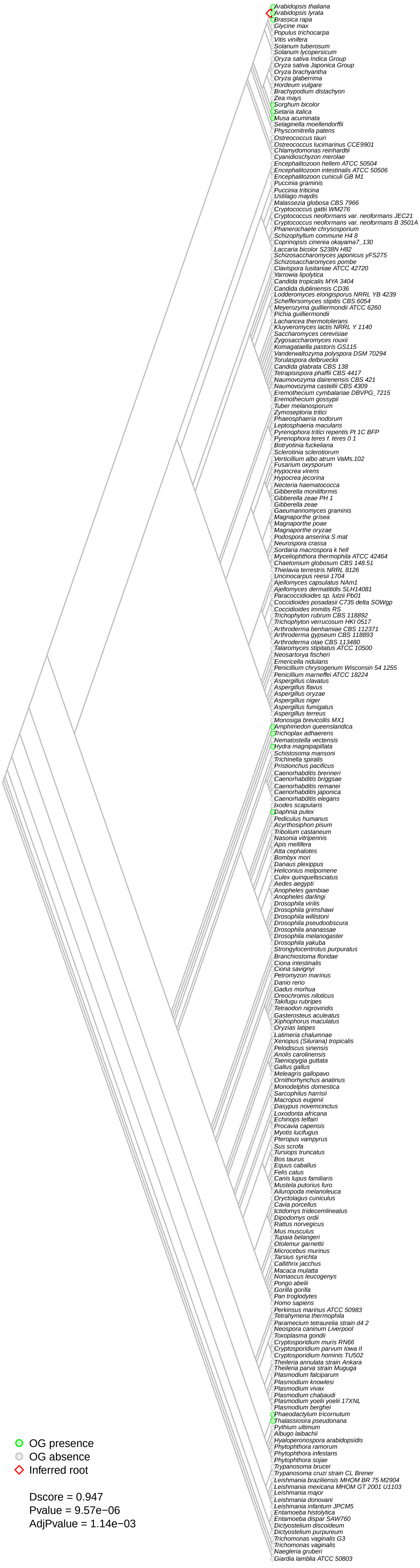
Arabidopsis thaliana
Arabidopsis lyrata
Brassica rapa
Glycine max
Populus trichocarpa
Vitis vinifera
Solanum tuberosum
Solanum lycopersicum
Oryza sativa Indica Group
Oryza sativa Japonica Group
Oryza brachyantha
Oryza glaberrima
Hordeum vulgare
Brachypodium distachyon
Zea mays
Sorghum bicolor
Setaria italica
Musa acuminata
Selaginella moellendorffii
Physcomitrella patens
Ostreococcus tauri
Ostreococcus lucimarinus CCE9901
Chlamydomonas reinhardtii
Cyanidioschyzon merolae
Encephalitozoon hellem ATCC 50504
Encephalitozoon intestinalis ATCC 50506
Encephalitozoon cuniculi GB M1
Puccinia graminis
Puccinia triticina
Ustilago maydis
Malassezia globosa CBS 7966
Cryptococcus gattii WM276
Cryptococcus neoformans var. neoformans JEC21
Cryptococcus neoformans var. neoformans B 3501A
Phanerochaete chrysosporium
Schizophyllum commune H 8
Coprinopsis cinerea okayama7_130
Laccaria bicolor S238N H82
Schizosaccharomyces japonicus yFS275
Schizosaccharomyces pombe
Clavispora lusitaniae ATCC 42720
Yarrowia lipolytica
Candida tropicalis MYA 3404
Candida dubliniensis CD36
Lodderomyces elongisporus NRRL YB 4239
Scheffersomyces stipitis CBS 6054
Meyerozyma guilliermondii ATCC 6260
Pichia guilliermondii
Lachancea thermotolerans
Kluyveromyces lactis NRRL Y 1140
Saccharomyces cerevisiae
Zygosaccharomyces rouxii
Komagataella pastoris GS115
Vanderwaltozyma polyspora DSM 70294
Torulaspora delbrueckii
Candida glabrata CBS 138
Tetrapisispora phaffii CBS 4417
Naumovozyma dairenensis CBS 421
Naumovozyma castellii CBS 4309
Eremothecium cymbalariae DBVPG_7215
Eremothecium gossypii
Tuber melanosporum
Zymoseptoria tritici
Phaeosphaeria nodorum
Leptosphaeria maculans
Pyrenophora tritici repentis Pt 1C BFP
Pyrenophora teres f. teres 0 1
Botryotinia fuckeliana
Sclerotinia sclerotiorum
Verticillium albo atrum VaMs.102
Fusarium oxysporum
Hypocrea virens
Hypocrea jecorina
Nectria haematococca
Gibberella moniliformis
Gibberella zeae PH 1
Gibberella zeae
Gaeumannomyces graminis
Magnaporthe grisea
Magnaporthe poae
Magnaporthe oryzae
Podospora anserina S mat
Neurospora crassa
Sordaria macrospora k hell
Myceliophthora thermophila ATCC 42464
Chaetomium globosum CBS 148.51
Thielavia terrestris NRRL 8126
Uncinocarpus reesii 1704
Ajellomyces capsulatus NAm1
Ajellomyces dermatitidis SLH14081
Paracoccidioides sp. lutzii Pb01
Coccidioides posadasii C735 delta SOWgp
Coccidioides immitis RS
Trichophyton rubrum CBS 118892
Trichophyton verrucosum HKI 0517
Arthroderma benhamiae CBS 112371
Arthroderma gypseum CBS 118893
Arthroderma otae CBS 113480
Talaromyces stipitatus ATCC 10500
Neosartorya fischeri
Emericella nidulans
Penicillium chrysogenum Wisconsin 54 1255
Penicillium marneffei ATCC 18224
Aspergillus clavatus
Aspergillus flavus
Aspergillus oryzae
Aspergillus niger
Aspergillus fumigatus
Aspergillus terreus
Monosiga brevicollis MX1
Amphimedon queenslandica
Trichoplax adhaerens
Nematostella vectensis
Hydra magnipapillata
Schistosoma mansoni
Trichinella spiralis
Pristionchus pacificus
Caenorhabditis brenneri
Caenorhabditis briggsae
Caenorhabditis remanei
Caenorhabditis japonica
Caenorhabditis elegans
Ixodes scapularis
Daphnia pulex
Pediculus humanus
Acyrthosiphon pisum
Tribolium castaneum
Nasonia vitripennis
Apis mellifera
Atta cephalotes
Bombyx mori
Danaus plexippus
Heliconius melpomene
Culex quinquefasciatus
Aedes aegypti
Anopheles gambiae
Anopheles darlingi
Drosophila virilis
Drosophila grimshawi
Drosophila willistoni
Drosophila pseudoobscura
Drosophila ananassae
Drosophila melanogaster
Drosophila yakuba
Strongylocentrotus purpuratus
Branchiostoma floridae
Ciona intestinalis
Ciona savignyi
Petromyzon marinus
Danio rerio
Gadus morhua
Oreochromis niloticus
Takifugu rubripes
Tetraodon nigroviridis
Gasterosteus aculeatus
Xiphophorus maculatus
Oryzias latipes
Latimeria chalumnae
Xenopus (Silurana) tropicalis
Pelodiscus sinensis
Anolis carolinensis
Taeniopygia guttata
Gallus gallus
Meleagris gallopavo
Ornithorhynchus anatinus
Monodelphis domestica
Sarcophilus harrisii
Macropus eugenii
Dasypus novemcinctus
Loxodonta africana
Echinops telfairi
Procavia capensis
Myotis lucifugus
Pteropus vampyrus
Sus scrofa
Tursiops truncatus
Bos taurus
Equus caballus
Felis catus
Canis lupus familiaris
Mustela putorius furo
Ailuropoda melanoleuca
Oryctolagus cuniculus
Cavia porcellus
Ictidomys tridecemlineatus
Dipodomys ordii
Rattus norvegicus
Mus musculus
Tupaia belangeri
Otolemur garnettii
Microcebus murinus
Tarsius syrichta
Callithrix jacchus
Macaca mulatta
Nomascus leucogenys
Pongo abelii
Gorilla gorilla
Pan troglodytes
Homo sapiens
Perkinsus marinus ATCC 50983
Tetrahymena thermophila
Paramecium tetraurelia strain d4 2
Neospora caninum Liverpool
Toxoplasma gondii
Cryptosporidium muris RN66
Cryptosporidium parvum Iowa II
Cryptosporidium hominis TU502
Theileria annulata strain Ankara
Theileria parva strain Muguga
Plasmodium falciparum
Plasmodium knowlesi
Plasmodium vivax
Plasmodium chabaudi
Plasmodium yoelii yoelii 17XNL
Plasmodium berghei
Phaeodactylum tricornutum
Thalassiosira pseudonana
Pythium ultimum
Albugo laibachii
Hyaloperonospora arabidopsidis
Phytophthora ramorum
Phytophthora infestans
Phytophthora sojae
Trypanosoma brucei
Trypanosoma cruzi strain CL Brener
Leishmania braziliensis MHOM BR 75 M2904
Leishmania mexicana MHOM GT 2001 U1103
Leishmania major
Leishmania donovani
Leishmania infantum JPCM5
Entamoeba histolytica
Entamoeba dispar SAW760
Dictyostelium discoideum
Dictyostelium purpureum
Trichomonas vaginalis G3
Trichomonas vaginalis
Naegleria gruberi
Giardia lamblia ATCC 50803
OG presence
OG absence
Inferred root
Dscore = 0.947
Pvalue = 9.57e−06
AdjPvalue = 1.14e−03
